# Child Mortality Estimation: A Comparison of UN IGME and IHME Estimates of Levels and Trends in Under-Five Mortality Rates and Deaths

**DOI:** 10.1371/journal.pmed.1001288

**Published:** 2012-08-28

**Authors:** Leontine Alkema, Danzhen You

**Affiliations:** 1 Department of Statistics and Applied Probability and Saw Swee Hock School of Public Health, National University of Singapore, Singapore; 2 United Nations Children's Fund, New York, New York, United States of America; Umeå Centre for Global Health Research, Umeå University, Sweden

## Abstract

**Background:**

Millennium Development Goal 4 calls for a reduction in the under-five mortality rate (U5MR) by two-thirds between 1990 and 2015. In 2011, estimates were published by the United Nations Inter-agency Group for Child Mortality Estimation (UN IGME) and the Institute for Health Metrics and Evaluation (IHME). The difference in the U5MR estimates produced by the two research groups was more than 10% and corresponded to more than ten deaths per 1,000 live births for 10% of all countries in 1990 and 20% of all countries in 2010, which can lead to conflicting conclusions with respect to countries' progress. To understand what caused the differences in estimates, we summarised differences in underlying data and modelling approaches used by the two groups, and analysed their effects.

**Methods and Findings:**

UN IGME and IHME estimation approaches differ with respect to the construction of databases and the pre-processing of data, trend fitting procedures, inclusion and exclusion of data series, and additional adjustment procedures. Large differences in U5MR estimates between the UN IGME and the IHME exist in countries with conflicts or civil unrest, countries with high HIV prevalence, and countries where the underlying data used to derive the estimates were different, especially if the exclusion of data series differed between the two research groups. A decomposition of the differences showed that differences in estimates due to using different data (inclusion of data series and pre-processing of data) are on average larger than the differences due to using different trend fitting methods.

**Conclusions:**

Substantial country-specific differences between UN IGME and IHME estimates for U5MR and the number of under-five deaths exist because of various differences in data and modelling assumptions used. Often differences are illustrative of the lack of reliable data and likely to decrease as more data become available. Improved transparency on methods and data used will help to improve understanding about the drivers of the differences.

*Please see later in the article for the Editors' Summary.*

## Abstract 2

Leontine Alkema and Danzhen You compare and summarize differences in underlying data and modelling approaches used by two key groups who publish data on global under-5 mortality rates

## Editors' Summary

**Background:**

In 2010, more than seven million children died before they reached their fifth birthday, and the global under-five mortality rate (also denoted in the literature as U5MR and 5q0) was 57 deaths per 1,000 live births. Most deaths before the age of five years occur in developing countries (about half occur in just five countries—India, Nigeria, the Democratic Republic of the Congo, Pakistan, and China), and most are caused by preventable or treatable diseases such as pneumonia, diarrhea, and malaria. Faced with this largely avoidable loss of young lives, in 1990, the United Nations (UN) World Summit for Children pledged to improve the survival of children. Later, in 2000, world leaders set a target of reducing under-five mortality to one-third of its 1990 level (12 million) by 2015, as Millennium Development Goal 4 (MDG 4). This goal, together with seven others, is designed to improve the social, economic, and health conditions in the world's poorest countries.

**Why Was This Study Done?:**

Although progress towards MDG 4 is accelerating, MDG 4 is unlikely to be reached. It is important, therefore, to know which countries are making poor progress towards MDG 4 so that extra resources can be concentrated in these areas. To monitor both national and global progress, accurate, up-to-date estimates of U5MR are essential. The first step in estimating U5MR is the collection of data on child deaths, usually through vital registration systems (which record all births and deaths) in developed countries and through surveys that ask women about their living and dead children in developing countries. Country-specific U5MR estimates that are comparable over time and across countries are obtained from these data using a statistical process called trend fitting. Two groups—the UN Inter-agency Group for Child Mortality Estimation (UN IGME) and the Institute for Health Metrics and Evaluation (IHME)—recently published new estimates of the levels and trends in U5MR and under-five deaths across the world. However, their estimates differ somewhat and, for some countries, disagree on the progress being made towards MDG 4. Here, the researchers examine the differences in the underlying data and the trend fitting approaches used by the UN IGME and the IHME to try to understand why their estimates are different.

**What Did the Researchers Do and Find?:**

The researchers first compared the estimates produced by the two groups. From 1990 to 2010, the UN IGME's global estimates of U5MR and under-five deaths were consistently slightly higher than those of the IHME. For example, in 2010, the UN IGME and IMHE estimates of U5MR were 56.7 and 53.9 deaths per 1,000 births, respectively. However, although the global estimates from the two groups were broadly similar, there were important differences between the two sets of estimates at the country level, particularly in countries where there was conflict or civil unrest (for example, Somalia) or high HIV prevalence. The researchers then examined the data used by the two groups to estimate under-five deaths and U5MR, the method used for U5MR trend fitting, and additional adjustment procedures (for example, the UN IGME incorporates feedback from experts and country consultations in its estimates). The UN IGME and IHME estimation approaches included differences in all of these areas, but differences in the data used caused on average larger differences in the estimates than the use of different trend fitting methods did.

**What Do These Findings Mean?:**

These findings show that the substantial country-specific differences between UN IGME and IHME estimates for U5MR and the number of under-five deaths are the result of several differences between the data and trend fitting methods used by the two groups. In particular, the findings indicate that the lack of reliable data in many developing countries, especially those where there is civil unrest or ongoing conflicts, is often responsible for differences in estimates. These differences should, therefore, decrease as more reliable data become available. For now, though, the differences between the UN IGME and IHME national estimates of child mortality may cause confusion about the true extent of progress towards MDG 4 and could foster policy inactivity if the reasons for the discrepancies are not made clear. The researchers call, therefore, for more transparency on the methods and data used in the estimation of U5MR and for a concerted effort by governments, UN agencies, and non-governmental organizations to improve the collection of reliable data on child deaths.

**Additional Information:**

Please access these websites via the online version of this summary at http://dx.doi.org/10.1371/journal.pmed.1001288.

- This paper is part of a collection of papers on Child Mortality Estimation Methods published in *PLOS Medicine*
- The United Nations Childrens Fund (UNICEF) works for children's rights, survival, development, and protection around the world; it provides information on Millennium Development Goal 4, and its Childinfo website provides detailed statistics about child survival and health, including a description of the UN Inter-agency Group for Child Mortality Estimation and a link to its database; the 2011 UN IGME report on Levels and Trends in Child Mortality is available
- The Institute for Health Metrics and Evaluation website includes a summary of their 2011 analysis of U5MR and under-five deaths
- The World Health Organization also has information about Millennium Development Goal 4 and provides estimates of child mortality rates (some information in several languages)
- Further information about the Millennium Development Goals is available

## Introduction

The Millennium Development Goal 4 (MDG 4) calls for a two-thirds reduction in the under-five mortality rate (U5MR; also denoted in the literature as _5_ *q*_0_) between 1990 and 2015. As global momentum and investment for accelerating child survival grows and only three years remain to achieve MDG 4, monitoring progress at the global and country level has become even more critical. In September 2011, the United Nations Inter-agency Group for Child Mortality Estimation (UN IGME) published an analysis to track trends in child mortality [1]. The estimated trends were the result of an analysis by the UN IGME agencies, in collaboration with academic scholars, and a country consultation process. In the same month, the Institute for Health Metrics and Evaluation (IHME), an academic institution, also published an analysis of trends in child mortality [2]. In this paper, we summarise differences between the UN IGME and the academic IHME estimates for U5MR and the number of under-five deaths at the country level, for the 186 countries for which both groups published estimates. This analysis is motivated by observed differences in country estimates by the two groups, as well as by recent discussions about the construction of global health estimates [3],[4]. In the Methods, we summarise the construction of databases and modelling approaches that are used by the two groups to estimate U5MR and the number of under-five deaths, and introduce a method to decompose differences in the estimates of U5MR and number of deaths. In the Results, we give an overview of the differences in country-specific estimates, explain the differences between estimates for selected countries, and discuss the decomposition of differences.

## Methods

### Summary of Differences in Databases and Modelling Approaches

Data on U5MR are often obtained from vital registration (VR) systems, surveys, or censuses. These data sources either record recent births and deaths, or collect information on child mortality retrospectively using so-called summary or full birth histories. Summary birth histories record the number of births that a woman has had, together with the number of children that are still alive, while full birth histories are a complete listing of all births and the age of death of any children who died before the age of five. Measurements are constructed from reported births and deaths below the age of 5 y (from VR data, information on household births and deaths during the last 12 mo prior to a census, and retrospective information from full birth histories), or from models applied to information from summary birth histories (referred to as indirect estimates). The availability of data varies over time within countries and across countries, and observations are subject to sampling and non-sampling errors. To obtain country-specific U5MR estimates that are comparable across time within countries, as well as across countries, trend fitting procedures are used. In this section, we explain the differences in the databases and all modelling steps involved in constructing U5MR estimates between the UN IGME and the IHME.

#### Databases

The differences in the underlying data used by the UN IGME and the IHME can be categorised into the following groups: completeness of database (where completeness refers to the inclusion of available data sources in the database), calculation of data from surveys and censuses, treatment of data from incomplete VR systems, and exclusion criteria applied to data series during curve fitting procedures. Differences in the underlying data used are summarised in Table 1.

**Table 1:** Overview of differences in modelling approach and data used by the UN IGME and the IHME for estimating the U5MR and the number of under-five deaths.

Category	UN IGME	IHME
**Estimation method**		
Default method	Fit loess smoother to U5MR observations	GPR
Countries with conflicts, natural disasters, or limited observations of dubious quality	Modified estimation method (changing the smoothing parameter α to better capture trends or using an adjusted method) based on expert opinion and evidence from other sources such as health intervention and coverage indicators	Data from selected periods of conflict or natural disaster are excluded when fitting the GPR; results are adjusted afterwards
**VR data**	VR data are adjusted for 12 European countries^a^; incomplete VR systems are not used except for two countries, where bias parameter is included in loess fit	Bias parameters included for VRs from selected low- and middle-income countries
**Data from surveys and censuses**		
Exclusion of data sources/observations	Surveys are excluded if their values are consistently below those of other data sources, or if data quality issues have been reported	Outliers excluded based on “broad discussion” of previous mortality estimates
Indirect estimates from surveys and censuses	UN Manual X methodology applied to aggregate data (excludes recent points based on reports of women 15–19 y and 20–24 y)	Methods based on [6]; update of parameter estimates and derivation of indirect estimates from maternal cohort data
Direct estimates from DHS surveys	Surveys are not pooled; estimates are based on various periods; each estimate is included once	All surveys within a country are pooled; 2-y estimates are constructed; each estimate is included twice
**Countries with high HIV prevalence**	Observations and estimation procedures are adjusted to account for selection bias resulting from HIV deaths	No adjustment of data or modelling procedure
**Estimation of under-five deaths**	Central mortality rates applied to estimated populations (probability of dying converted to central mortality rate); breakdown into ages 0 and 1–4 y	Cohort deaths are “allocated” to particular age groups based on cohort size and exact exposure to mortality risk in different periods; each yearly birth cohort is divided into 52 birth-week cohorts and followed to age 5 y

a There are concerns about the incompleteness of early infant mortality data from civil registration in some European countries. The problem is not necessarily derived from a dysfunction of the civil registration system; it is often caused by different definitions being used for live births, which influences the counting of early infants. The UN IGME carried out an analysis of the ratio of early neonatal (under 7-d) deaths to total neonatal deaths. The average value of this ratio for Western European countries was 0.77, with few values below 0.7. A statistical analysis of this ratio for available country-years found that the ratio was significantly lower than the Western European average for the following countries: Belarus, Bulgaria, Czech Republic, Estonia, Greece, Hungary, Latvia, Lithuania, Romania, Russian Federation, Slovakia, and Spain. In only four countries did this ratio change significantly over time, and in all cases it was decreasing, not increasing. Based on this analysis, it was decided to apply a 10% upward adjustment to under-five mortality for Belarus, Hungary, and Lithuania, and a 20% adjustment for the other countries, including the Russian Federation. In all cases, a single country-specific correction factor was applied to the entire time series.

The UN IGME database, which contains the underlying data used for estimation, is publicly available at http://www.childmortality.org (downloaded October 2011). The comparison of the completeness of the UN IGME's and the IHME's databases was difficult because the IHME's database is not publicly available (despite requested access, see Discussion). We compared the databases on the basis of the IHME's graphs for each country in the online appendix of [2] and found that the data sources for the UN IGME and the IHME estimates are similar for the majority of countries, but differences do exist and will be pointed out in the Results.

For data sources included in the database, the methods used to calculate the estimates of U5MR used by the UN IGME and the IHME were different. For summary birth history data, the UN IGME used the indirect methods outlined in the United Nation's Manual X [5] (excluding recent points based on reports of women aged 15–19 y and 20–24 y because of selection bias), whereas the IHME used an updated version of the methods described by Rajaratnam et al. [6] to calculate estimates.

For data from full birth histories, the UN IGME constructed estimates by combining information by calendar year on the number of deaths and surviving children for intervals of various numbers of years. Interval lengths were based on the level of child mortality, the sample size, and the variation in child mortality over time (the coefficient of variation of the estimate was used to decide what interval length to use) [7]. The IHME summarised data by 2-y intervals preceding the survey date. To construct direct U5MR estimates from data collected by Demographic and Health Surveys (DHS), the IHME combined the data from all birth histories of available DHS surveys within a country to obtain one series of estimates. We assume that these series were included twice in the 2011 database (double-counted), given that the series were included twice in the 2010 database, and no change was reported with respect to the double-counting of DHS series in the 2011 article [2]. The UN IGME calculated direct estimates for each DHS survey independently (using the birth histories of one survey at a time) to obtain one series of direct estimates per survey, and did not duplicate any series in the database.

For data from VR systems, the two research groups also used different methods to calculate U5MR, as the data are not exactly the same for most of the data points. The UN IGME and the IHME dealt differently with data from incomplete VR systems. The UN IGME adjusted VR data for 12 European countries (Belarus, Bulgaria, Czech Republic, Estonia, Greece, Hungary, Latvia, Lithuania, Romania, Russian Federation, Slovakia, and Spain) based on an assessment of early infant mortality data. The UN IGME did not include incomplete VR data from other countries. Data from incomplete VR were generally included and not adjusted in the IHME estimation process, but underreporting bias in the VR data was estimated for selected low- and middle-income countries (explained further below).

The standards to exclude data from surveys and censuses in curve fittings were different as well. The UN IGME excluded data from one data source if the data points were consistently below other data sources for all years (which indicates potential underreporting of child deaths), or if data quality issues had been reported. The IHME also identified and excluded some outlier data points but, in general, favoured the inclusion of data points rather than exclusion (as noted in [8]).

#### U5MR trend fitting

To obtain U5MR estimates that were comparable over time and across countries, both the UN IGME and the IHME fitted a curve, or equivalently, a regression model, to data from surveys, censuses, VR systems, or other data sources. However, the type of regression model that was used differed. The UN IGME used loess (locally weighted least squares) regression, whereas the IHME used Gaussian process regression (GPR) to derive trend estimates.

In the loess regression approach used by the UN IGME, the estimates of U5MR were obtained by local fits of a weighted linear regression model to the data. A smoothing parameter alpha (α) determined the range of points included in each local fit and their weights (the flexibility of the fitted trend line decreases with α). Default settings for α were based on the number of surveys and data points from VR in each country [9].

In the GPR approach used by the IHME, the first step is to obtain prior estimates of the mean trend in each country. For the majority of countries, these estimates were constructed by fitting a loess curve to the data in each country. The smoothing parameter for the loess curve was estimated using cross-validation. In the second step, trajectories of the U5MR over time were obtained based on the assumption that these trajectories are realisations of a Gaussian process, for which the mean trend is given by the prior estimates of the mean trend, and for which a Matern covariance function describes the correlation of U5MR in different years. The parameters of the covariance function were estimated using cross-validation as well. When constructing the U5MR trajectories, the variance of a U5MR observation was taken to be the sum of its sampling and non-sampling variance, where its non-sampling variance was estimated for different data sources using data from all countries. Biases of incomplete VR were estimated while constructing the trajectories; for countries with data from incomplete VR and surveys, the bias parameter was estimated by assuming that additional data sources are centred at the true U5MR. For countries with data from incomplete VR only, the bias was given a prior mean based on information on biases from a subset of countries in the region.

#### Additional adjustments

The IHME did not make any adjustments for countries with high HIV prevalence, whereas the UN IGME used a modified estimation procedure for all countries in which HIV prevalence in the general population was more than 5% at any point during the epidemic period (17 countries in total) [10]. For these countries, the UN IGME assumed that the reported data were biased because mothers who have died of AIDS are not captured to report the information of their children, who also have a higher risk of dying if they are HIV positive. To estimate the U5MR for high HIV prevalence countries, the UN IGME adjusted the survey data to account for selection bias and used slightly different steps to obtain the trend lines [9].

For countries with conflicts or limited observations of dubious quality, the UN IGME carried out adjustments based on expert opinion and evidence from other sources, such as health intervention and coverage indicators. Examples of countries where adjustments were carried out are Somalia, Angola, Afghanistan, Democratic Republic of the Congo (DRC), and North Korea. For countries with natural disasters that led to sudden increases in U5MR, such as Haiti, the UN IGME carried out an adjustment to estimate the peak in U5MR.

The IHME did not adjust estimates for countries with data from dubious quality. It did carry out adjustments during periods with natural disasters, emergencies, conflicts, or extreme events.

After initial fits were obtained for all countries by the UN IGME, data and country estimates were evaluated during the meetings of the members of the UN IGME and its Technical Advisory Group. Additional adjustments were carried out if deemed appropriate, given additional information about the country and data sources under consideration. These adjustments were carried out mainly for the countries with conflicts or limited information, or involved the exclusion of data sources, as discussed earlier. For a limited number of countries, the smoothing parameter α used in the loess trend fitting procedure was adjusted to better capture more recent trends in the data.

Furthermore, country consultations were carried out to incorporate countries' feedback on both the underlying data and the estimates before the estimates were finalised and published. The objective of the country consultation was to maximise identification of all relevant data and to allow countries to review and provide feedback on estimates; it was not, however, a country clearance process. After the consultations, estimates were revised for about 20 countries using new data the countries provided [9].

#### Estimation of under-five deaths

Besides using different estimates of U5MR, the UN IGME and the IHME also used different methods to calculate the number of under-five deaths. The UN IGME obtained the number of deaths by multiplying population by the central mortality rate, which was converted from the probability of dying. The IHME allocated deaths to cohorts at particular ages, based on cohort size and exact exposure to mortality risk in different periods. Both the UN IGME and the IHME used population estimates generated by the United Nations Population Division [11].

### Decomposition of Differences in U5MR and the Number of Deaths

We examined how much of the difference in U5MR estimates between the two research groups was caused by the IHME's use of GPR to do the trend fitting versus UN IGME's use of the loess fit, by decomposing the differences in U5MR estimates into differences “due to different use of data” and differences “due to use of GPR”. If Δ*R* represents the total difference in U5MR between the loess fit to the UN IGME data (denoted by *R*_IGME_) and the GPR fit to the IHME data (denoted by *R*_IHME_) in a particular year for a particular country, the decomposition is given by
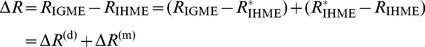
(1)where Δ*R* ^(d)^ represents the differences “due to different use of data” (the difference between the loess fit to the UN IGME data, *R*_IGME_, and the loess fit to the IHME data, 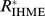), and Δ*R* ^(m)^ represents the difference “due to use of GPR” (the difference between the loess fit to the IHME data, 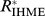, and the GPR fit to the IHME data, *R*_IHME_).

We carried out this decomposition for countries without any VR data, that had not been classified as high HIV prevalence countries, and where the UN IGME used a standard estimation procedure. We chose this set of countries to exclude differences in U5MR estimates that were caused by differences in assumptions about the potential bias of the VR and differences in adjustments for conflict countries and high HIV prevalence countries, and thus focused on differences in estimates that are caused by the fitting procedure.

To construct the decomposition, we intended to fit the loess smoother to IHME data. Unfortunately, we did not have access to the 2011 IHME database. Instead, we used the 2010 IHME dataset that was made available to us, and fitted the loess smoother to it. To get GPR estimates that were based on the same dataset, we reran the IHME estimation procedure that was used in 2010. This “2010 GPR method” differed slightly from the 2011 method used by the IHME, but the software that implemented the 2010 method was made available to us, while the updated 2011 estimation method was not (and the publication did not provide sufficient information to reproduce the updated estimation method). For a fair comparison with the UN IGME estimates (to avoid including more recent data in the UN IGME dataset), we also refitted the loess curve to the UN IGME observations after excluding all observations that were collected after 2009 (the IHME database was constructed in 2010). In total, we fitted the loess smoother and GPR in 36 countries. For both the IHME and UN IGME loess estimates, we used the country-specific smoothing parameters that were used by the UN IGME in 2011 to guarantee the use of the same smoothing parameter in both fits, to investigate the differences caused by the model fit versus the dataset used.

Similarly, to examine how much of the difference in the estimates of the number of deaths was caused by differences in the estimated U5MR, and how much was caused by the use of an alternative estimation method for the number of deaths by the IHME, we decomposed differences in the estimated number of under-five deaths into differences due to different mortality rates and differences due to the use of a different estimation method by the IHME. This decomposition was carried out for all 186 countries for the estimates in 1990, 2000, and 2010.

## Results

### Comparison of Estimates

**Figure 1:**
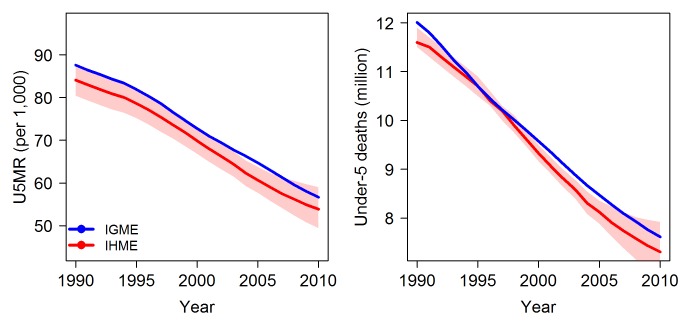
Comparison of global estimates of the U5MR and the number of under-five deaths, from 1990 to 2010. Estimates by the UN IGME (blue) and the IHME (red, with 95% uncertainty intervals represented by the shaded areas).

Figure 1 shows the estimates of the U5MR and the number of under-five deaths at the global level generated by the UN IGME and the IHME. Estimates for the years 1990 to 2010 from the two groups are used for comparison purposes (the UN IGME–generated trend estimates of under-five mortality start as early as 1960 and go up to the year 2010, whereas the IHME published estimates cover the period from 1990 to 2011). From 1990 to 2010, the UN IGME's global estimates of the U5MR are consistently higher than the IHME's estimates, but relative differences are small (4.9% on average). For 2010, the global point estimates of the U5MR by the UN IGME and the IHME are 56.7 and 53.9 deaths per 1,000 births, respectively. The UN IGME's estimate is within the IHME's uncertainty interval, which ranges from 49.4 to 59.0 deaths per 1,000 births. The difference in the estimated number of under-five deaths is about 0.3 million for the year 2010: the UN IGME estimated 7.6 million deaths, and the IHME estimated 7.3 million deaths, with an uncertainty interval of 6.8 million to 7.9 million.

**Figure 2:**
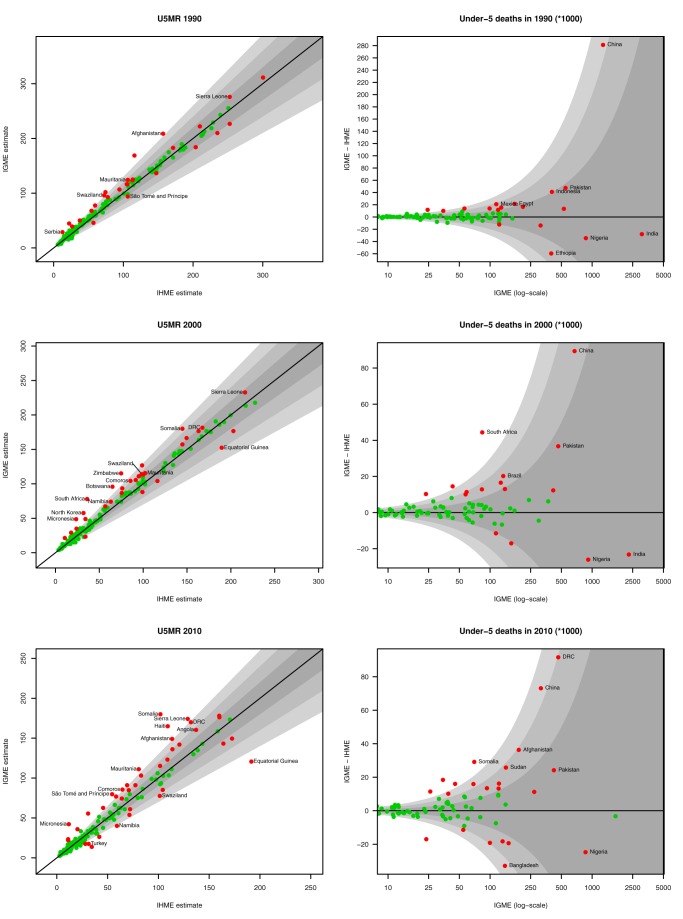
UN IGME and IHME estimates of U5MR and under-five deaths for 1990, 2000, and 2010. Left: UN IGME U5MR estimates are plotted against IHME U5MR estimates. Grey areas represent relative differences of up to ±10%, 20%, and 30%, respectively. Countries for which the estimates differ by more than ten deaths per 1,000 births are highlighted in red. Right: Difference in the number of under-five deaths between the UN IGME and IHME estimates for 1990, 2000, and 2010, plotted against the UN IGME estimate of the number of deaths (on the log scale). Grey areas represent relative differences of up to ±10%, 20%, and 30%, respectively. Countries for which the estimates differ by more than 10,000 deaths are highlighted in red.

Although the global estimates appear broadly similar, there are important differences between the two sets of estimates at the country level, particularly for the most recent years. A comparison of the estimates for all 186 countries for which both groups published estimates from 1990 to 2010 is given in Figure S1 and Table S1 and is summarised here for the years 1990, 2000, and 2010. Figure 2 gives an overview of the differences between the two sets of estimates of the U5MR and the number of under-five deaths for all countries. Large differences are found between UN IGME and IHME estimates for some countries, particularly for the reference year 2010. The number of countries with absolute differences greater than ten deaths per 1,000 live births and relative differences greater than 10% between UN IGME and IHME estimates of U5MR has increased for the reference year 2010 (38 countries, i.e., 20% of all countries) compared to for the reference years 2000 (27 countries, 15%) and 1990 (18 countries, 10%; see Table 2). Moreover, more countries with a minimum difference of ten deaths per 1,000 births have a greater than 20% difference between UN IGME and IHME estimates for the year 2010 (25 countries, 13%) compared to for the years 2000 (12 countries, 6%) and 1990 (ten countries, 5%). Countries with the largest differences in the estimated U5MR for the year 2010 include Somalia (difference of 78.1 deaths per 1,000 live births), Equatorial Guinea (70.7 deaths per 1,000 live births), Haiti (55.6 deaths per 1,000 live births), and Sierra Leone (45.1 deaths per 1,000 live births). For 13% of all countries, the UN IGME estimate for 2010 is at least 30% higher than the IHME estimate, and, vice versa, for 8% of all countries, the IHME estimate is at least 30% higher than the UN IGME estimate.

**Table 2:** The number of countries (proportion of countries) with a given absolute difference and relative difference in U5MR as estimated by the UN IGME and the IHME, in 1990, 2000, and 2010.

Year	Absolute Difference	Relative Difference, Number (Proportion) of Countries			
		0%–10%	10%–20%	20%–30%	30%+
1990	0–10 deaths/1,000 births	109 (0.59)	35 (0.19)	8 (0.04)	5 (0.03)
	10–20 deaths/1,000 births	8 (0.04)	6 (0.03)	3 (0.02)	2 (0.01)
	20+ deaths/1,000 births	1 (0.01)	2 (0.01)	3 (0.02)	2 (0.01)
2000	0–10 deaths/1,000 births	104 (0.56)	28 (0.15)	6 (0.03)	9 (0.05)
	10–20 deaths/1,000 births	4 (0.02)	13 (0.07)	0 (0)	5 (0.03)
	20+ deaths/1,000 births	0 (0)	2 (0.01)	2 (0.01)	5 (0.03)
2010	0–10 deaths/1,000 births	82 (0.44)	34 (0.18)	16 (0.09)	12 (0.06)
	10–20 deaths/1,000 births	1 (0.01)	7 (0.04)	3 (0.02)	8 (0.04)
	20+ deaths/1,000 births	0 (0)	6 (0.03)	6 (0.03)	8 (0.04)

**Figure 3:**
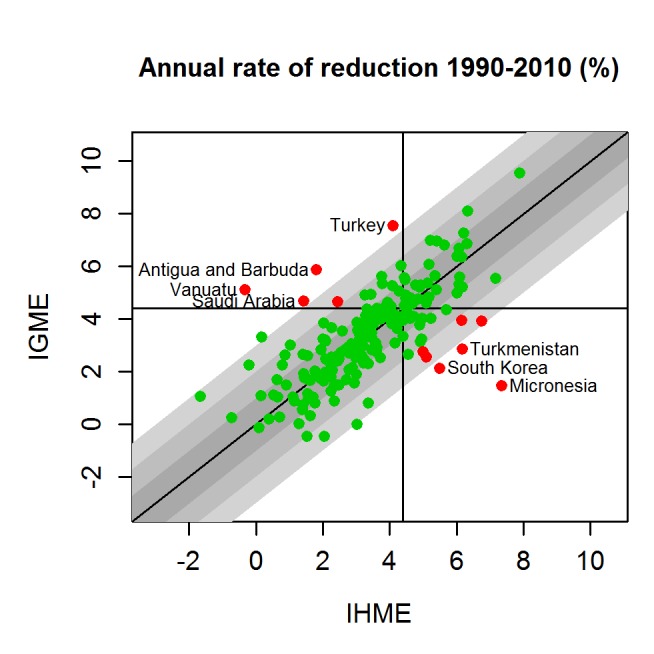
UN IGME and IHME estimates of the annual rate of reduction for 1990–2010. UN IGME estimates are plotted against IHME estimates. Grey area illustrates absolute differences of up to 1%, 2%, and 3%, respectively (absolute difference). Red indicates that the difference is at least 2% and the conclusion as to whether the country is on track to meet MDG 4 (a 4.4% annual decline) differs between the IHME and the UN IGME.

Figure 3 illustrates the differences in the estimates of the annual rate of reduction (ARR) from 1990 to 2010 for all countries. Also here, important differences in estimates exist. For 6.4% of all countries (12 countries), the difference in estimates is at least 2% (absolute), and moreover, the UN IGME and the IHME are in disagreement with respect to whether the ARR has met the MDG 4 target of 4.4%. For example, for Micronesia, the IHME estimated an ARR of over 7%, while the UN IGME estimated less than 2%. Vice versa, for Turkey, the UN IGME has estimated an ARR of over 7%, while the IHME's estimate is around 4%.

Similar to the U5MR comparison, differences in the estimated number of under-five deaths are larger for the year 2010 than for the years 1990 and 2000 (Figure 2). The number of countries with an absolute difference of more than 10,000 under-five deaths for 2010 is 22 (12% of all countries), whereas 16 (9%) and 18 (10%) countries are in this category in 2000 and 1990, respectively. As expected, many countries with substantial differences in estimated U5MR also have substantial differences in the estimated number of deaths (e.g., Somalia, DRC, Haiti, Turkey, and Viet Nam). Additionally, for some countries, the relative difference in the estimated number of deaths exceeds the relative difference in the estimated U5MR. Examples of such countries are Bangladesh and China, for which the differences in U5MR are less than ten deaths per 1,000 live births and proportional differences in U5MR are 15% and 16%, respectively, while the relative differences in the estimated number of deaths between the UN IGME and the IHME are more than 20%. For the four countries with the highest number of under-five deaths in 2010 (India, Nigeria, Pakistan, and Ethiopia), the difference in the number of deaths is less than 10% between UN IGME and IHME estimates. This difference does result in an absolute difference of more than 10,000 deaths for Nigeria, Pakistan, and Ethiopia.

### Country Examples

For the majority of countries, differences between UN IGME and IHME estimates were caused by a combination of differences in the data used and differences in modelling approach. In this section, we repeat the main differences and give examples of countries with large differences in estimates (highlighted in Figures 2 and 3) for which the differences in data used or modelling approach can be identified as one of the main drivers of the difference.

#### Completeness of the database

**Figure 4:**
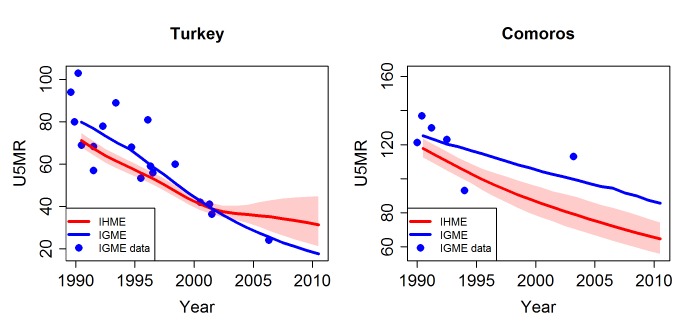
Comparison of U5MR estimates from 1990 to 2010 for examples of countries with different completeness of the databases used by the UN IGME and the IHME. Estimates by the UN IGME (blue line) and the IHME (red line, with 95% confidence intervals represented by the shaded areas). Data from the UN IGME 2011 database (IGME data) are added as blue dots.

For several countries, including Columbia, Ghana, Kenya, Malawi, Morocco, Nepal, and Occupied Palestinian Territory, the IHME database included observations from past censuses, which were not available in the UN IGME database. The UN IGME database included more recent datasets that were not included in the IHME database for some countries (e.g., the Cape Verde 2005 DHS survey, Chad 2010 Multiple Indicator Cluster Survey [MICS], Comoros 2003 census, Syria 2009 Pan Arab Population and Family Health Project survey, Turkey 2008 DHS survey, Turkmenistan 2006 MICS, Zambia 2007 Demographic Sample Survey, and Zimbabwe 2009 Multiple Indicator Monitoring Survey). In addition, the IHME did not include data for the most recent year from well-functioning VR systems for many industrialised countries (e.g., Belgium, Canada, Denmark, New Zealand, Sweden, Switzerland, and the US). Differences in completeness are likely to be the drivers for the large differences in estimates of U5MR between the UN IGME and the IHME for countries such as Turkey and Comoros (Figure 4; note that the IHME data series are not available and thus not shown; comparisons were made based on data shown in the graphs of the online appendix of [2]). The IHME's U5MR estimate for Turkey is 31.3 per 1,000 live births for the year 2010, which is much higher than the UN IGME's estimate (which is 17.6 per 1,000 live births for the year 2010) and the observation from the 2008 Turkey DHS survey (24 per 1,000 live births for the year 2006), which was not available in the IHME database. For Comoros, the IHME estimate included data only up to 1996, whereas the UN IGME estimate also included 2003 census data in its curve fitting, resulting in different trend lines after 1996. For example, the estimate of U5MR for the year 2003 is 78.5 per 1,000 live births by the IHME, which is much lower than the UN IGME estimate of 99.2 and the 2003 census finding of 113.

#### Treatment of incomplete VR and calculation of U5MR data

**Figure 5:**
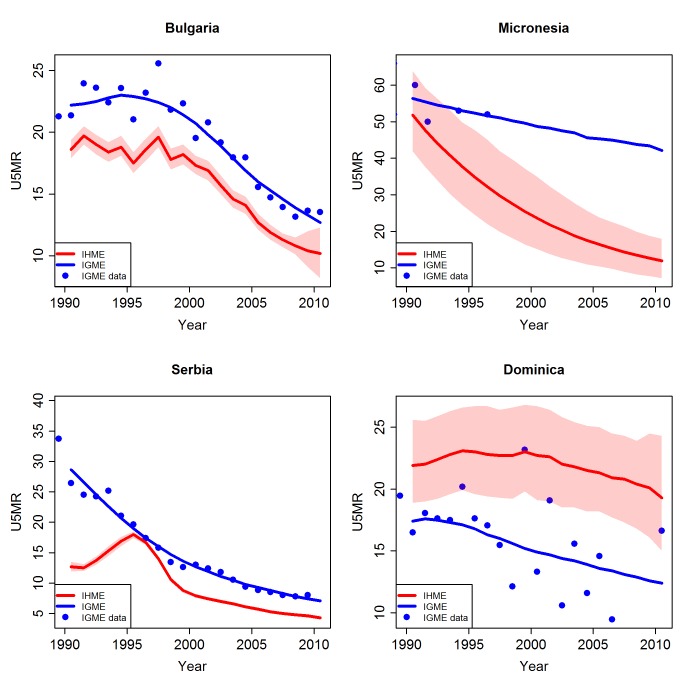
Comparison of U5MR estimates from 1990 to 2010 for examples of countries with different treatment of vital registration data by the UN IGME and the IHME. Estimates by the UN IGME (blue line) and the IHME (red line, with 95% confidence intervals represented by the shaded areas). Data from the UN IGME 2011 database (IGME data) are added as blue dots.

The differences in treatment of incomplete VR caused some differences in estimates. An example is Bulgaria (see Figure 5), where the UN IGME adjusted the VR observations upwards by 20%. The IHME's estimates were lower: either it did not estimate a bias, or the estimated bias was small. Micronesia is an example of a country where the UN IGME excluded the VR data on the basis of incompleteness, while the IHME included the data. Lastly, the UN IGME did not carry out any adjustment of the data in Serbia and Dominica, but the IHME's VR data (as observed in the online appendix of [2]) are very different from the UN IGME's VR data; therefore, the estimates derived from the VR data were also different.

#### Exclusion of data series from surveys or censuses

**Figure 6:**
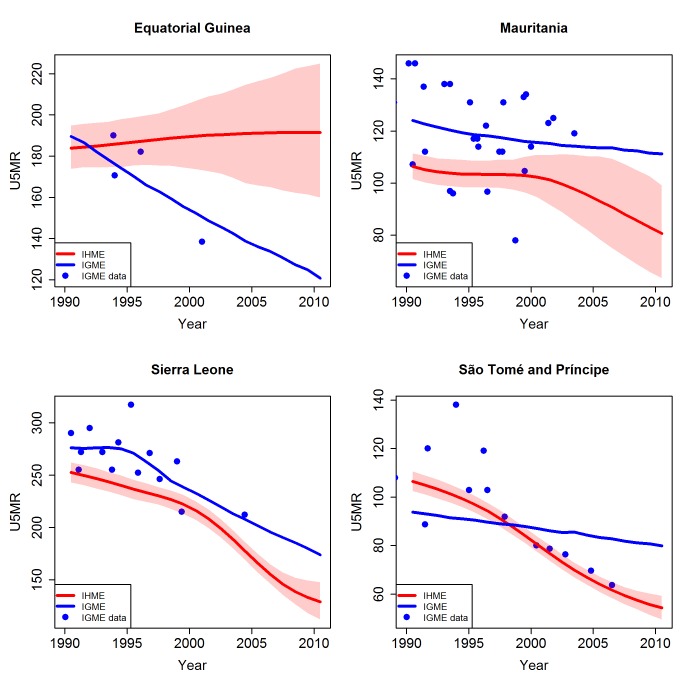
Comparison of U5MR estimates from 1990 to 2010 for examples of countries where different data series were included and excluded by the UN IGME and the IHME. Estimates by the UN IGME (blue line) and the IHME (red line, with 95% confidence intervals represented by the shaded areas). Data from the UN IGME 2011 database (IGME data) are added as blue dots.

The differences in exclusion of data resulted in large differences in estimates for countries such as Equatorial Guinea, Sierra Leone, Sao Tome and Principe, and Mauritania (see Figure 6). For Equatorial Guinea, several inclusion differences led to the difference in U5MR estimates: data from 1983, 1994, and 2001 censuses were included by the UN IGME but not included by the IHME. In addition, for the MICS, carried out in 2000, the UN IGME used the indirect estimates based on reports of women aged 25–29 y and 30–34 y and excluded data from older women (motivated by poor data quality of the data derived from older women in this survey), while the IHME included the whole series, which shows an increasing trend in U5MR. This difference in the underlying data used caused substantial differences in estimates by the two groups. Similarly, differences in estimates of U5MR occurred for Sierra Leone because the DHS survey that was carried out in 2008 was excluded by the UN IGME, but included by the IHME. For Sao Tome and Principe, the UN IGME excluded the 2006 MICS (for which U5MR values were consistently below those of other data sources), whereas this survey was included by the IHME. For Mauritania, the 2007 MICS was excluded by the IHME and included by the UN IGME, explaining the higher estimates by the UN IGME compared to the IHME.

#### Adjustments in high HIV prevalence countries

**Figure 7:**
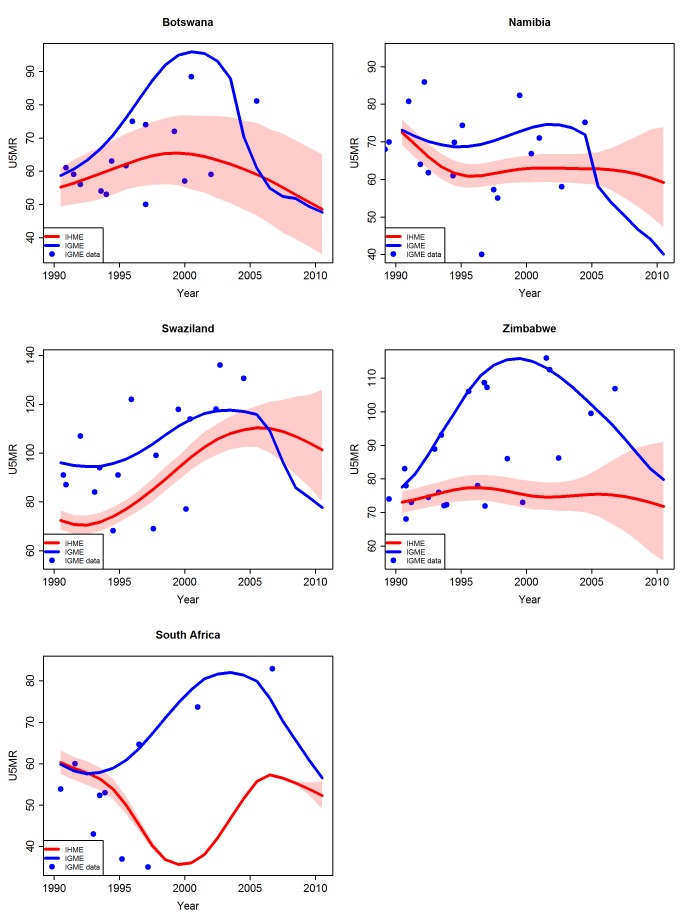
Comparison of U5MR estimates from 1990 to 2010 for examples of high HIV prevalence countries where the UN IGME carried out an adjusted estimation procedure. Estimates by the UN IGME (blue line) and the IHME (red line, with 95% confidence intervals represented by the shaded areas). Data from the UN IGME 2011 database (IGME data) are added as blue dots. Note that for South Africa, treatment of VR data also differs between the UN IGME and the IHME.

The adjustment procedure used by the UN IGME resulted in higher UN IGME estimates than IHME estimates during the peak of the HIV epidemic for high HIV prevalence countries such as Botswana, Lesotho, Namibia, South Africa, Swaziland, and Zimbabwe (Figure 7). An additional reason why the estimates for South Africa are very different between the UN IGME and the IHME is the treatment of VR: the UN IGME excludes the VR, while the IHME includes the VR data, and the IHME estimates follow these data points closely in recent years.

#### Adjustments in countries with conflicts or with limited observations of dubious quality

**Figure 8:**
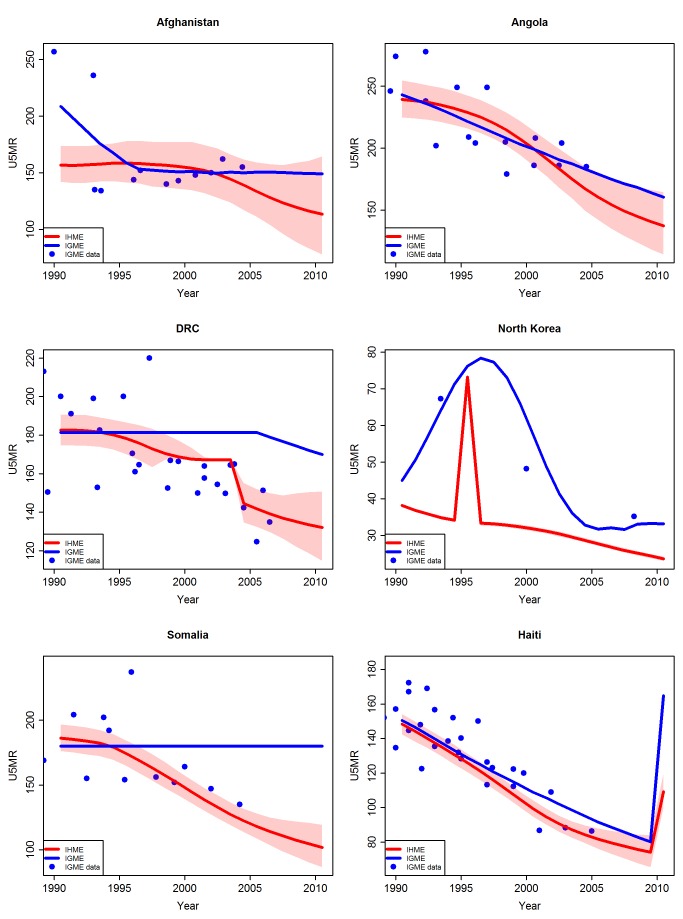
Comparison of U5MR estimates from 1990 to 2010 for examples of countries with conflicts/natural disasters or dubious data quality. Estimates by the UN IGME (blue line) and the IHME (red line, with 95% confidence intervals represented by the shaded areas). Data from the UN IGME 2011 database (IGME data) are added as blue dots.

Differences in estimation method had a large impact in countries with conflicts or with limited observations of dubious quality. Examples of countries where adjustments were carried out by the UN IGME are Somalia, Angola, Afghanistan, DRC, and North Korea (Figure 8). For all of these countries except DRC and North Korea, the IHME did not use an adjusted estimation procedure. For DRC and North Korea, the IHME carried out an adjustment during certain periods only. For Haiti, where an earthquake in 2010 led to a sudden increase in the U5MR, both the UN IGME and the IHME carried out an adjustment to estimate the peak in U5MR in 2010, but the adjustment by the IHME was smaller than UN IGME's adjustment, leading to a difference in estimates.

### Decomposition of Differences in U5MR Estimates

**Figure 9:**
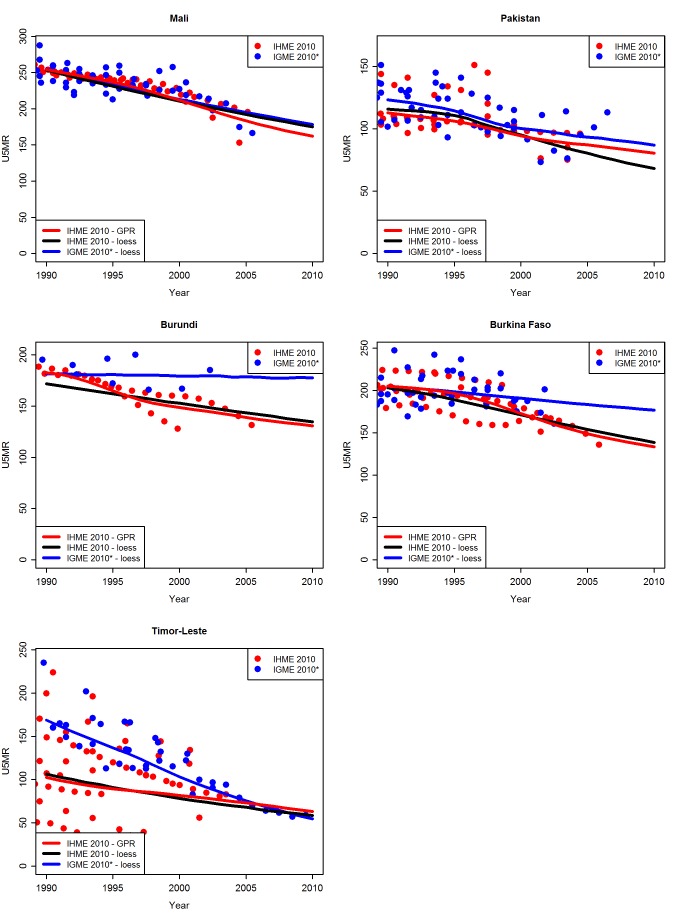
Examples of U5MR estimates based on loess versus GPR fitting methods. For each country is shown (i) loess fit to the 2011 UN IGME database (IGME 2010*; data and fit in blue; dataset excludes data collected in 2010), (ii) loess fit to the 2010 IHME database (data in red; fit in black), and (iii) GPR fit to the 2010 IHME database (IHME 2010; data and fit in red).

The results of the decomposition of differences in U5MR estimates for all 36 countries are shown in Figure S2. Overall, the differences that arise from fitting the loess smoother versus the 2010 version of the GPR to the IHME data are small compared to the differences between fitting the loess smoother to the IHME data versus the UN IGME data. Exceptions are countries such as Mali and Pakistan, where the most recent observation is several years before 2010, and the extrapolations of loess and GPR differ (Figure 9). For countries such as Burundi and Burkina Faso (Figure 9), the differences for 2010 are caused by a difference in the underlying data that are used: even though the most recent observation is several years before 2010, the extrapolations based on the loess or GPR are similar for the IHME data. For Timor-Leste in 1990, the difference in the underlying data used results in a large difference in U5MR estimates, while the difference between GPR and loess fits to the IHME data is very small.

**Figure 10:**
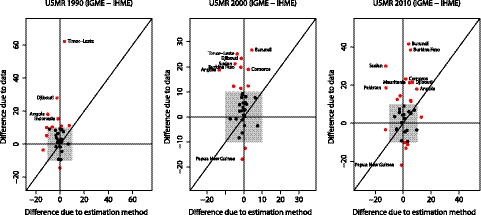
Decomposition of differences in U5MR for 1990, 2000, and 2010 into differences due to data and differences due to use of GPR. The grey box represents differences up to ten deaths per 1,000 births. Countries for which the difference due to either factor is larger than ten deaths per 1,000 births are highlighted in red.

The decomposition of the difference in the U5MR estimates for a country in a particular year into differences that are due to different use of data and differences that are due to using the GPR 2010 estimation method instead of the loess smoother is summarised in Figure 10 for 1990, 2000, and 2010, where the differences due to the data (Δ*R* ^(d)^) are plotted against the differences due to use of the GPR method (Δ*R* ^(m)^). The figure illustrates that in the decomposition exercise in 2010, for Pakistan, the UN IGME estimate is lower by 20 deaths per 1,000 live births than the IHME's estimate because of the estimation method. For Burundi and Burkina Faso, the UN IGME estimate is higher by around 40 deaths per 1,000 births because of the data used. For Timor-Leste in 1990, the difference in the datasets used gives a difference of around 60 deaths per 1,000 live births, while the difference due to the estimation method used is close to zero. Table 3 summarises the mean differences (with standard deviations of the differences). Generally, differences due to different data are positive (UN IGME estimate >IHME estimate; the average difference is around 6 deaths per 1,000 births in all years), and larger than differences due to the estimation method used (which are close to zero).

**Table 3:** Summary of decomposition results: mean differences in U5MR in 1990, 2000, and 2010.

Difference in U5MR	Year		
	1990	2000	2010
Due to data	6.0 (12.4)	6.1 (10.2)	6.2 (14.1)
Due to use of GPR (IHME 2010) as compared to loess	−1.6 (4.7)	−0.8 (3.6)	0.8 (5.9)

Results are given as the mean difference in deaths per 1,000 live births (standard deviation).

### Decomposition of Differences in the Estimated Number of Under-Five Deaths

**Figure 11:**
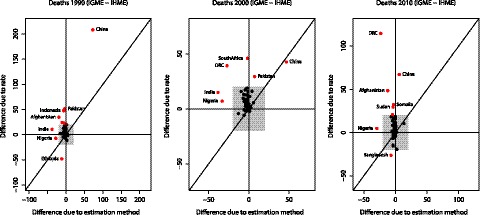
Decomposition of differences in under-five deaths for 1990, 2000, and 2010 into differences due to rates and differences due to estimation method. The grey box represents differences up to 20,000 deaths. Countries for which the difference due to either factor is larger than 20,000 deaths are highlighted in red.

The decomposition of differences in the estimated number of under-five deaths for all 186 countries for 1990, 2000, and 2010 is summarised in Figure 11, where differences due to U5MR differences are plotted against differences due to the estimation method used. For the estimated number of under-five deaths in 1990, the largest absolute difference due to different rates occurs in China—a difference of about 209,000 deaths is caused by the different U5MRs used by the two groups to calculate the number of under-five deaths. Due to China's population size, a small difference in rates causes a large difference in the estimated number of deaths. The estimated number of deaths in Pakistan, Ethiopia, Indonesia, and Afghanistan are also affected substantially by using different rates. The largest impact of the use of different methods on the estimated number of under-five deaths in 1990 occurs in China, India, Niger, Afghanistan, and DRC. For China in 1990, a difference of 73,000 deaths is caused by using different estimation methods. Similarly, for the estimated number of under-five deaths in 2000 and 2010, the largest absolute differences due to the use of different rates between the two groups occur in countries with a large population, or countries where the rates estimated by the two groups differ substantially, such as Afghanistan. In general, differences due to rates are positive (UN IGME estimate >IHME estimate) and larger than differences due to the estimation method used (see Table 4).

**Table 4:** Summary of decomposition results: mean differences in under-five deaths in 1990, 2000, and 2010.

Difference in Under-Five Deaths	Year		
	1990	2000	2010
Due to rate	3.0 (17.2)	2.3 (6.8)	2.4 (12.0)
Due to alternative IHME estimation method	−0.9 (6.9)	−0.9 (5.8)	−0.7 (3.4)

Results are given as the mean difference in the number of deaths in thousands (standard deviation).

## Discussion

In this article, we compared the UN IGME and IHME estimates of U5MR, ARR, and the number of under-five deaths. We found that the estimates of U5MR from the UN IGME and the IHME are similar at the global level: both UN IGME and IHME estimates confirm that substantial progress has been made in reducing child mortality in the last two decades. However, differences between the UN IGME and IHME estimates of U5MR exist at the country level. The difference in estimated U5MR was more than 10% and corresponded to more than ten deaths per 1,000 live births for 10% of all countries in 1990, 15% of all countries in 2000, and 20% of all countries in 2010. The largest differences in the estimates of U5MR are found in countries with conflicts or civil unrest (e.g., Afghanistan, Angola, and Somalia), countries with high HIV prevalence (e.g., Botswana, Namibia, and Zimbabwe), and countries where the underlying data used to derive the estimates were different, especially if the exclusion of data series differed between the two research groups, as described for Equatorial Guinea and Sierra Leone. We also found that the differences in estimates due to data are on average larger than the differences due to trend fitting method.

The finding that differences in estimates due to data are on average larger than the differences due to curve fitting method suggests that differences in estimates can most often be understood by examining the differences in the underlying data used instead of the trend fitting model used. Future discussion should therefore be more focused on strengthening the underlying data. Nonetheless, the choice of the trend fitting model is important for producing accurate estimates and for communicating to users the construction of the U5MR estimates. The IHME has chosen its trend fitting method (GPR) based on predictive validity in out-of-sample exercises and has shown previously that GPR has better out-of-sample performance than the simpler loess method that is used by the UN IGME [8]. Given the lack of recent U5MR data for many countries, the out-of-sample accuracy is an advantage of the GPR approach over the loess approach, but differences in U5MR estimates caused by different approaches are limited according to our decomposition results. The disadvantage of the GPR approach is the complexity of the fitting procedure; currently, most users of the U5MR estimates are not able to reproduce the GPR estimates and might have limited understanding of how the procedure works because its explanation in the recent publication [2] is very limited. This is not an issue for the UN IGME estimation procedure; an explanation of the methods that were used, the data, and the necessary software are available at http://www.childmortality.org. We feel that, ultimately, countries should be able to monitor their progress towards MDG 4 and have full access to software, data, and an explanation of how to carry out the trend fitting procedure.

### Countries with Conflicts, Civil Unrest, or Natural Disasters

The observed differences between the UN IGME and IHME estimates for countries with conflicts, civil unrest, or major natural disasters confirm that estimating child mortality is especially challenging in such countries, where mortality rates and needs for data are high, but strong scientific evidence is hard to develop because of a lack of systematic and sound epidemiologic data. Special attention should be paid to the development of complex emergency surveillance systems that can give more accurate information on child mortality during conflict periods [12]. Such information could reduce differences in estimates. In the current setting, the UN IGME carries out expert-based adjustments for countries with conflicts or with limited observations of dubious quality, based on other available information, such as observed changes in health intervention coverage or trends in neighbouring countries, which can potentially lead to big differences between the default (objective) loess data-driven result and the expert-based (more subjective) published estimates. While recognising the need for objectivity of the estimates where possible, we feel that in situations where data are insufficient for an objective approach, such an approach is preferred to the objective data-driven approach that the IHME uses. Reproducible methods to estimate U5MR while incorporating external information should be explored to improve the validity as well as the objectivity of estimates in countries with conflicts, civil unrest, or major natural disasters.

Some might argue that instead of trying to estimate U5MR in periods with major natural disasters, such as during and shortly after the earthquake in Haiti, a “disaster-removed” U5MR should be provided to focus on the underlying rate of change. Although we agree that in some instances such estimates could be useful to examine a country's accomplishments in reducing U5MR without their being overshadowed by events that are beyond the country's control, we would argue that the default set of U5MR estimates should provide an accurate picture of mortality rates in the country. Moreover, major natural disasters could very well disturb public health infrastructure for a period extending far beyond the period in which the disaster occurred, which would make a “disaster-removed” trend a challenging phenomenon to estimate.

### High HIV Prevalence Countries

The differences in estimates for high HIV prevalence countries between the UN IGME and the IHME are the result of different assumptions used by the two groups. The IHME did not make any adjustment for potential biases in U5MR observations in countries with generalised HIV epidemics. In 2010, after analysing DHS data and carrying out a simulation exercise, IHME researchers concluded: “Overall, it is our continuing judgment that while there is a bias downwards, the uncertainty in each of the required steps to correct for this bias would not make this effort worthwhile and could lead to dramatically misleading conclusions. It is also against our principle that measurement of mortality outcomes should be based on observed data rather than models imposed on the data. We believe this is an important area for future research and if the accumulating body of evidence on the impact of HIV allows for more accurate survivor bias correction then we believe it would be appropriate to implement it” ([8], p. 16 of the online appendix). The IHME comments in their discussion of their 2011 estimates that the effect of high levels of HIV-related mortality on estimates of under-five mortality remains unclear, and advises readers to interpret levels and trends in under-five mortality in countries with large HIV epidemics with caution [2]. The UN IGME did carry out an adjustment of U5MR observations in such countries, to account for underreporting biases [10]. The study by Hallett et al. [13] also indicated that an adjustment is appropriate, based on empirical evidence and a modelling exercise. The authors concluded that “under-five mortality statistics based on retrospective fertility histories need to be corrected for bias due to correlation between deaths among mothers and their young children in populations with generalized HIV epidemics” ([13], p. 11).

### Data from Vital Registration Systems

Complete VR systems are the preferred source of data on child mortality because they collect information as events occur and they cover the entire population. However, many developing countries lack fully functioning VR systems that accurately record all births and deaths. Enhanced efforts to strengthen VR systems are needed. In the meantime, as data from complete VR systems are not available and household surveys or censuses are the primary sources of data on child mortality in many developing countries, the assessment of data quality and development of clear criteria for excluding data sources are important areas for future research. Reliable data are crucial to generate reliable estimates of under-five mortality, regardless which model is used. More research is needed to evaluate and quantify the quality of retrospective data from censuses and surveys, e.g., to estimate errors in data series constructed from full or summary birth histories because of recall biases or reporting biases [14].

The adjustment methods used by the UN IGME and the IHME for data from incomplete VR, e.g., for Bulgaria, leave room for improvement. The advantage of the adjustment carried out by the UN IGME is that it was based on an analysis of the VR systems in the countries, which suggested that there are issues with the reporting of early infant deaths. Its disadvantage is that the uncertainty that is associated with the underreporting estimate is not taken into account. The adjustment of the IHME is based on average differences between the VR estimates and available survey and census data. The advantage of this approach is that it produces a country-specific unbiased estimate of the underreporting bias, if the other data sources are available and unbiased (i.e., do not have underreporting issues), that includes an uncertainty assessment. Its disadvantage is that the quality of the adjustment depends on the availability and quality of other data sources. For Bulgaria, we speculate (based on the 2010 dataset) that the IHME did not carry out a bias adjustment because the only alternative data source in the country, indirect estimates from a census in 1965, were below the VR observations, which would have led to the conclusion that the VR data are unbiased. This conflicts with the analyses by the UN IGME, which indicated 20% underreporting of under-five mortality in the VR data. Adoption of a method that incorporates an estimate of VR underreporting and accounts for the uncertainty therein could eliminate the differences.

### Uncertainty in U5MR Estimates

Differences in the point estimates for U5MR between the UN IGME and the IHME might very well be illustrative of the uncertainty in levels and trends of U5MR within a country, and the lack of reliable data. The finding that differences in estimates are largest for more recent years is not surprising, given the limited availability of data for more recent years for many countries. Quantification of uncertainty could assist in explaining differences in estimates: taking into account the uncertainty, most estimates from the UN IGME and the IHME might no longer be significantly different. The IHME presented uncertainty bounds with their estimates, but has not yet validated the accuracy of its bounds, i.e., how often the uncertainty intervals are expected to contain the “true but unknown” U5MR. A comparison of the 2011 IHME estimates with the bounds that they published in 2010 reveals that revised U5MR estimates are outside the previously constructed uncertainty bounds for 10% of all countries in 2010, and 33% of all countries in 1990, which makes the use and interpretation of the previously constructed bounds troublesome. Given that the 2011 estimation method was reported to be similar to the 2010 method, with no detailed explanation of the changes of method to calculate uncertainty bounds [2], we speculate that the uncertainty bounds that the IHME published in 2011 contain the true U5MR levels for fewer than 95% of all countries. The UN IGME did not publish uncertainty bounds with their 2011 U5MR estimates because the intervals that were constructed with the loess estimation procedure had not yet been validated, and were deemed too narrow. As uncertainty assessments of U5MR are crucial for interpretation of trends, the construction of accurate uncertainty bounds should be a main focus of future research on U5MR estimation methods.

### Transparency in Data and Methods

In our analysis of the main drivers of the differences in the UN IGME and IHME estimates, we were unable to further examine the exact causes of discrepancies for each country between the two sets of estimates since the IHME database is not publicly available. Responding to repeated requests from the authors to share the data, IHME researchers and staff pointed out that researchers were occupied producing the Global Burden of Disease estimates and would not have time to make the data available until the academic papers on this topic were submitted. No projected date has been given so far for sharing the data (as of August 8, 2012), ten months after the IHME's paper on child mortality was published [2]. Given the IHME's principle on transparency, as stated on their website, “we will foster transparency and accountability by providing an explicit data audit trail which provides enough detail for results to be replicated by others” [15], we urge the IHME to provide such data trails in a timely matter, preferably at the same time as the publication of estimates.

### Conclusions

Differences in national estimates of child mortality, as presented here for the UN IGME and the IHME, may cause confusion about the true extent of progress on achieving MDG 4 for countries, policy-makers, donors, and researchers and may foster policy inaction if the reasons for the discrepancies are not clear. Improved transparency on methods and the underlying data used, as well as analyses like we discussed here, will help to improve understanding about the drivers of the differences in estimates and guide the users of the estimates in interpreting conflicting findings for levels or trends. Ultimately, discrepancies in estimates can be reduced through a more concerted effort from countries, United Nations agencies, non-governmental organisations, and donor communities to support data collection to ensure reliable data from surveys or censuses and to strengthen VR systems for real-time monitoring.

## Supporting Information

Figure S1 **Comparison of U5MR estimates from 1990 to 2010 for all countries.** Estimates by the UN IGME (blue) and the IHME (red, with 95% confidence intervals represented by the shaded areas). Data from the UN IGME 2011 database are added as blue dots (IGME data).
(PDF)

Figure S2 **U5MR estimates for 36 countries based on loess versus GPR fitting methods.** For each country is shown (i) loess fit to the 2011 UN IGME database (IGME 2010*; data and fit in blue; dataset excludes data collected in 2010), (ii) loess fit to the 2010 IHME database (data in red; fit in black), and (iii) GPR fit to the 2010 IHME database (IHME 2010; data and fit in red).
(PDF)

Table S1 **Overview of UN IGME and IHME estimates and their (relative) differences for the U5MR and the number of under-five deaths for 1990, 2000, and 2010.** Differences in U5MR estimates of more than 10% or more than ten deaths per 1,000 births, as well as relative differences in the estimates of under-five deaths of more than 10% or more than 10,000 deaths, are highlighted.
(PDF)

## Abbreviations

ARR: annual rate of reduction
DHS: Demographic and Health Surveys
DRC: Democratic Republic of the Congo
GPR: Gaussian process regression
UN IGME: United Nations Inter-agency Group for Child Mortality Estimation
IHME: Institute for Health Metrics and Evaluation
MDG 4: Millennium Development Goal 4
MICS: Multiple Indicator Cluster Survey
U5MR: under-five mortality rate
VR: vital registration
