# Supplementary material for: Child Mortality Estimation: A Comparison of UN IGME and IHME Estimates of Levels and Trends in Under-Five Mortality Rates and Deaths
Source: PLoS Med. 2012 Aug 28;9(8):e1001288. doi: 10.1371/journal.pmed.1001288 (PMC3429386; doi:10.1371/journal.pmed.1001288)
Supplement: Figure S1 — Comparison of U5MR estimates from 1990 to 2010 for all countries. Estimates by the UN IGME (blue) and the IHME (red, with 95% confidence intervals represented by the shaded areas). Data from the UN IGME 2011 database are added as blue dots (IGME data). (PDF) [file pmed.1001288.s001.pdf]

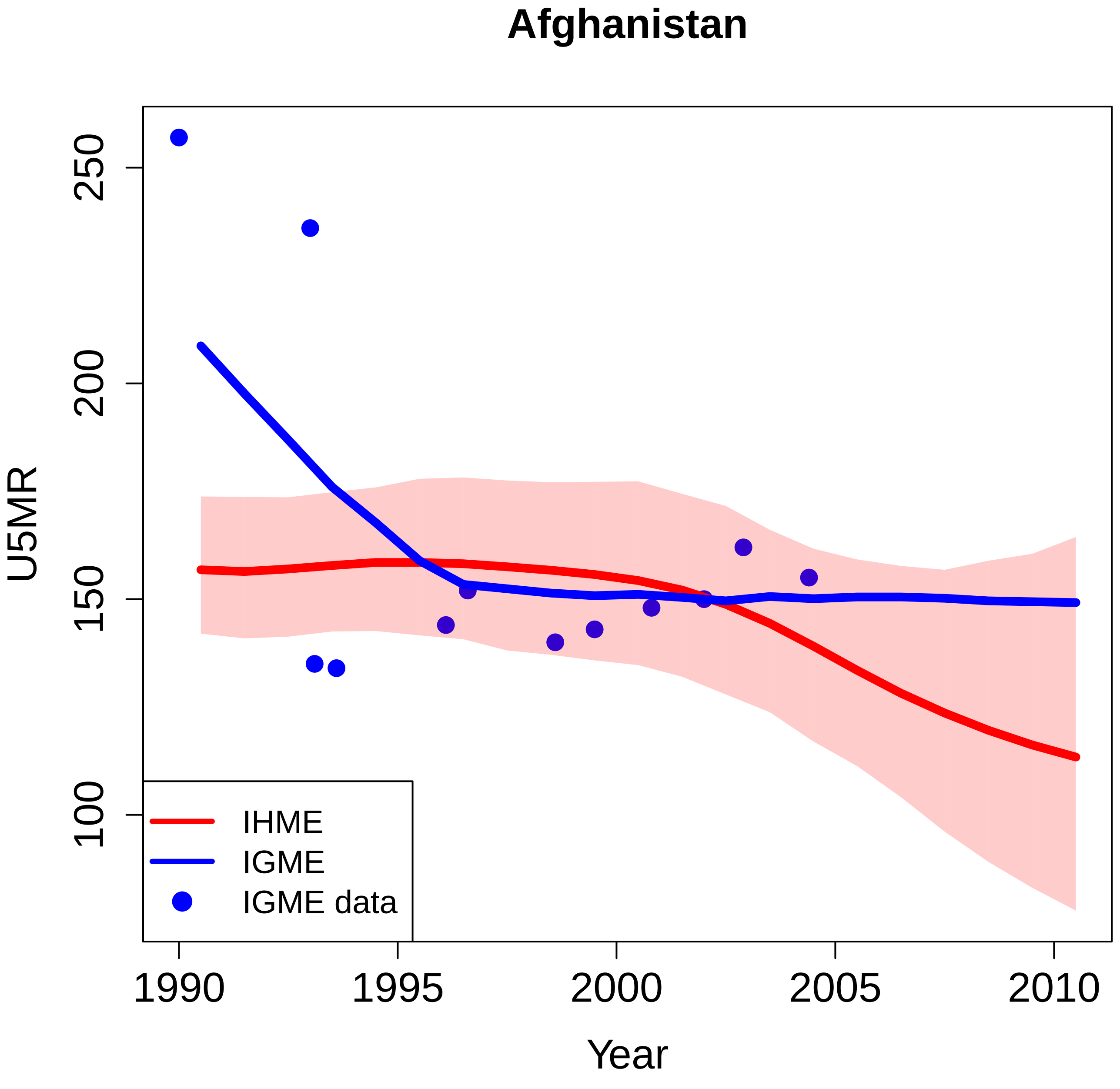

Afghanistan
U5MR
250
200
150
100
1990
1995
2000
2005
2010
Year
IHME
IGME
IGME data

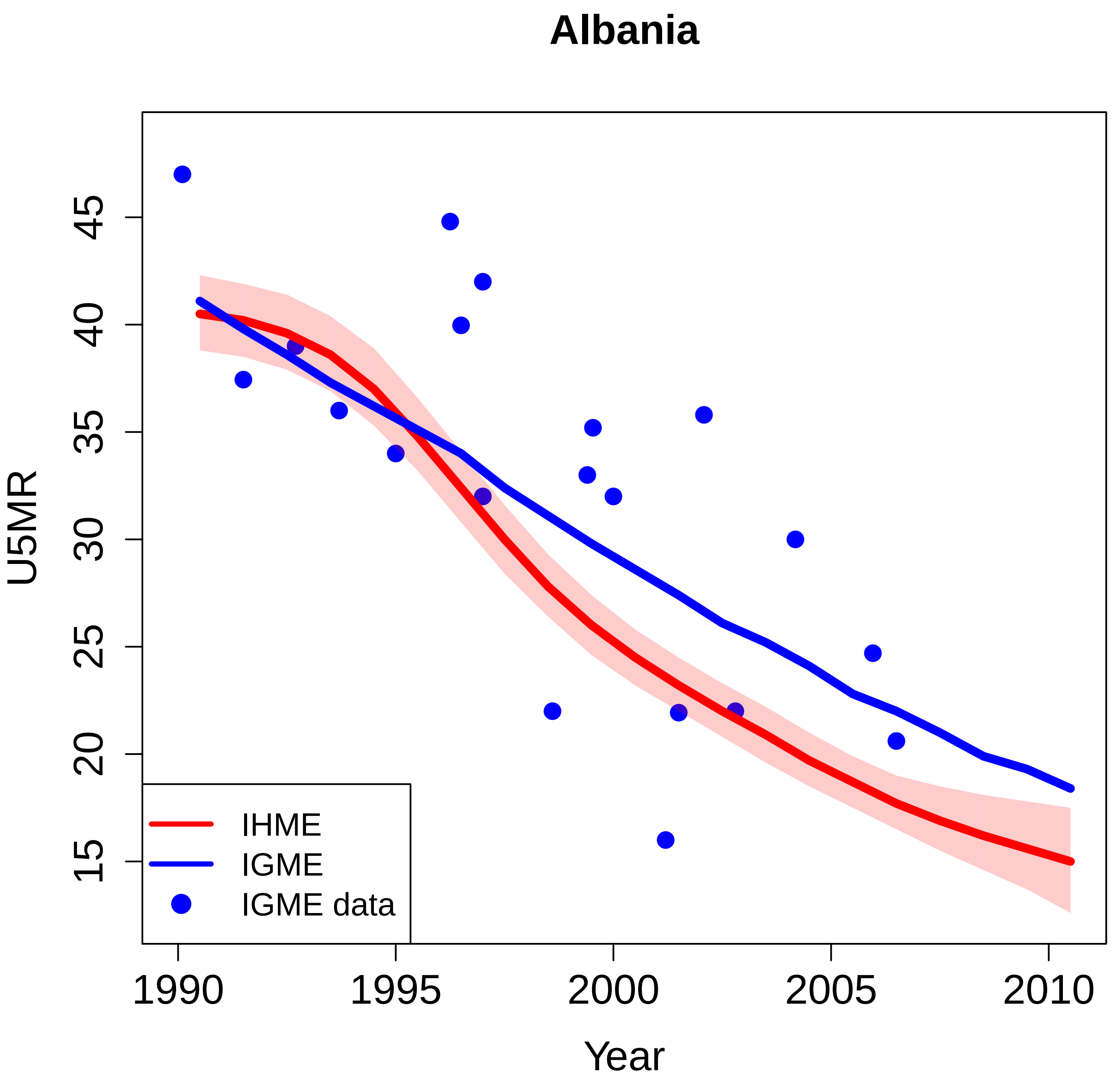

Albania
U5MR
45
40
35
30
25
20
15
1990
1995
2000
2005
2010
Year
IHME
IGME
IGME data

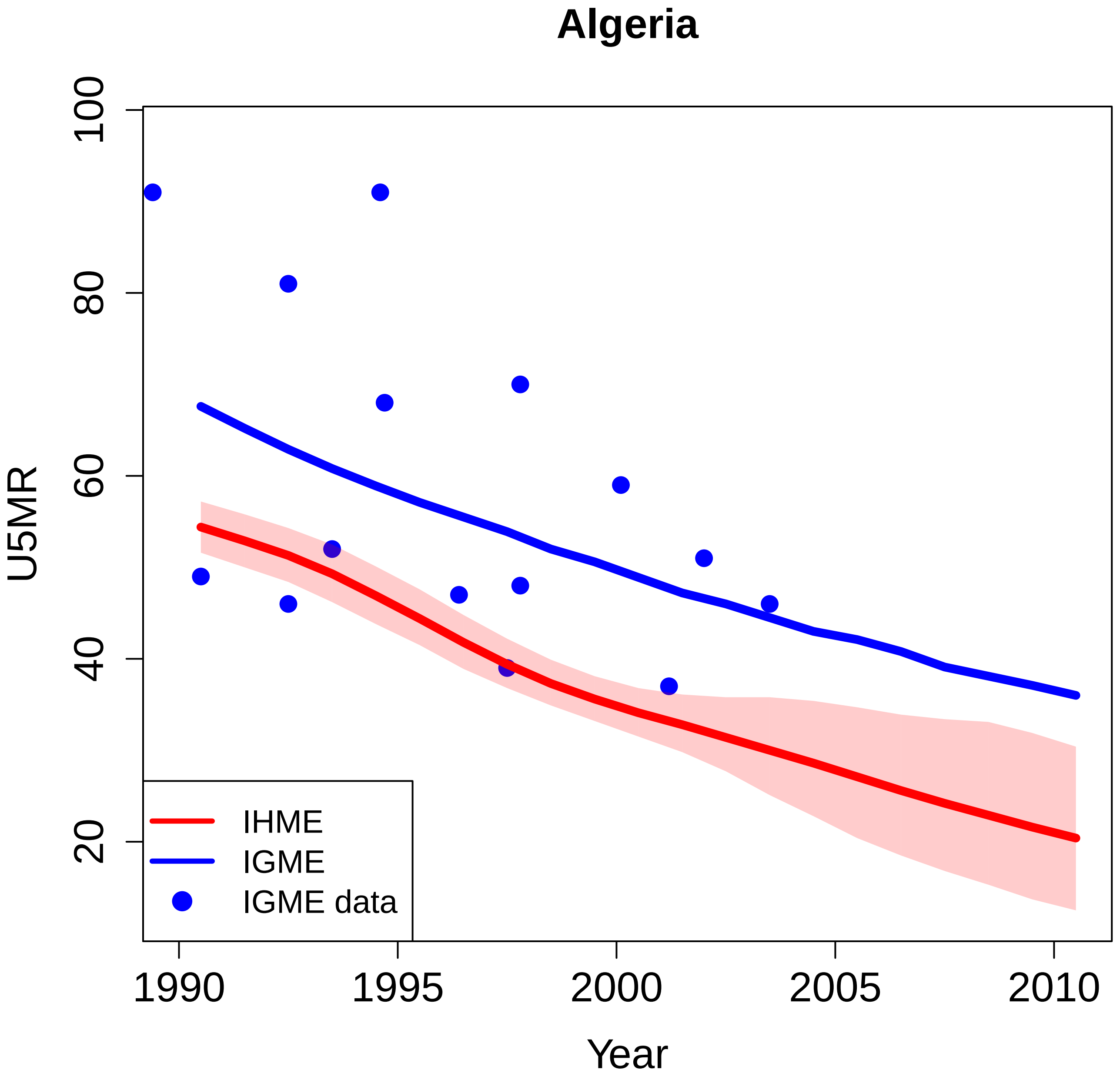
Algeria
100
80
60
40
20
U5MR
1990
1995
2000
2005
2010
Year
IHME
IGME
IGME data

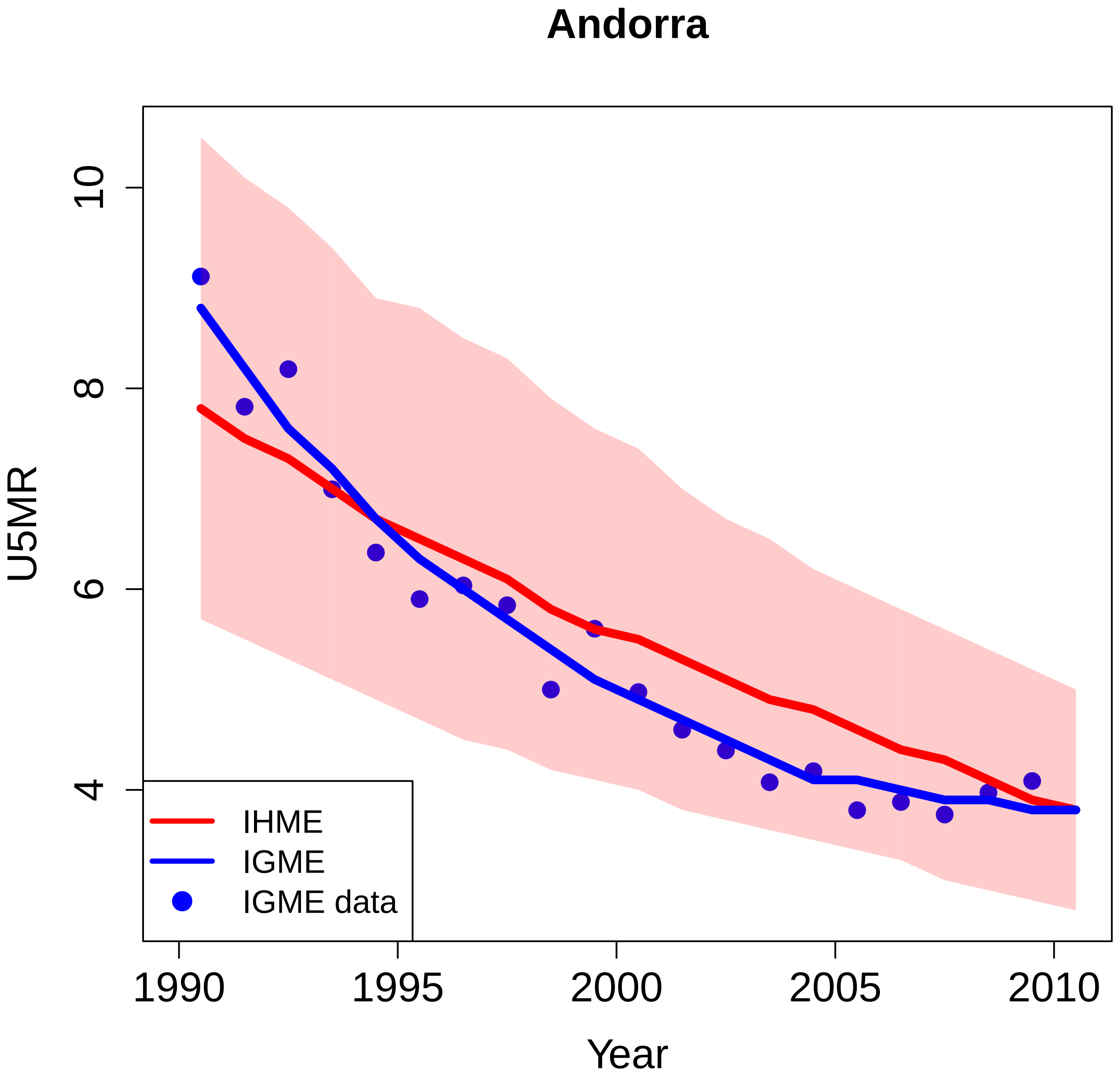
Andorra
U5MR
10
8
6
4
1990
1995
2000
2005
2010
Year
IHME
IGME
IGME data

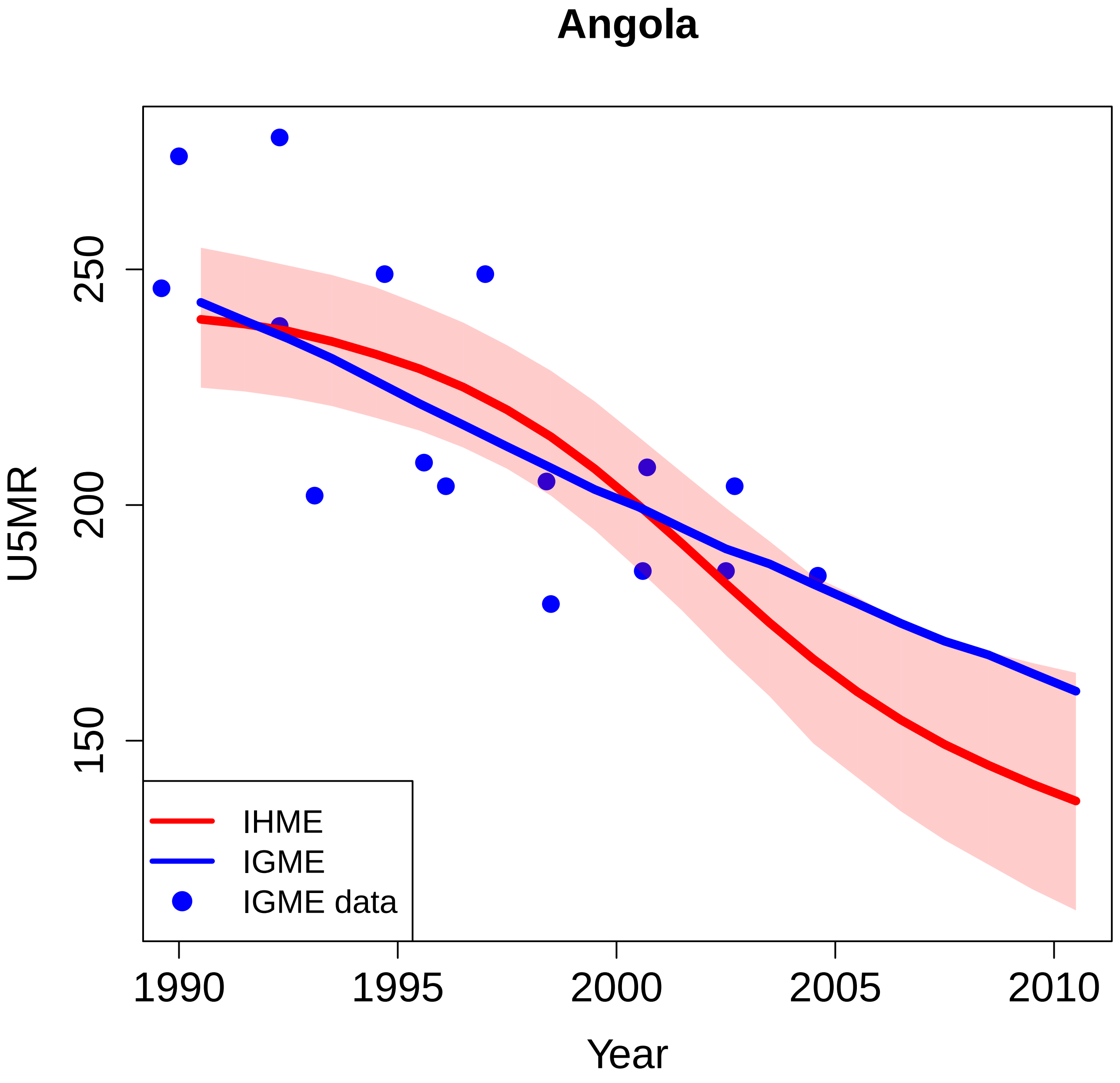
Angola
U5MR
250
200
150
1990
1995
2000
2005
2010
Year
IHME
IGME
IGME data

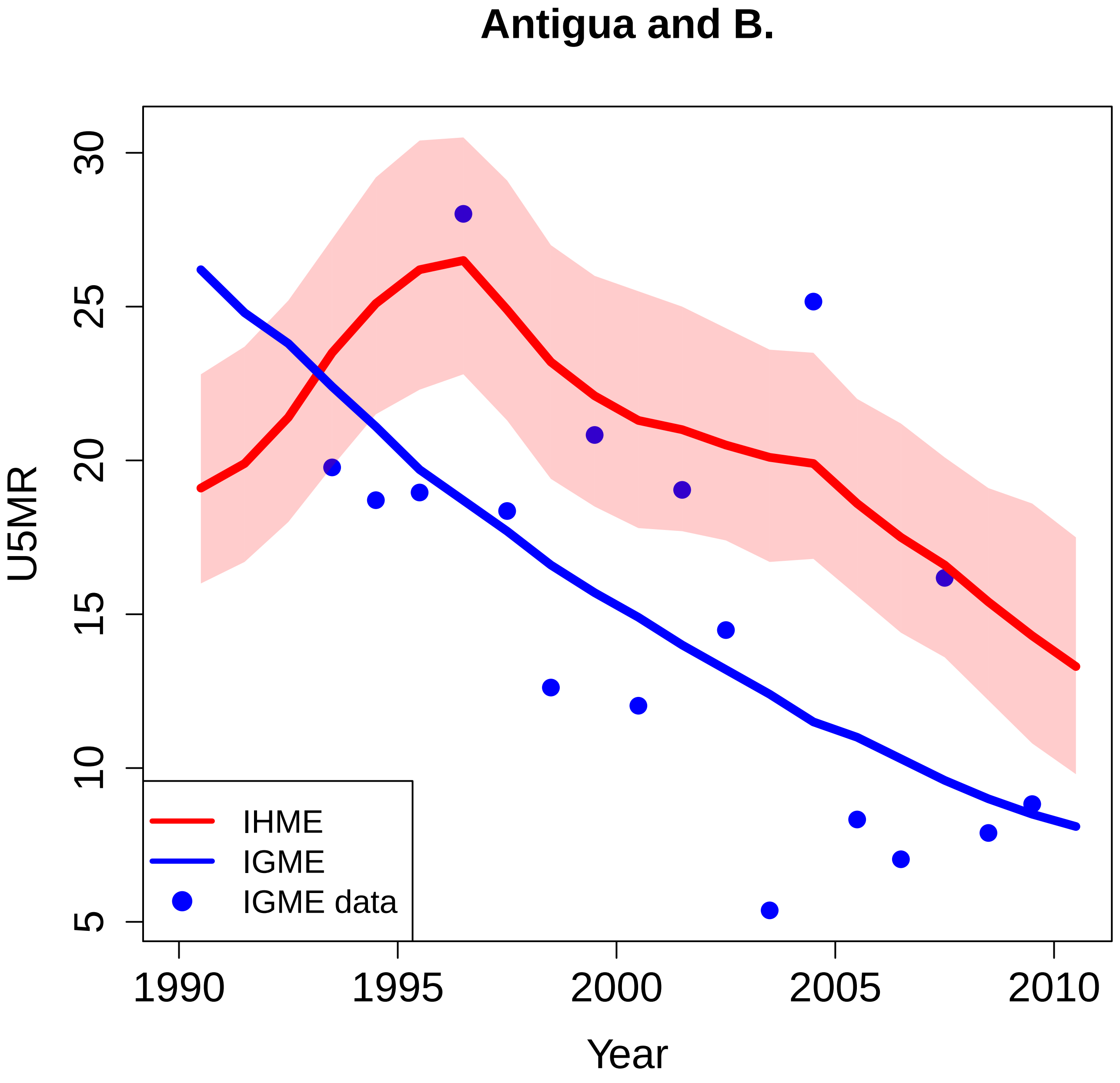
Antigua and B.
U5MR
Year
30
25
20
15
10
5
1990
1995
2000
2005
2010
IHME
IGME
IGME data

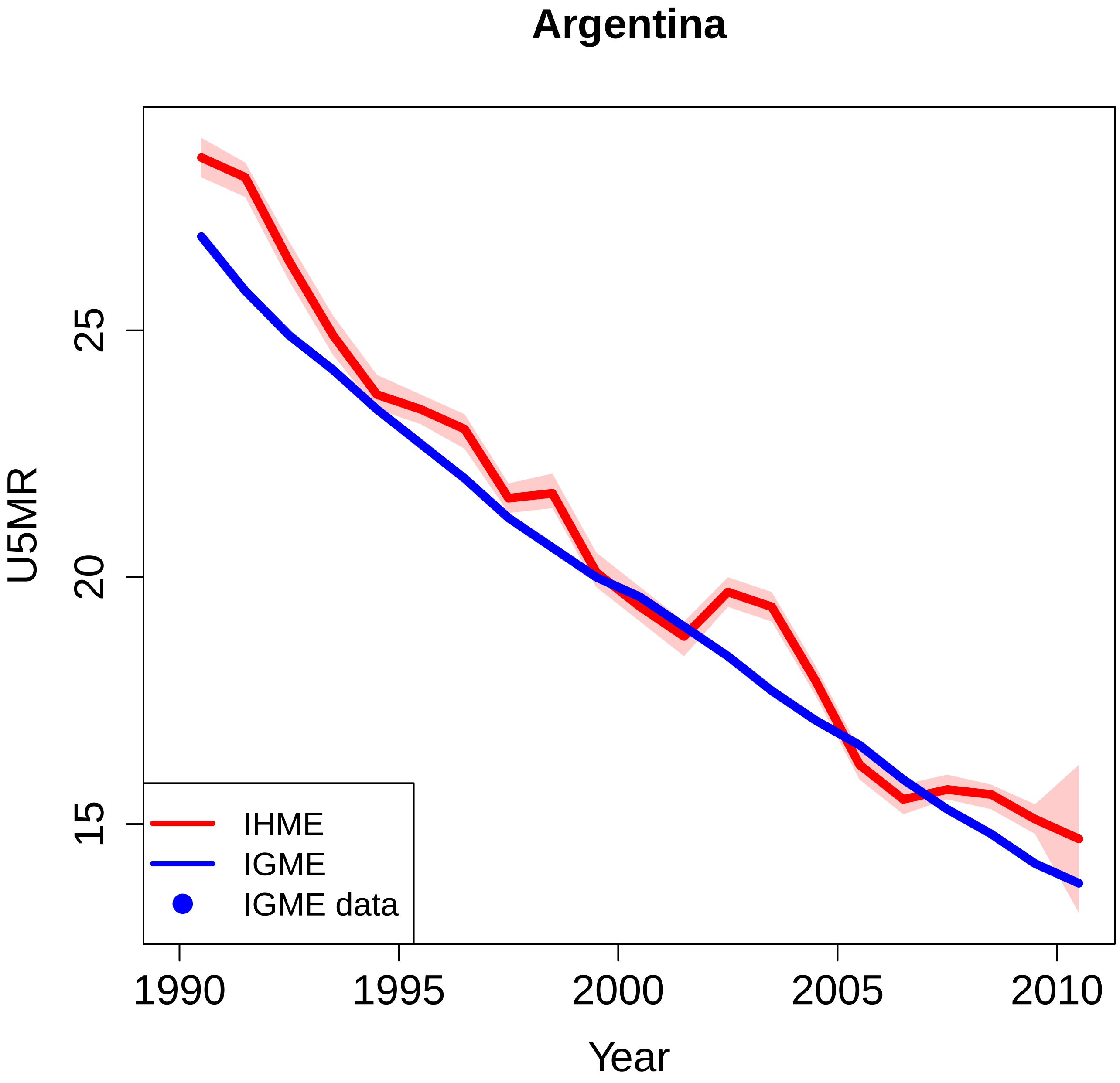

Argentina
U5MR
25
20
15
1990
1995
2000
2005
2010
Year
IHME
IGME
IGME data

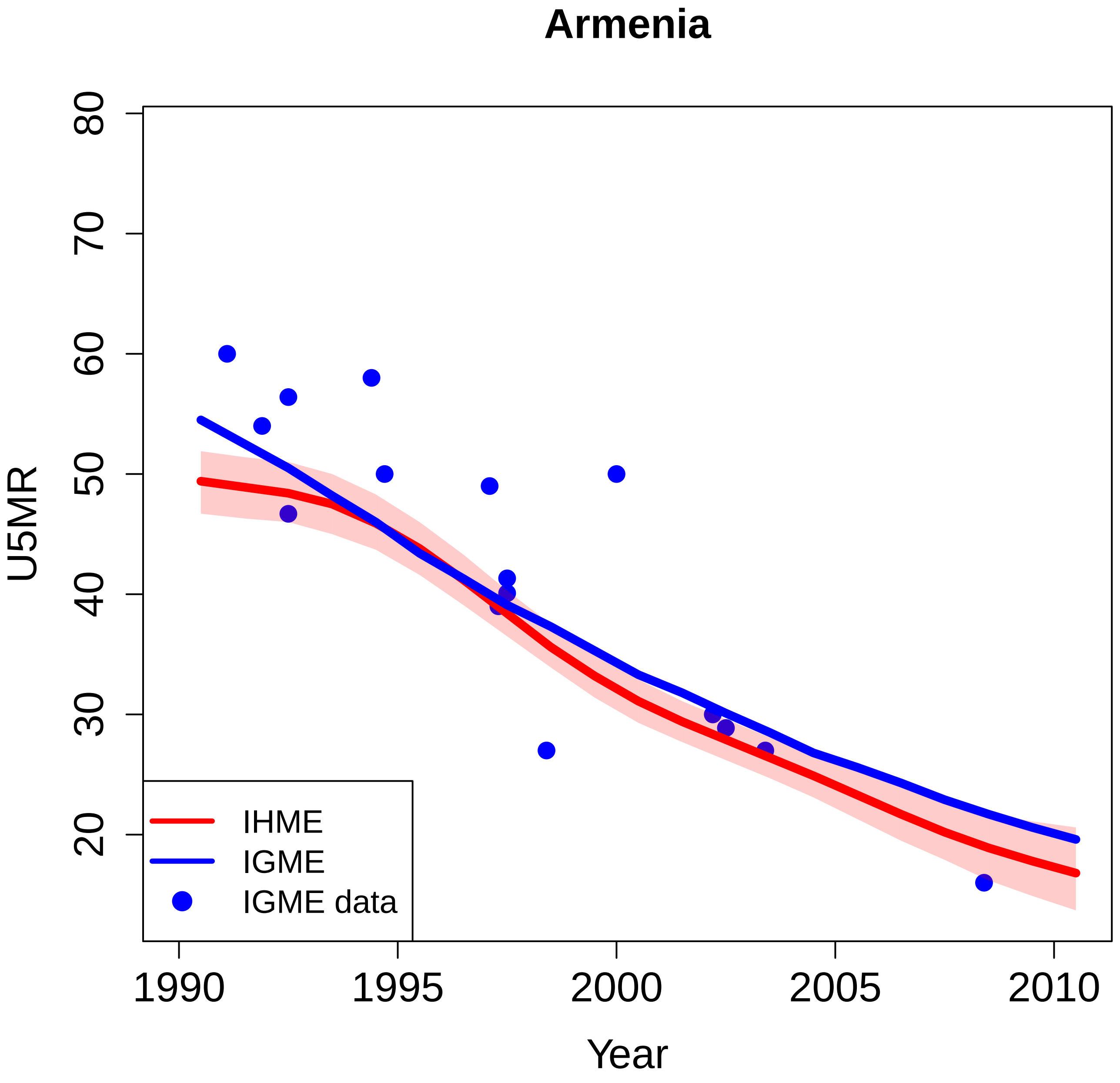

Armenia
U5MR
80
70
60
50
40
30
20
1990
1995
2000
2005
2010
Year
IHME
IGME
IGME data

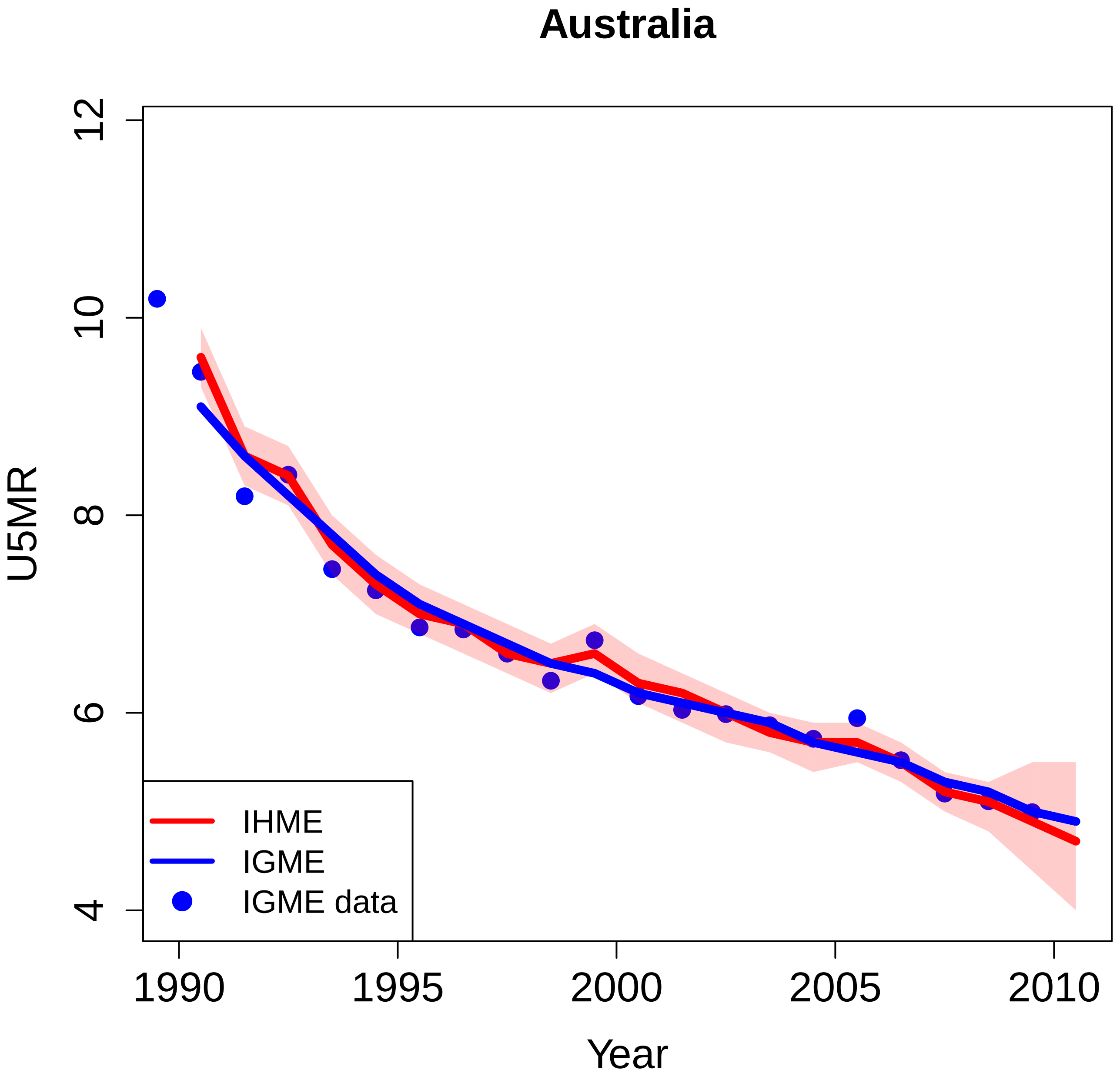

Australia
U5MR
12
10
8
6
4
1990
1995
2000
2005
2010
Year
IHME
IGME
IGME data

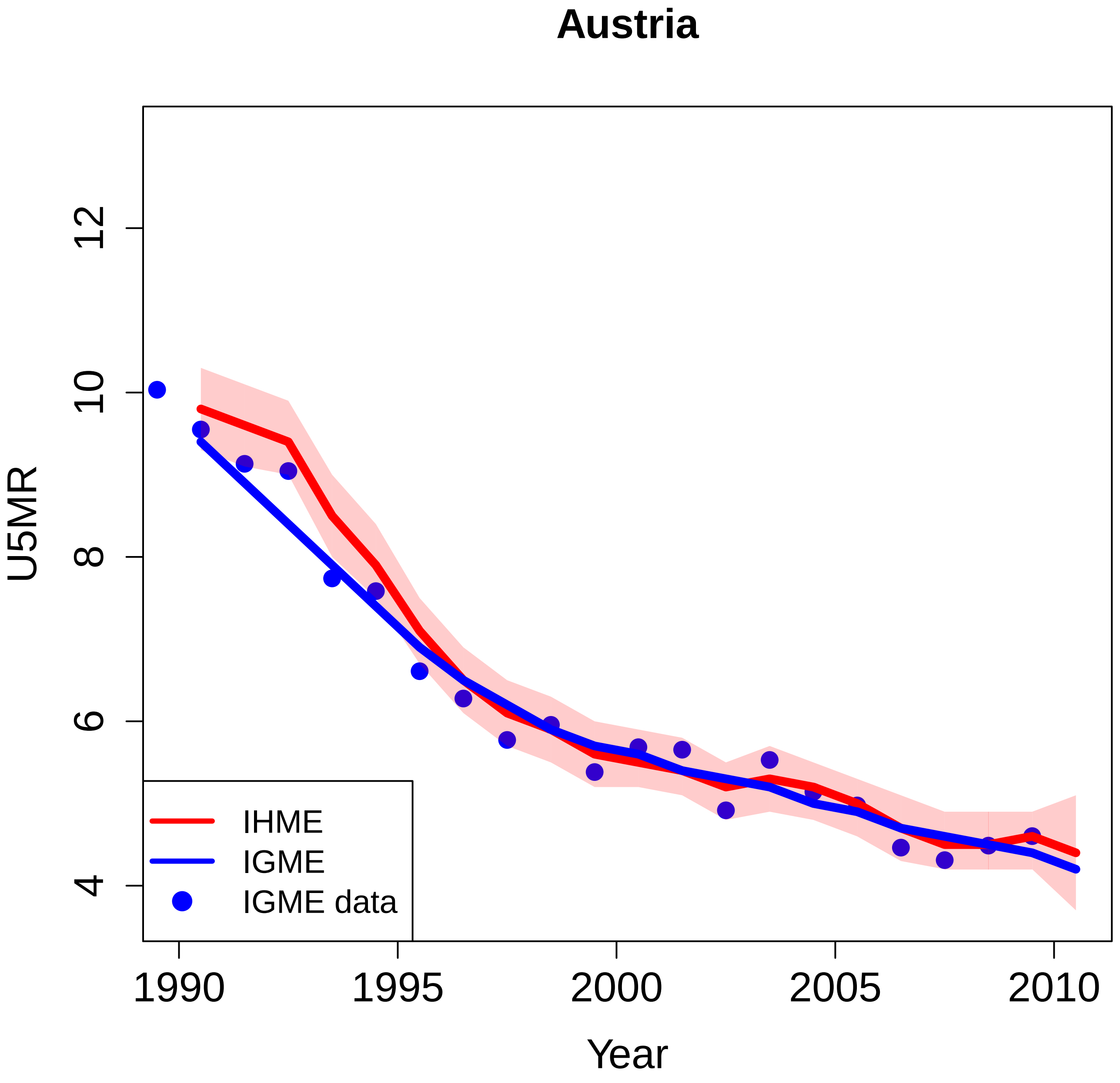
Austria
U5MR
12
10
8
6
4
1990
1995
2000
2005
2010
Year
IHME
IGME
IGME data

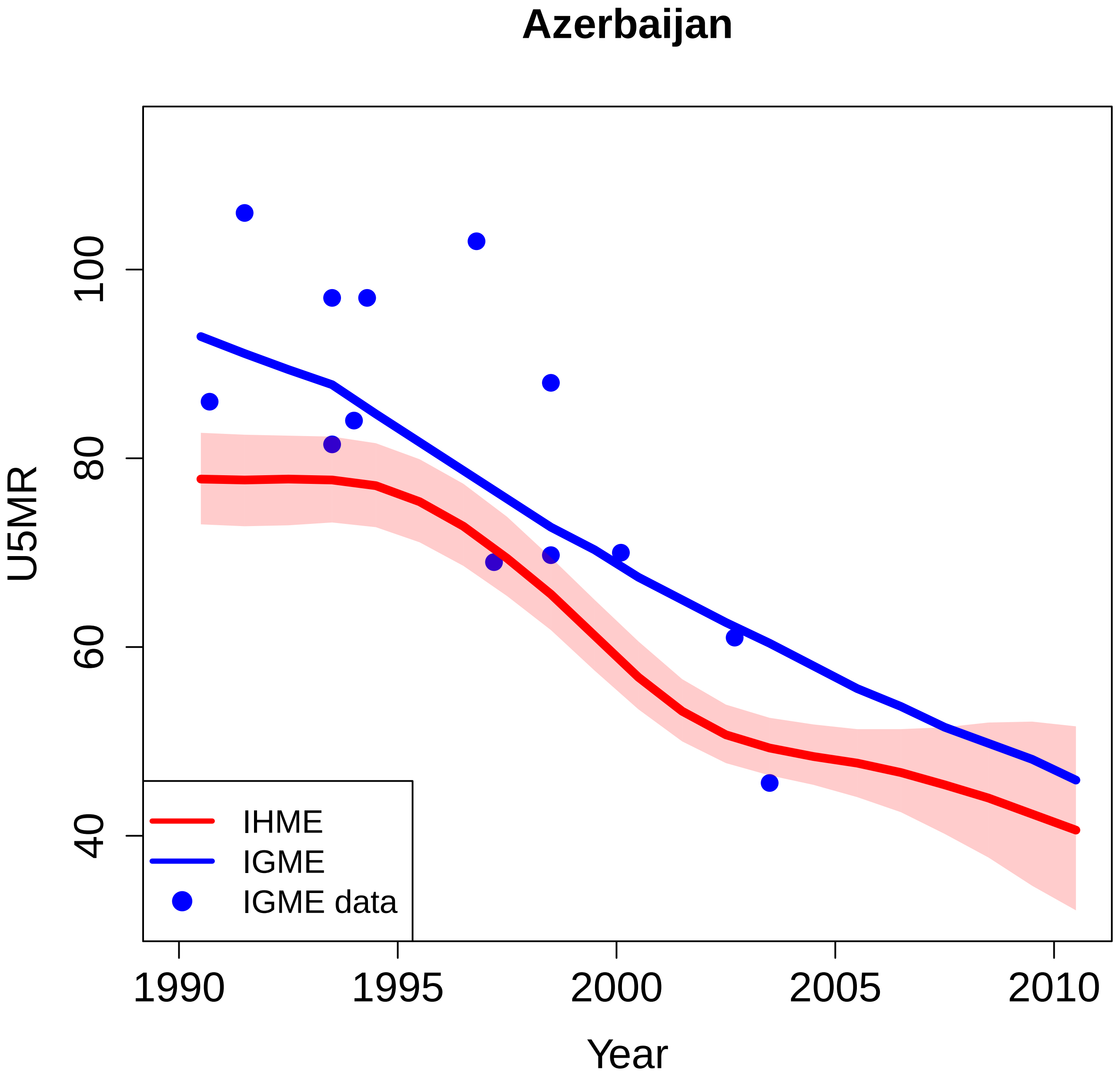

Azerbaijan
U5MR
100
80
60
40
1990
1995
2000
2005
2010
Year
IHME
IGME
IGME data

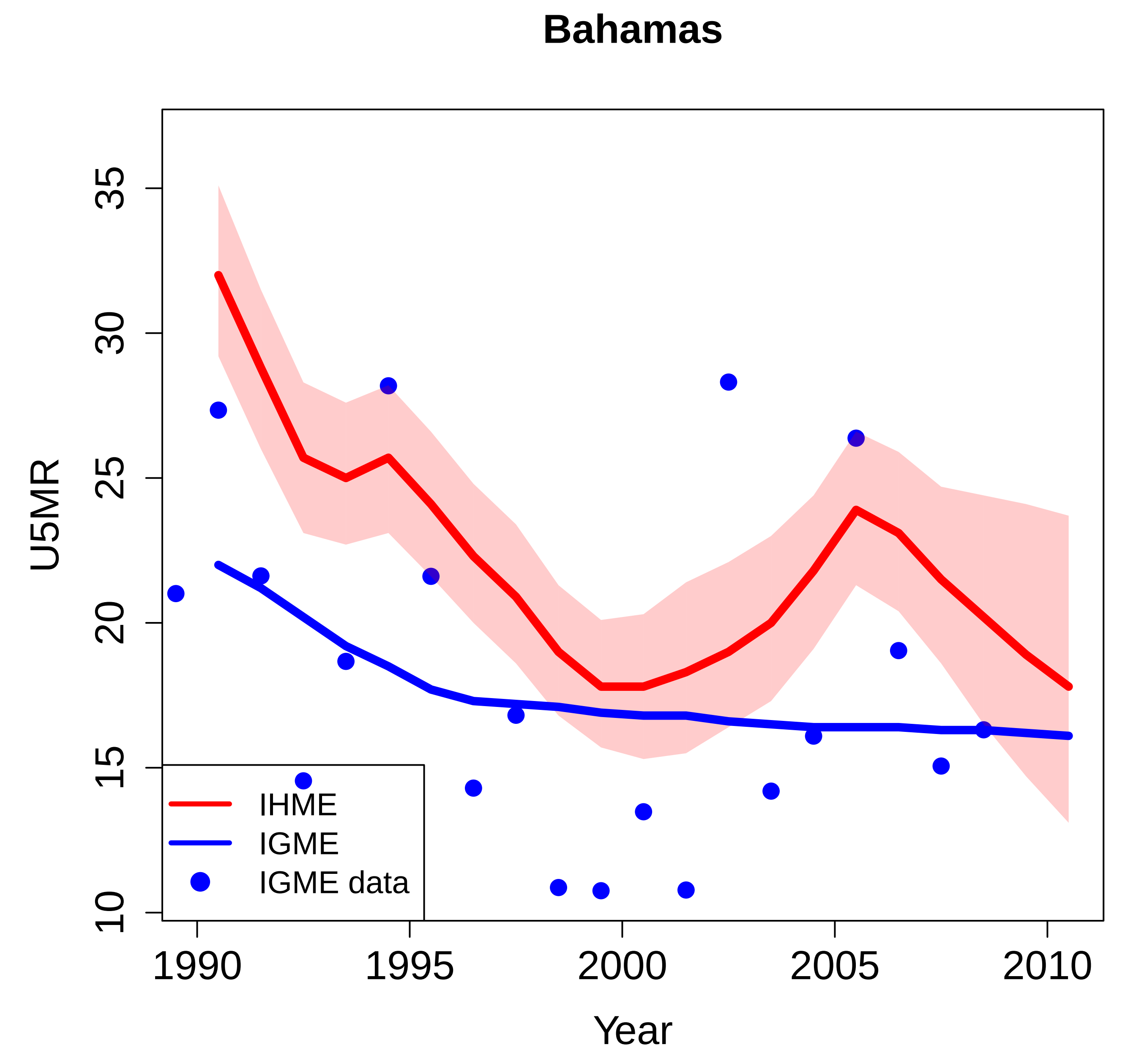

Bahamas
U5MR
Year
35
30
25
20
15
10
1990
1995
2000
2005
2010
IHME
IGME
IGME data

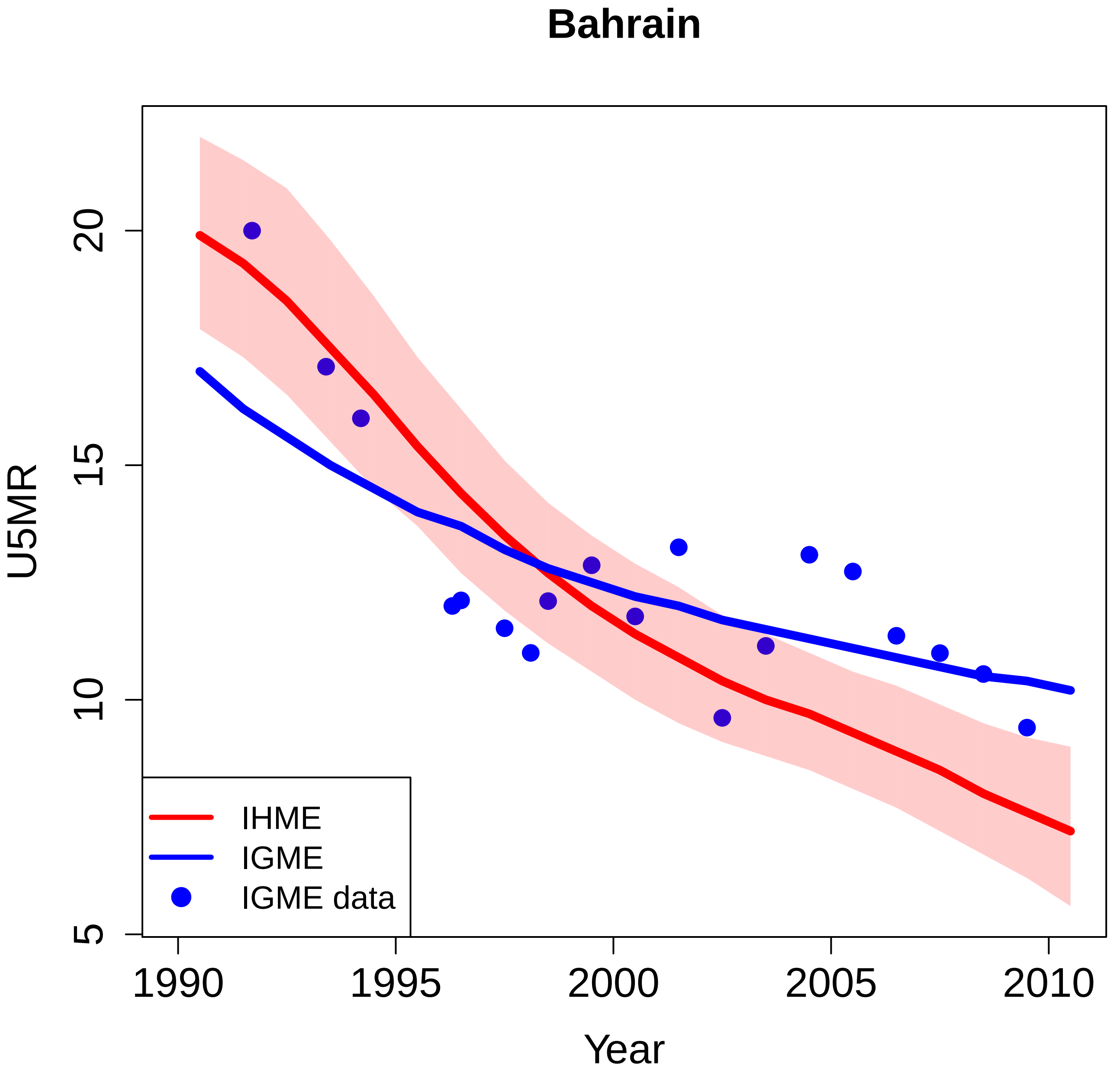

Bahrain
U5MR
Year
20
15
10
5
1990
1995
2000
2005
2010
IHME
IGME
IGME data

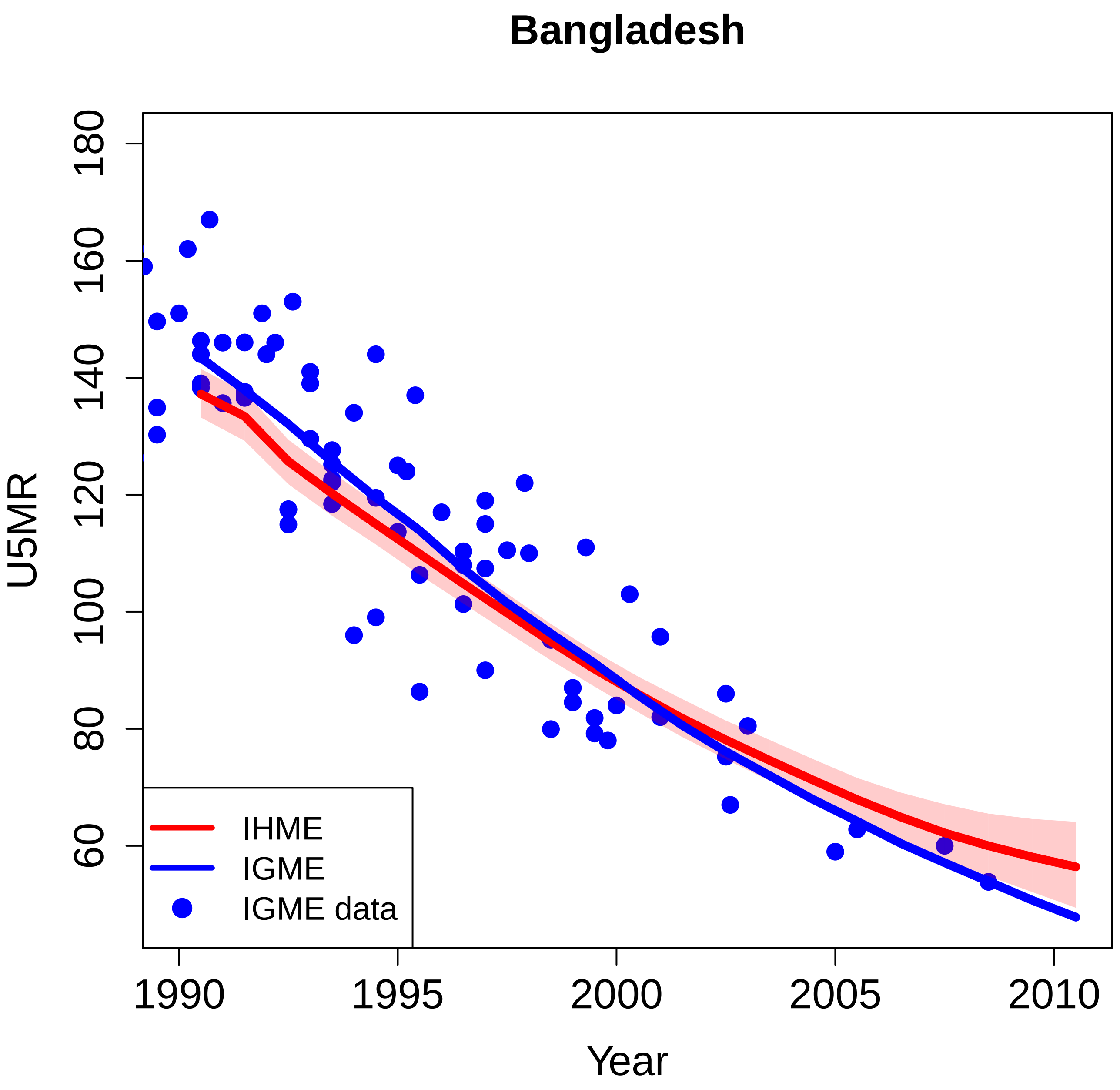

Bangladesh
U5MR
Year
180
160
140
120
100
80
60
1990
1995
2000
2005
2010
IHME
IGME
IGME data

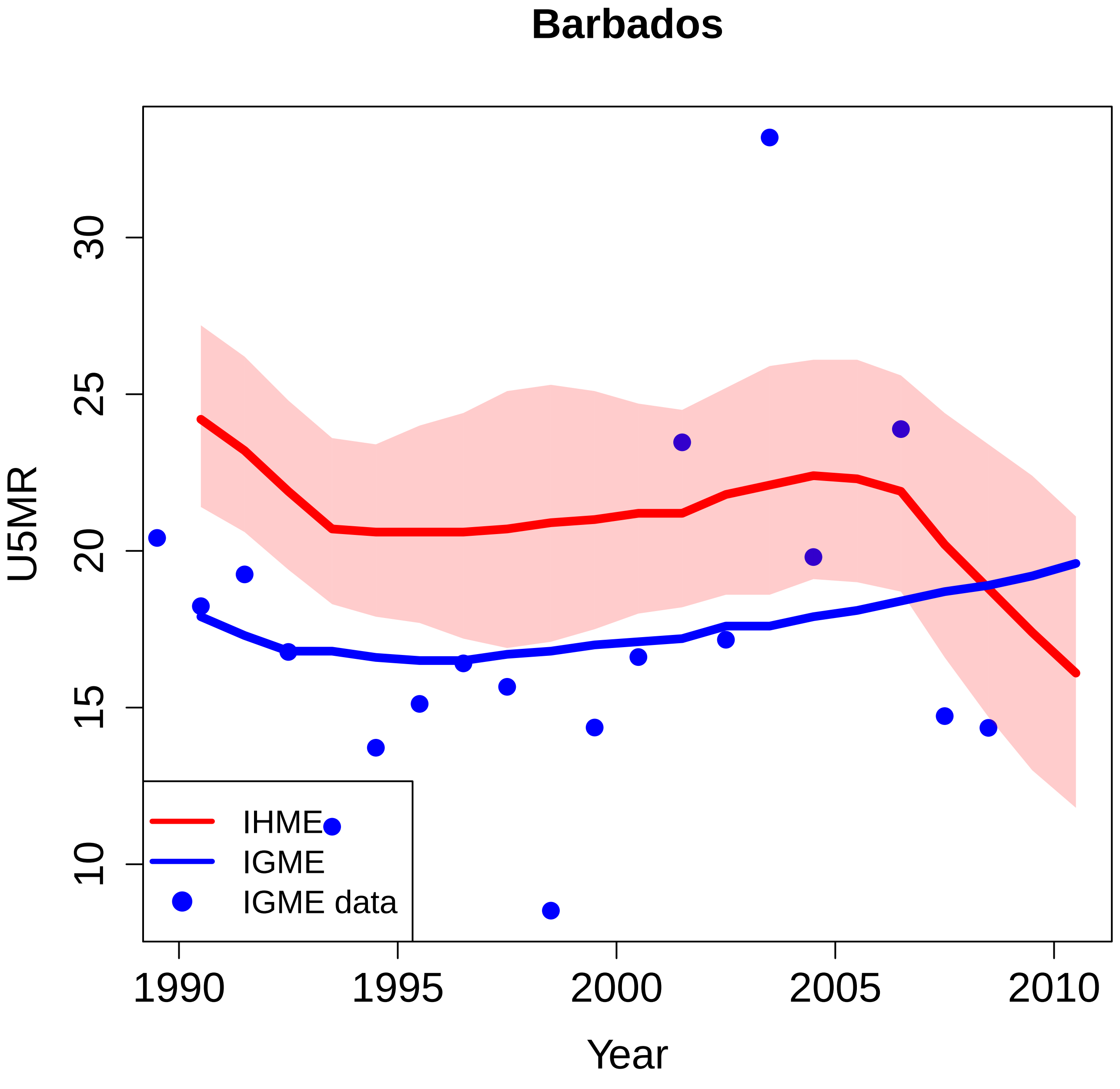

Barbados
U5MR
10
15
20
25
30
1990
1995
2000
2005
2010
Year
IHME
IGME
IGME data

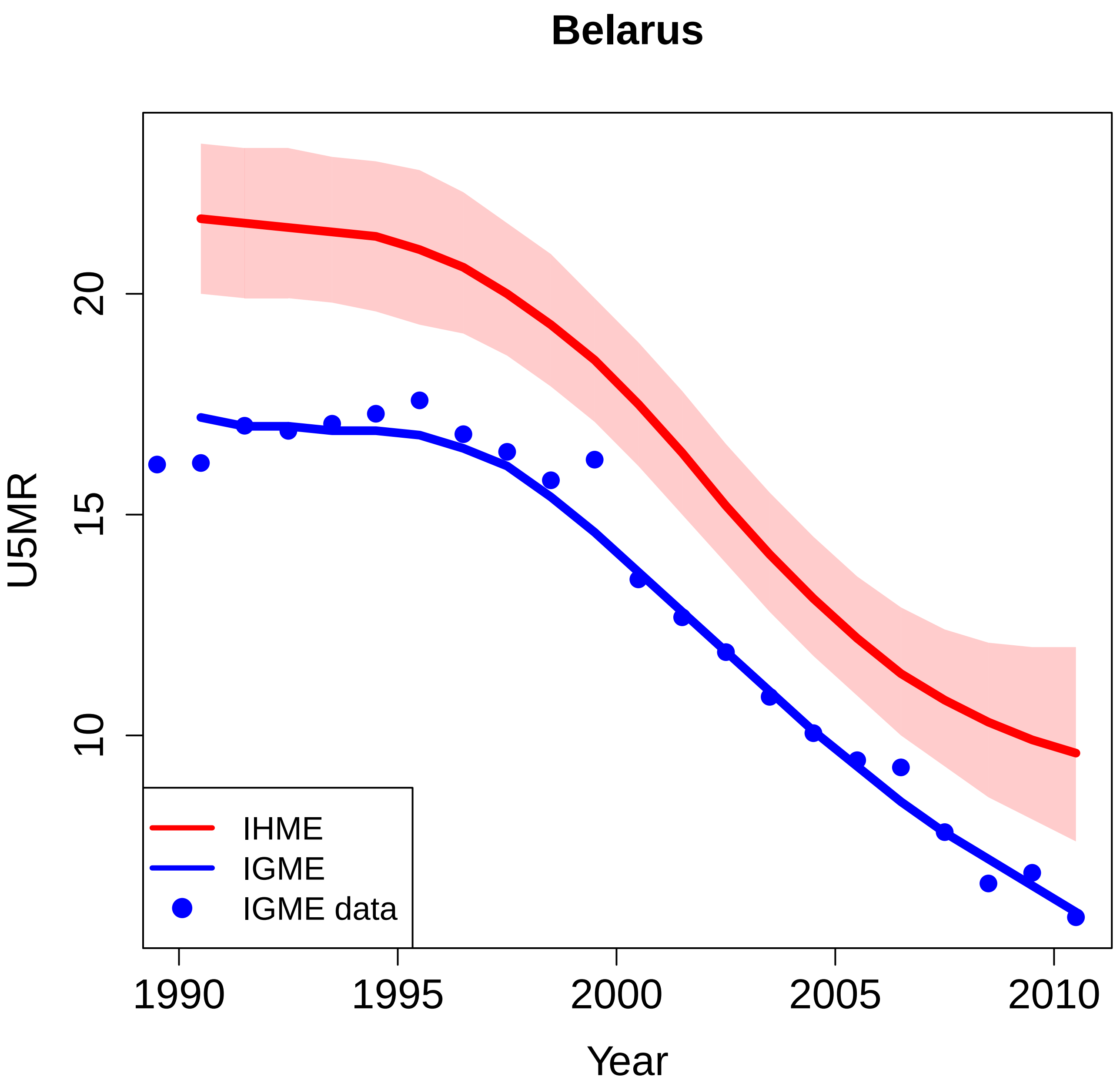
Belarus
U5MR
20
15
10
1990
1995
2000
2005
2010
Year
IHME
IGME
IGME data

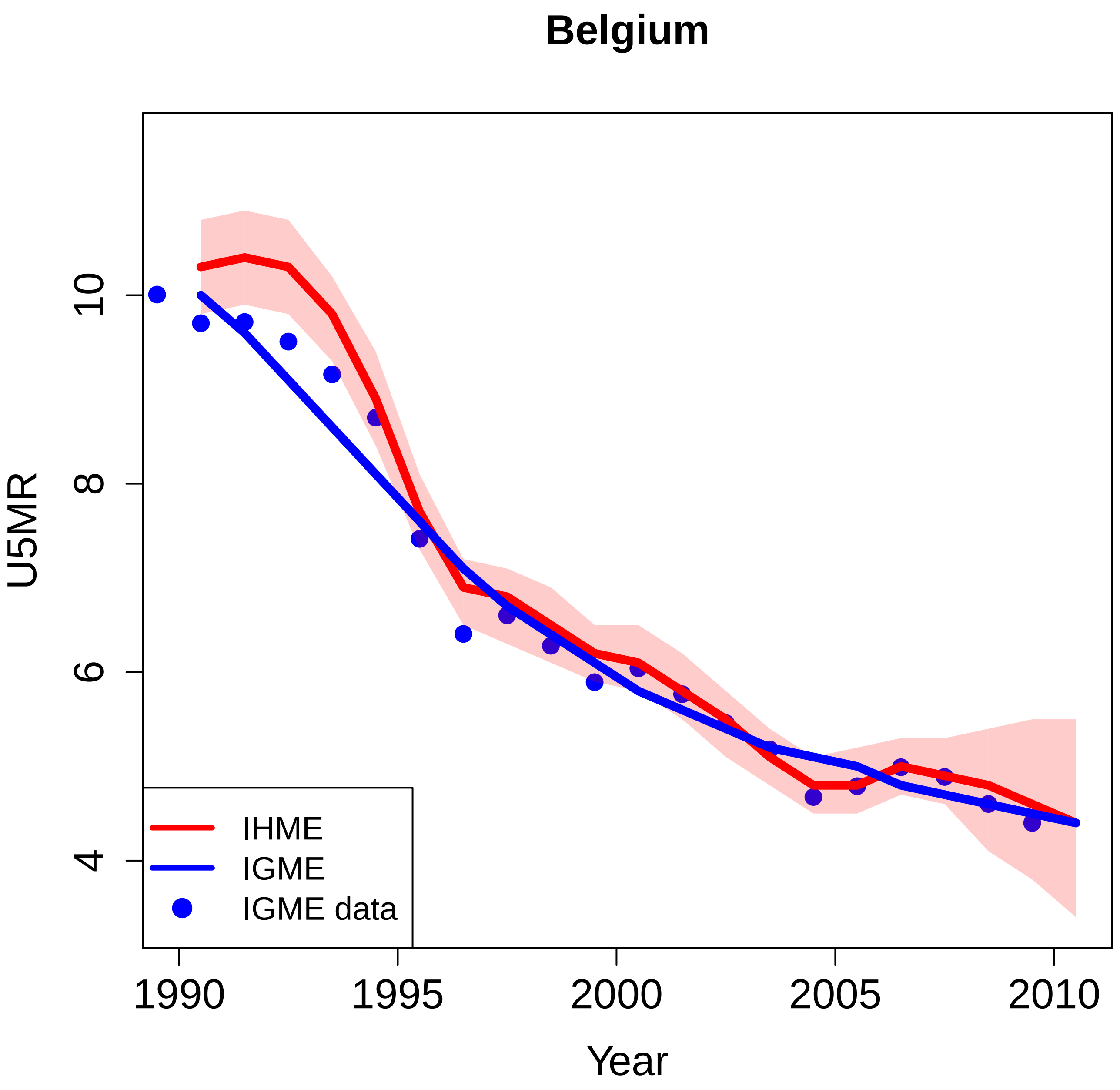
Belgium
U5MR
10
8
6
4
1990
1995
2000
2005
2010
Year
IHME
IGME
IGME data

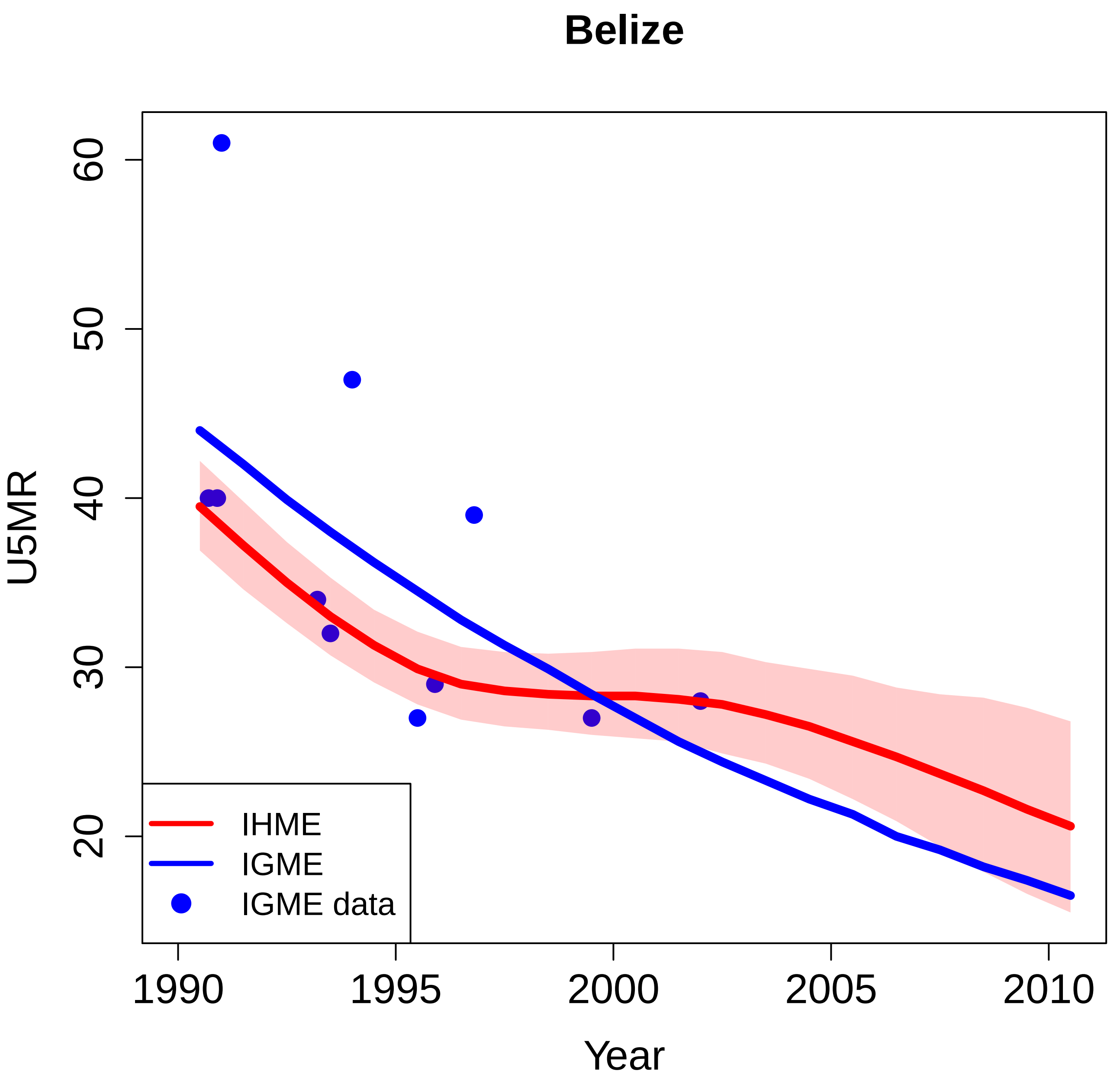
Belize
U5MR
60
50
40
30
20
1990
1995
2000
2005
2010
Year
IHME
IGME
IGME data

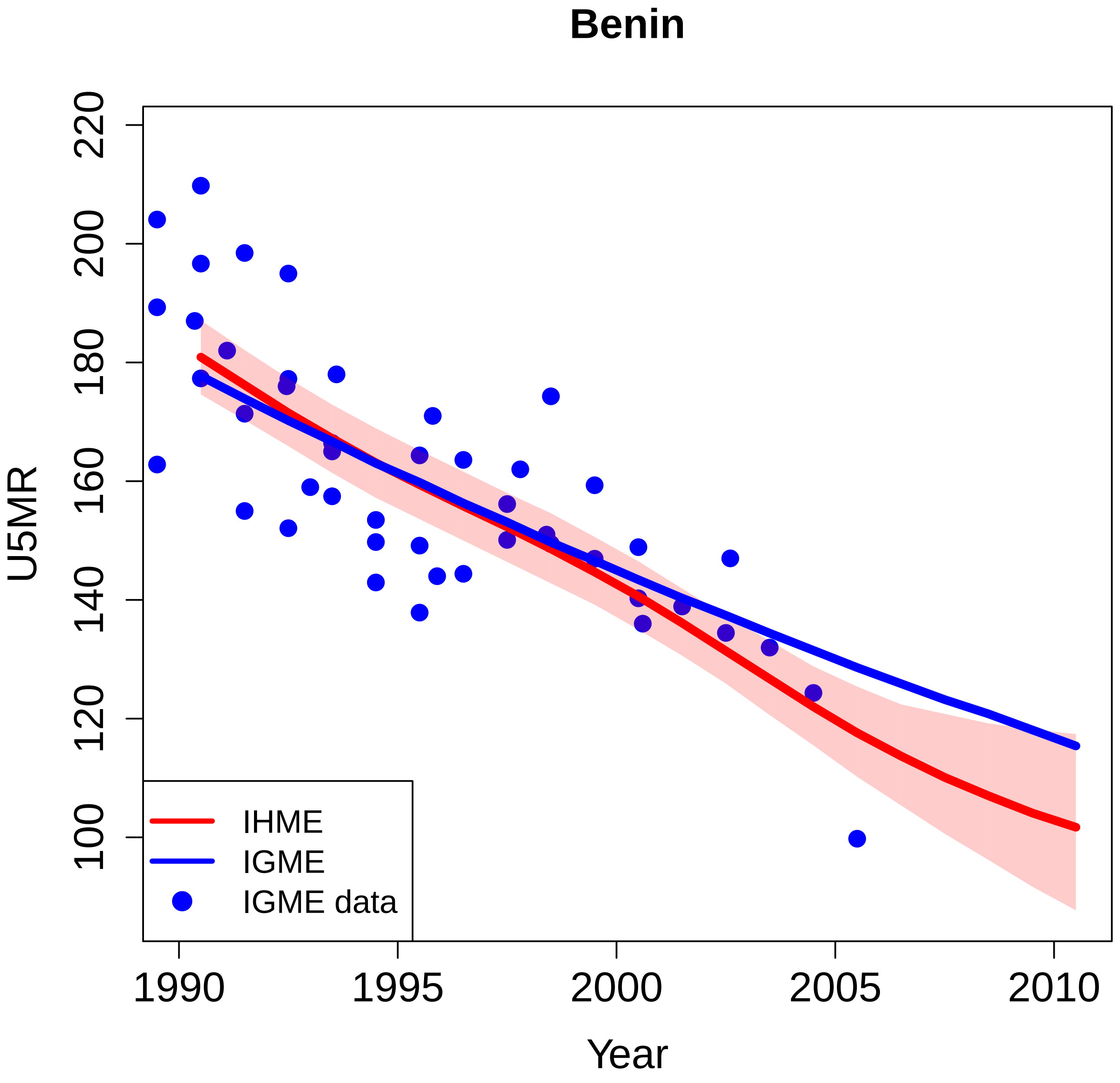
Benin
U5MR
Year
100
120
140
160
180
200
220
1990
1995
2000
2005
2010
IHME
IGME
IGME data

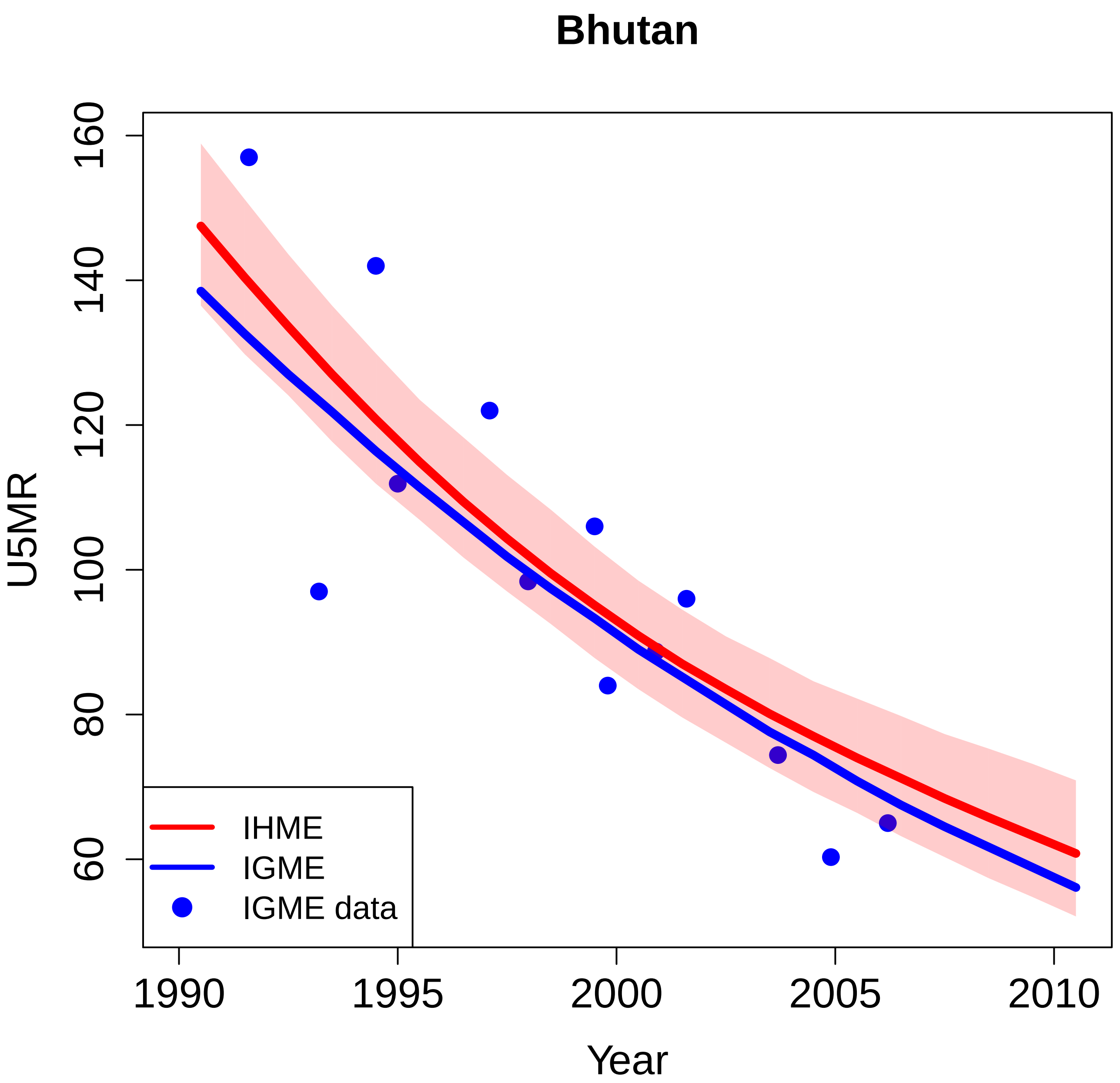
Bhutan
U5MR
160
140
120
100
80
60
1990
1995
2000
2005
2010
Year
IHME
IGME
IGME data

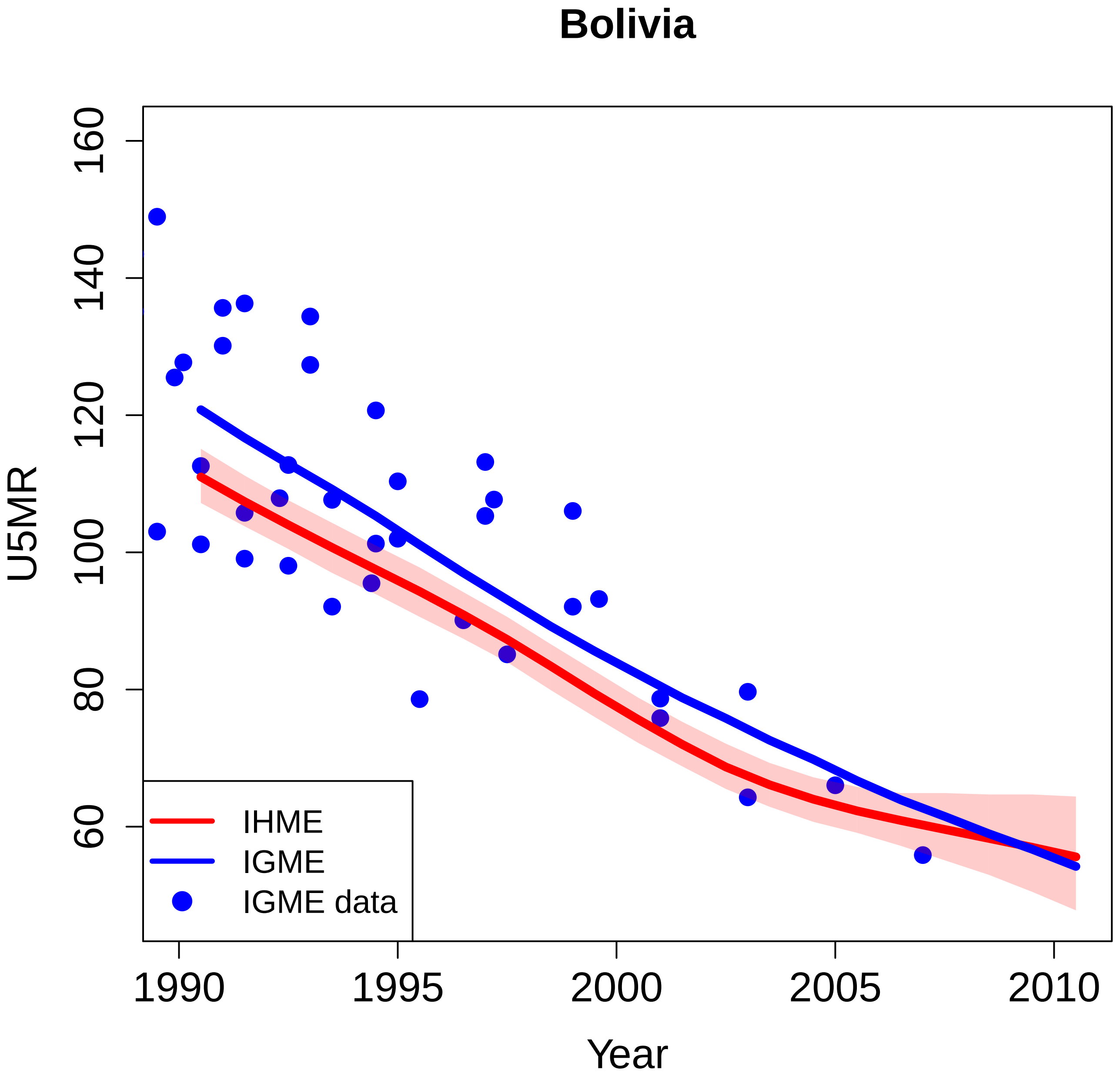
Bolivia
160
140
120
100
80
60
U5MR
1990
1995
2000
2005
2010
Year
IHME
IGME
IGME data

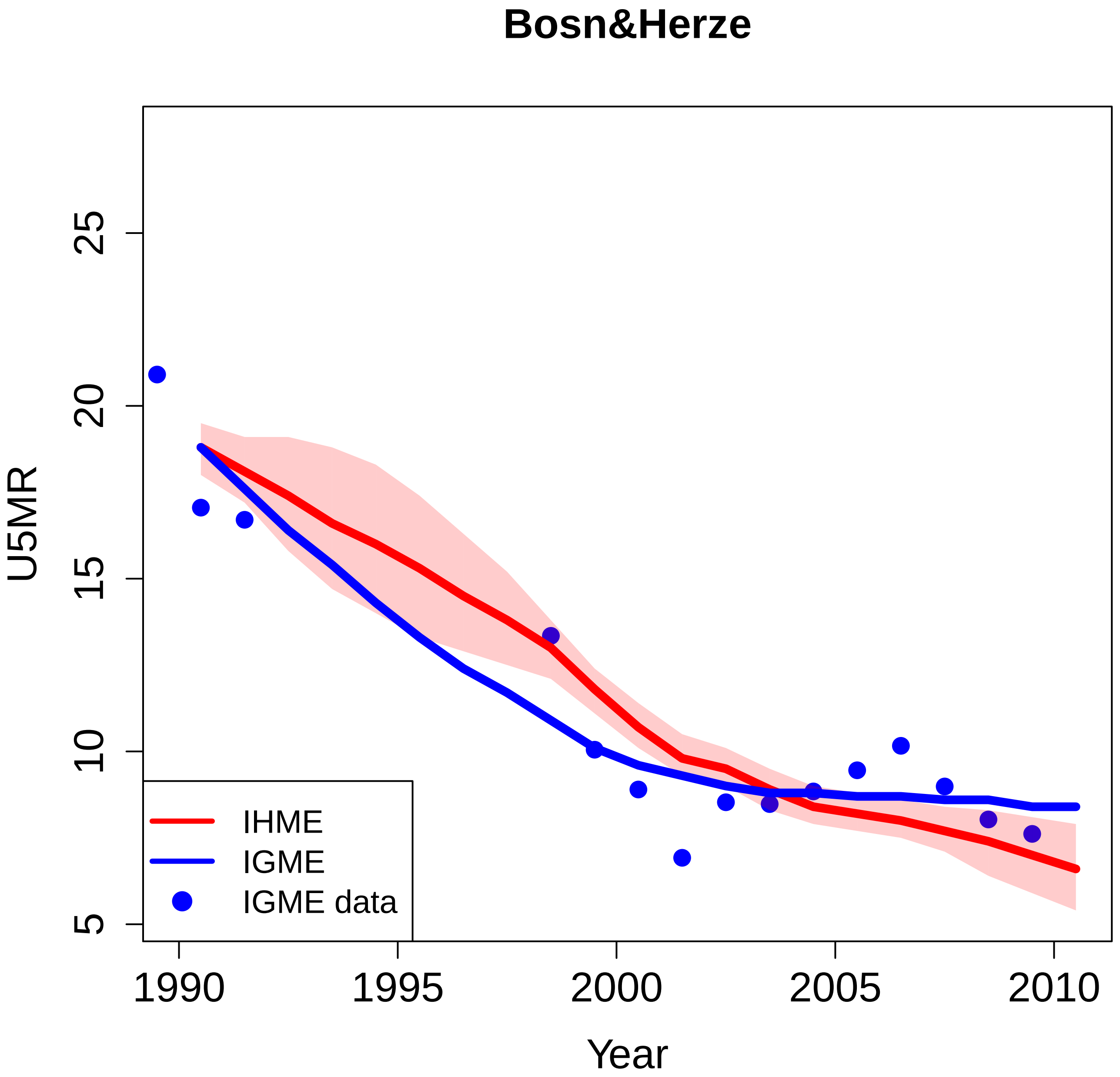

Bosn&Herze
U5MR
Year
IHME
IGME
IGME data
5
10
15
20
25
1990
1995
2000
2005
2010

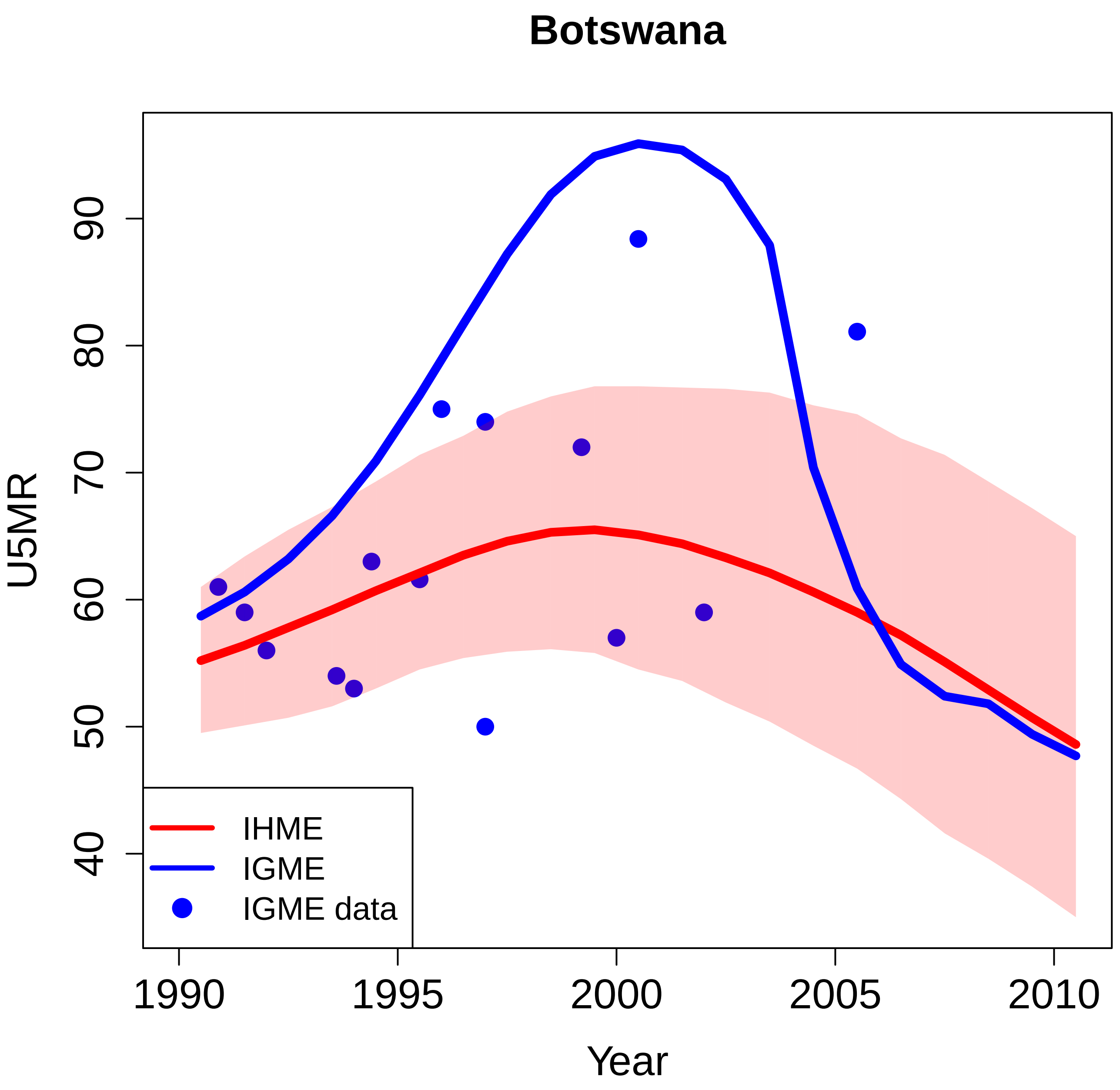

Botswana
U5MR
Year
90
80
70
60
50
40
1990
1995
2000
2005
2010
IHME
IGME
IGME data

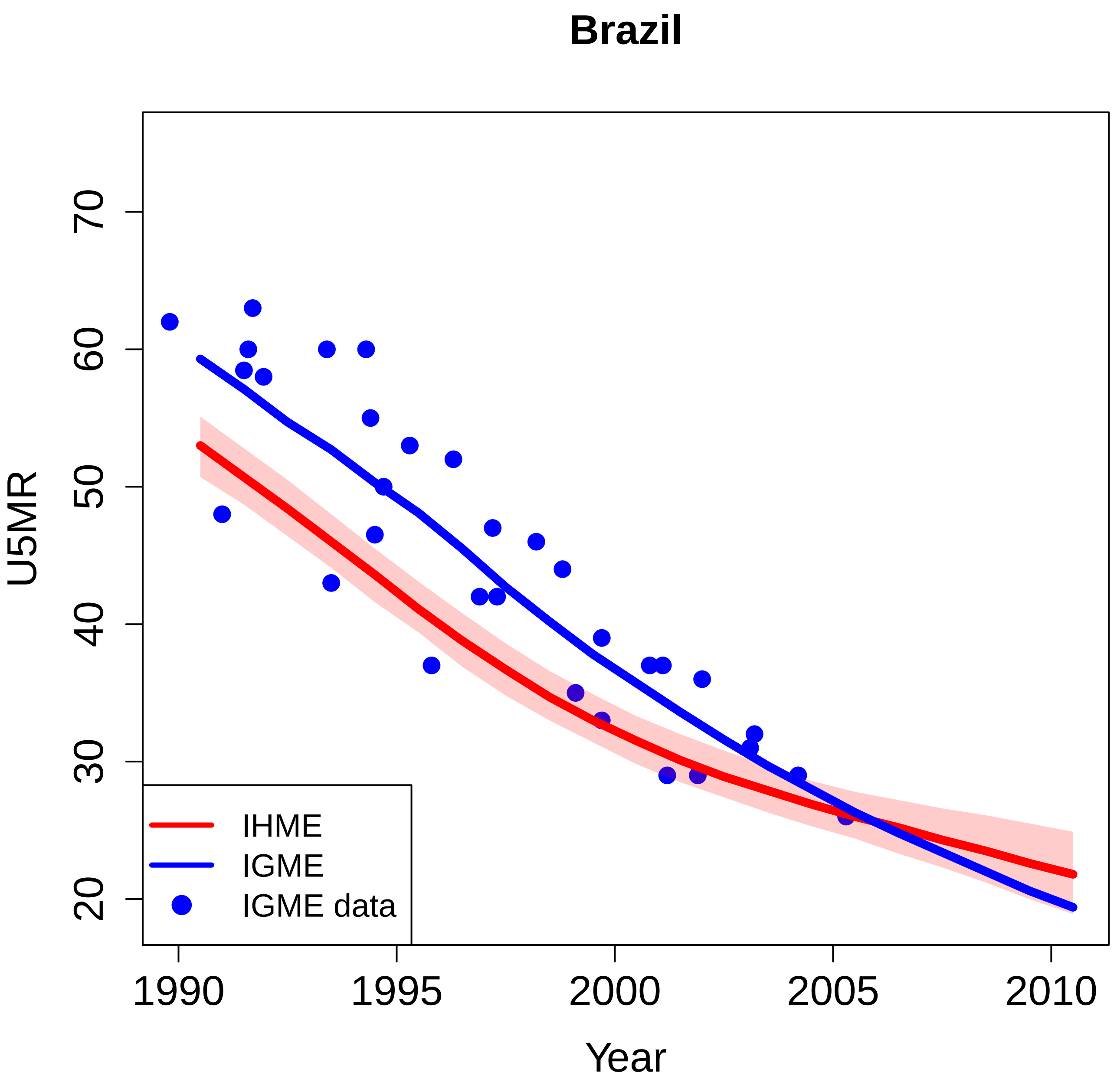
Brazil
U5MR
70
60
50
40
30
20
1990
1995
2000
2005
2010
Year
IHME
IGME
IGME data

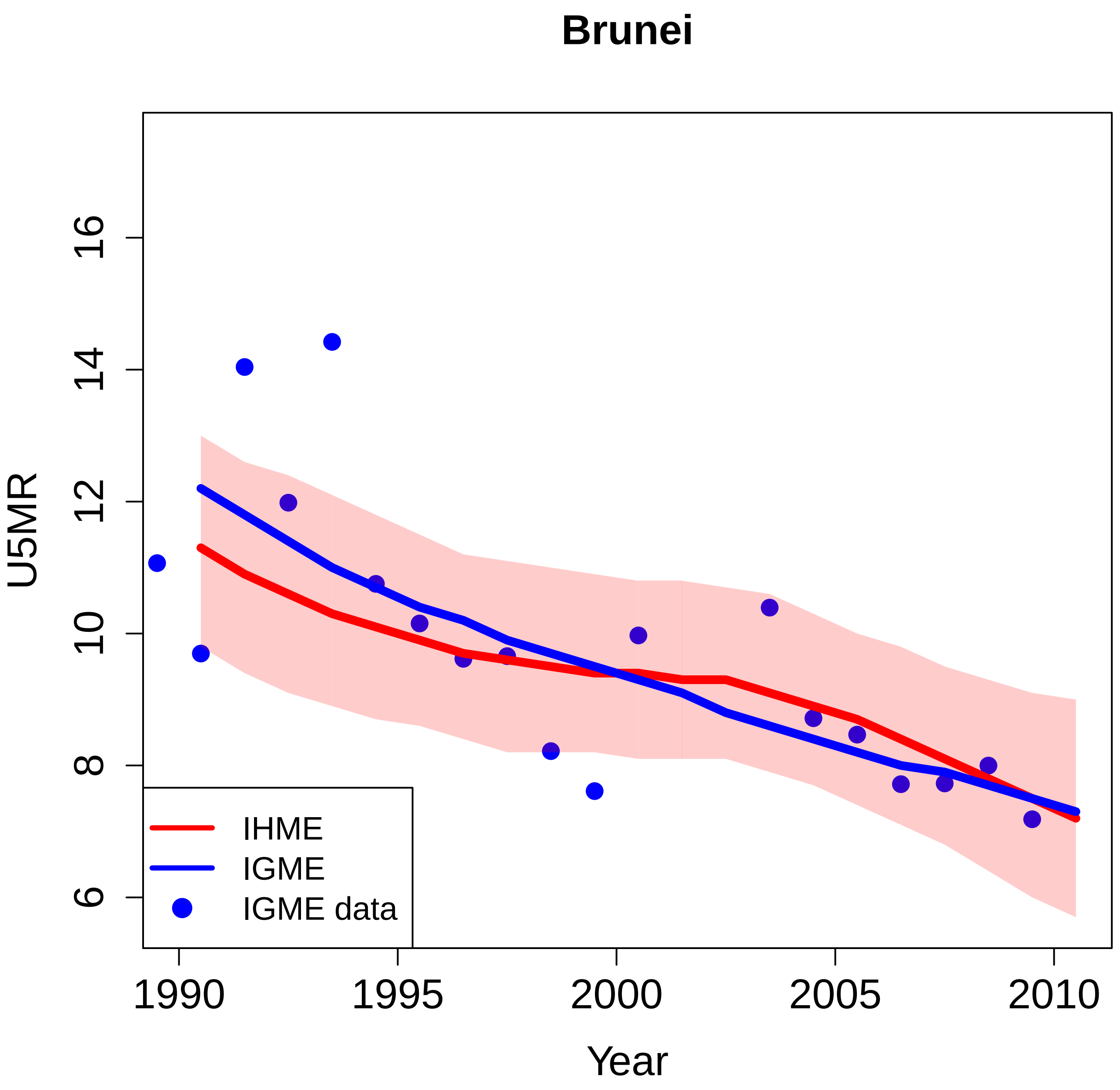

Brunei
U5MR
16
14
12
10
8
6
1990
1995
2000
2005
2010
Year
IHME
IGME
IGME data

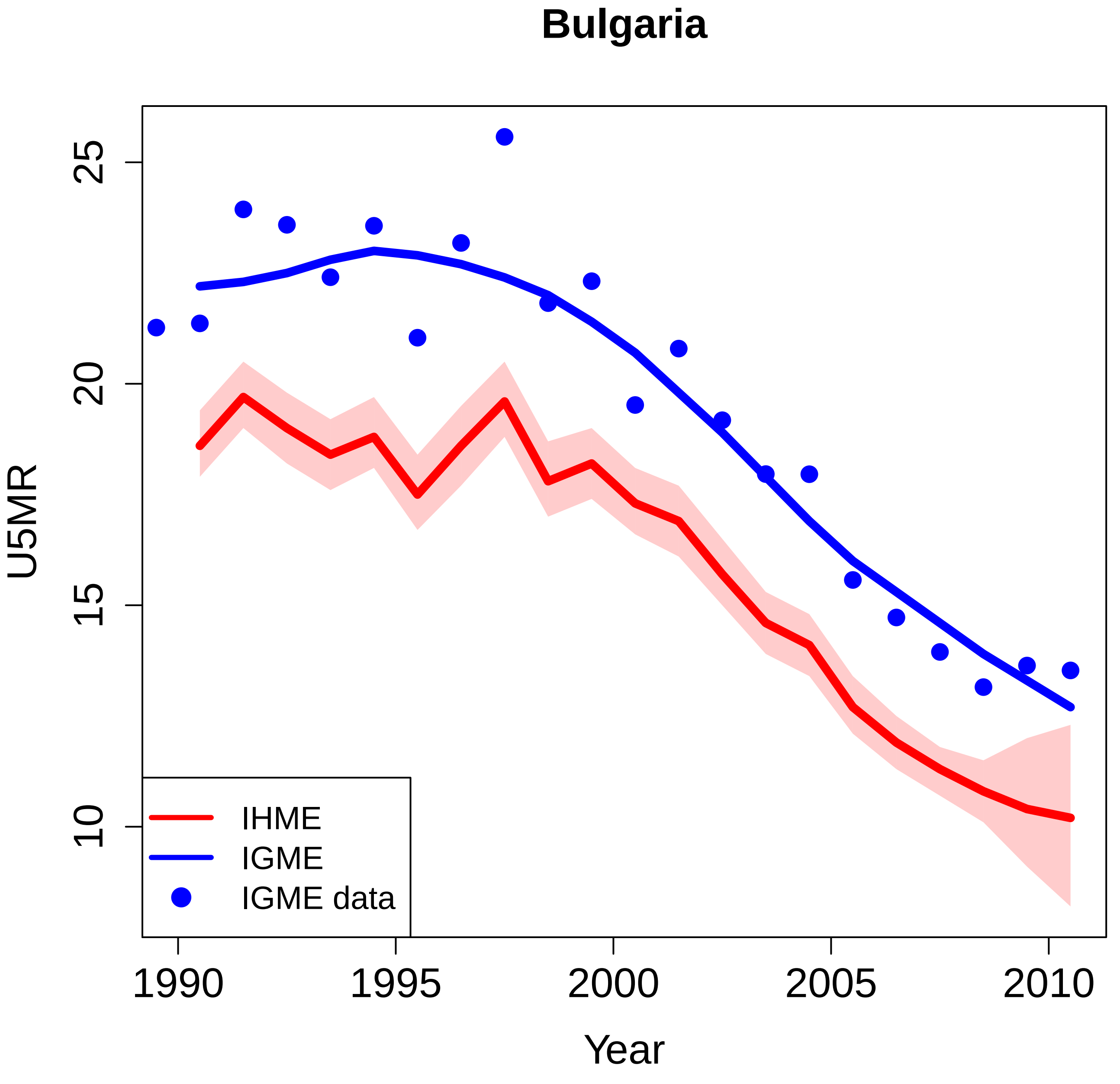
Bulgaria
U5MR
25
20
15
10
1990
1995
2000
2005
2010
Year
IHME
IGME
IGME data

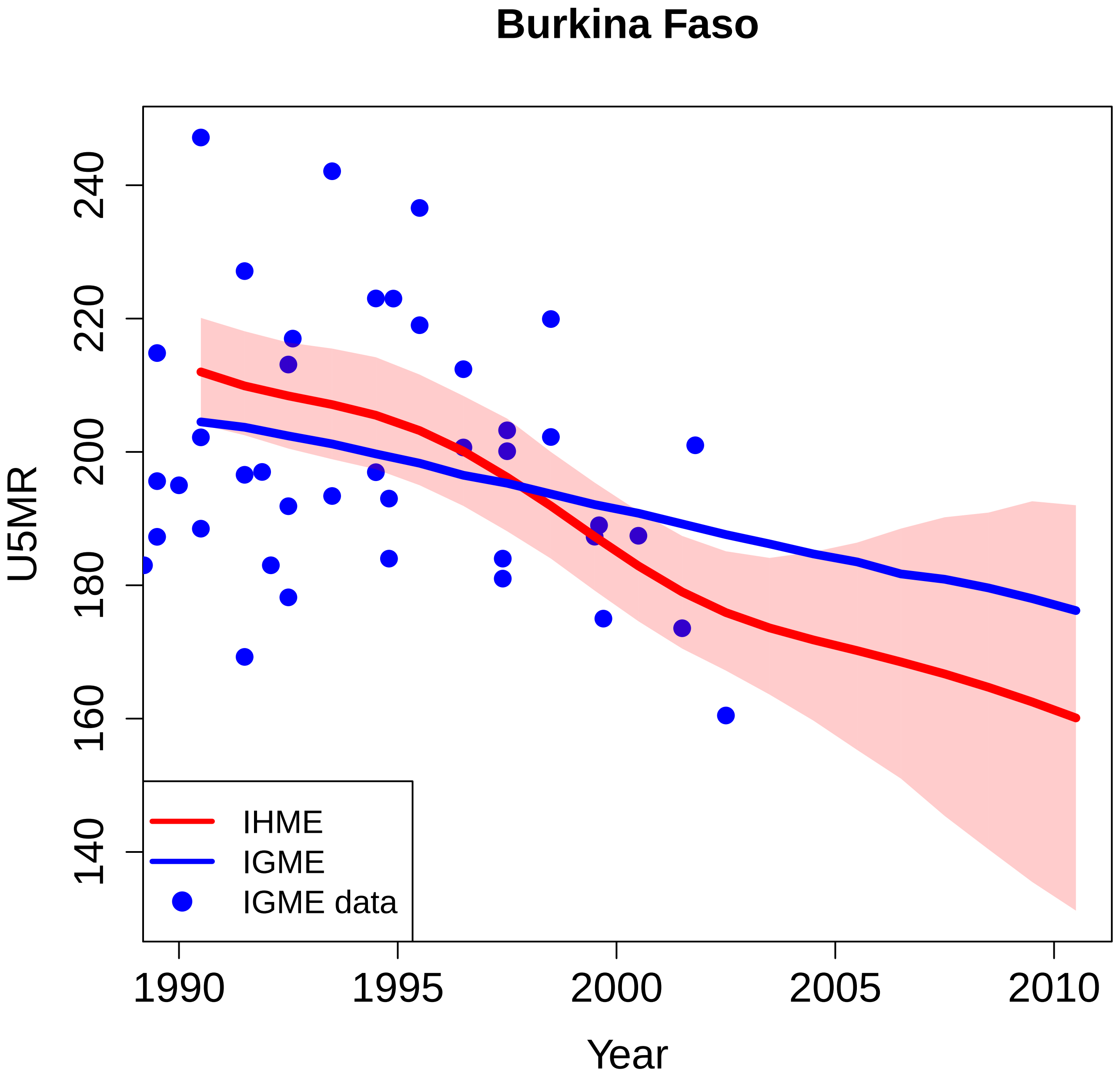

Burkina Faso
U5MR
240
220
200
180
160
140
1990
1995
2000
2005
2010
Year
IHME
IGME
IGME data

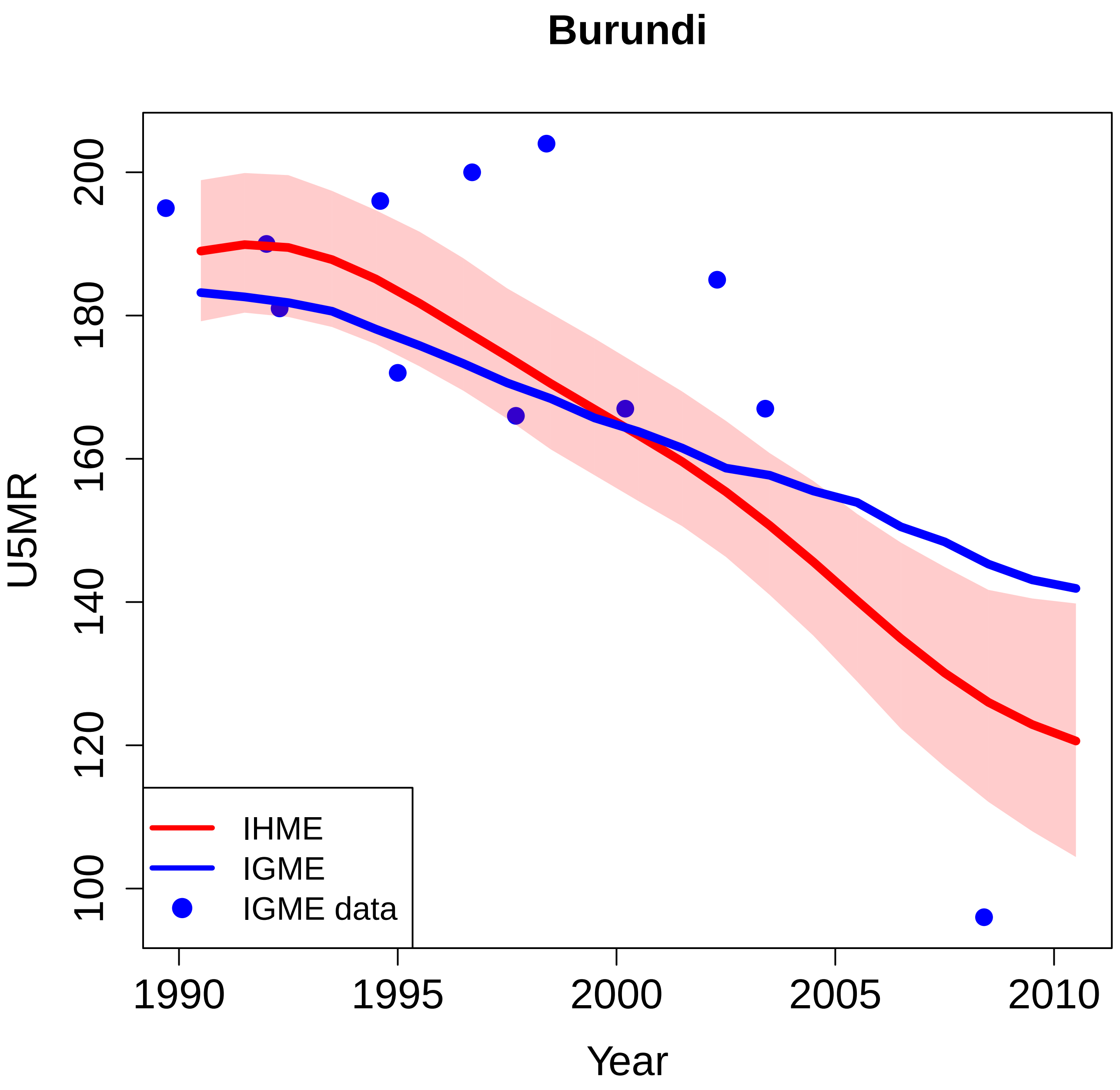

Burundi
U5MR
200
180
160
140
120
100
1990
1995
2000
2005
2010
Year
IHME
IGME
IGME data

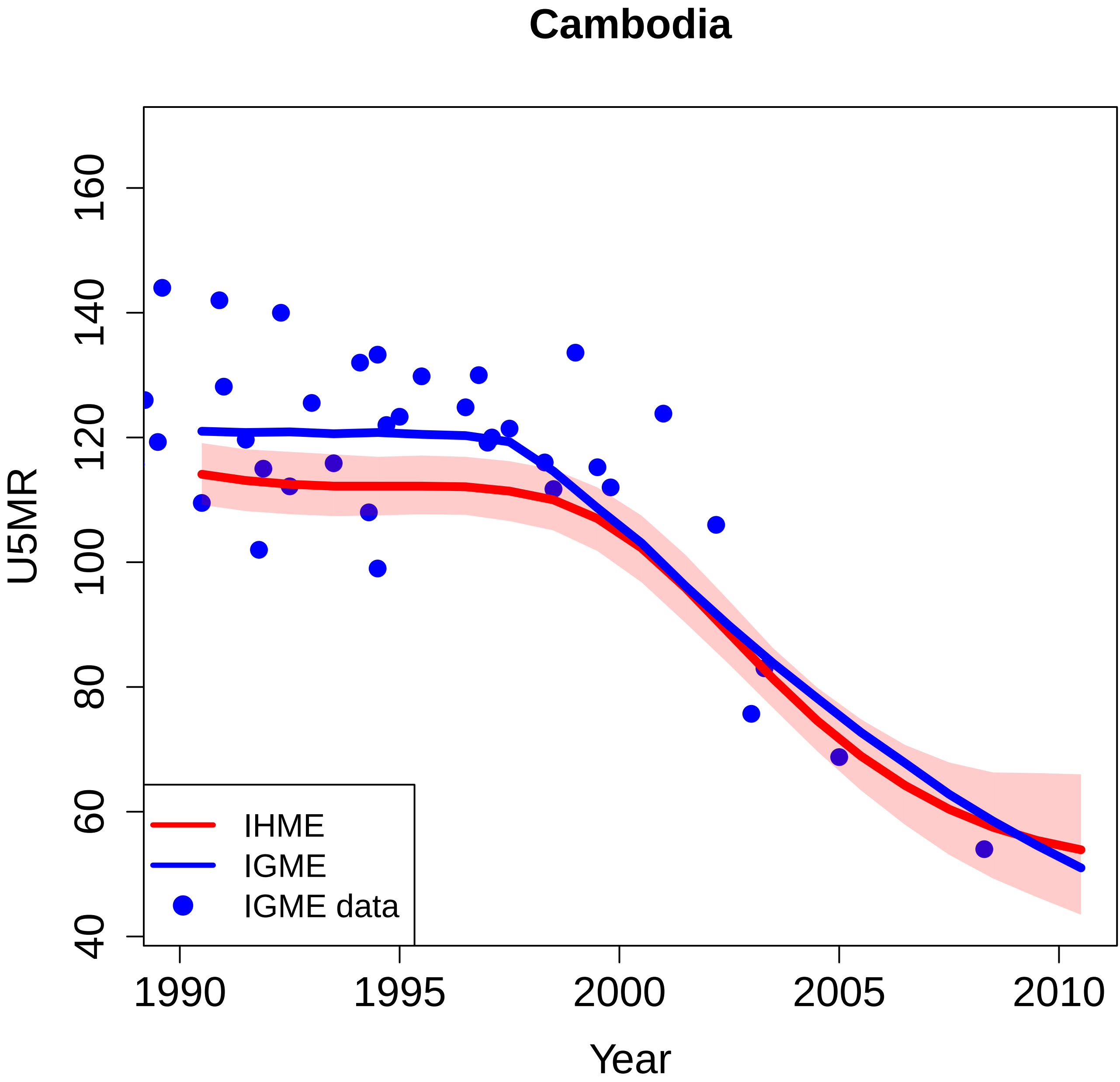

Cambodia
U5MR
Year
40
60
80
100
120
140
160
1990
1995
2000
2005
2010
IHME
IGME
IGME data

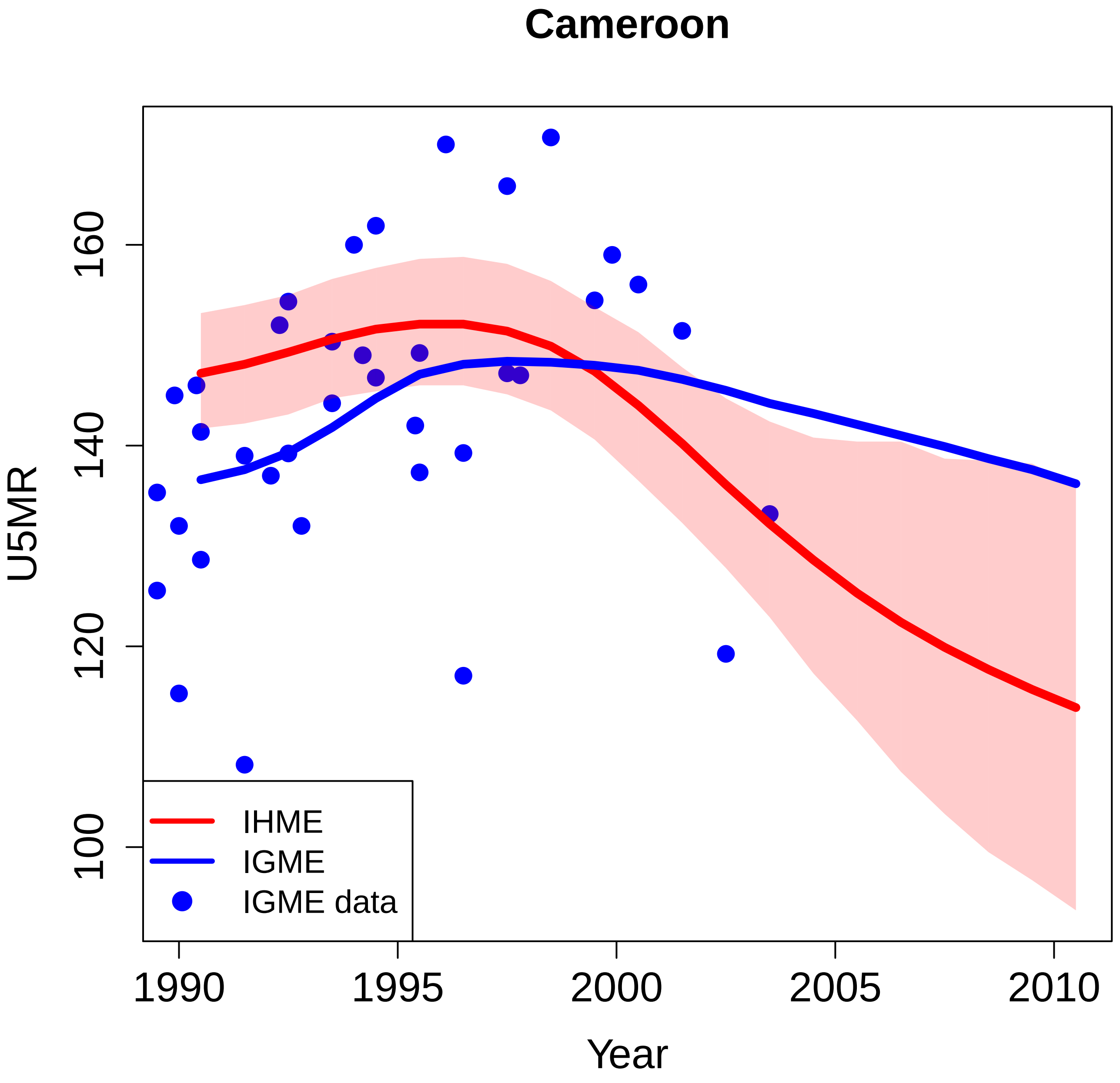

Cameroon
U5MR
100
120
140
160
1990
1995
2000
2005
2010
Year
IHME
IGME
IGME data

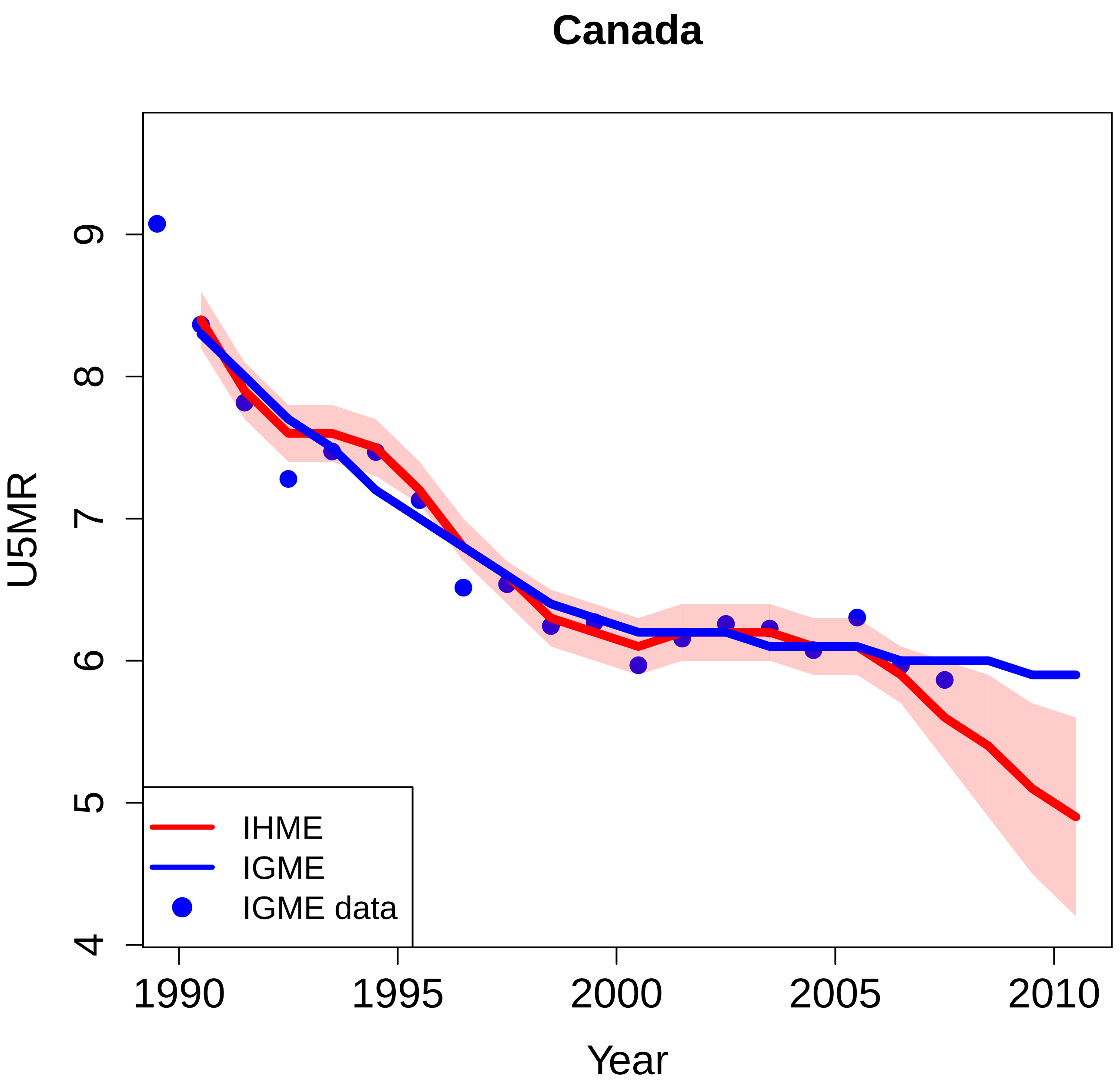

Canada
U5MR
Year
4
5
6
7
8
9
1990
1995
2000
2005
2010
IHME
IGME
IGME data

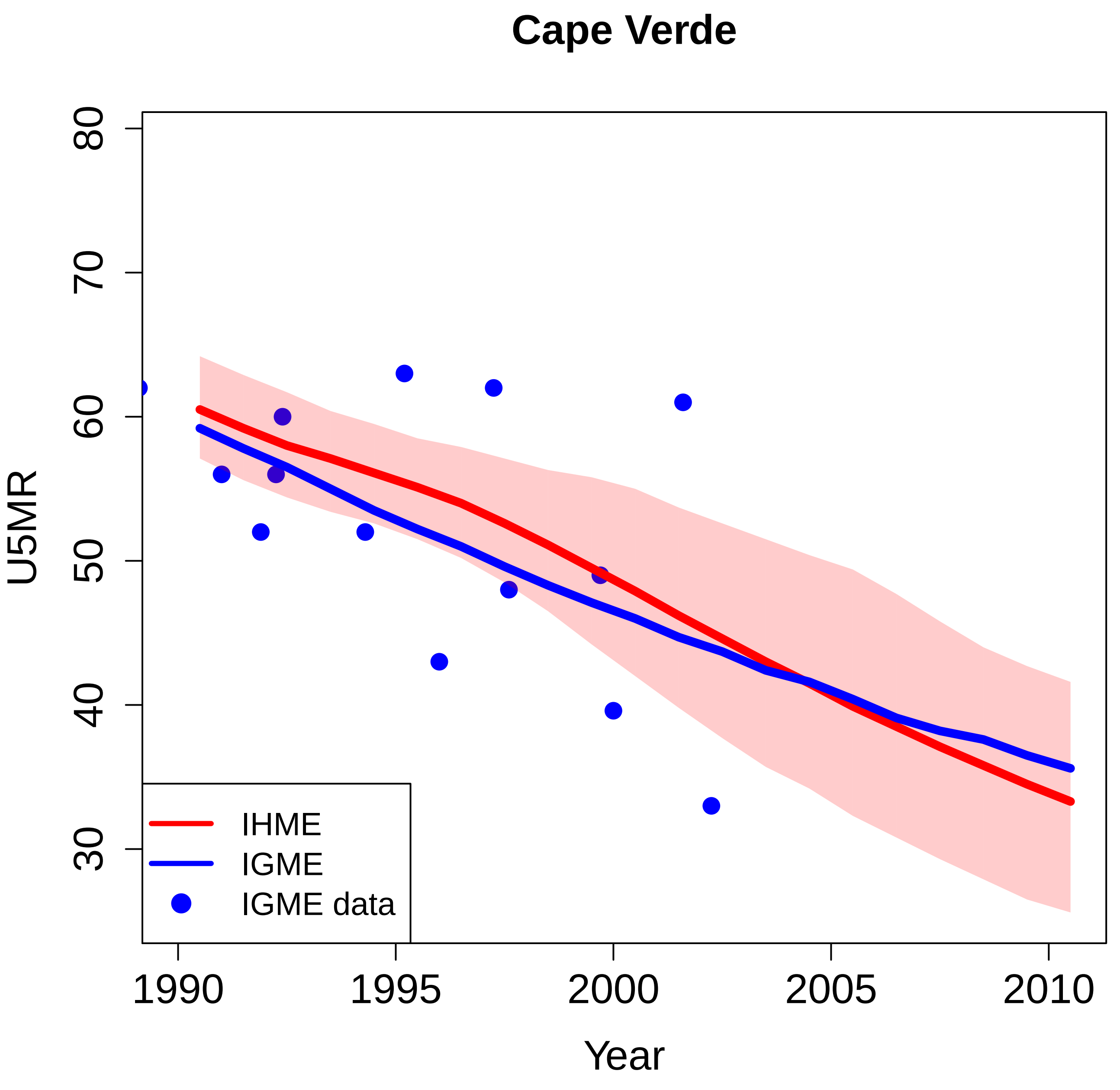
Cape Verde
U5MR
80
70
60
50
40
30
1990
1995
2000
2005
2010
Year
IHME
IGME
IGME data

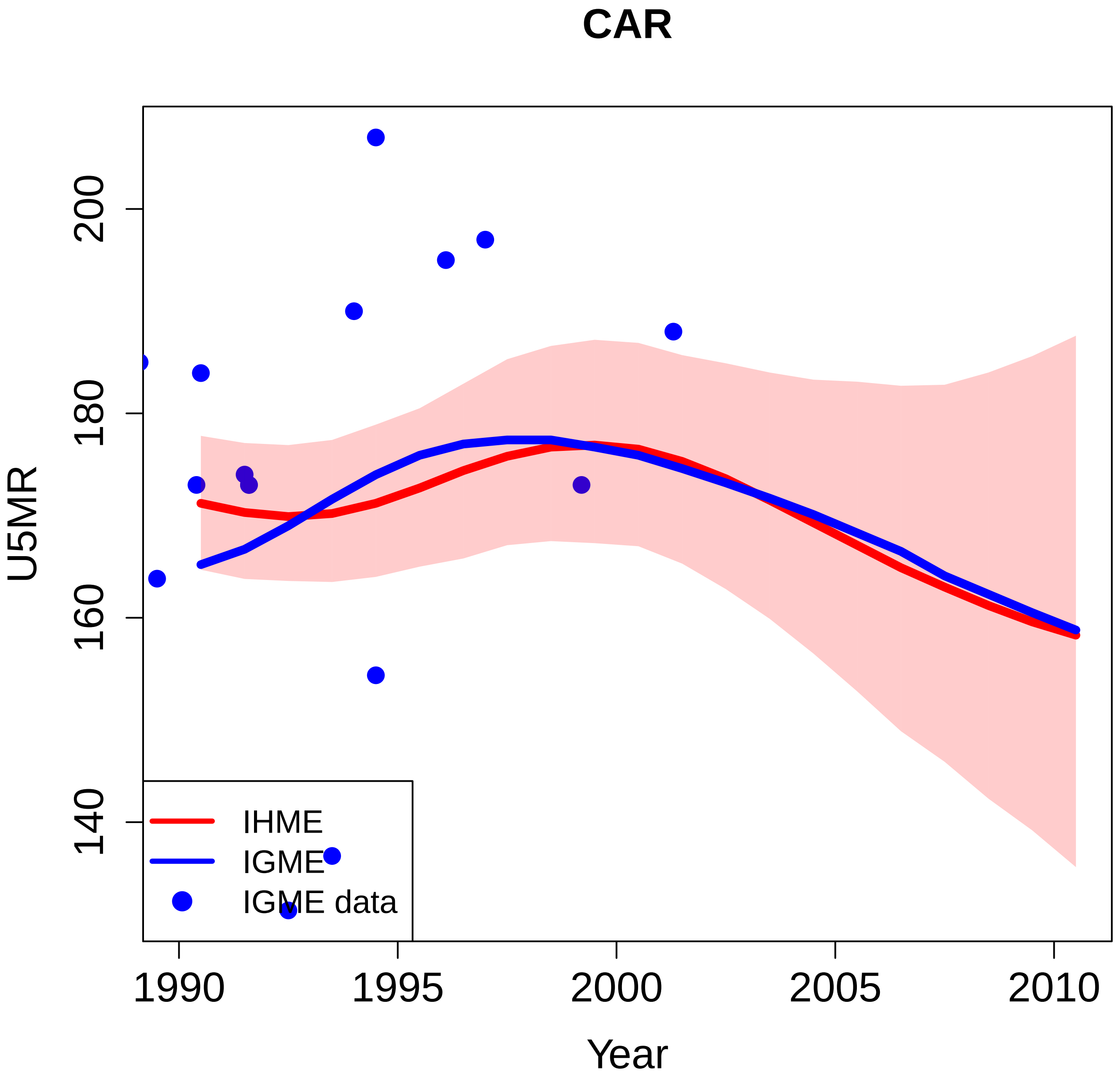

CAR
U5MR
200
180
160
140
1990
1995
2000
2005
2010
Year
IHME
IGME
IGME data

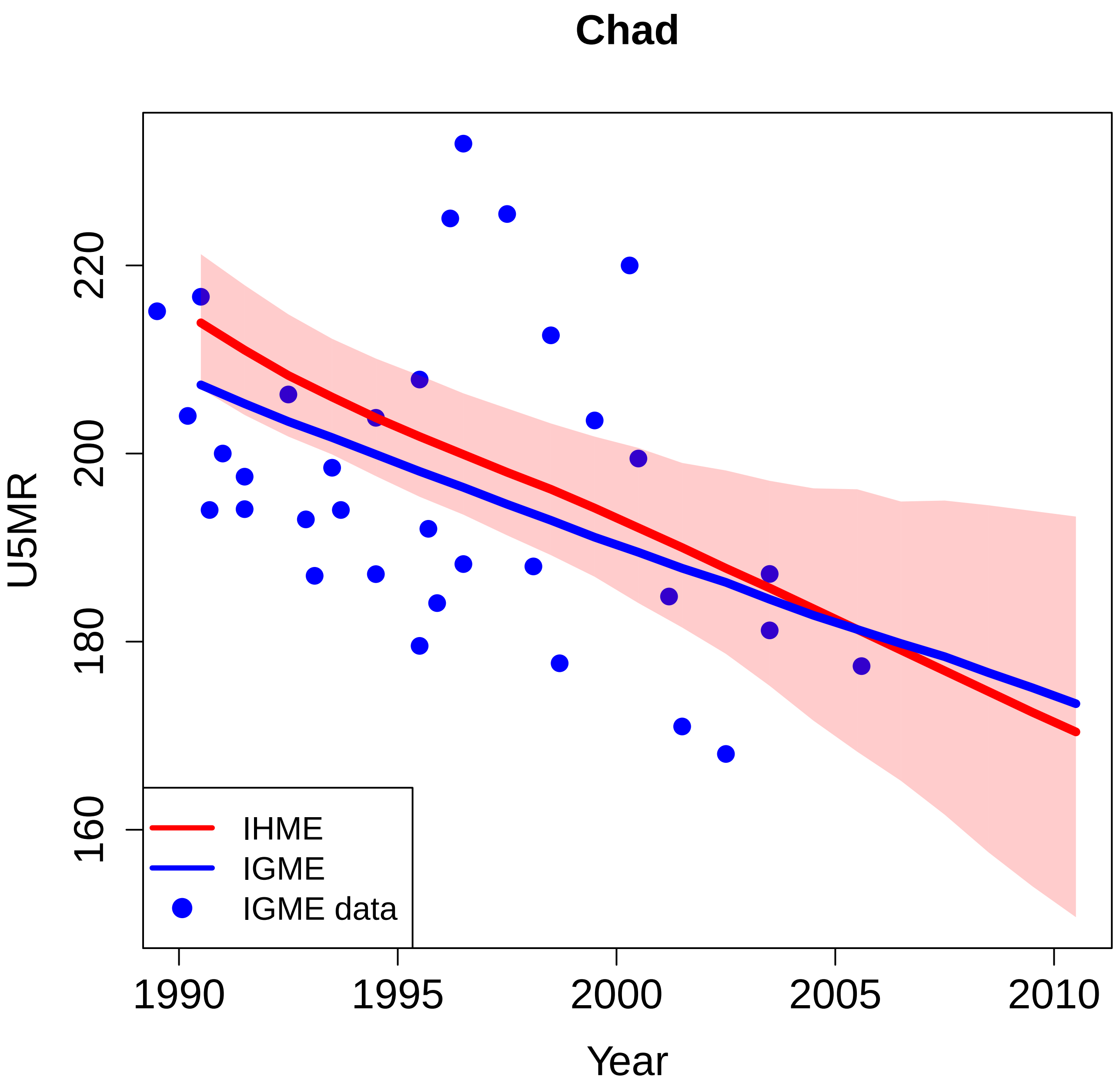
Chad
U5MR
160
180
200
220
1990
1995
2000
2005
2010
Year
IHME
IGME
IGME data

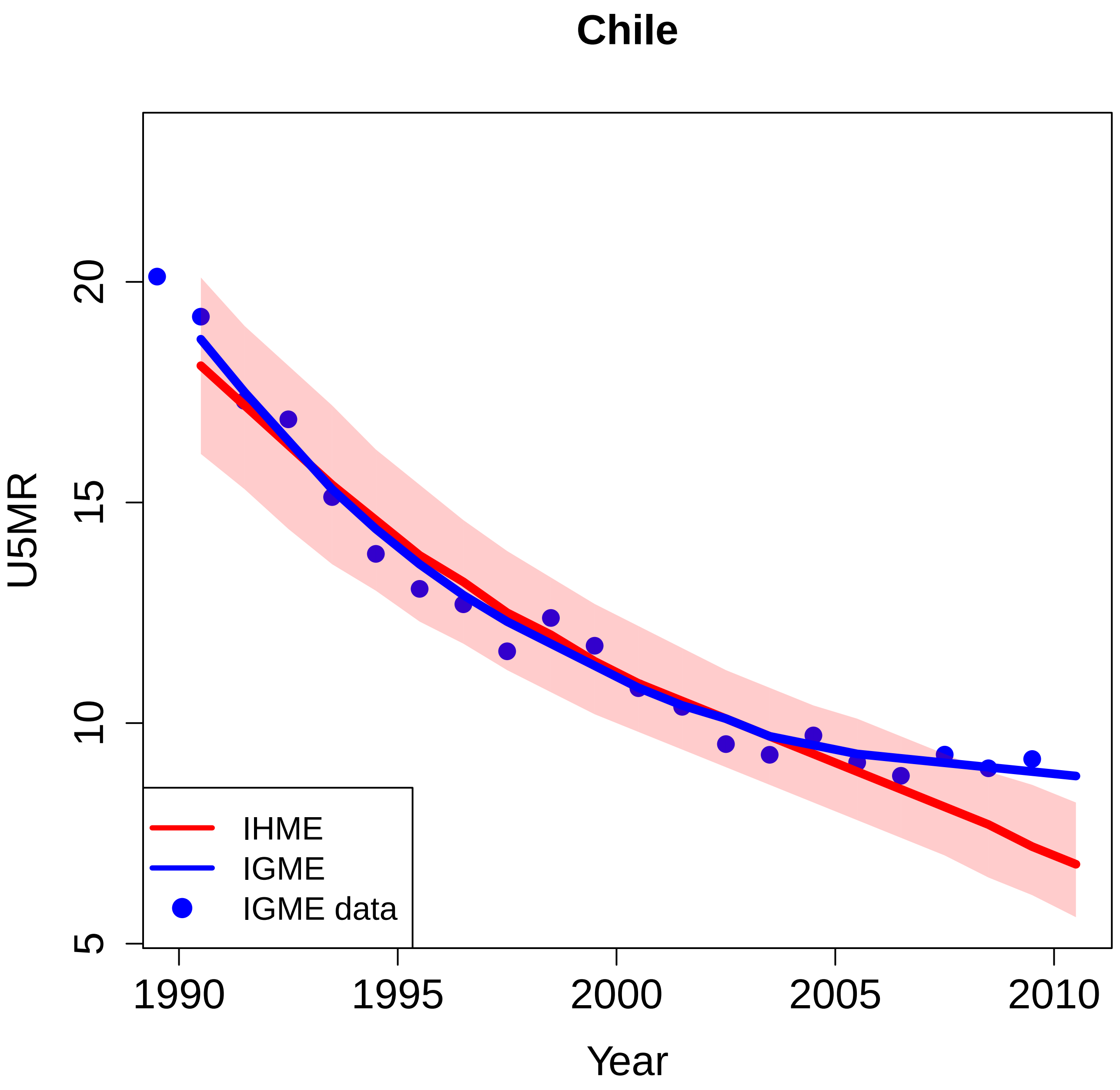
Chile
U5MR
20
15
10
5
1990
1995
2000
2005
2010
Year
IHME
IGME
IGME data

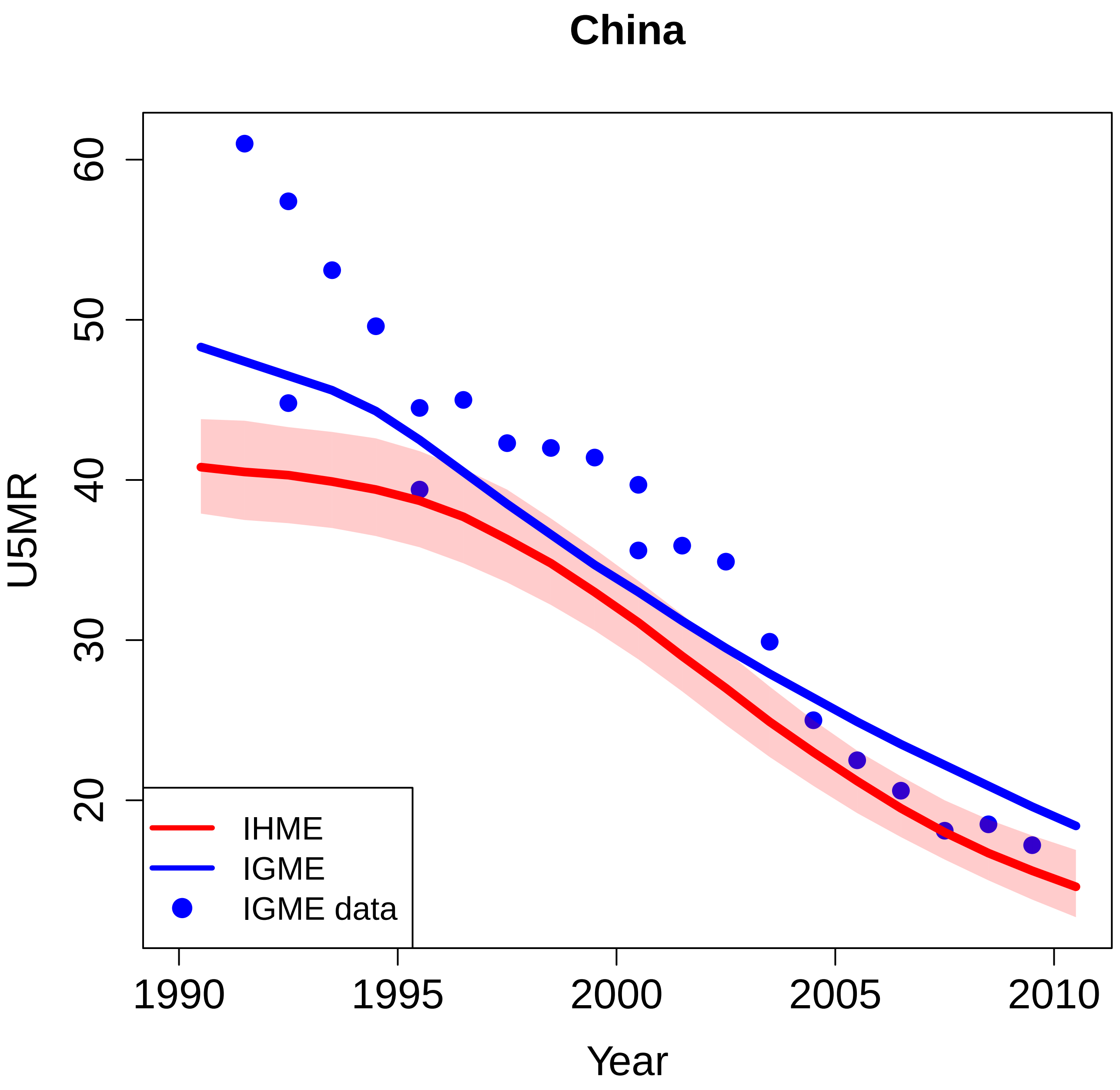

China
U5MR
60
50
40
30
20
1990
1995
2000
2005
2010
Year
IHME
IGME
IGME data

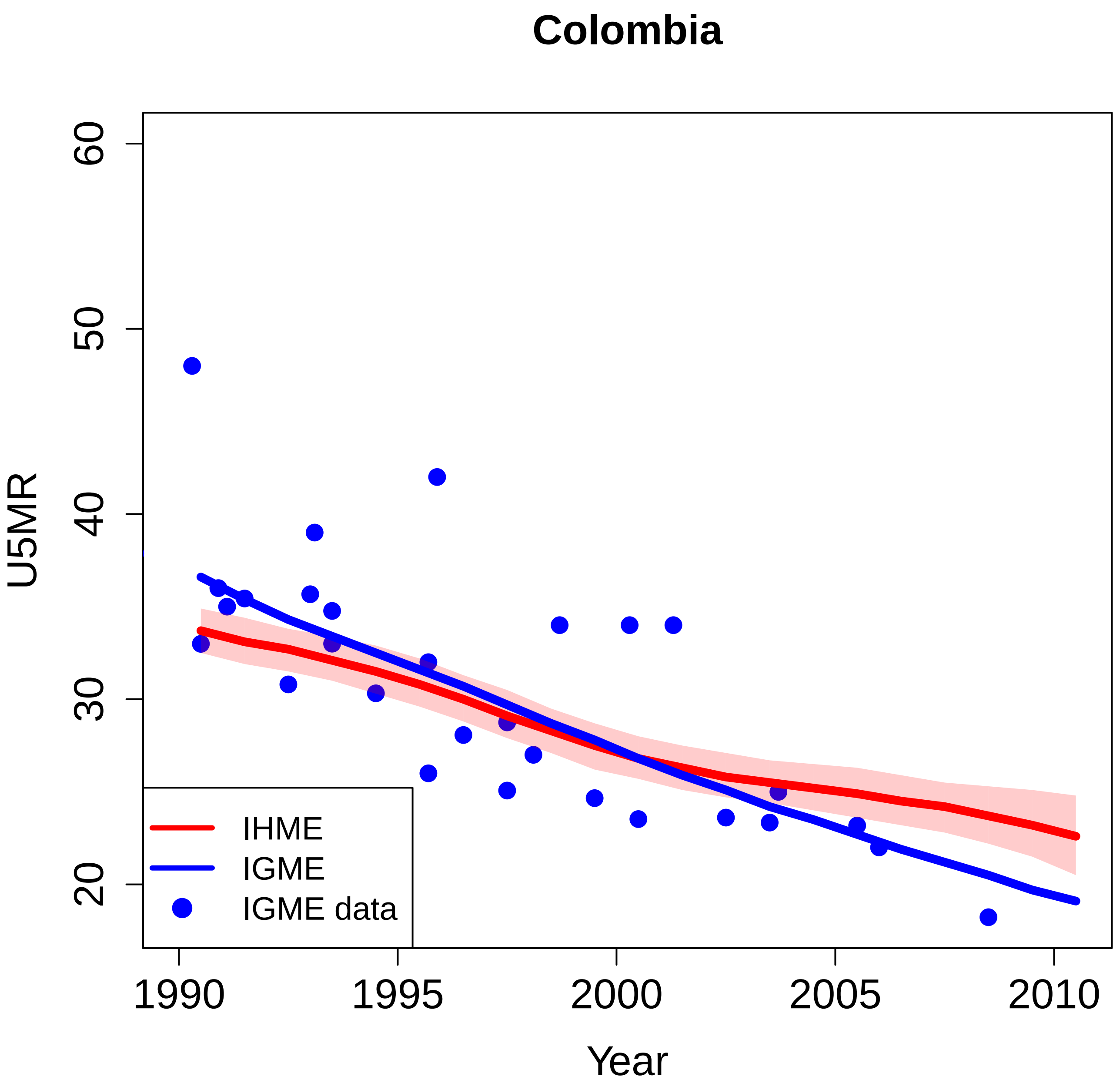

Colombia
U5MR
60
50
40
30
20
1990
1995
2000
2005
2010
Year
IHME
IGME
IGME data

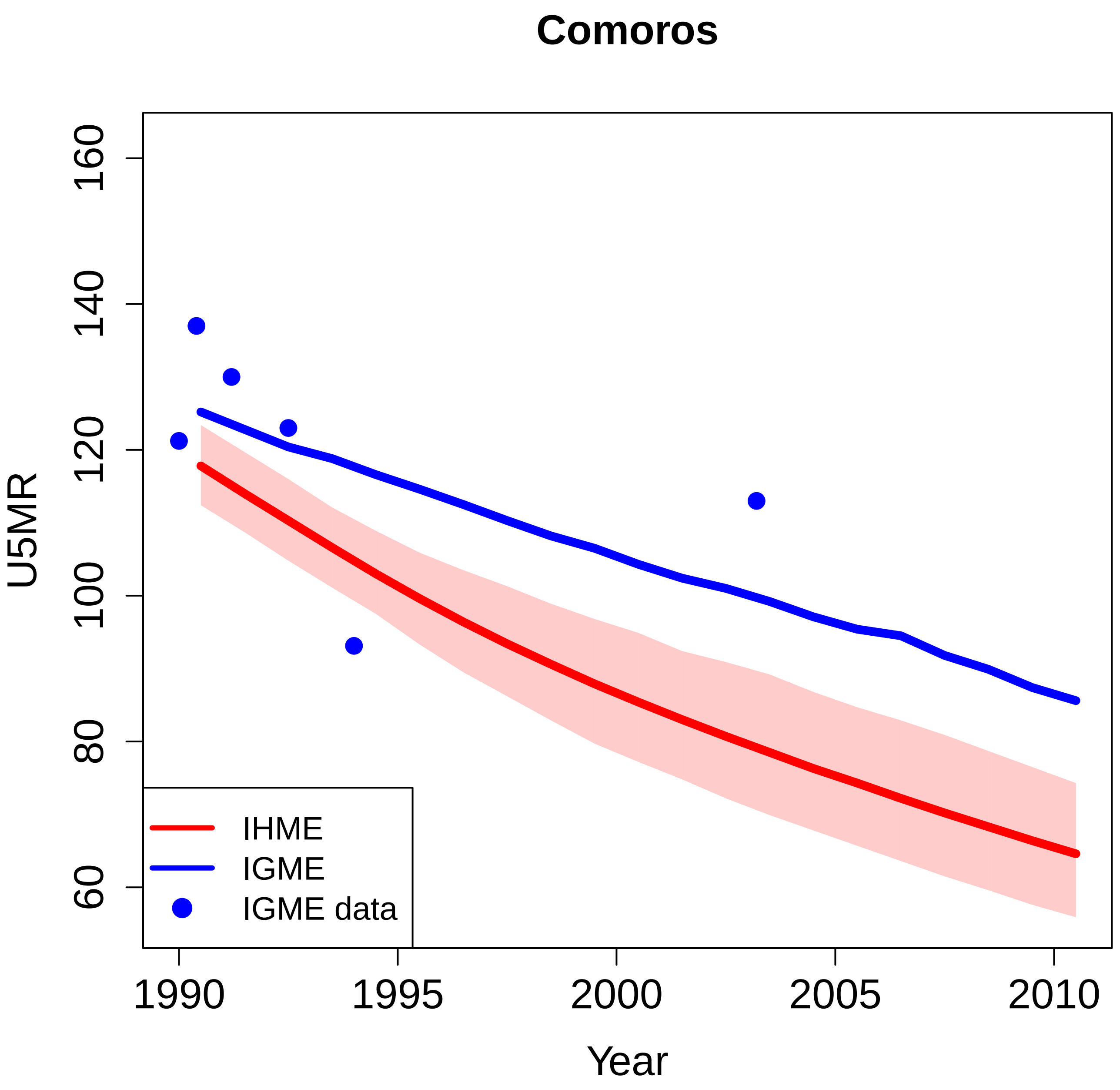
Comoros
U5MR
160
140
120
100
80
60
1990
1995
2000
2005
2010
Year
IHME
IGME
IGME data

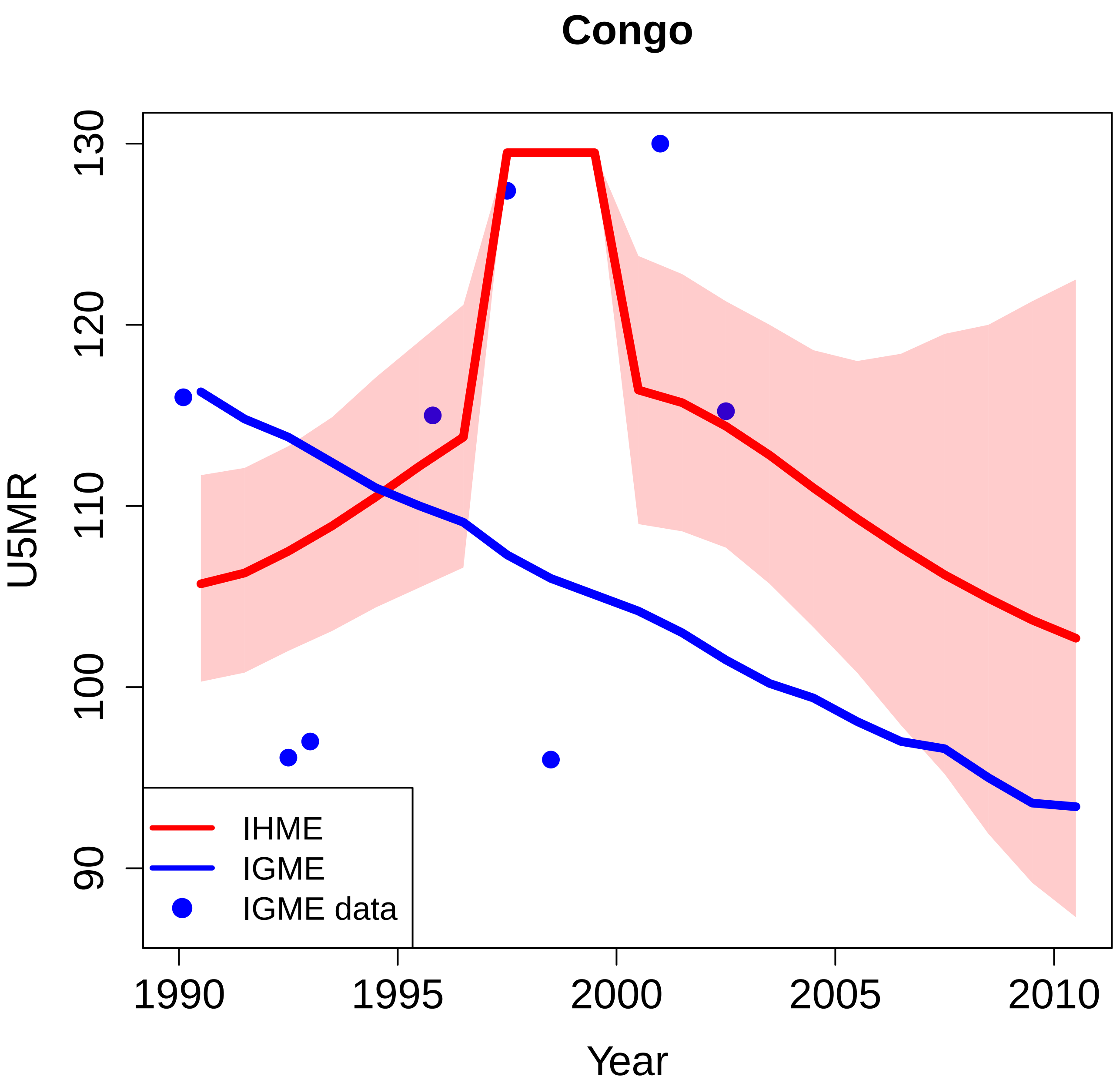
Congo
U5MR
Year
130
120
110
100
90
1990
1995
2000
2005
2010
IHME
IGME
IGME data

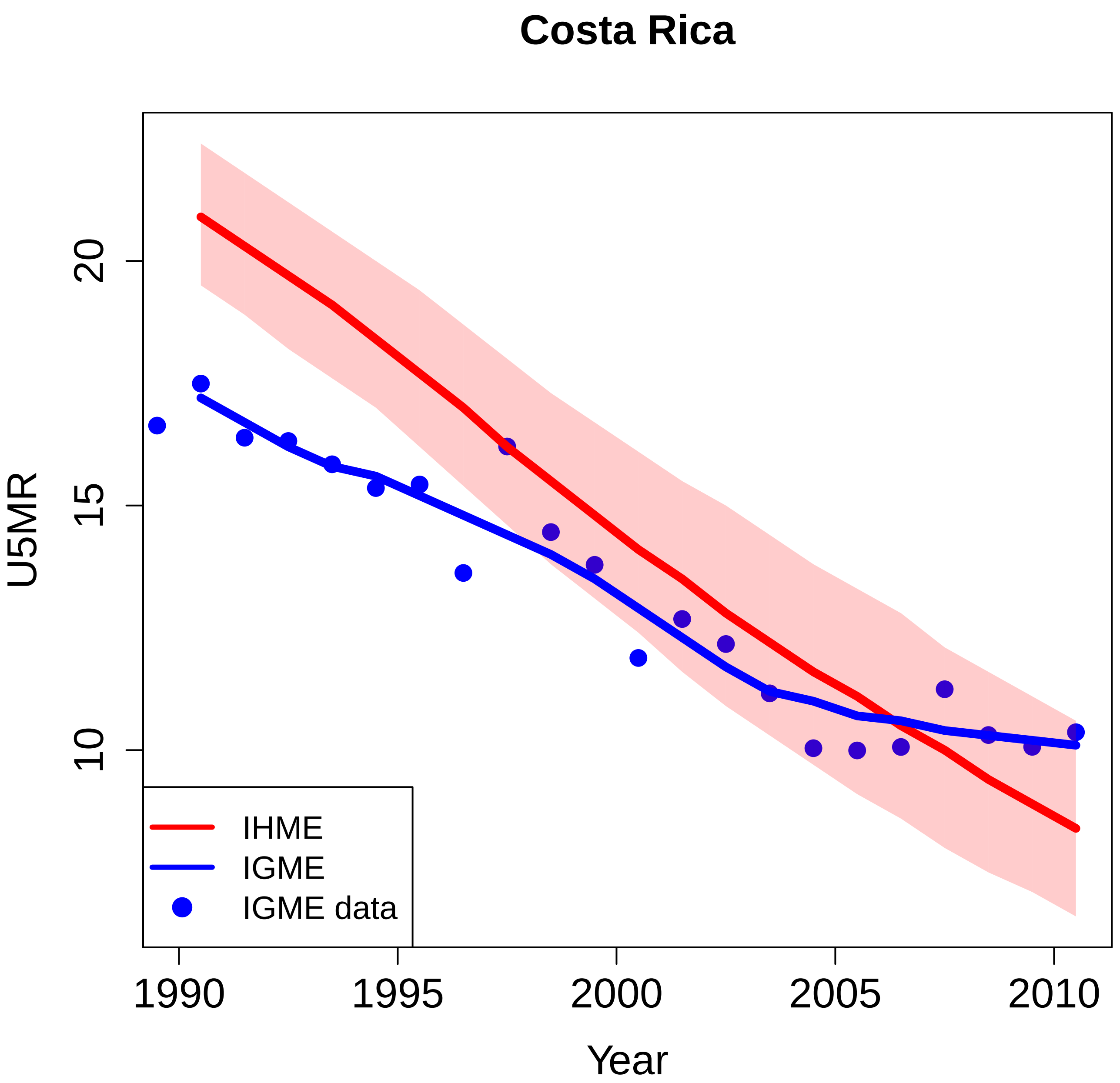

Costa Rica
U5MR
20
15
10
1990
1995
2000
2005
2010
Year
IHME
IGME
IGME data

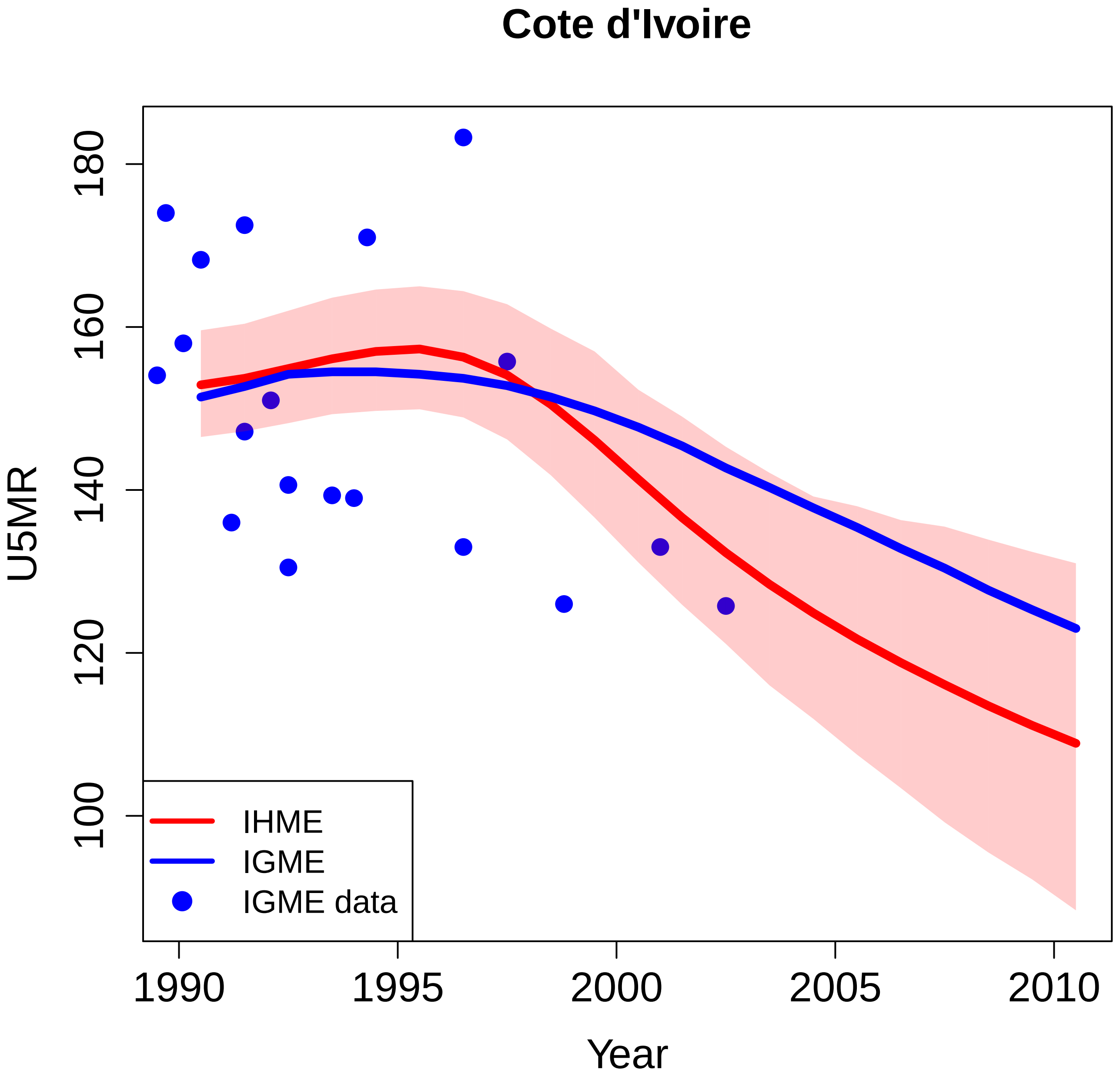

Cote d'Ivoire
U5MR
Year
100
120
140
160
180
1990
1995
2000
2005
2010
IHME
IGME
IGME data

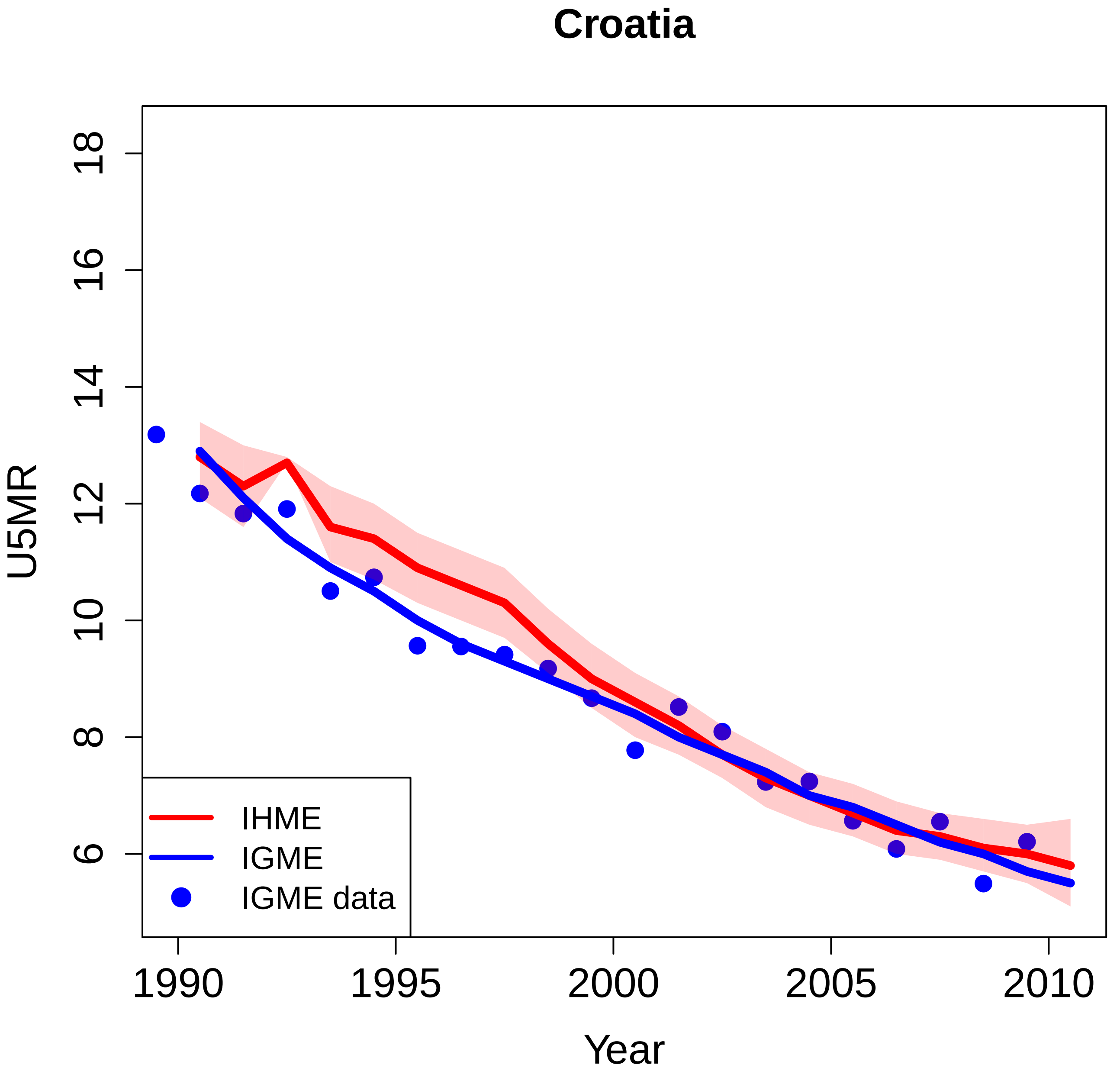

Croatia
U5MR
18
16
14
12
10
8
6
1990
1995
2000
2005
2010
Year
IHME
IGME
IGME data

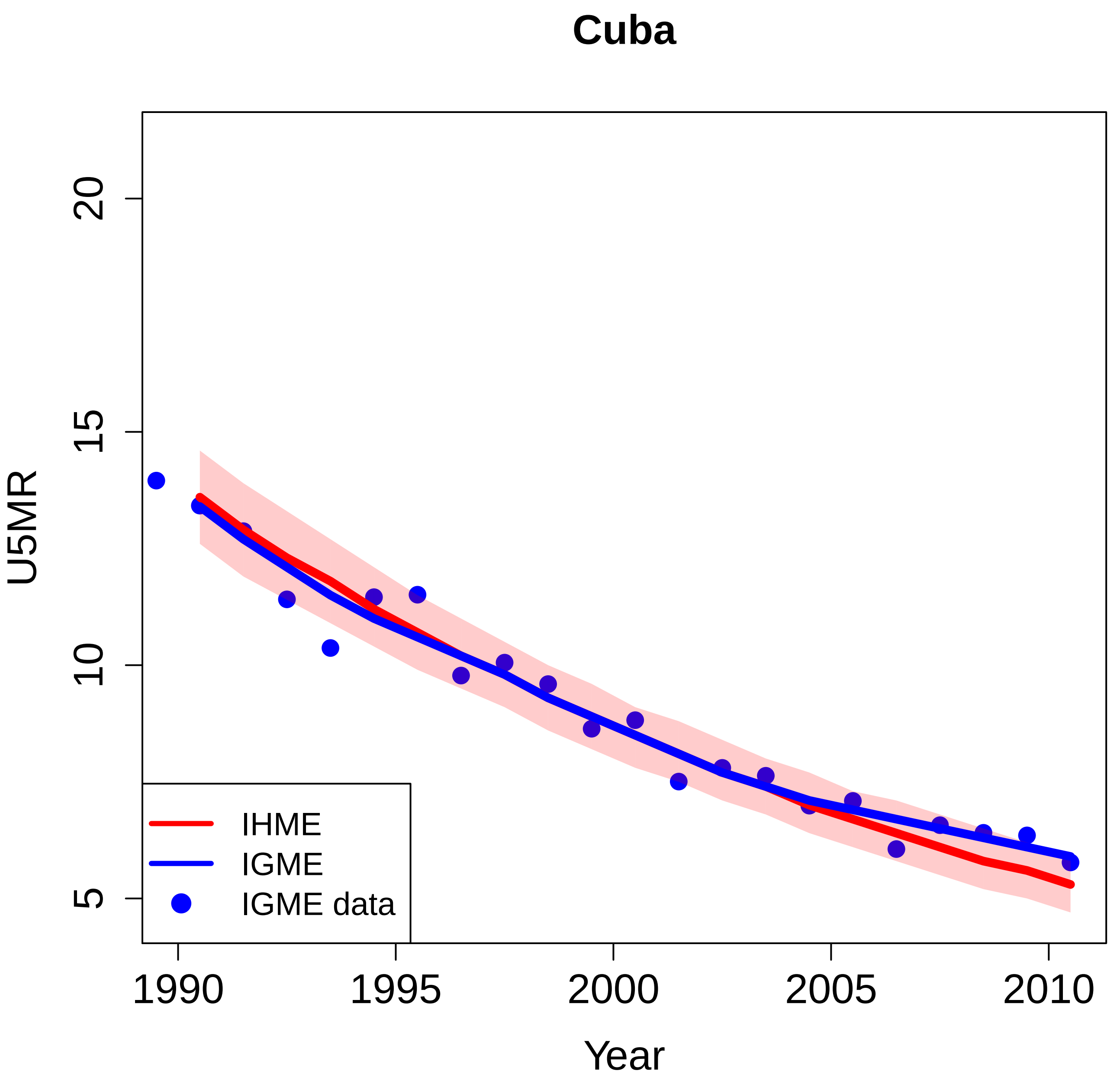
Cuba
U5MR
20
15
10
5
1990
1995
2000
2005
2010
Year
IHME
IGME
IGME data

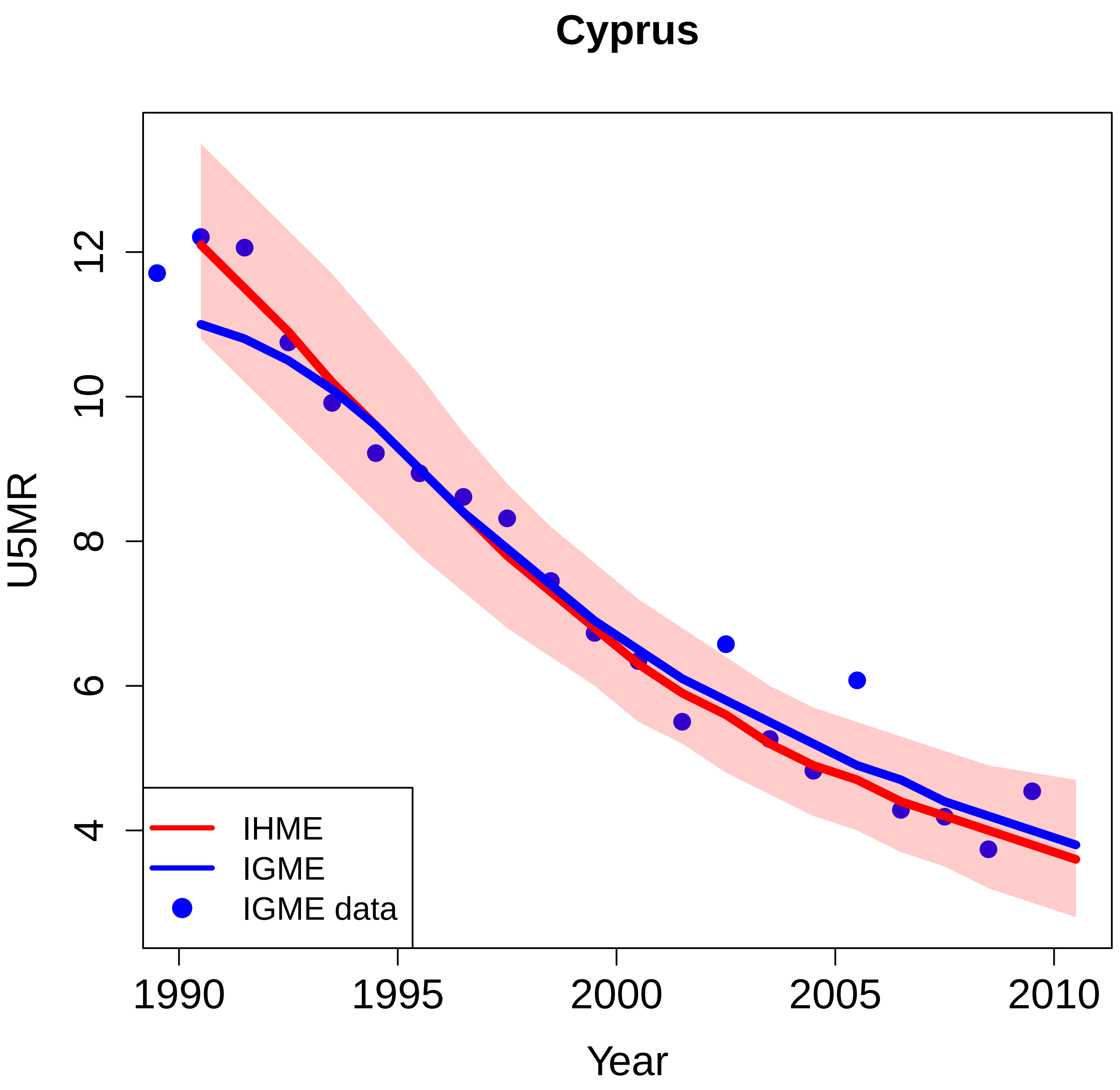
Cyprus
U5MR
12
10
8
6
4
1990
1995
2000
2005
2010
Year
IHME
IGME
IGME data

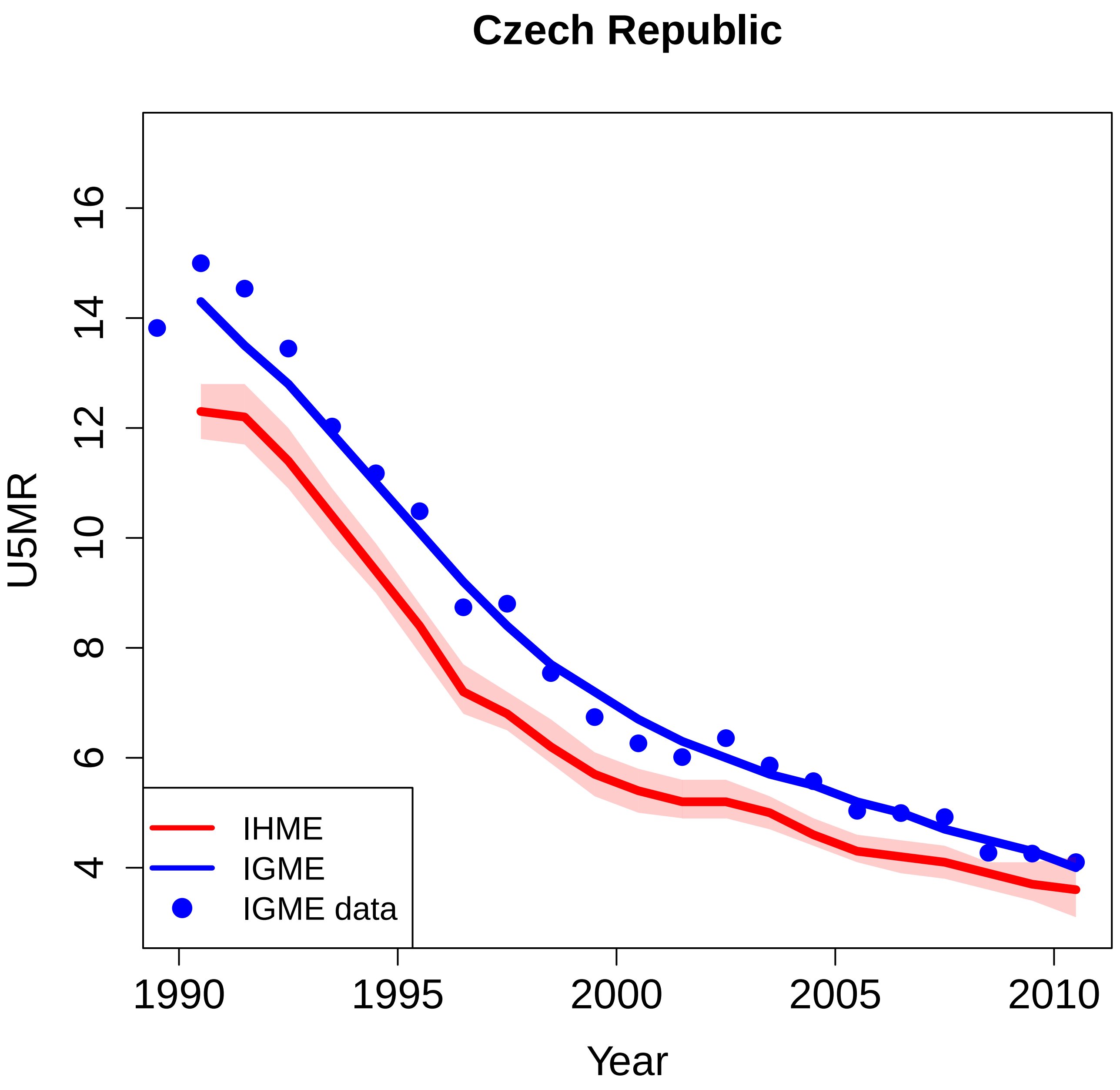
Czech Republic
U5MR
16
14
12
10
8
6
4
1990
1995
2000
2005
2010
Year
IHME
IGME
IGME data

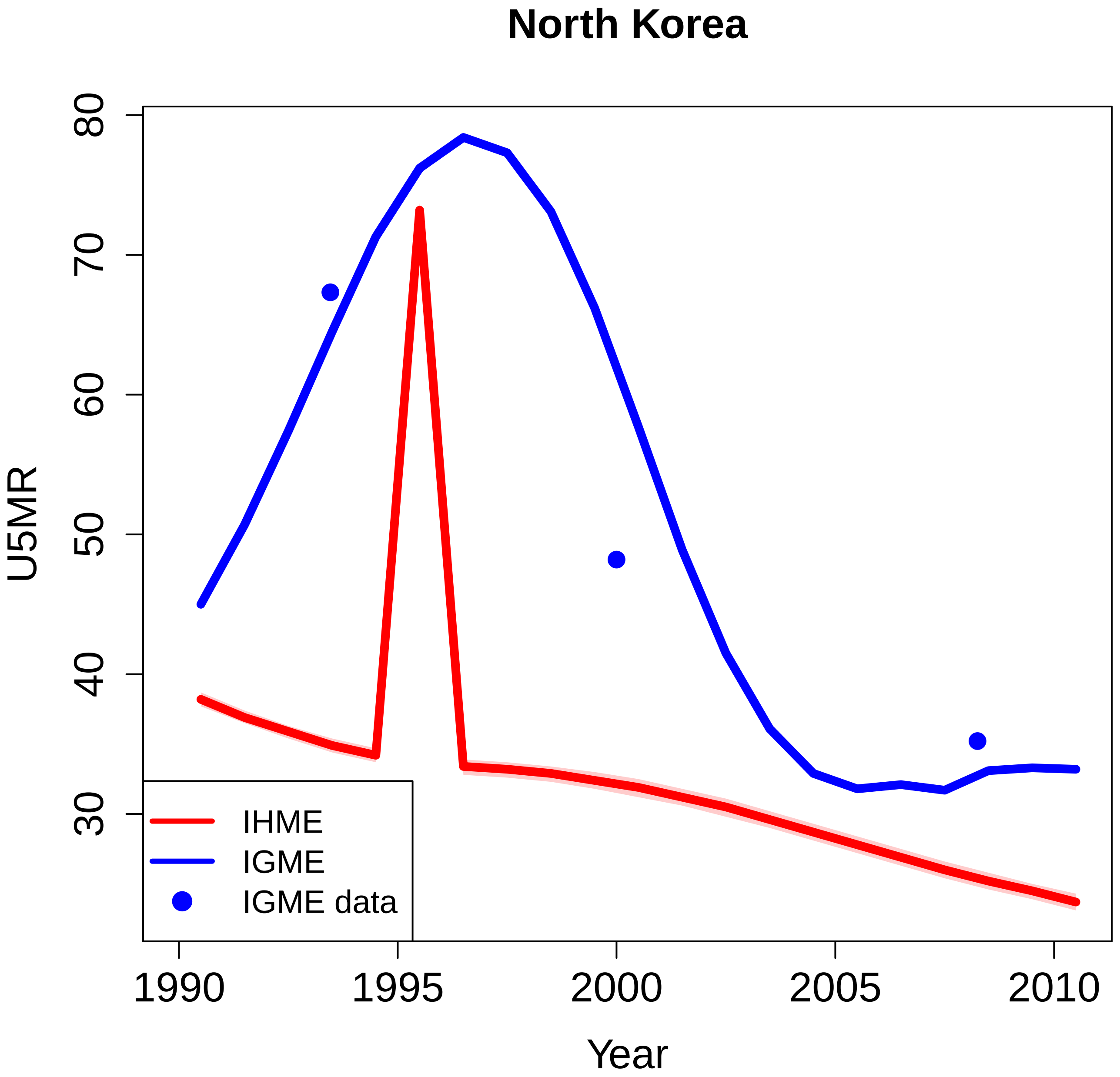

North Korea
U5MR
80
70
60
50
40
30
1990
1995
2000
2005
2010
Year
IHME
IGME
IGME data

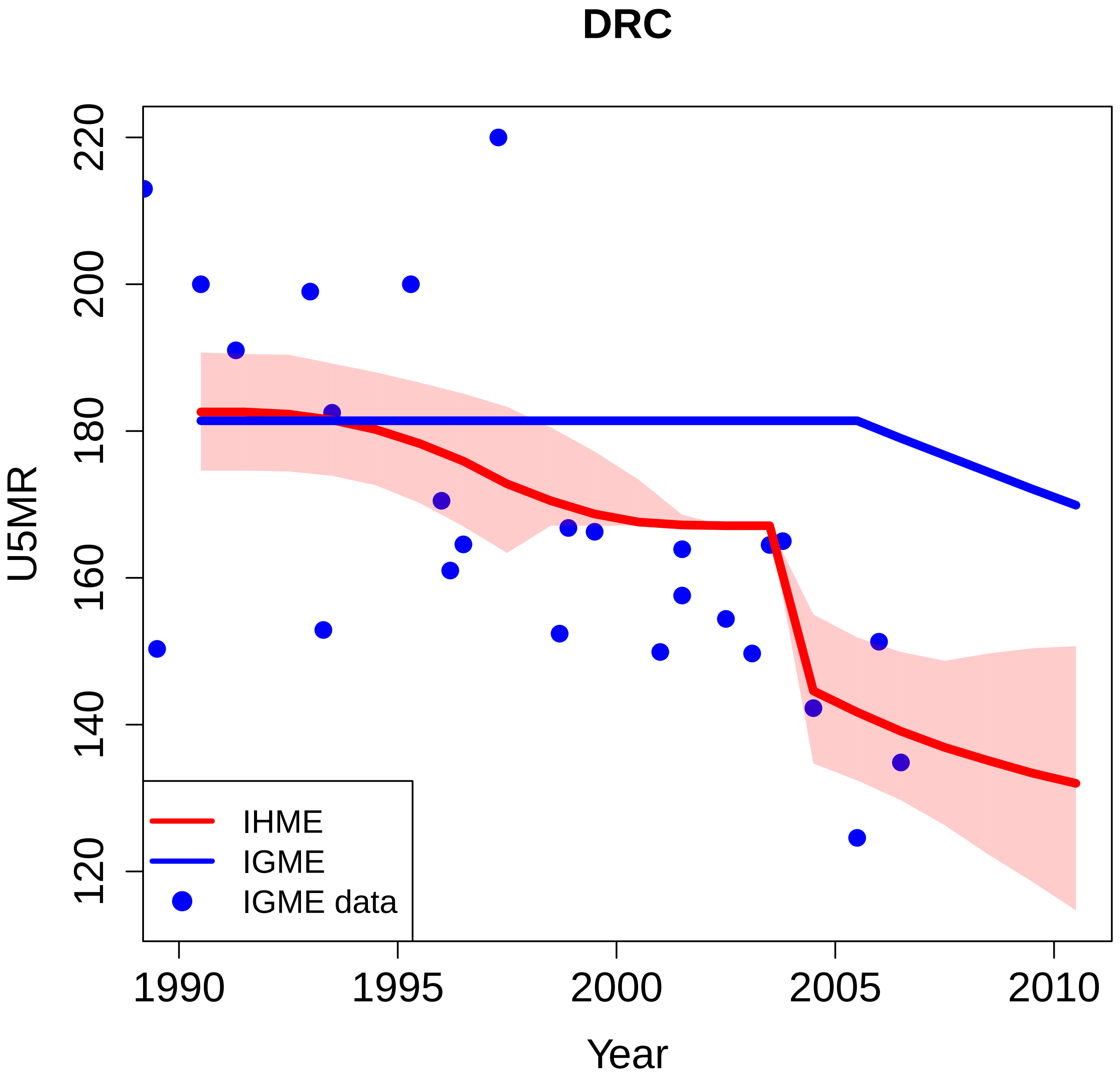

DRC
U5MR
220
200
180
160
140
120
Year
1990
1995
2000
2005
2010
IHME
IGME
IGME data

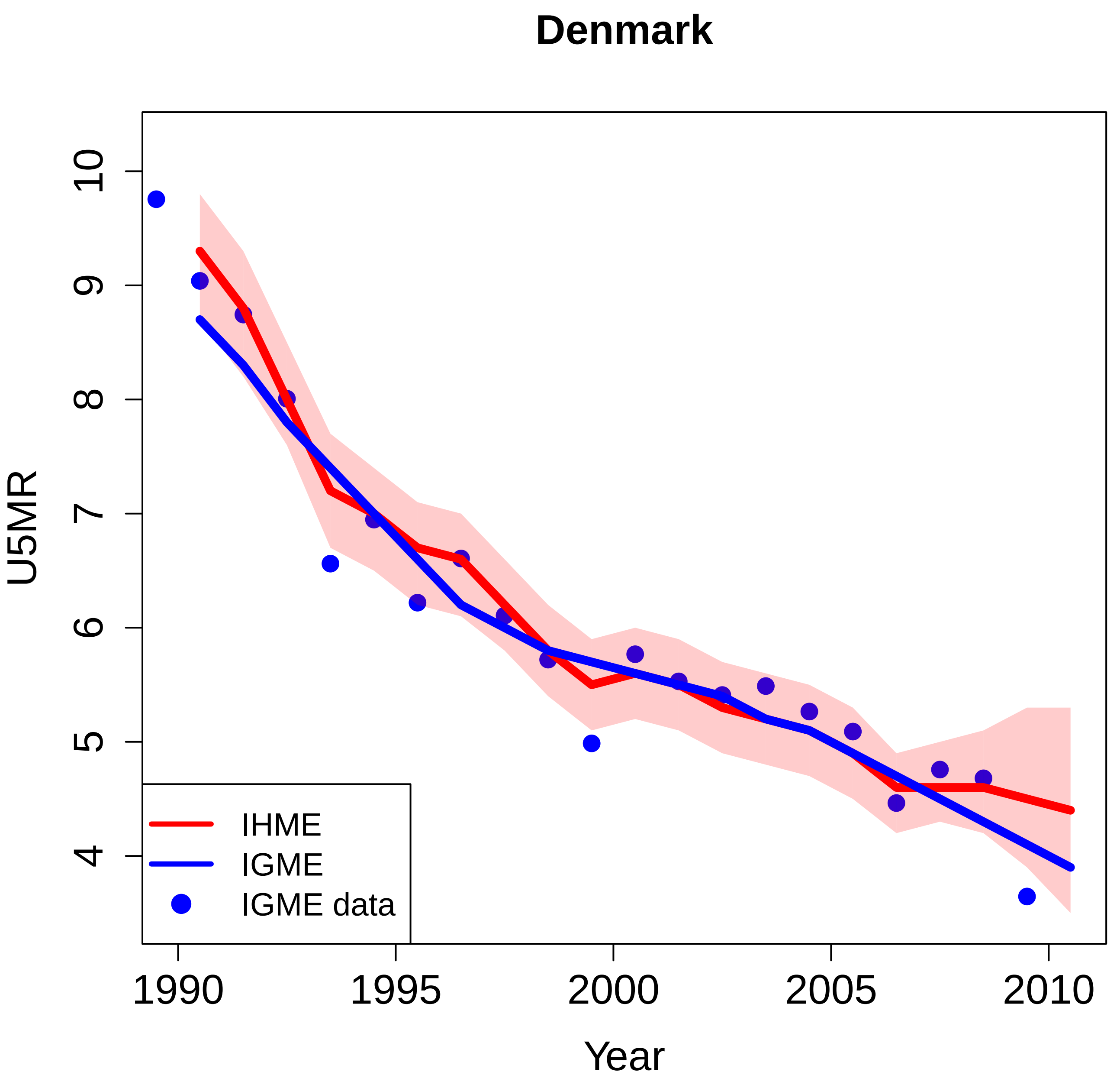

Denmark
U5MR
10
9
8
7
6
5
4
1990
1995
2000
2005
2010
Year
IHME
IGME
IGME data

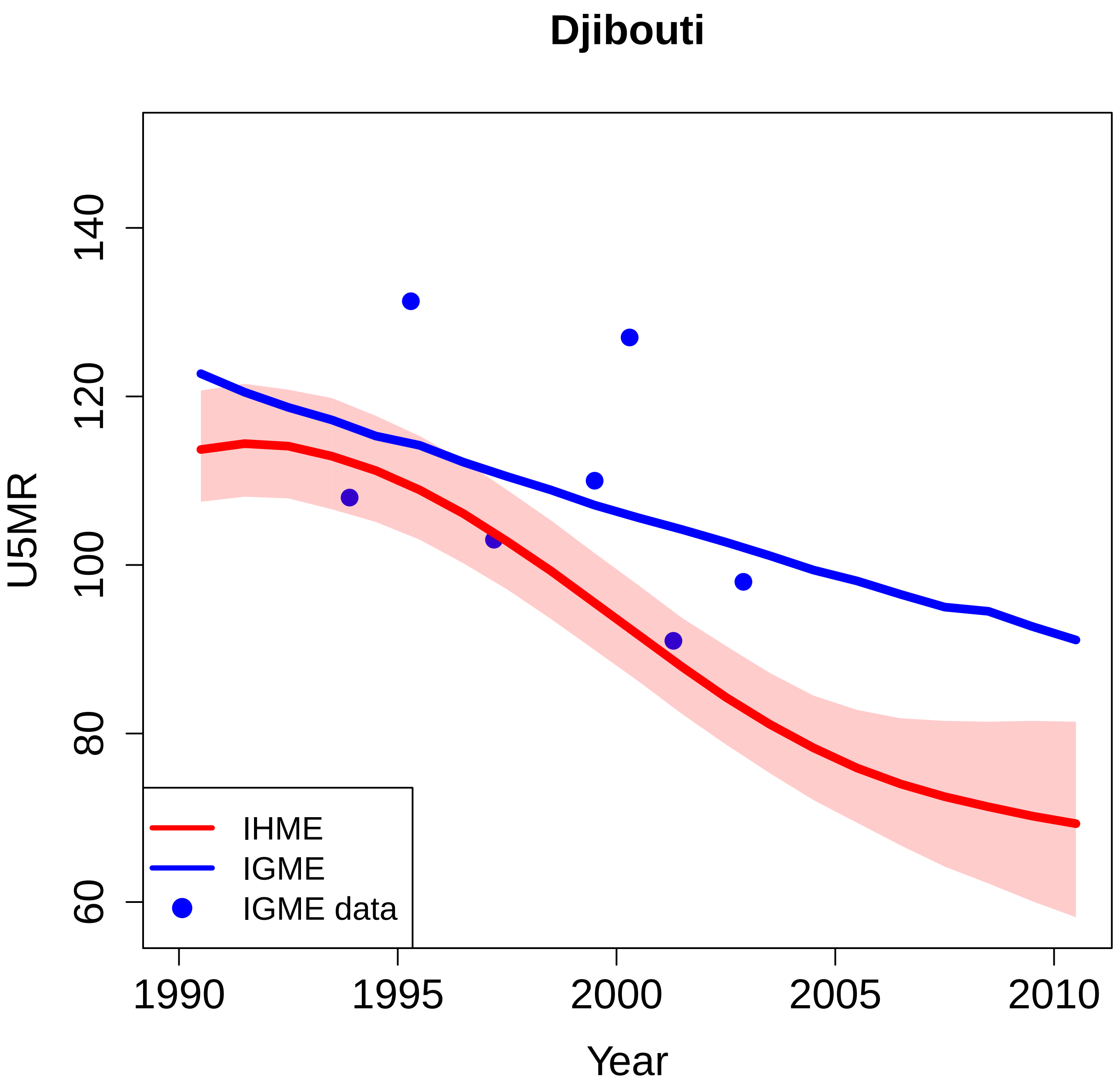
Djibouti
U5MR
Year
60
80
100
120
140
1990
1995
2000
2005
2010
IHME
IGME
IGME data

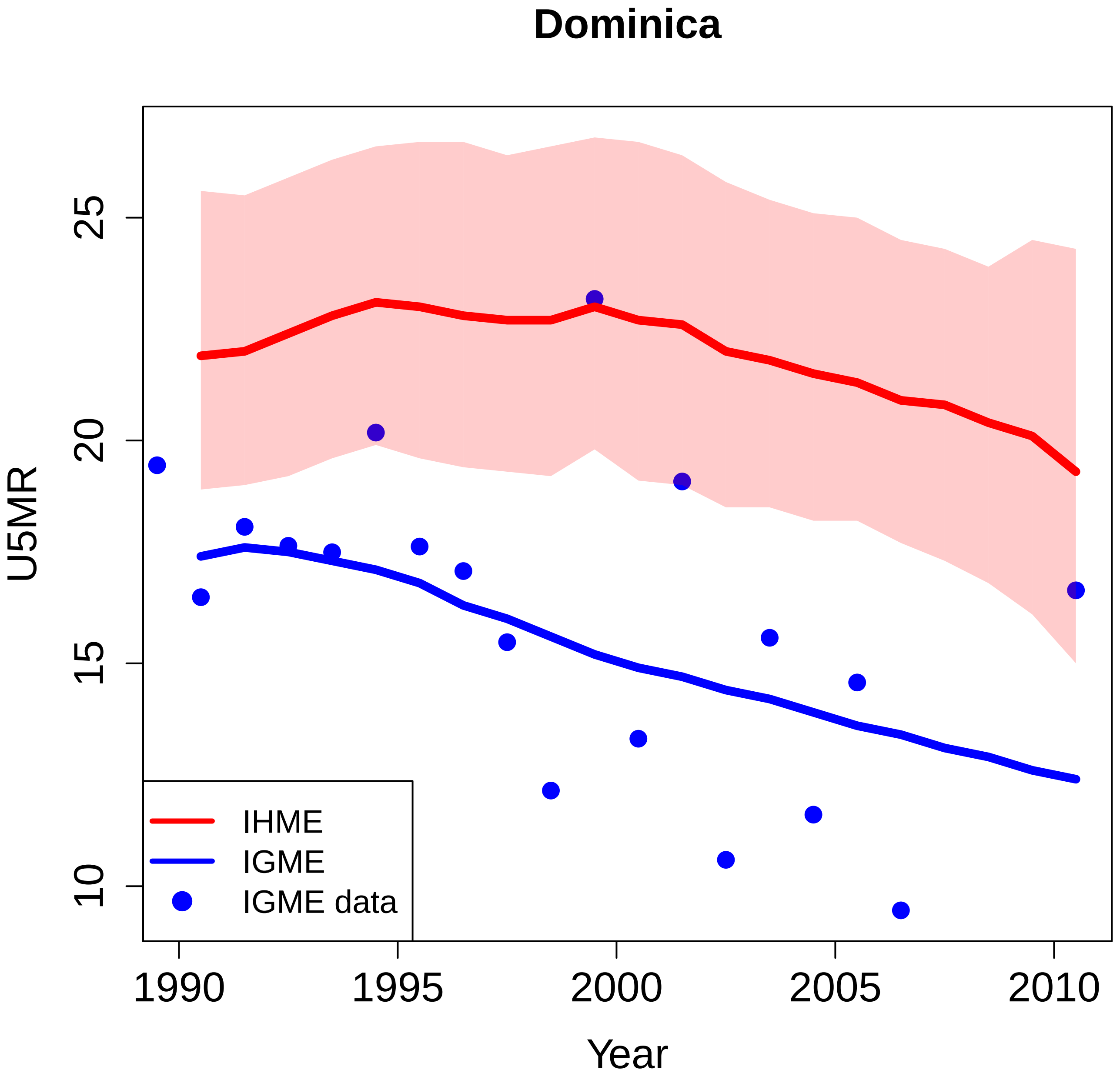
Dominica
U5MR
25
20
15
10
1990
1995
2000
2005
2010
Year
IHME
IGME
IGME data

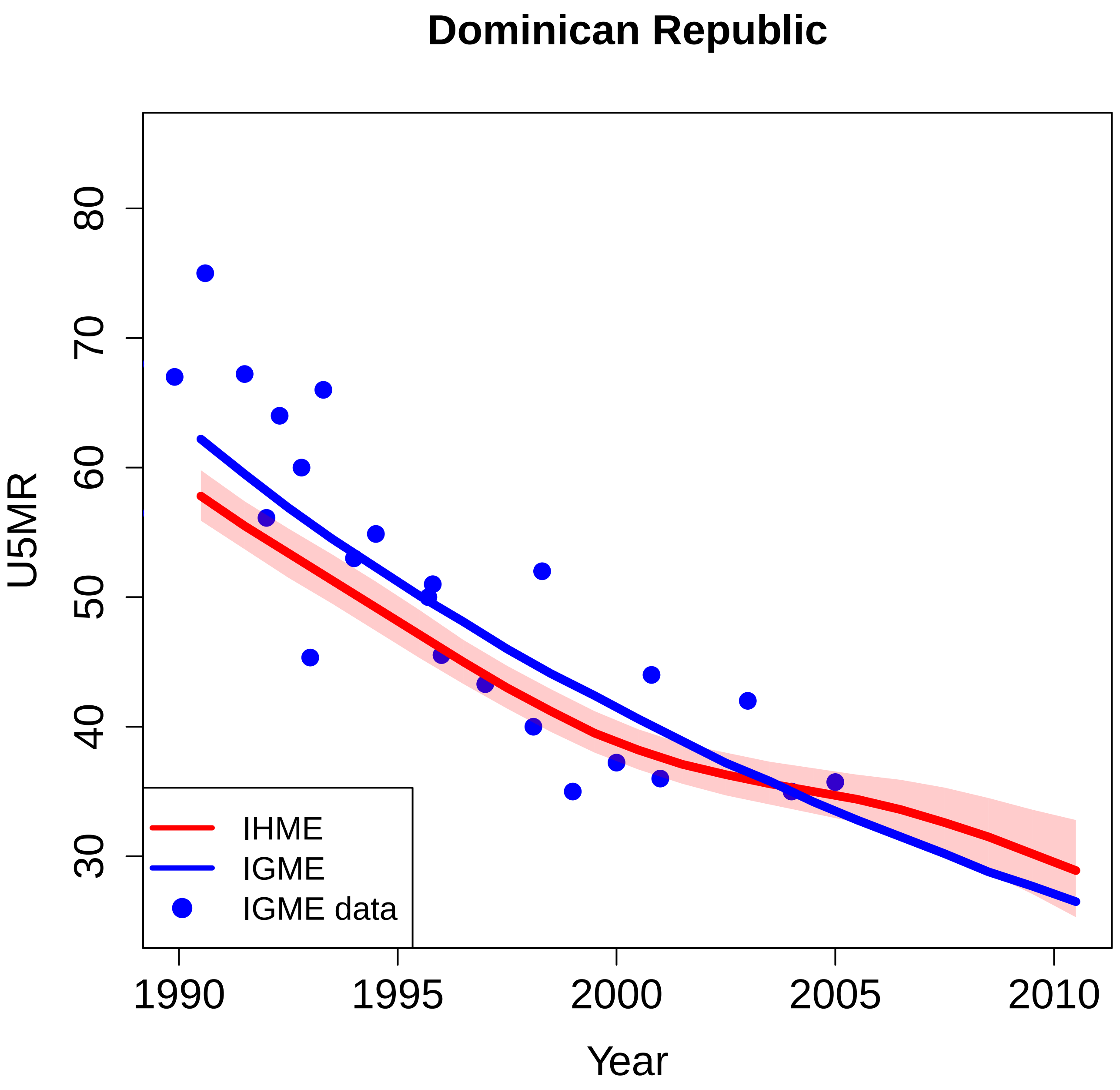

Dominican Republic
U5MR
80
70
60
50
40
30
1990
1995
2000
2005
2010
Year
IHME
IGME
IGME data

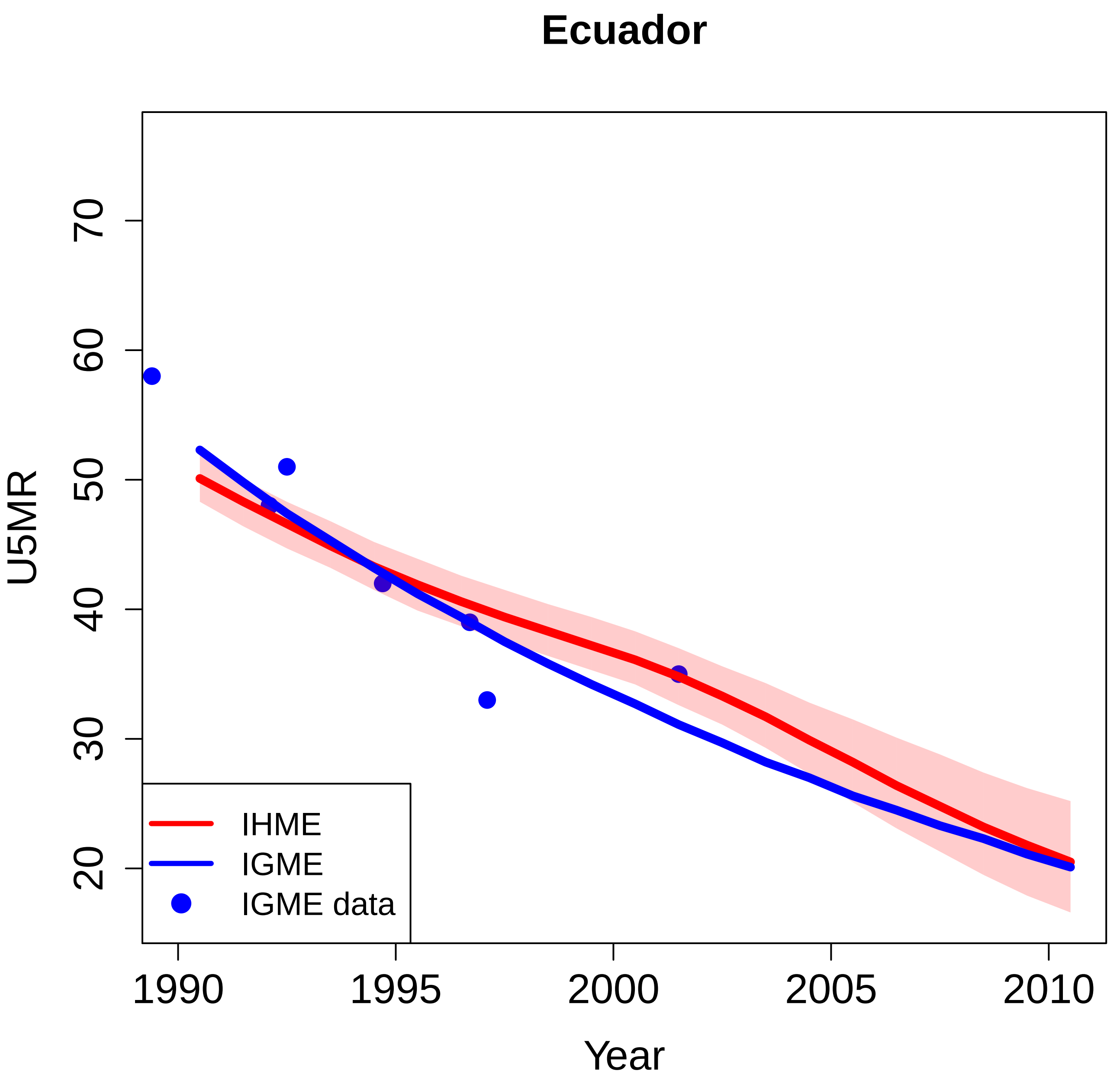
Ecuador
U5MR
Year
70
60
50
40
30
20
1990
1995
2000
2005
2010
IHME
IGME
IGME data

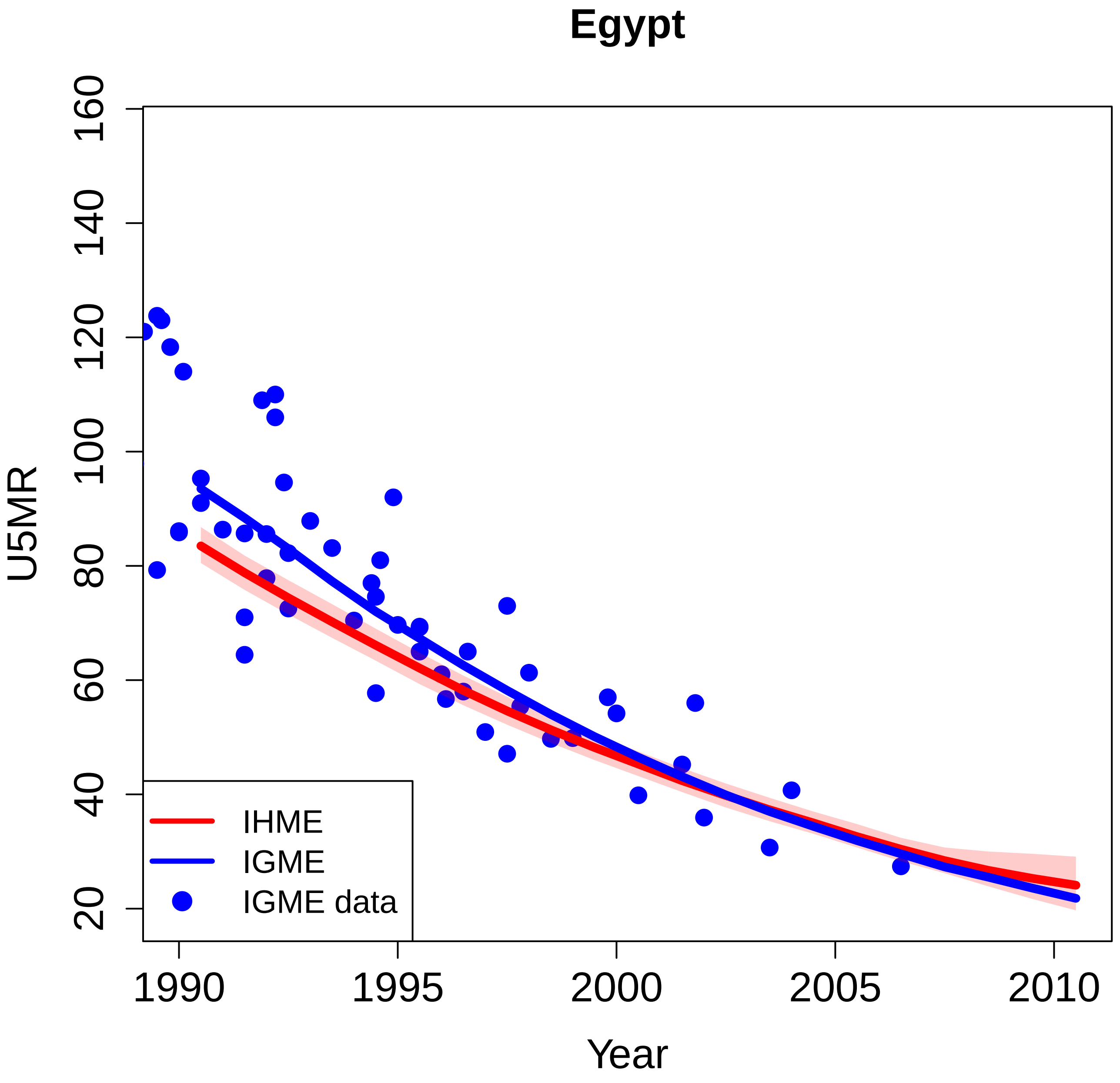

Egypt
U5MR
Year
20
40
60
80
100
120
140
160
1990
1995
2000
2005
2010
IHME
IGME
IGME data

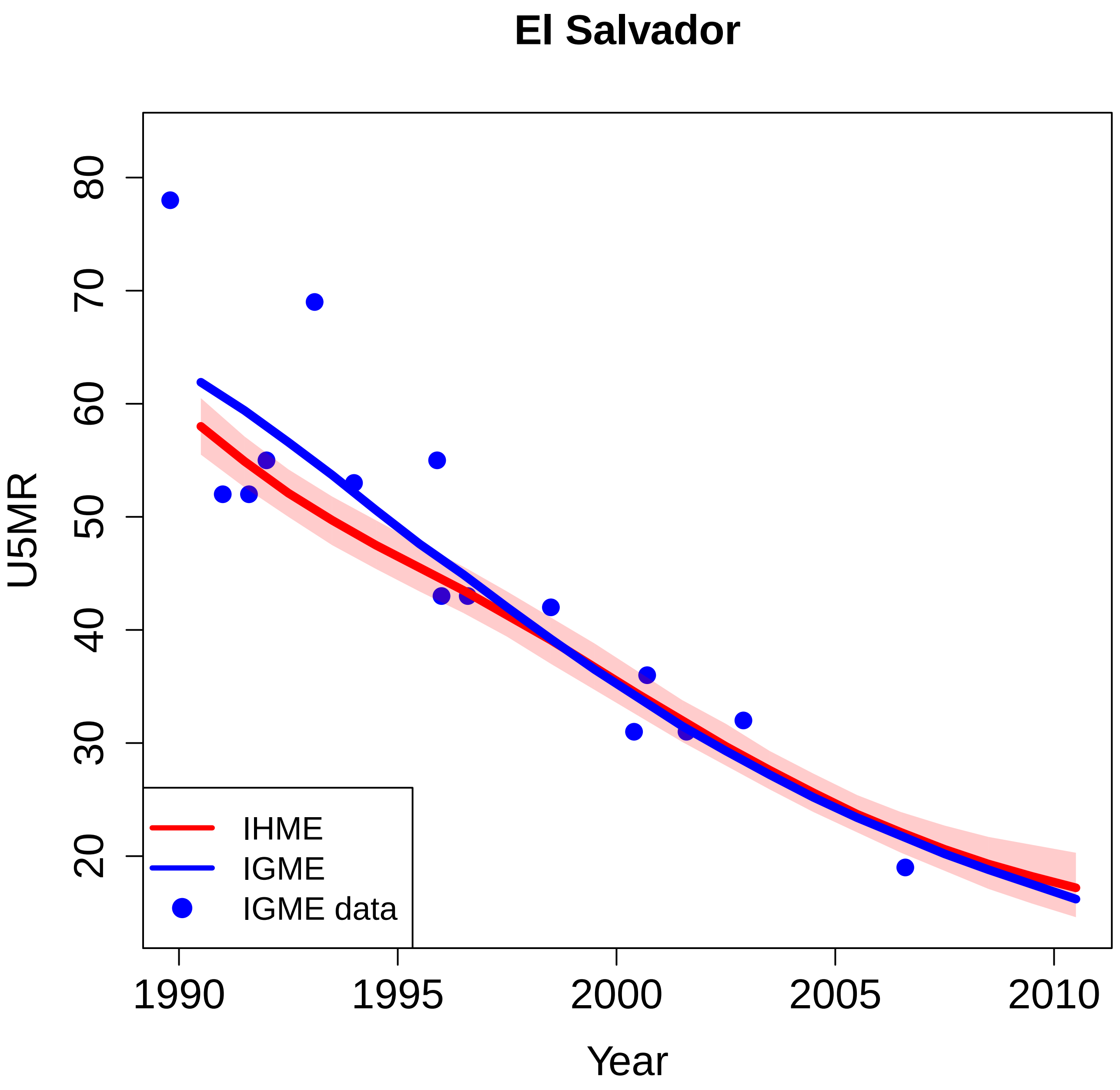
El Salvador
U5MR
80
70
60
50
40
30
20
1990
1995
2000
2005
2010
Year
IHME
IGME
IGME data

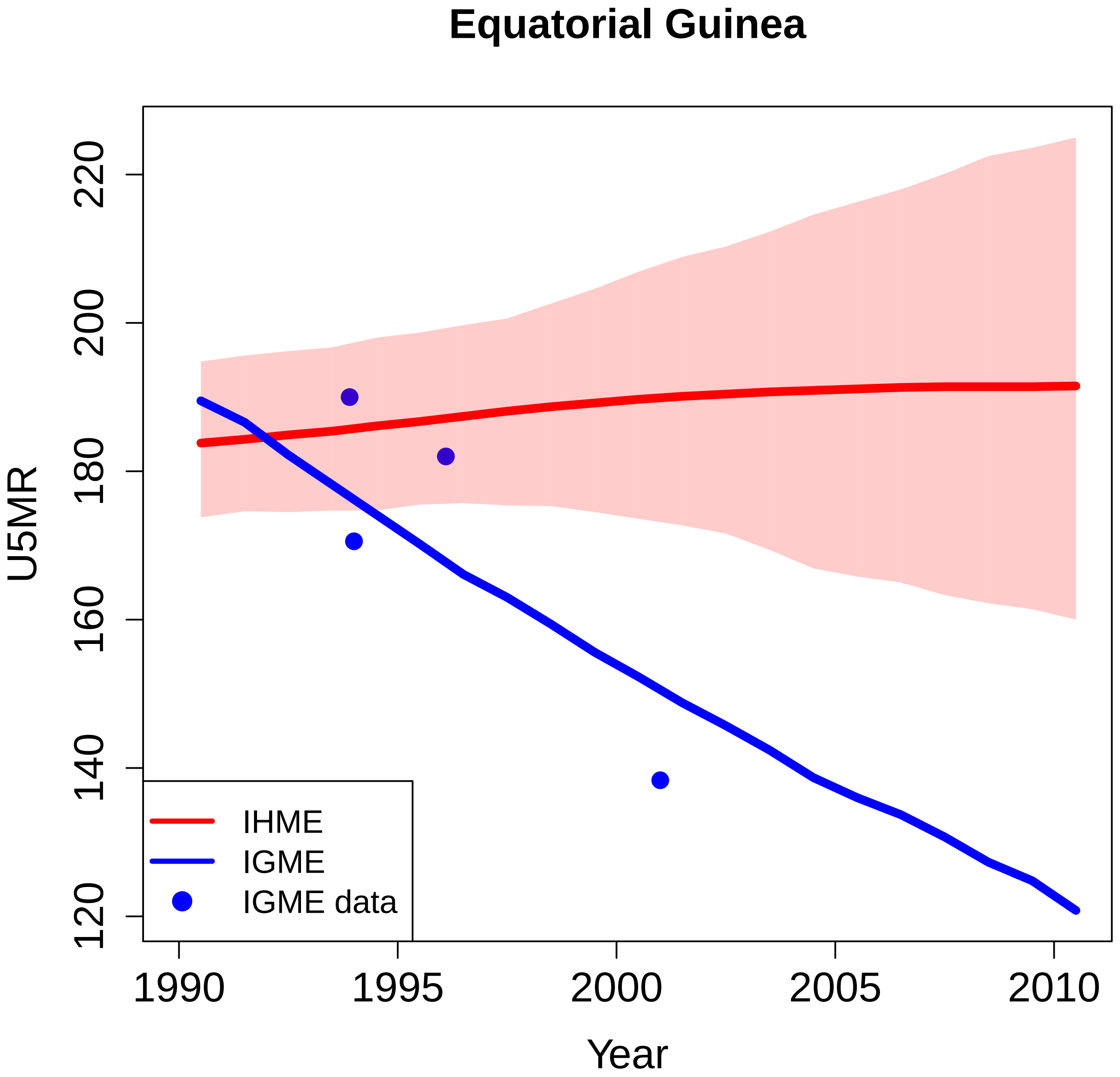

Equatorial Guinea
U5MR
Year
120
140
160
180
200
220
1990
1995
2000
2005
2010
IHME
IGME
IGME data

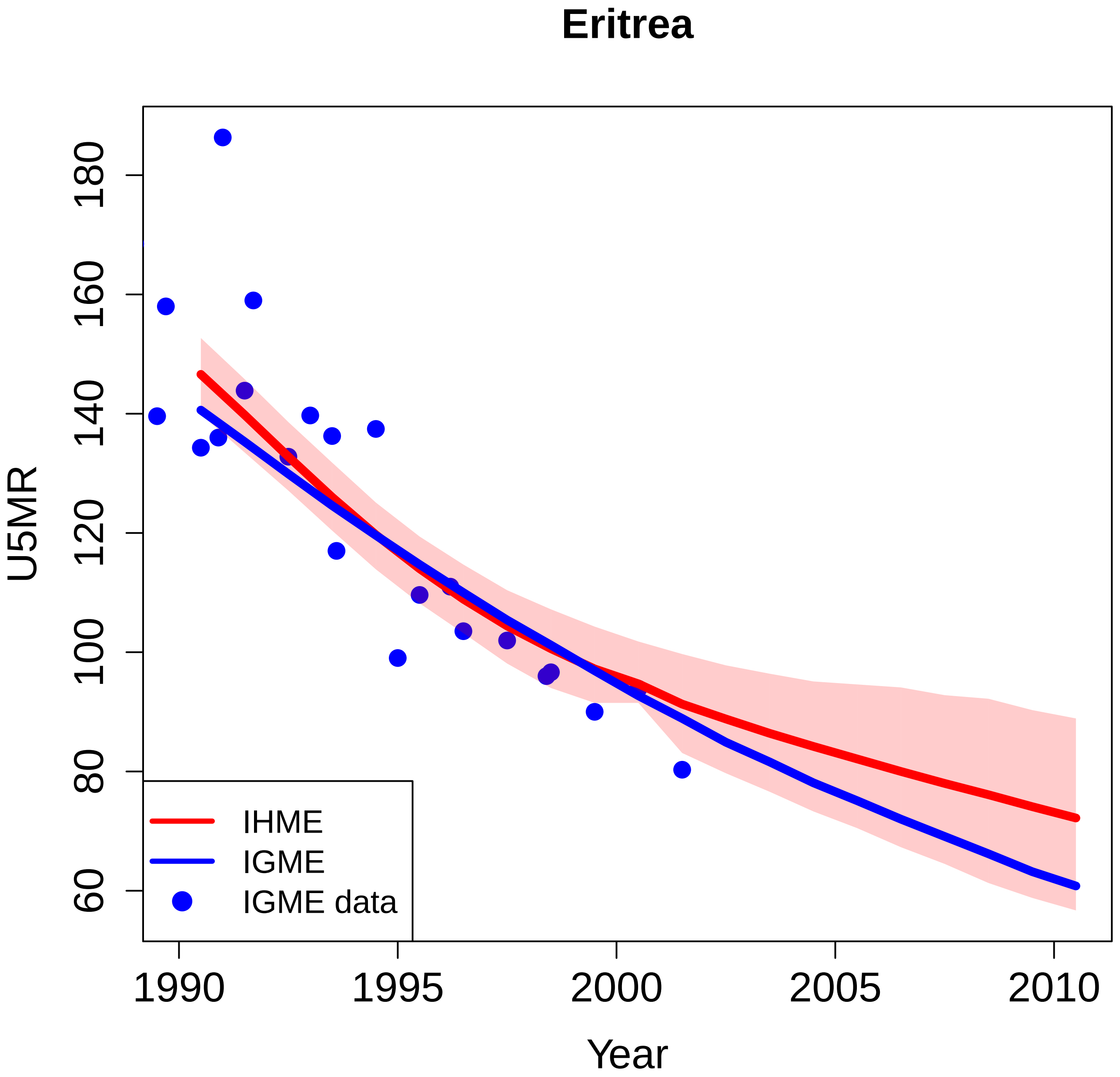
Eritrea
U5MR
180
160
140
120
100
80
60
1990
1995
2000
2005
2010
Year
IHME
IGME
IGME data

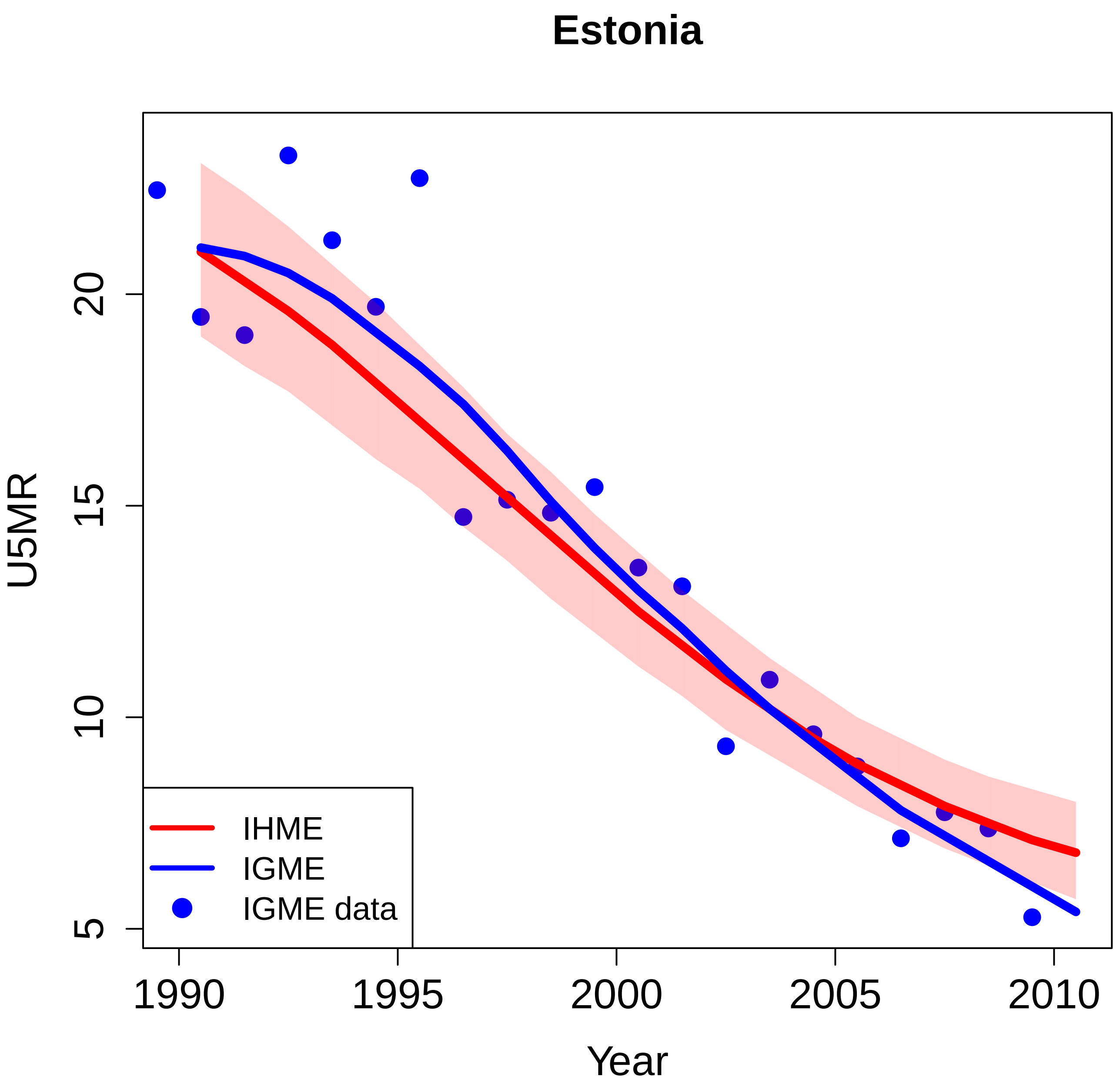

Estonia
U5MR
Year
5
10
15
20
1990
1995
2000
2005
2010
IHME
IGME
IGME data

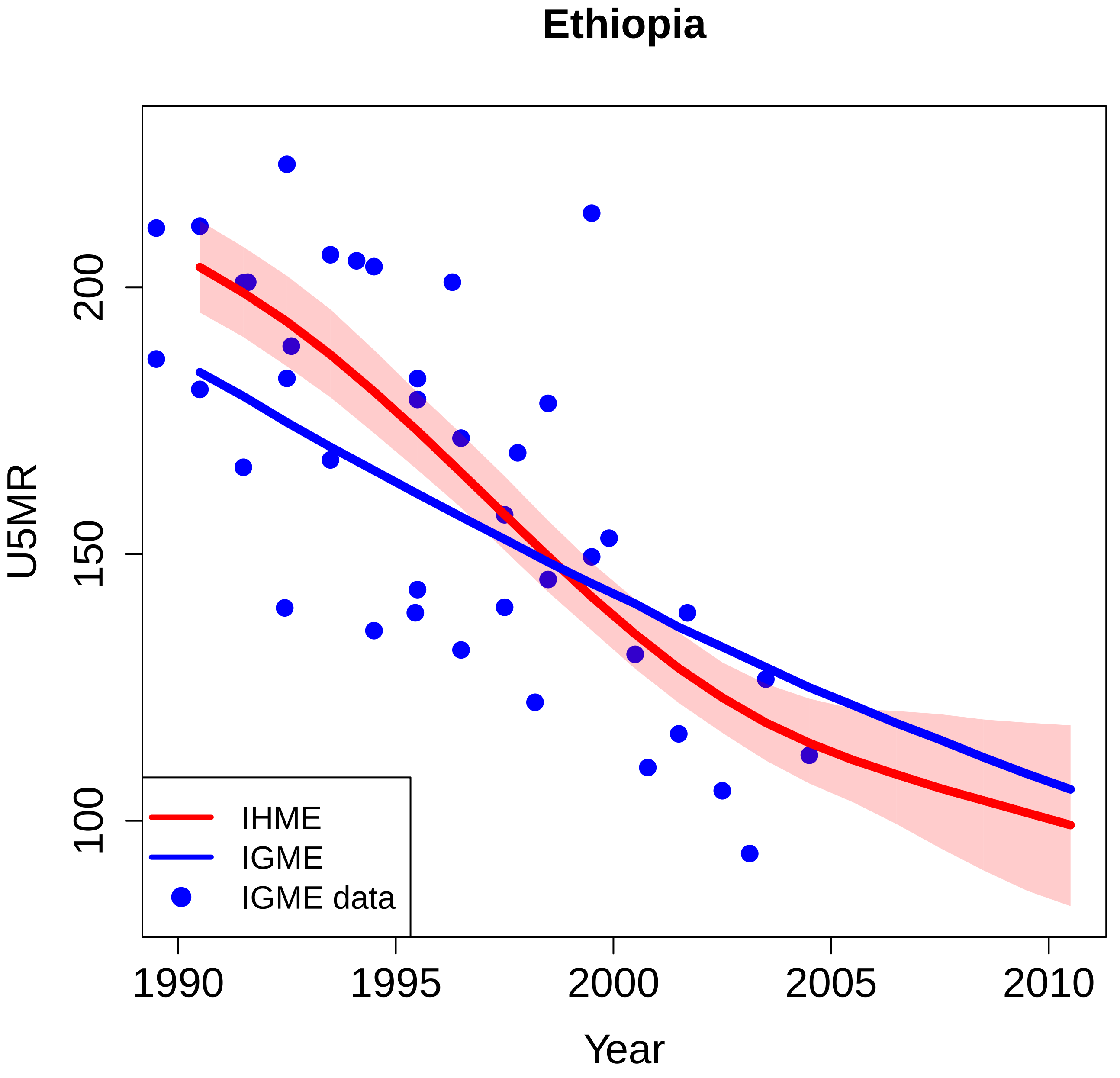
Ethiopia
U5MR
200
150
100
1990
1995
2000
2005
2010
Year
IHME
IGME
IGME data

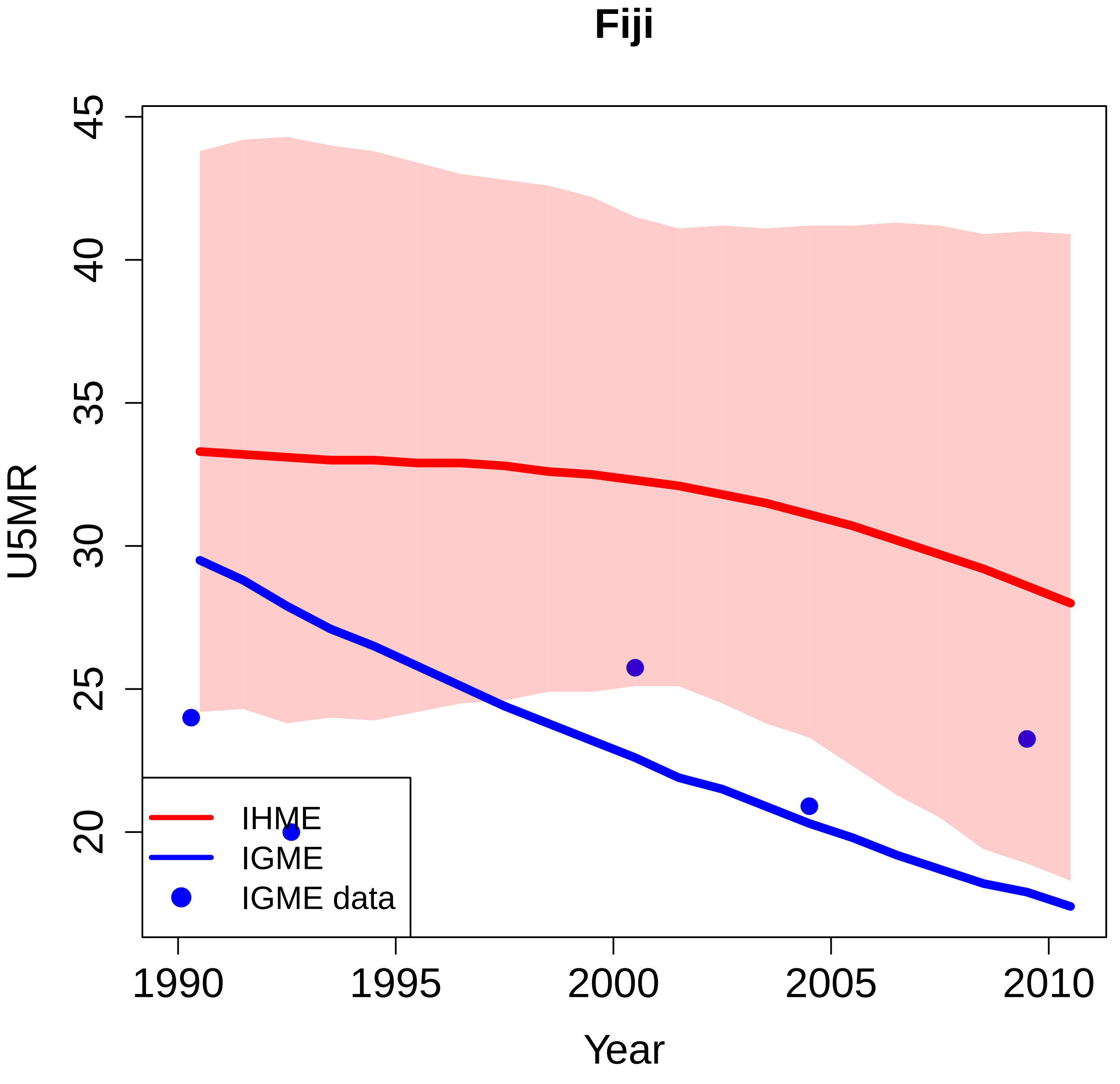
Fiji
U5MR
45
40
35
30
25
20
1990
1995
2000
2005
2010
Year
IHME
IGME
IGME data

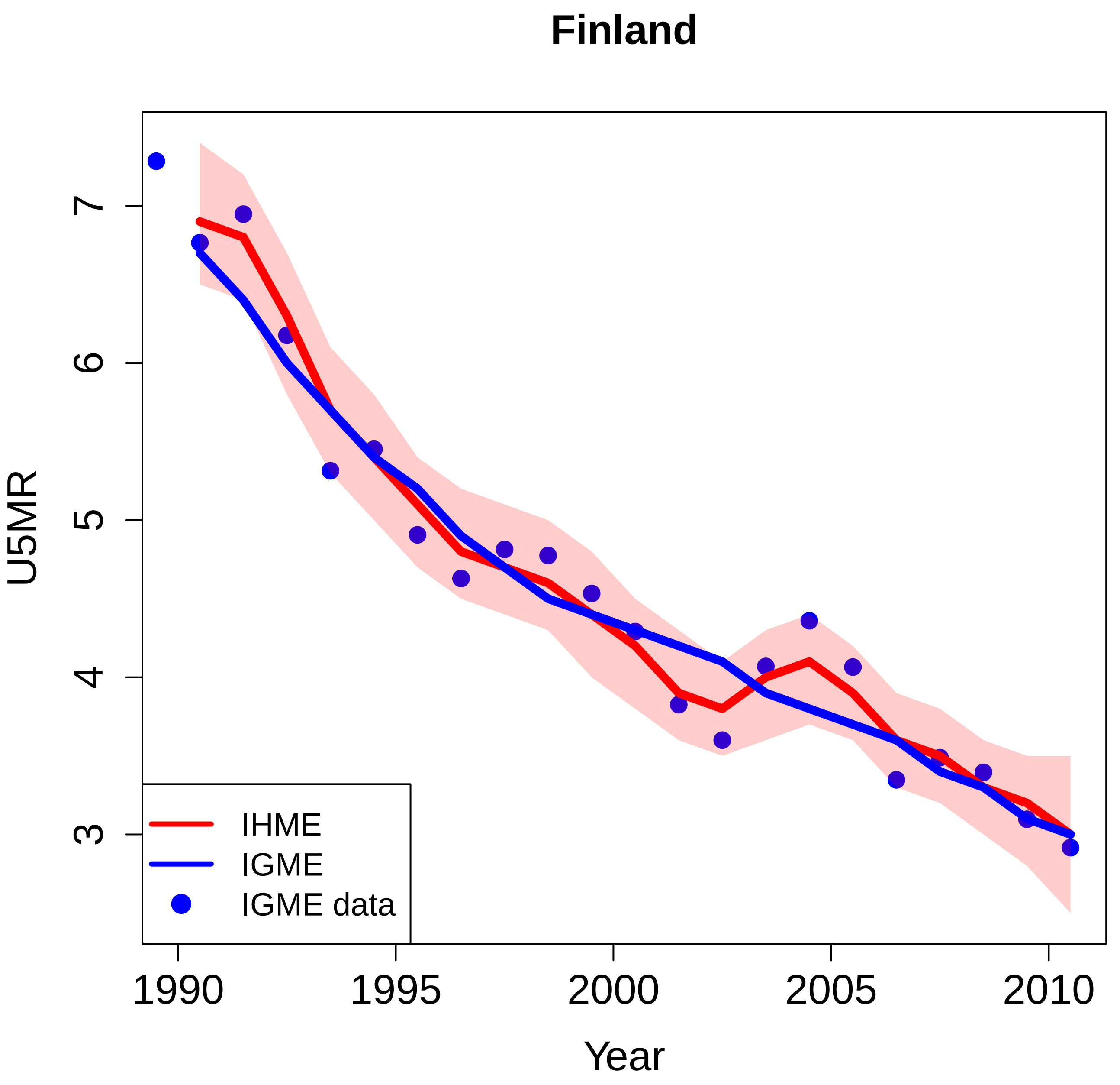

Finland
U5MR
7
6
5
4
3
1990
1995
2000
2005
2010
Year
IHME
IGME
IGME data

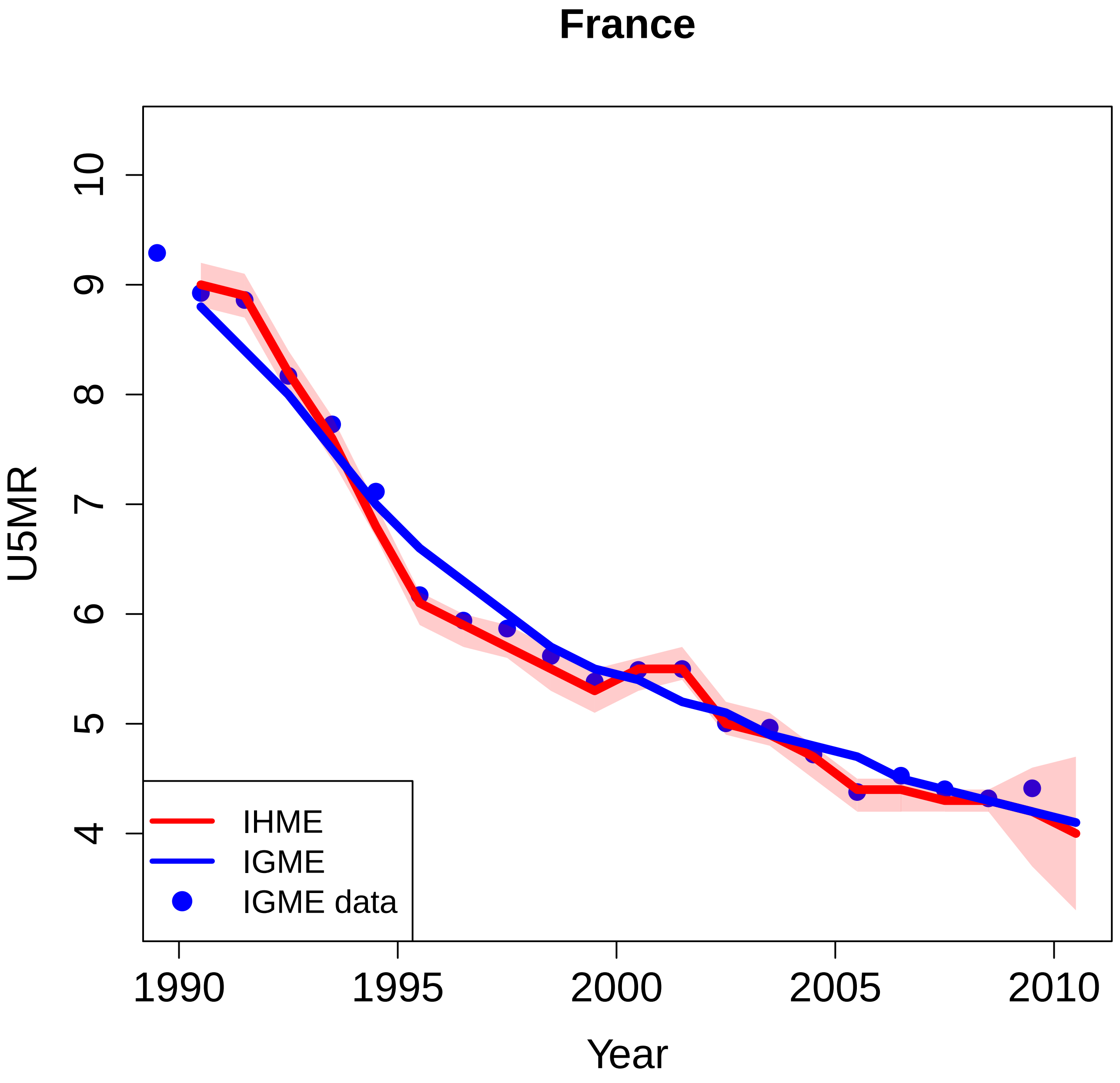
France
U5MR
Year
10
9
8
7
6
5
4
1990
1995
2000
2005
2010
IHME
IGME
IGME data

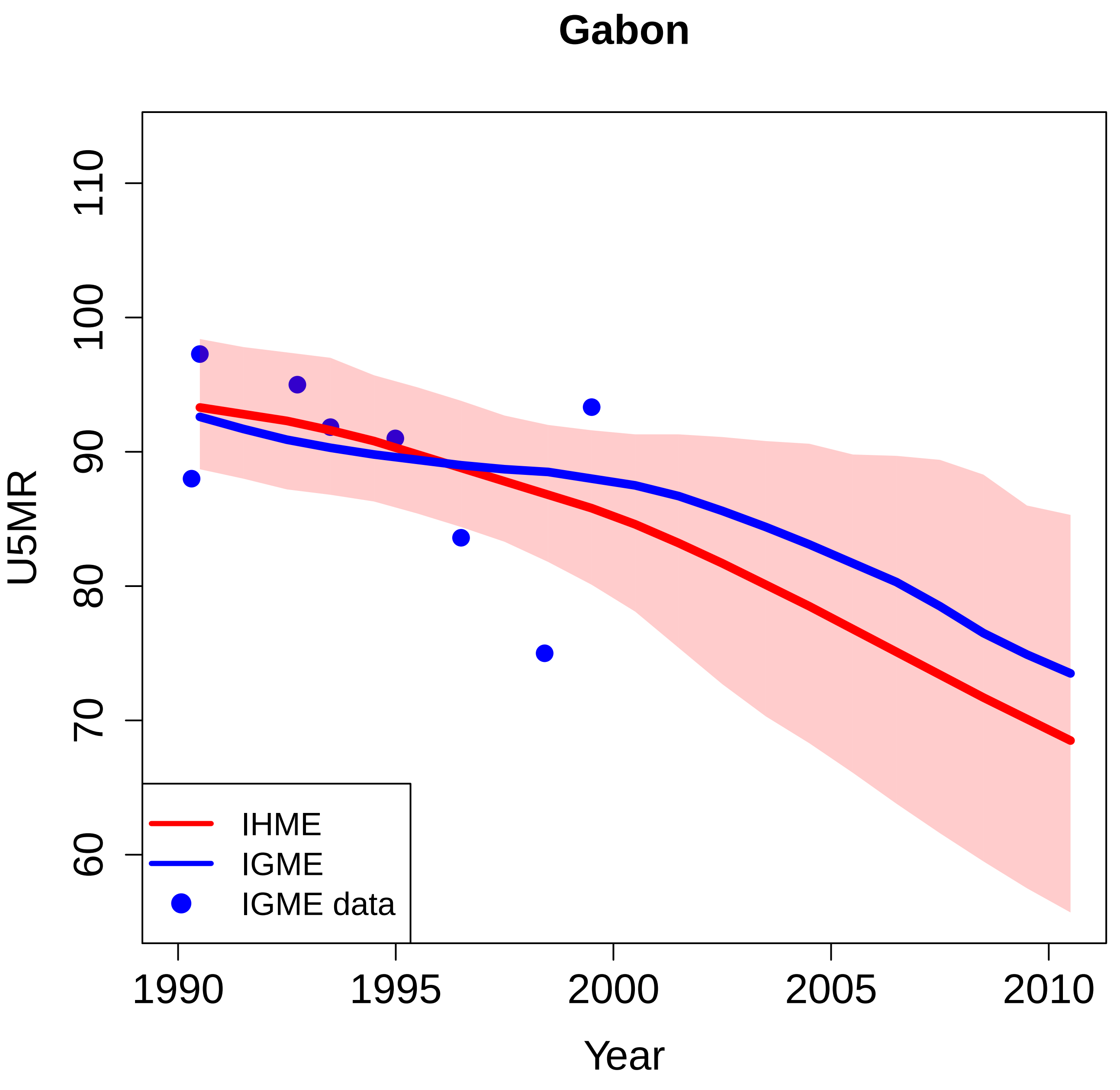
Gabon
U5MR
110
100
90
80
70
60
1990
1995
2000
2005
2010
Year
IHME
IGME
IGME data

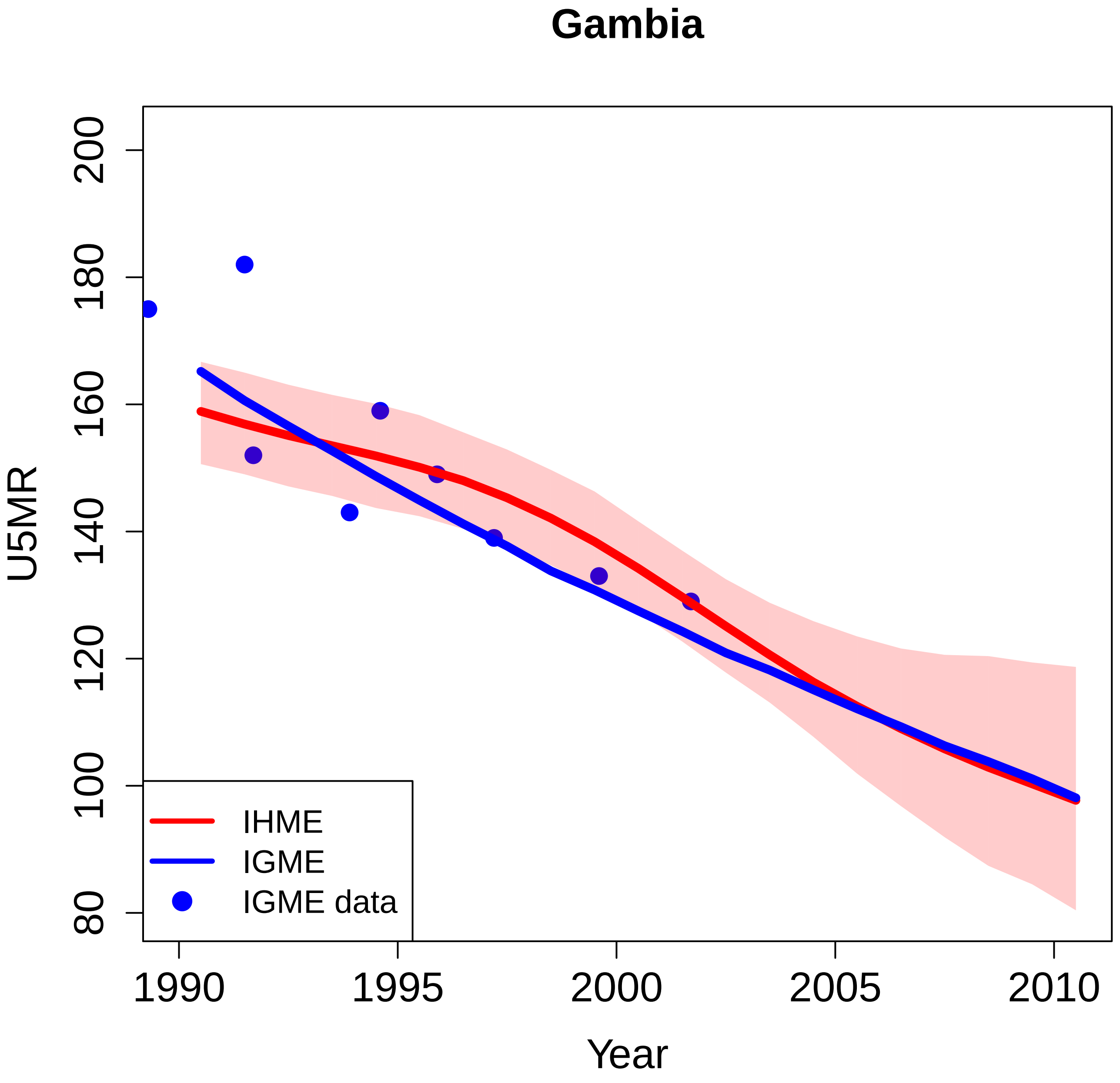
Gambia
U5MR
200
180
160
140
120
100
80
1990
1995
2000
2005
2010
Year
IHME
IGME
IGME data

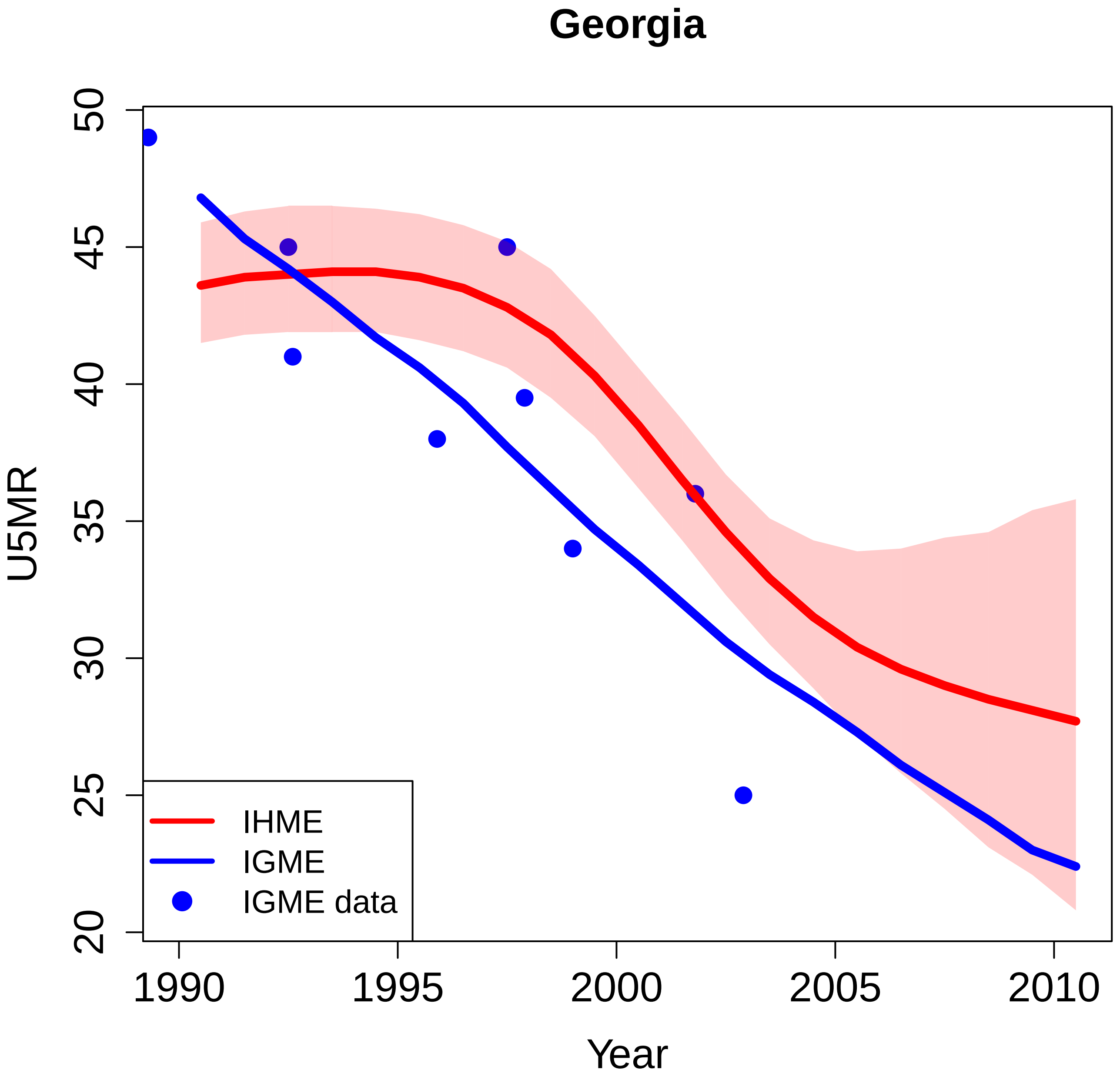
Georgia
U5MR
50
45
40
35
30
25
20
1990
1995
2000
2005
2010
Year
IHME
IGME
IGME data

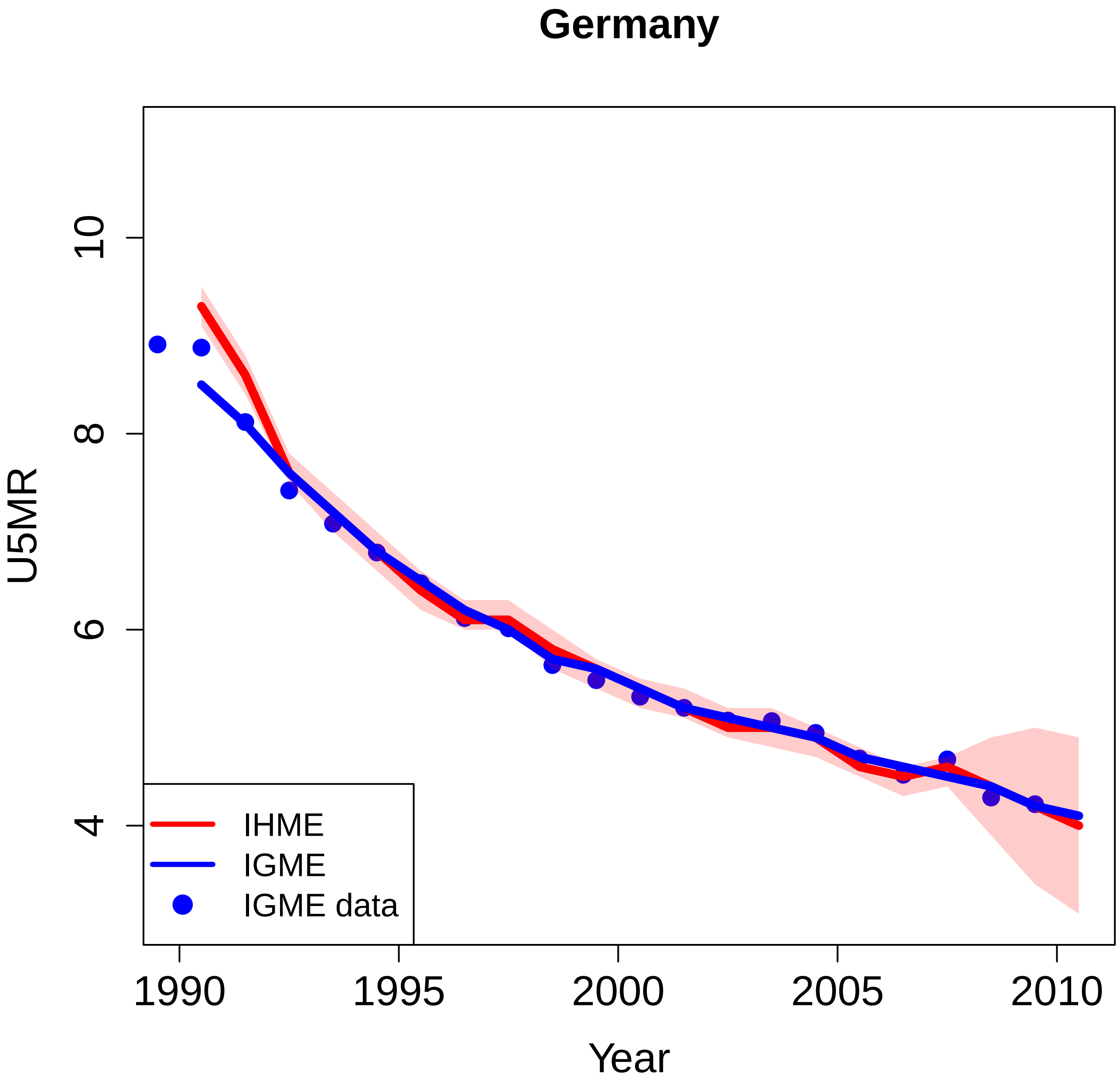

Germany
U5MR
Year
10
8
6
4
1990
1995
2000
2005
2010
IHME
IGME
IGME data

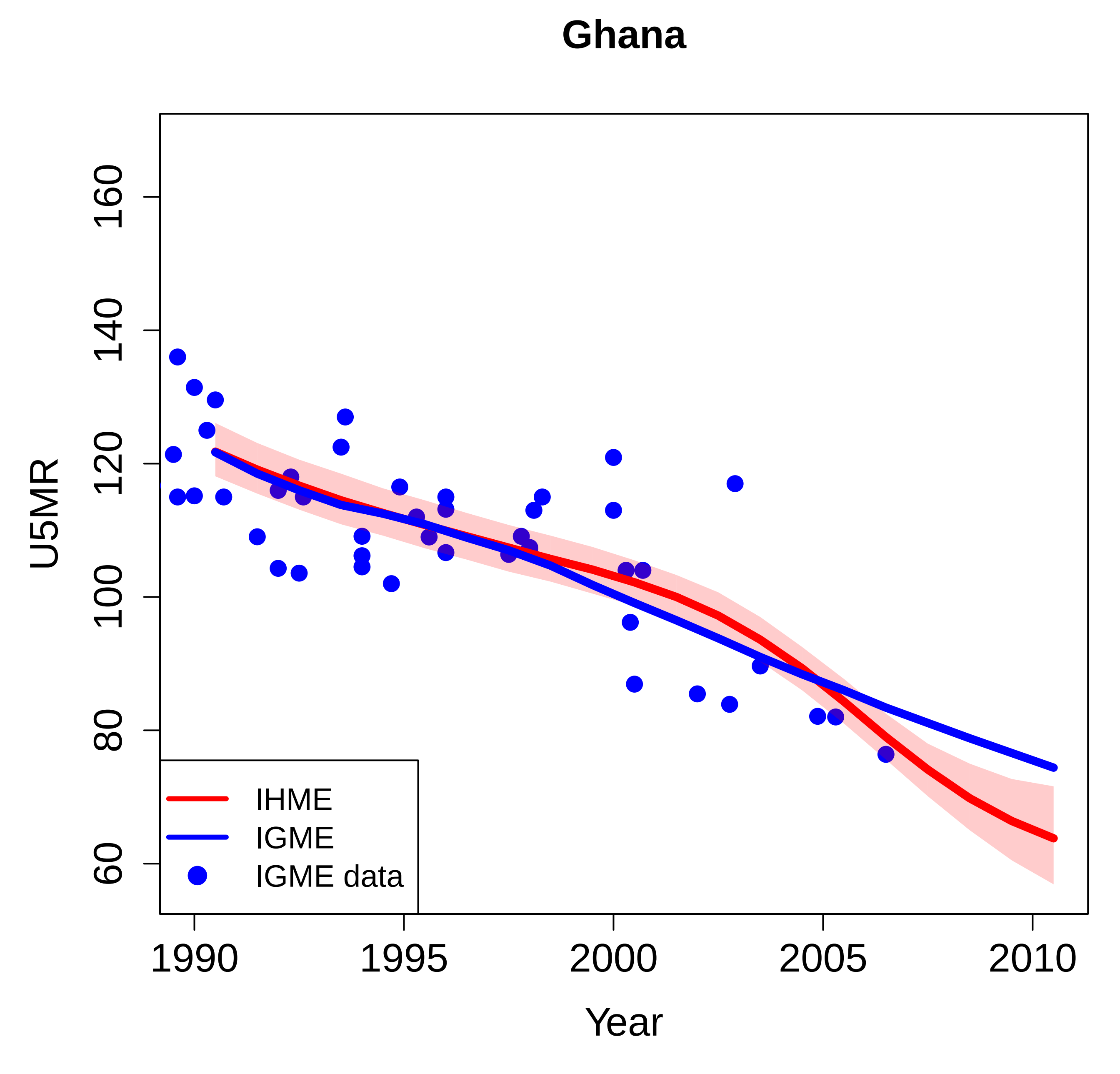

Ghana
U5MR
160
140
120
100
80
60
1990
1995
2000
2005
2010
Year
IHME
IGME
IGME data

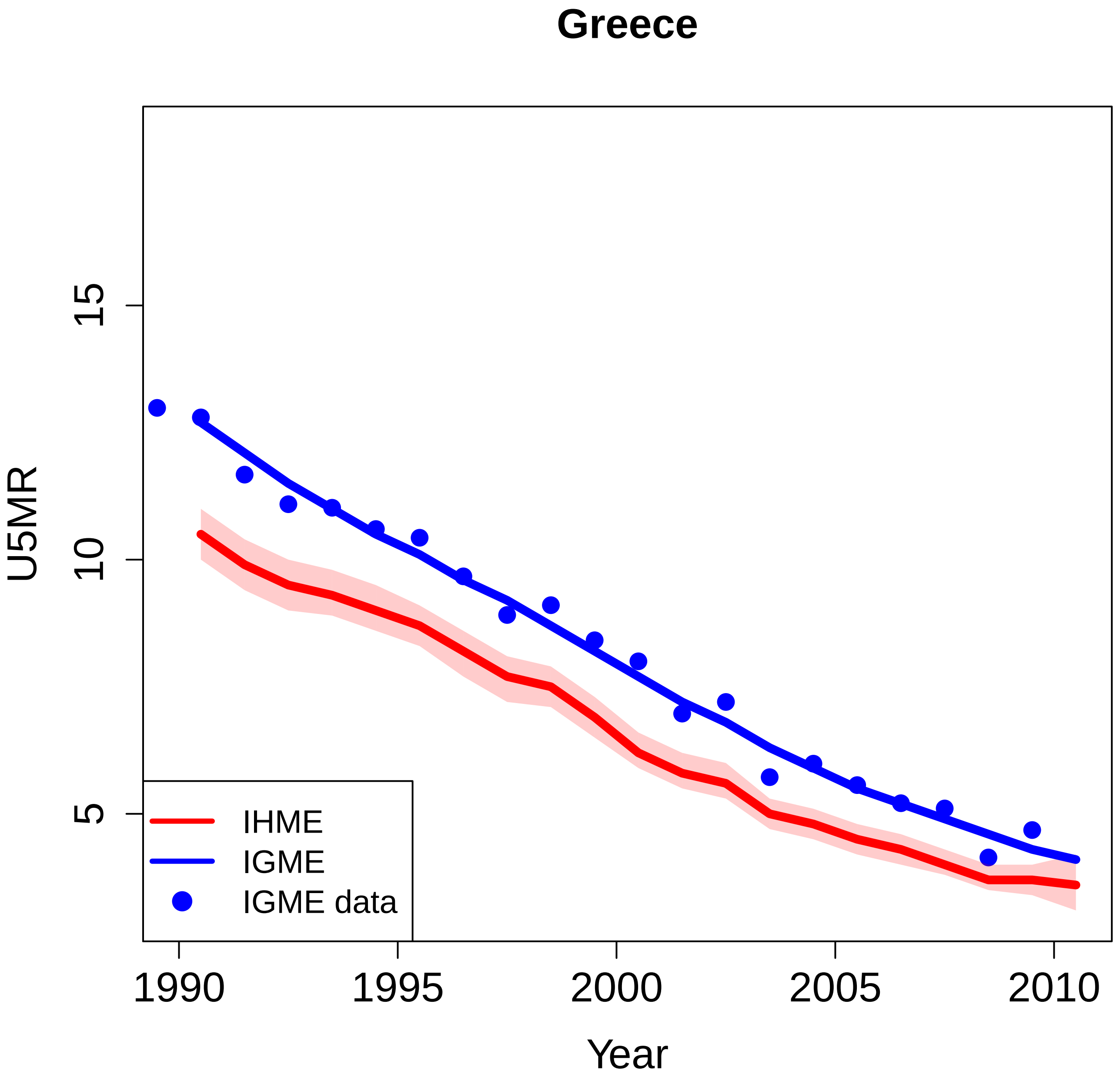

Greece
U5MR
15
10
5
1990
1995
2000
2005
2010
Year
IHME
IGME
IGME data

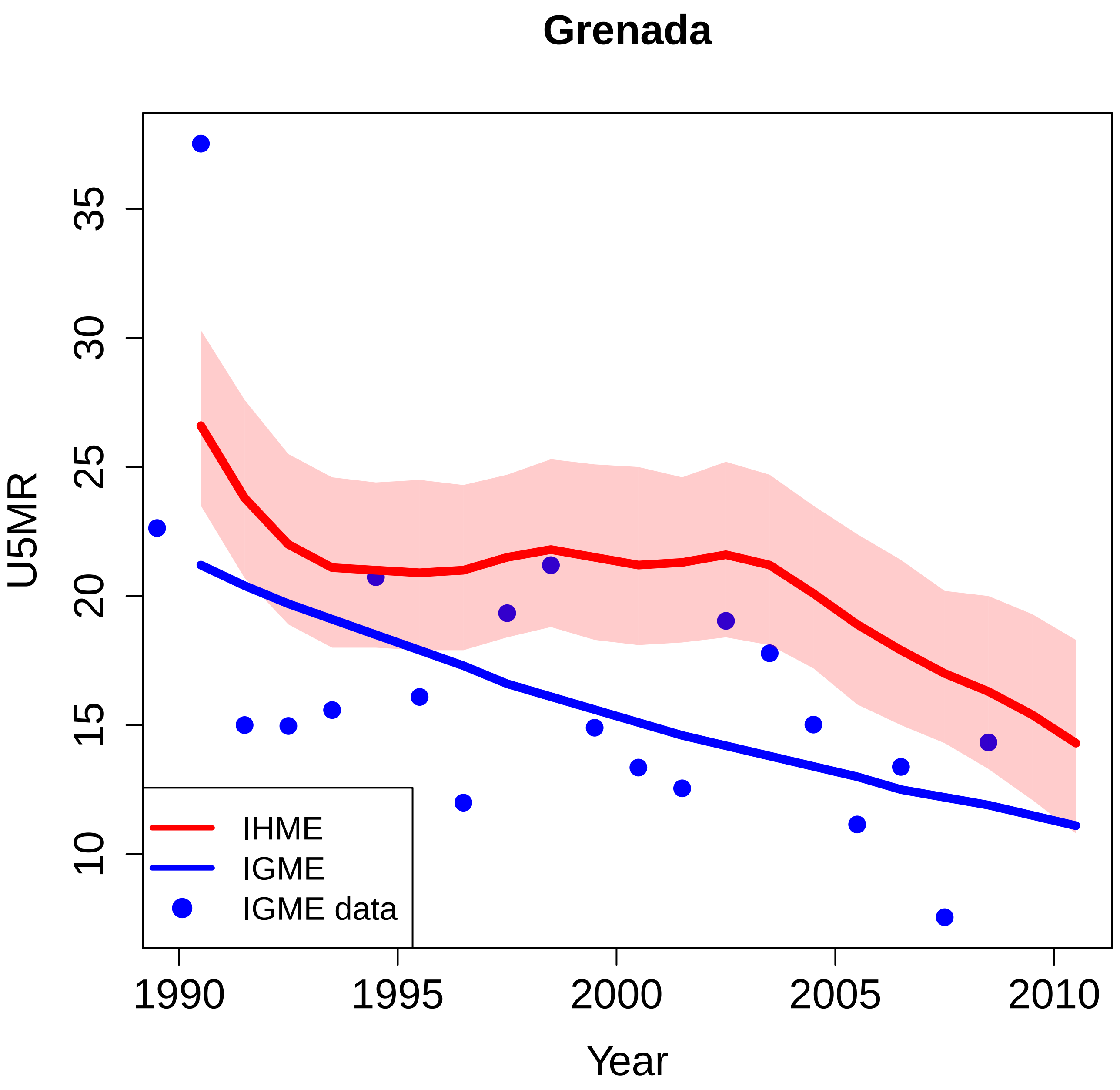

Grenada
U5MR
Year
10
15
20
25
30
35
1990
1995
2000
2005
2010
IHME
IGME
IGME data

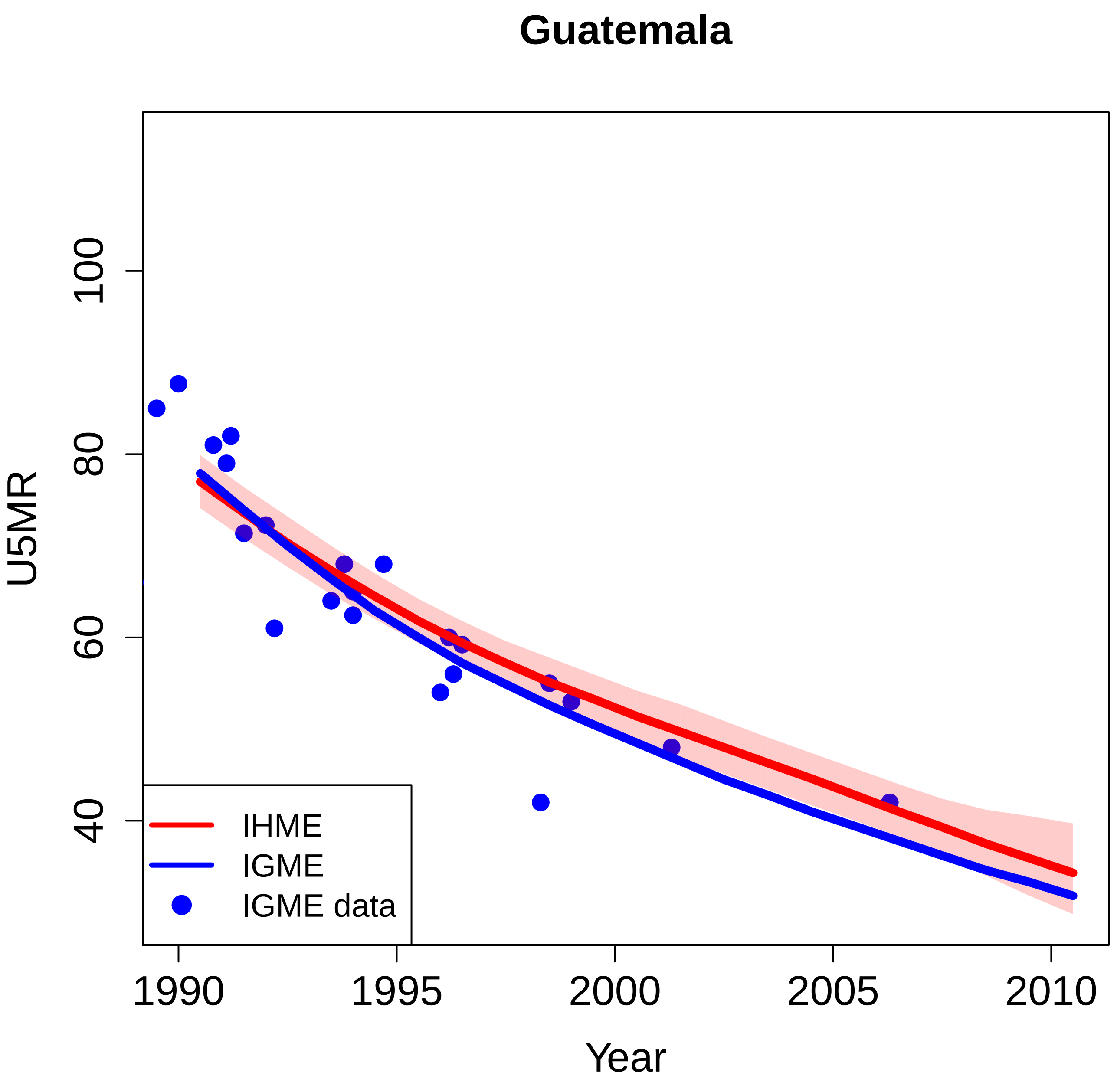

Guatemala
U5MR
100
80
60
40
1990
1995
2000
2005
2010
Year
IHME
IGME
IGME data

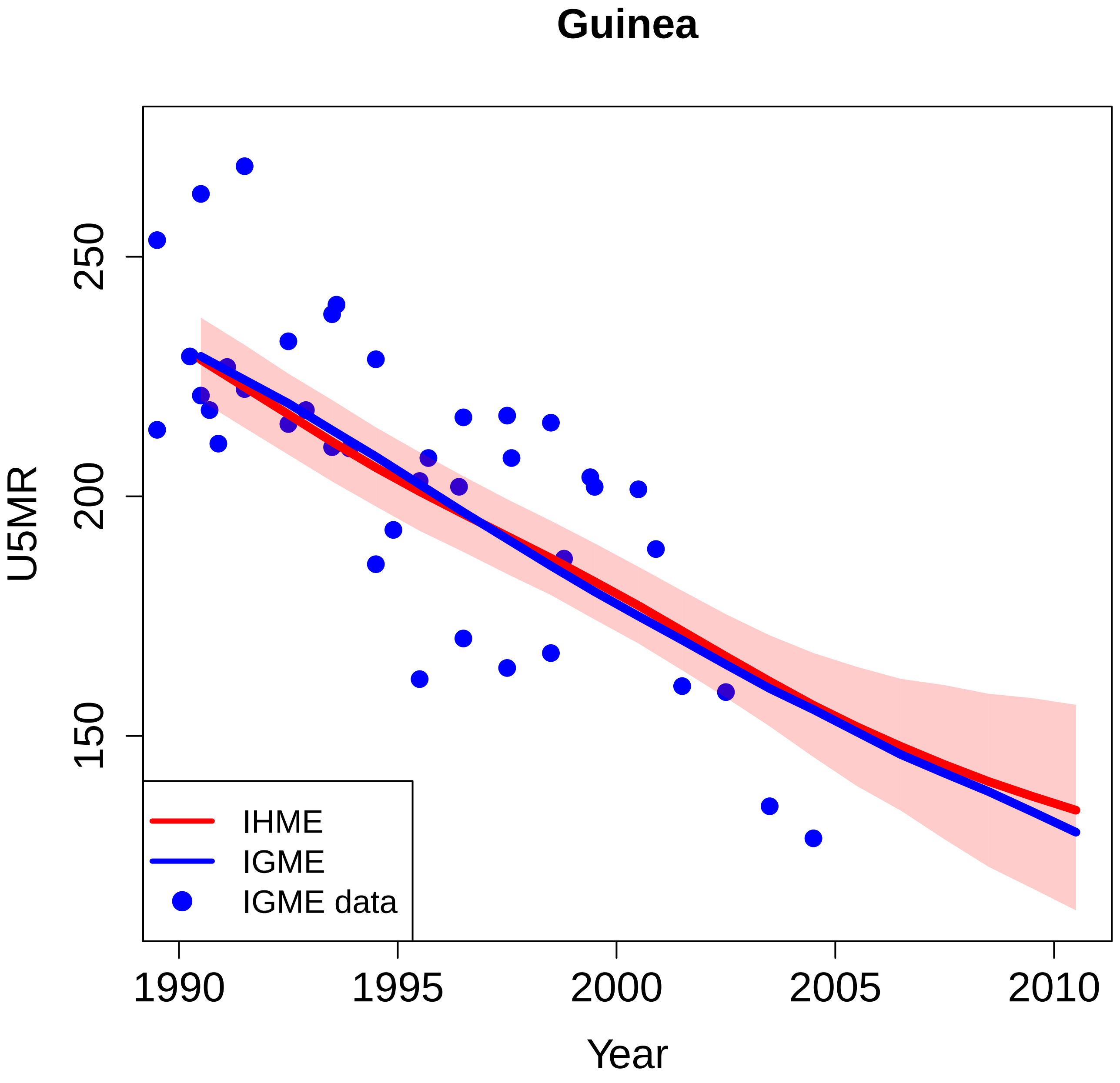
Guinea
U5MR
250
200
150
Year
1990
1995
2000
2005
2010
IHME
IGME
IGME data

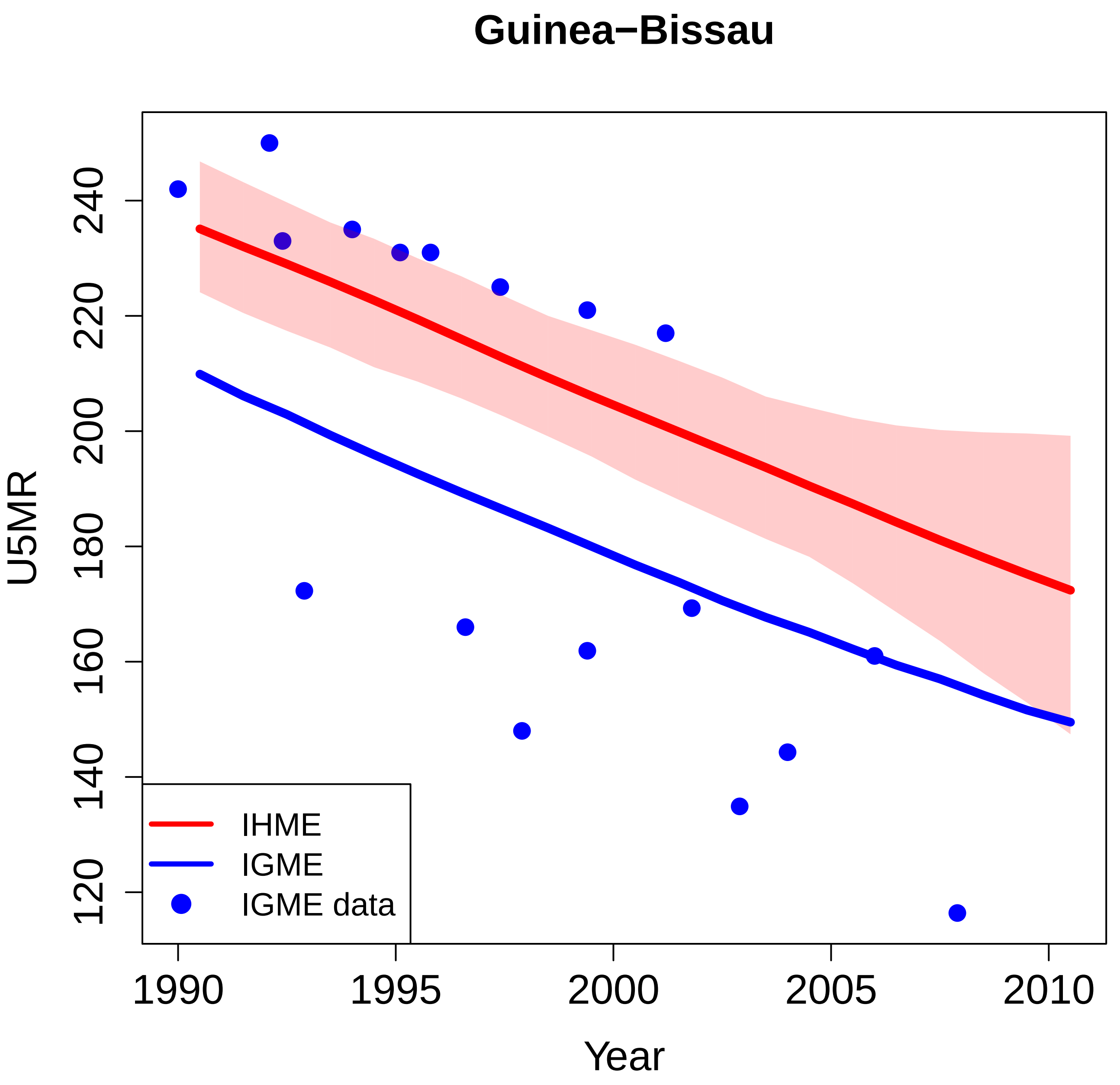
Guinea−Bissau
U5MR
Year
120
140
160
180
200
220
240
1990
1995
2000
2005
2010
IHME
IGME
IGME data

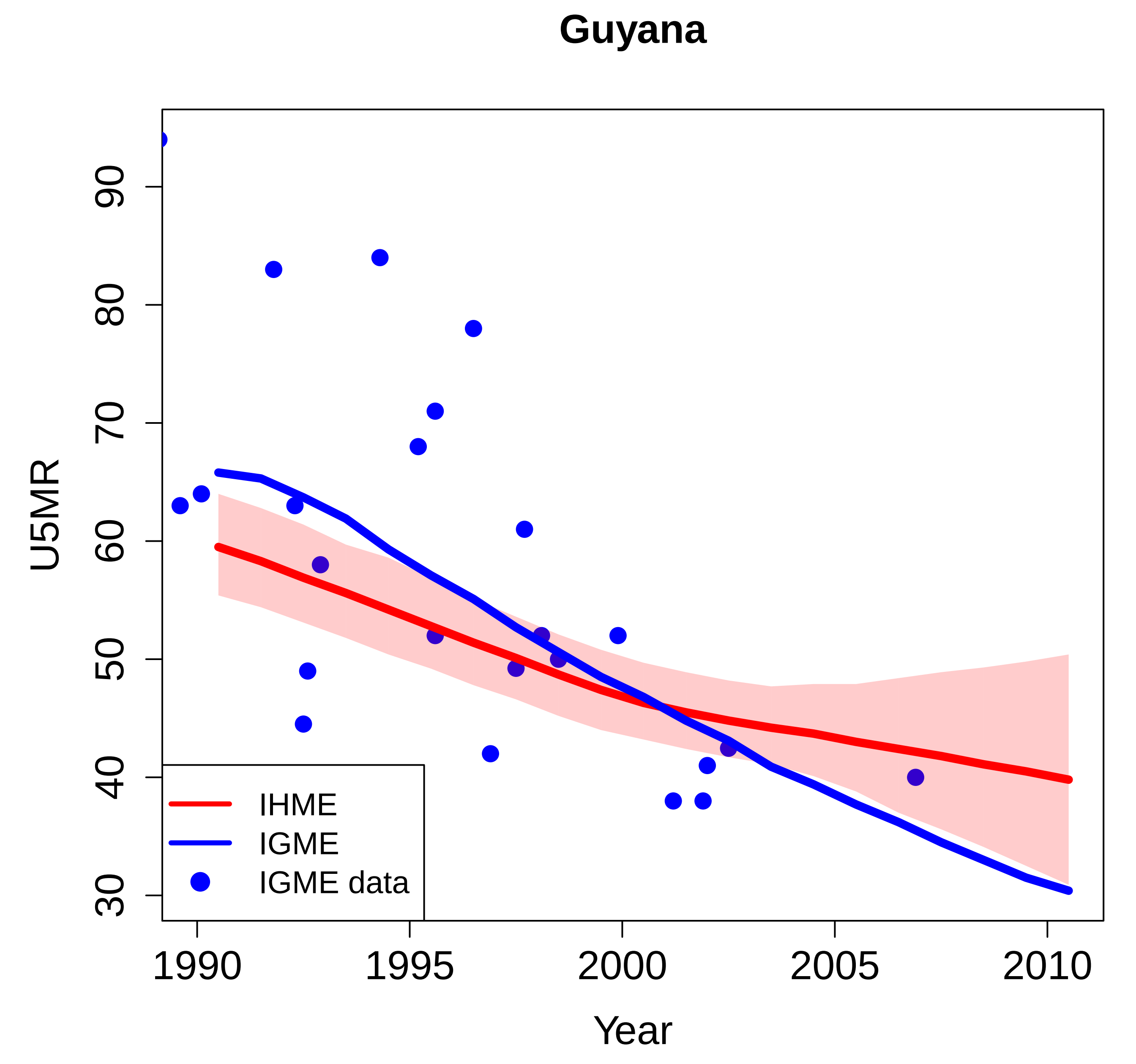
Guyana
U5MR
30
40
50
60
70
80
90
1990
1995
2000
2005
2010
Year
IHME
IGME
IGME data

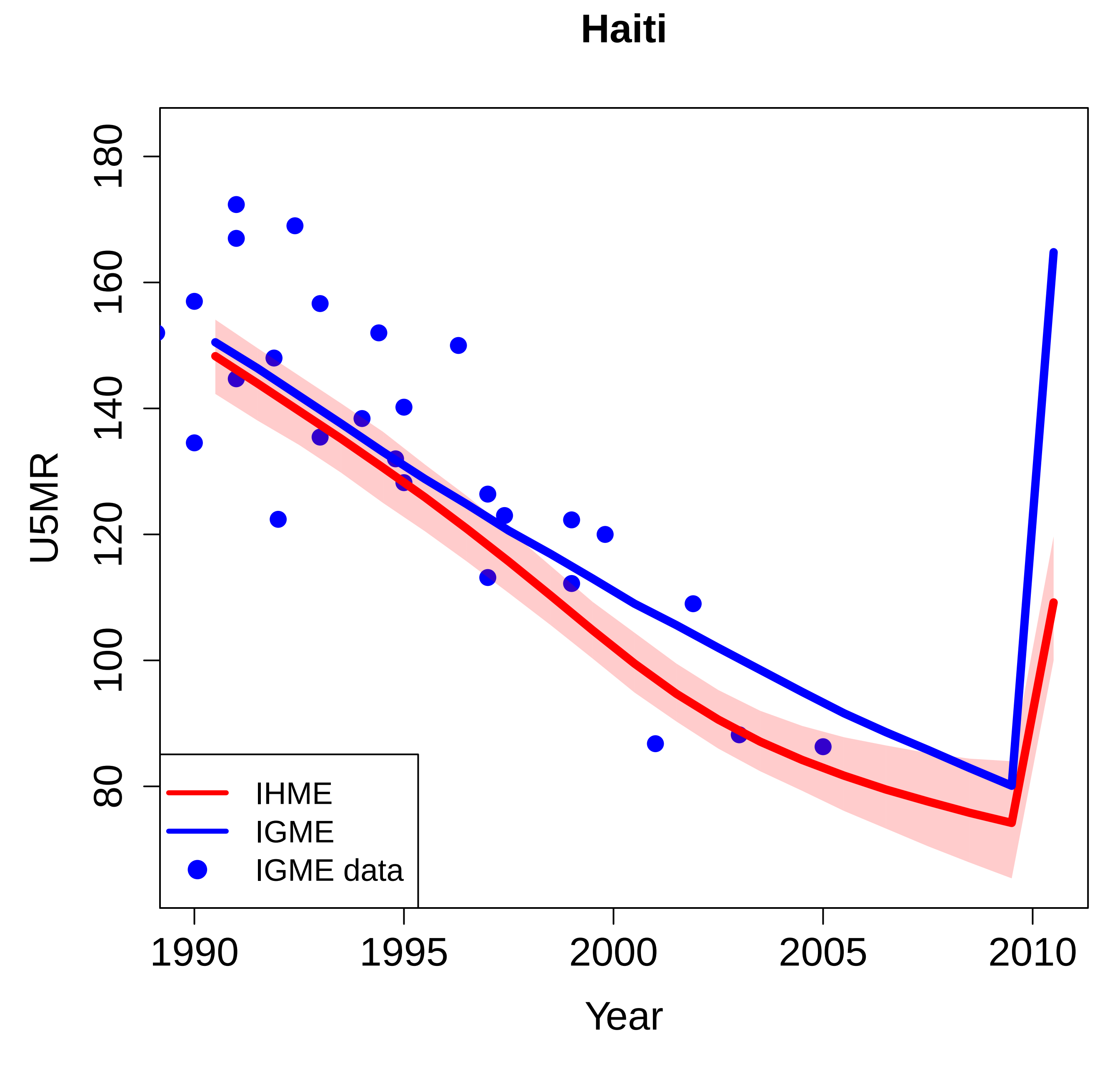

Haiti
U5MR
180
160
140
120
100
80
1990
1995
2000
2005
2010
Year
IHME
IGME
IGME data

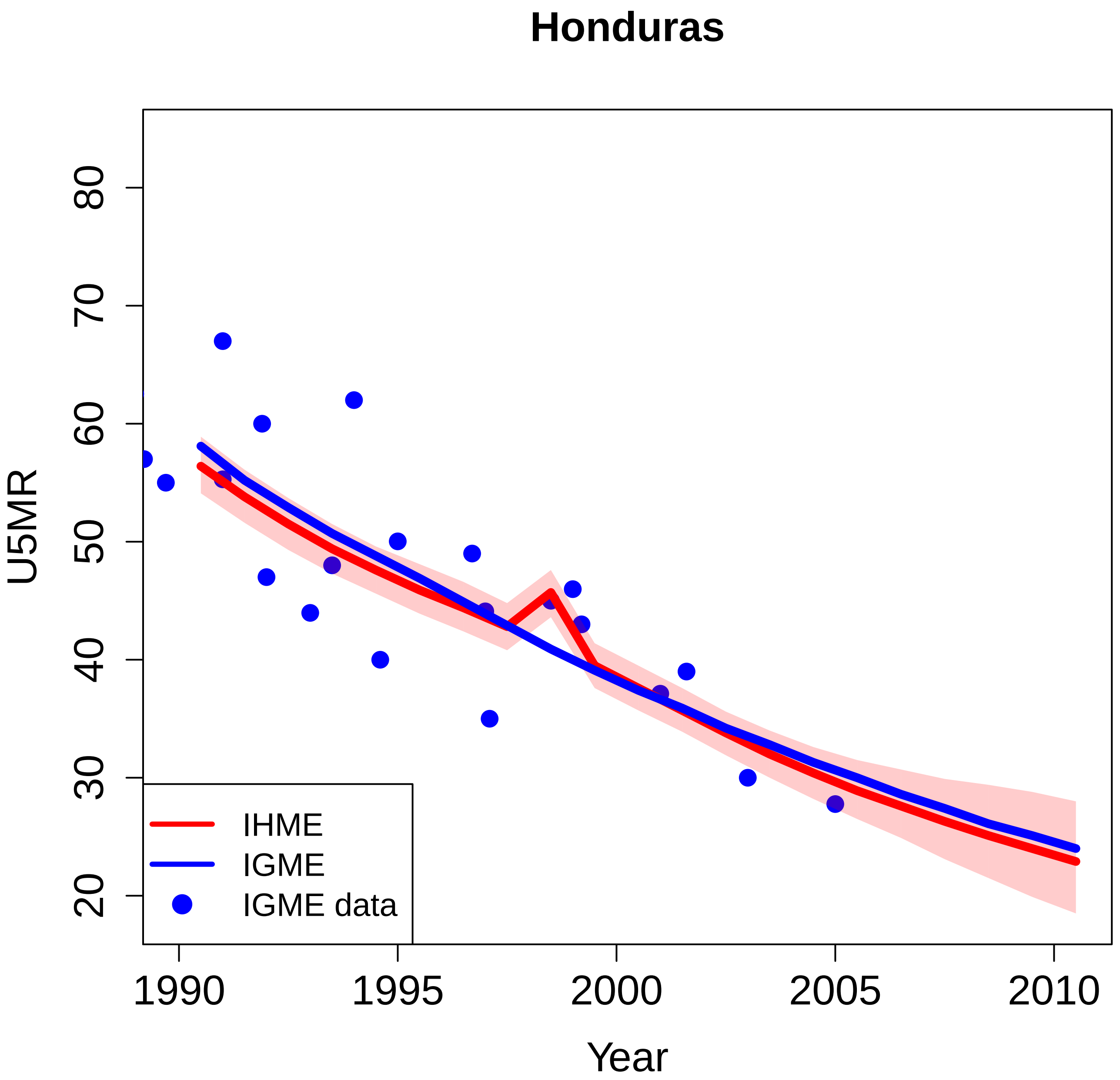

Honduras
U5MR
80
70
60
50
40
30
20
1990
1995
2000
2005
2010
Year
IHME
IGME
IGME data

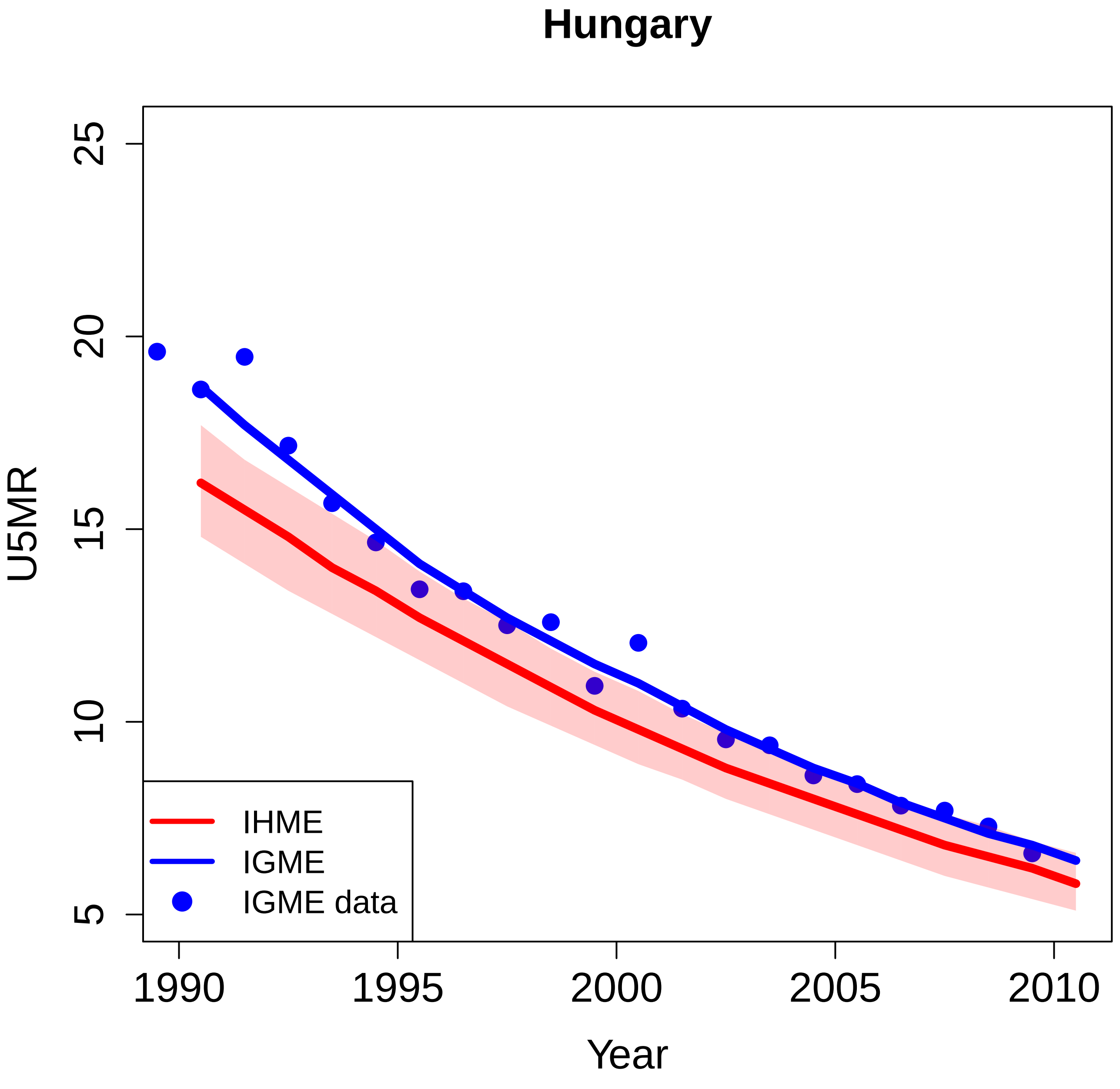
Hungary
U5MR
Year
25
20
15
10
5
1990
1995
2000
2005
2010
IHME
IGME
IGME data

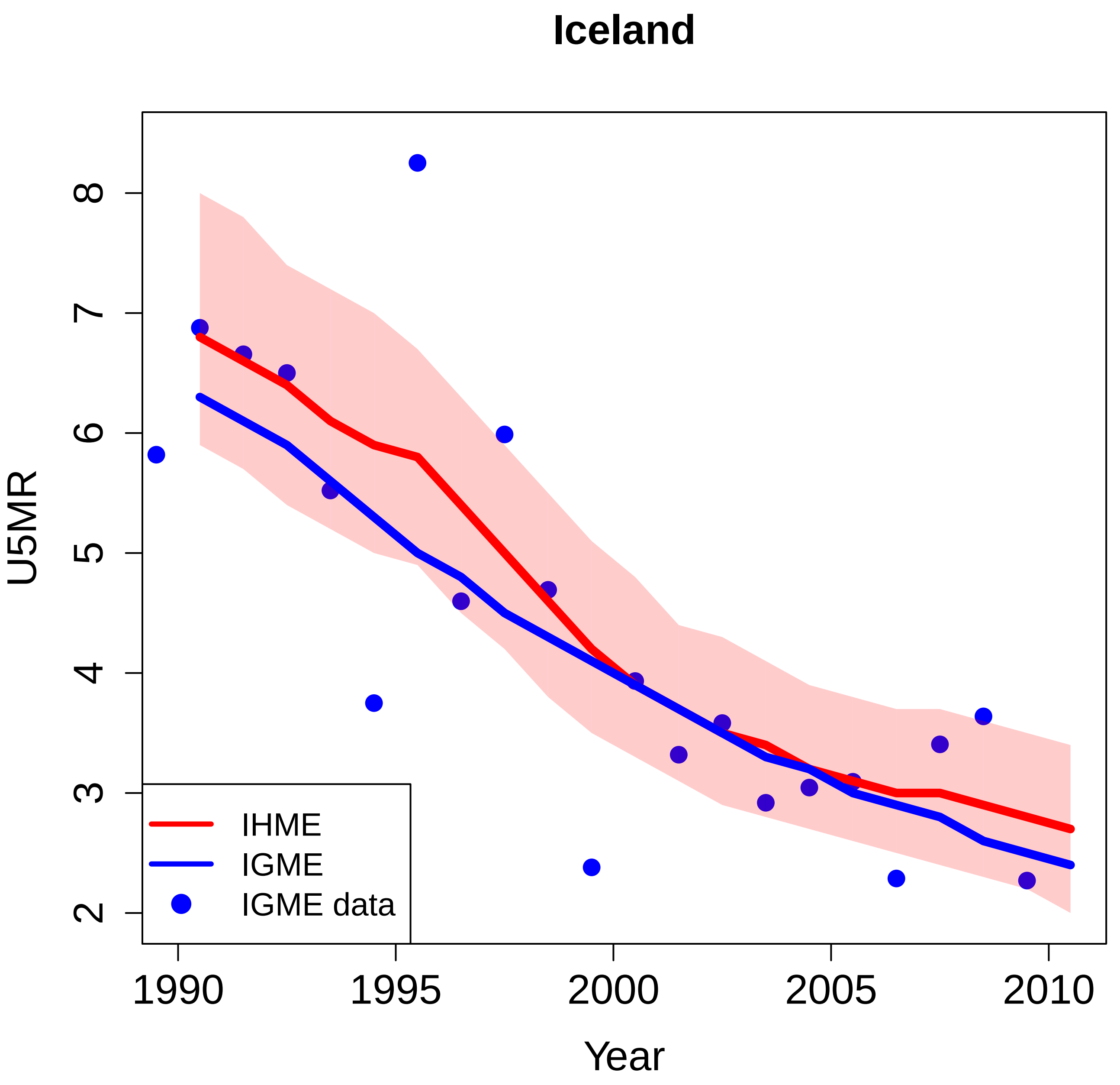
Iceland
U5MR
Year
2
3
4
5
6
7
8
1990
1995
2000
2005
2010
IHME
IGME
IGME data

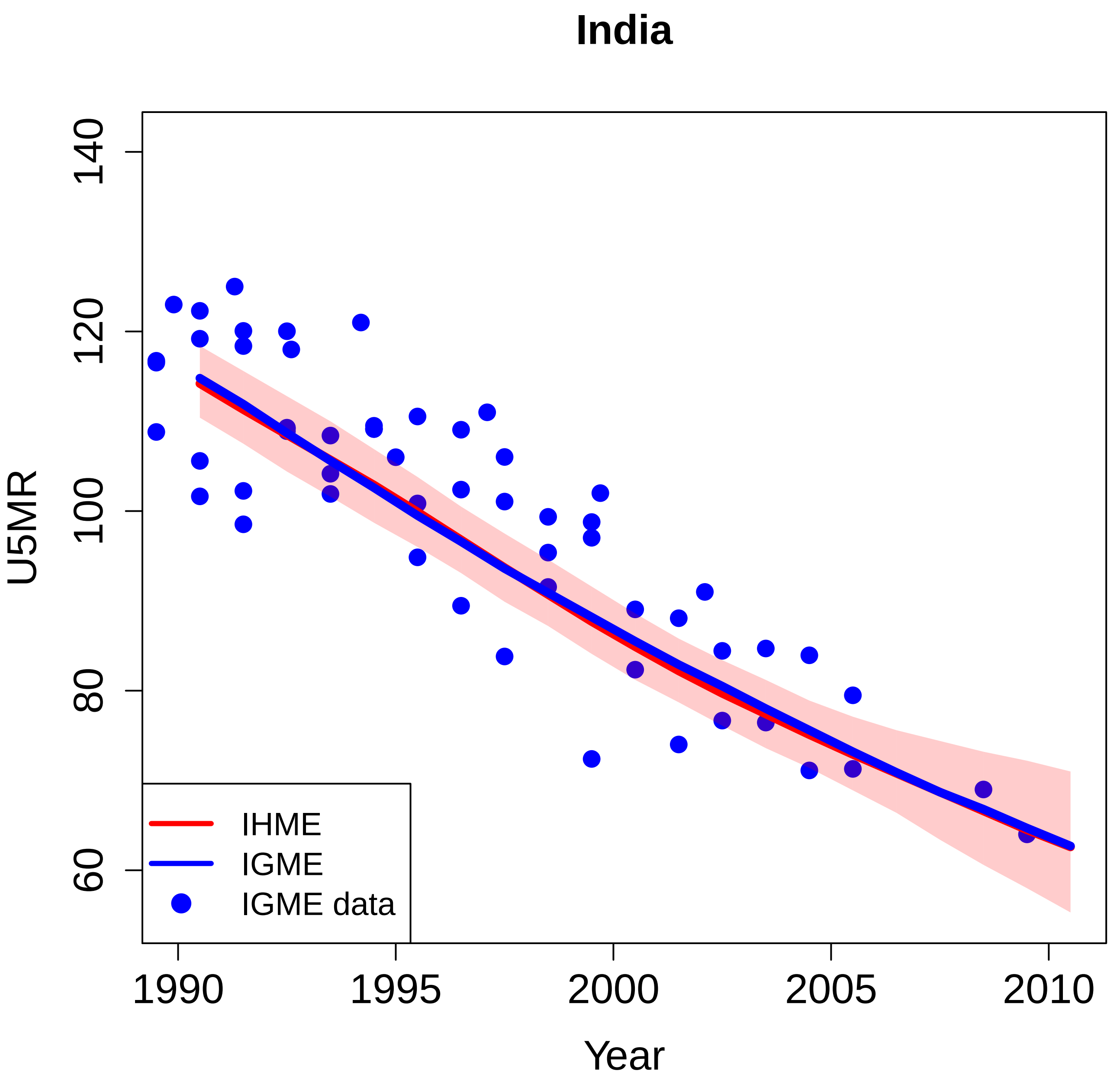
India
U5MR
140
120
100
80
60
1990
1995
2000
2005
2010
Year
IHME
IGME
IGME data

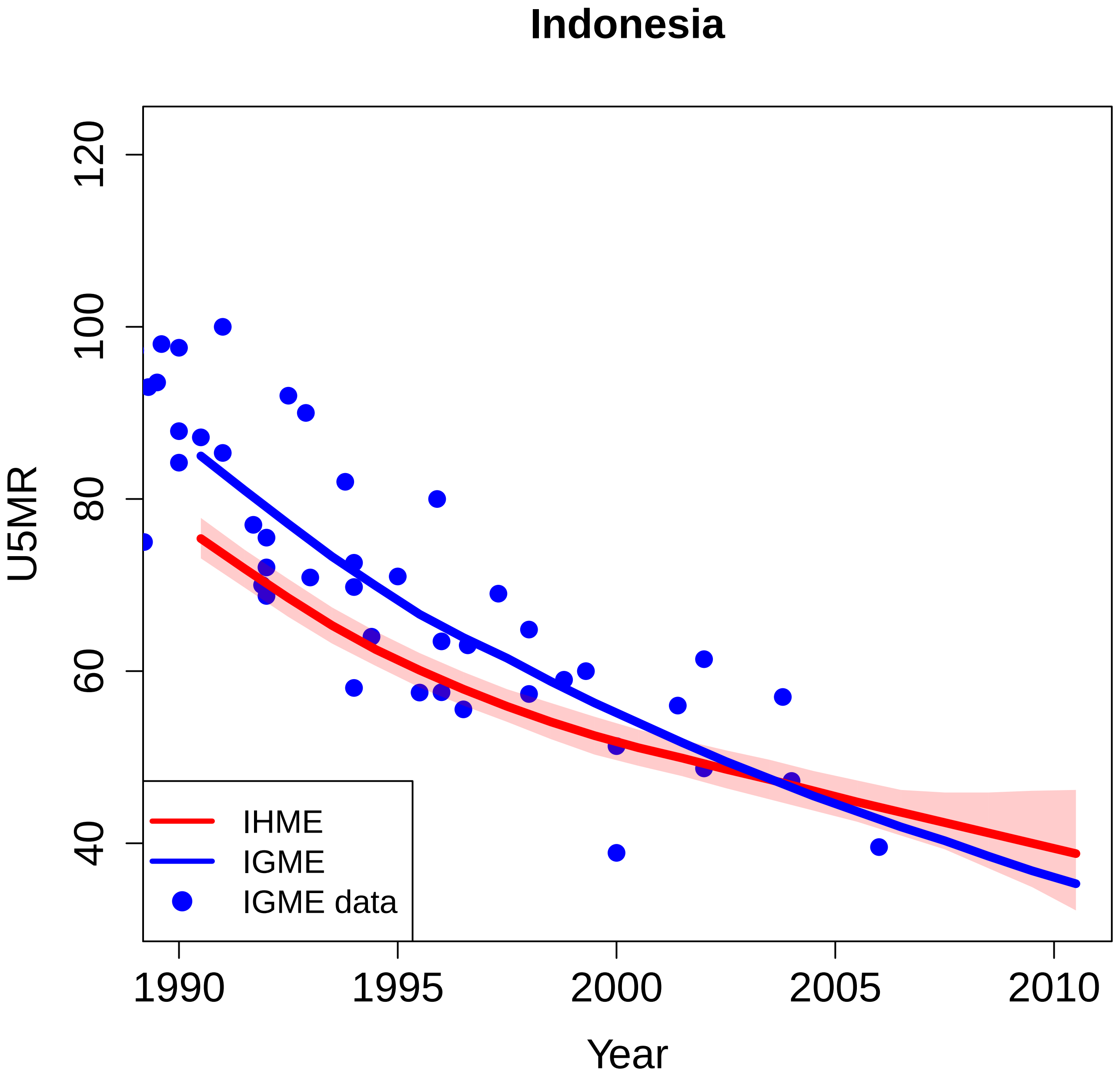
Indonesia
U5MR
120
100
80
60
40
1990
1995
2000
2005
2010
Year
IHME
IGME
IGME data

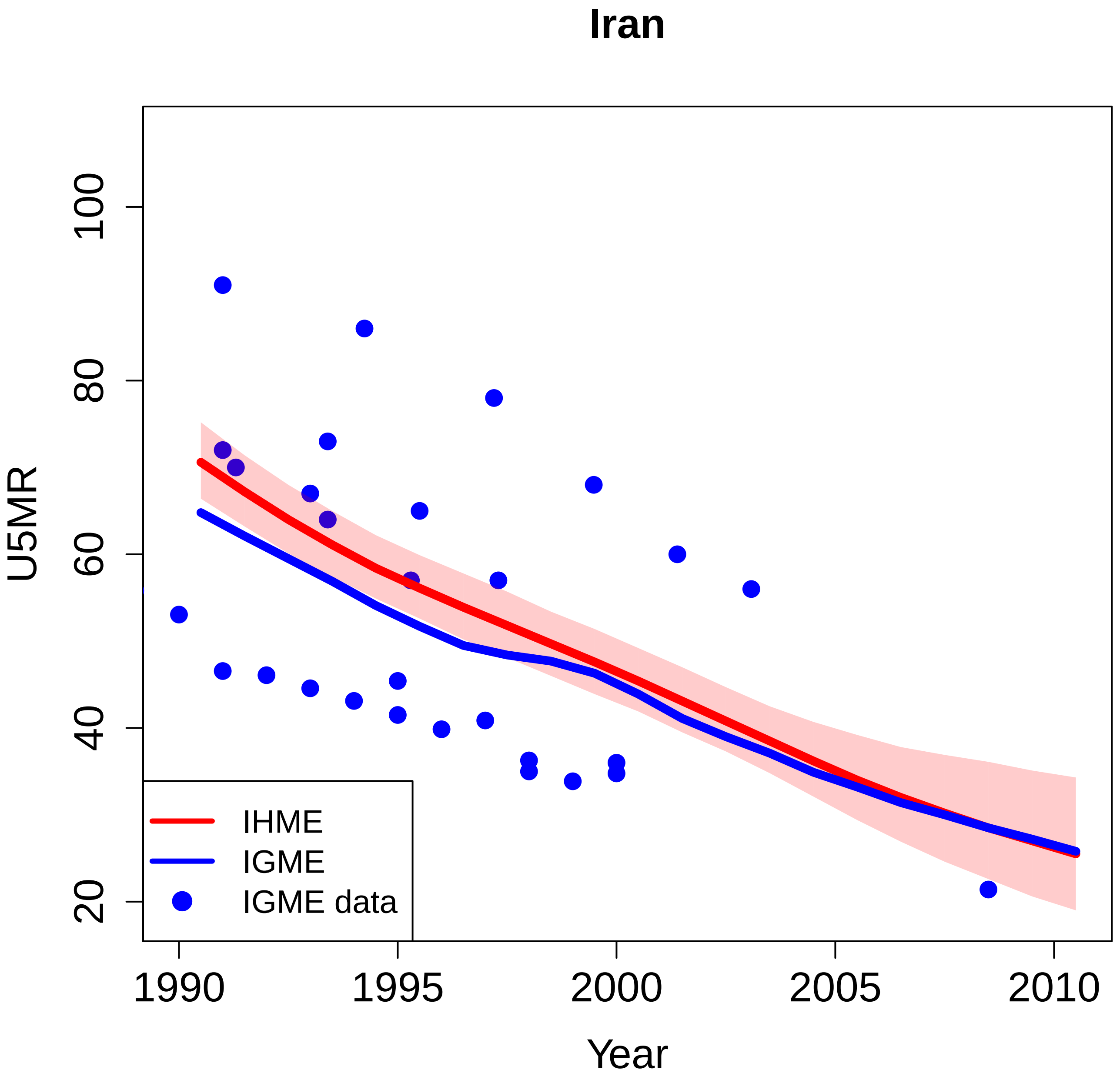
Iran
U5MR
Year
20
40
60
80
100
1990
1995
2000
2005
2010
IHME
IGME
IGME data

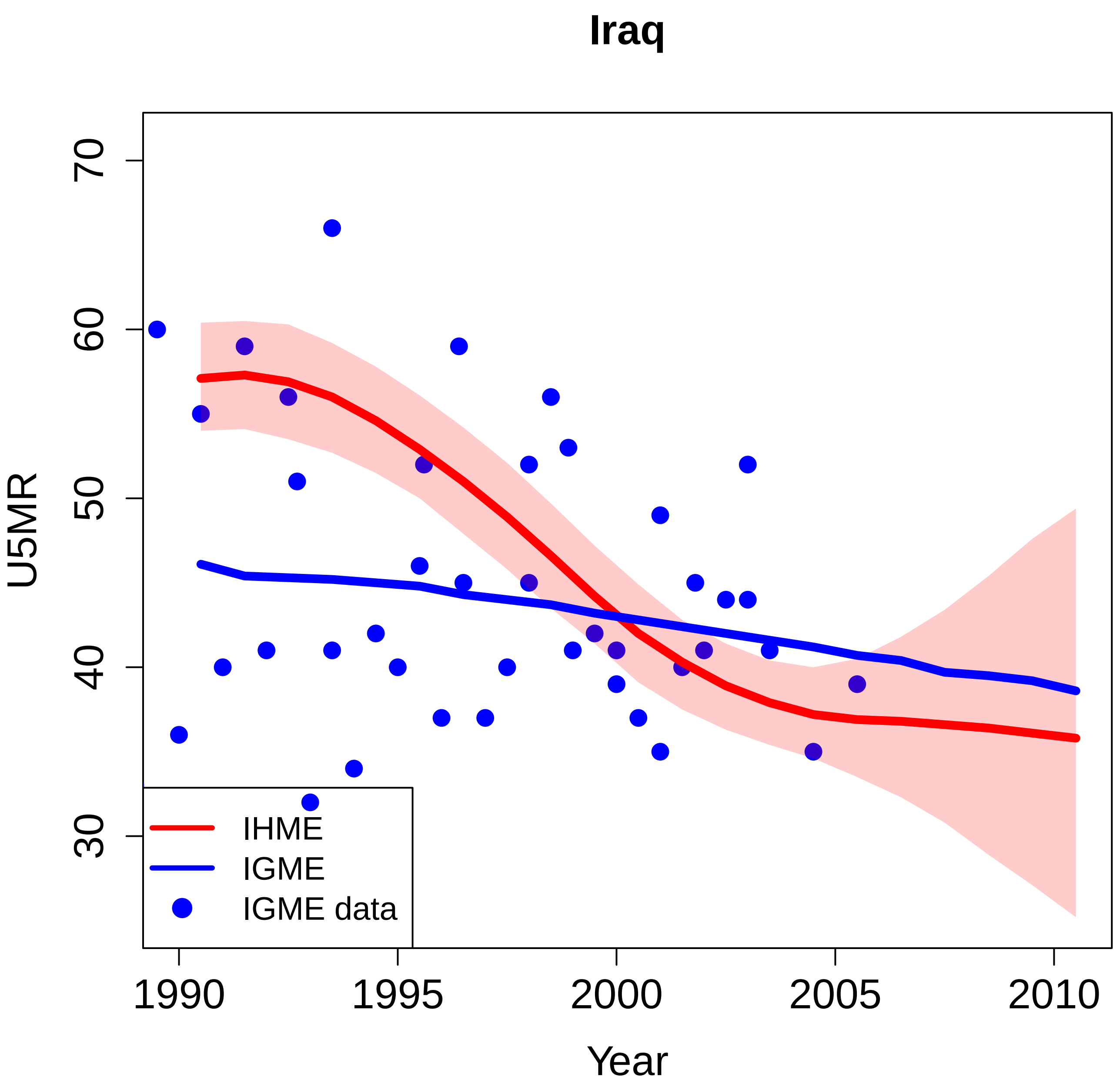
Iraq
U5MR
70
60
50
40
30
1990
1995
2000
2005
2010
Year
IHME
IGME
IGME data

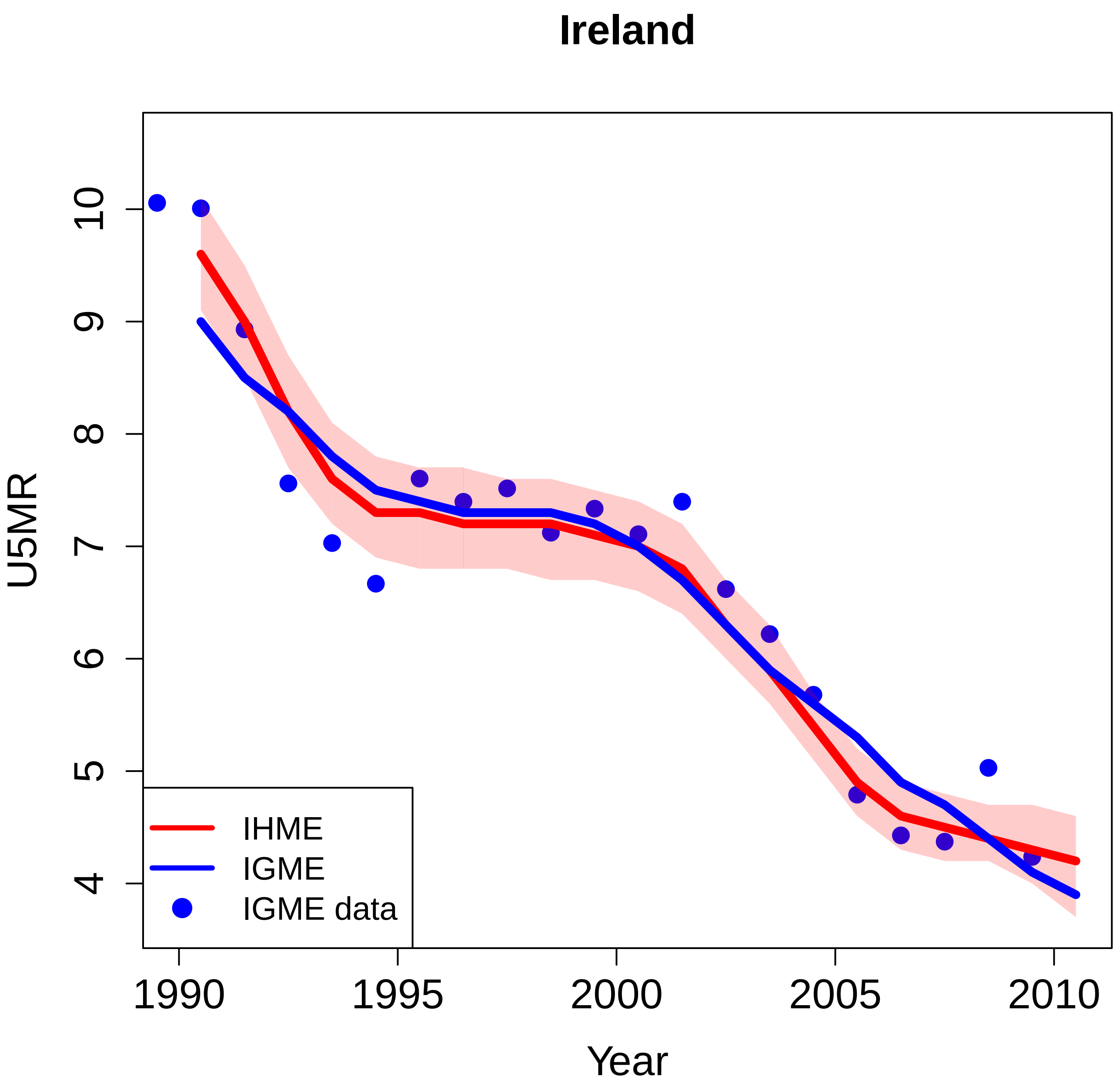
Ireland
U5MR
Year
10
9
8
7
6
5
4
1990
1995
2000
2005
2010
IHME
IGME
IGME data

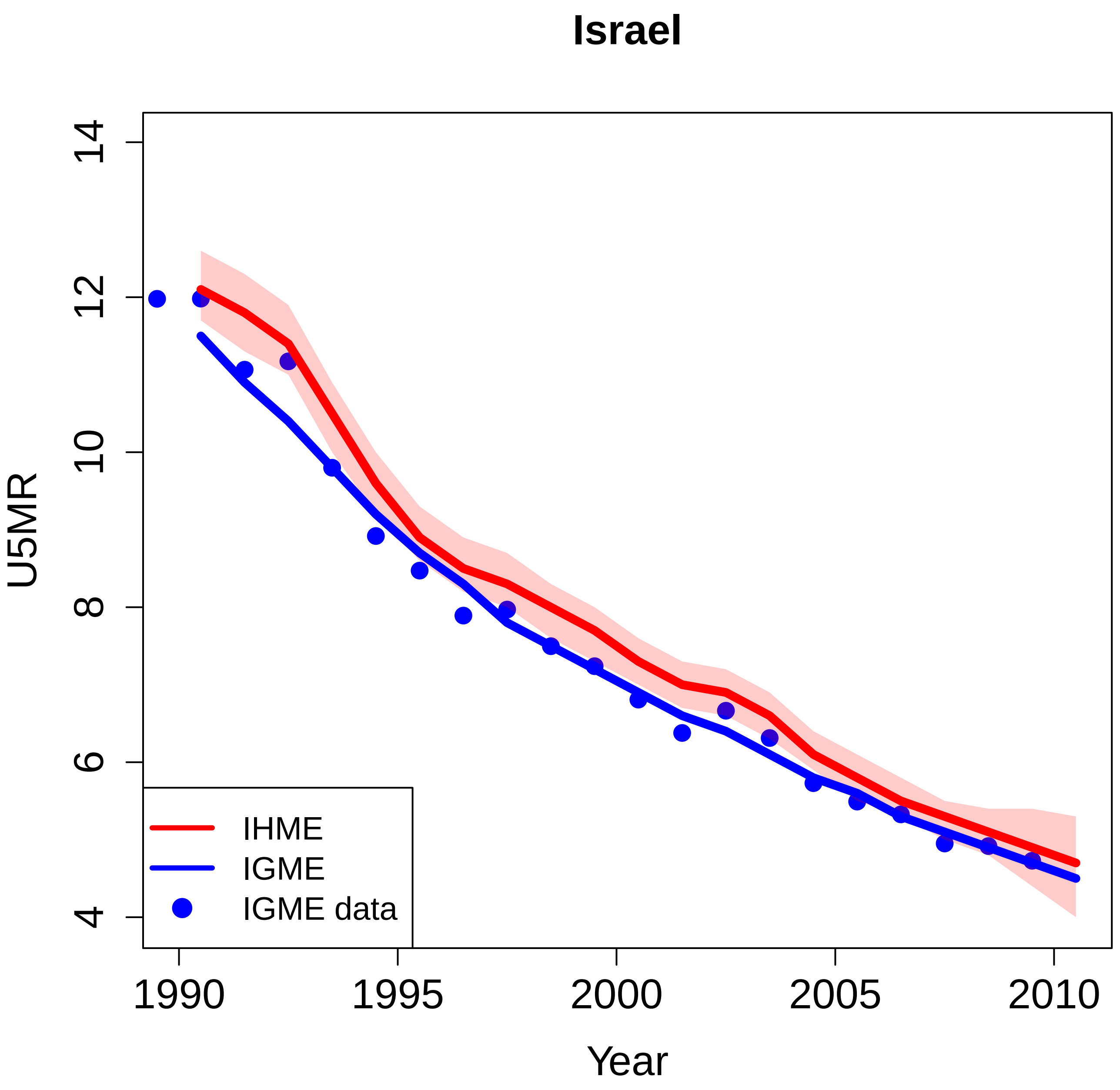
Israel
U5MR
Year
4
6
8
10
12
14
1990
1995
2000
2005
2010
IHME
IGME
IGME data

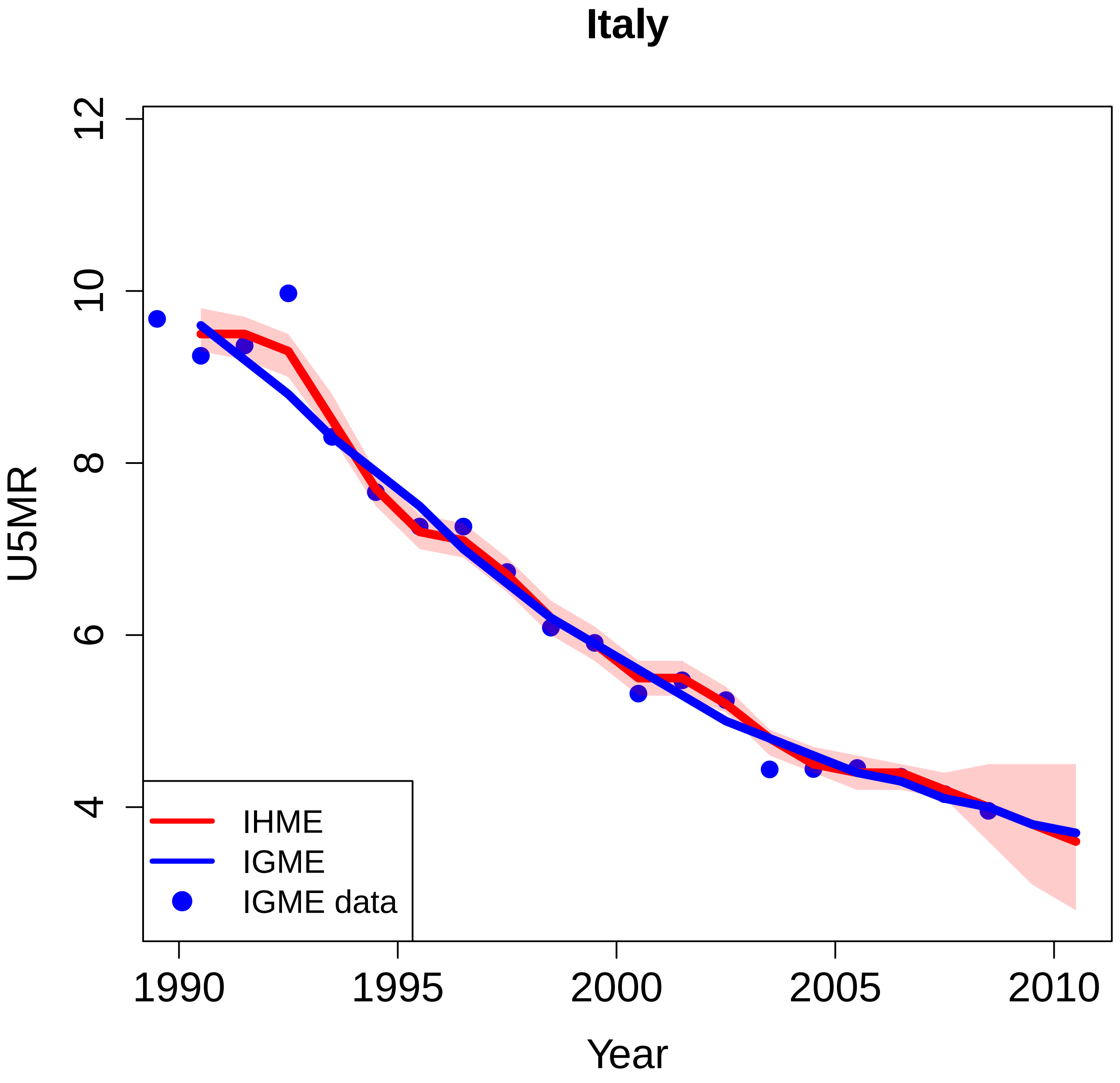
Italy
U5MR
12
10
8
6
4
1990
1995
2000
2005
2010
Year
IHME
IGME
IGME data

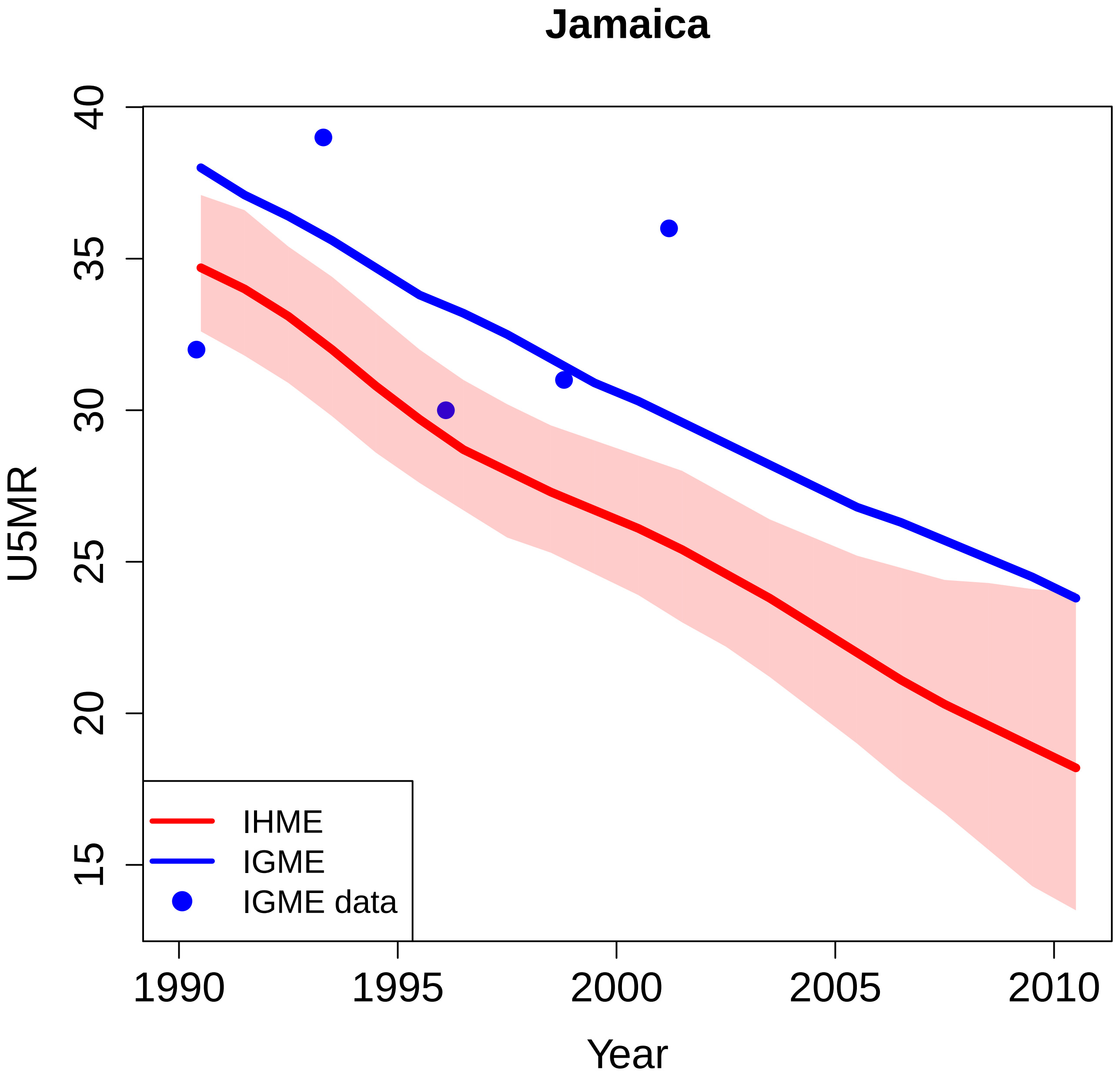
Jamaica
U5MR
40
35
30
25
20
15
1990
1995
2000
2005
2010
Year
IHME
IGME
IGME data

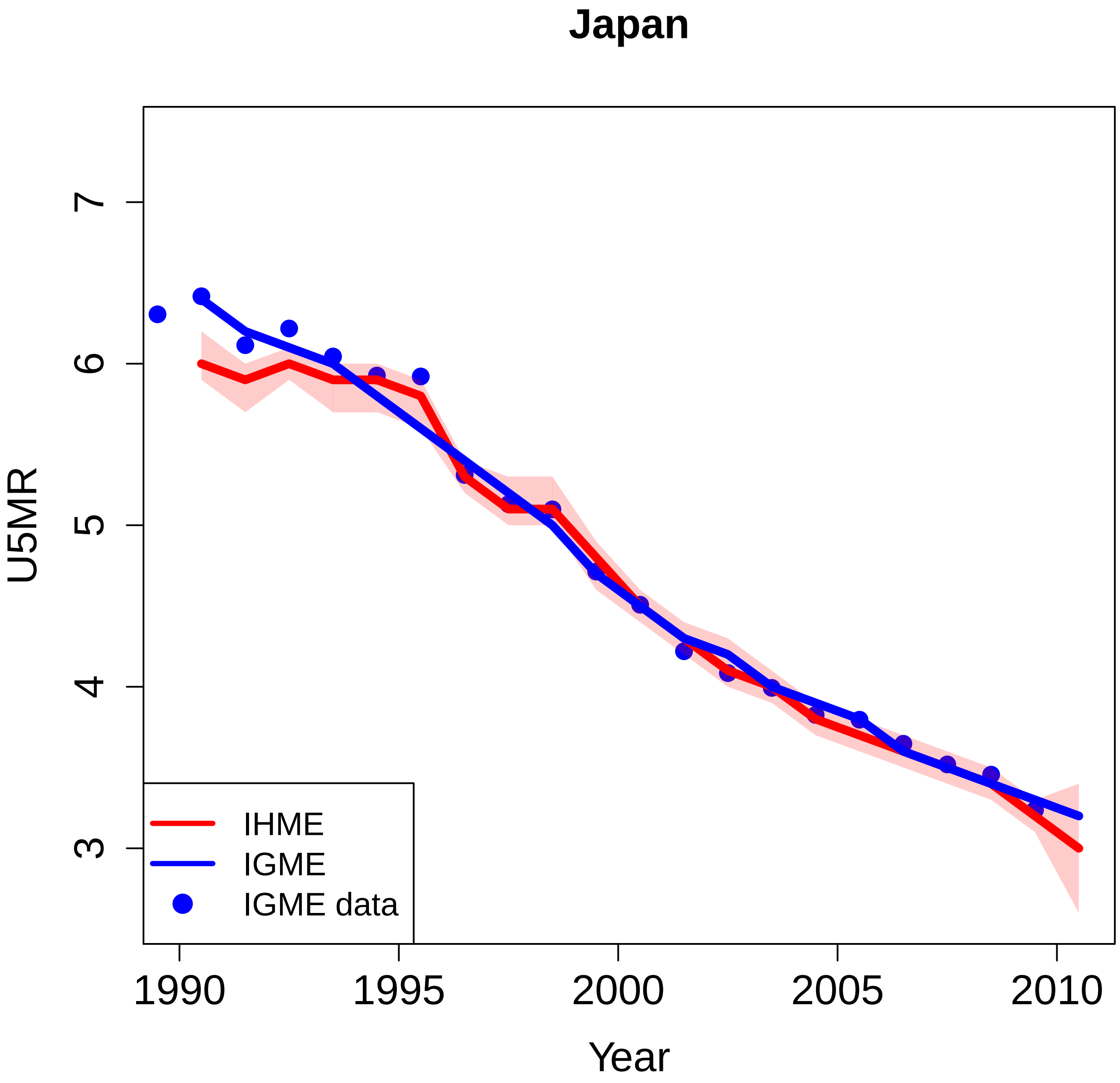
Japan
U5MR
7
6
5
4
3
1990
1995
2000
2005
2010
Year
IHME
IGME
IGME data

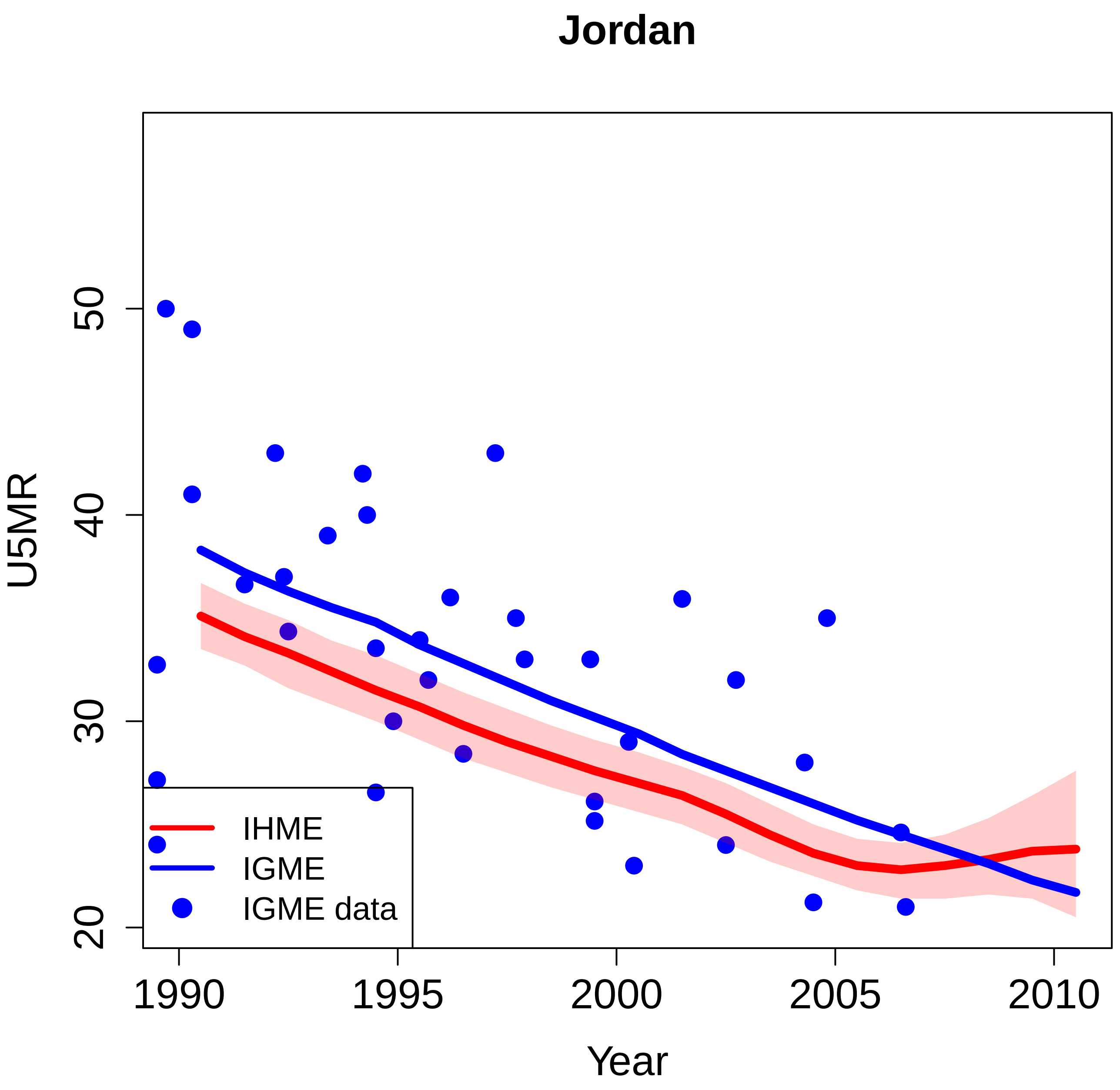

Jordan
U5MR
50
40
30
20
1990
1995
2000
2005
2010
Year
IHME
IGME
IGME data

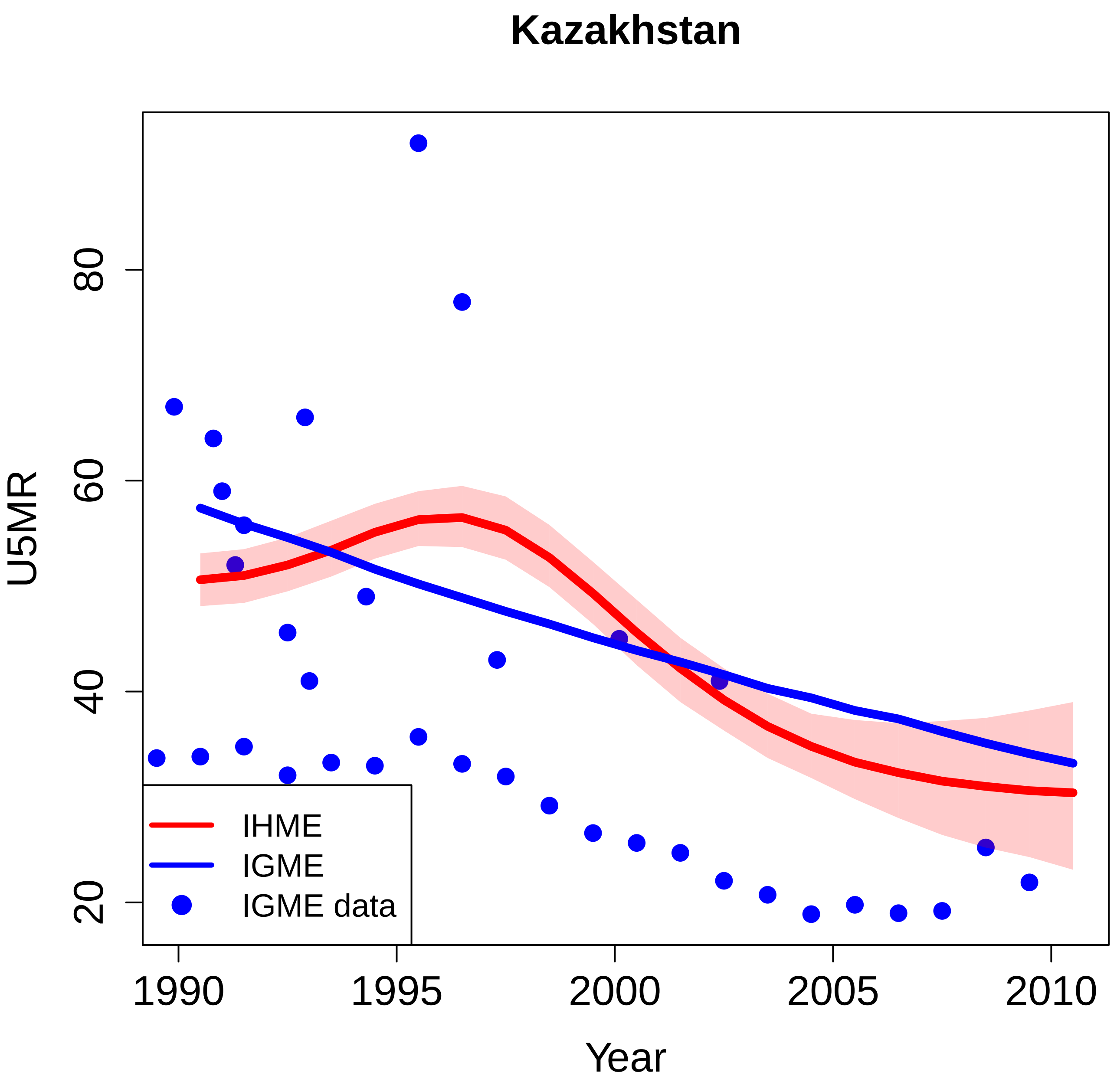

Kazakhstan
U5MR
80
60
40
20
1990
1995
2000
2005
2010
Year
IHME
IGME
IGME data

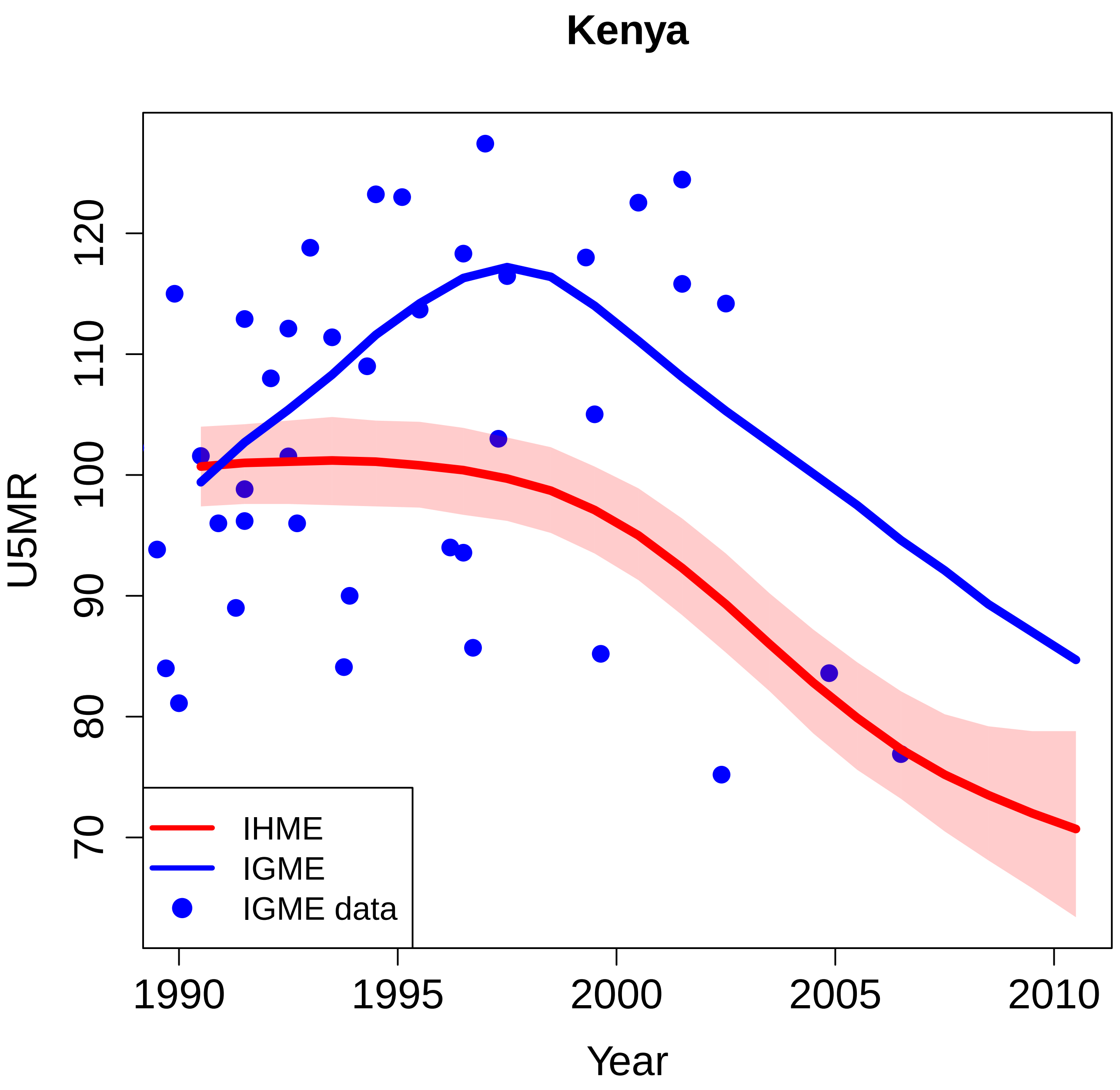
Kenya
U5MR
Year
70
80
90
100
110
120
1990
1995
2000
2005
2010
IHME
IGME
IGME data

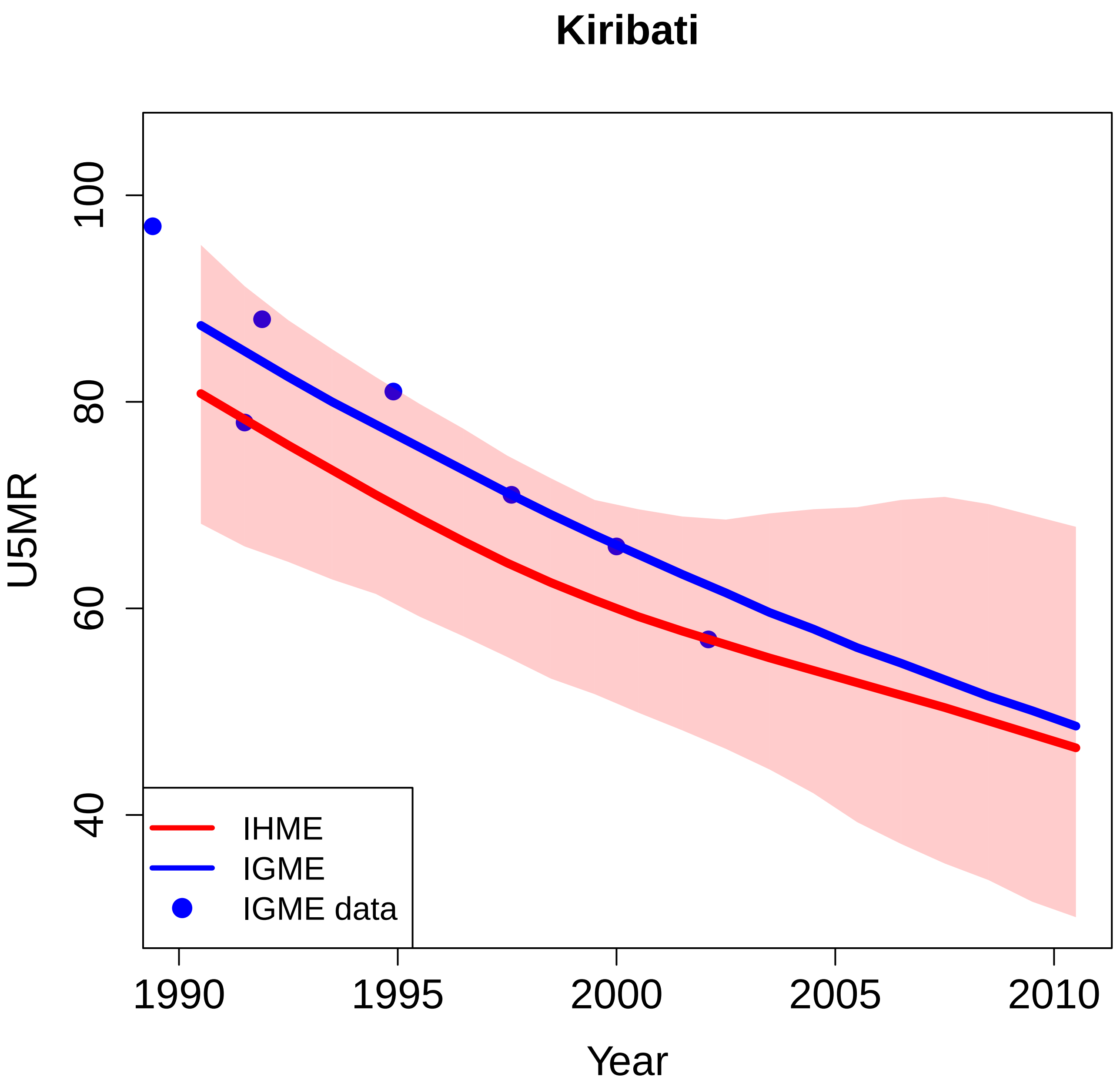

Kiribati
100
80
60
40
U5MR
1990
1995
2000
2005
2010
Year
IHME
IGME
IGME data

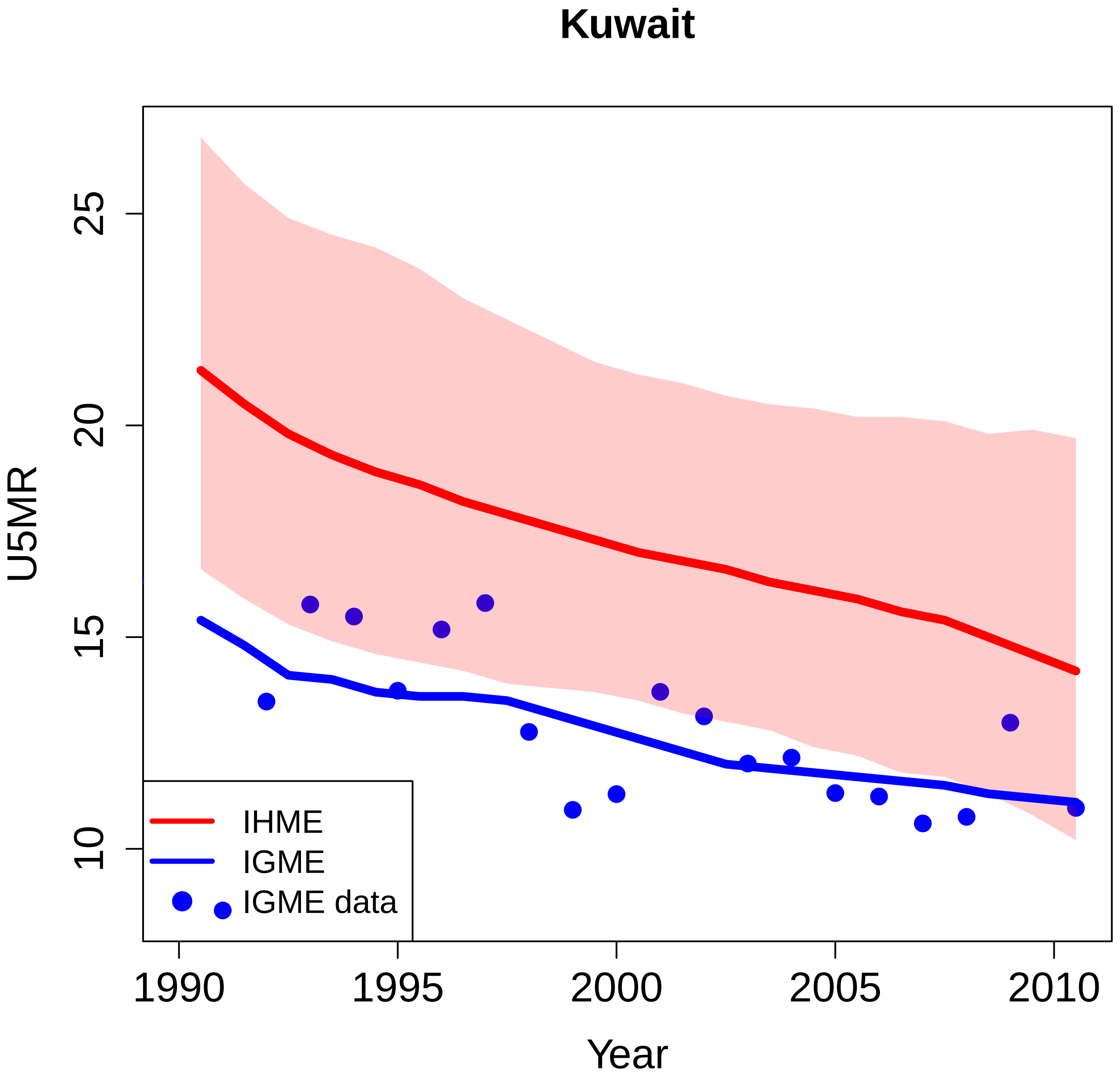

Kuwait
U5MR
Year
25
20
15
10
1990
1995
2000
2005
2010
IHME
IGME
IGME data

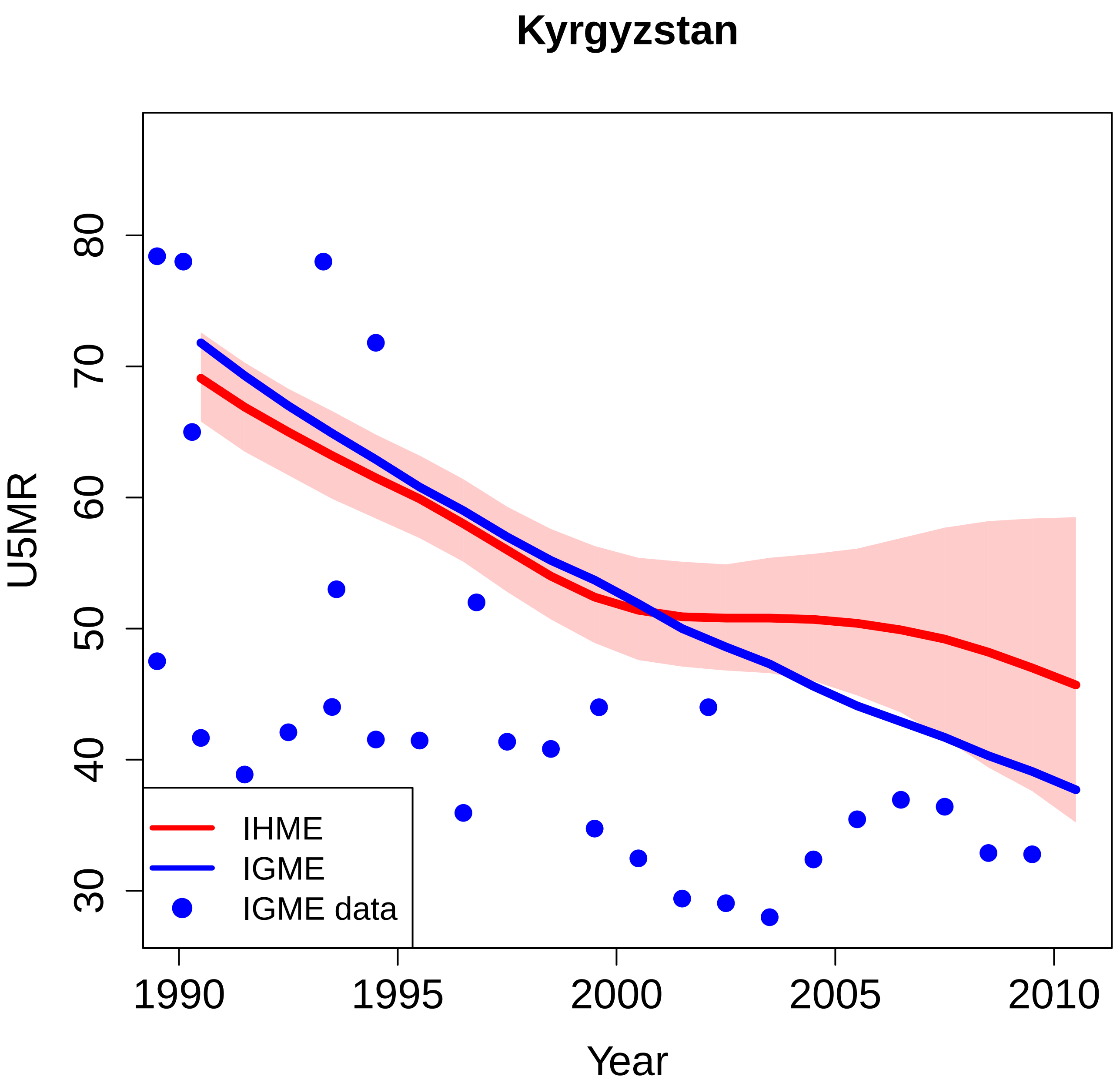

Kyrgyzstan
U5MR
30
40
50
60
70
80
1990
1995
2000
2005
2010
Year
IHME
IGME
IGME data

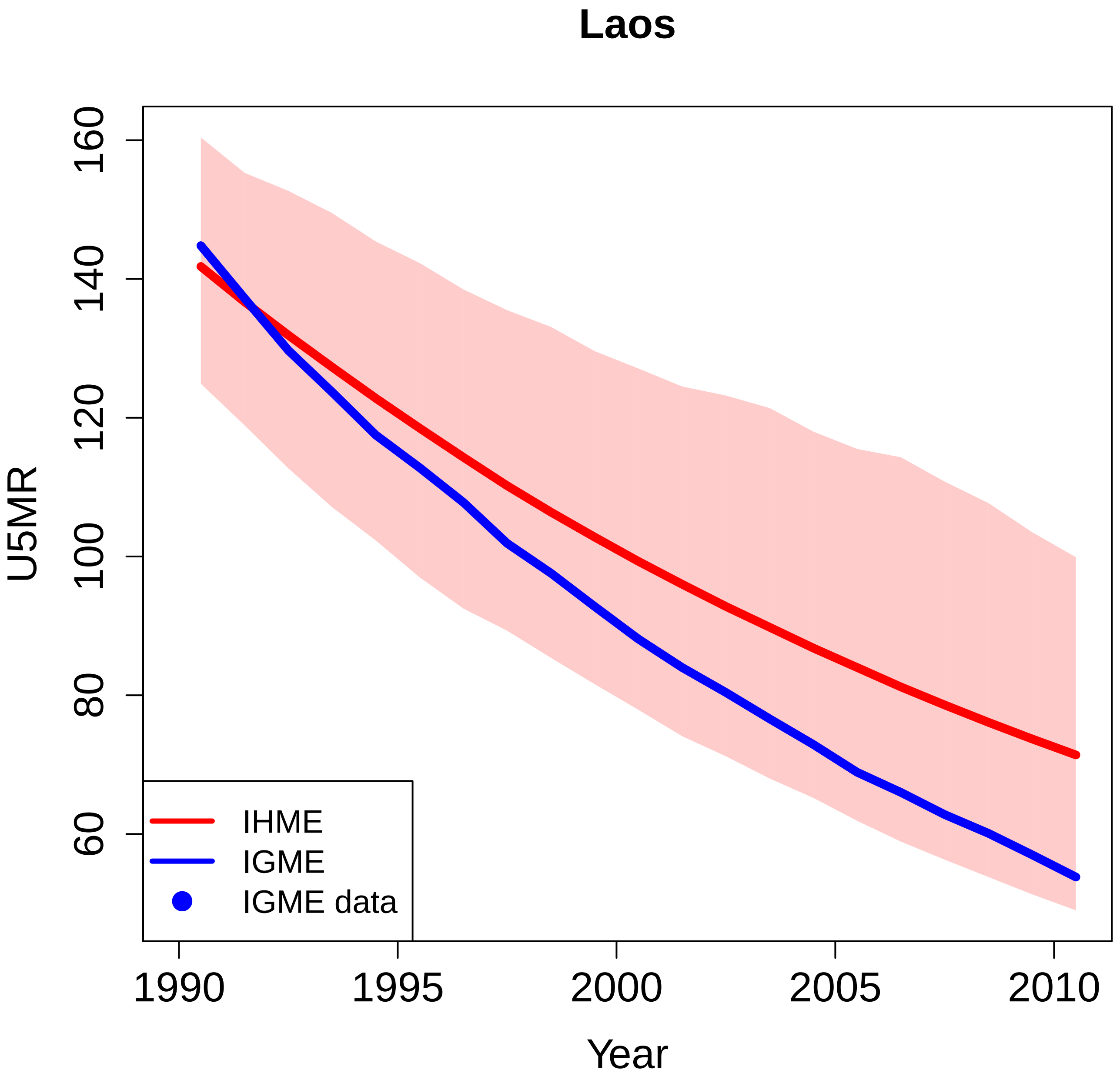

Laos
U5MR
160
140
120
100
80
60
1990
1995
2000
2005
2010
Year
IHME
IGME
IGME data

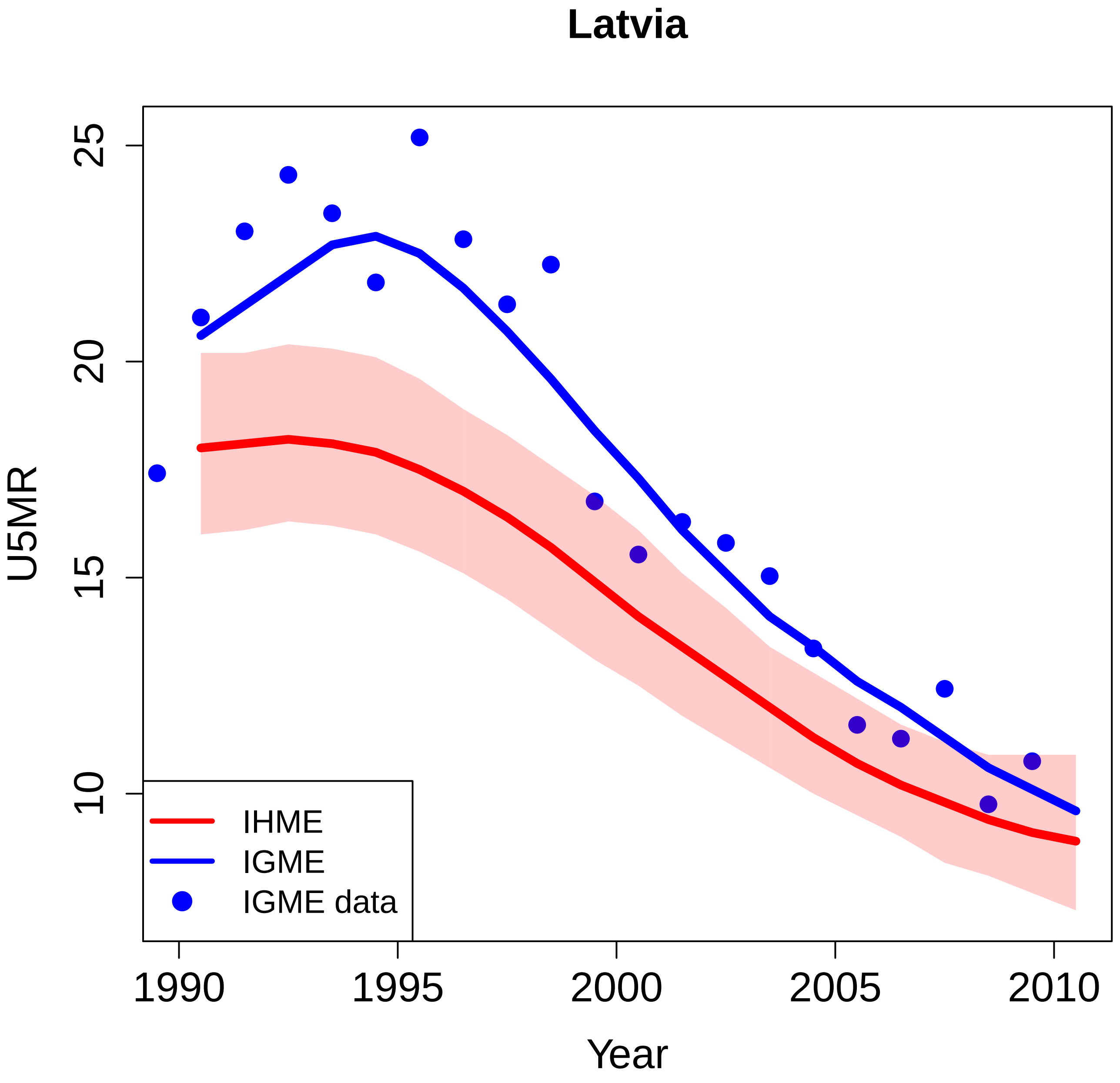

Latvia
U5MR
Year
25
20
15
10
1990
1995
2000
2005
2010
IHME
IGME
IGME data

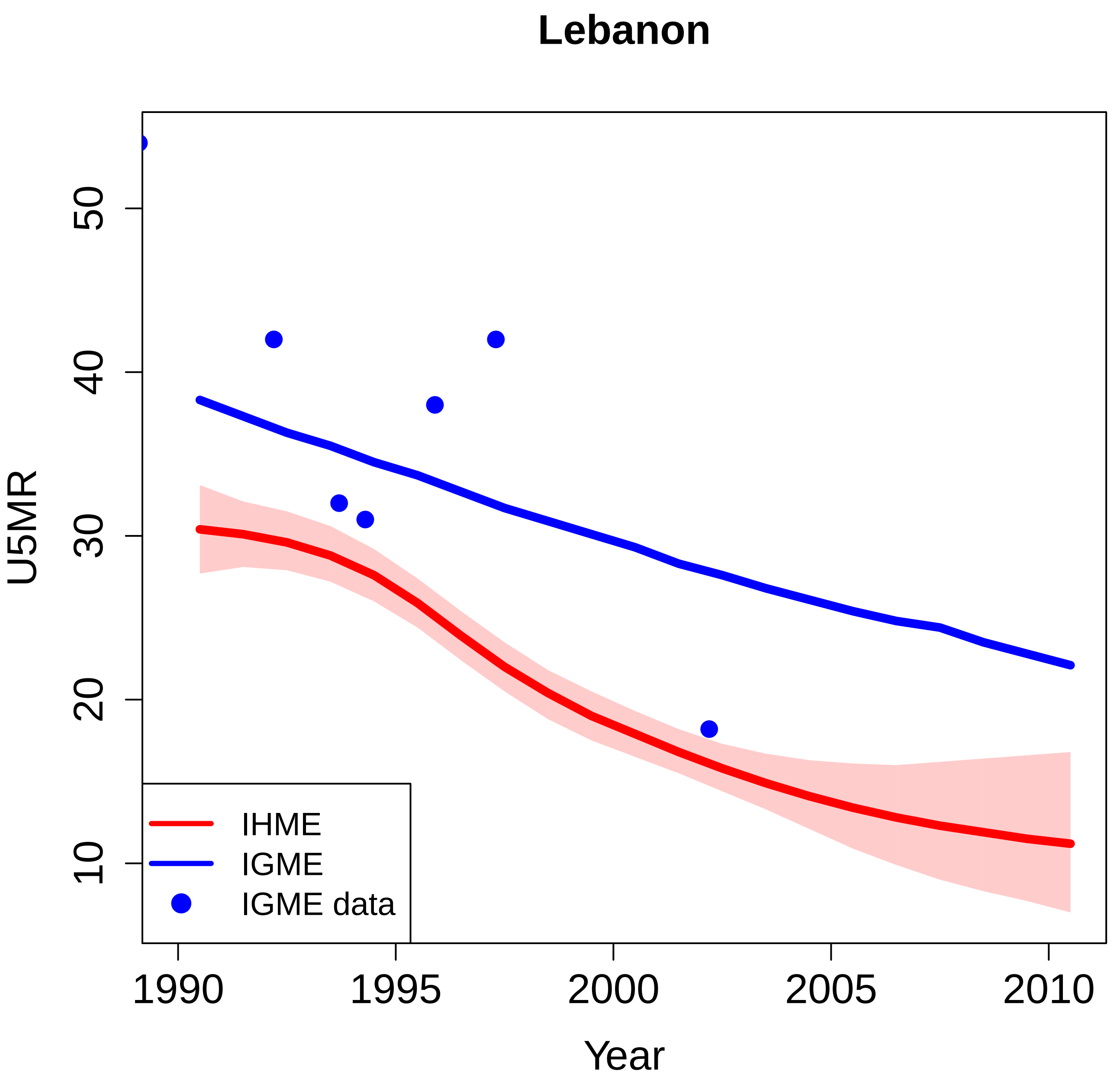
Lebanon
U5MR
10
20
30
40
50
1990
1995
2000
2005
2010
Year
IHME
IGME
IGME data

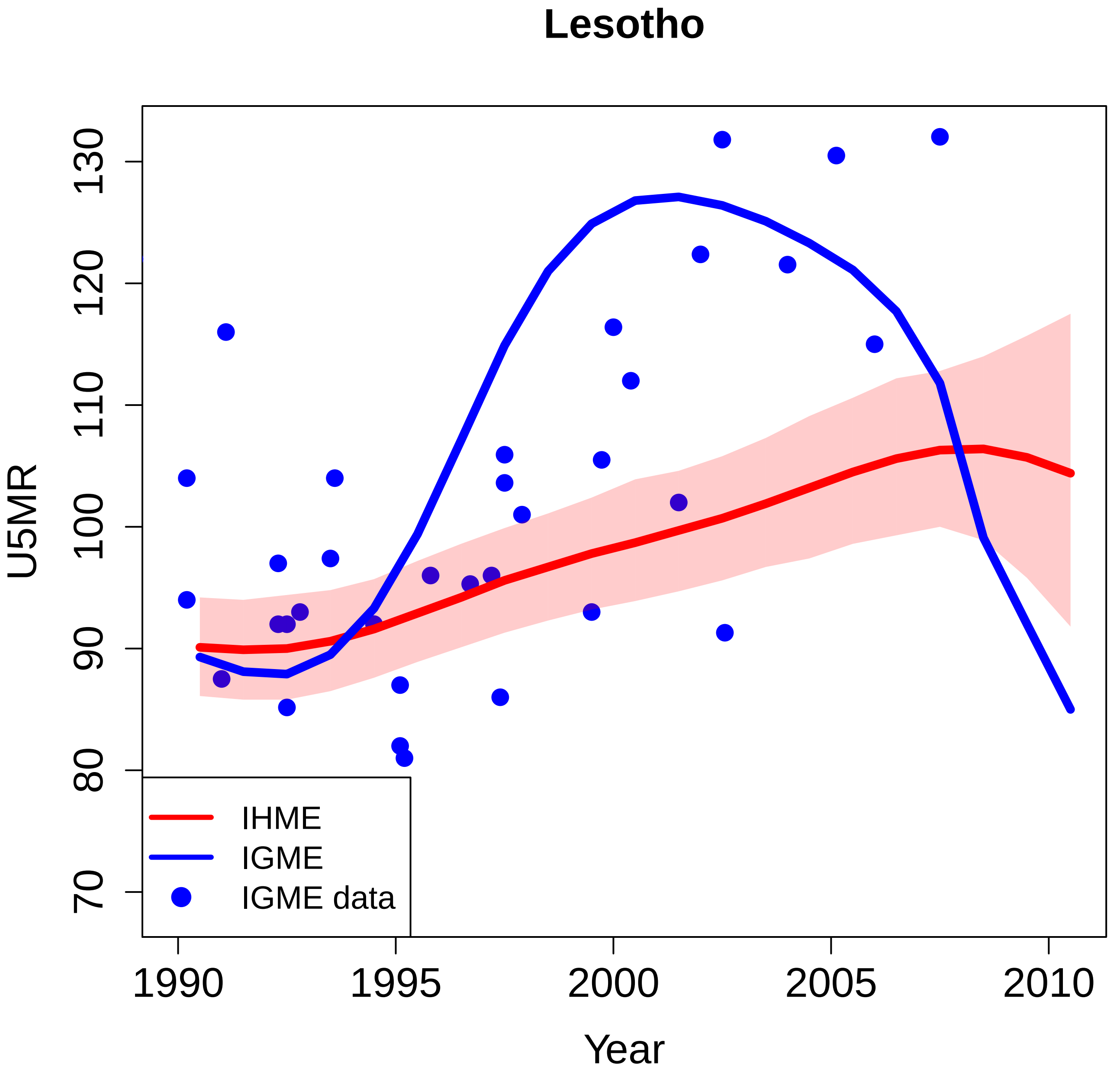

Lesotho
U5MR
Year
70
80
90
100
110
120
130
1990
1995
2000
2005
2010
IHME
IGME
IGME data

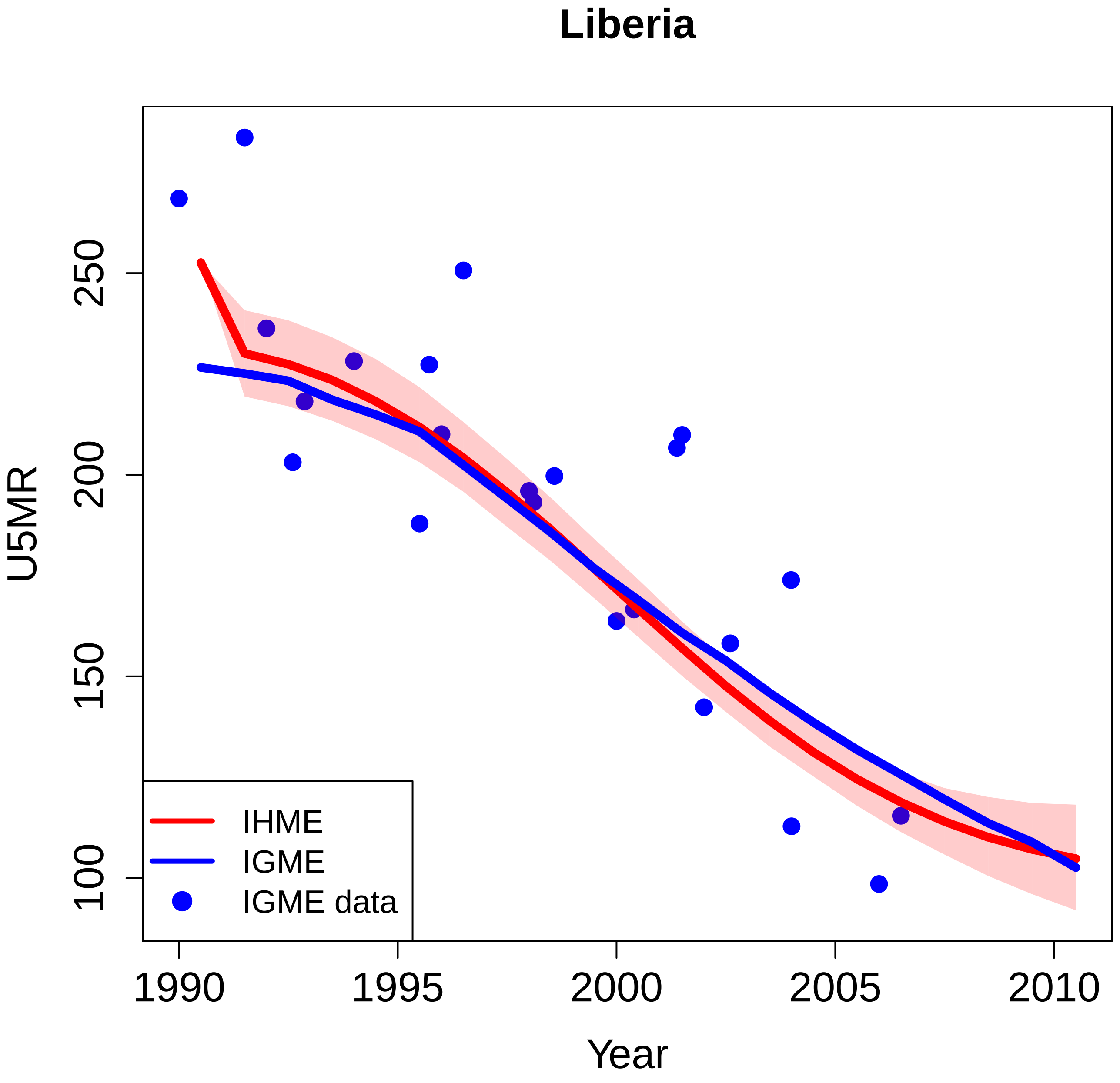

Liberia
U5MR
100
150
200
250
1990
1995
2000
2005
2010
Year
IHME
IGME
IGME data

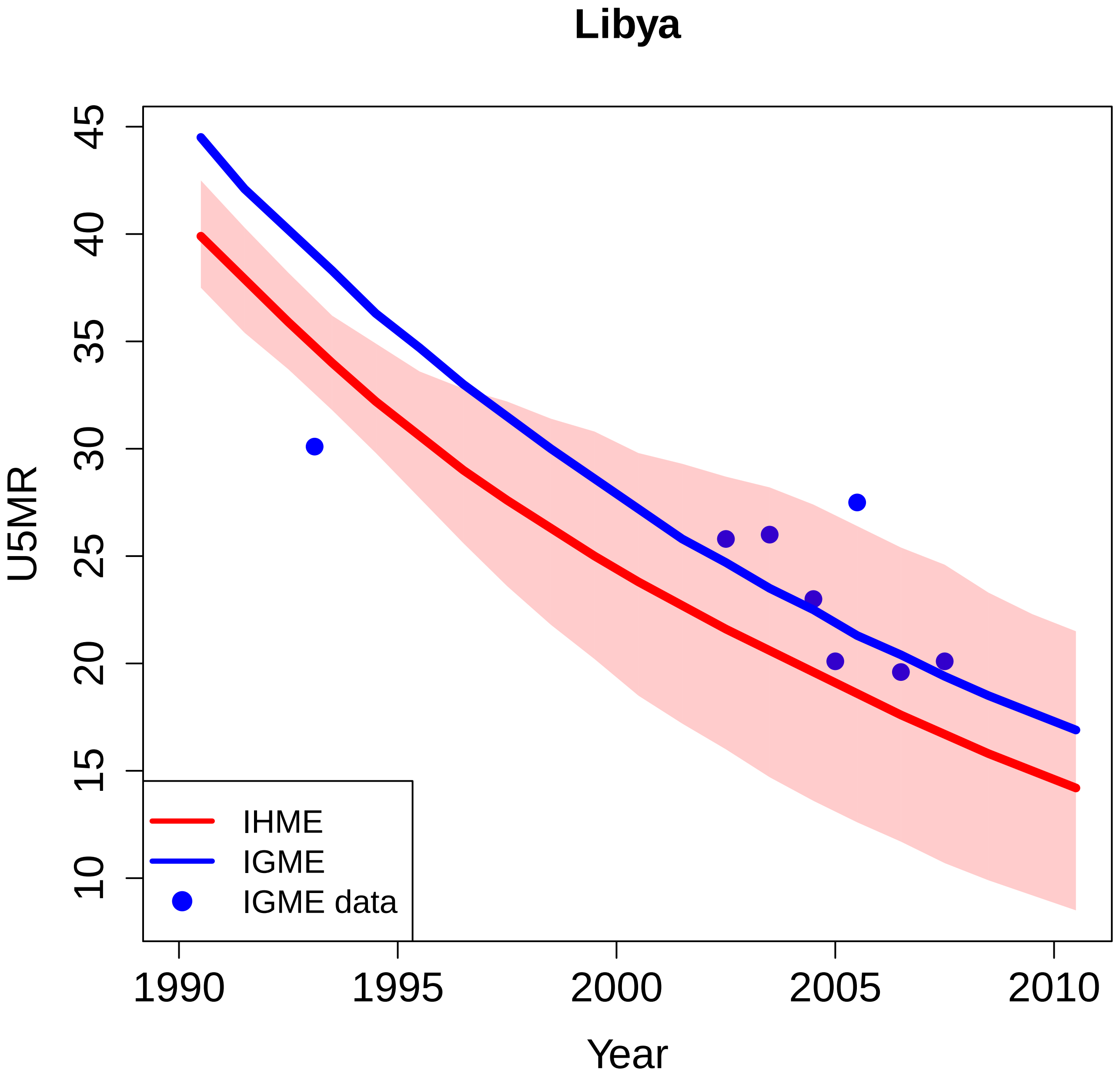

Libya
U5MR
Year
45
40
35
30
25
20
15
10
1990
1995
2000
2005
2010
IHME
IGME
IGME data

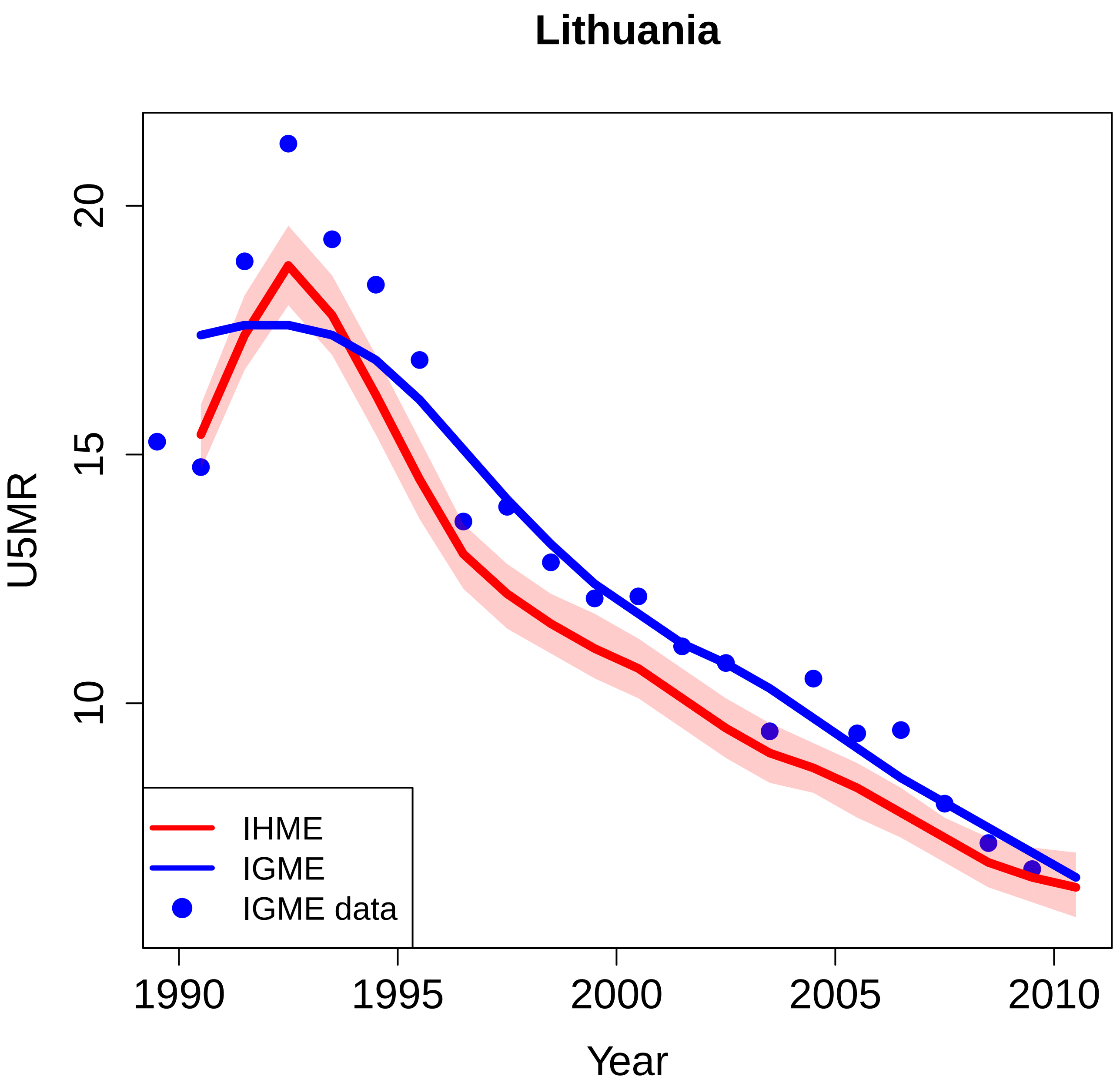
Lithuania
U5MR
20
15
10
1990
1995
2000
2005
2010
Year
IHME
IGME
IGME data

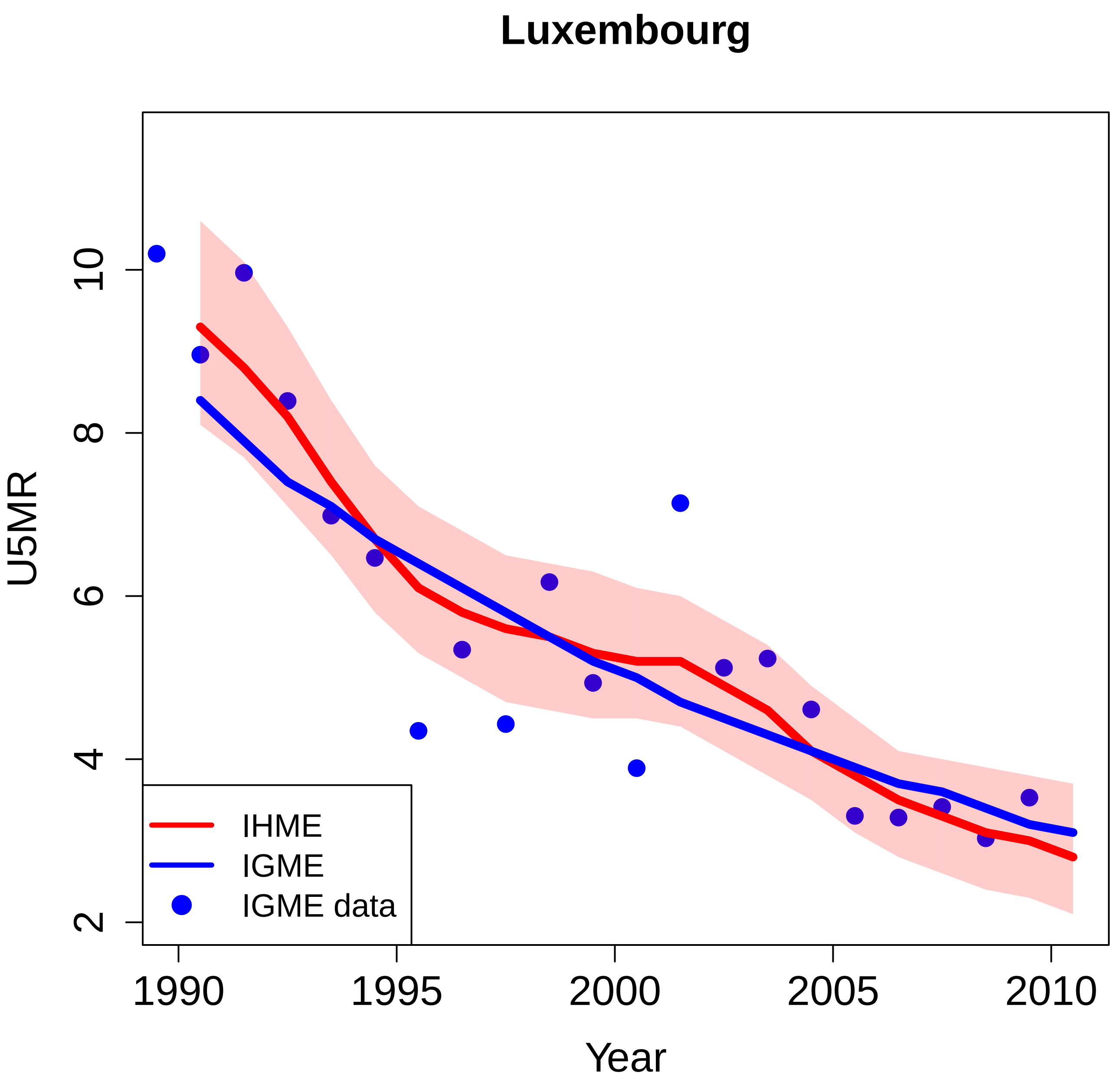
Luxembourg
U5MR
10
8
6
4
2
1990
1995
2000
2005
2010
Year
IHME
IGME
IGME data

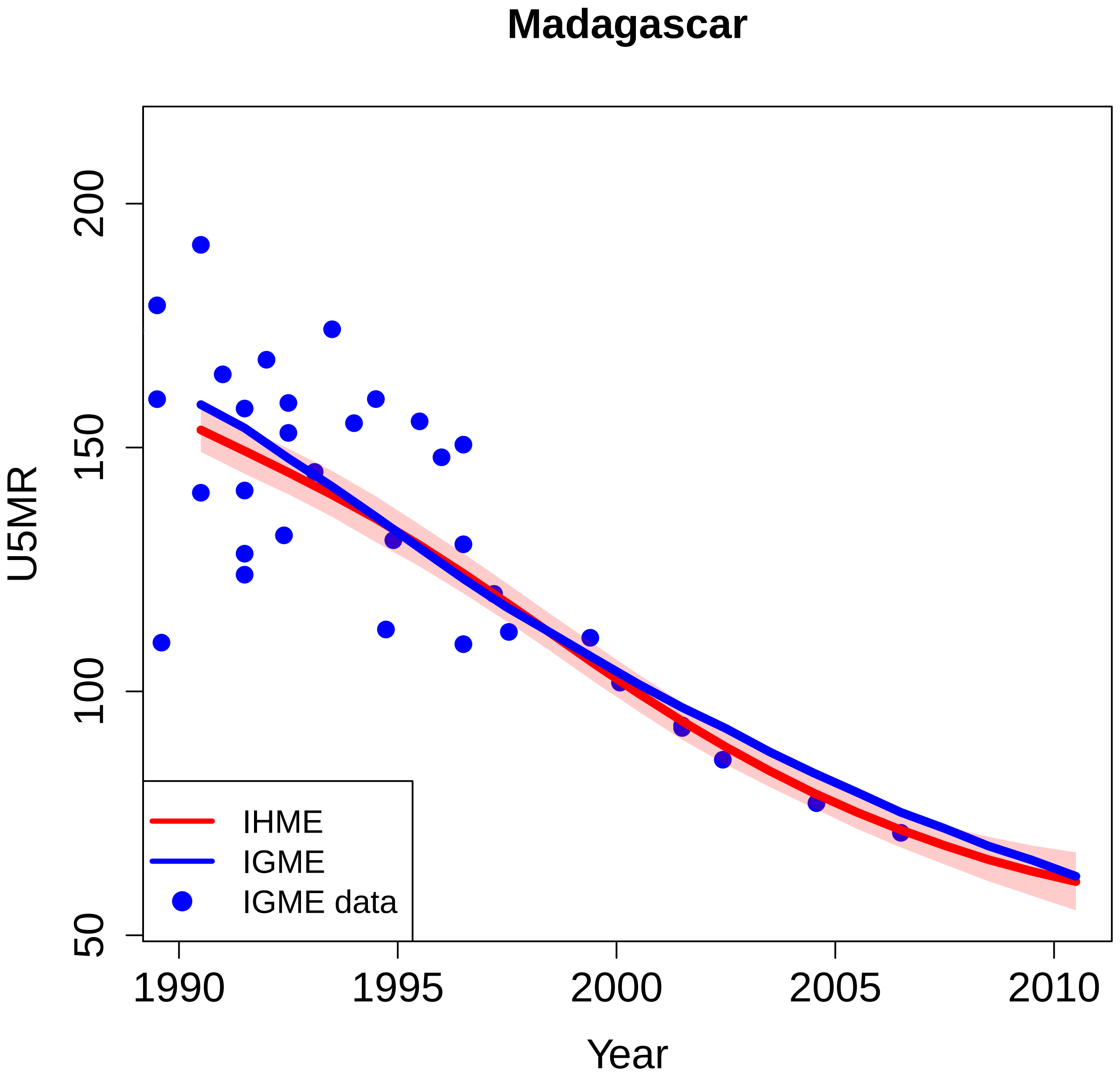

Madagascar
U5MR
Year
200
150
100
50
1990
1995
2000
2005
2010
IHME
IGME
IGME data

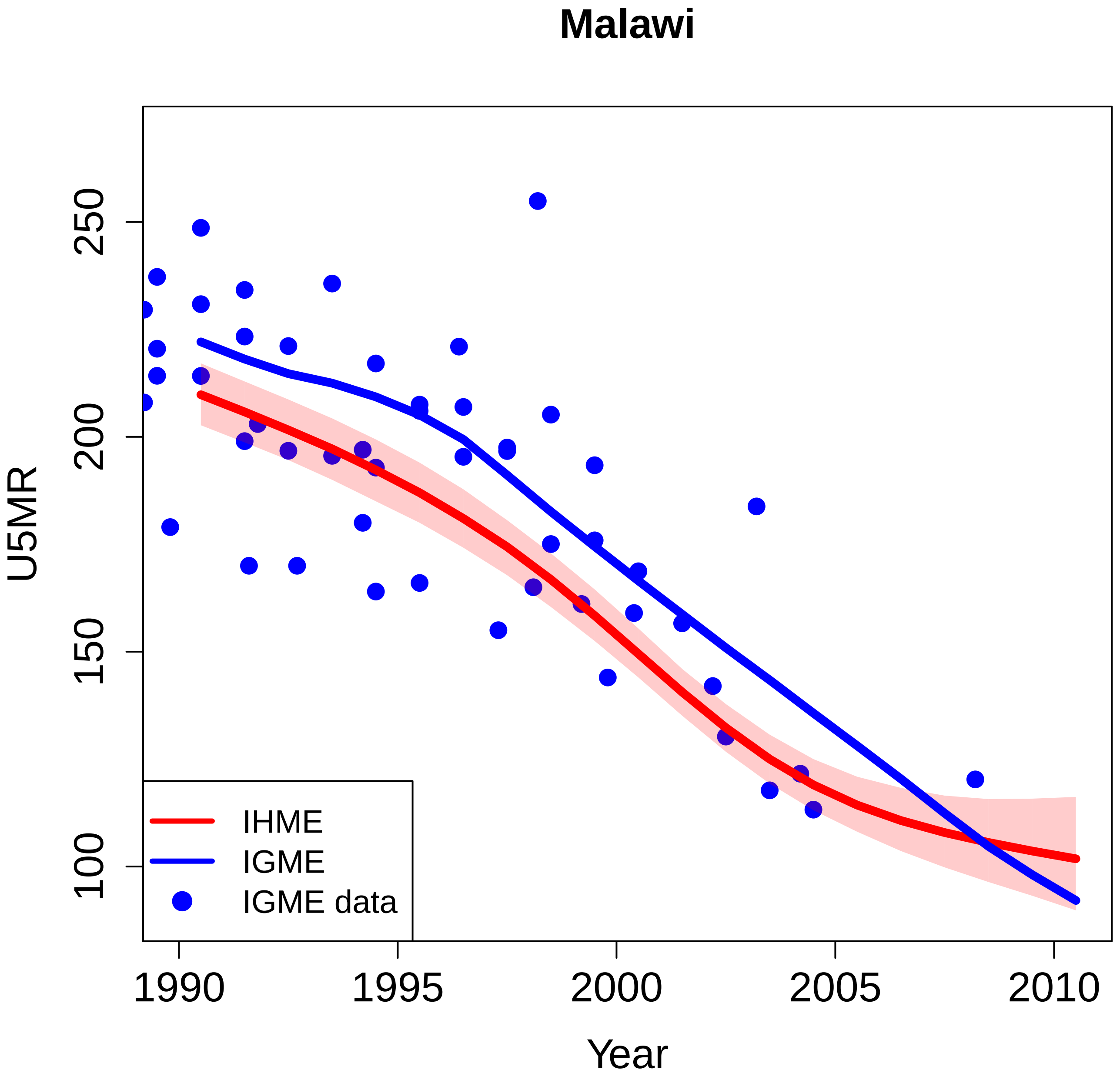
Malawi
U5MR
250
200
150
100
1990
1995
2000
2005
2010
Year
IHME
IGME
IGME data

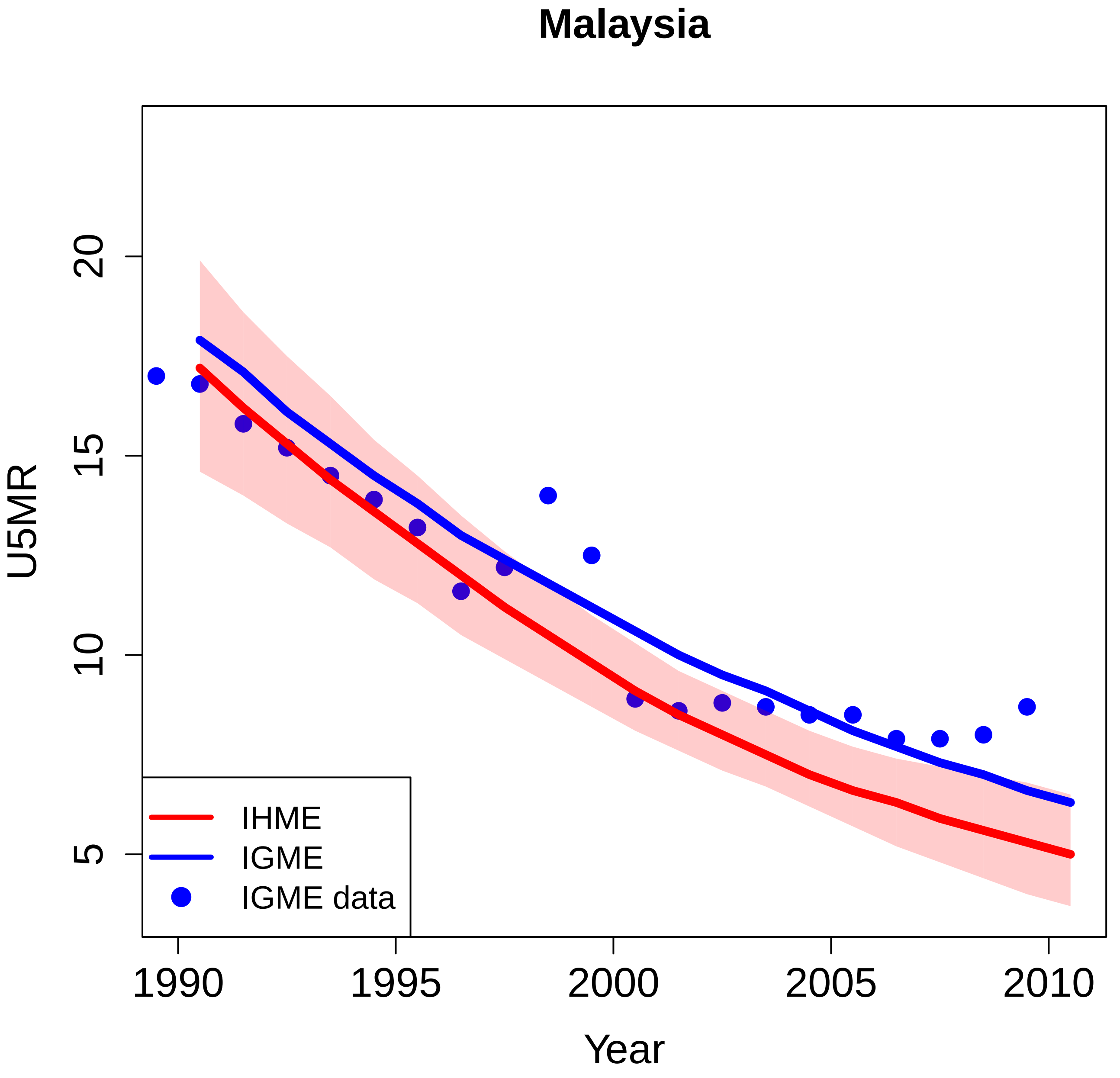
Malaysia
U5MR
20
15
10
5
1990
1995
2000
2005
2010
Year
IHME
IGME
IGME data

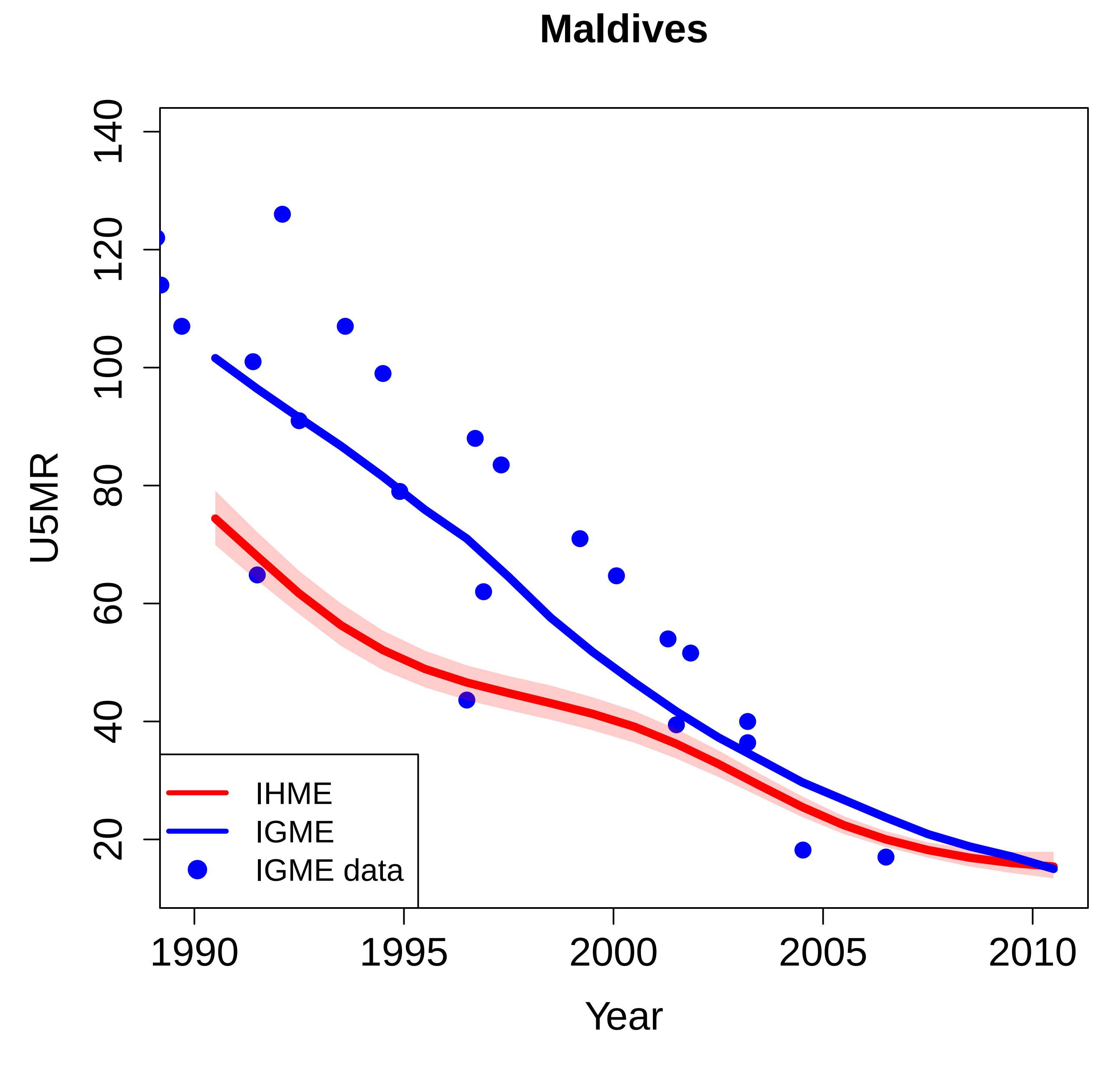

Maldives
U5MR
140
120
100
80
60
40
20
1990
1995
2000
2005
2010
Year
IHME
IGME
IGME data

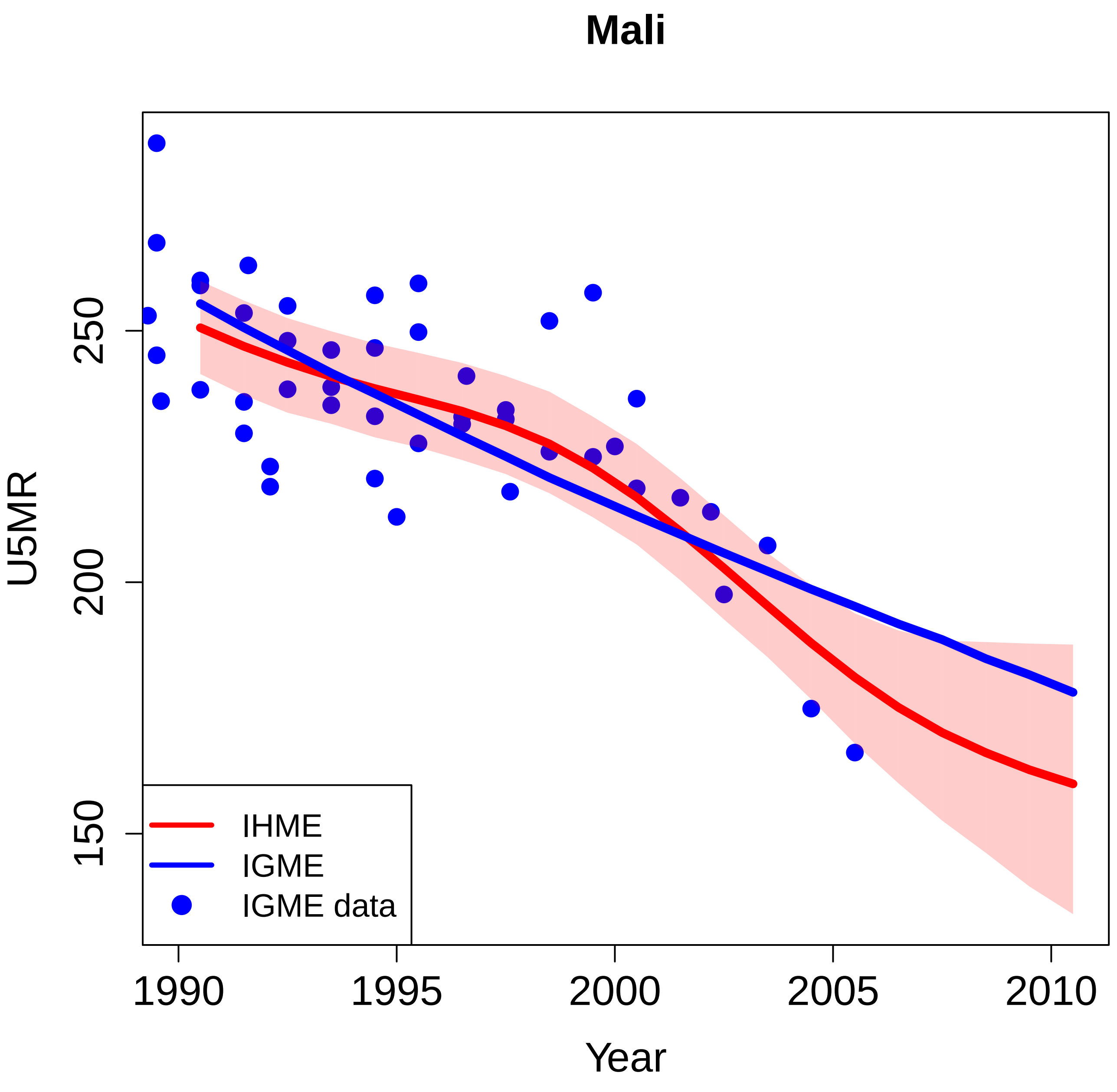

Mali
U5MR
250
200
150
1990
1995
2000
2005
2010
Year
IHME
IGME
IGME data

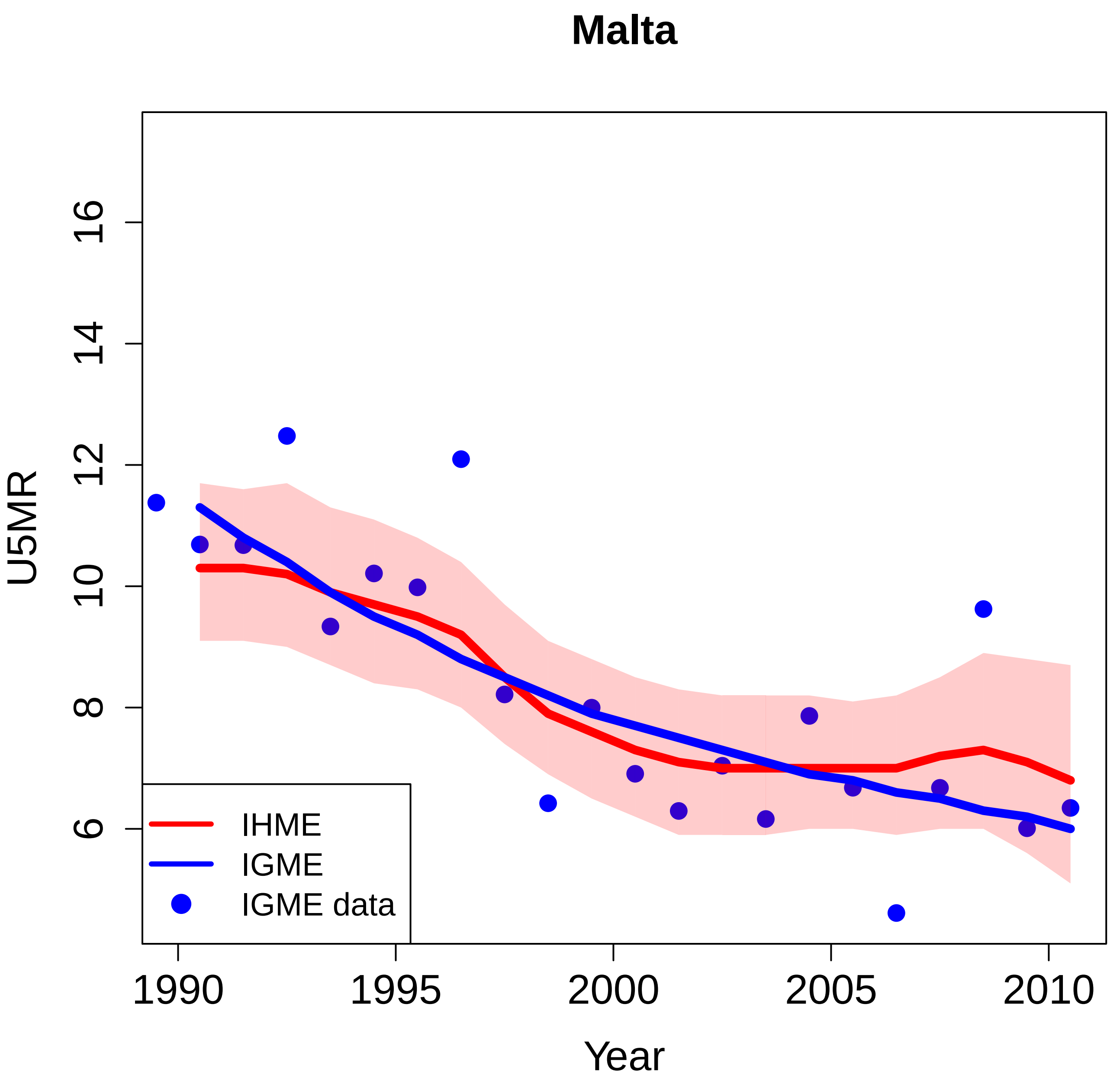
Malta
U5MR
16
14
12
10
8
6
1990
1995
2000
2005
2010
Year
IHME
IGME
IGME data

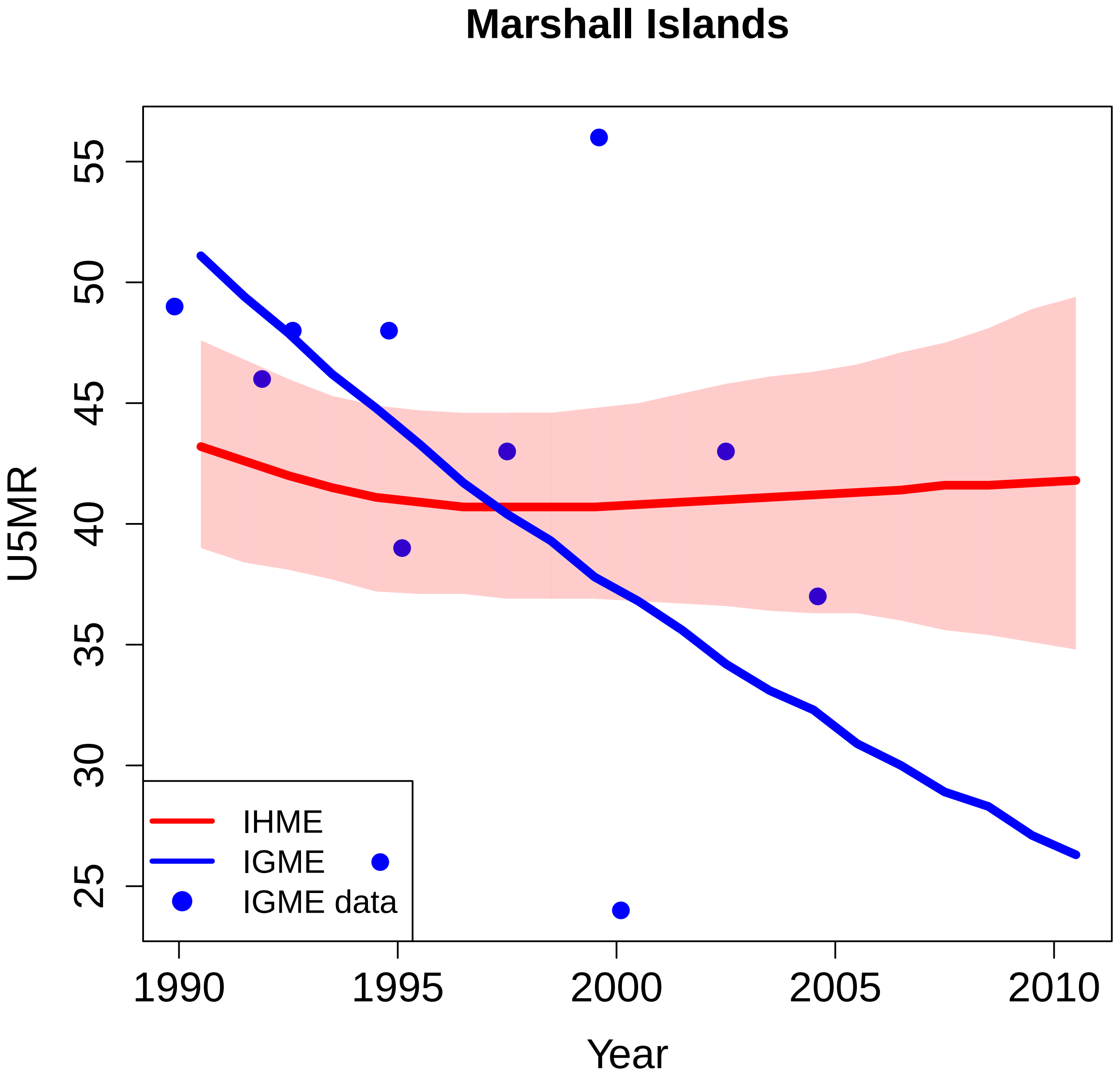
Marshall Islands
U5MR
25
30
35
40
45
50
55
1990
1995
2000
2005
2010
Year
IHME
IGME
IGME data

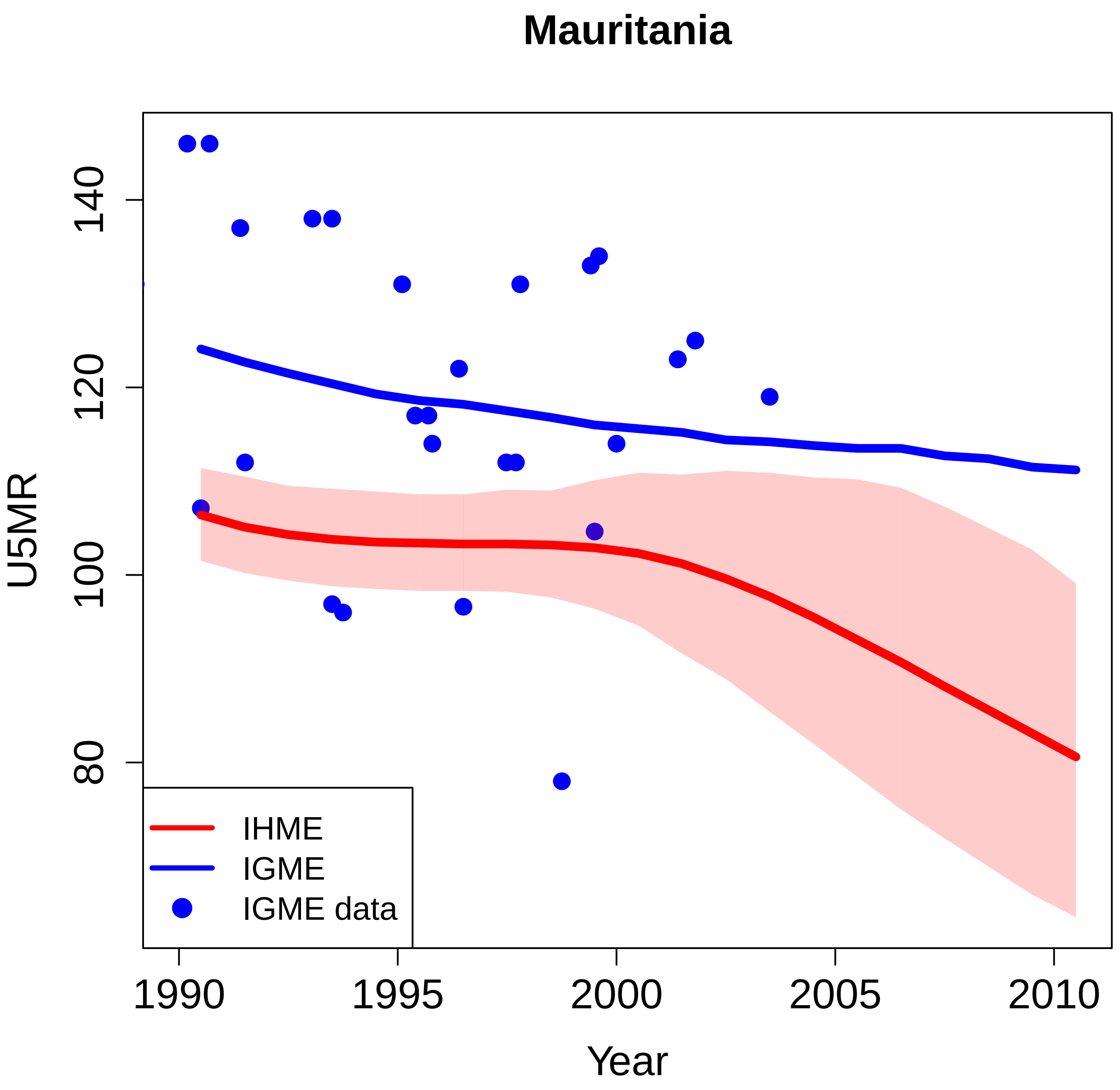
Mauritania
U5MR
80
100
120
140
1990
1995
2000
2005
2010
Year
IHME
IGME
IGME data

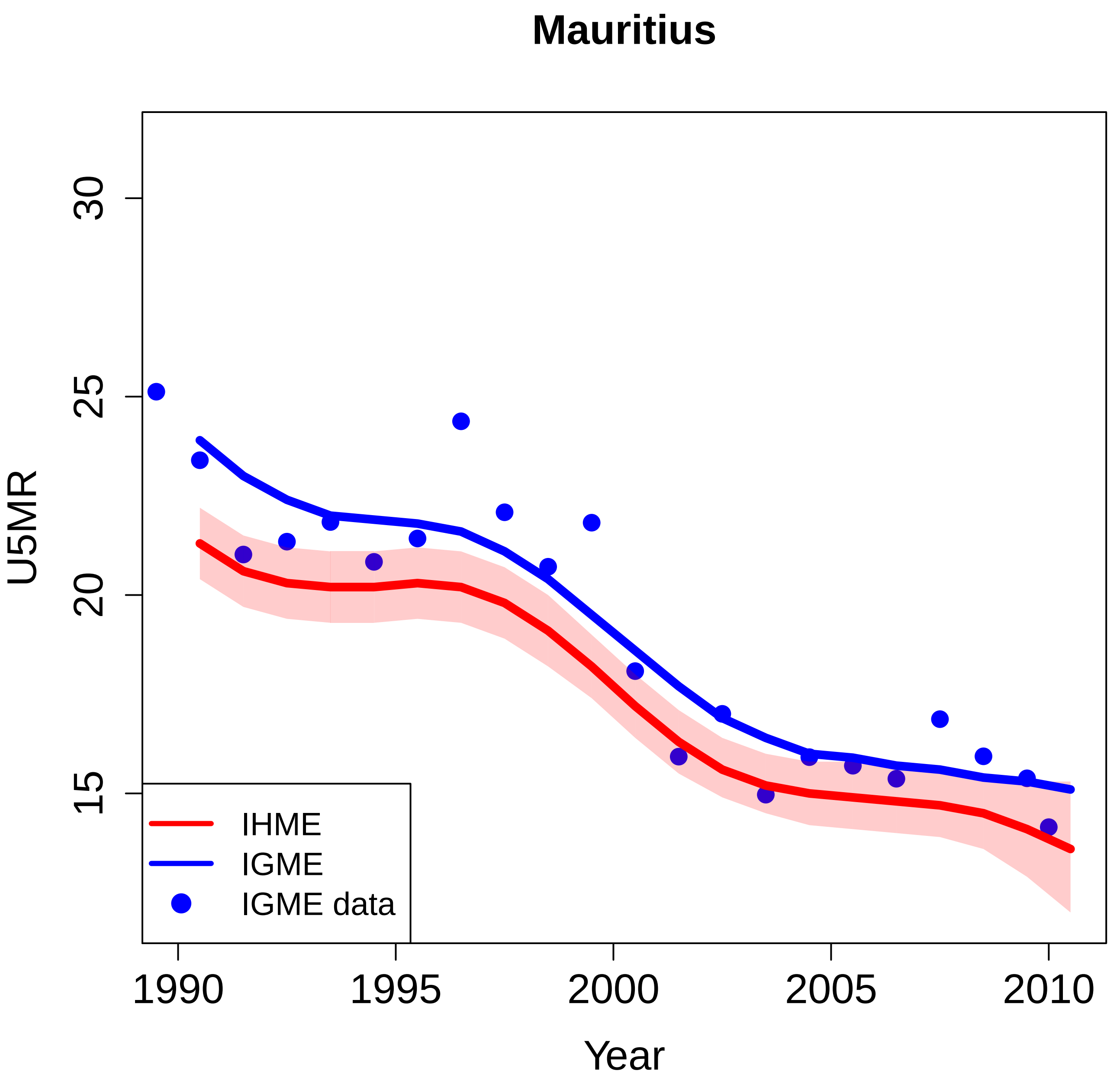

Mauritius
U5MR
30
25
20
15
1990
1995
2000
2005
2010
Year
IHME
IGME
IGME data

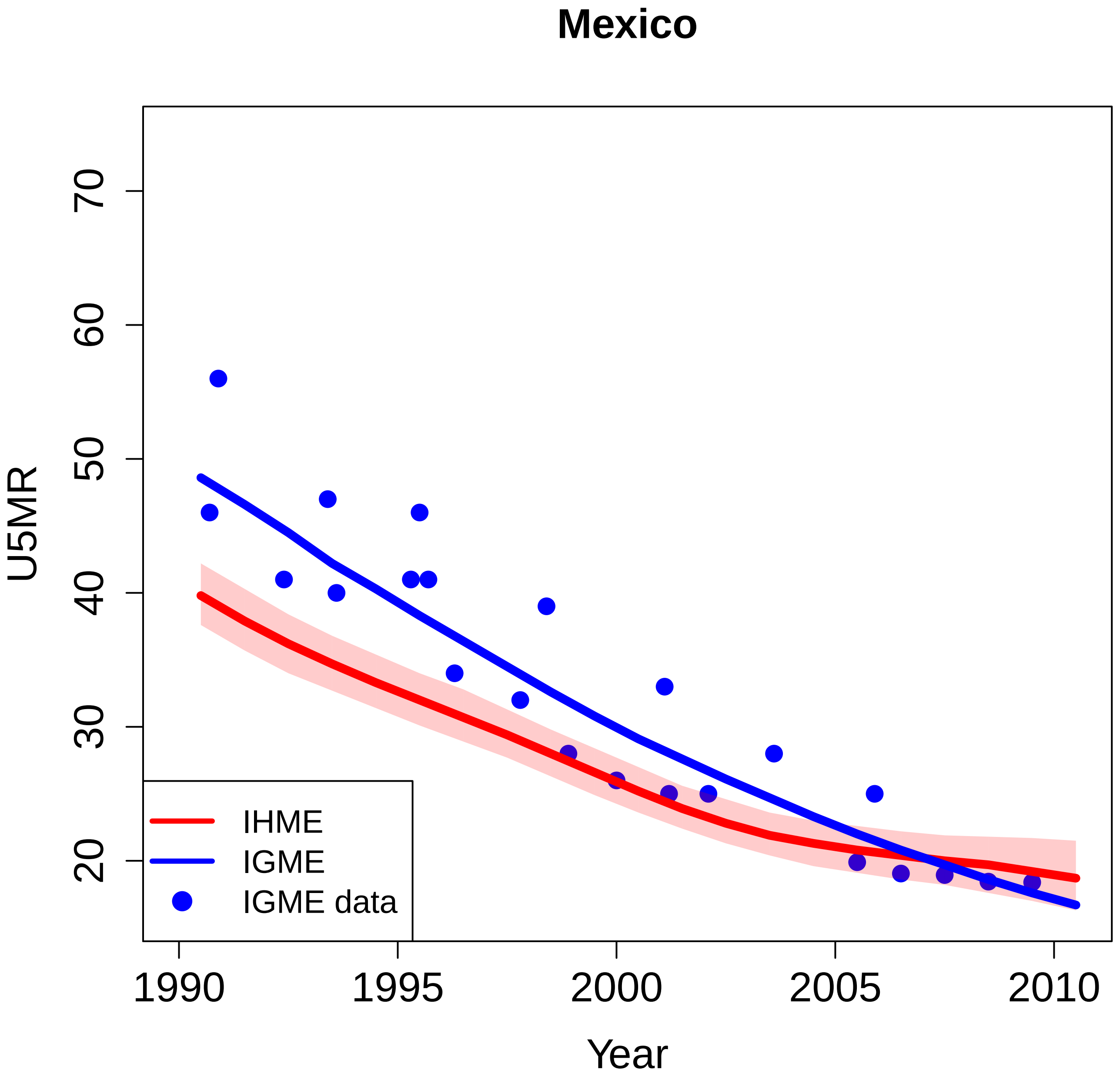
Mexico
U5MR
70
60
50
40
30
20
1990
1995
2000
2005
2010
Year
IHME
IGME
IGME data

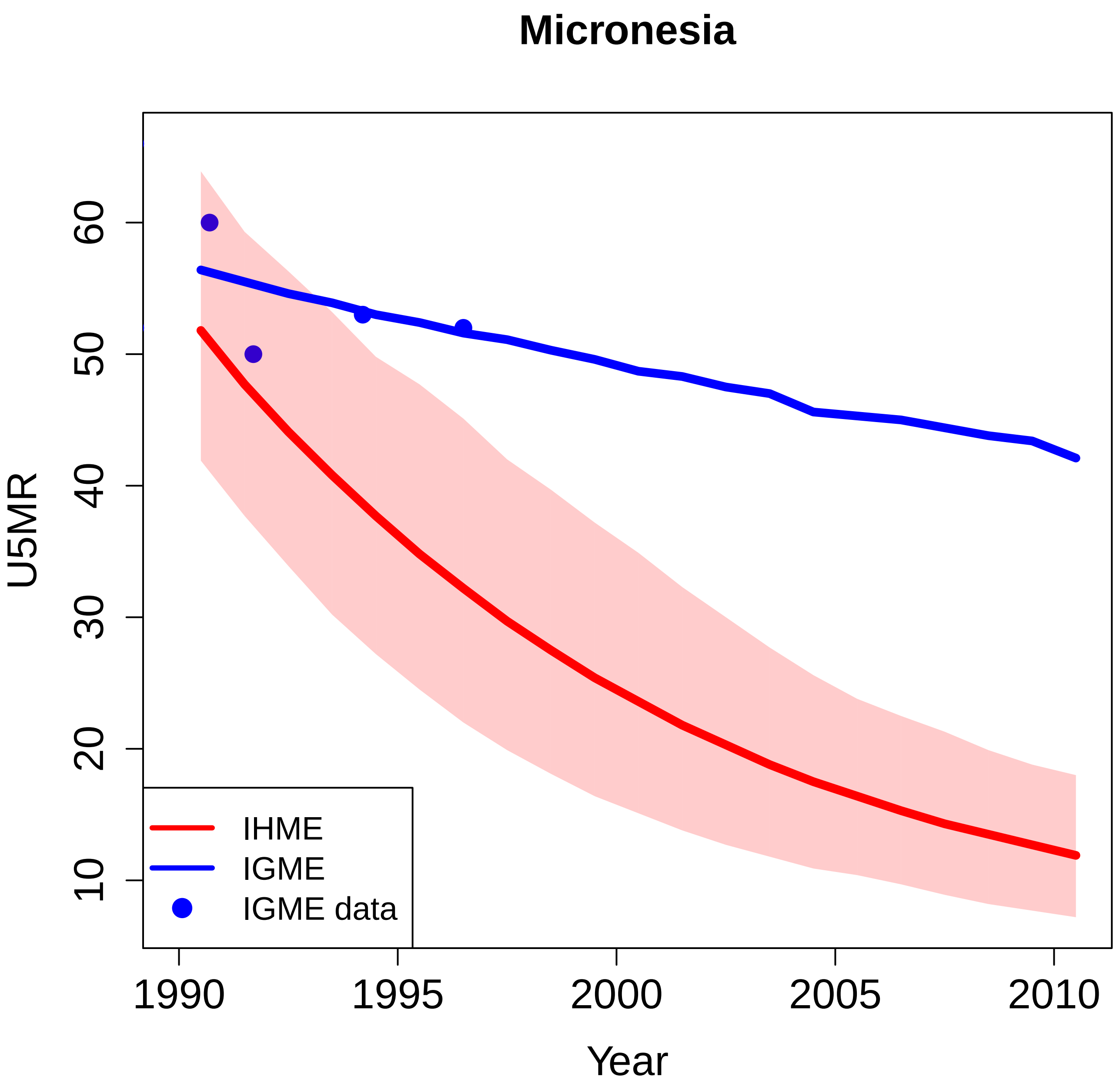

Micronesia
U5MR
60
50
40
30
20
10
1990
1995
2000
2005
2010
Year
IHME
IGME
IGME data

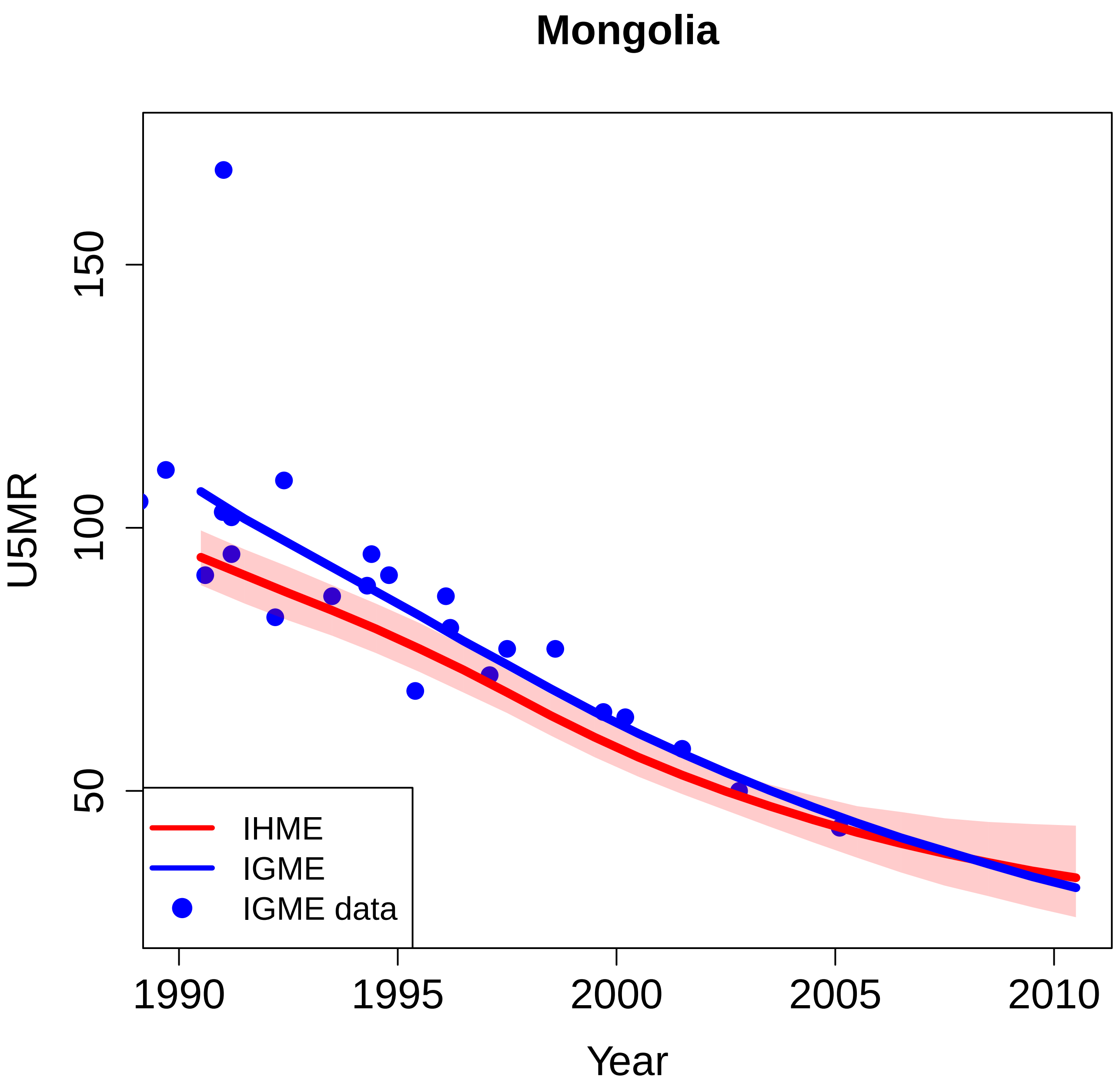

Mongolia
U5MR
150
100
50
1990
1995
2000
2005
2010
Year
IHME
IGME
IGME data

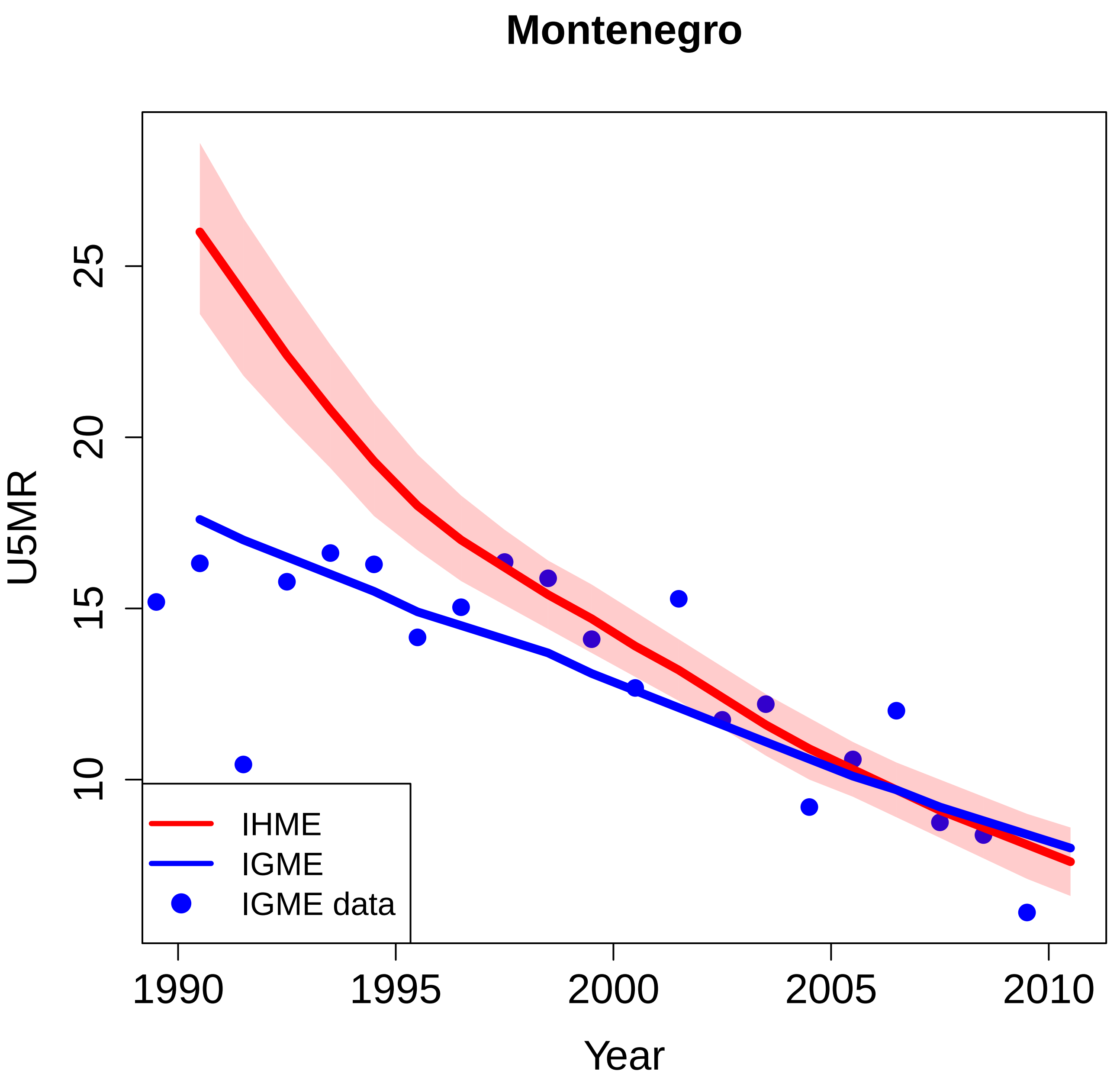

Montenegro
U5MR
Year
10
15
20
25
1990
1995
2000
2005
2010
IHME
IGME
IGME data

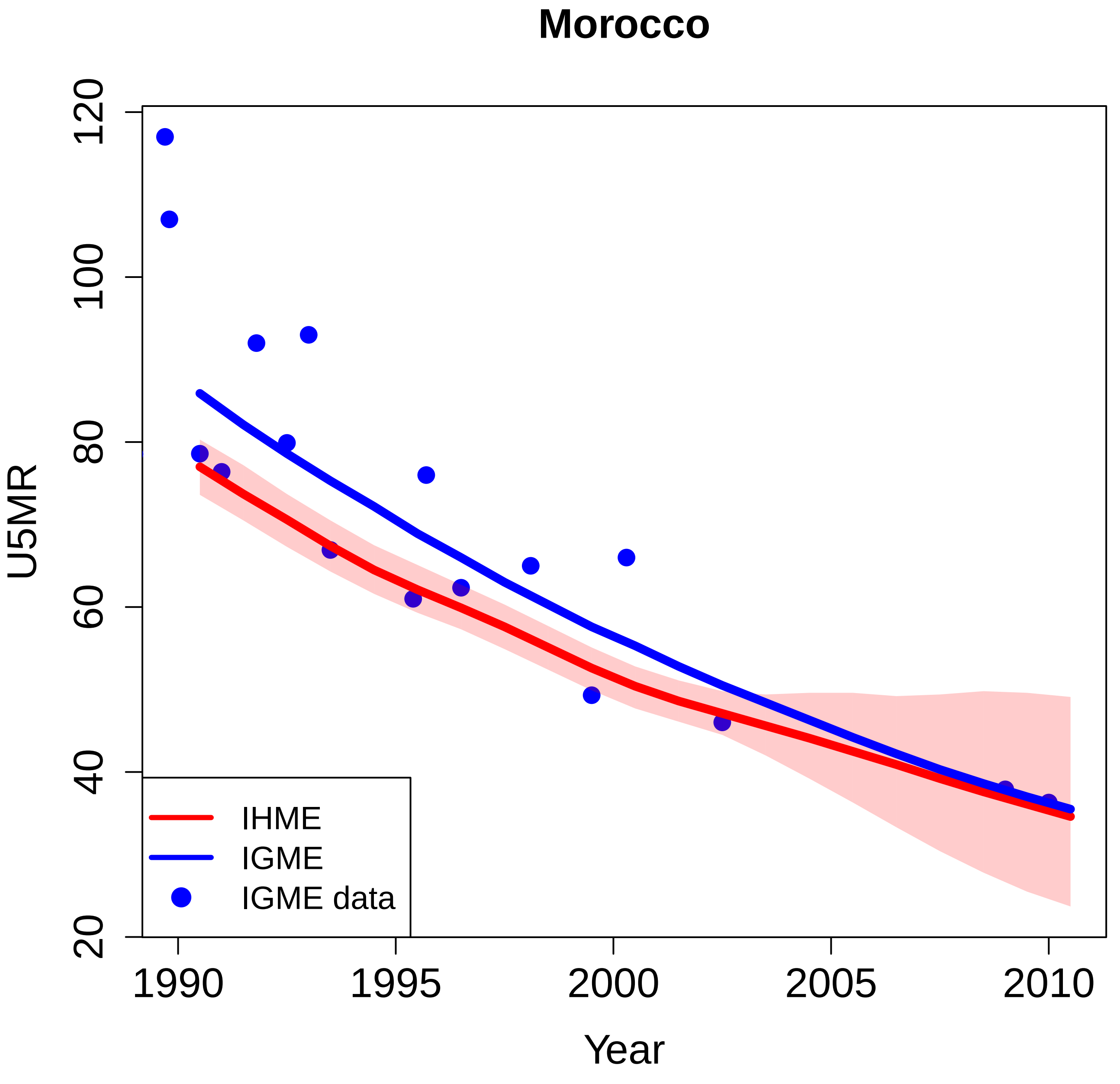
Morocco
U5MR
Year
120
100
80
60
40
20
1990
1995
2000
2005
2010
IHME
IGME
IGME data

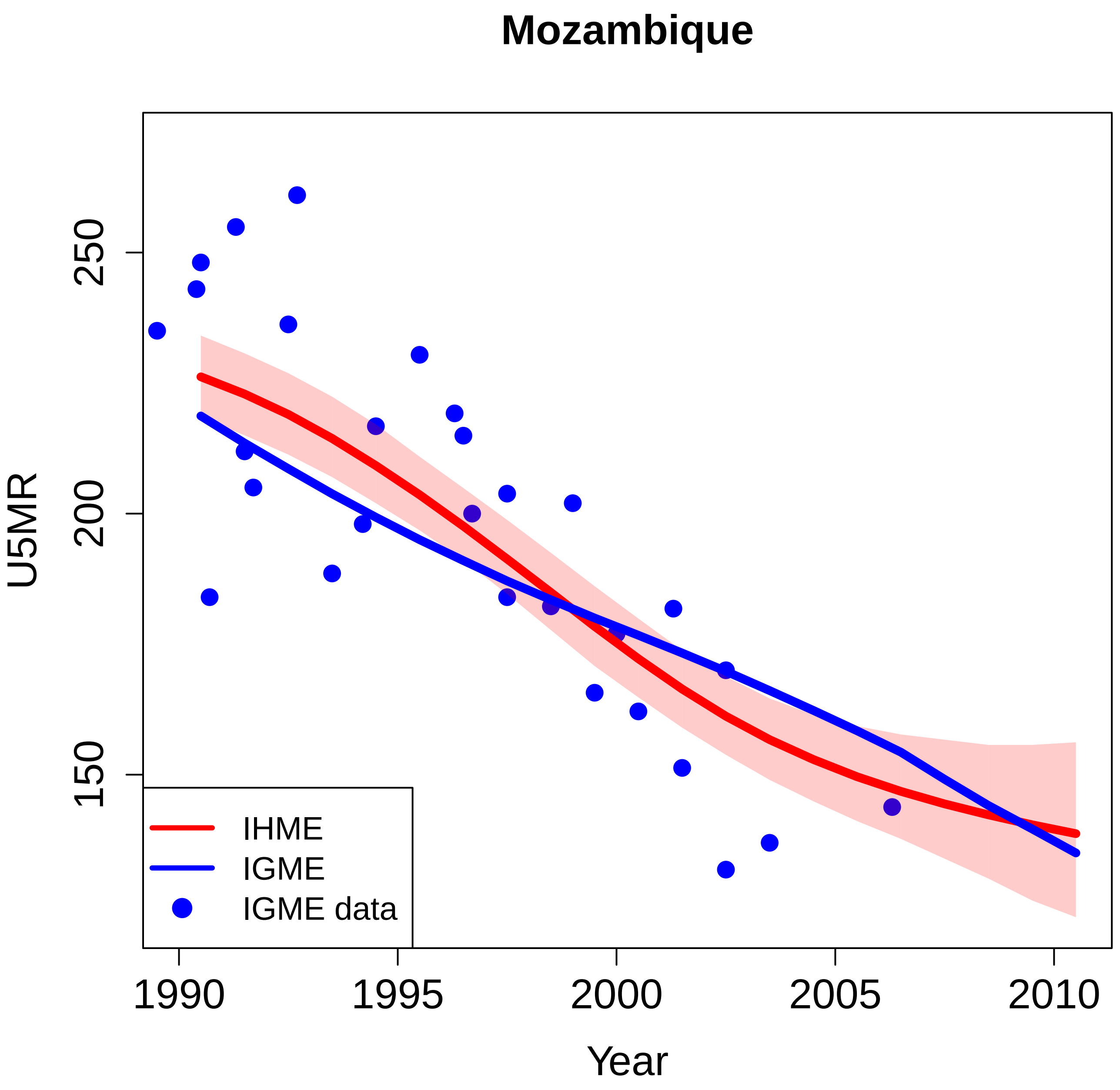
Mozambique
U5MR
250
200
150
1990
1995
2000
2005
2010
Year
IHME
IGME
IGME data

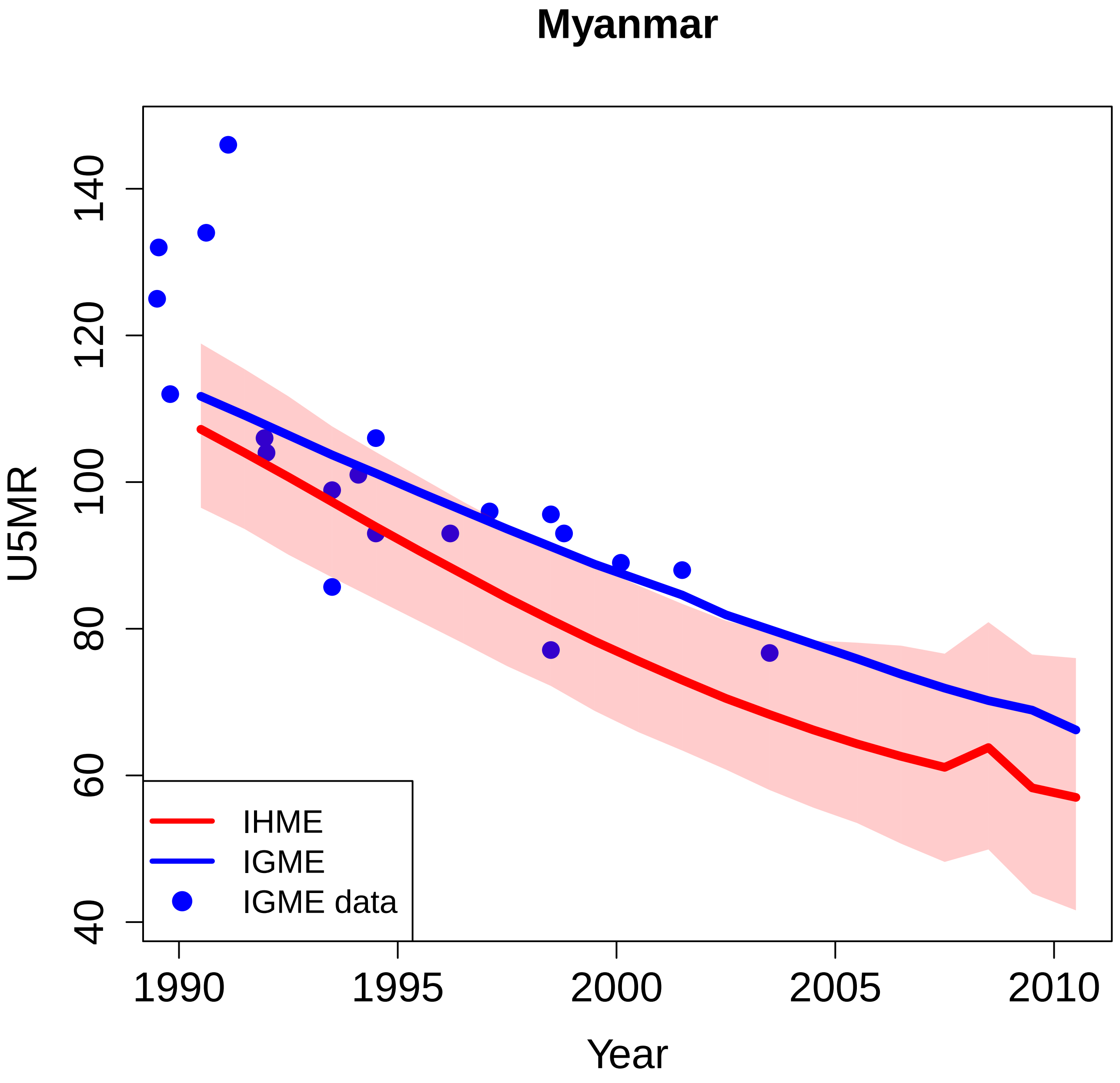

Myanmar
U5MR
140
120
100
80
60
40
1990
1995
2000
2005
2010
Year
IHME
IGME
IGME data

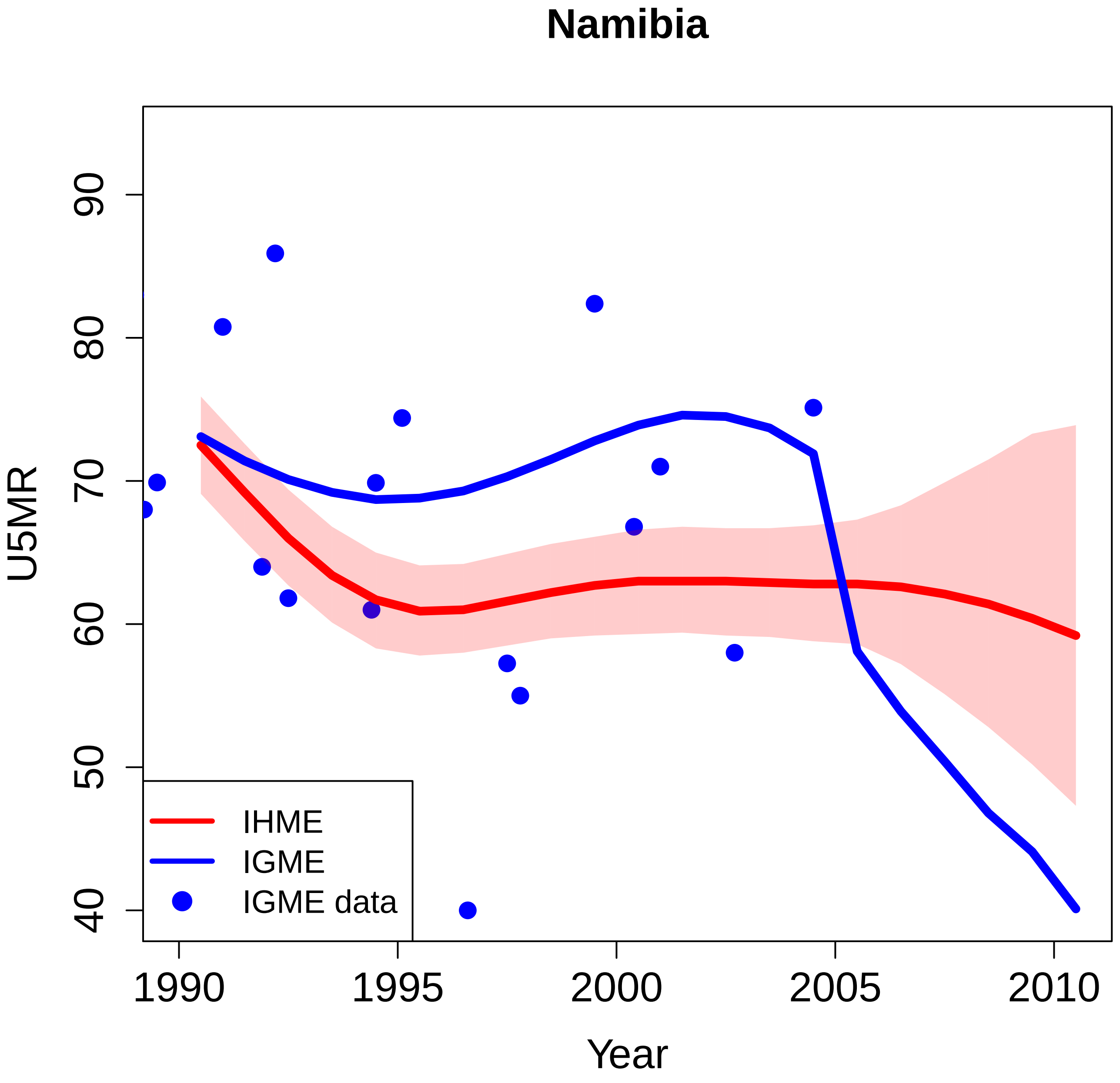
Namibia
U5MR
90
80
70
60
50
40
1990
1995
2000
2005
2010
Year
IHME
IGME
IGME data

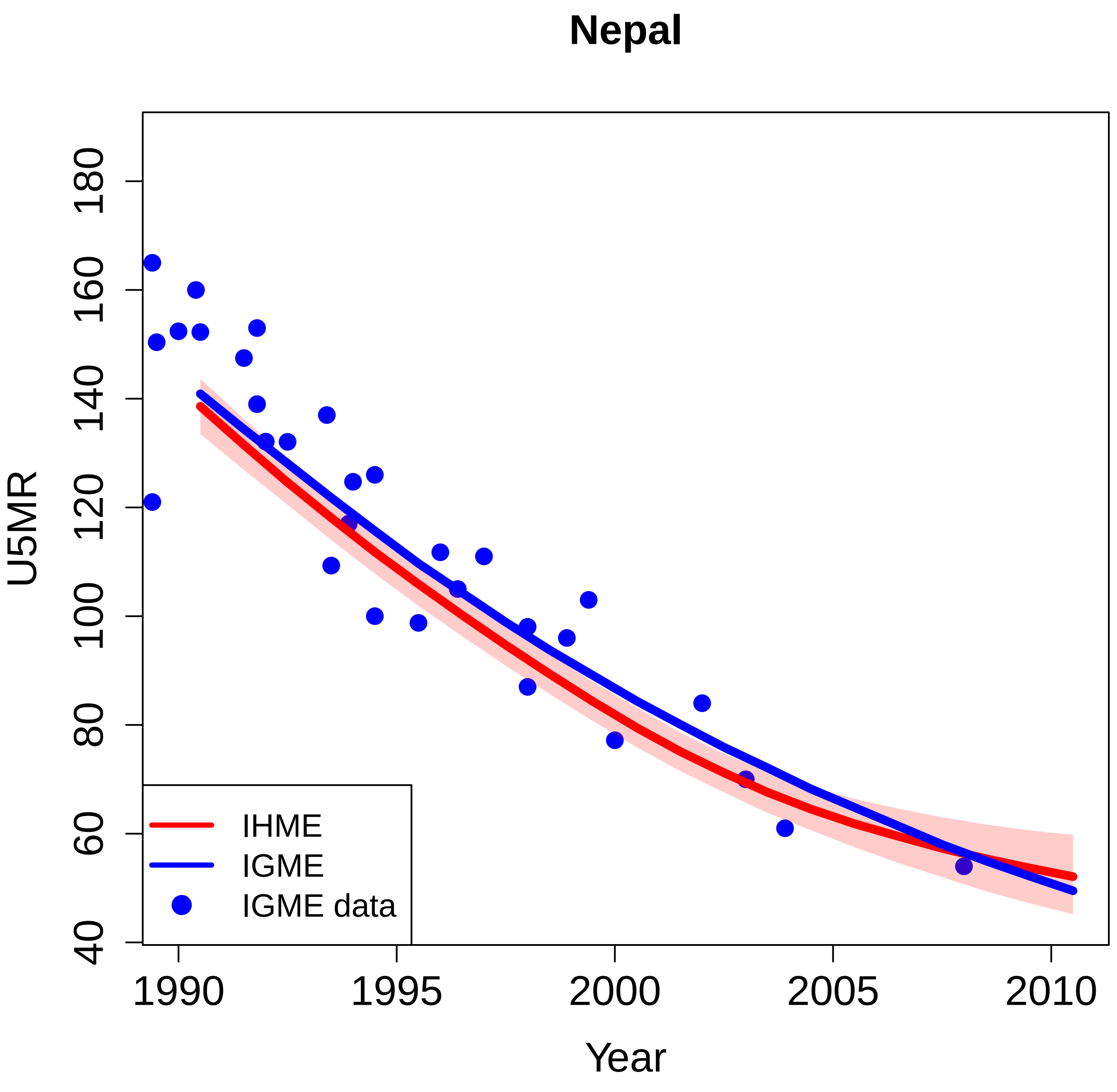
Nepal
U5MR
Year
180
160
140
120
100
80
60
40
1990
1995
2000
2005
2010
IHME
IGME
IGME data

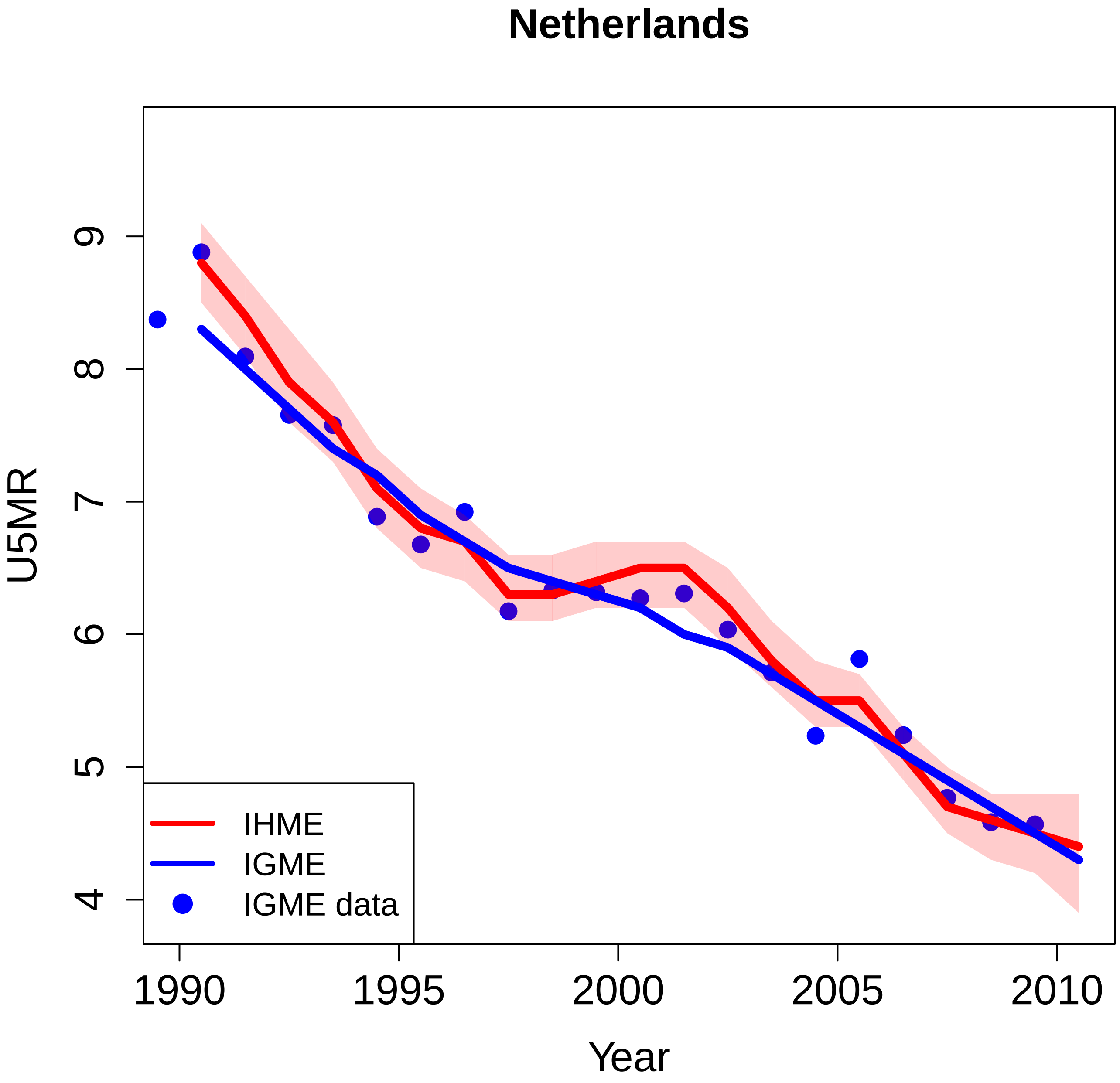

Netherlands
U5MR
Year
4
5
6
7
8
9
1990
1995
2000
2005
2010
IHME
IGME
IGME data

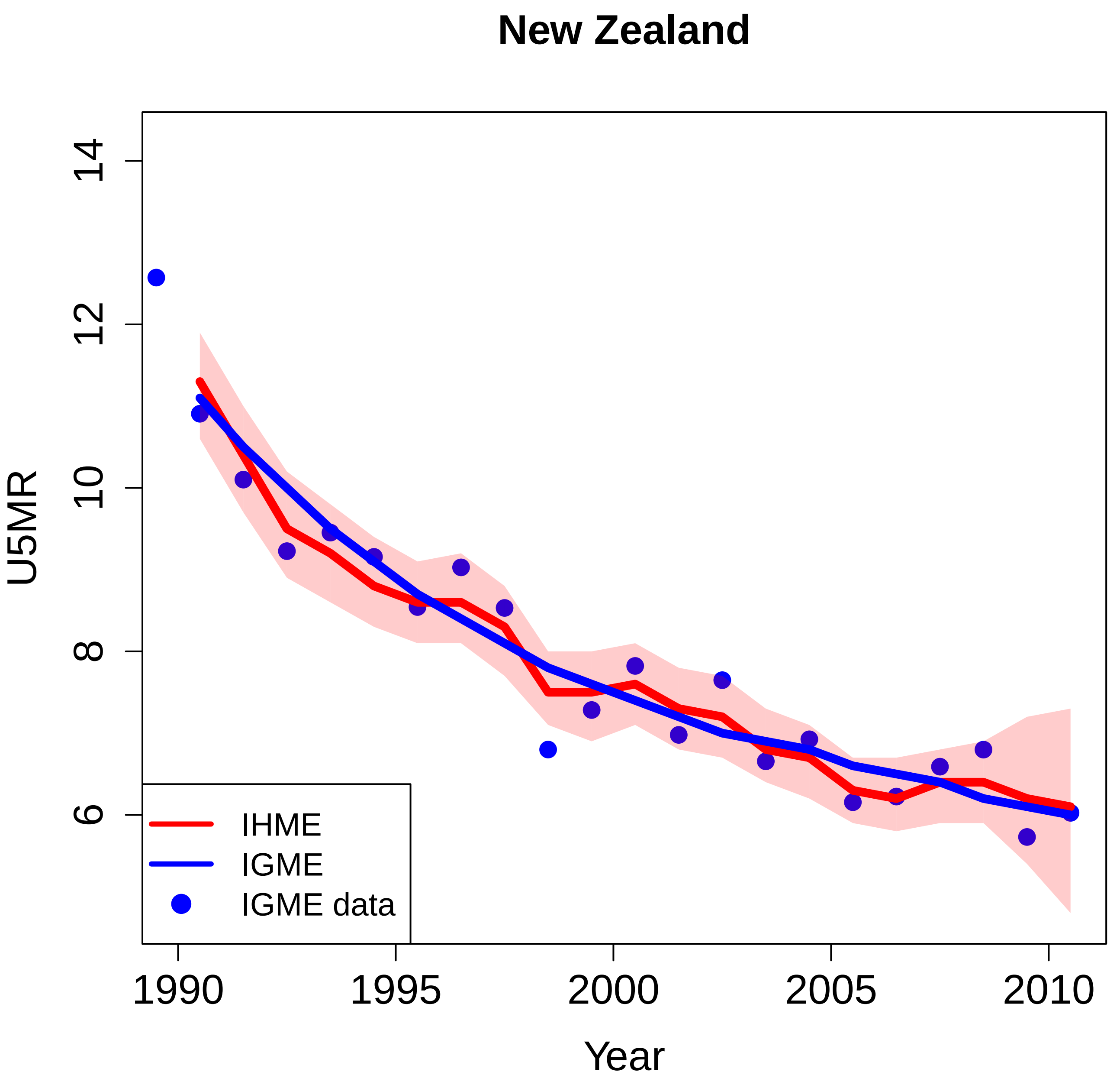
New Zealand
U5MR
14
12
10
8
6
1990
1995
2000
2005
2010
Year
IHME
IGME
IGME data

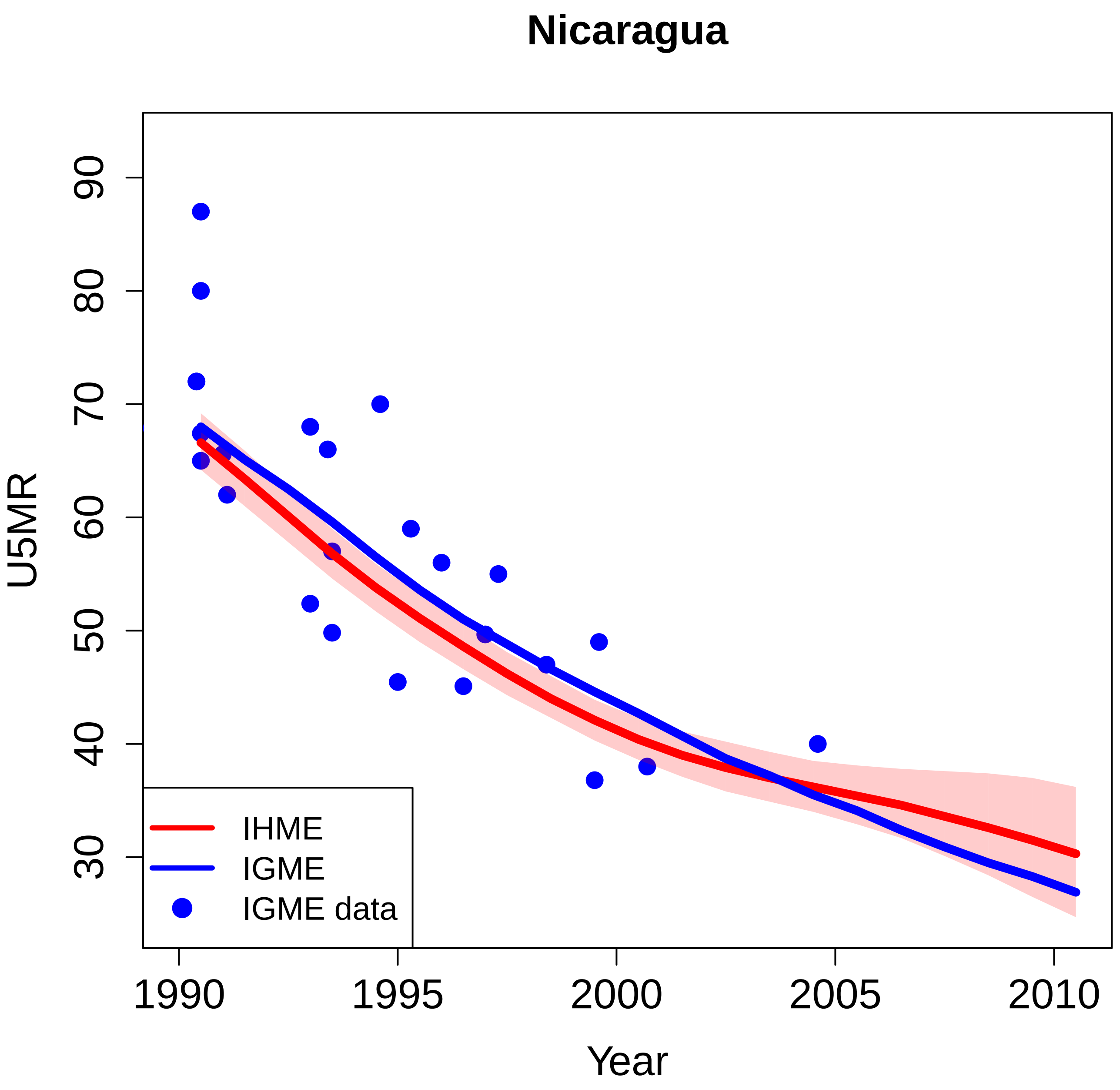
Nicaragua
U5MR
90
80
70
60
50
40
30
1990
1995
2000
2005
2010
Year
IHME
IGME
IGME data

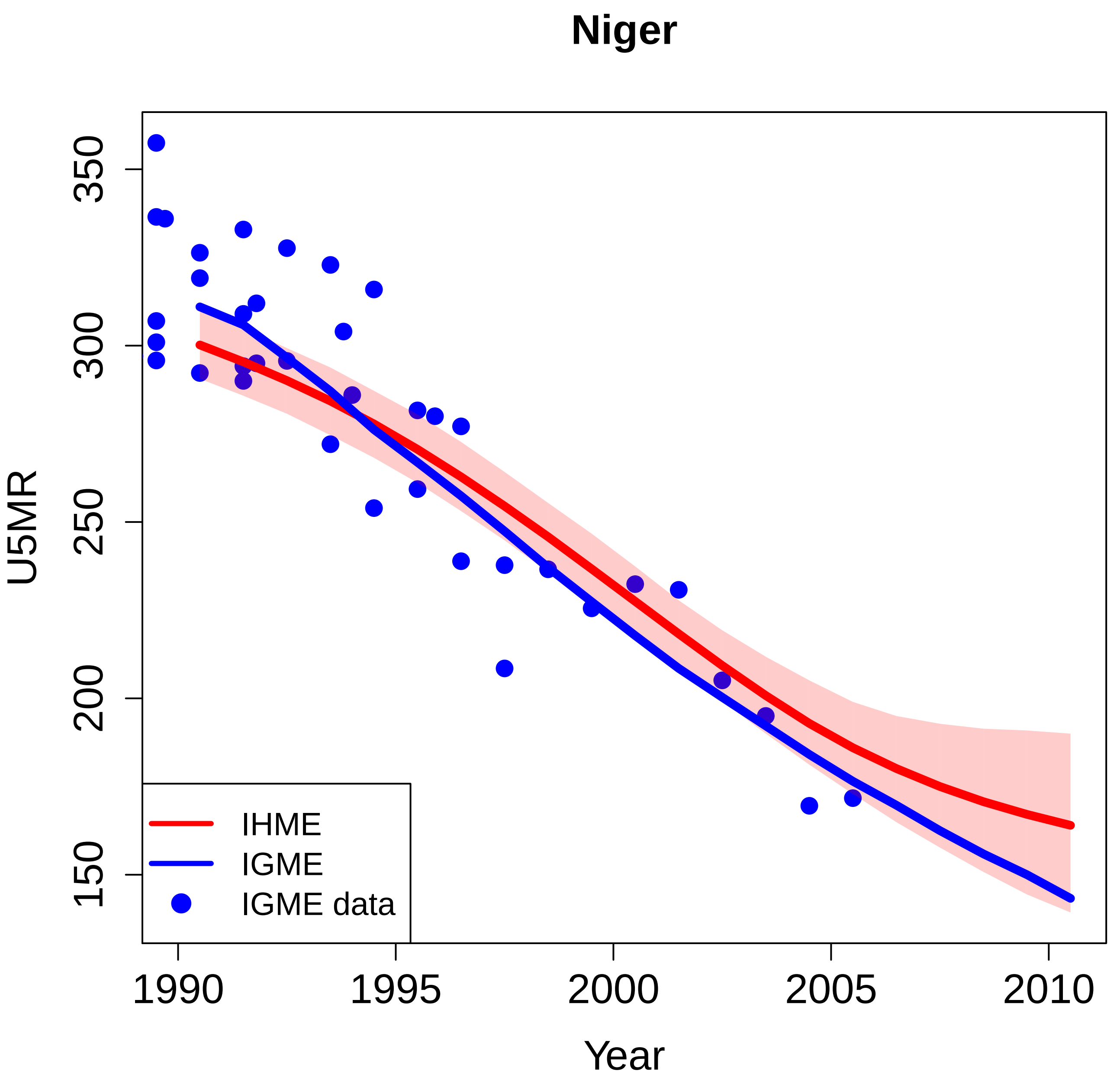
Niger
U5MR
350
300
250
200
150
1990
1995
2000
2005
2010
Year
IHME
IGME
IGME data

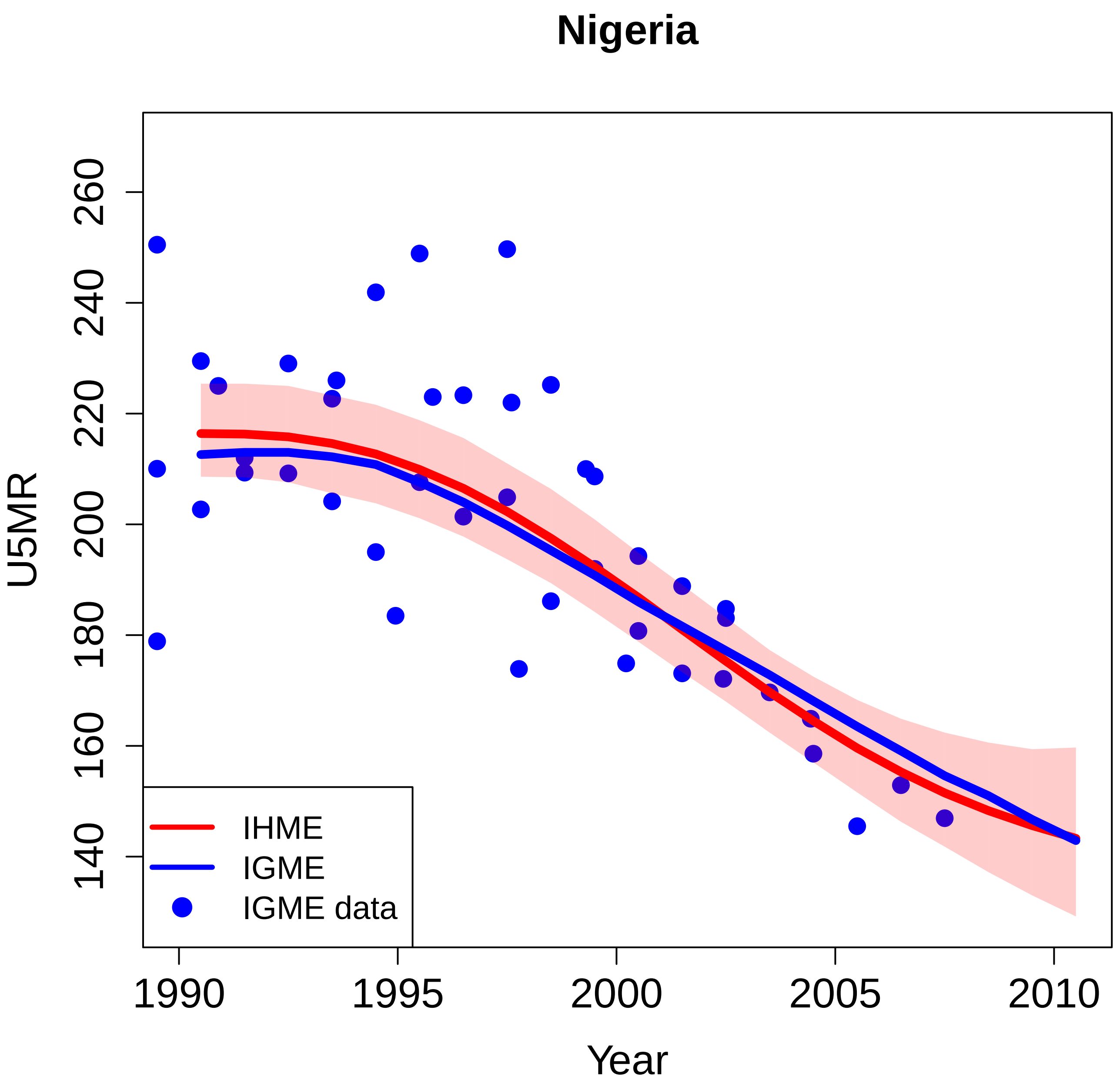

Nigeria
U5MR
140
160
180
200
220
240
260
1990
1995
2000
2005
2010
Year
IHME
IGME
IGME data

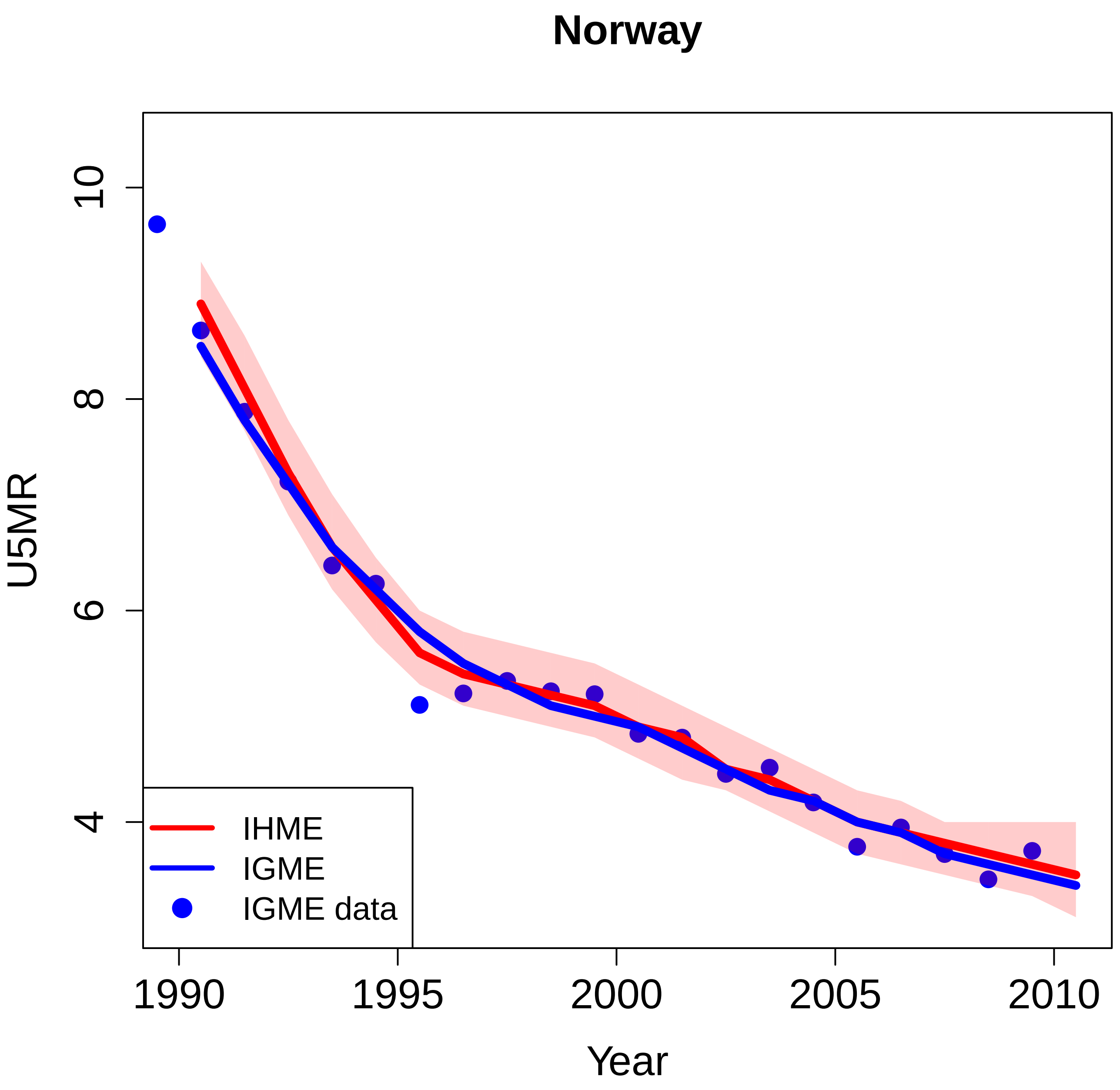

Norway
U5MR
10
8
6
4
1990
1995
2000
2005
2010
Year
IHME
IGME
IGME data

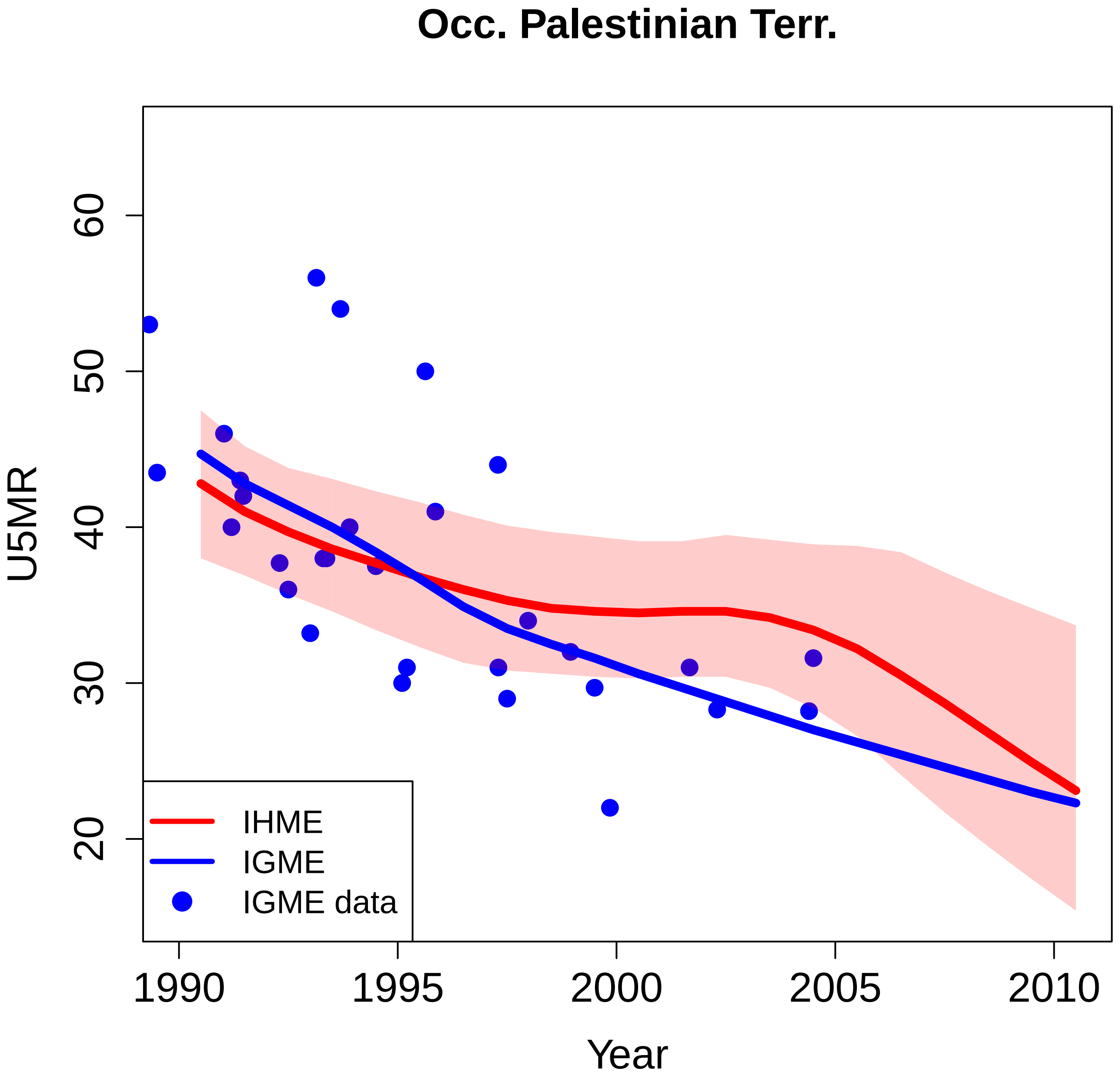

Occ. Palestinian Terr.
U5MR
20
30
40
50
60
1990
1995
2000
2005
2010
Year
IHME
IGME
IGME data

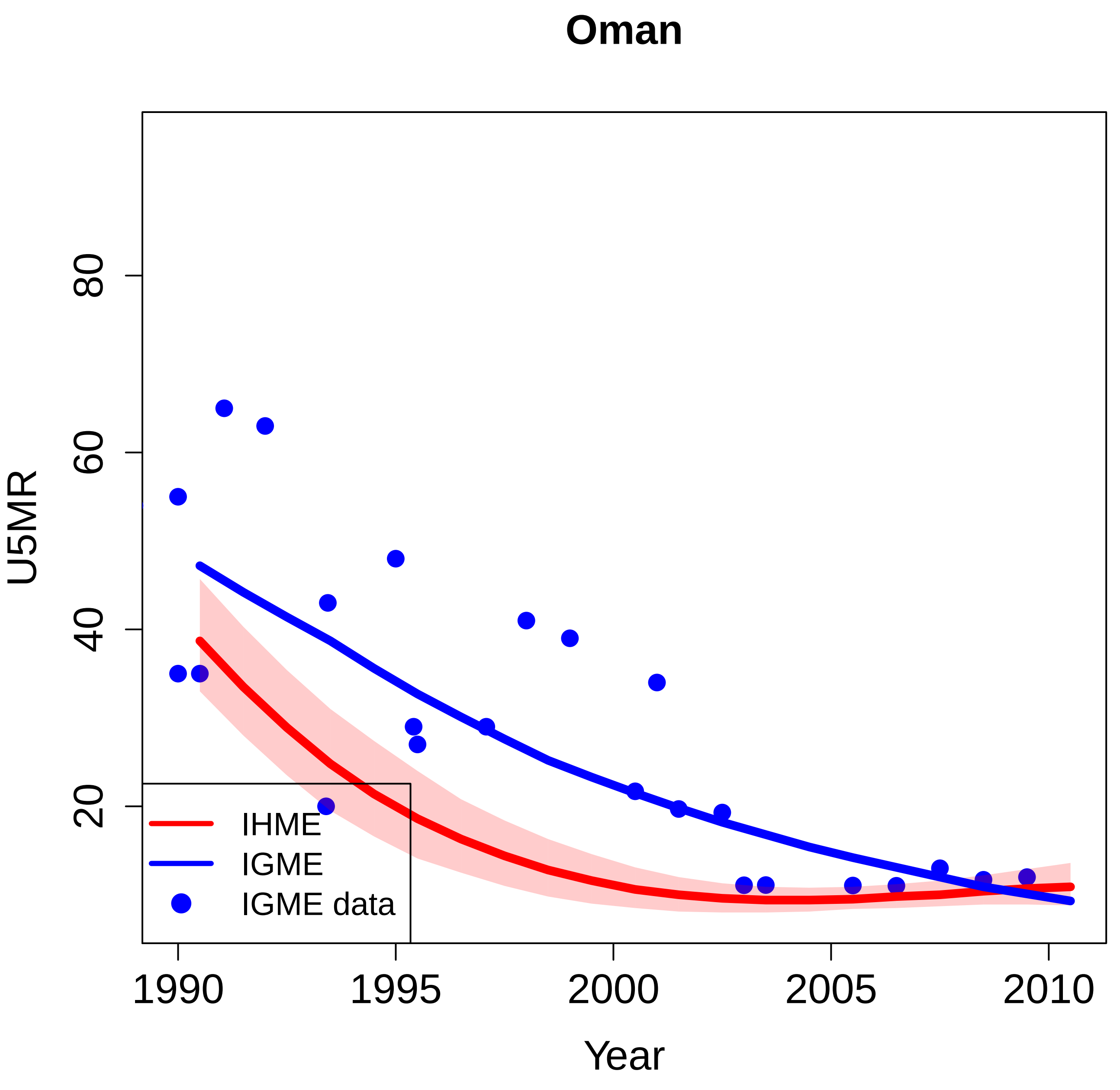

Oman
U5MR
80
60
40
20
1990
1995
2000
2005
2010
Year
IHME
IGME
IGME data

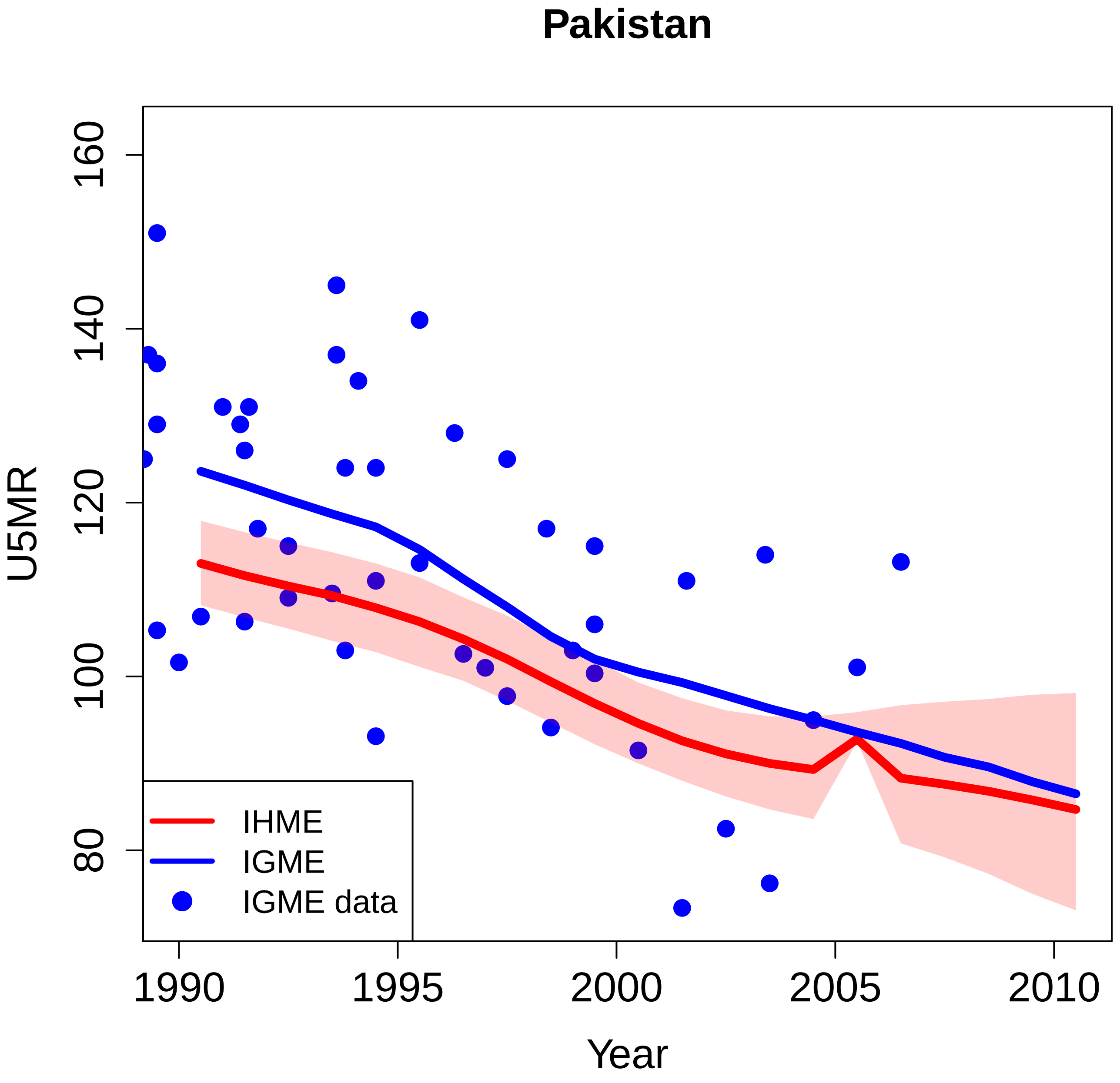

Pakistan
U5MR
160
140
120
100
80
1990
1995
2000
2005
2010
Year
IHME
IGME
IGME data

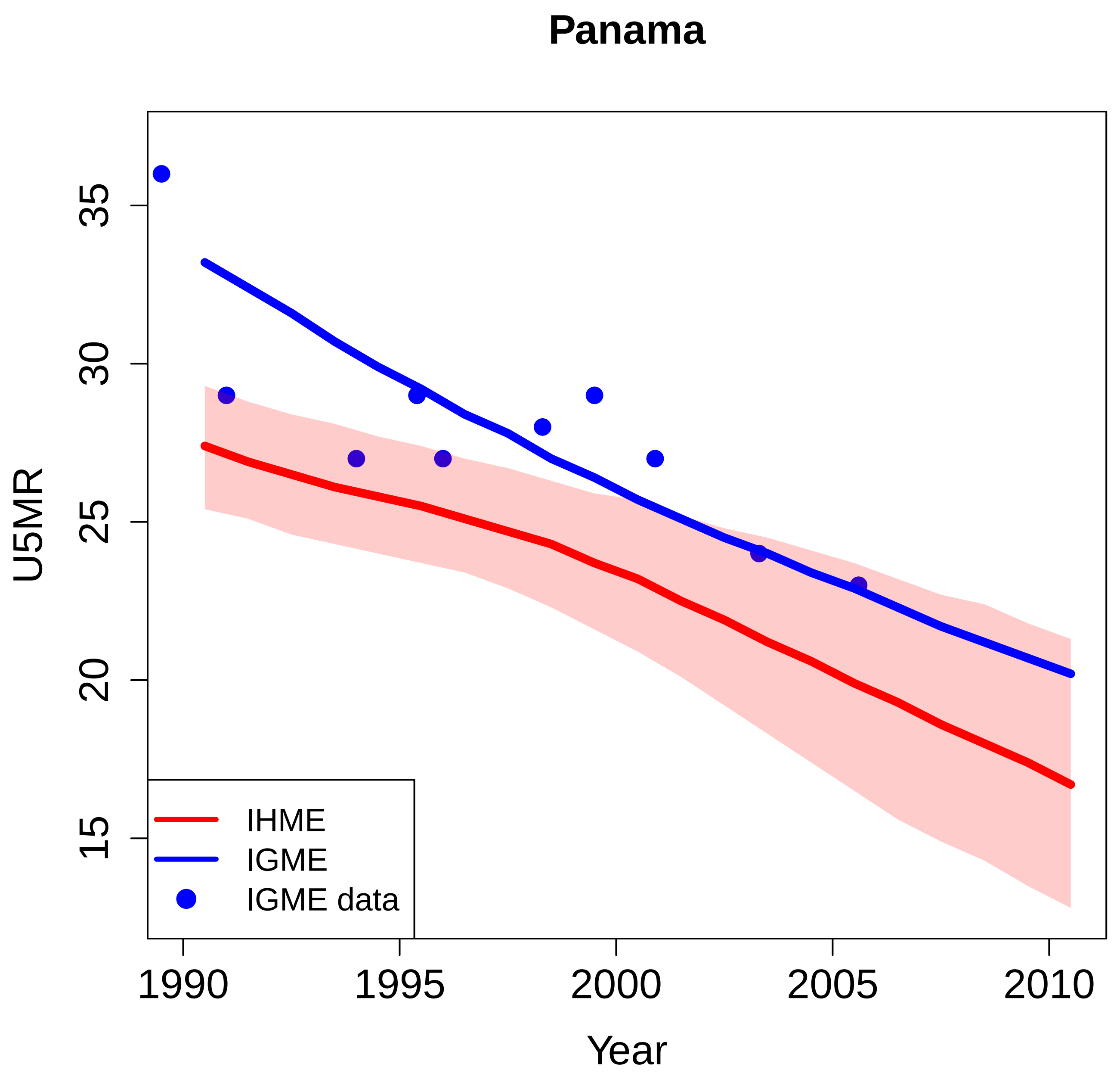

Panama
U5MR
35
30
25
20
15
1990
1995
2000
2005
2010
Year
IHME
IGME
IGME data

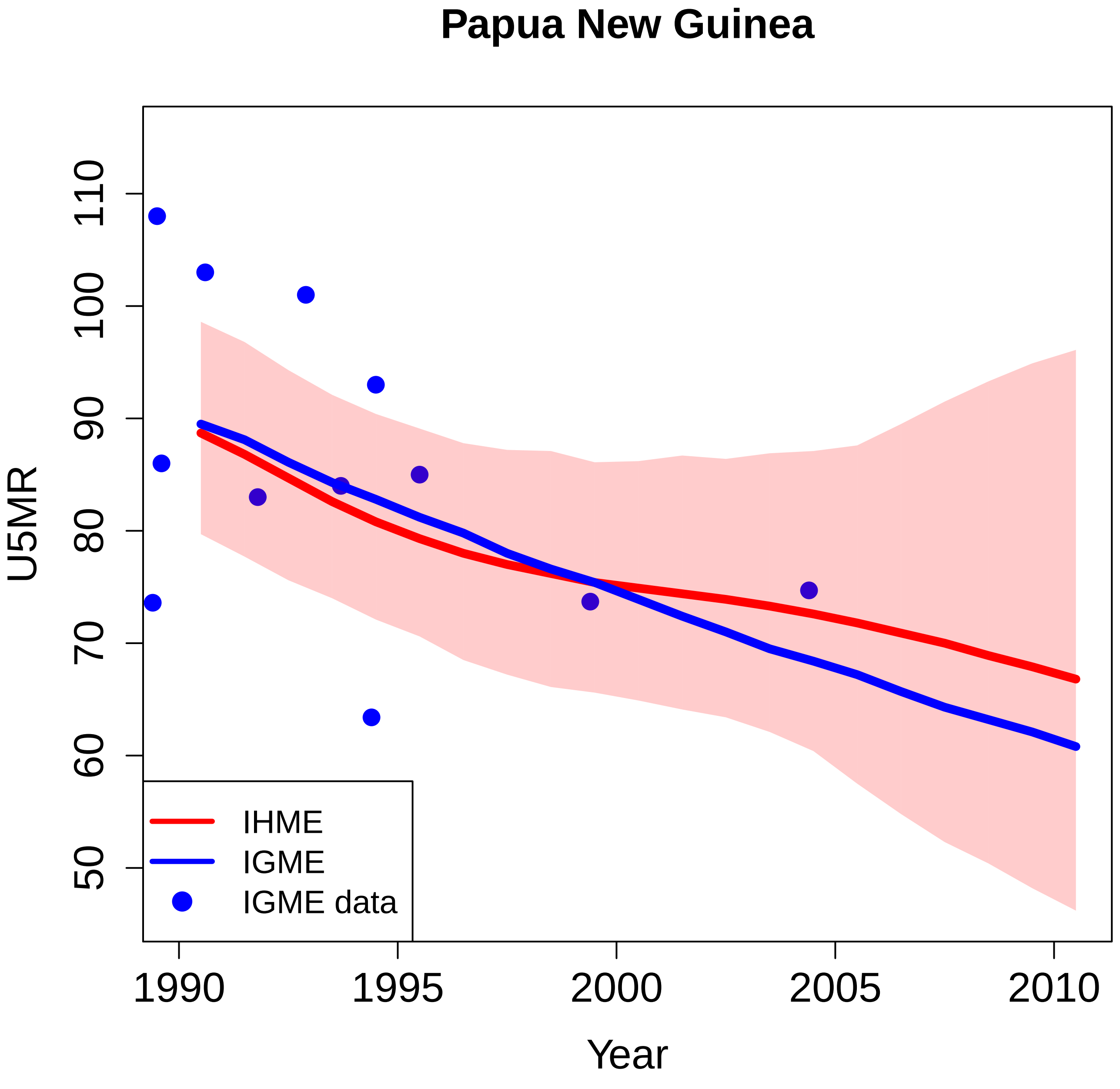

Papua New Guinea
U5MR
110
100
90
80
70
60
50
1990
1995
2000
2005
2010
Year
IHME
IGME
IGME data

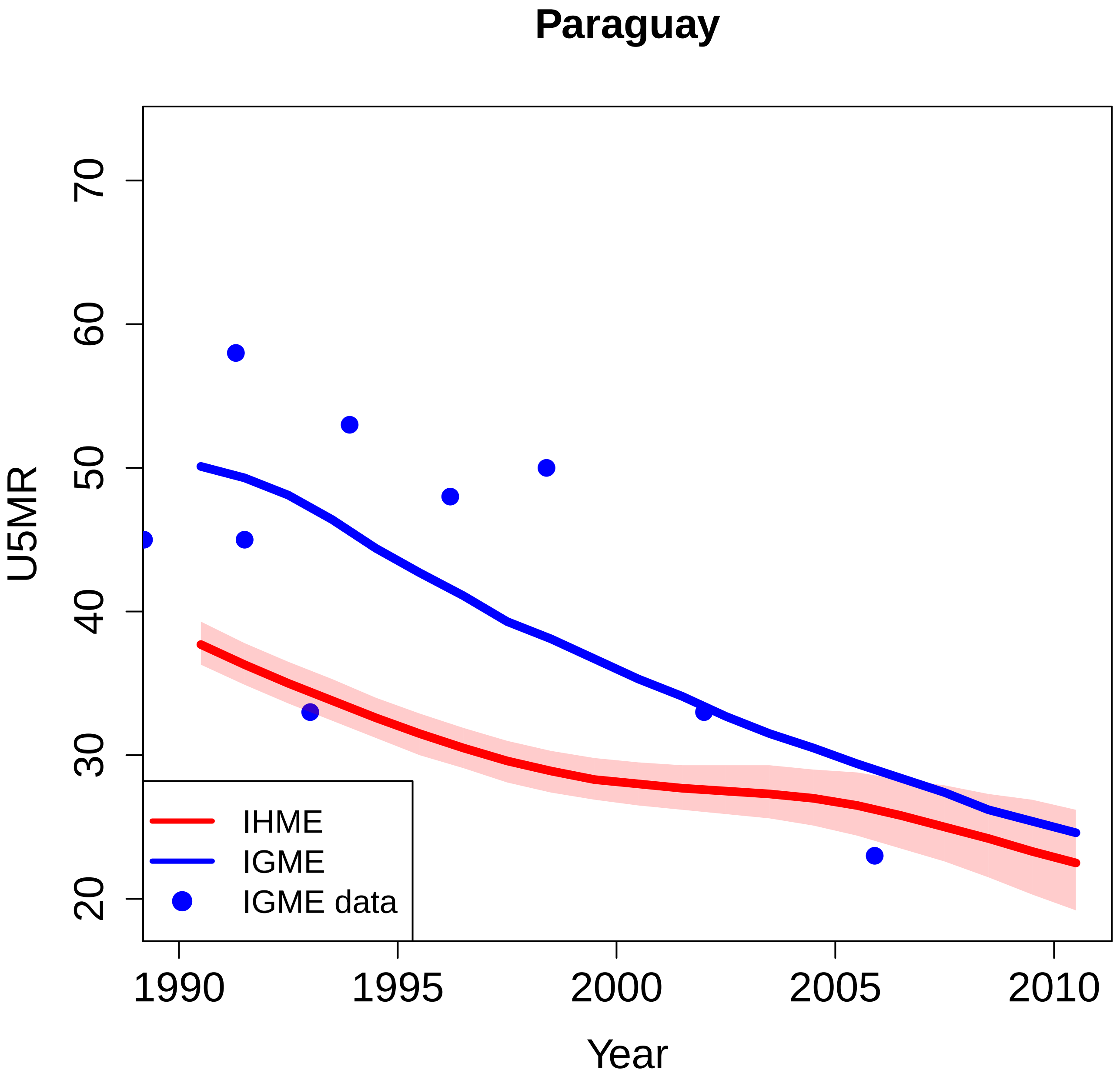
Paraguay
U5MR
70
60
50
40
30
20
1990
1995
2000
2005
2010
Year
IHME
IGME
IGME data

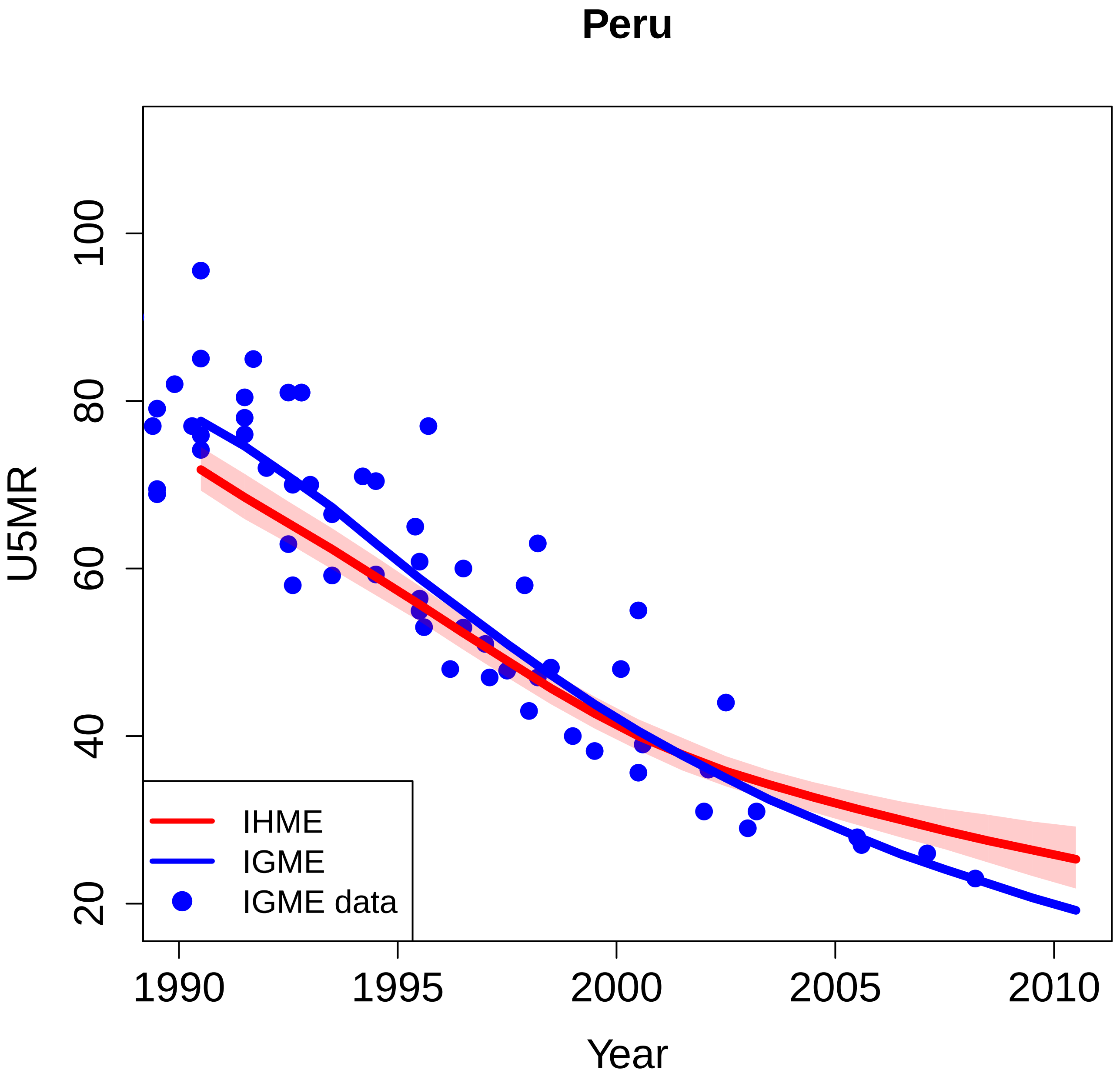

Peru
U5MR
100
80
60
40
20
1990
1995
2000
2005
2010
Year
IHME
IGME
IGME data

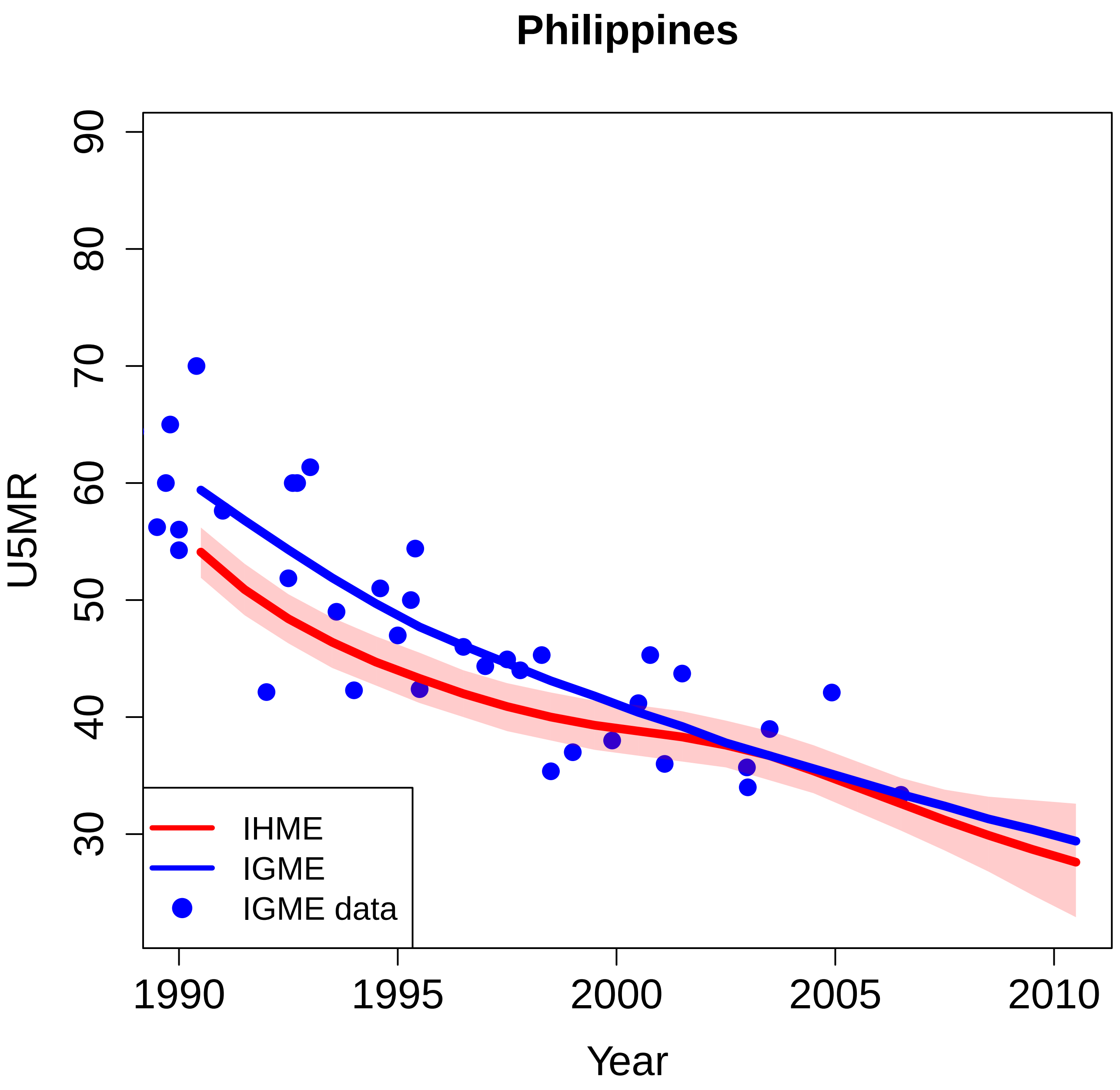
Philippines
U5MR
90
80
70
60
50
40
30
1990
1995
2000
2005
2010
Year
IHME
IGME
IGME data

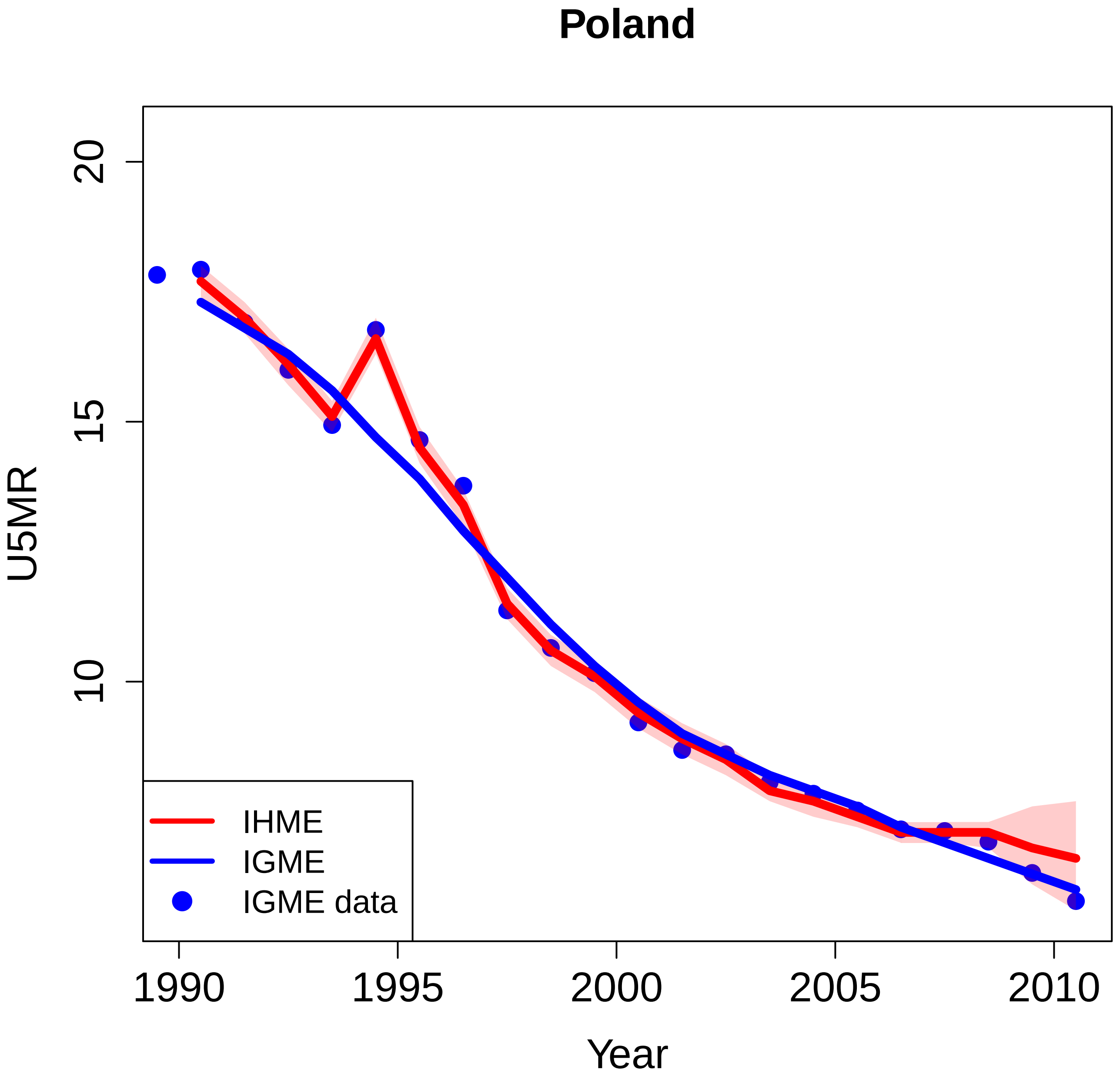

Poland
U5MR
20
15
10
1990
1995
2000
2005
2010
Year
IHME
IGME
IGME data

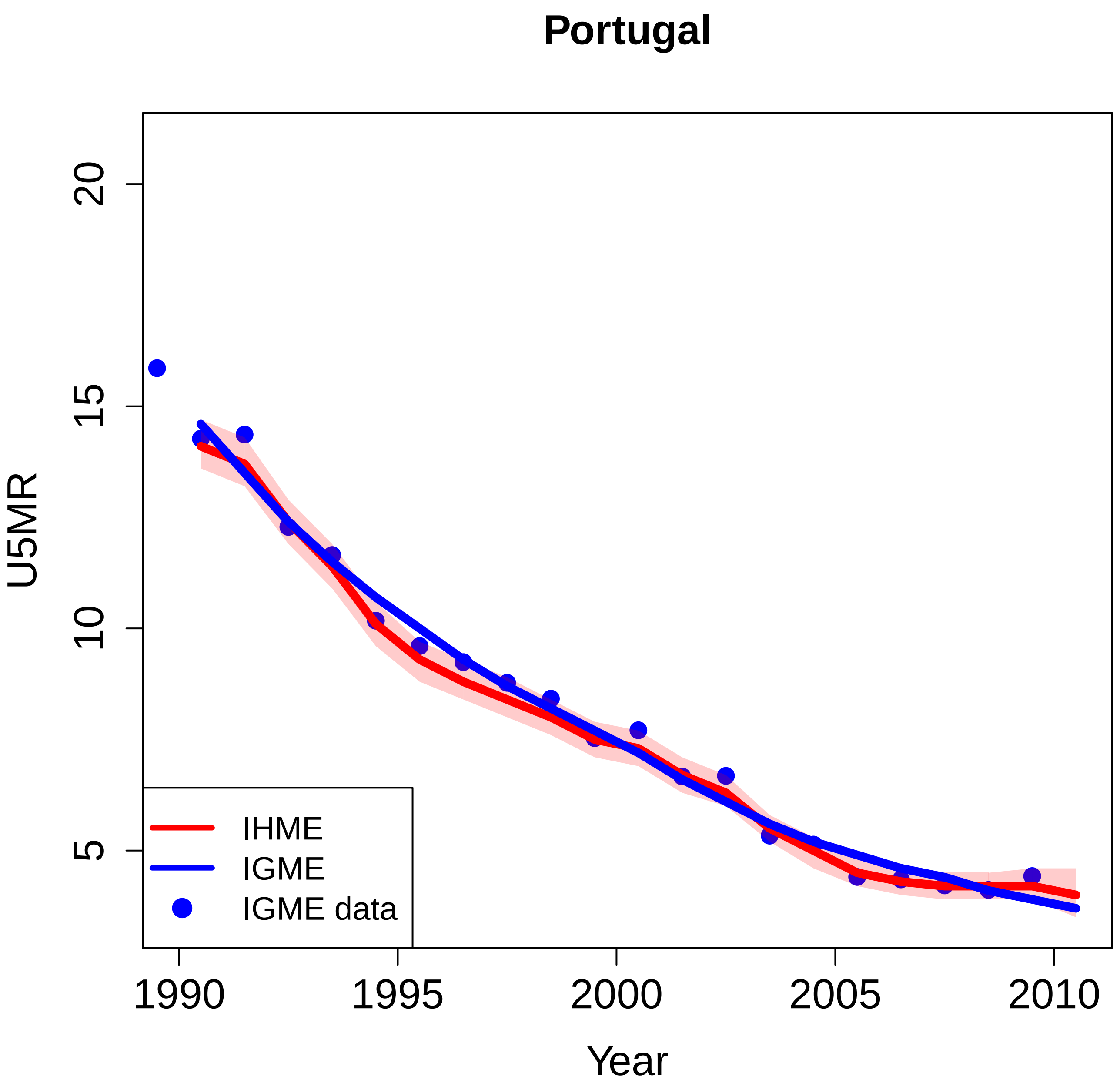
Portugal
U5MR
20
15
10
5
1990
1995
2000
2005
2010
Year
IHME
IGME
IGME data

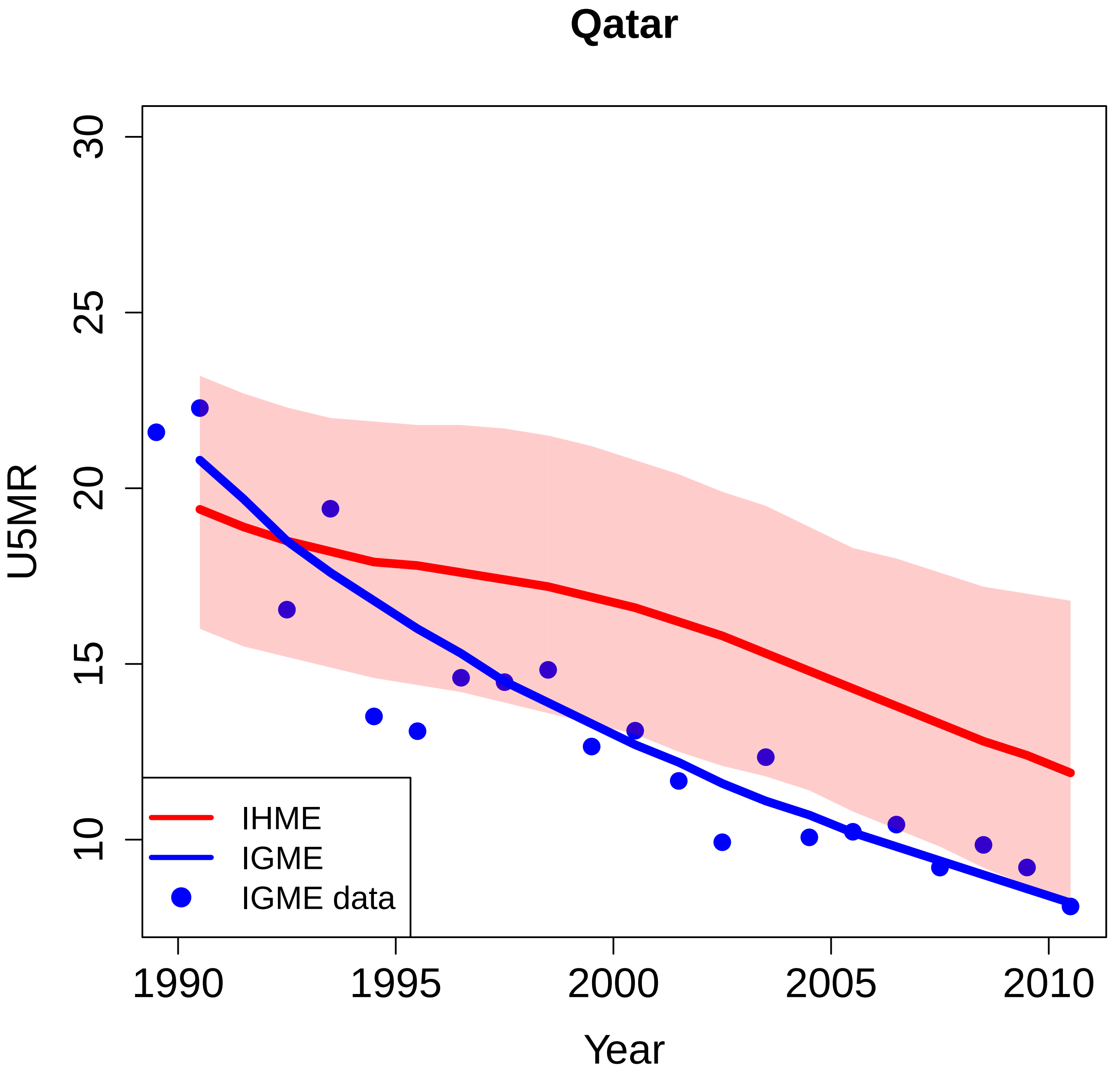

Qatar
U5MR
30
25
20
15
10
1990
1995
2000
2005
2010
Year
IHME
IGME
IGME data

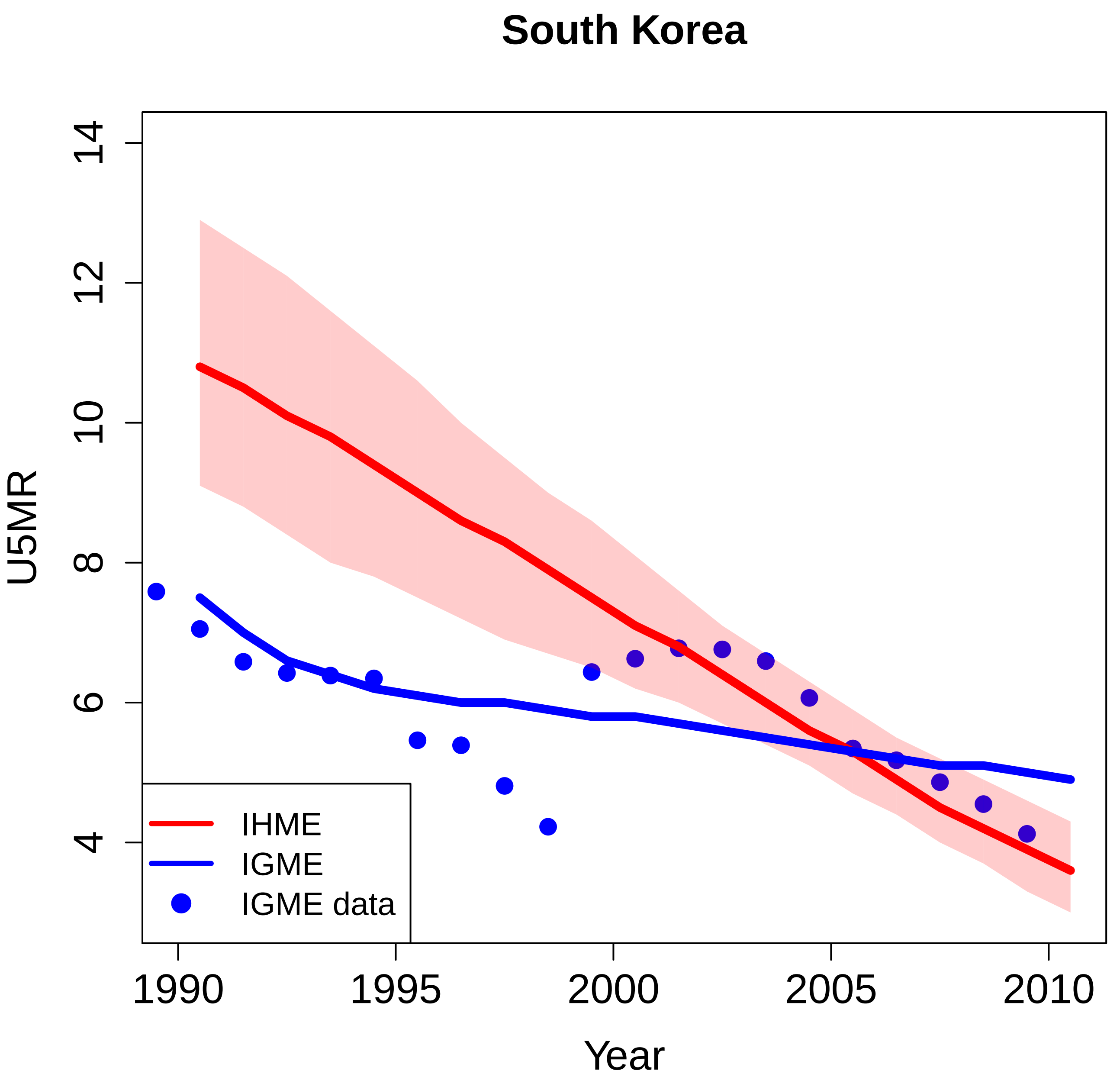
South Korea
U5MR
Year
14
12
10
8
6
4
1990
1995
2000
2005
2010
IHME
IGME
IGME data

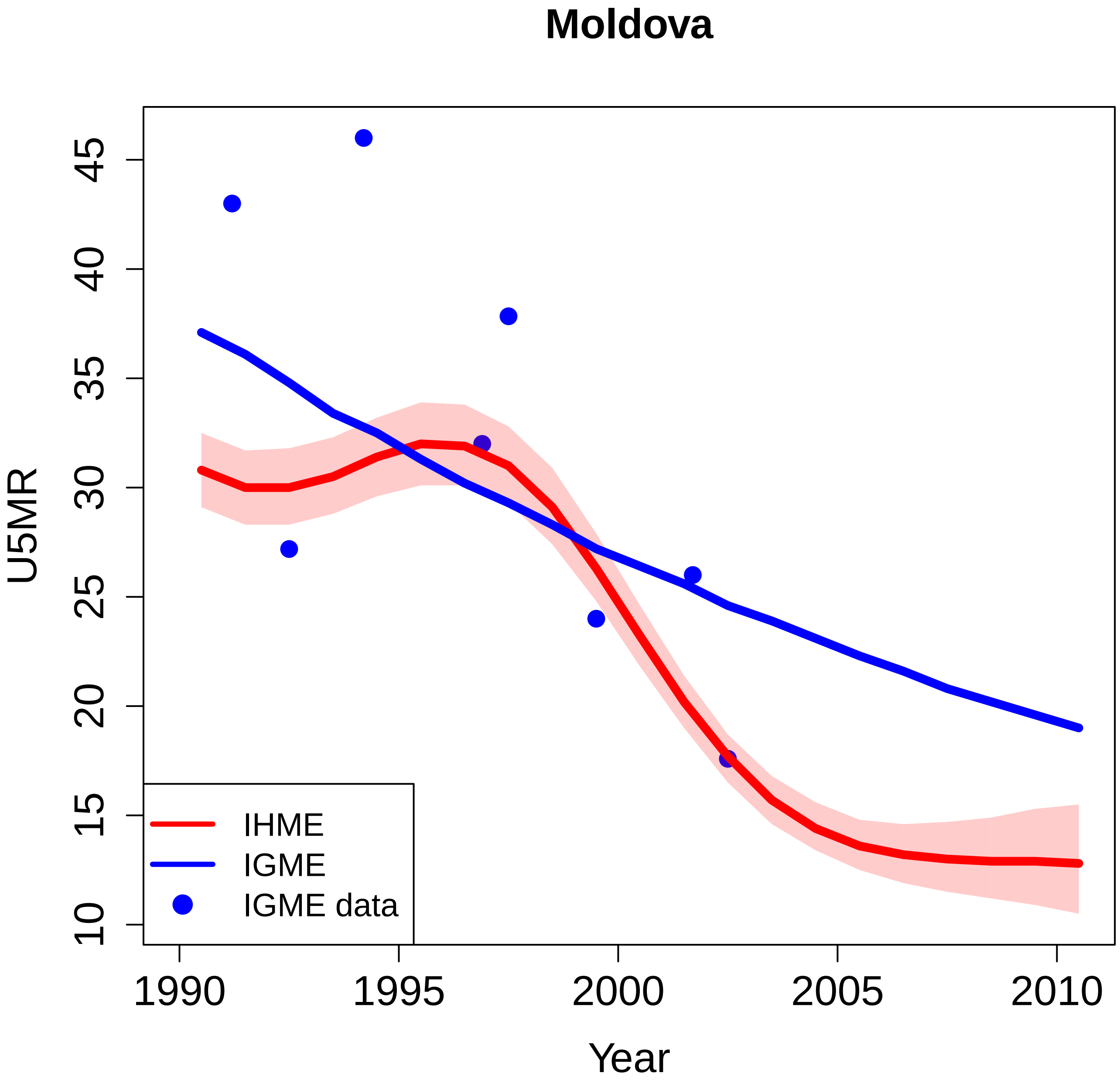

Moldova
U5MR
Year
45
40
35
30
25
20
15
10
1990
1995
2000
2005
2010
IHME
IGME
IGME data

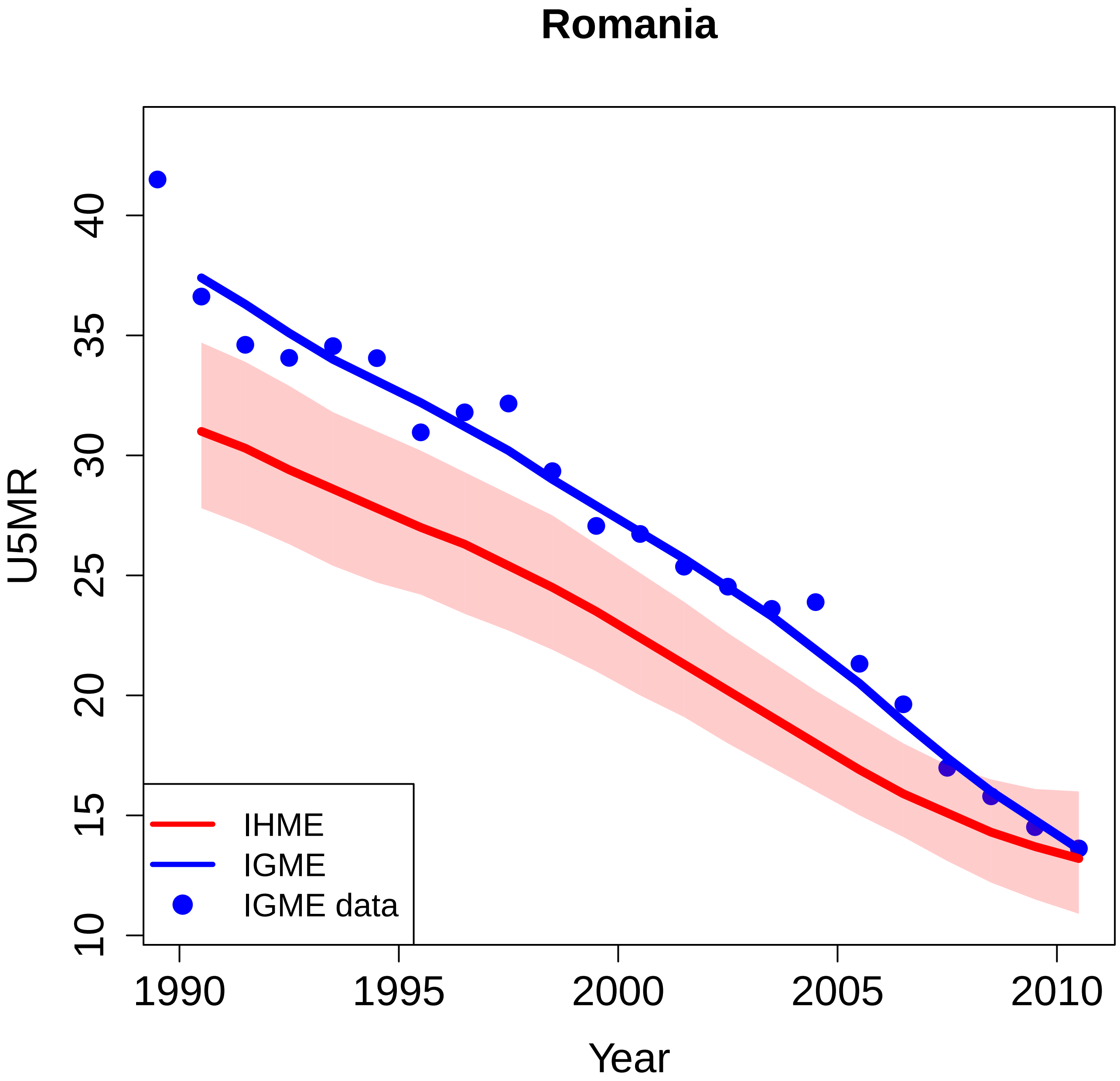
Romania
U5MR
10
15
20
25
30
35
40
1990
1995
2000
2005
2010
Year
IHME
IGME
IGME data

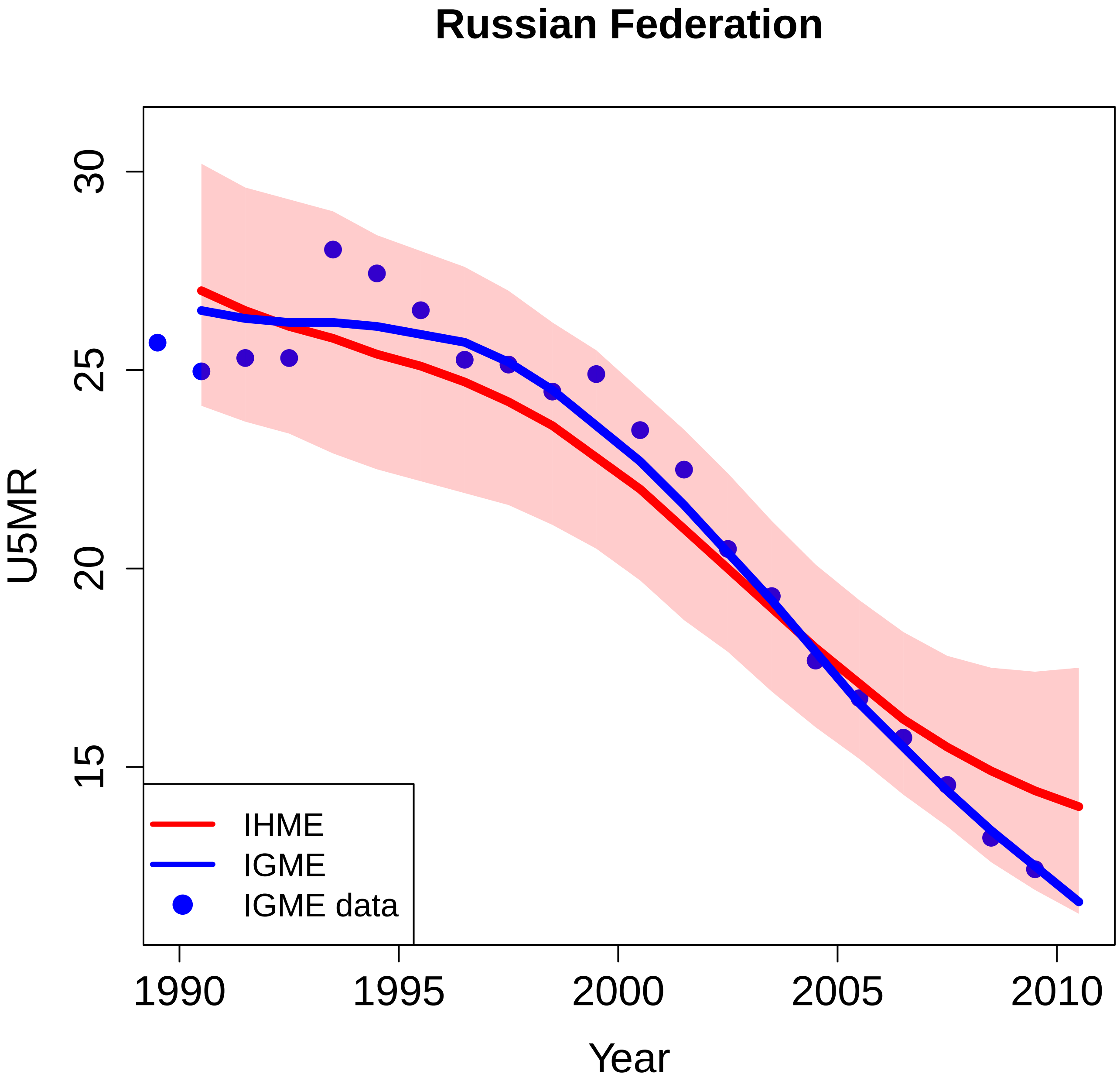
Russian Federation
U5MR
30
25
20
15
1990
1995
2000
2005
2010
Year
IHME
IGME
IGME data

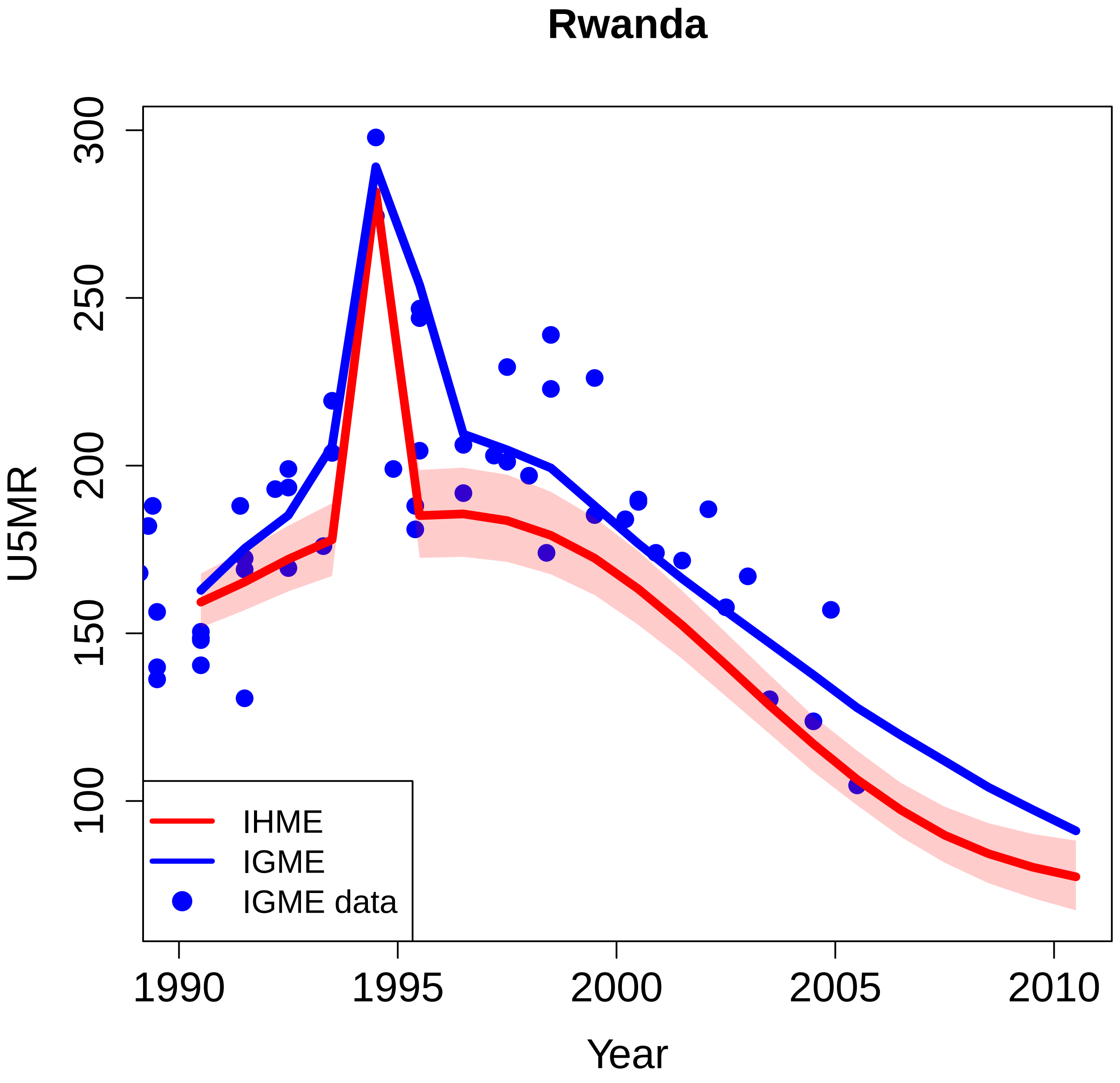

Rwanda
U5MR
Year
300
250
200
150
100
1990
1995
2000
2005
2010
IHME
IGME
IGME data

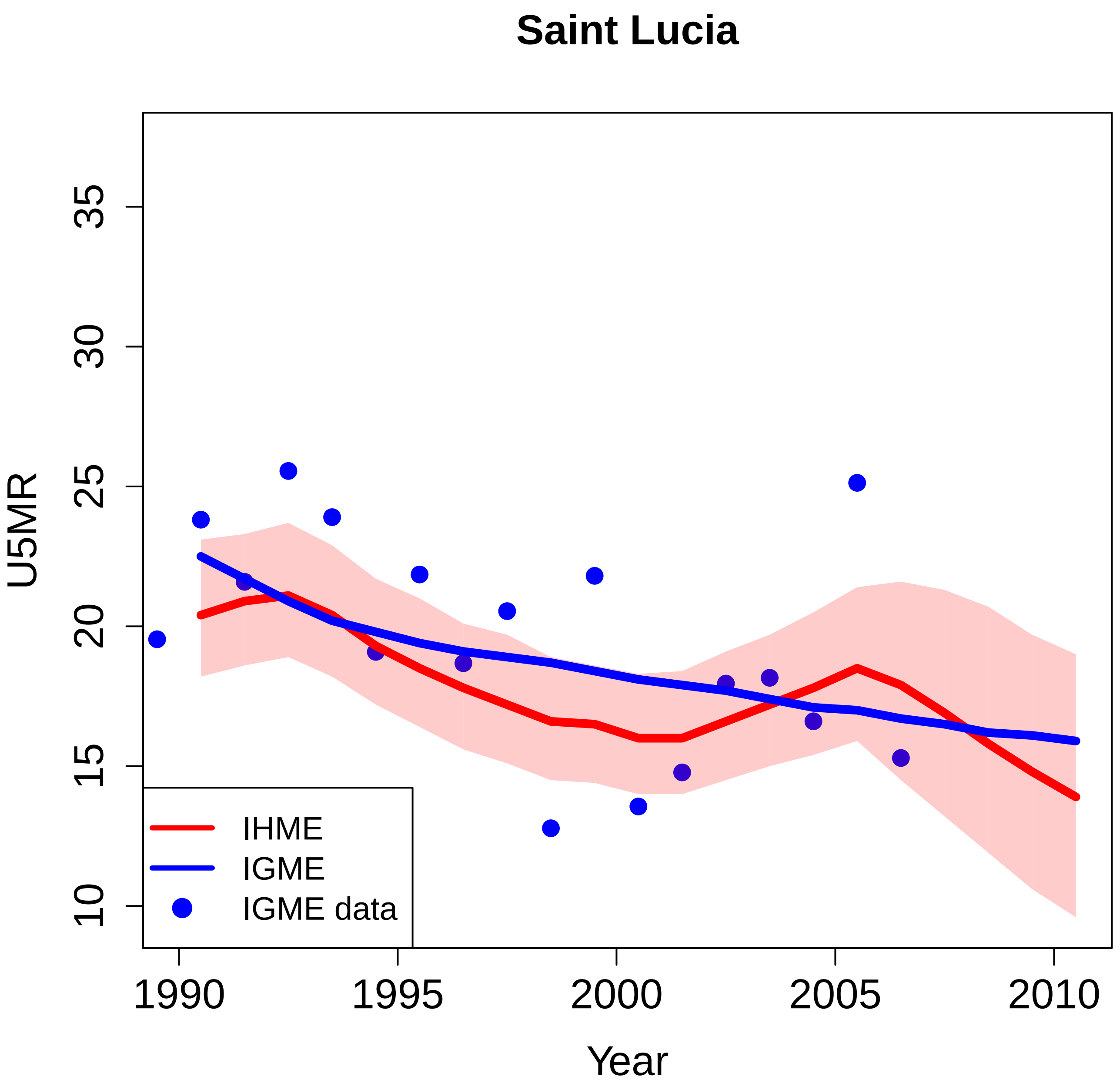

Saint Lucia
U5MR
10
15
20
25
30
35
1990
1995
2000
2005
2010
Year
IHME
IGME
IGME data

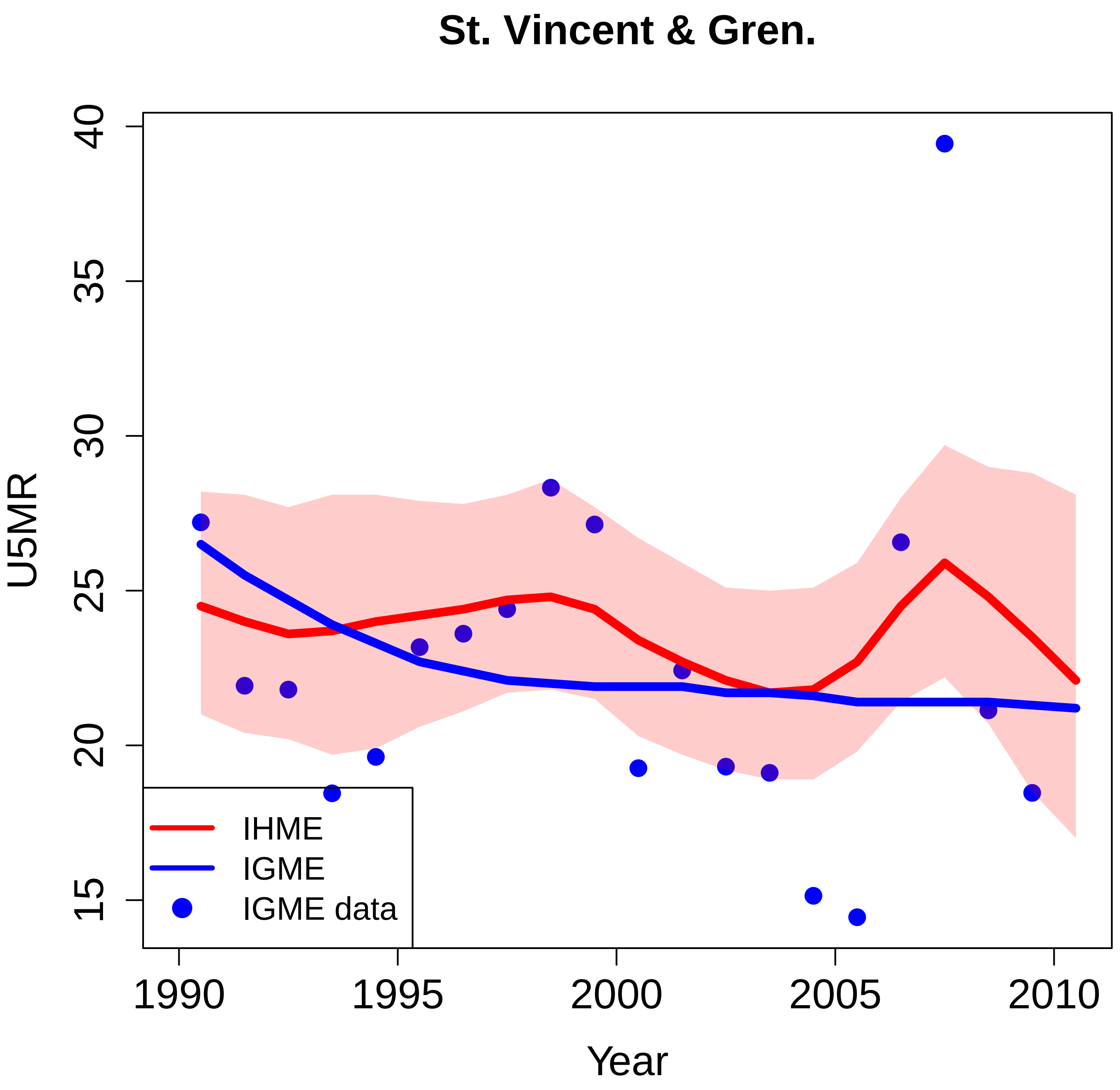
St. Vincent & Gren.
U5MR
40
35
30
25
20
15
1990
1995
2000
2005
2010
Year
IHME
IGME
IGME data

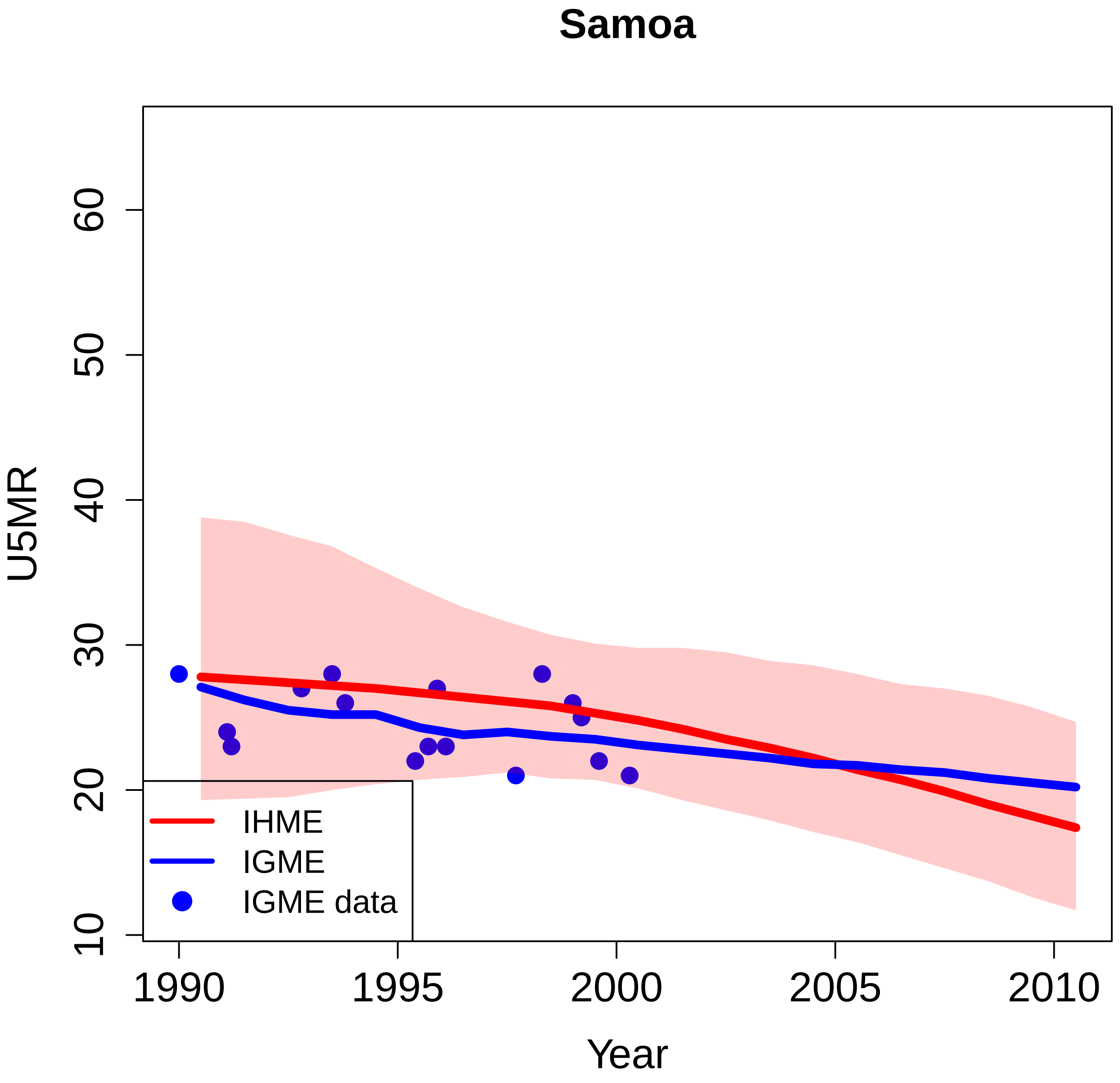
Samoa
U5MR
60
50
40
30
20
10
1990
1995
2000
2005
2010
Year
IHME
IGME
IGME data

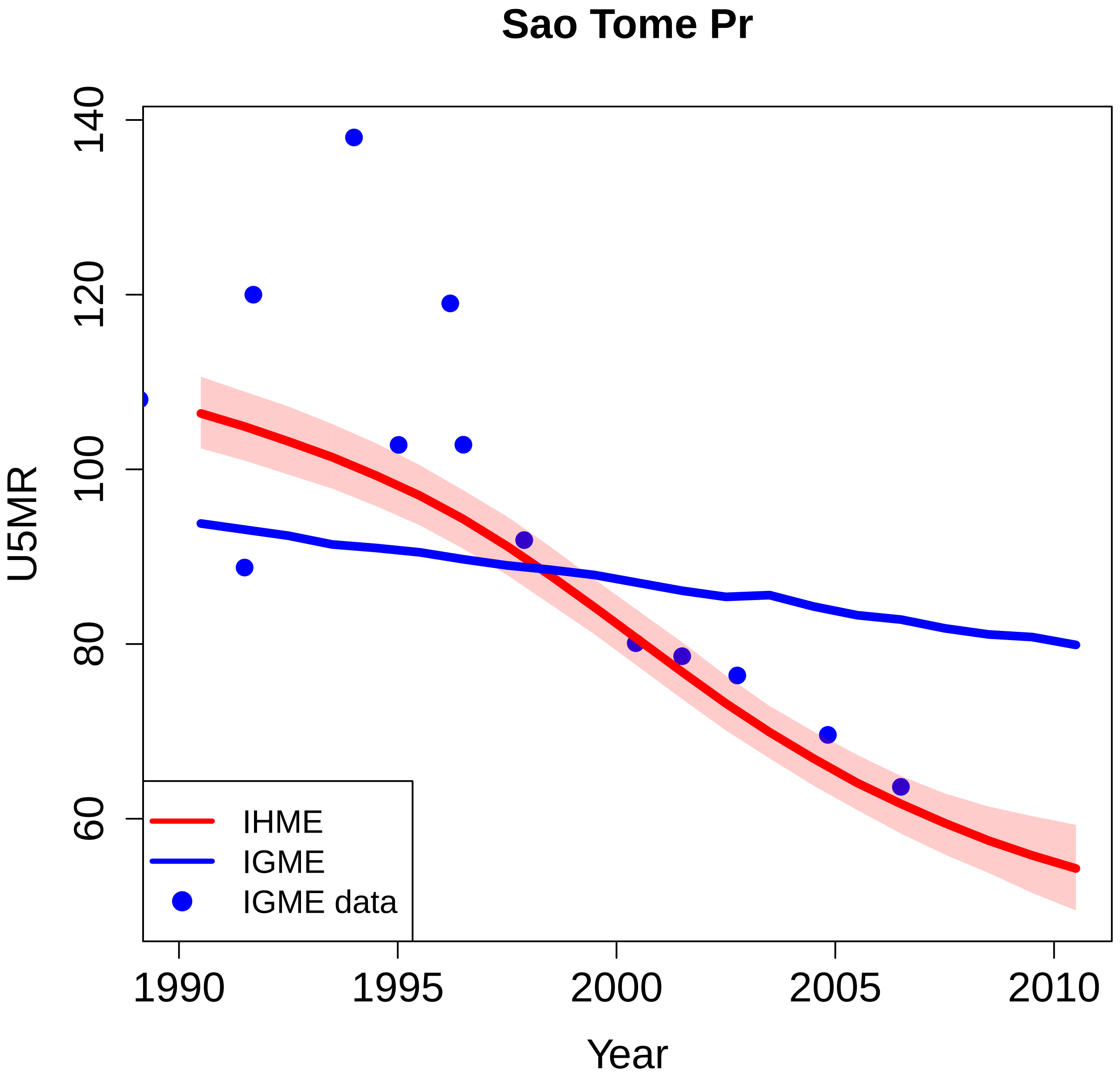
Sao Tome Pr
140
120
100
80
60
U5MR
1990
1995
2000
2005
2010
Year
IHME
IGME
IGME data

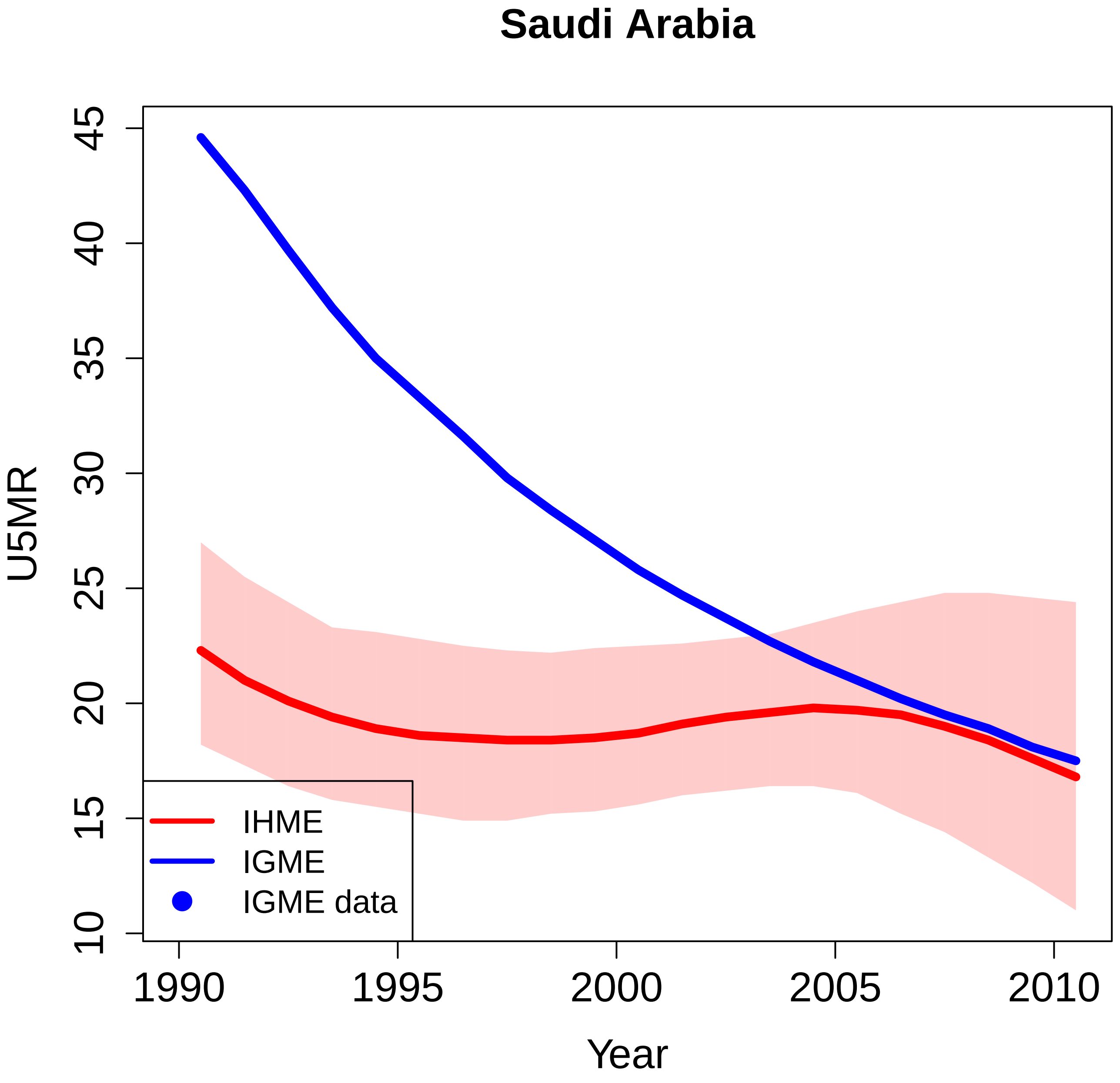
Saudi Arabia
U5MR
Year
45
40
35
30
25
20
15
10
1990
1995
2000
2005
2010
IHME
IGME
IGME data

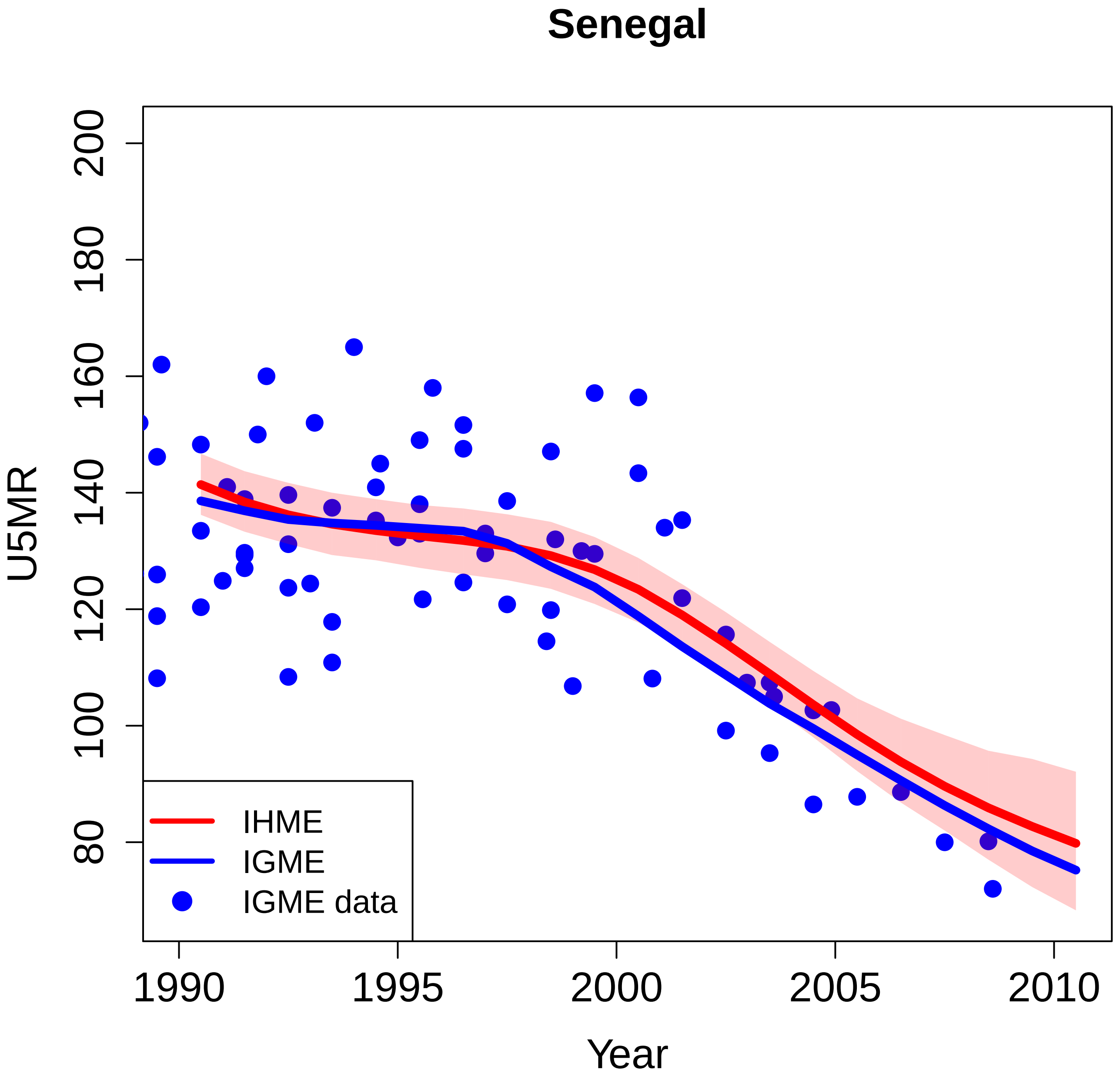
Senegal
U5MR
200
180
160
140
120
100
80
1990
1995
2000
2005
2010
Year
IHME
IGME
IGME data

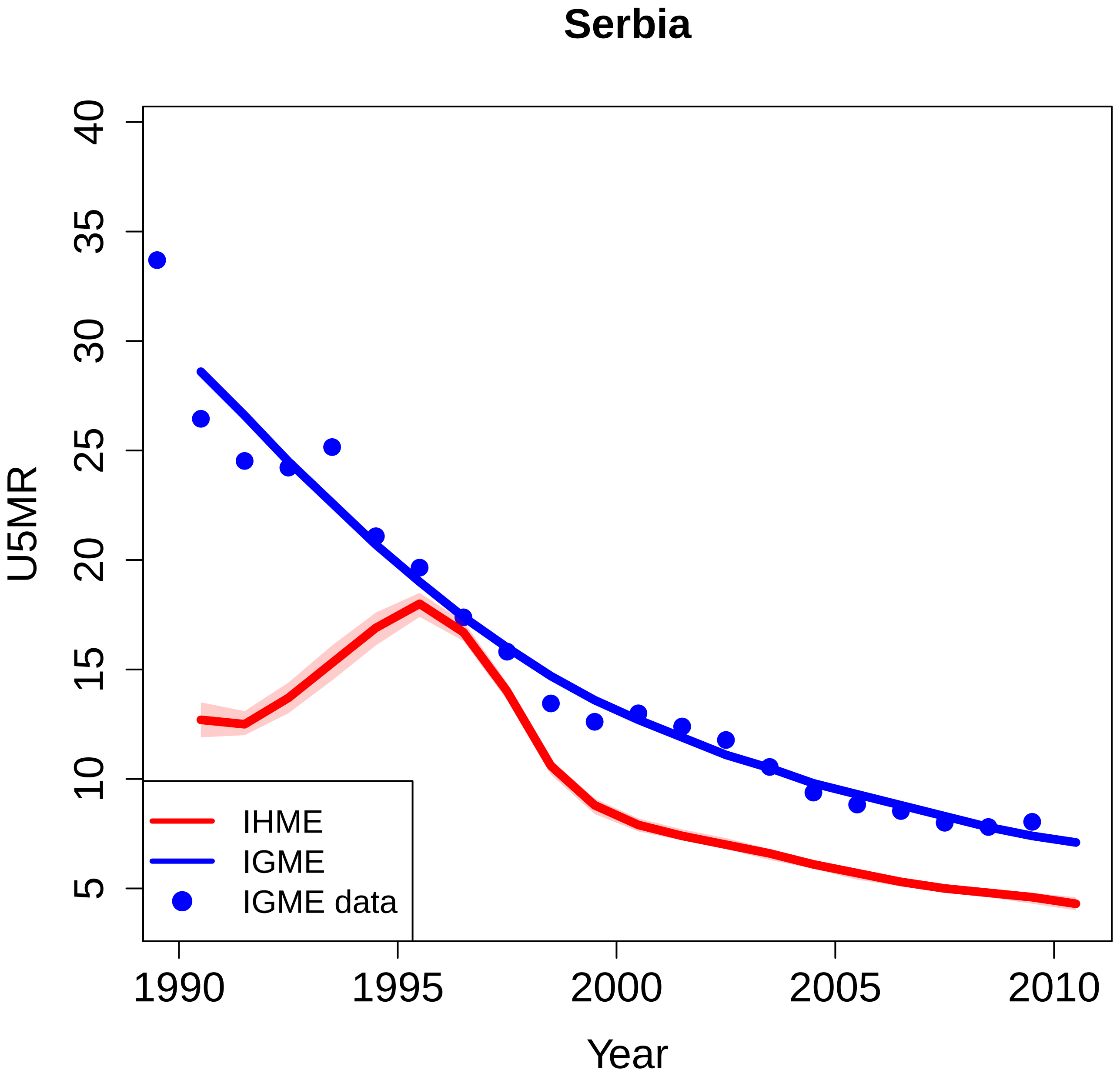

Serbia
U5MR
40
35
30
25
20
15
10
5
1990
1995
2000
2005
2010
Year
IHME
IGME
IGME data

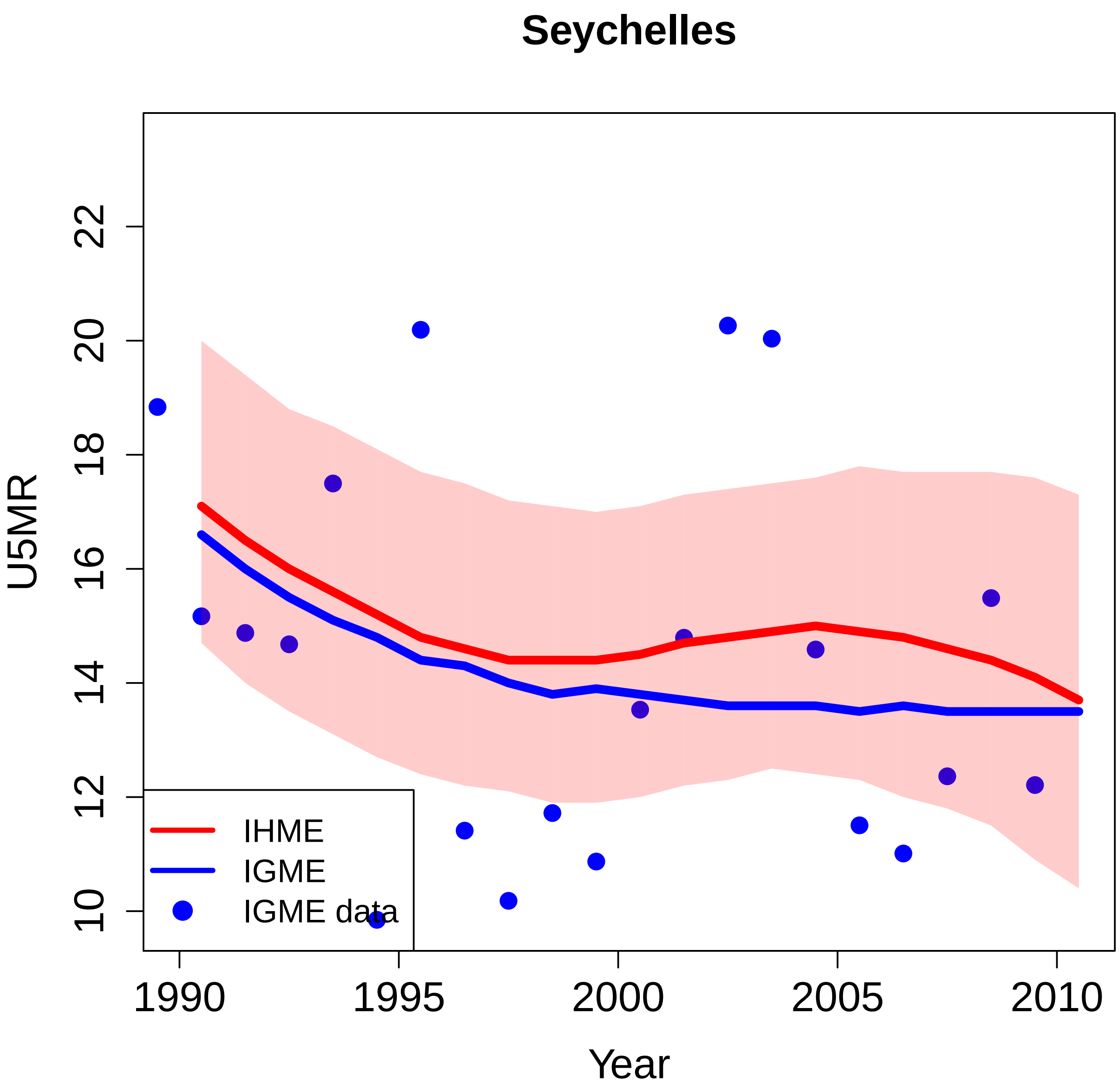

Seychelles
U5MR
22
20
18
16
14
12
10
IHME
IGME
IGME data
1990
1995
2000
2005
2010
Year

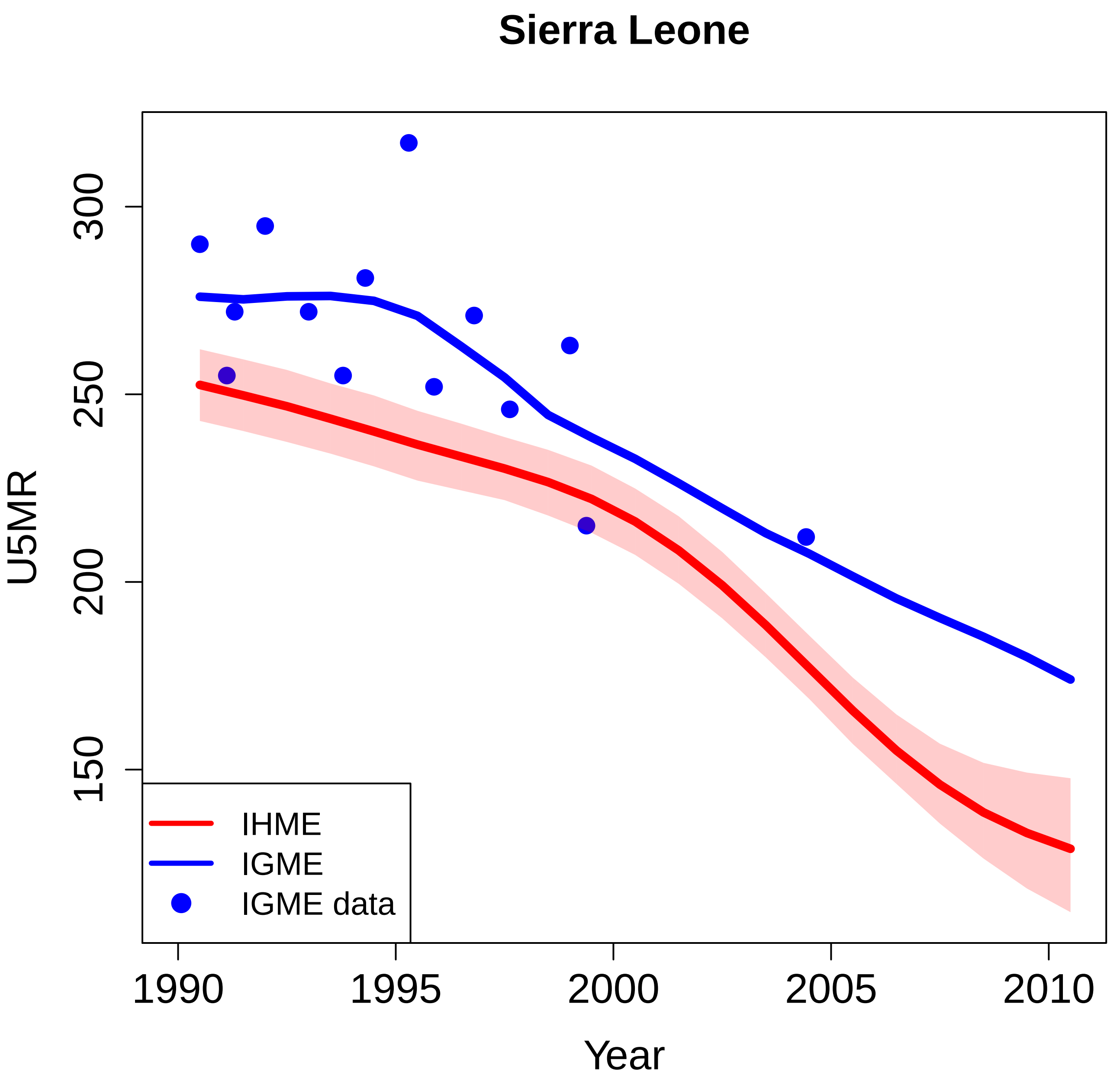
Sierra Leone
U5MR
300
250
200
150
1990
1995
2000
2005
2010
Year
IHME
IGME
IGME data

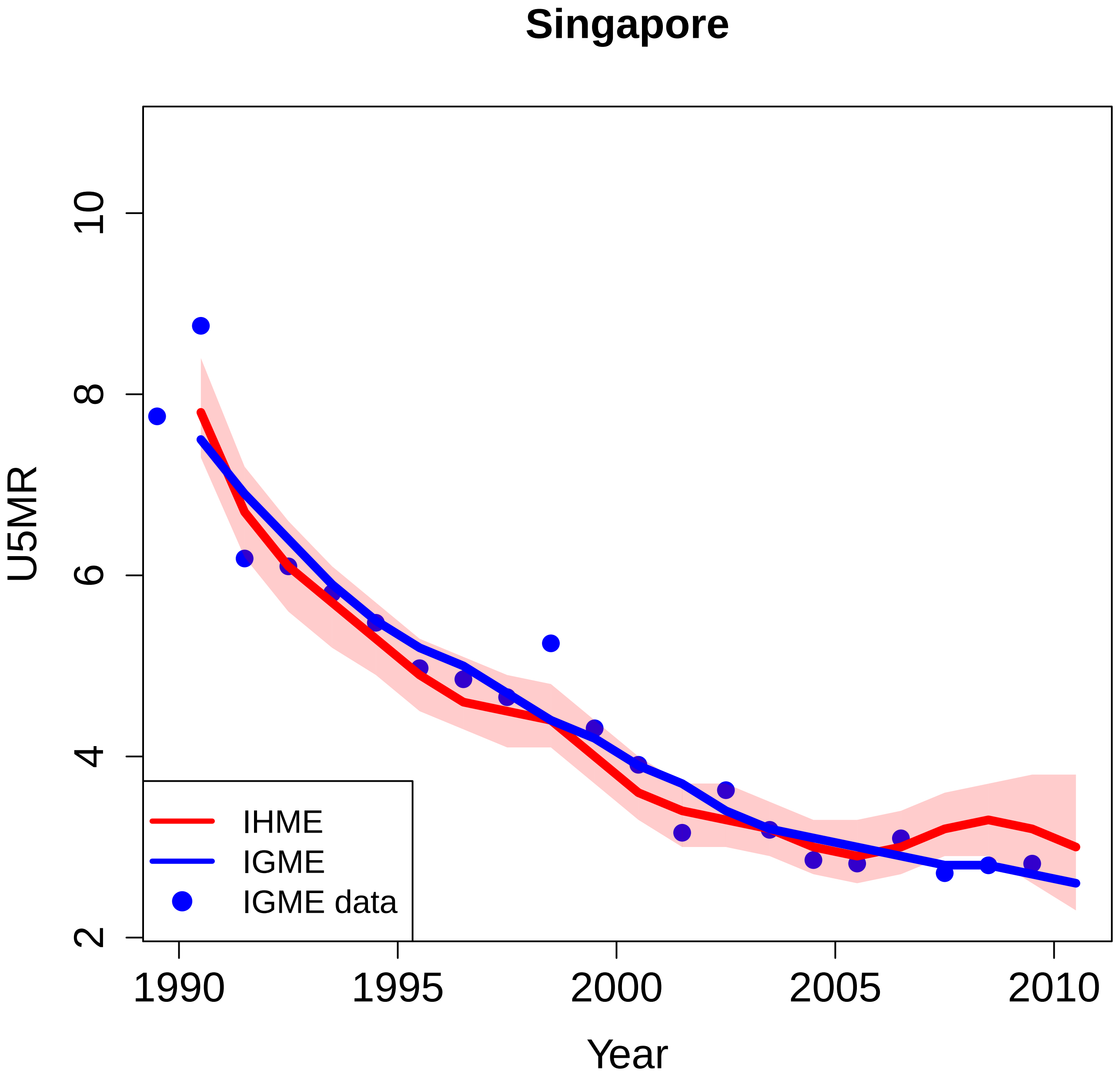
Singapore
U5MR
Year
10
8
6
4
2
1990
1995
2000
2005
2010
IHME
IGME
IGME data

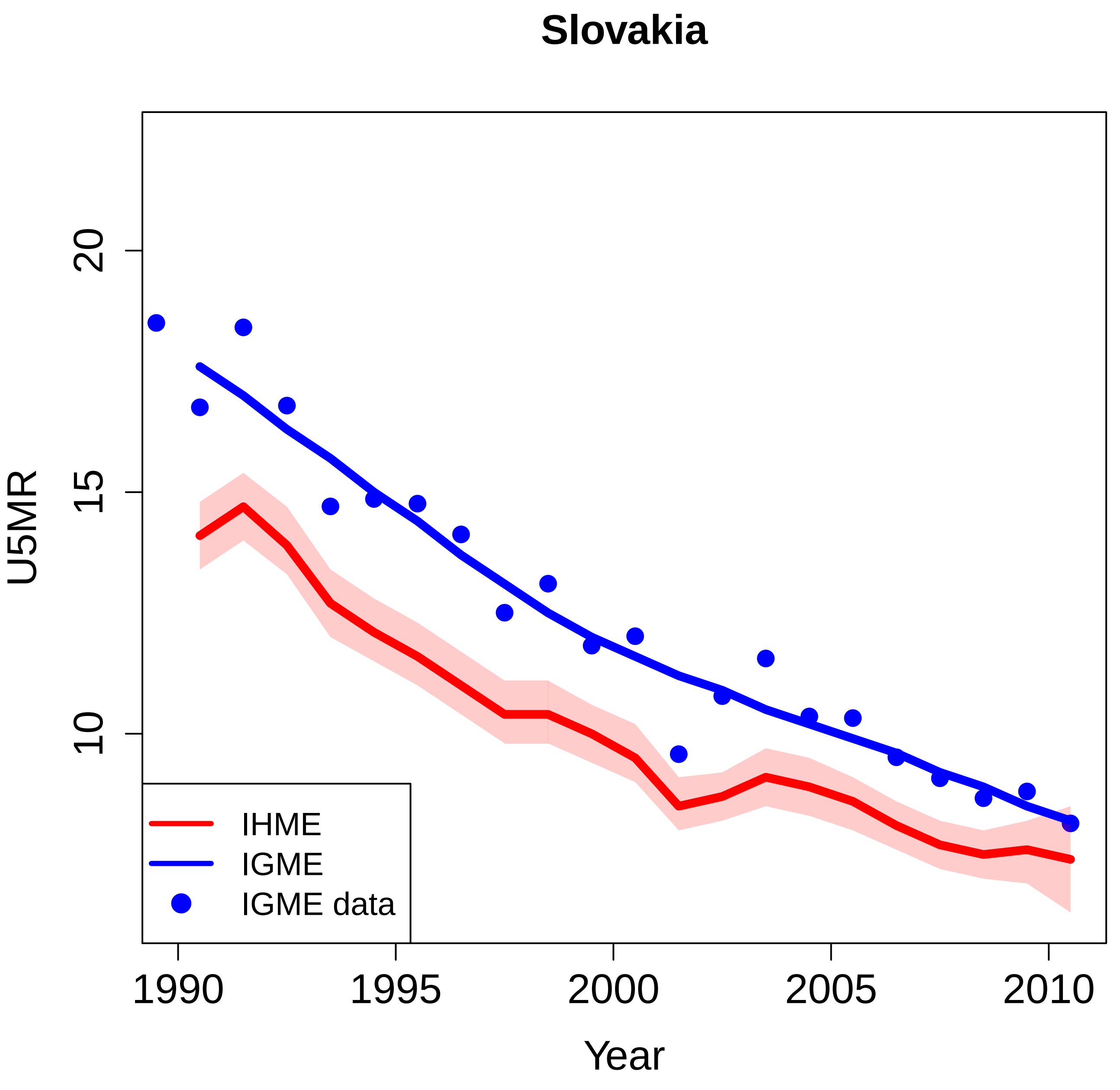
Slovakia
U5MR
20
15
10
1990
1995
2000
2005
2010
Year
IHME
IGME
IGME data

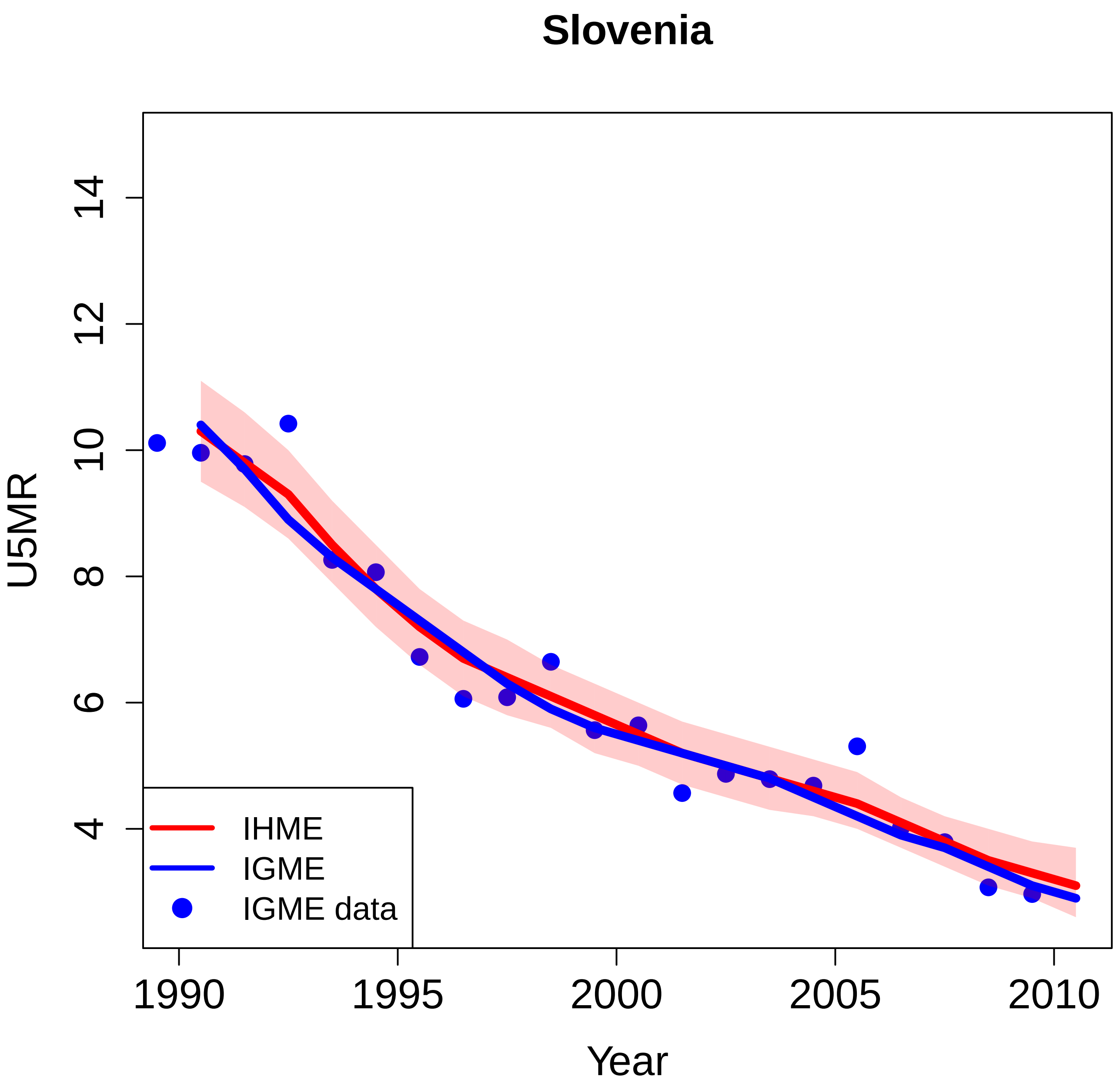
Slovenia
U5MR
4
6
8
10
12
14
1990
1995
2000
2005
2010
Year
IHME
IGME
IGME data

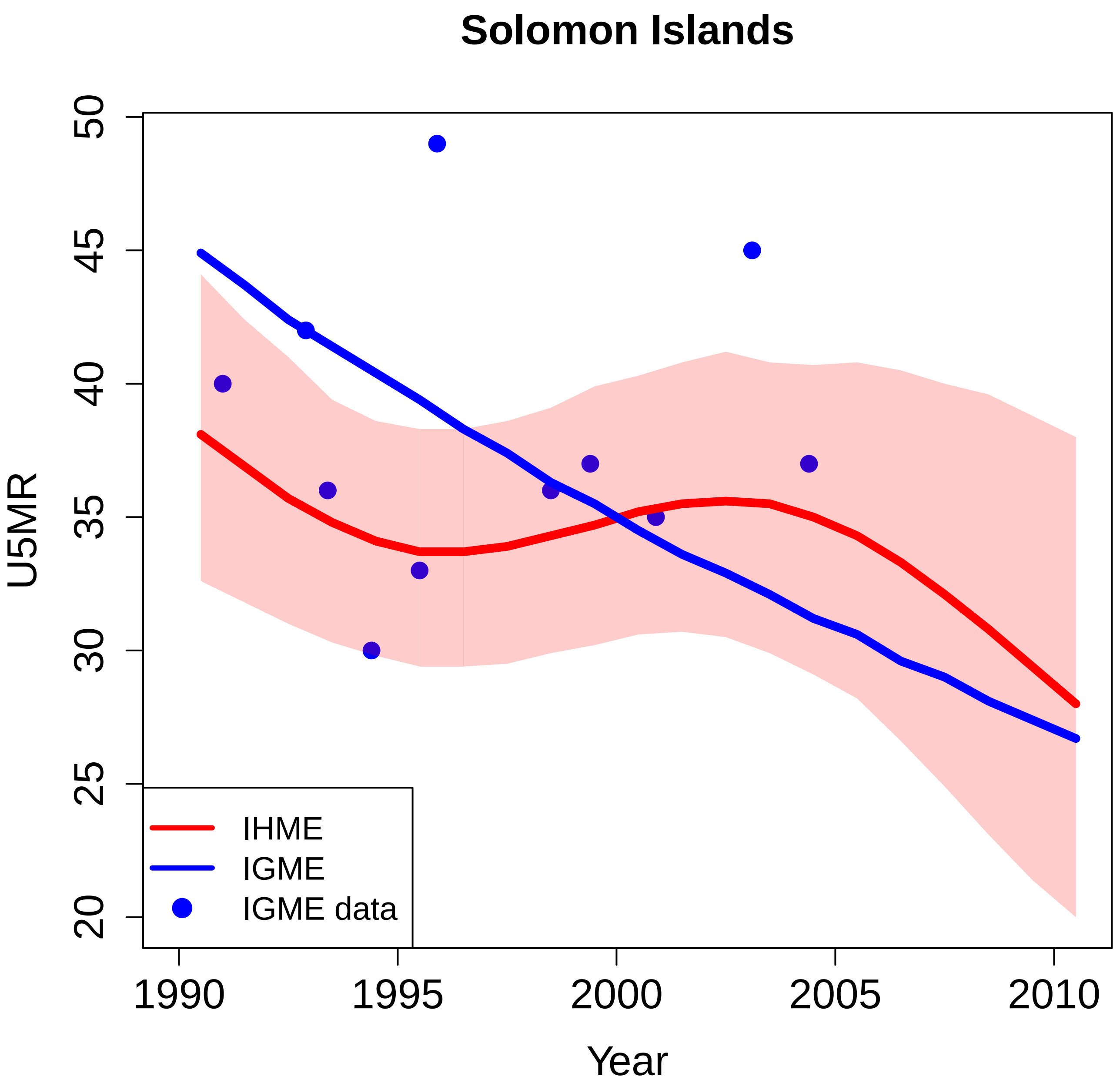
Solomon Islands
U5MR
50
45
40
35
30
25
20
1990
1995
2000
2005
2010
Year
IHME
IGME
IGME data

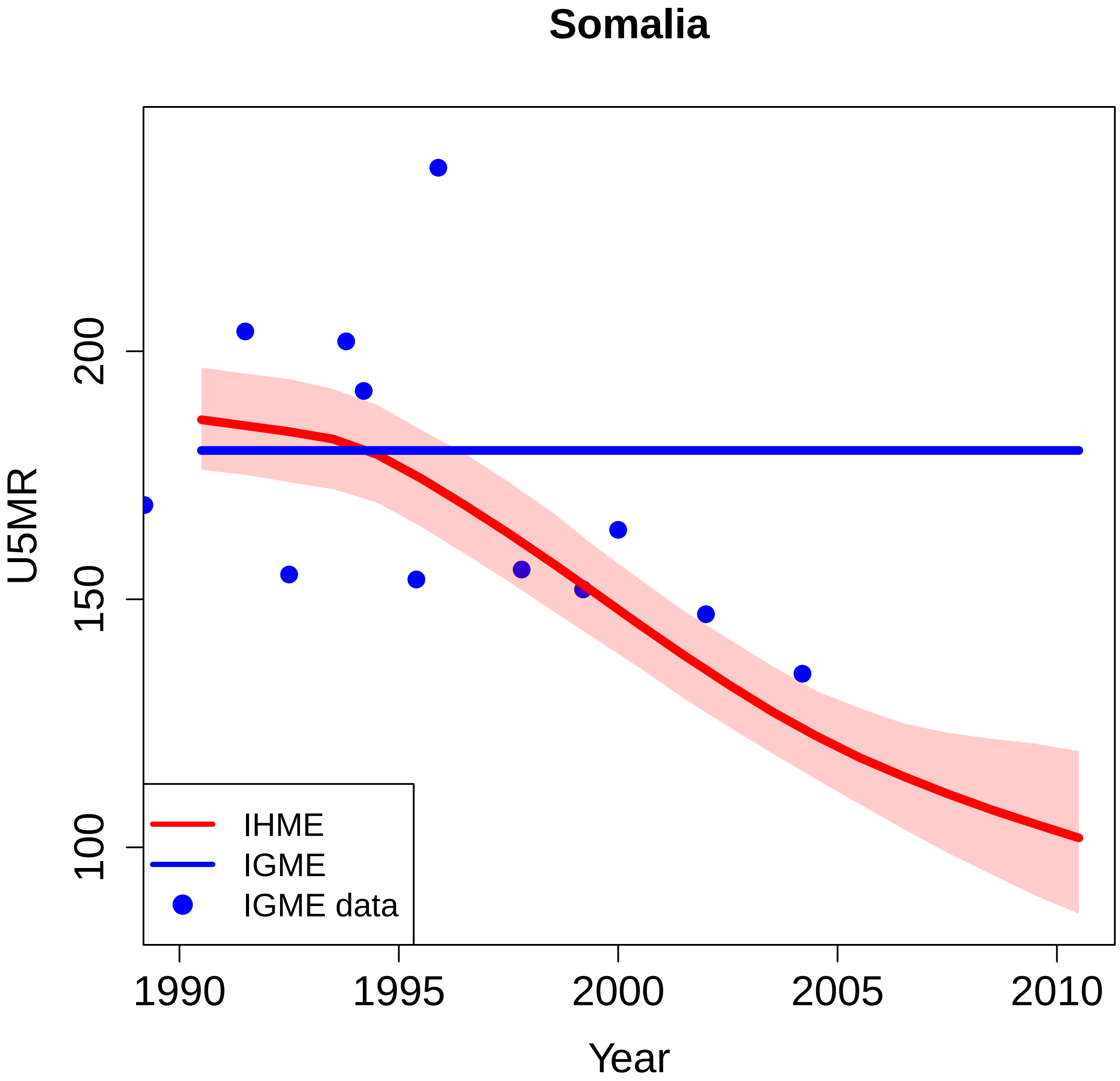
Somalia
U5MR
100
150
200
1990
1995
2000
2005
2010
Year
IHME
IGME
IGME data

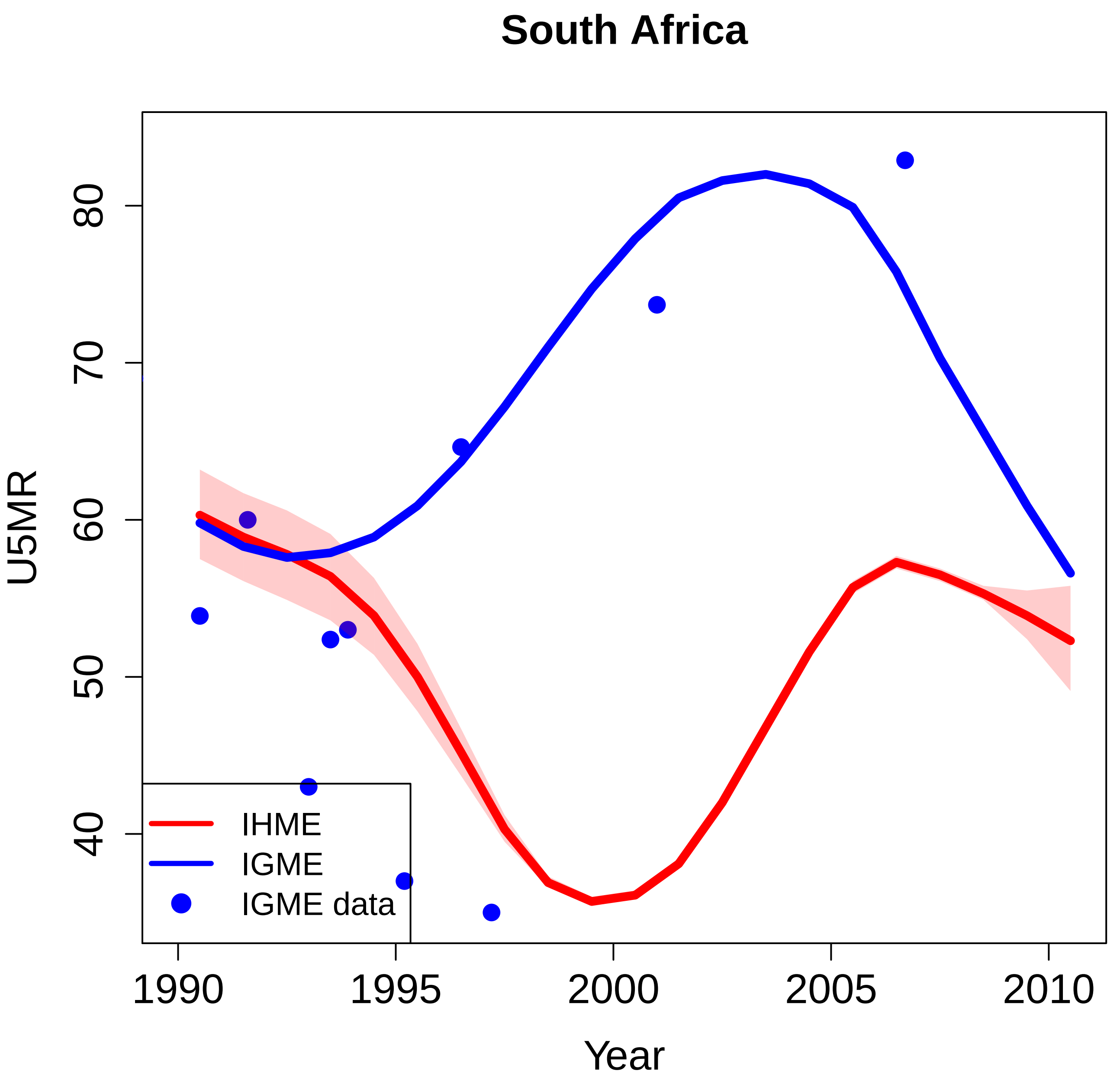
South Africa
U5MR
80
70
60
50
40
1990
1995
2000
2005
2010
Year
IHME
IGME
IGME data

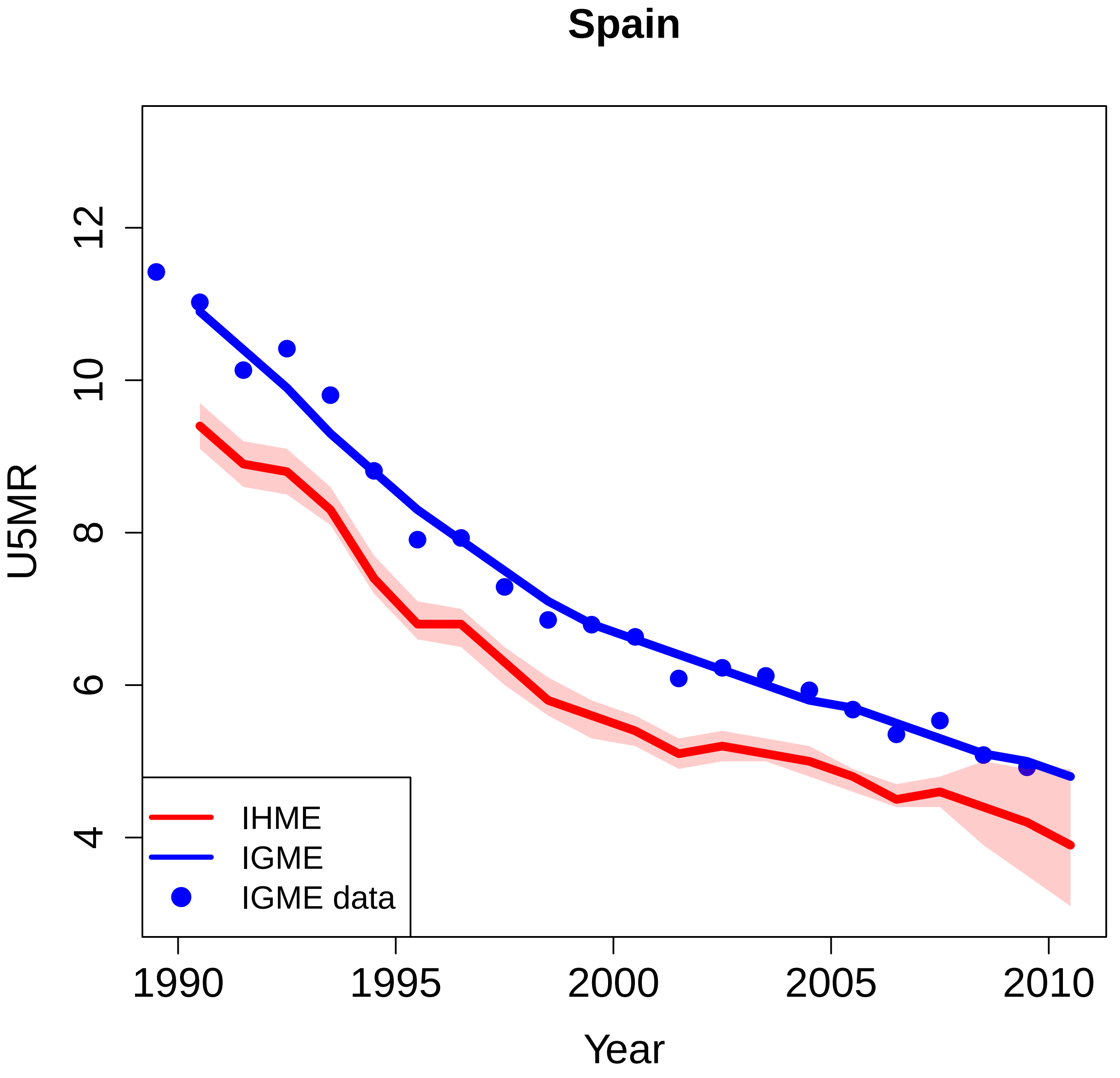
Spain
U5MR
12
10
8
6
4
1990
1995
2000
2005
2010
Year
IHME
IGME
IGME data

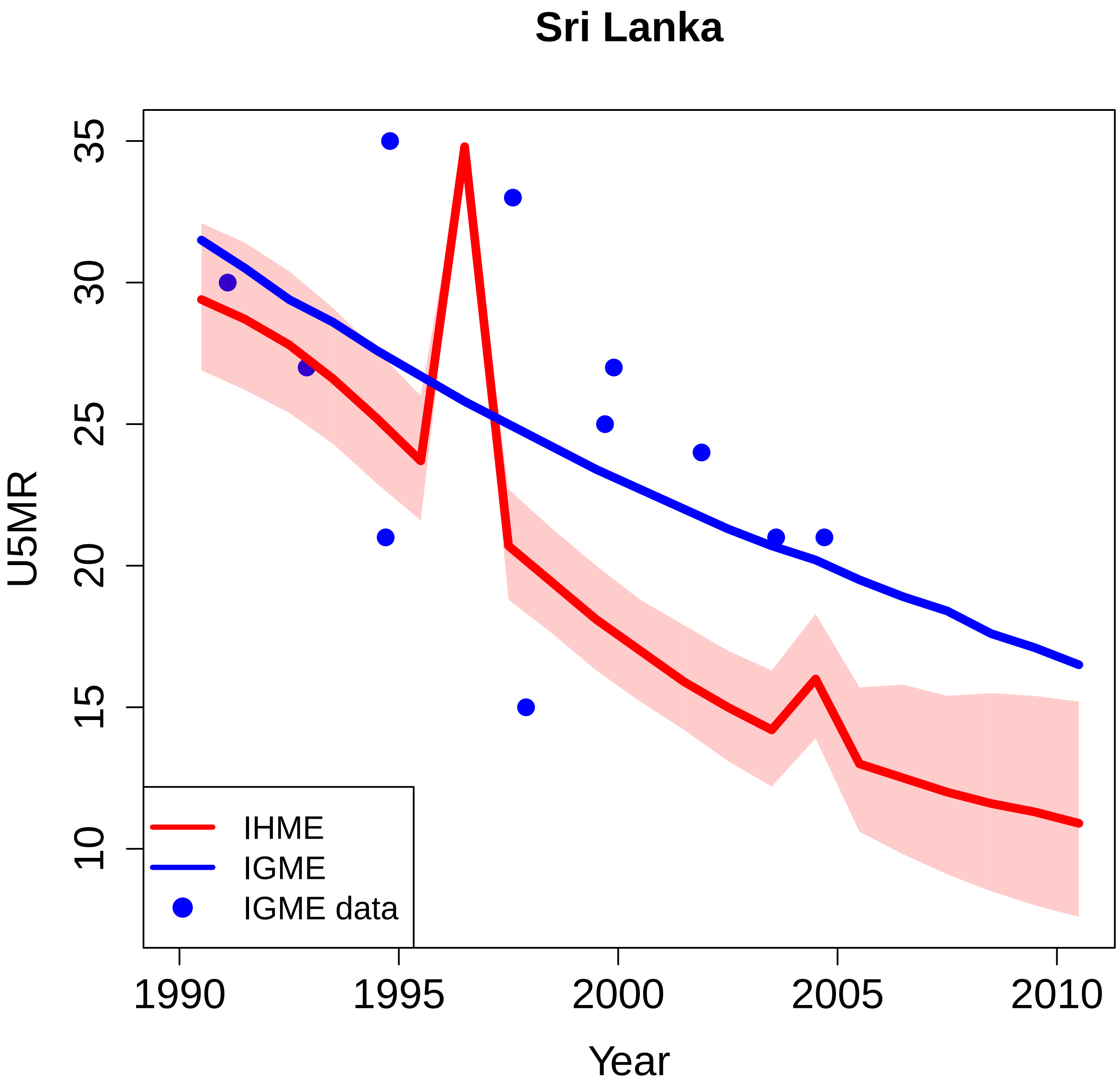

Sri Lanka
U5MR
10
15
20
25
30
35
1990
1995
2000
2005
2010
Year
IHME
IGME
IGME data

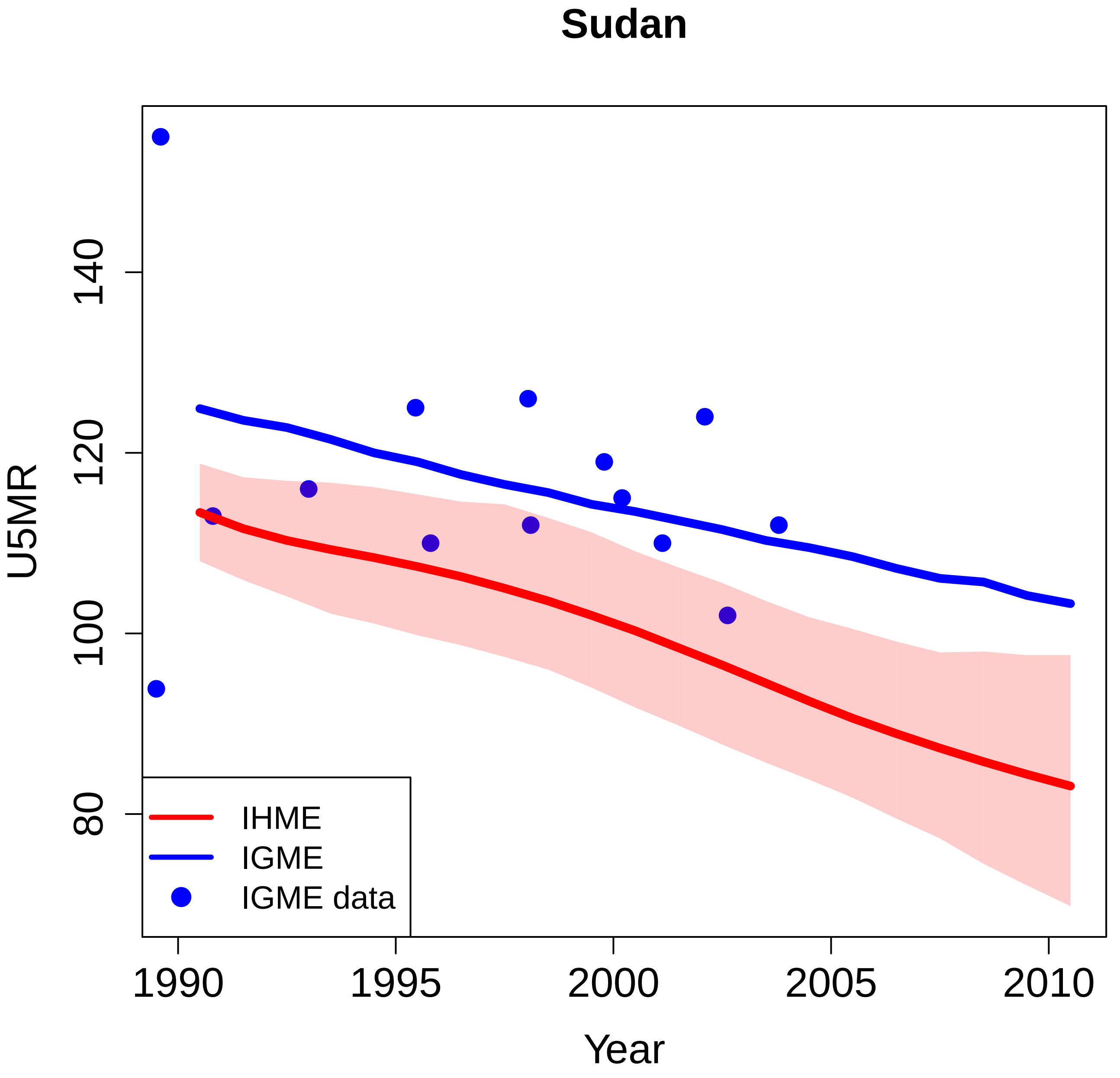
Sudan
U5MR
140
120
100
80
1990
1995
2000
2005
2010
Year
IHME
IGME
IGME data

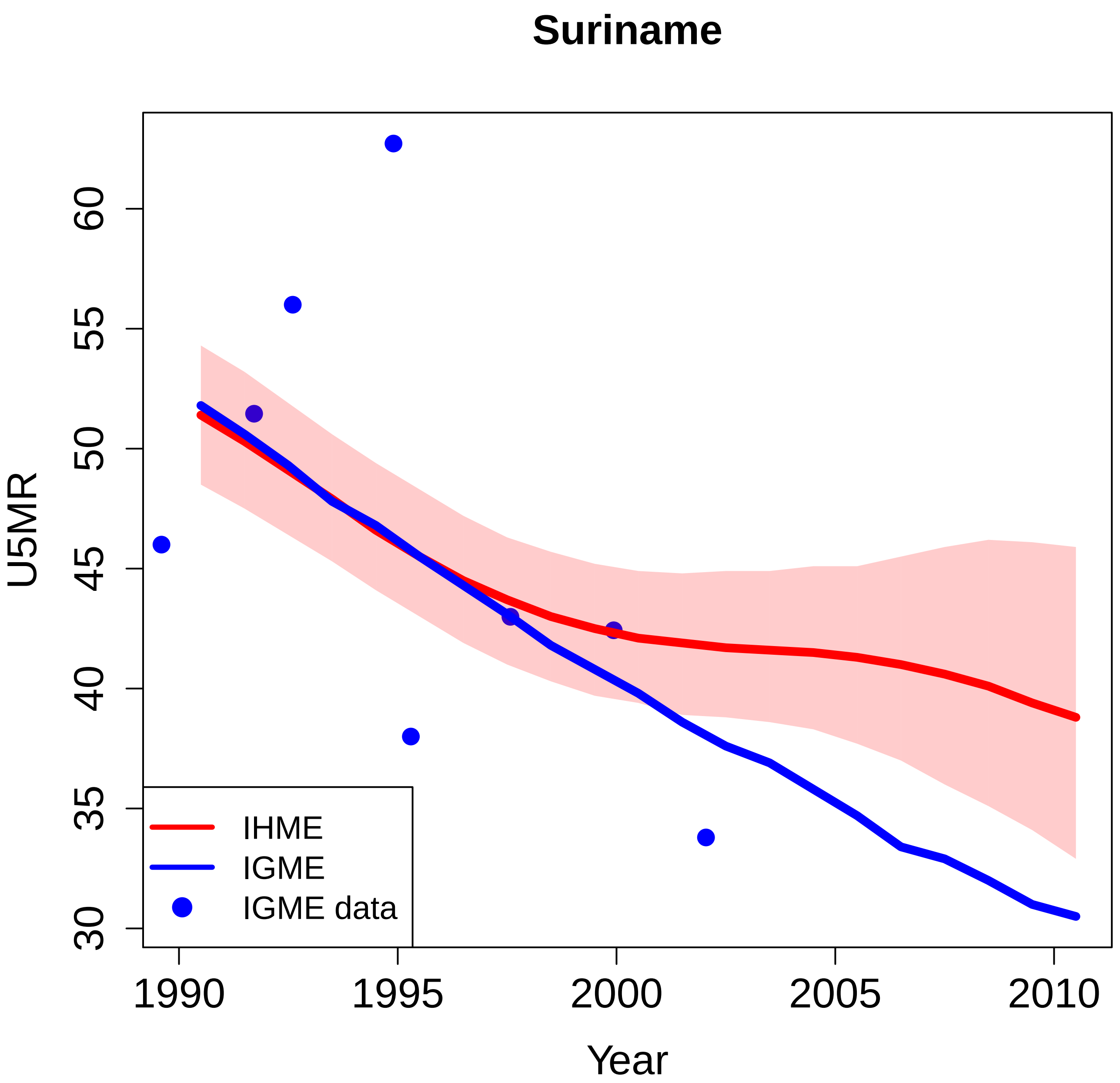
Suriname
U5MR
Year
30
35
40
45
50
55
60
1990
1995
2000
2005
2010
IHME
IGME
IGME data

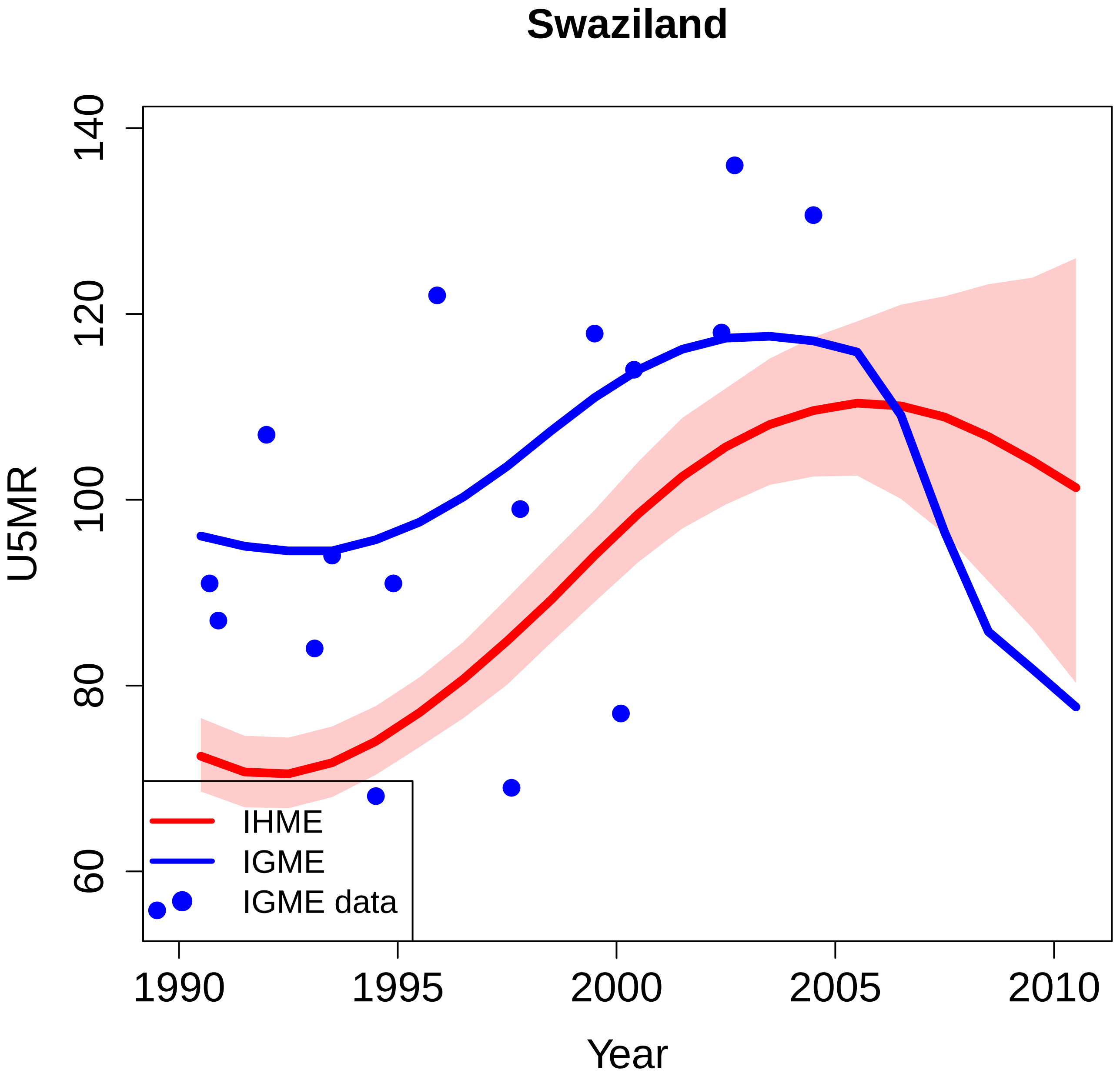

Swaziland
U5MR
140
120
100
80
60
1990
1995
2000
2005
2010
Year
IHME
IGME
IGME data

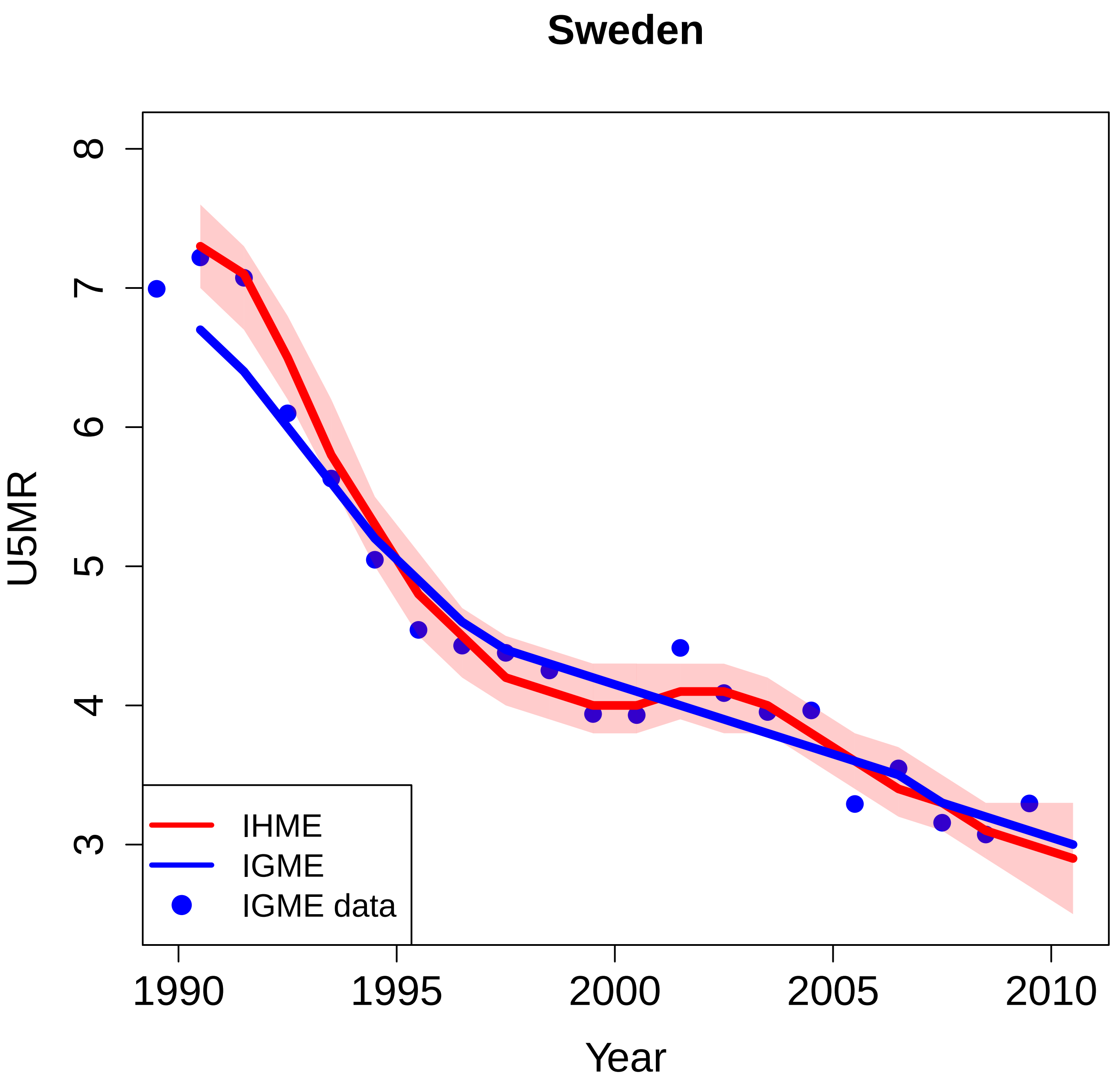
Sweden
U5MR
Year
8
7
6
5
4
3
1990
1995
2000
2005
2010
IHME
IGME
IGME data

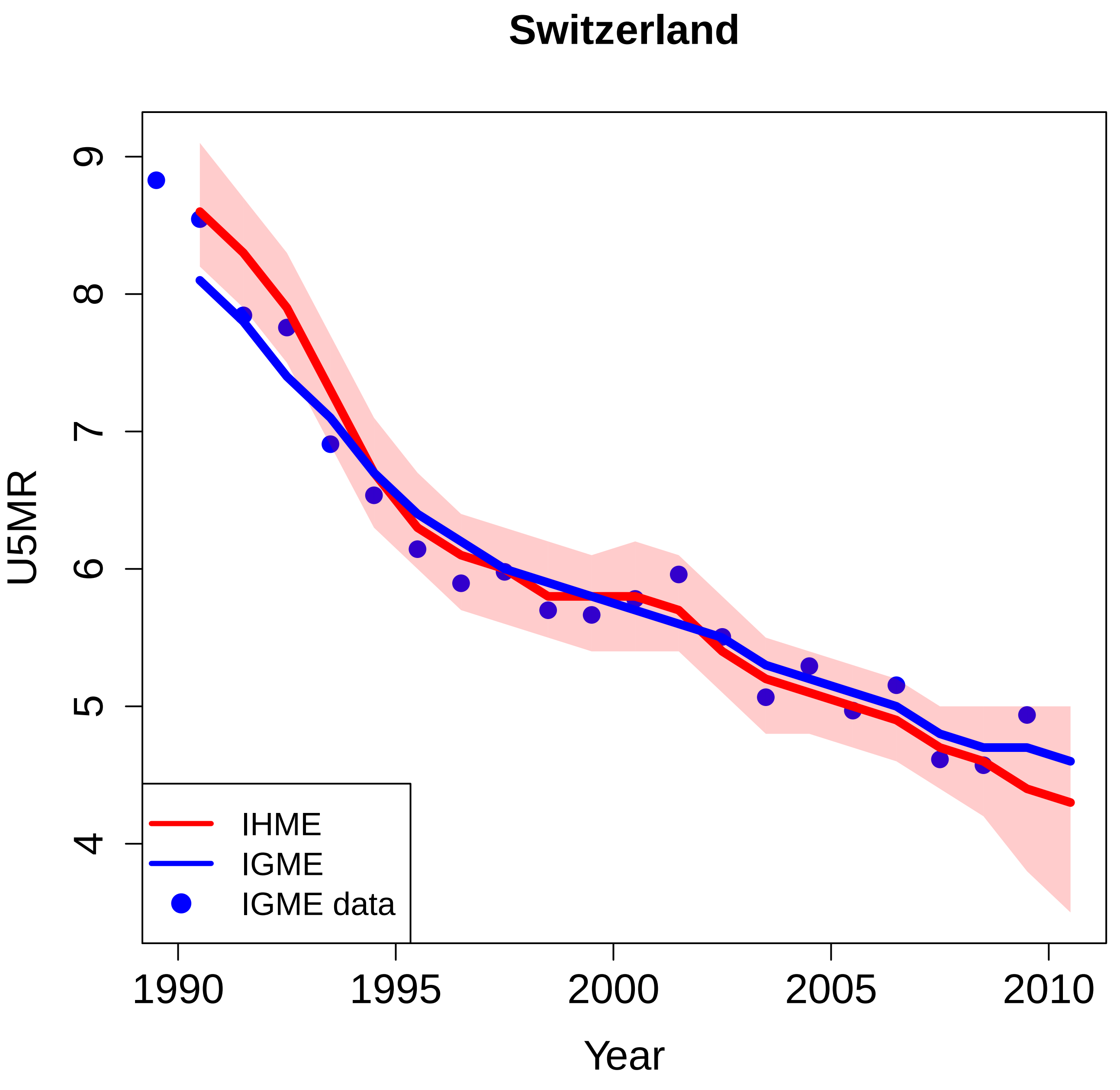
Switzerland
U5MR
Year
9
8
7
6
5
4
1990
1995
2000
2005
2010
IHME
IGME
IGME data

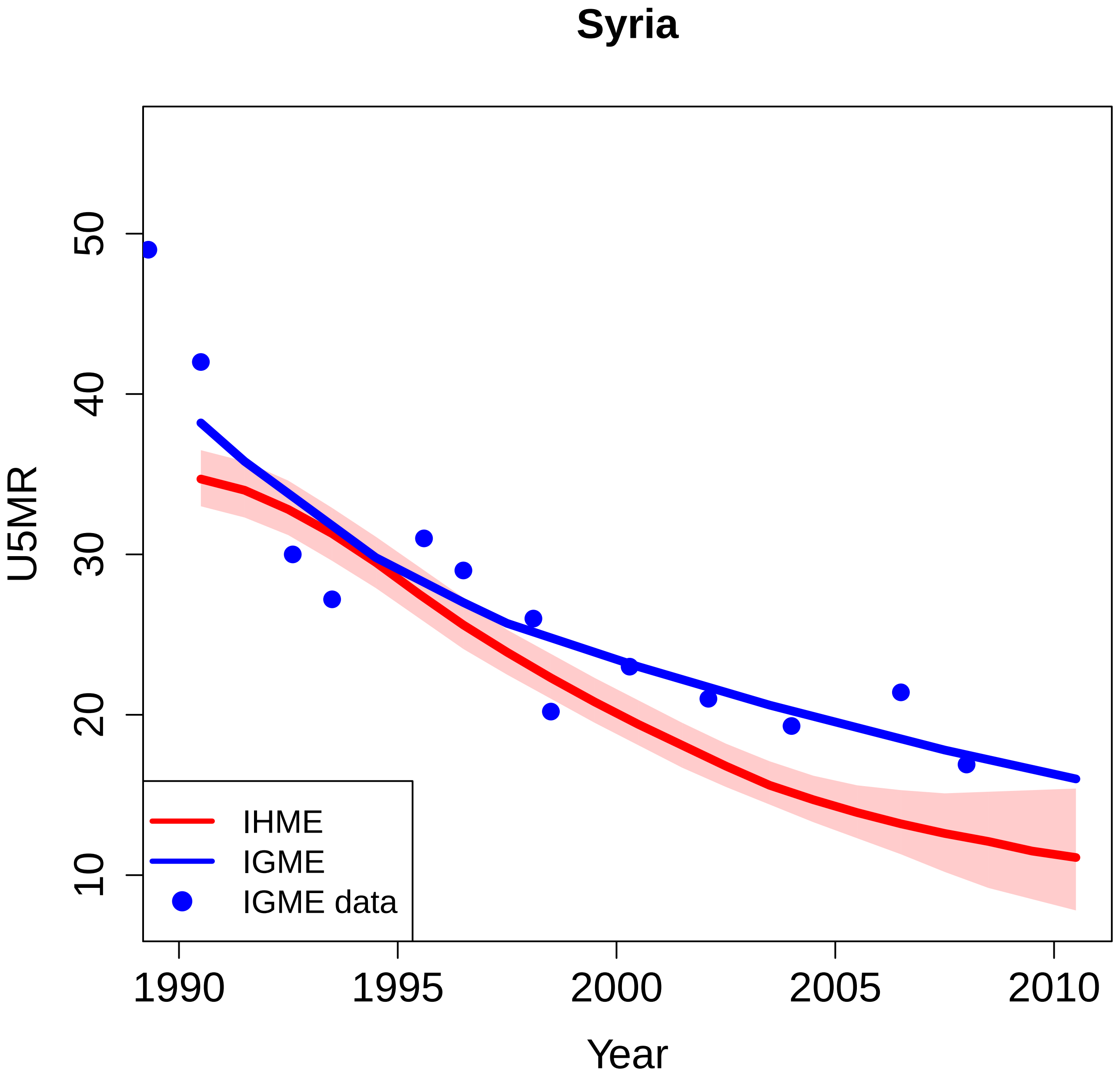
Syria
U5MR
50
40
30
20
10
1990
1995
2000
2005
2010
Year
IHME
IGME
IGME data

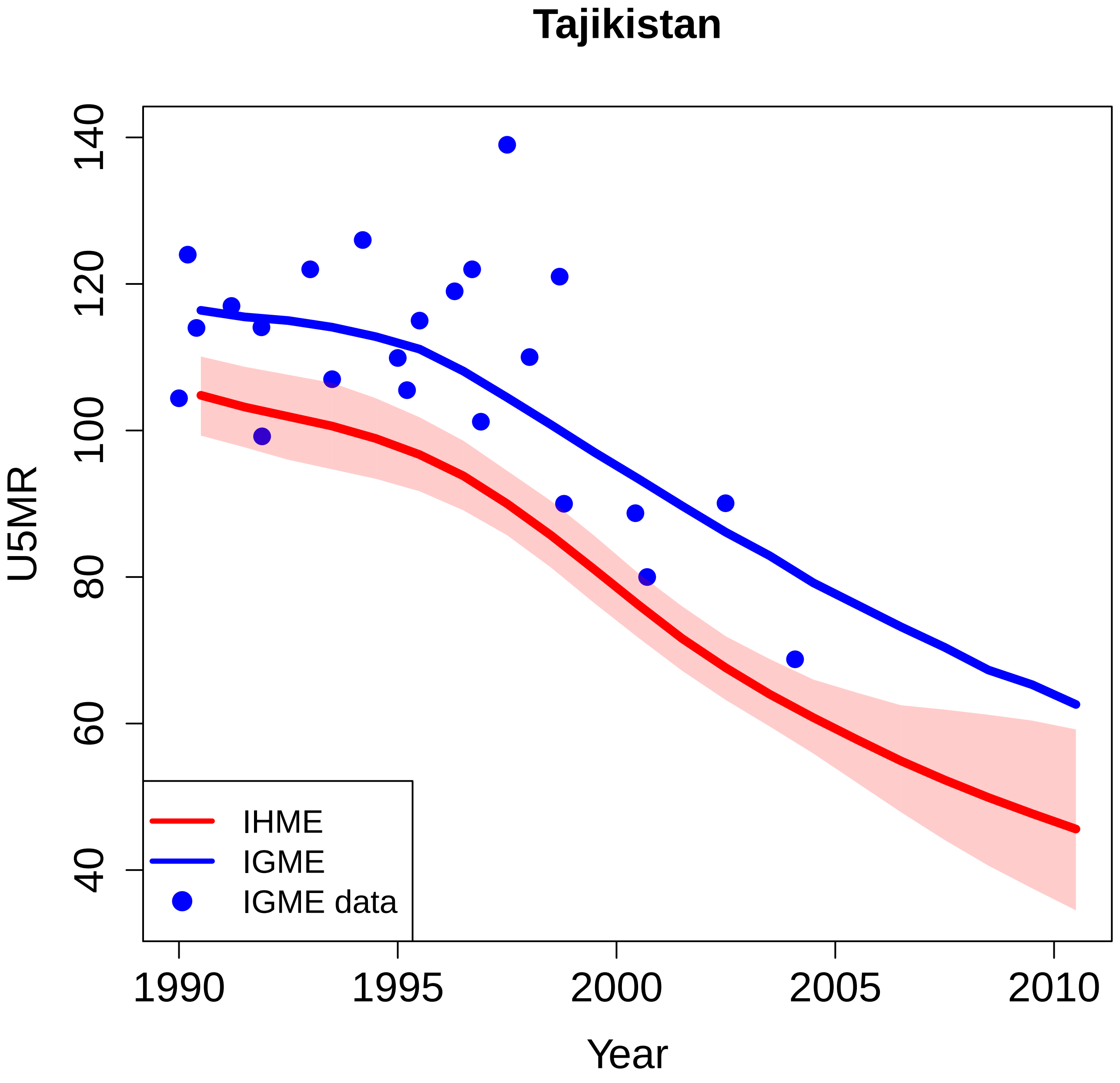
Tajikistan
U5MR
140
120
100
80
60
40
1990
1995
2000
2005
2010
Year
IHME
IGME
IGME data

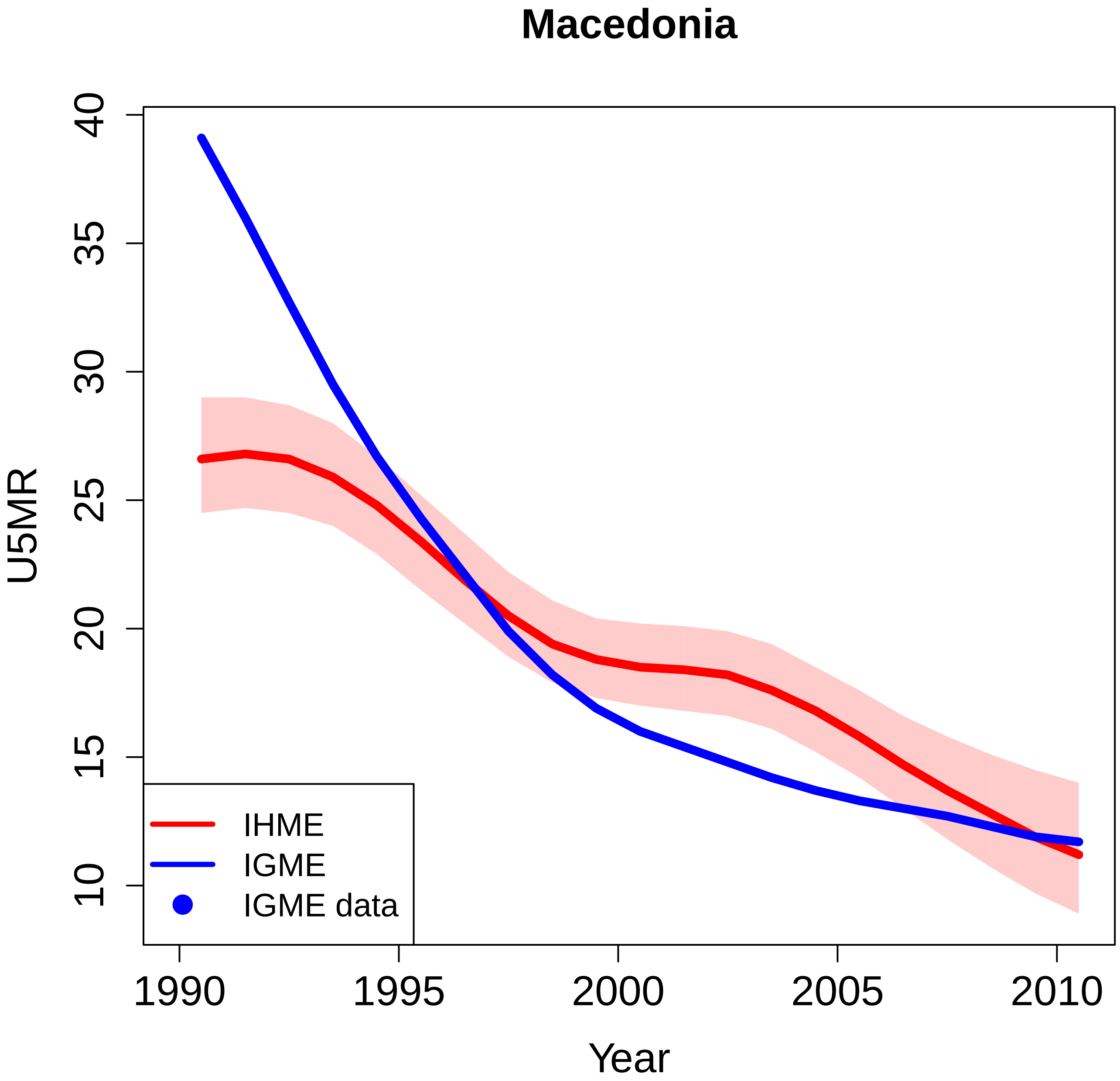

Macedonia
U5MR
40
35
30
25
20
15
10
1990
1995
2000
2005
2010
Year
IHME
IGME
IGME data

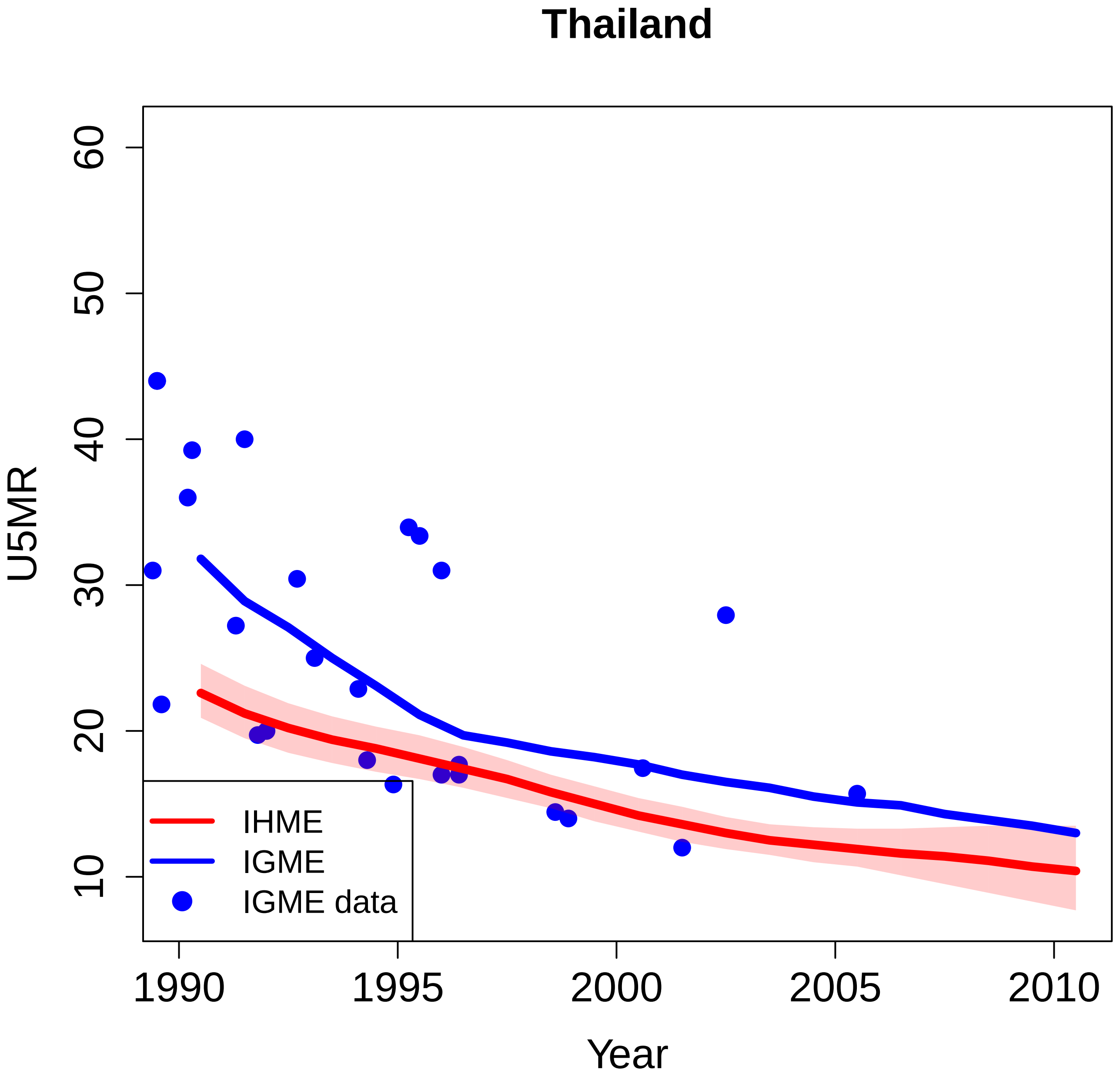

Thailand
U5MR
10
20
30
40
50
60
1990
1995
2000
2005
2010
Year
IHME
IGME
IGME data

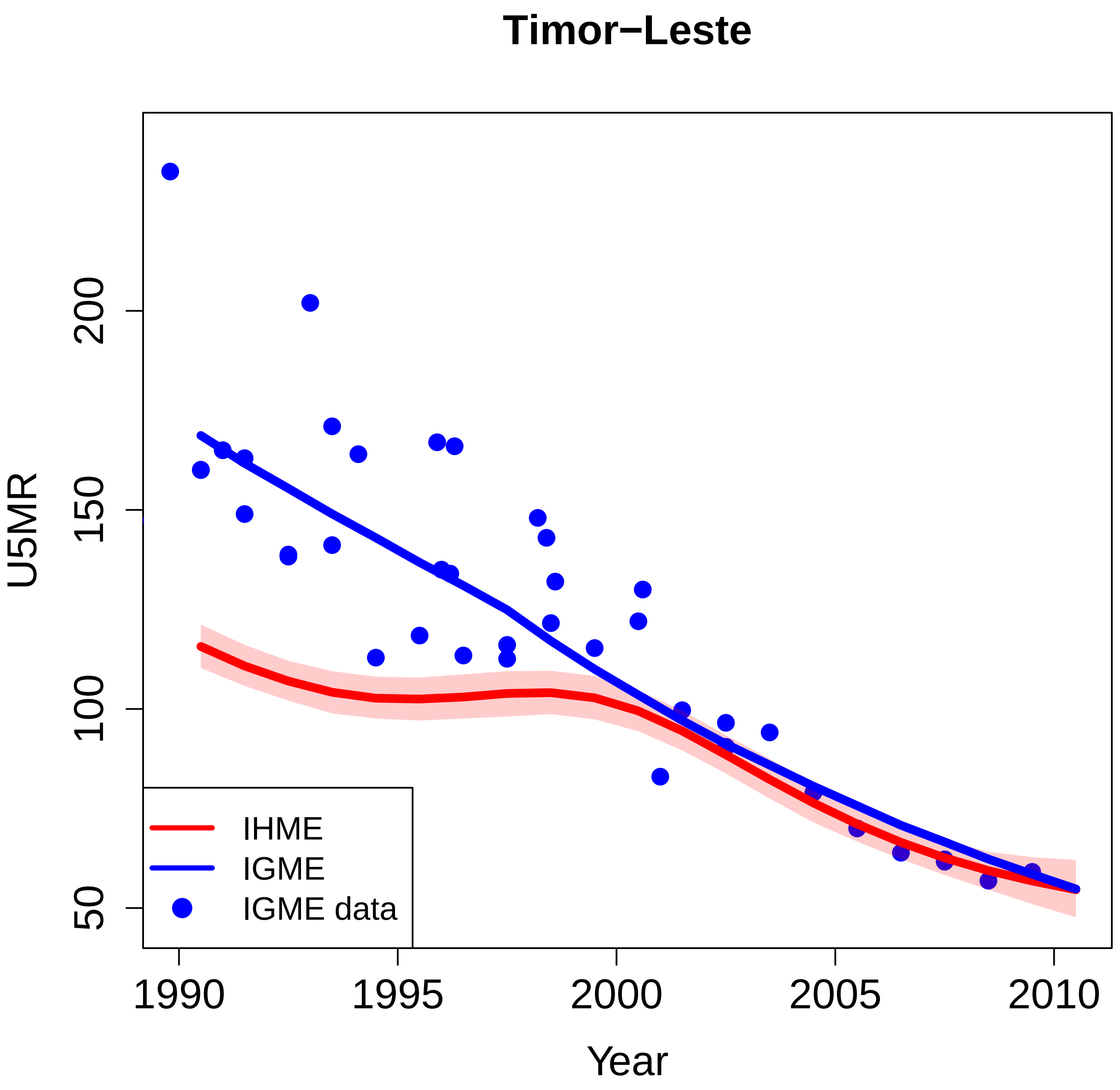

Timor−Leste
U5MR
Year
50
100
150
200
1990
1995
2000
2005
2010
IHME
IGME
IGME data

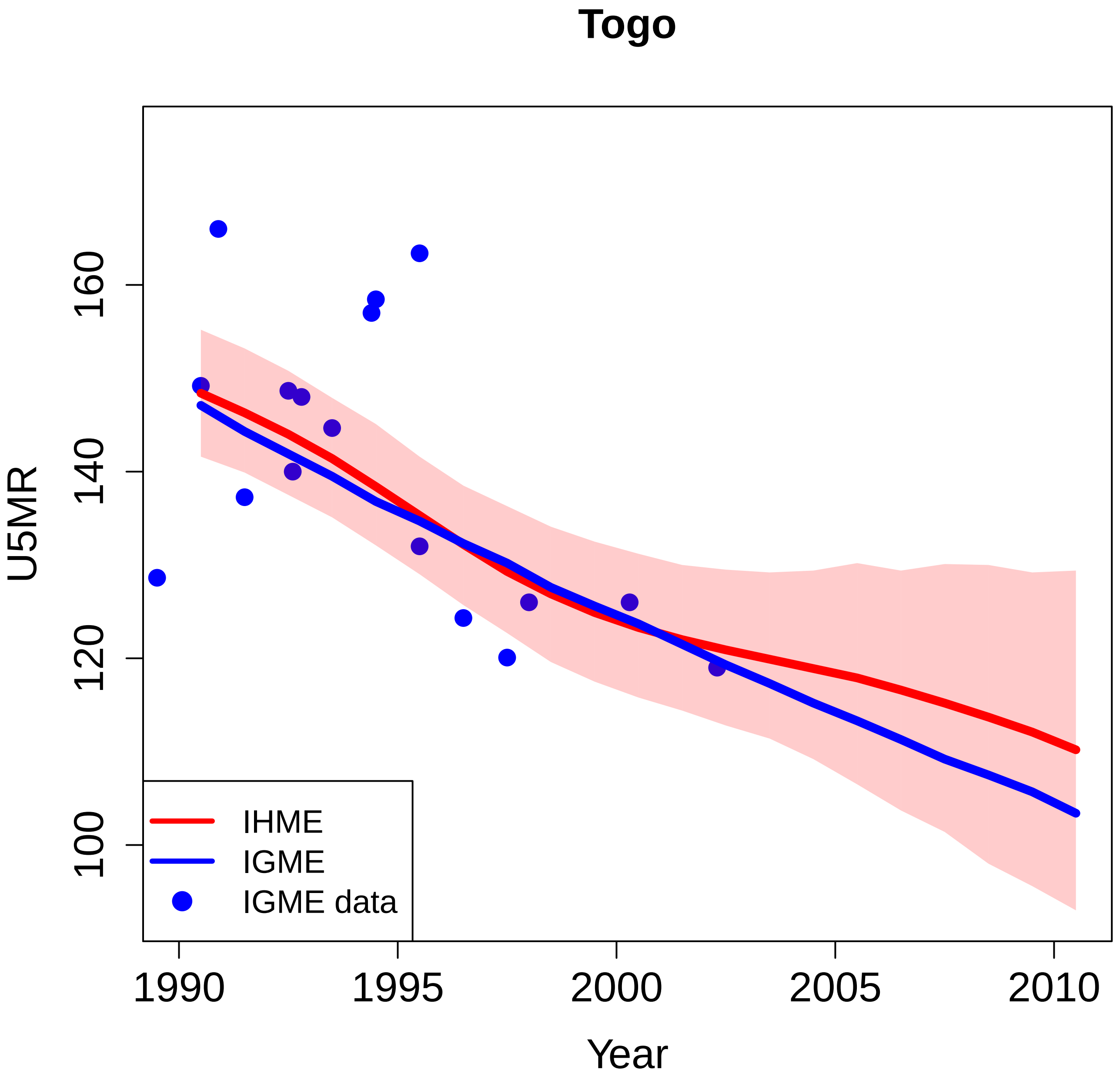

Togo
U5MR
160
140
120
100
1990
1995
2000
2005
2010
Year
IHME
IGME
IGME data

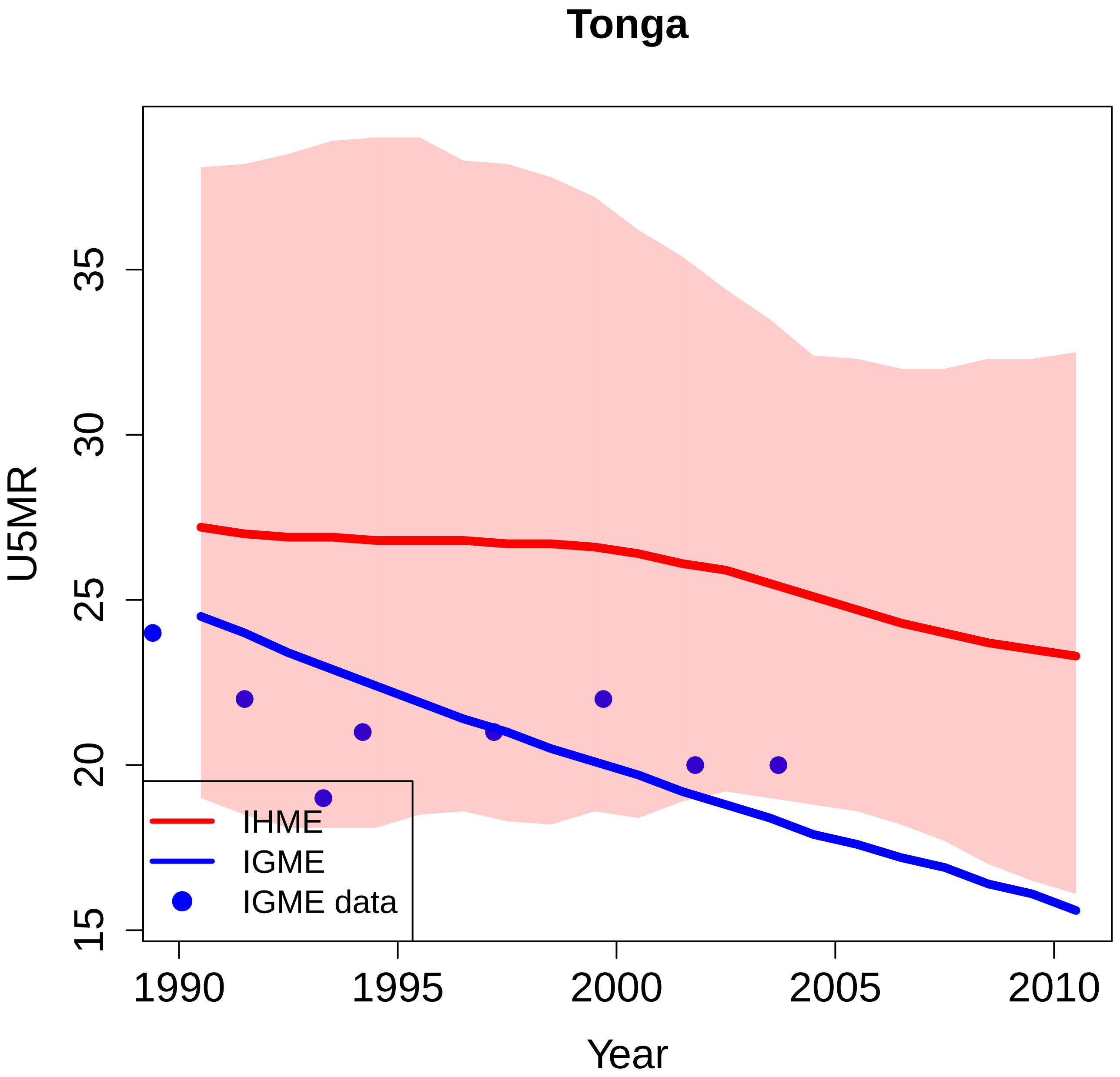

Tonga
U5MR
35
30
25
20
15
1990
1995
2000
2005
2010
Year
IHME
IGME
IGME data

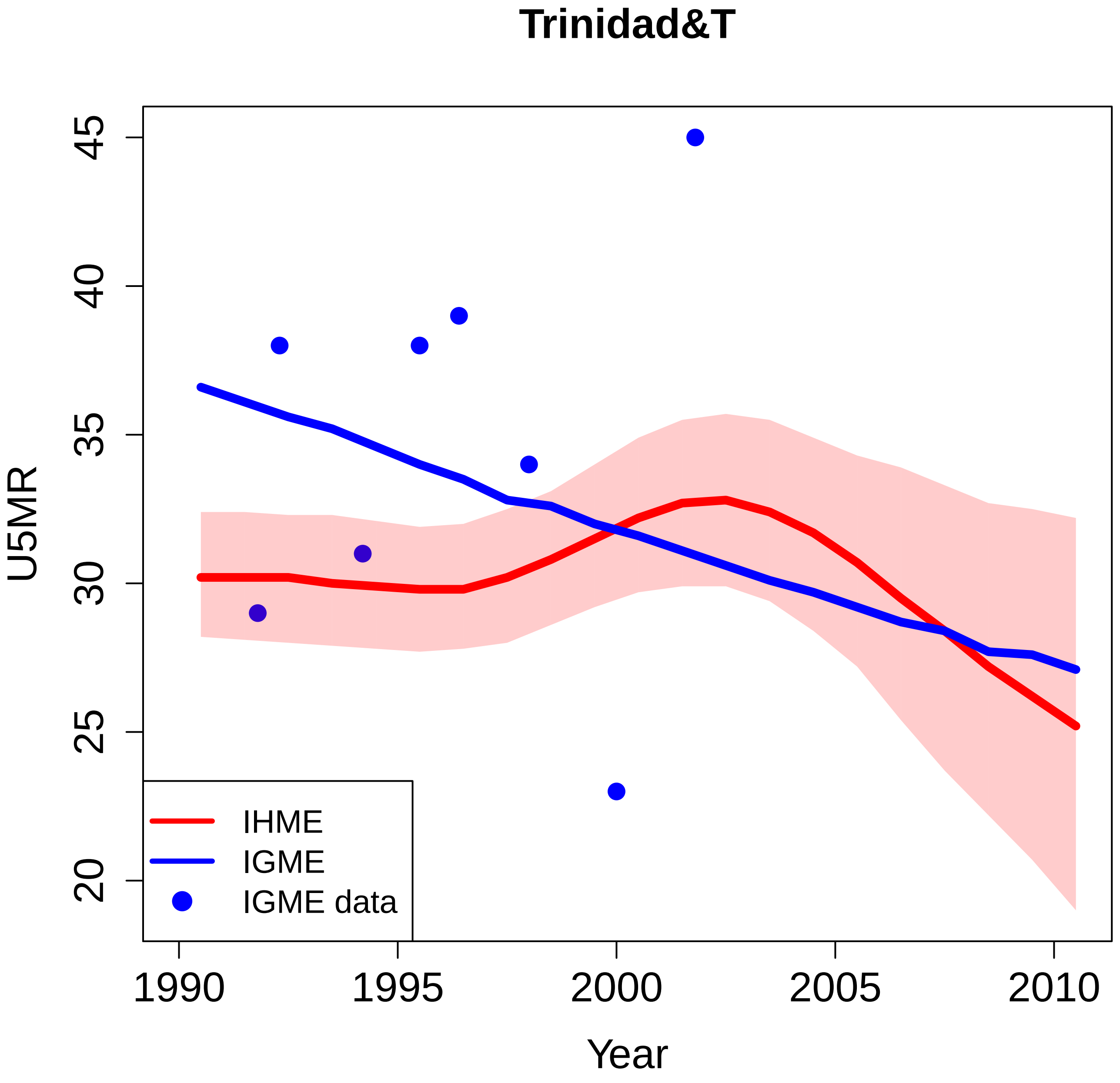

Trinidad&T
U5MR
45
40
35
30
25
20
1990
1995
2000
2005
2010
Year
IHME
IGME
IGME data

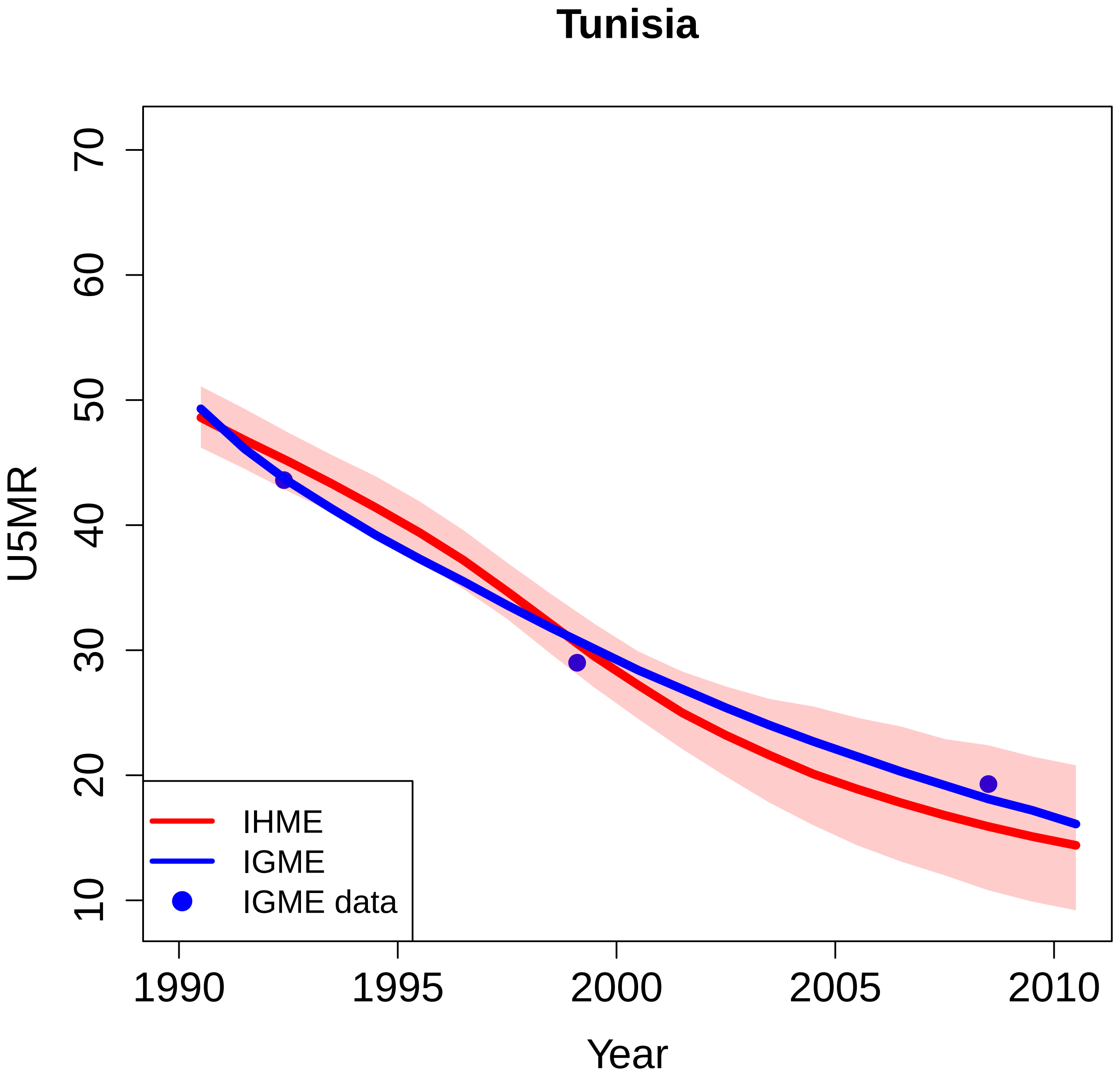
Tunisia
U5MR
70
60
50
40
30
20
10
1990
1995
2000
2005
2010
Year
IHME
IGME
IGME data

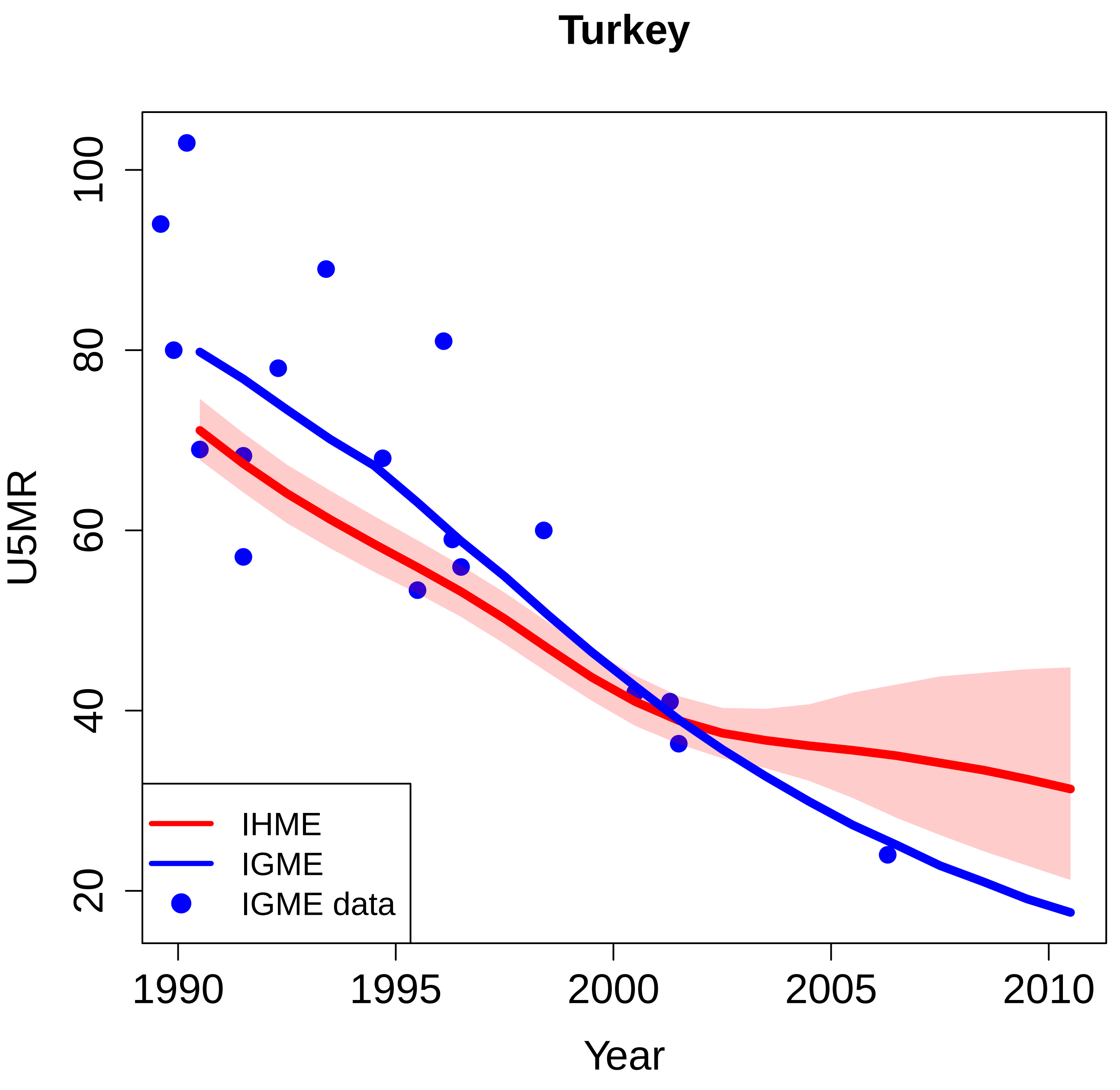

Turkey
U5MR
Year
20
40
60
80
100
1990
1995
2000
2005
2010
IHME
IGME
IGME data

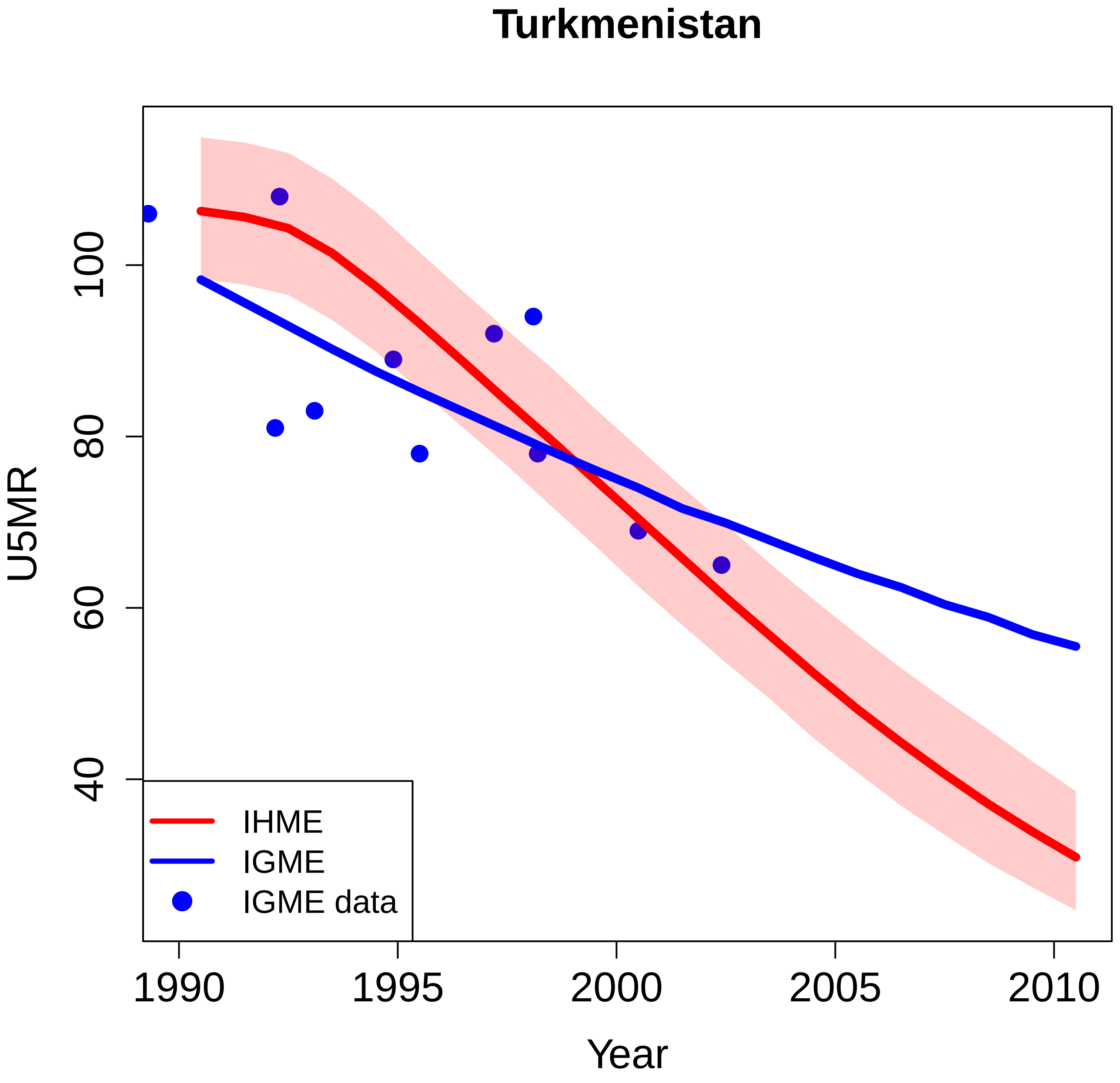

Turkmenistan
U5MR
Year
40
60
80
100
1990
1995
2000
2005
2010
IHME
IGME
IGME data

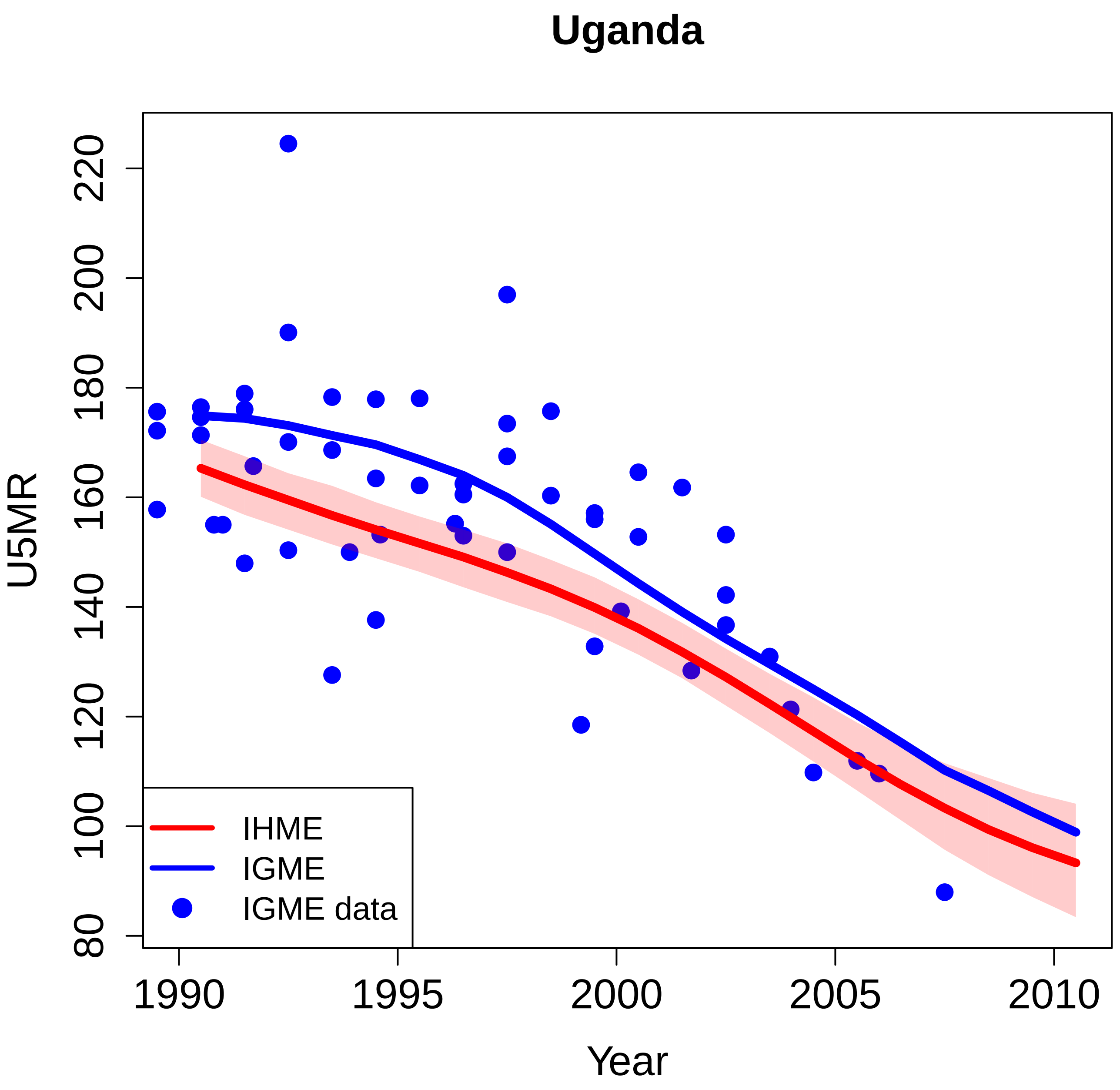

Uganda
220
200
180
160
140
120
100
80
U5MR
1990
1995
2000
2005
2010
Year
IHME
IGME
IGME data

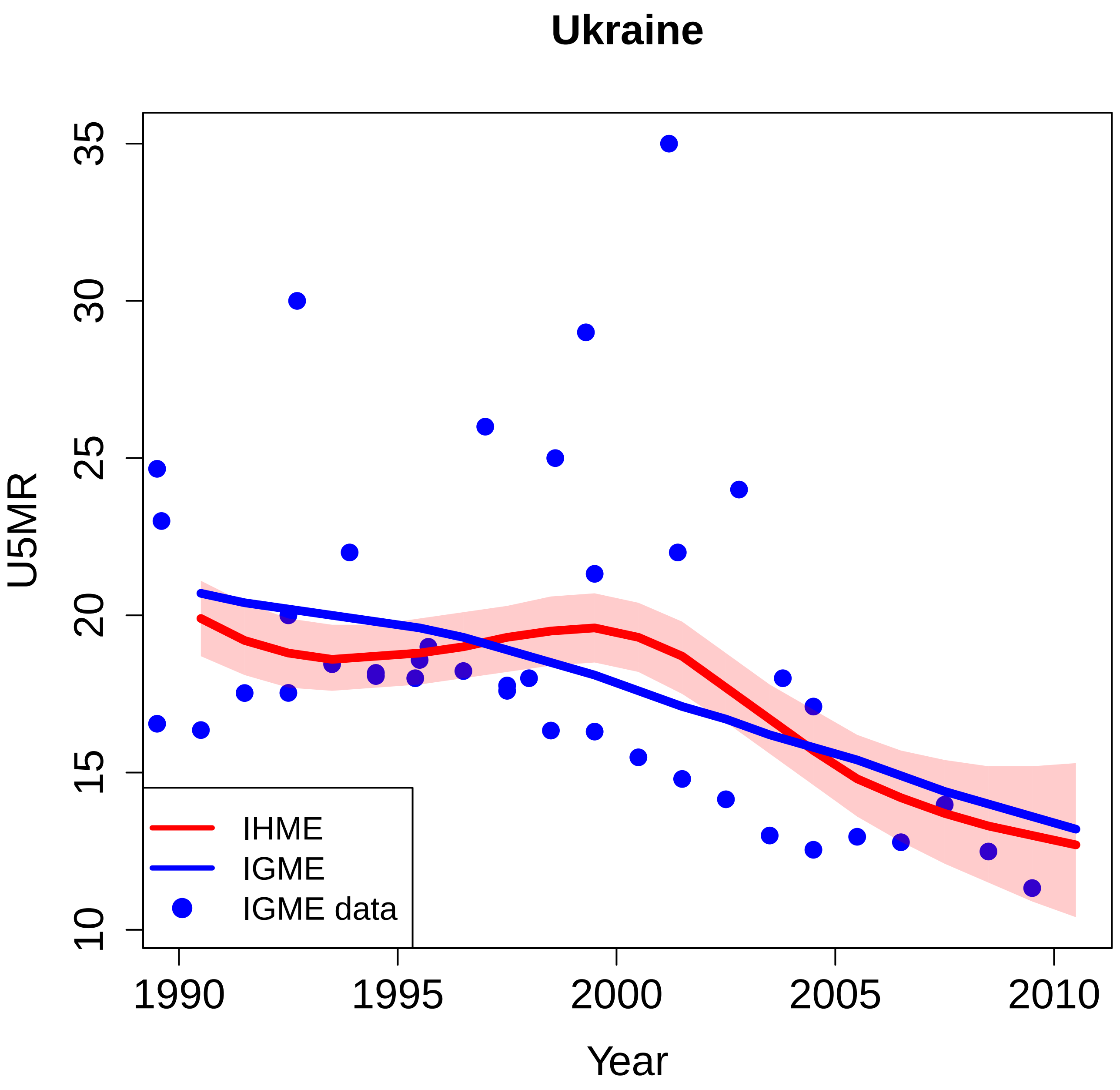
Ukraine
U5MR
Year
10
15
20
25
30
35
1990
1995
2000
2005
2010
IHME
IGME
IGME data

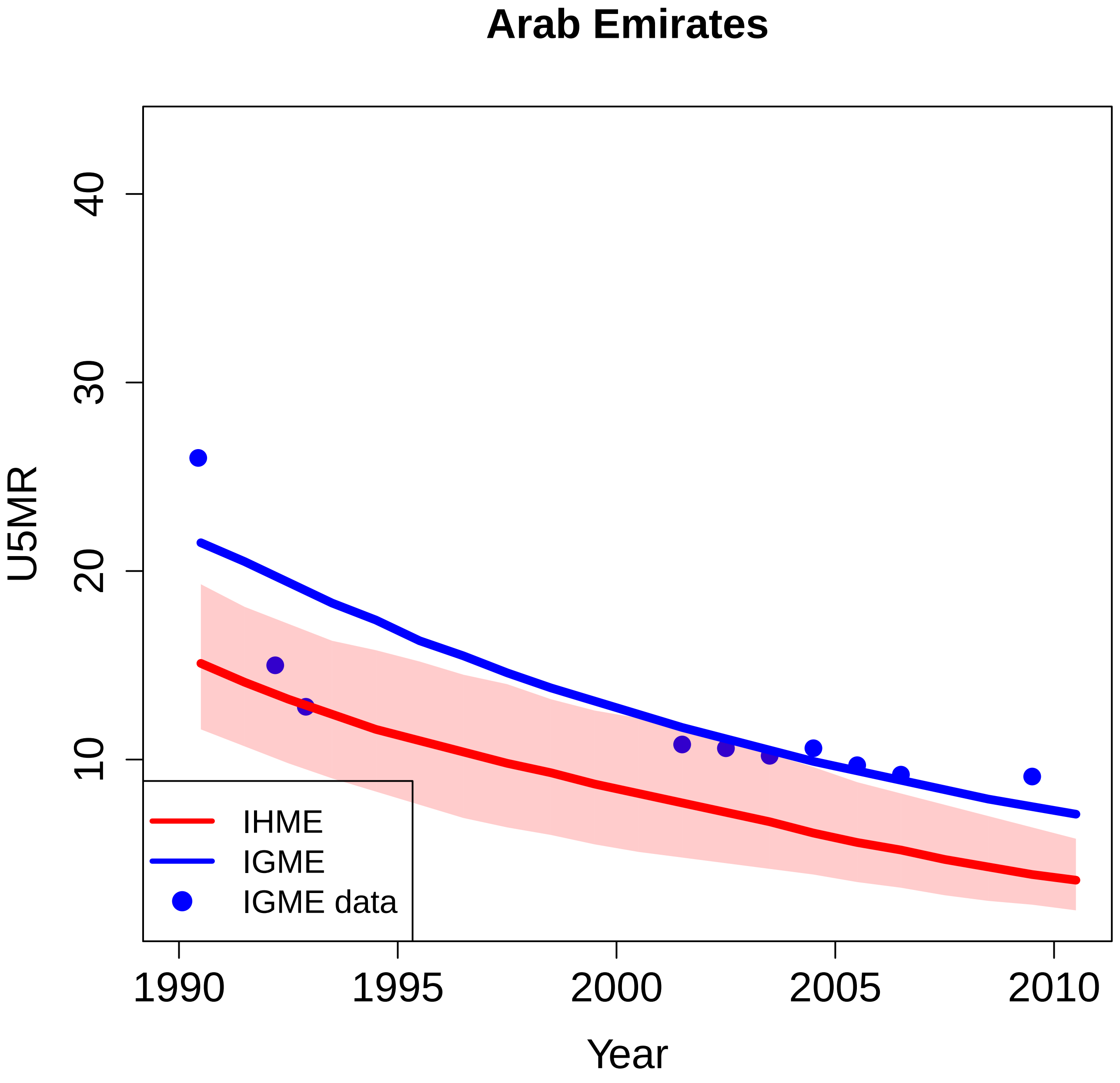
Arab Emirates
U5MR
40
30
20
10
1990
1995
2000
2005
2010
Year
IHME
IGME
IGME data

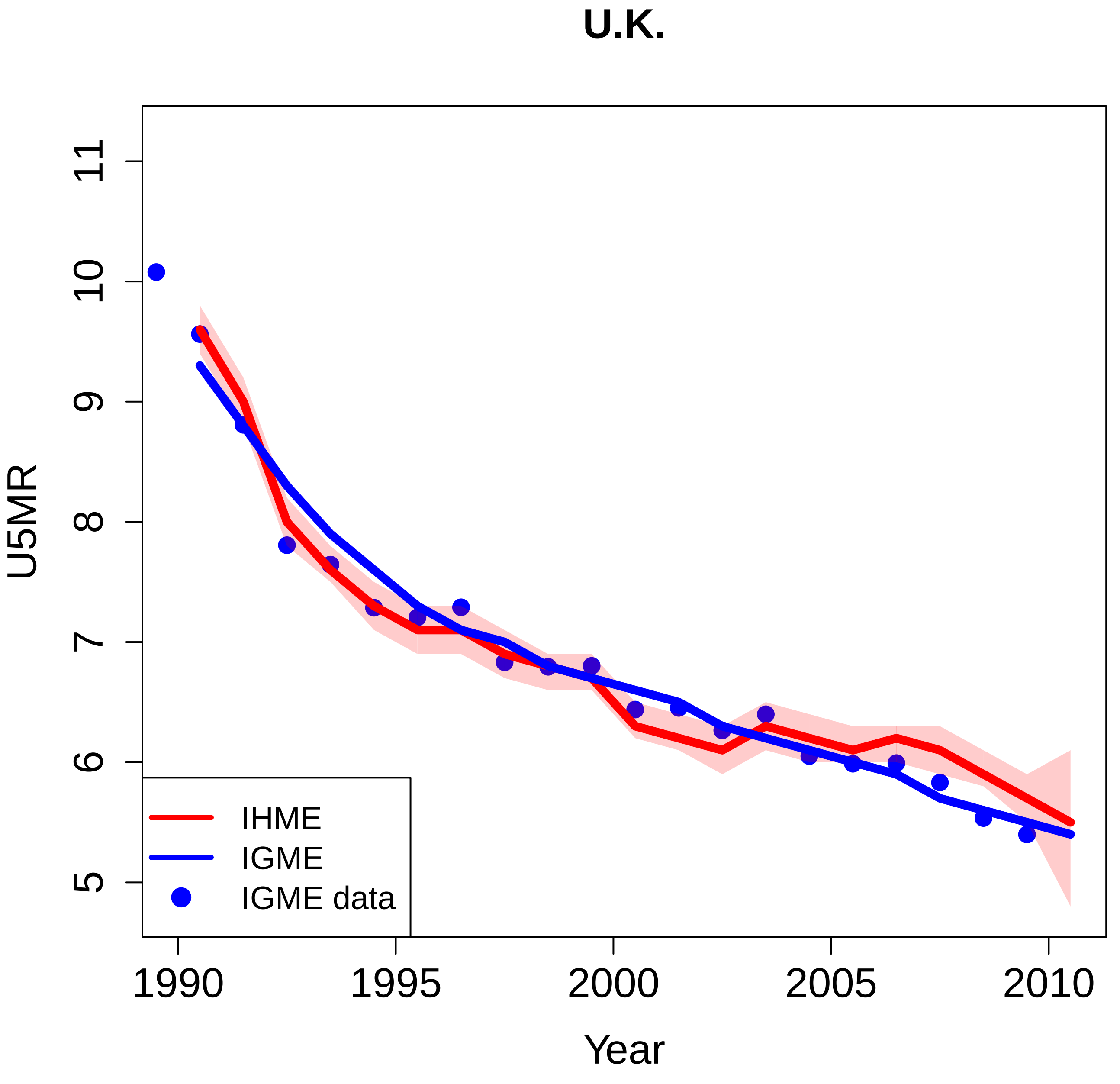
U.K.
U5MR
Year
IHME
IGME
IGME data
5
6
7
8
9
10
11
1990
1995
2000
2005
2010

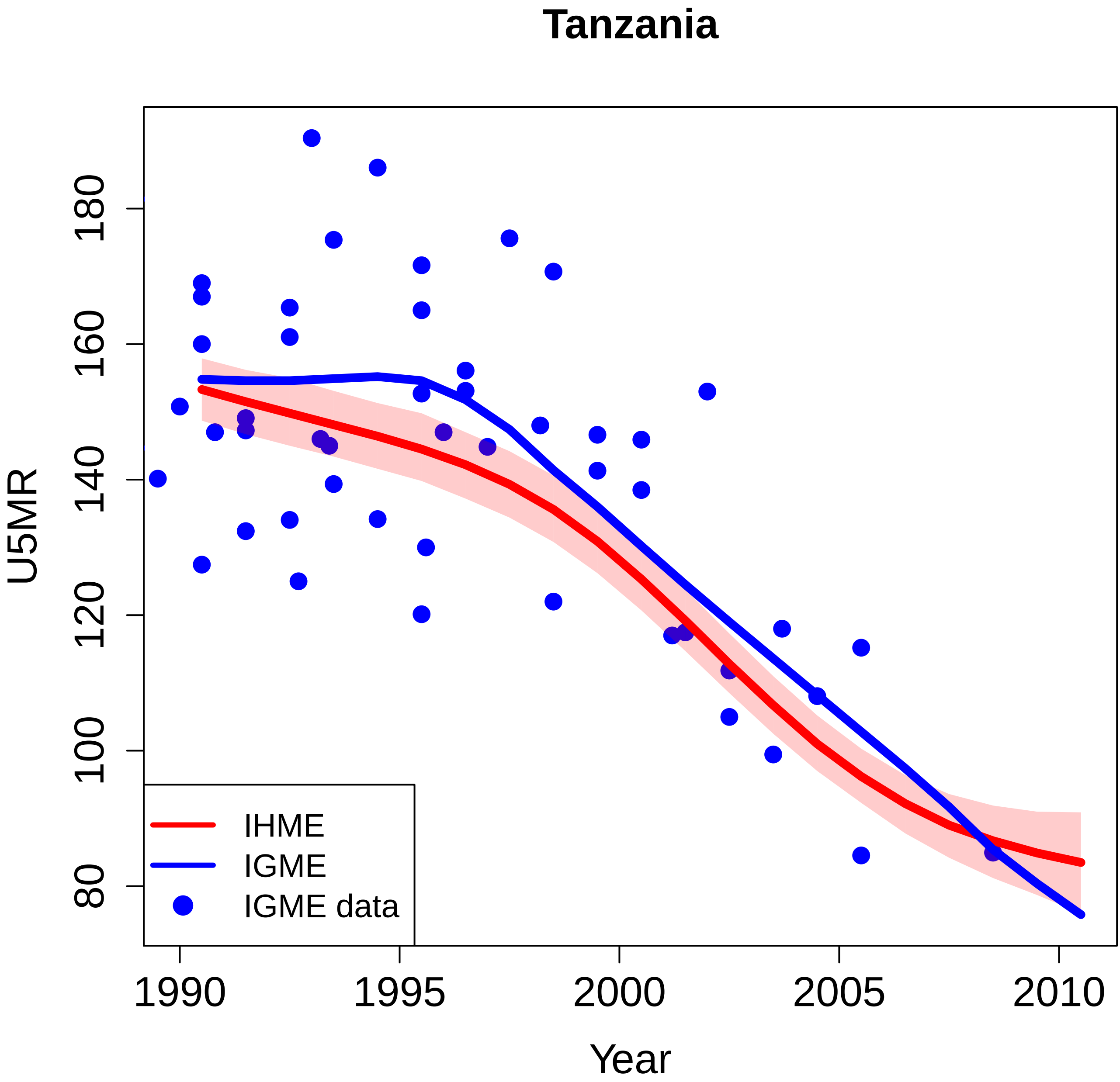
Tanzania
U5MR
Year
180
160
140
120
100
80
1990
1995
2000
2005
2010
IHME
IGME
IGME data

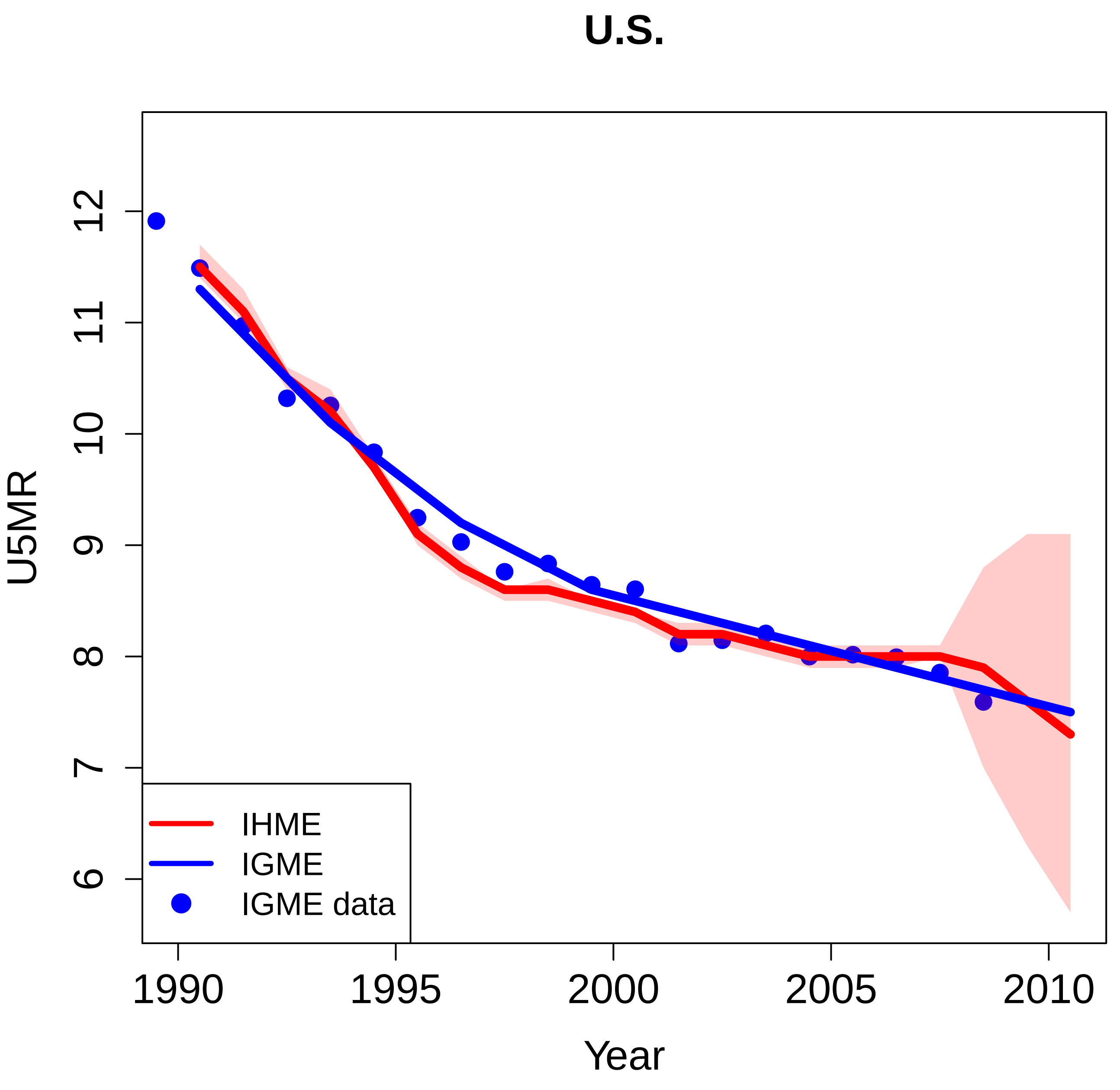
U.S.
U5MR
Year
1990
1995
2000
2005
2010
6
7
8
9
10
11
12
IHME
IGME
IGME data

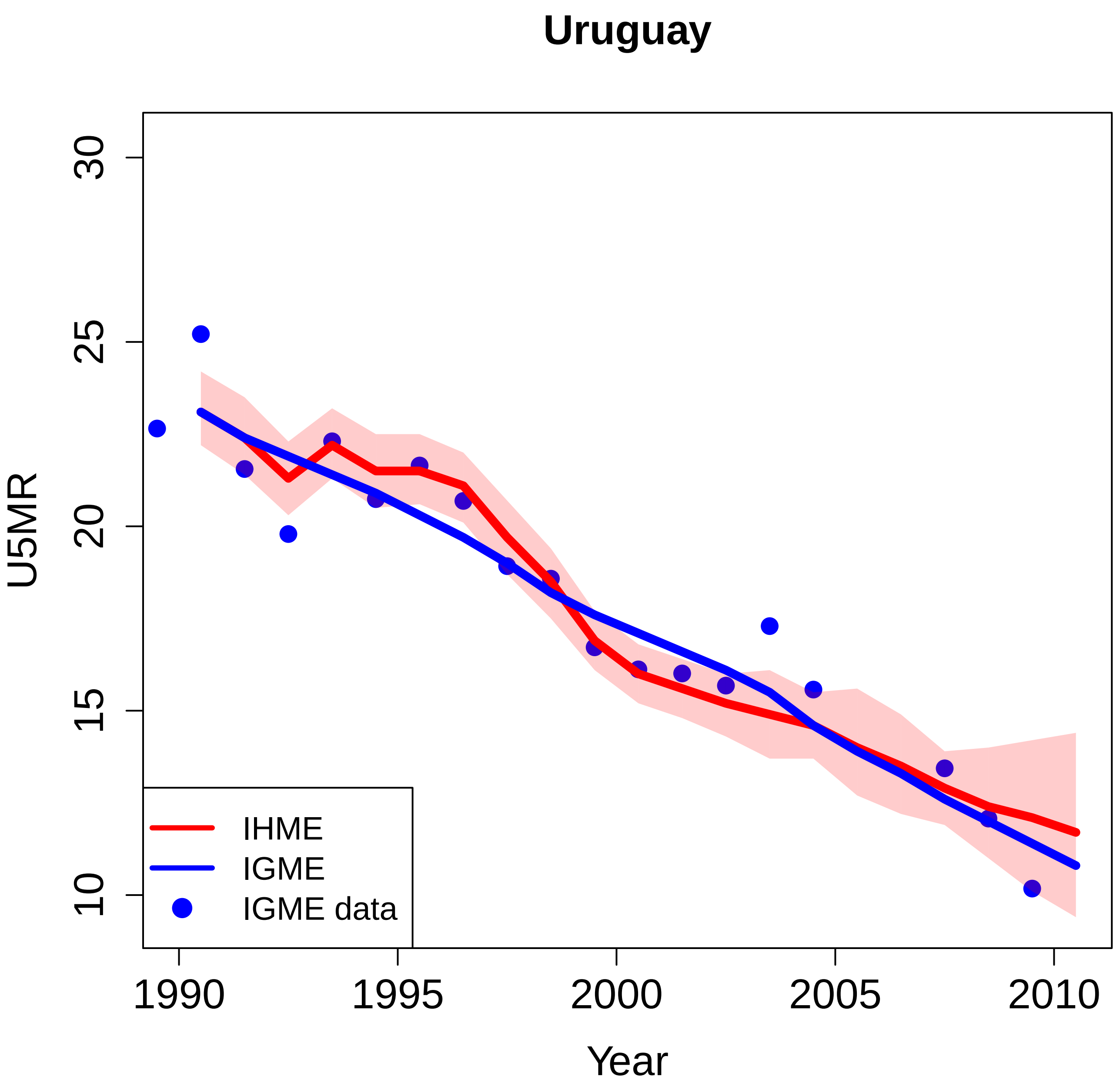

Uruguay
U5MR
30
25
20
15
10
1990
1995
2000
2005
2010
Year
IHME
IGME
IGME data

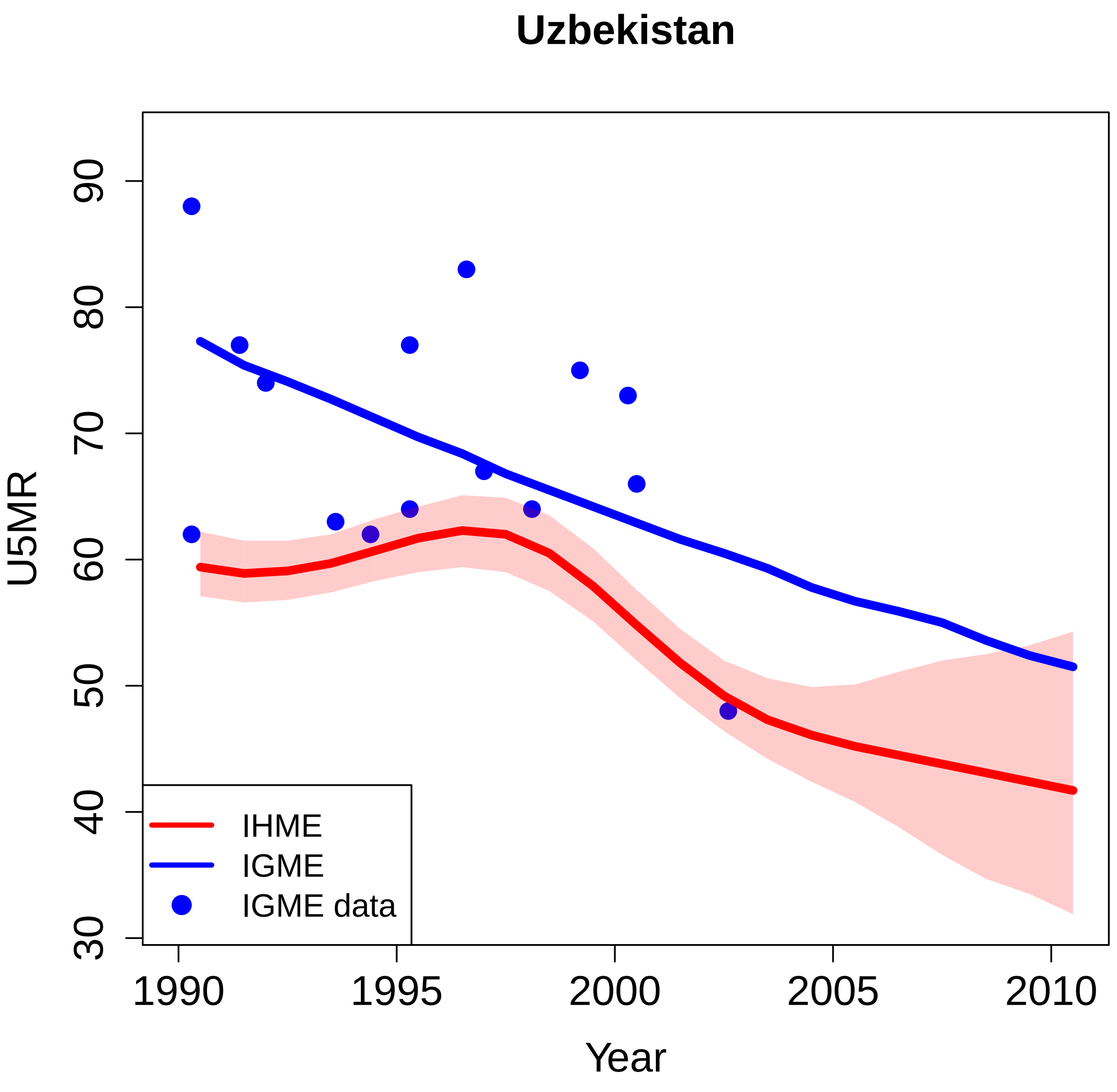
Uzbekistan
U5MR
Year
30
40
50
60
70
80
90
1990
1995
2000
2005
2010
IHME
IGME
IGME data

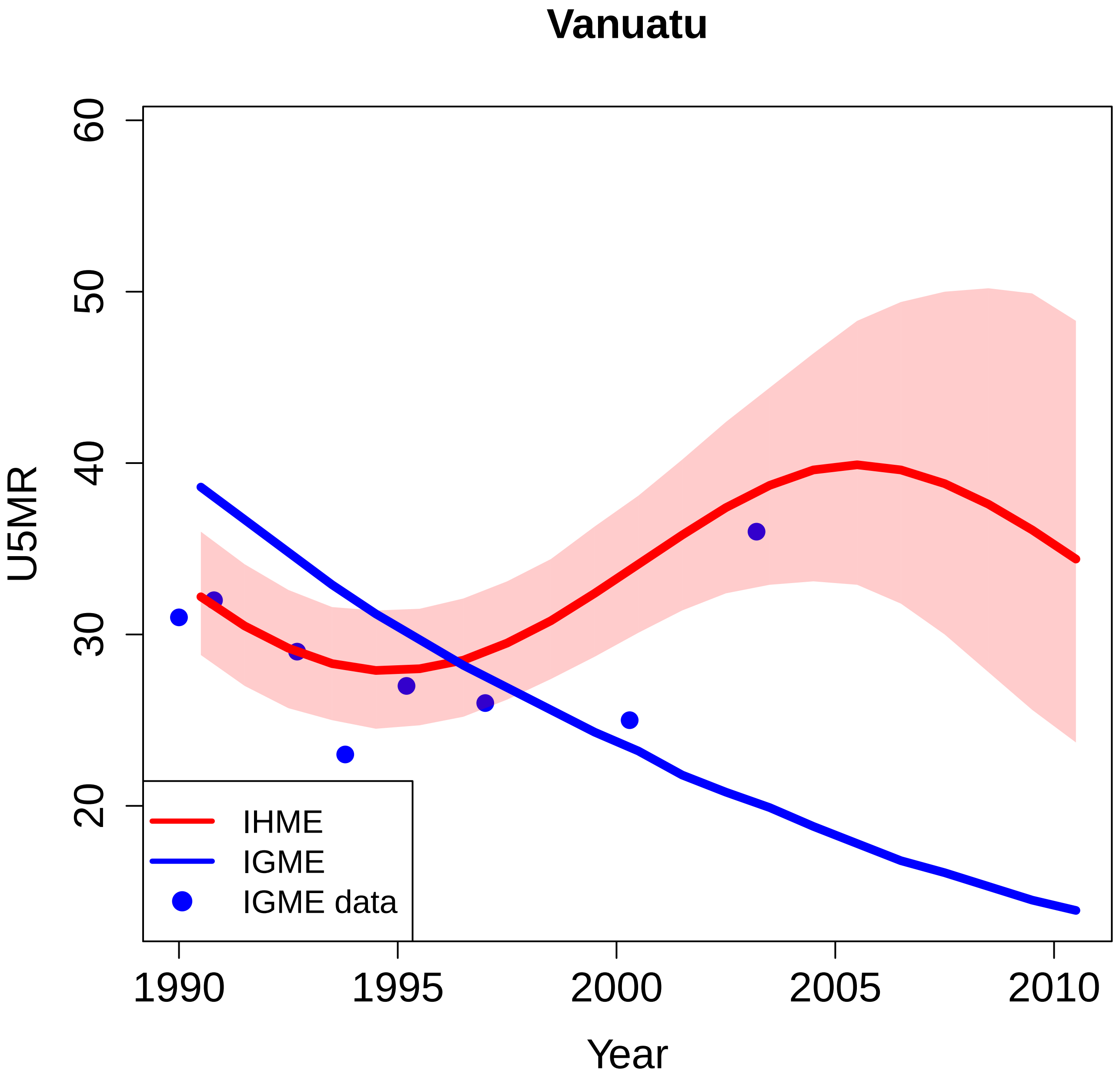

Vanuatu
U5MR
60
50
40
30
20
1990
1995
2000
2005
2010
Year
IHME
IGME
IGME data

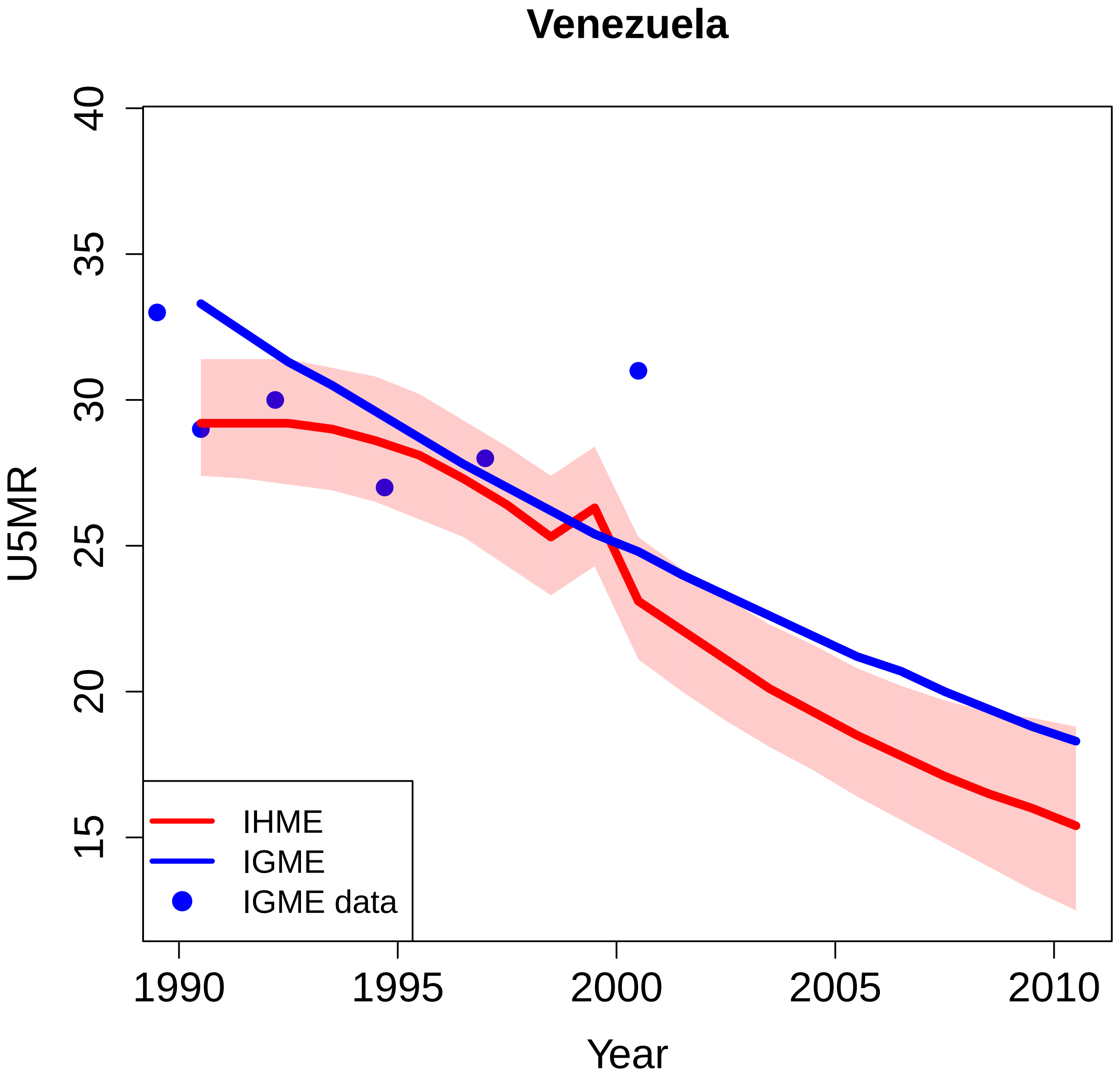
Venezuela
U5MR
40
35
30
25
20
15
1990
1995
2000
2005
2010
Year
IHME
IGME
IGME data

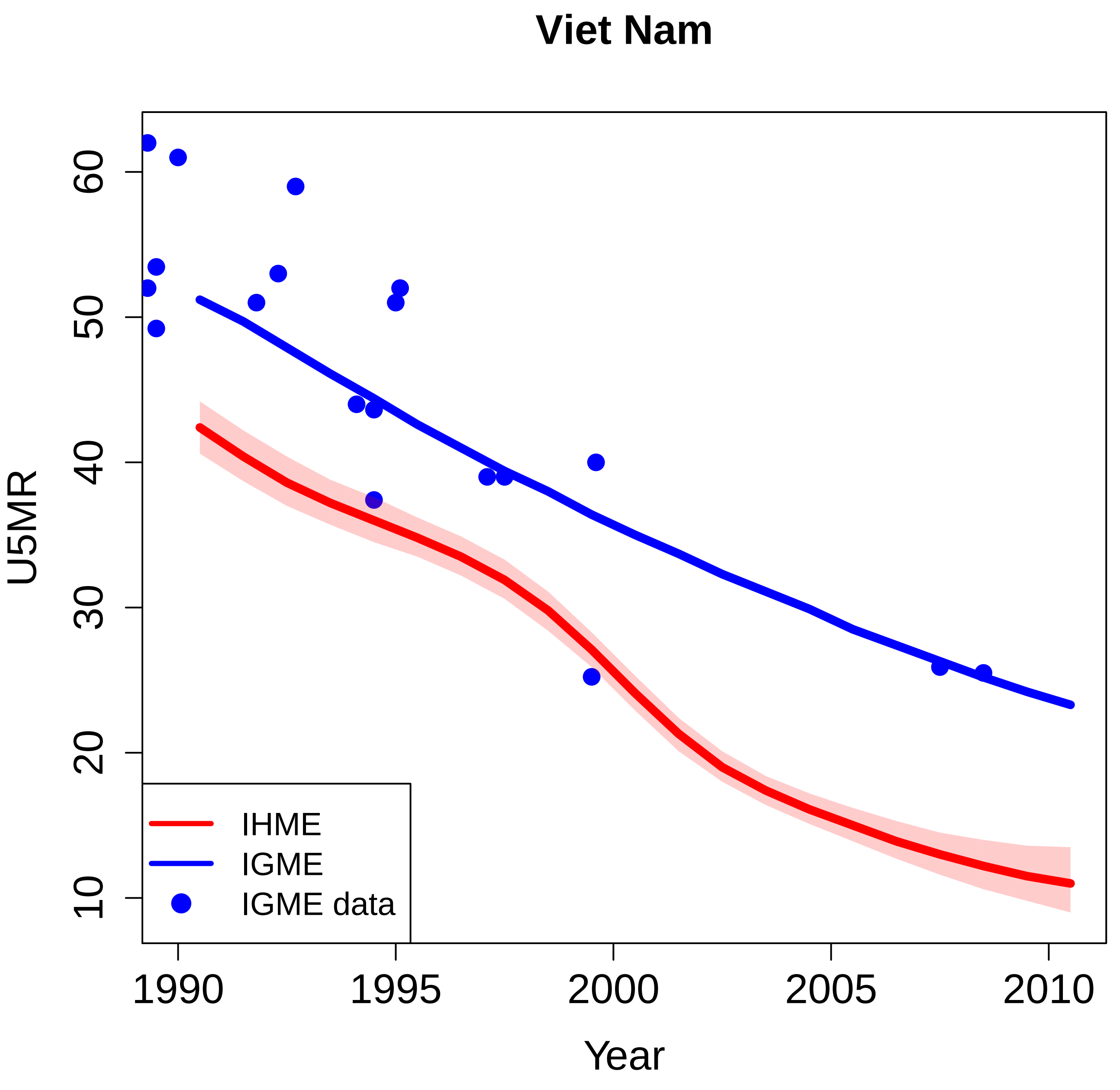

Viet Nam
U5MR
Year
10
20
30
40
50
60
1990
1995
2000
2005
2010
IHME
IGME
IGME data

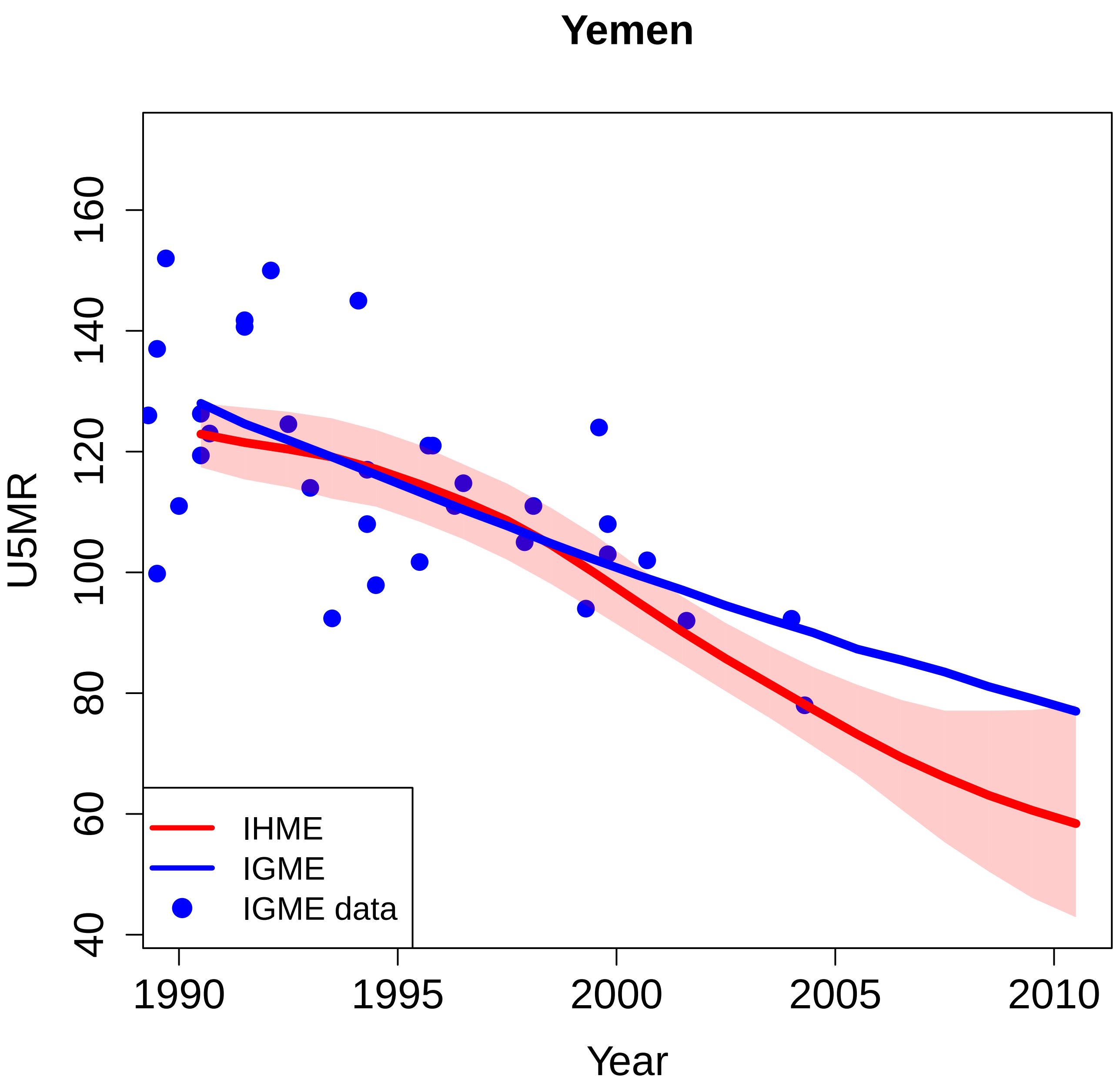

Yemen
U5MR
160
140
120
100
80
60
40
1990
1995
2000
2005
2010
Year
IHME
IGME
IGME data

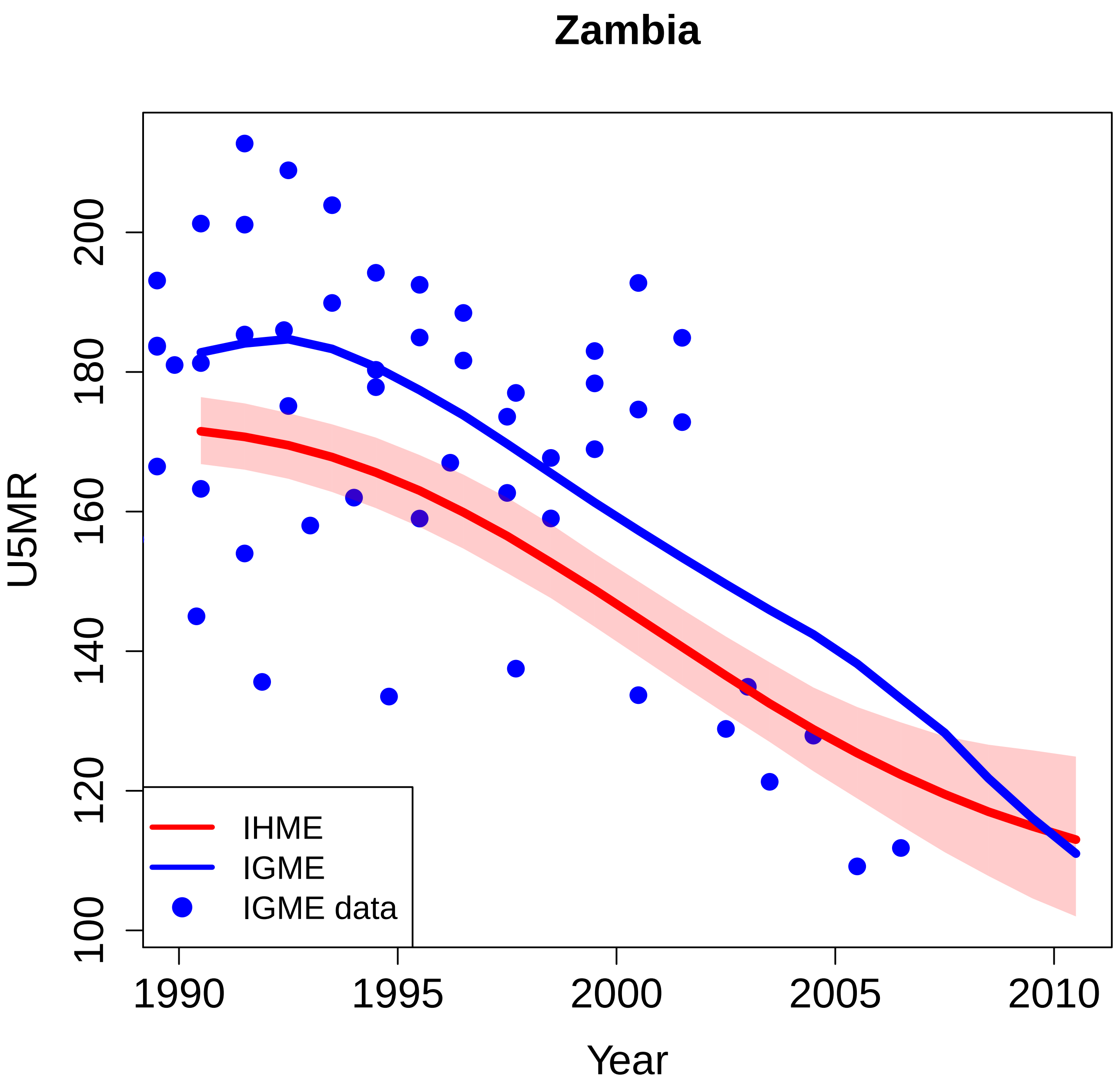

Zambia
U5MR
100
120
140
160
180
200
1990
1995
2000
2005
2010
Year
IHME
IGME
IGME data

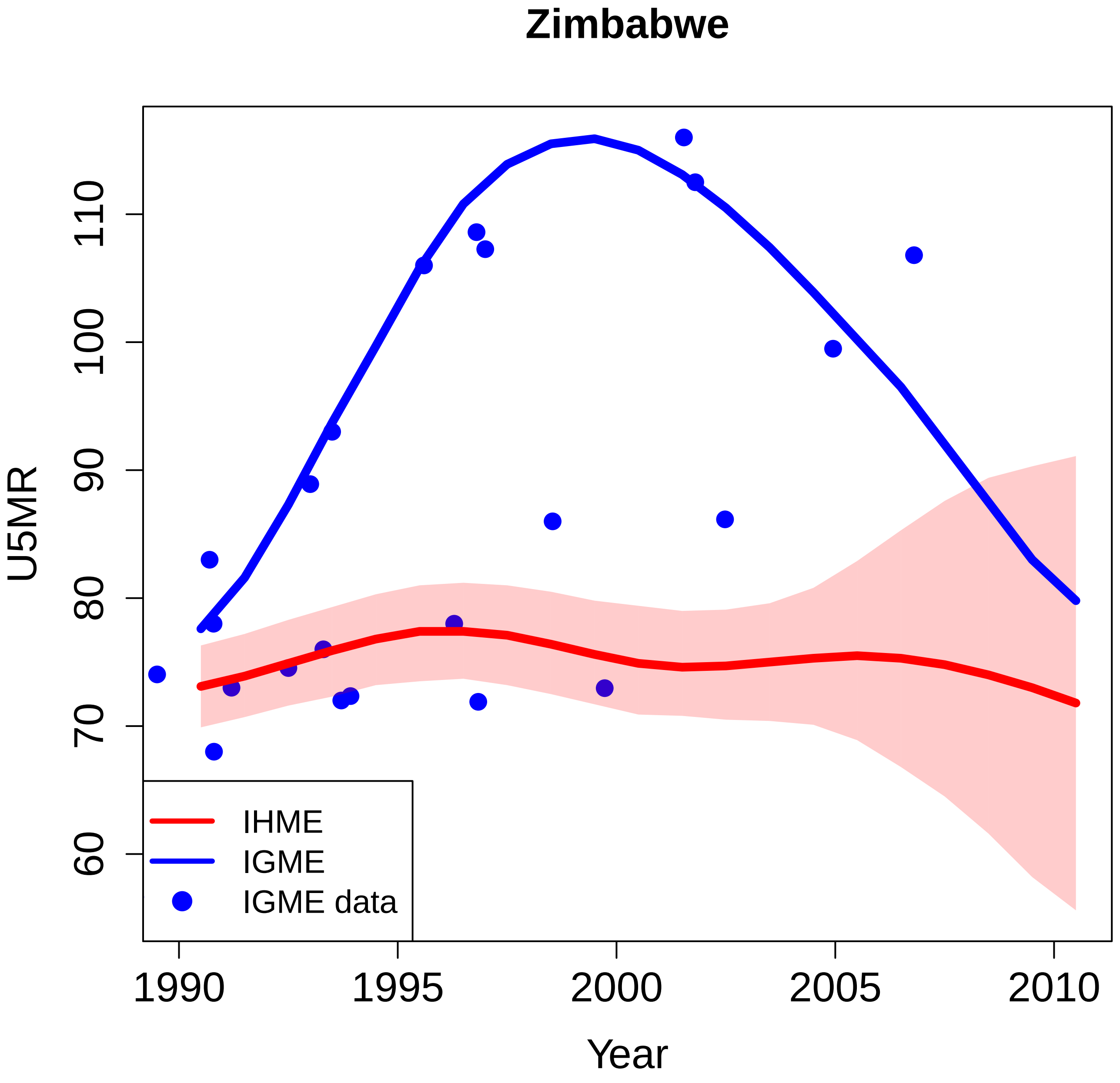

Zimbabwe
U5MR
110
100
90
80
70
60
1990
1995
2000
2005
2010
Year
IHME
IGME
IGME data
