# Supplementary material for: Child Mortality Estimation: A Comparison of UN IGME and IHME Estimates of Levels and Trends in Under-Five Mortality Rates and Deaths
Source: PLoS Med. 2012 Aug 28;9(8):e1001288. doi: 10.1371/journal.pmed.1001288 (PMC3429386; doi:10.1371/journal.pmed.1001288)
Supplement: Figure S2 — U5MR estimates for 36 countries based on loess versus GPR fitting methods. For each country is shown (i) loess fit to the 2011 UN IGME database (IGME 2010*; data and fit in blue; dataset excludes data collected in 2010), (ii) loess fit to the 2010 IHME database (data in red; fit in black), and (iii) GPR fit to the 2010 IHME database (IHME 2010; data and fit in red). (PDF) [file pmed.1001288.s002.pdf]

Angola

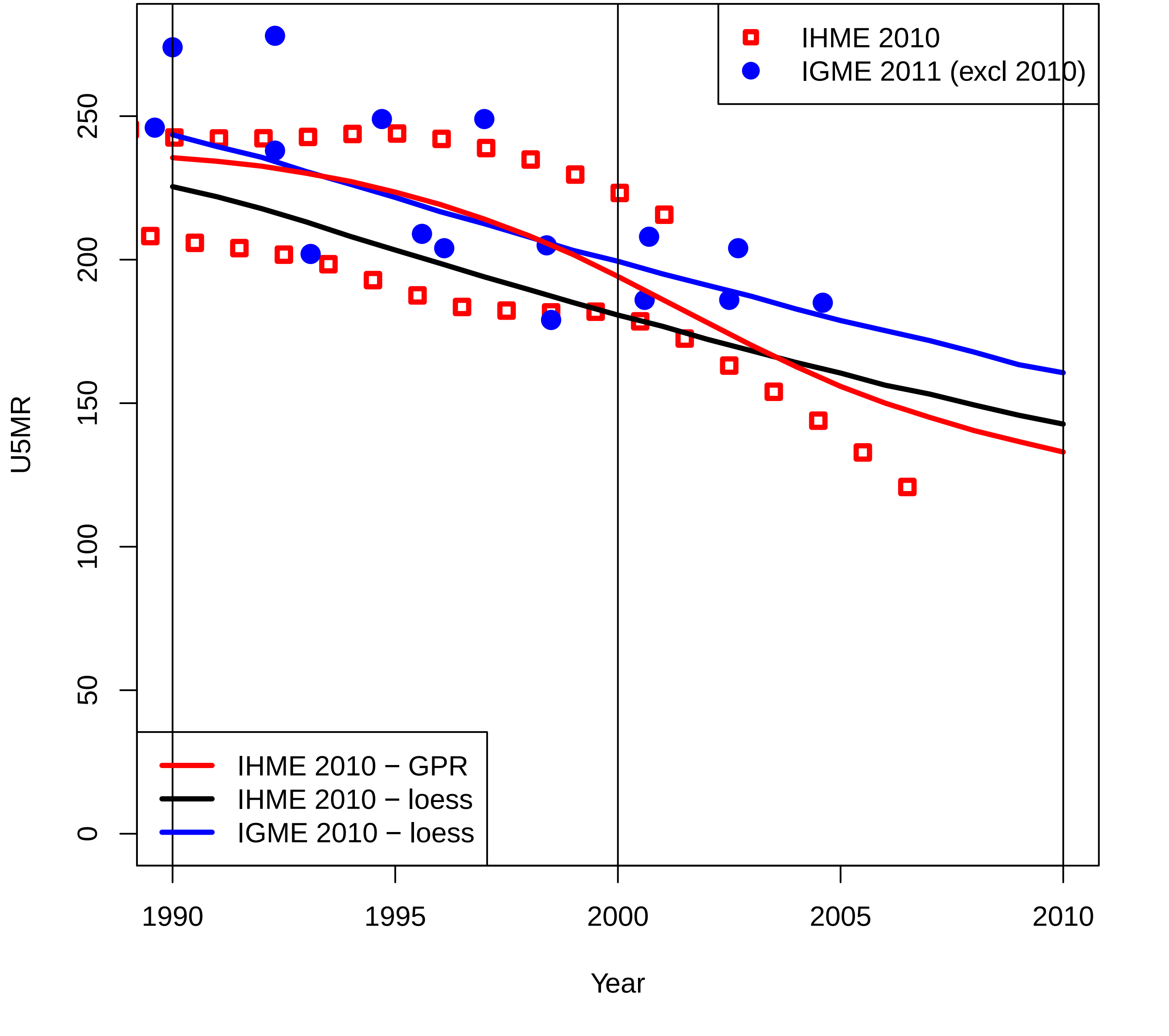

IHME 2010
IGME 2011 (excl 2010)
IHME 2010 − GPR
IHME 2010 − loess
IGME 2010 − loess
U5MR
0
50
100
150
200
250
1990
1995
2000
2005
2010
Year

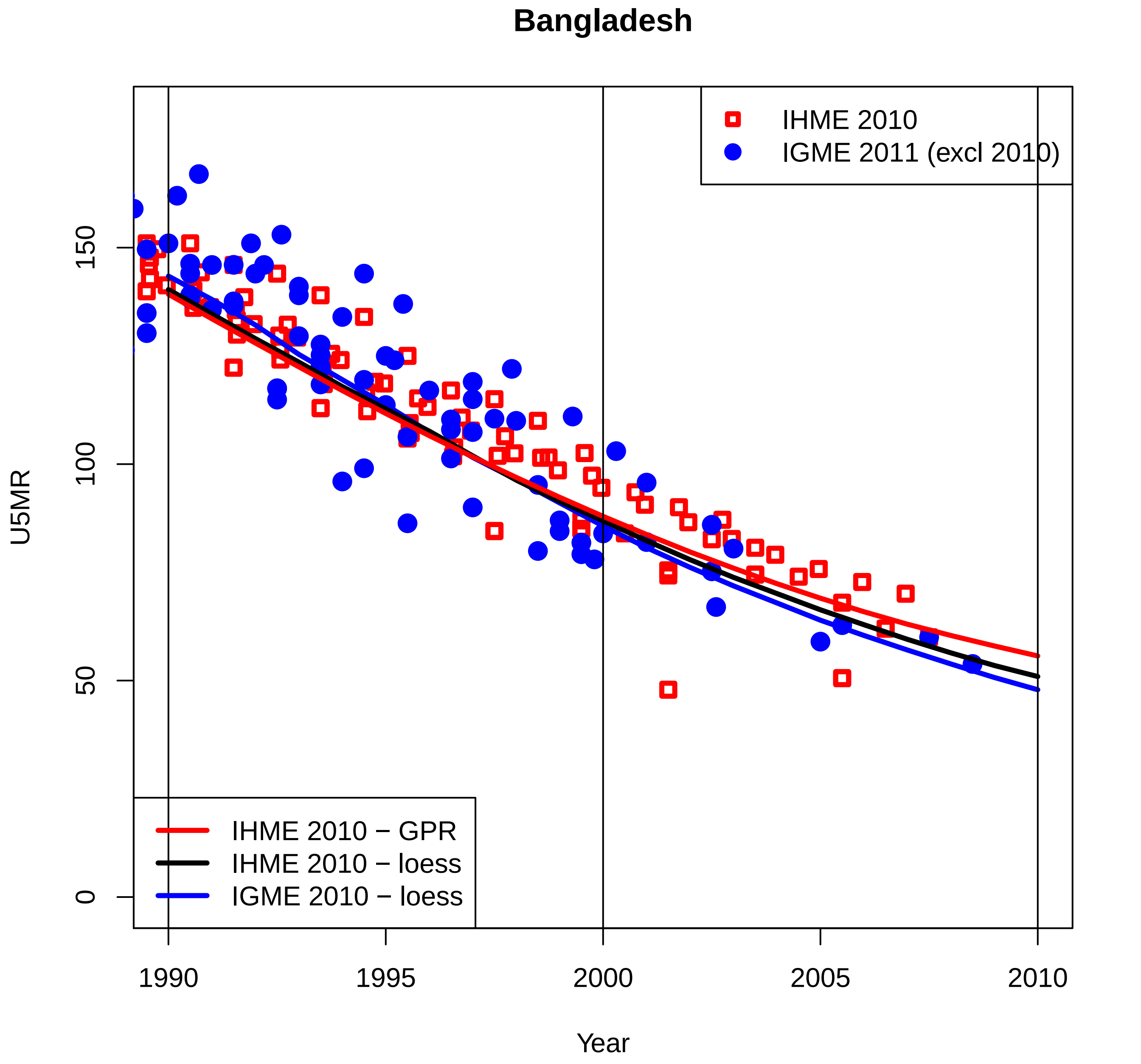
Bangladesh
IHME 2010
IGME 2011 (excl 2010)
IHME 2010 − GPR
IHME 2010 − loess
IGME 2010 − loess
U5MR
0
50
100
150
1990
1995
2000
2005
2010
Year

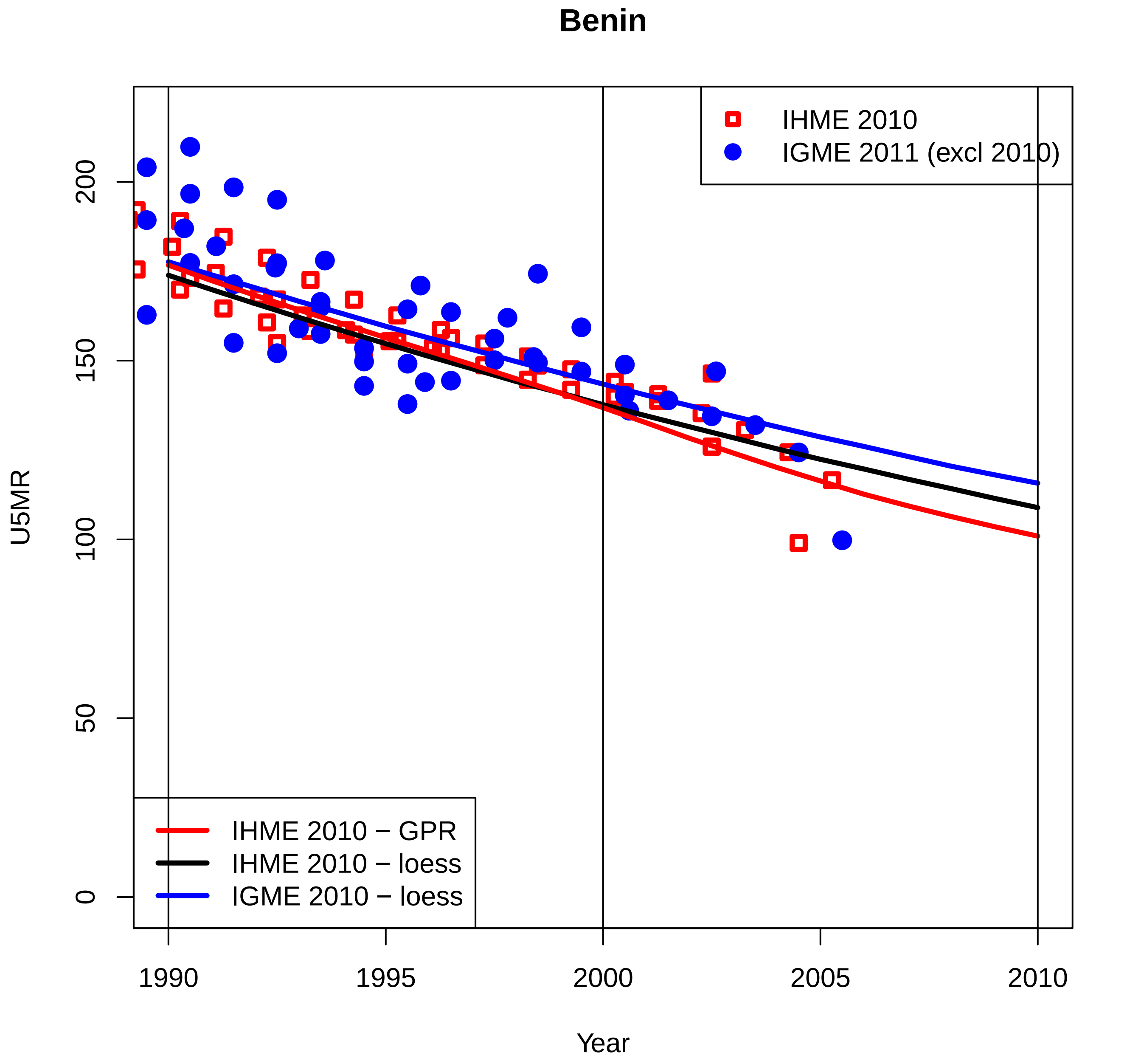
Benin
IHME 2010
IGME 2011 (excl 2010)
IHME 2010 − GPR
IHME 2010 − loess
IGME 2010 − loess
U5MR
0
50
100
150
200
1990
1995
2000
2005
2010
Year

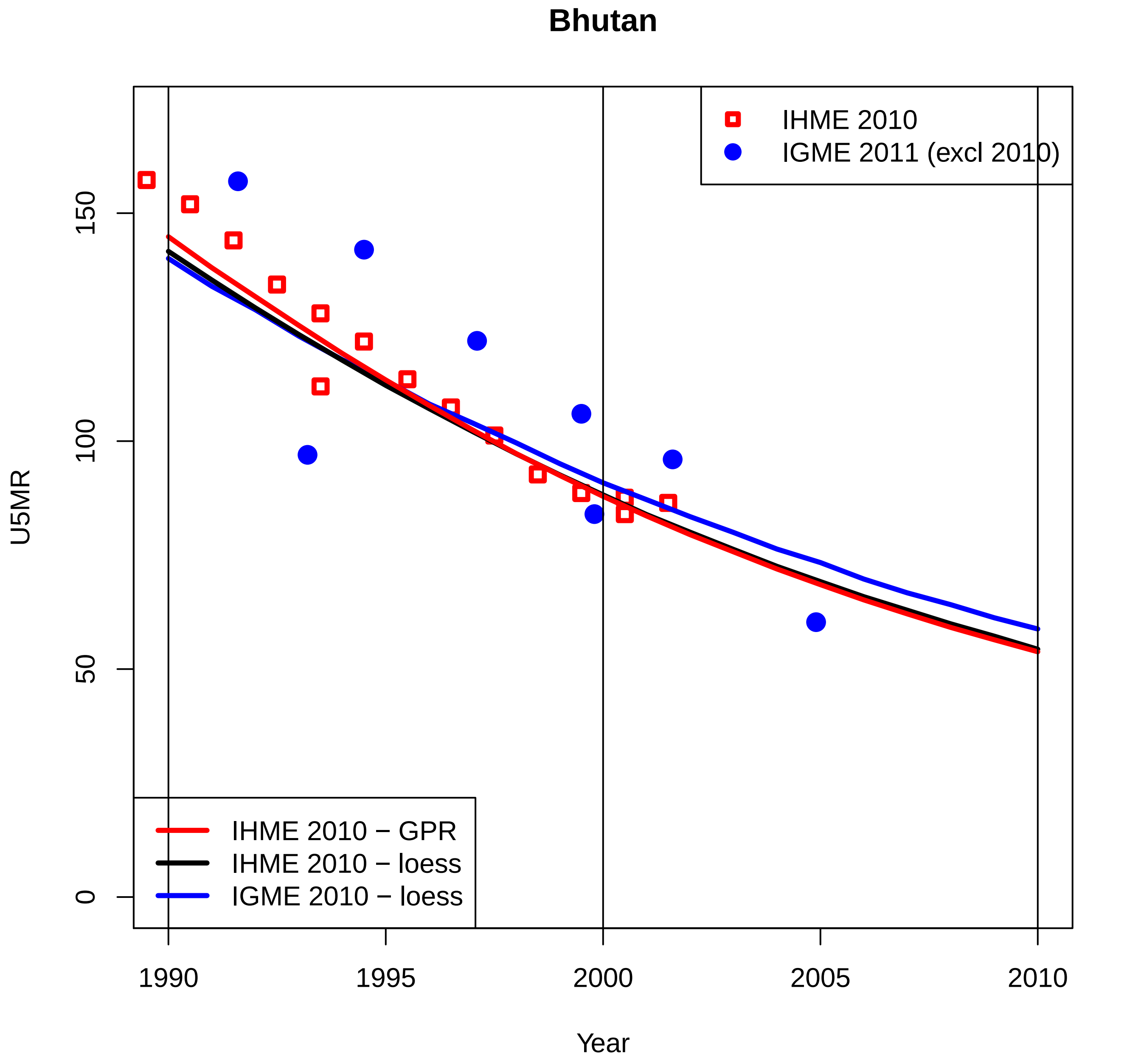
Bhutan
IHME 2010
IGME 2011 (excl 2010)
IHME 2010 − GPR
IHME 2010 − loess
IGME 2010 − loess
U5MR
150
100
50
0
1990
1995
2000
2005
2010
Year

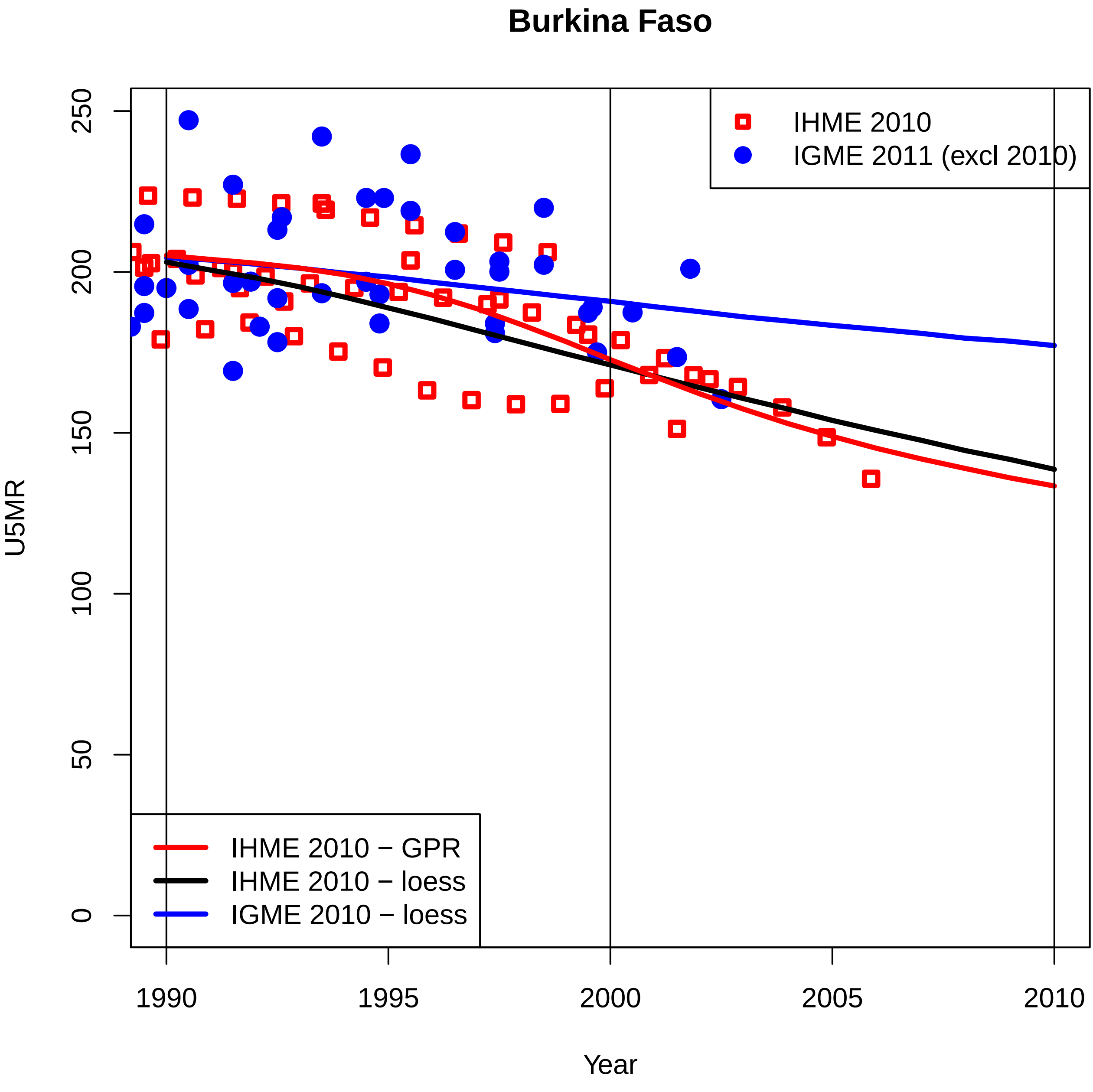

Burkina Faso
IHME 2010
IGME 2011 (excl 2010)
IHME 2010 − GPR
IHME 2010 − loess
IGME 2010 − loess
U5MR
0
50
100
150
200
250
1990
1995
2000
2005
2010
Year

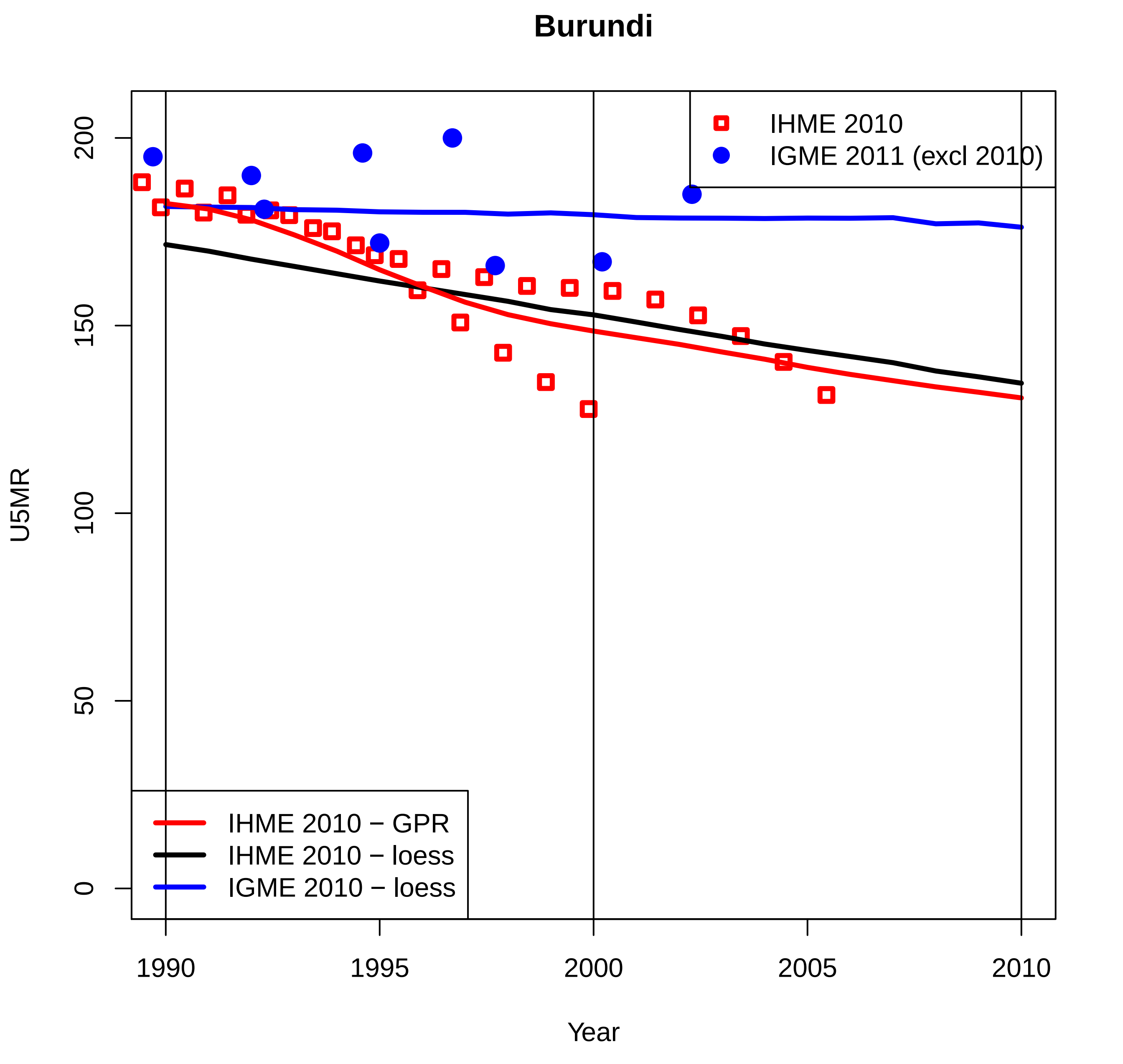

Burundi
IHME 2010
IGME 2011 (excl 2010)
U5MR
200
150
100
50
0
1990
1995
2000
2005
2010
Year
IHME 2010 − GPR
IHME 2010 − loess
IGME 2010 − loess

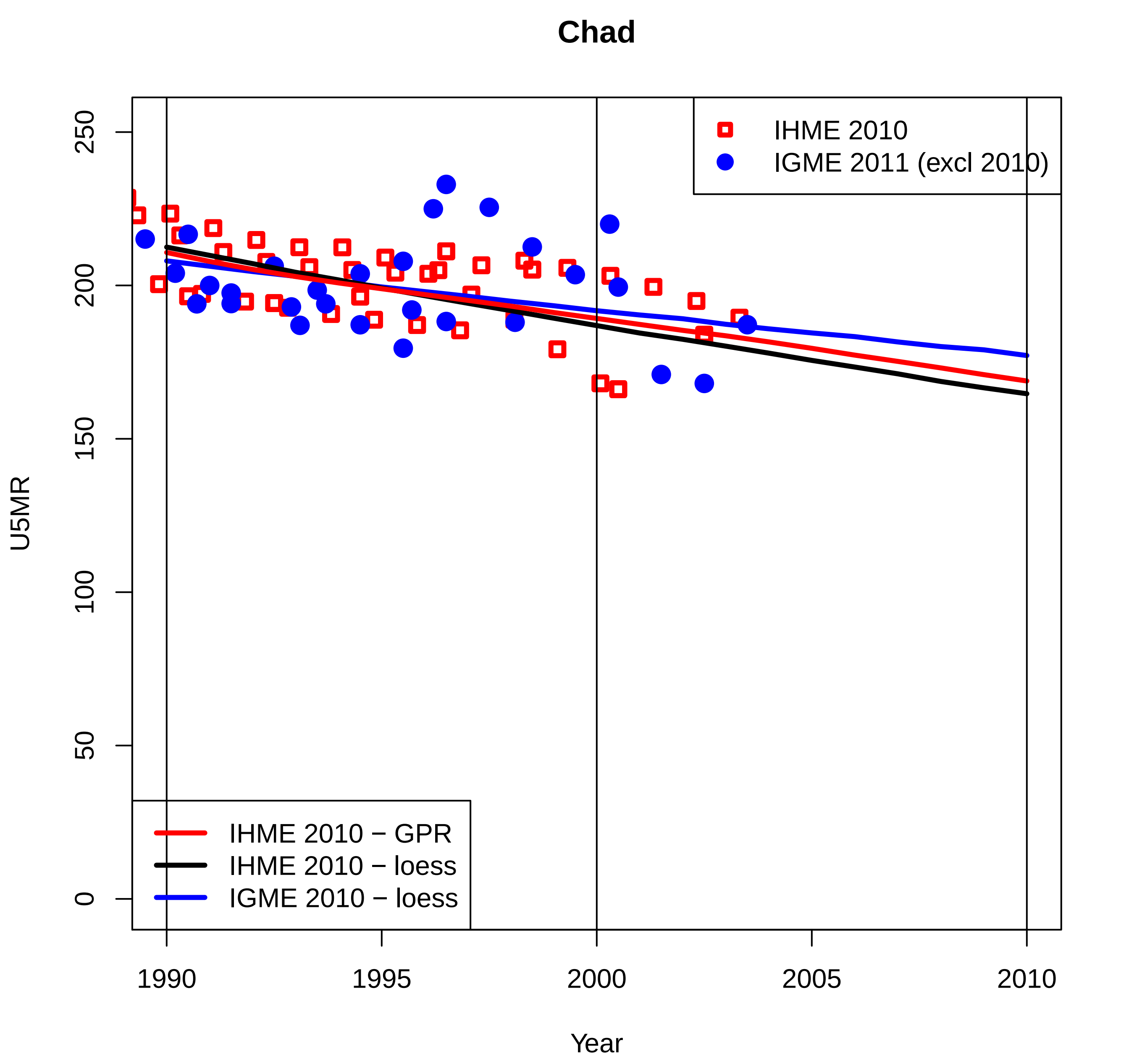

Chad
IHME 2010
IGME 2011 (excl 2010)
IHME 2010 − GPR
IHME 2010 − loess
IGME 2010 − loess
U5MR
0
50
100
150
200
250
1990
1995
2000
2005
2010
Year

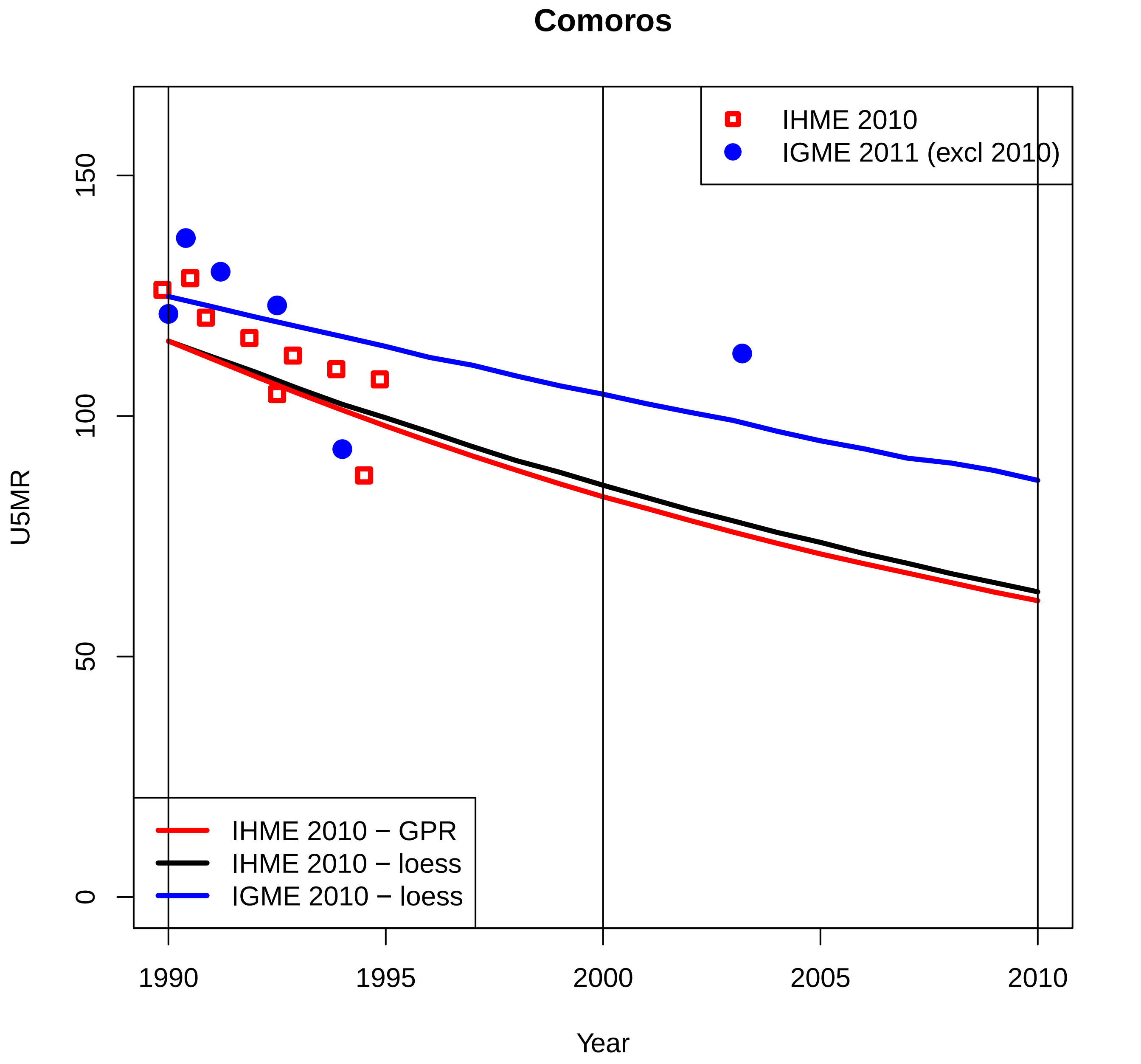
Comoros
IHME 2010
IGME 2011 (excl 2010)
IHME 2010 − GPR
IHME 2010 − loess
IGME 2010 − loess
U5MR
0
50
100
150
1990
1995
2000
2005
2010
Year

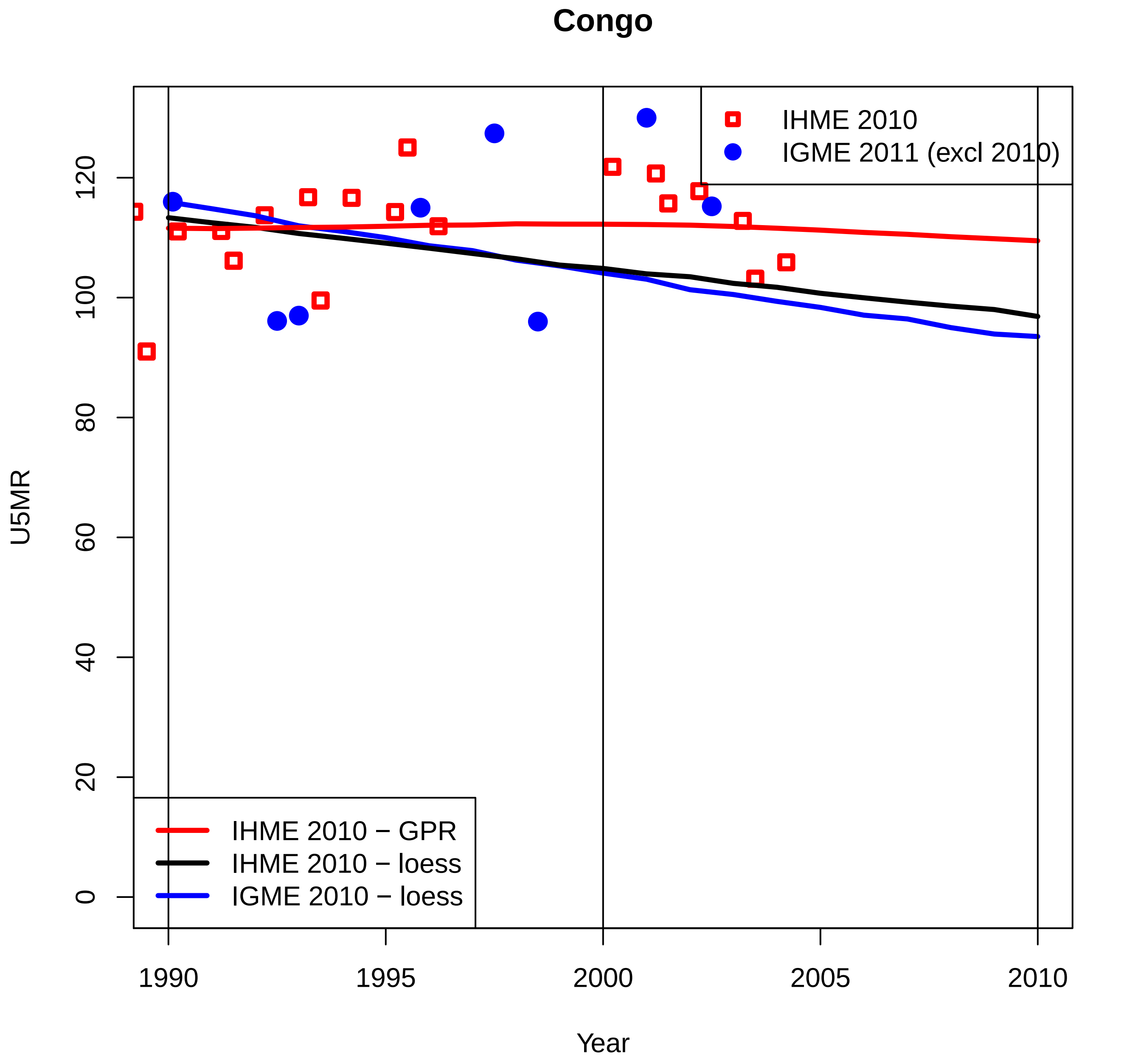

Congo
IHME 2010
IGME 2011 (excl 2010)
IHME 2010 − GPR
IHME 2010 − loess
IGME 2010 − loess
U5MR
0
20
40
60
80
100
120
1990
1995
2000
2005
2010
Year

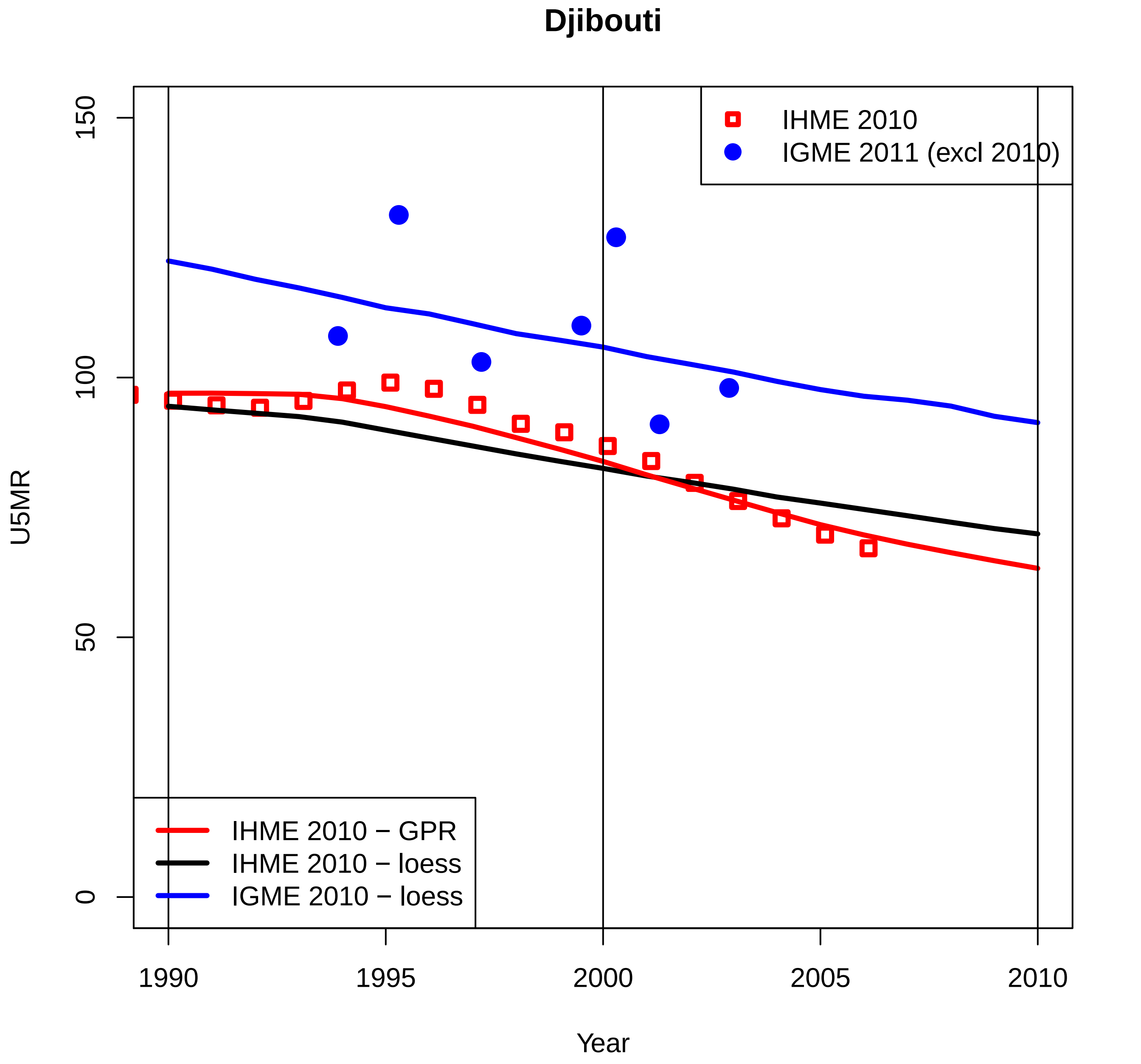

Djibouti
IHME 2010
IGME 2011 (excl 2010)
IHME 2010 − GPR
IHME 2010 − loess
IGME 2010 − loess
U5MR
0
50
100
150
1990
1995
2000
2005
2010
Year

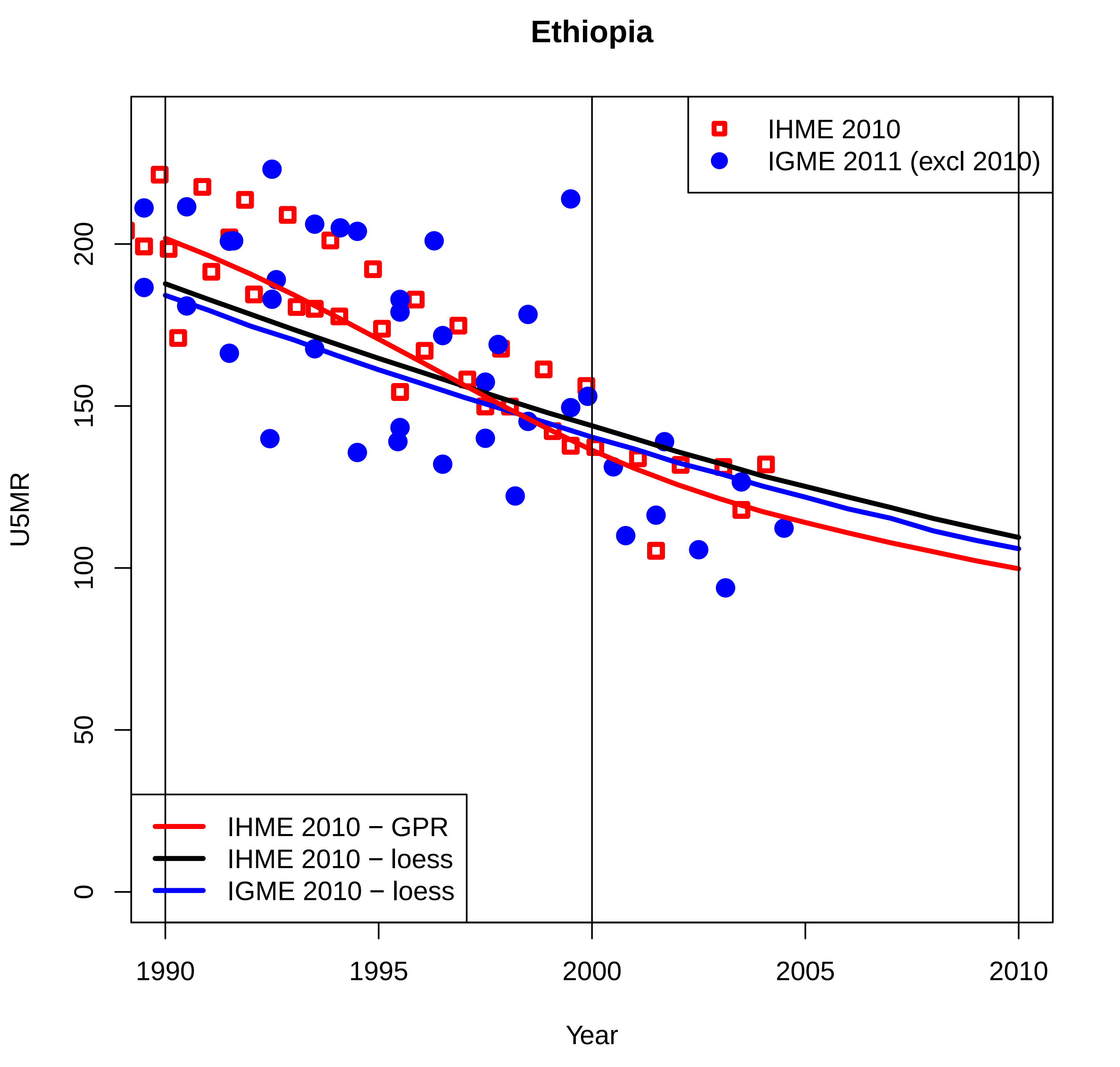

Ethiopia
IHME 2010
IGME 2011 (excl 2010)
IHME 2010 − GPR
IHME 2010 − loess
IGME 2010 − loess
U5MR
0
50
100
150
200
1990
1995
2000
2005
2010
Year

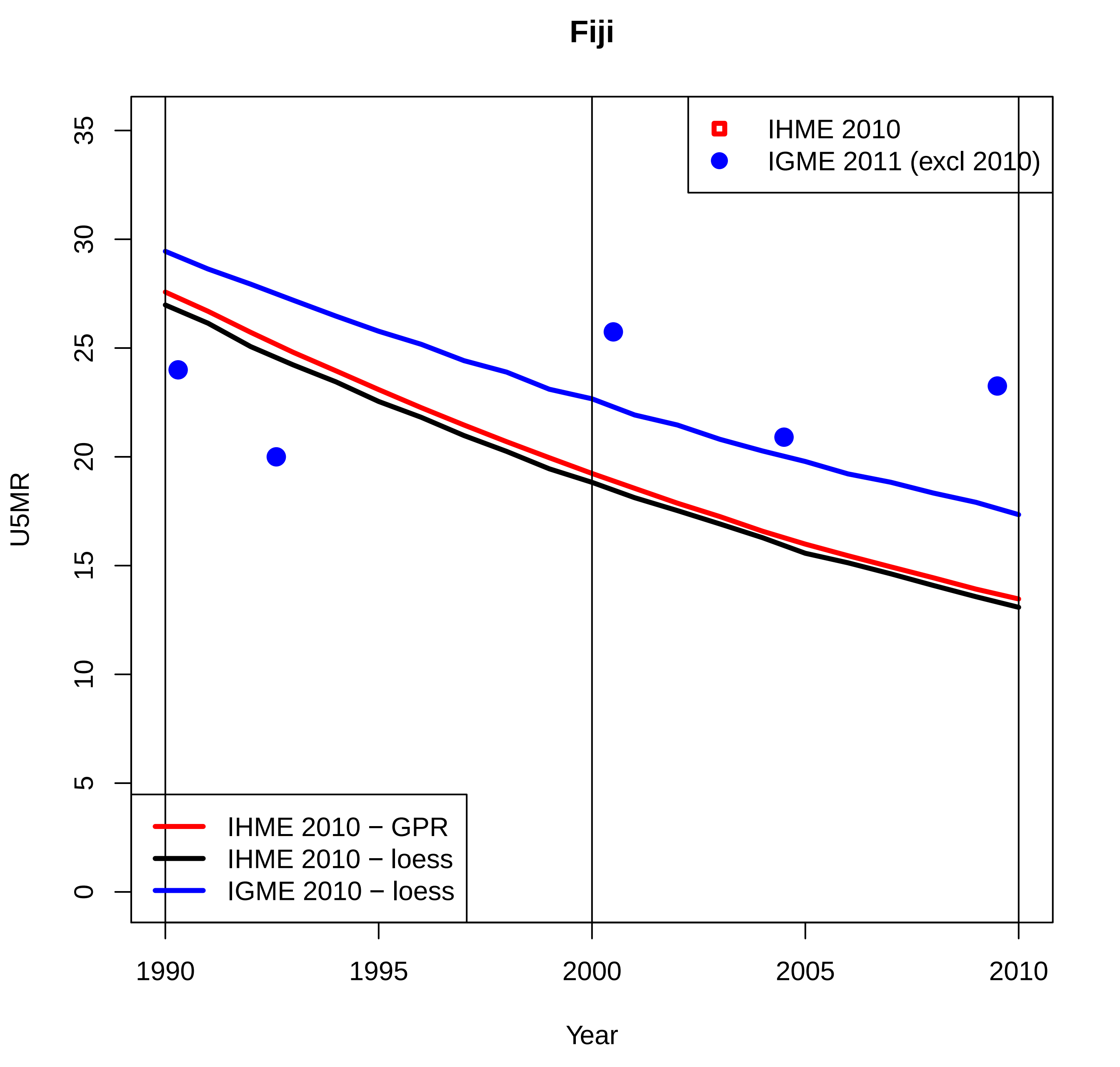
Fiji
IHME 2010
IGME 2011 (excl 2010)
IHME 2010 − GPR
IHME 2010 − loess
IGME 2010 − loess
U5MR
Year
0
5
10
15
20
25
30
35
1990
1995
2000
2005
2010

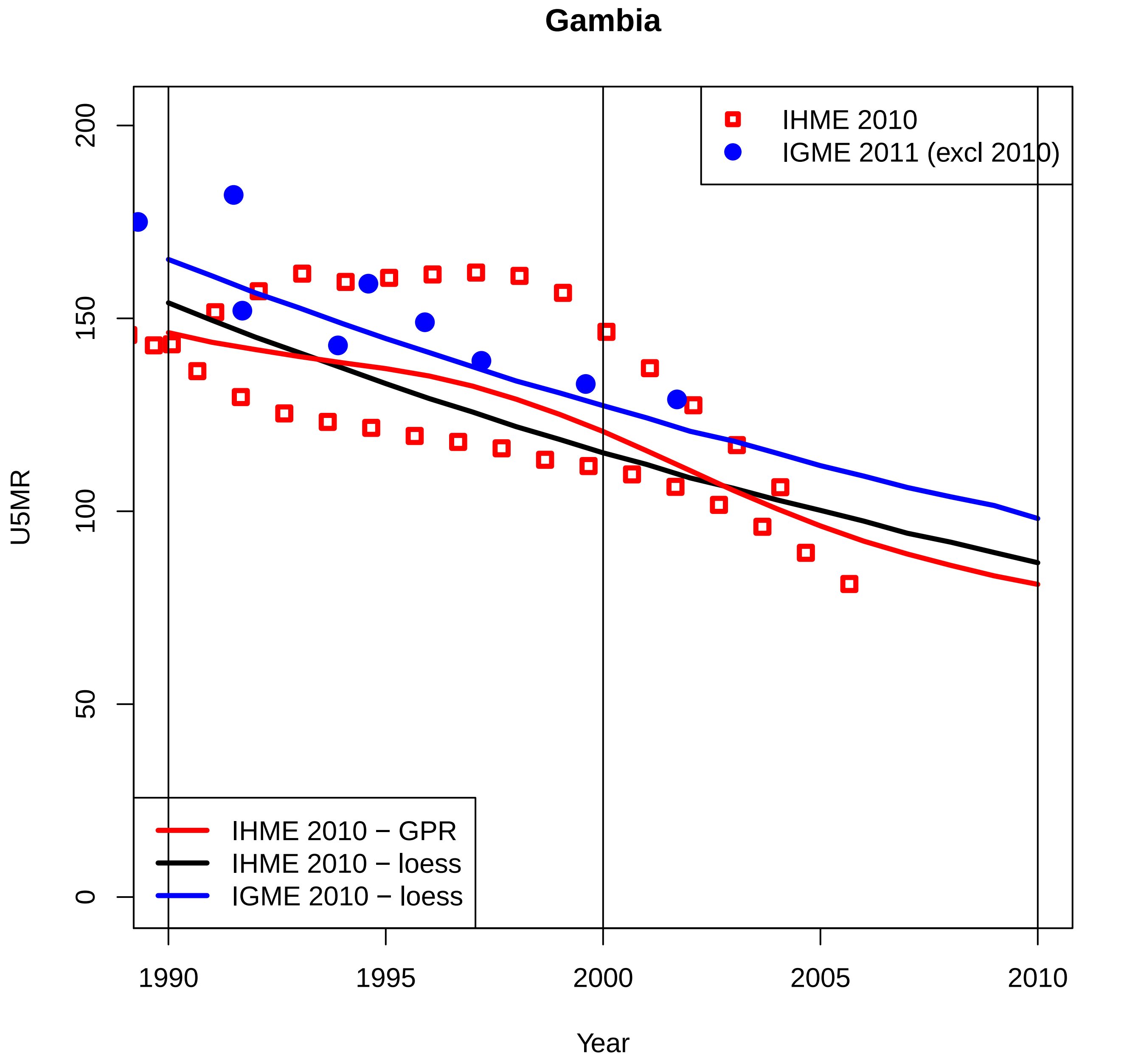

Gambia
U5MR
Year
0
50
100
150
200
1990
1995
2000
2005
2010
IHME 2010
IGME 2011 (excl 2010)
IHME 2010 − GPR
IHME 2010 − loess
IGME 2010 − loess

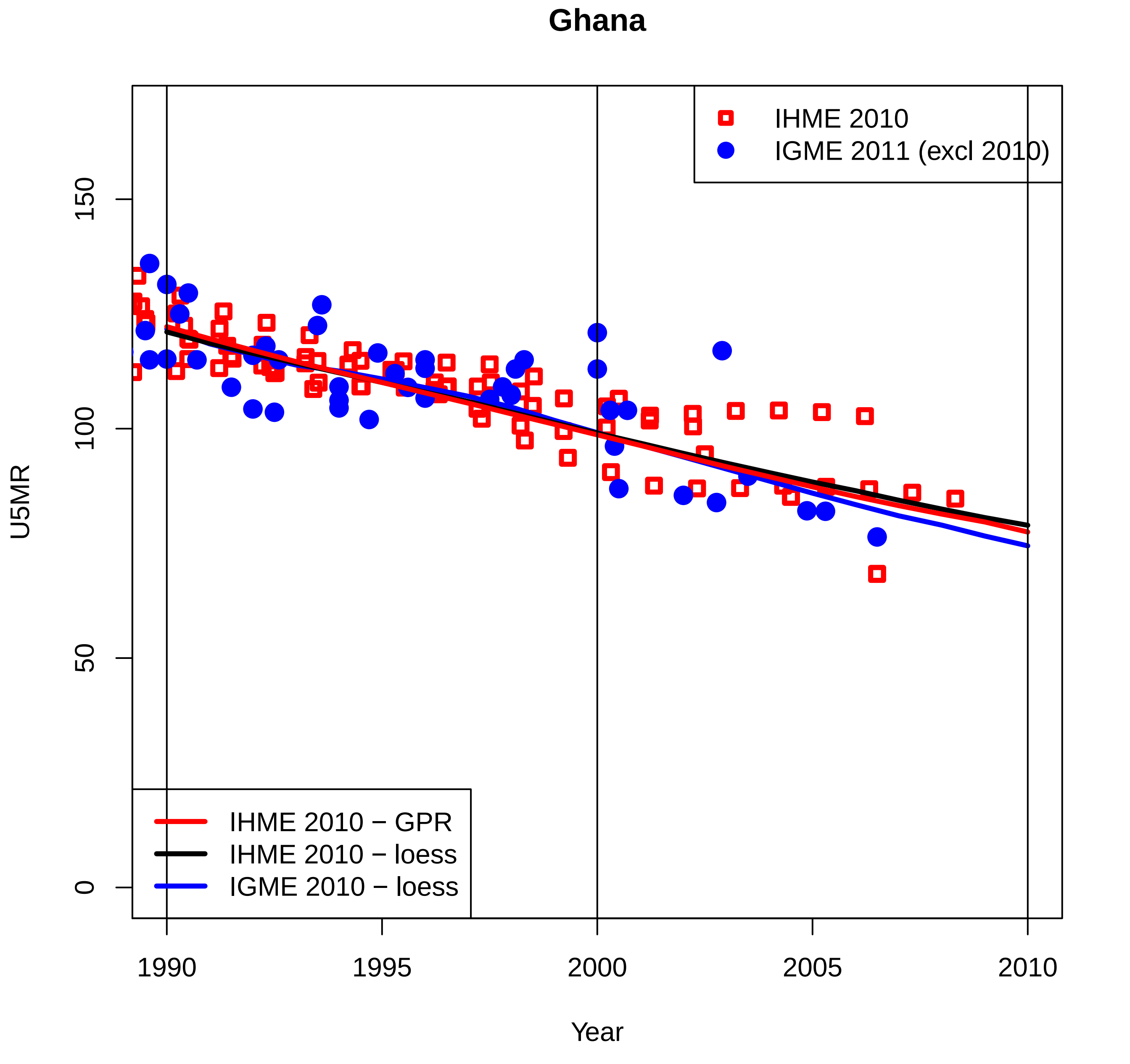

Ghana
IHME 2010
IGME 2011 (excl 2010)
IHME 2010 − GPR
IHME 2010 − loess
IGME 2010 − loess
U5MR
0
50
100
150
Year
1990
1995
2000
2005
2010

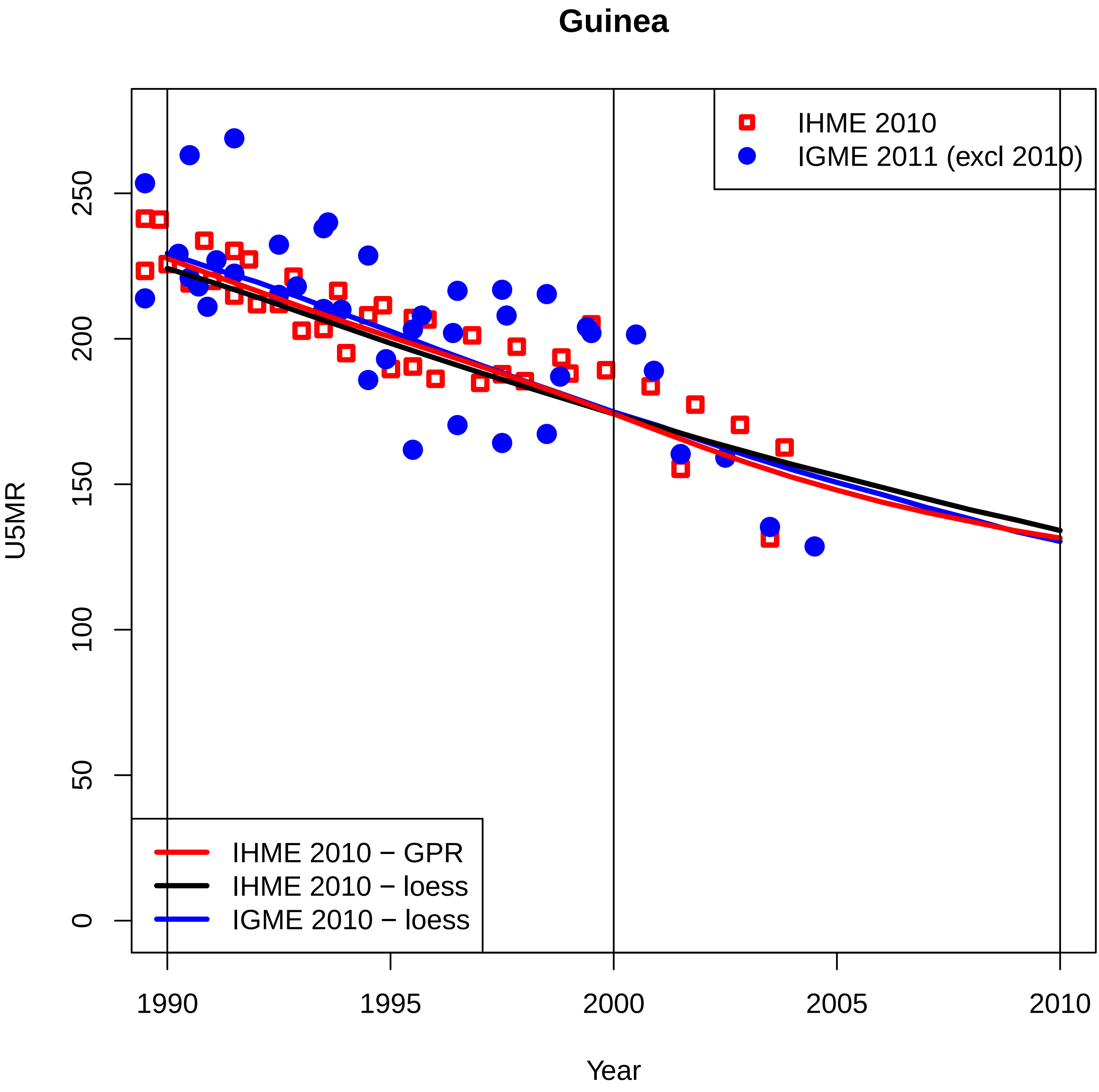
Guinea
IHME 2010
IGME 2011 (excl 2010)
IHME 2010 − GPR
IHME 2010 − loess
IGME 2010 − loess
U5MR
0
50
100
150
200
250
1990
1995
2000
2005
2010
Year

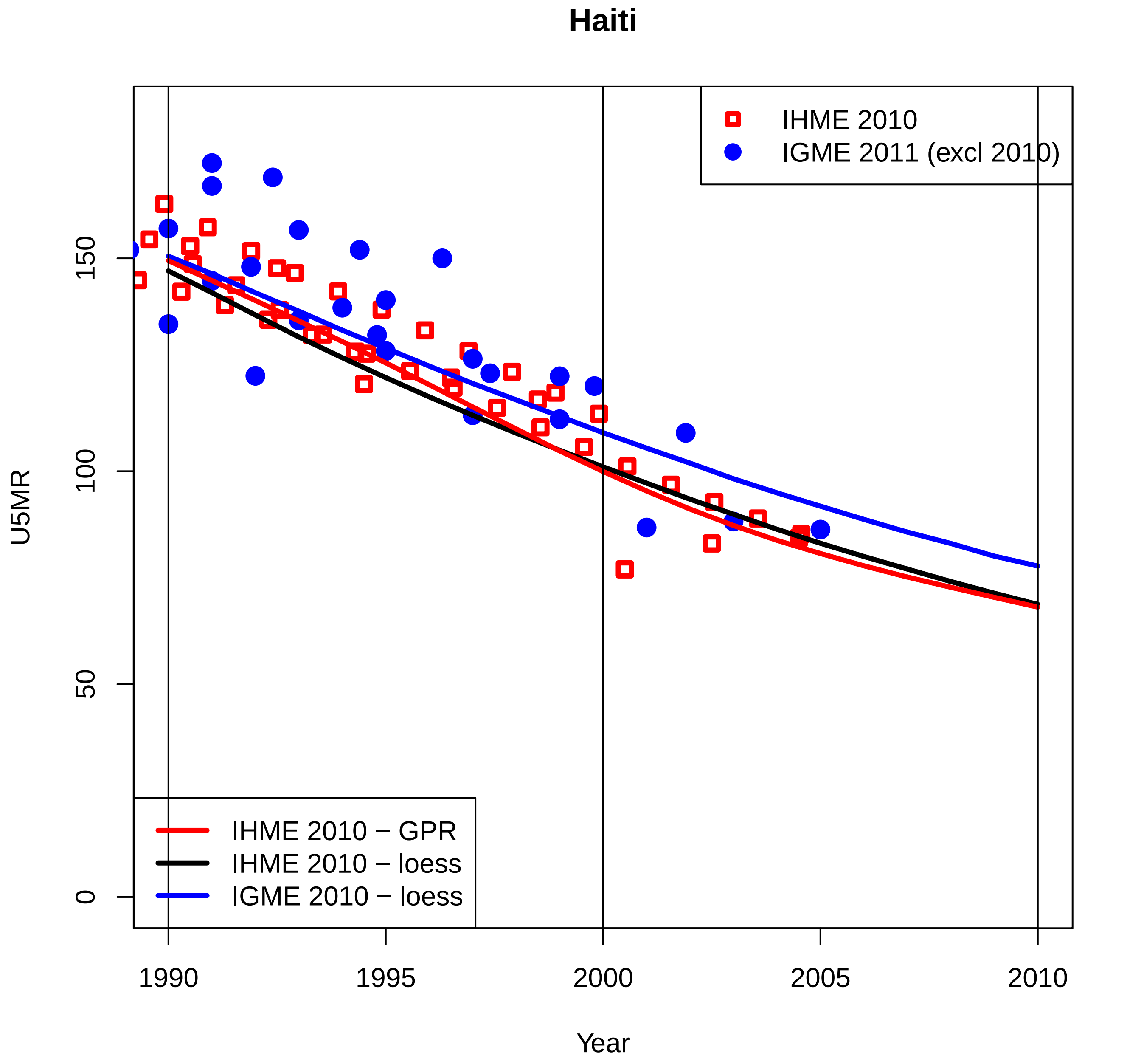

Haiti
IHME 2010
IGME 2011 (excl 2010)
IHME 2010 − GPR
IHME 2010 − loess
IGME 2010 − loess
U5MR
0
50
100
150
1990
1995
2000
2005
2010
Year

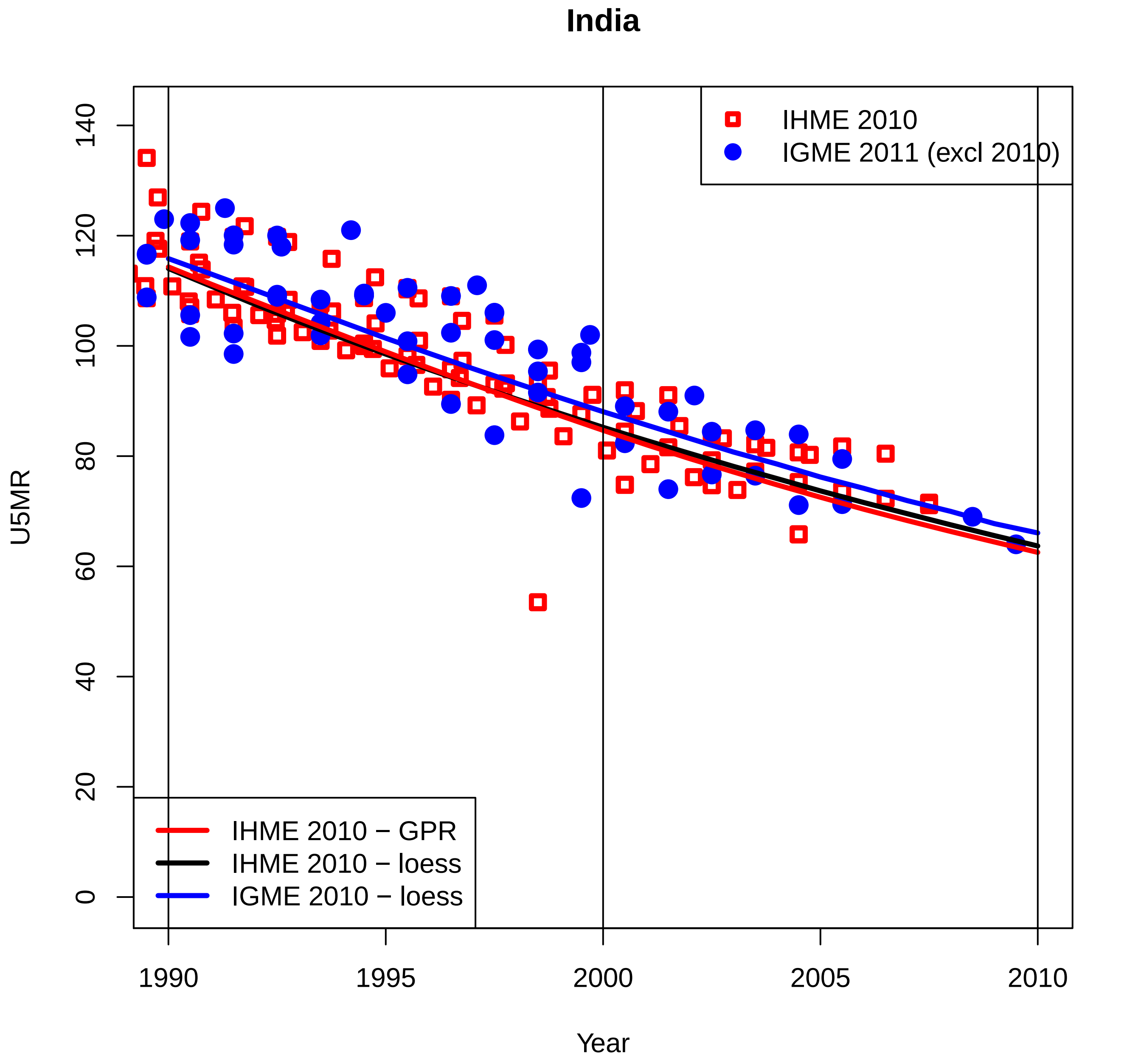
India
U5MR
Year
0
20
40
60
80
100
120
140
1990
1995
2000
2005
2010
IHME 2010
IGME 2011 (excl 2010)
IHME 2010 − GPR
IHME 2010 − loess
IGME 2010 − loess

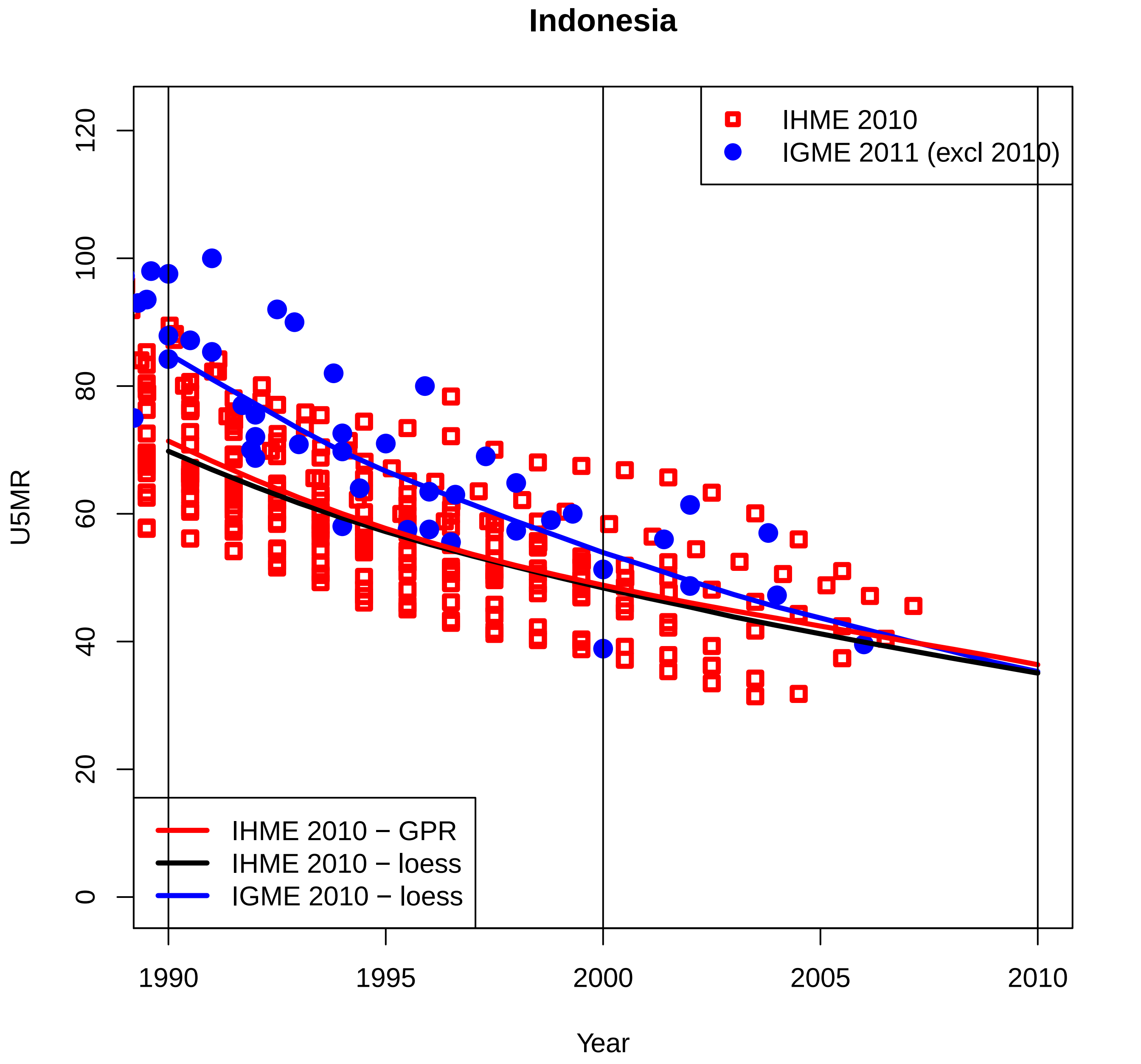

Indonesia
IHME 2010
IGME 2011 (excl 2010)
IHME 2010 − GPR
IHME 2010 − loess
IGME 2010 − loess
U5MR
0
20
40
60
80
100
120
1990
1995
2000
2005
2010
Year

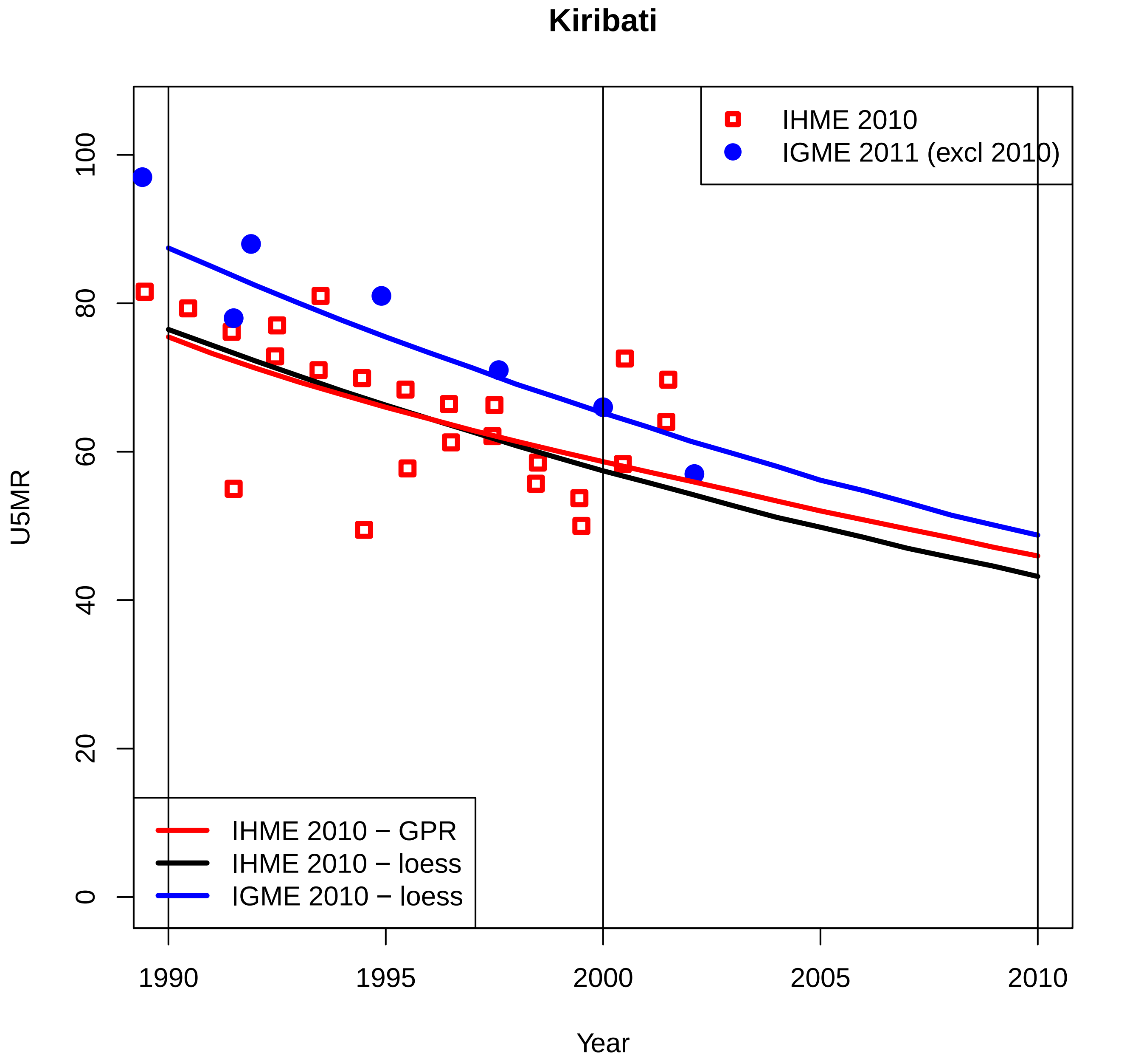

Kiribati
IHME 2010
IGME 2011 (excl 2010)
IHME 2010 − GPR
IHME 2010 − loess
IGME 2010 − loess
U5MR
0
20
40
60
80
100
1990
1995
2000
2005
2010
Year

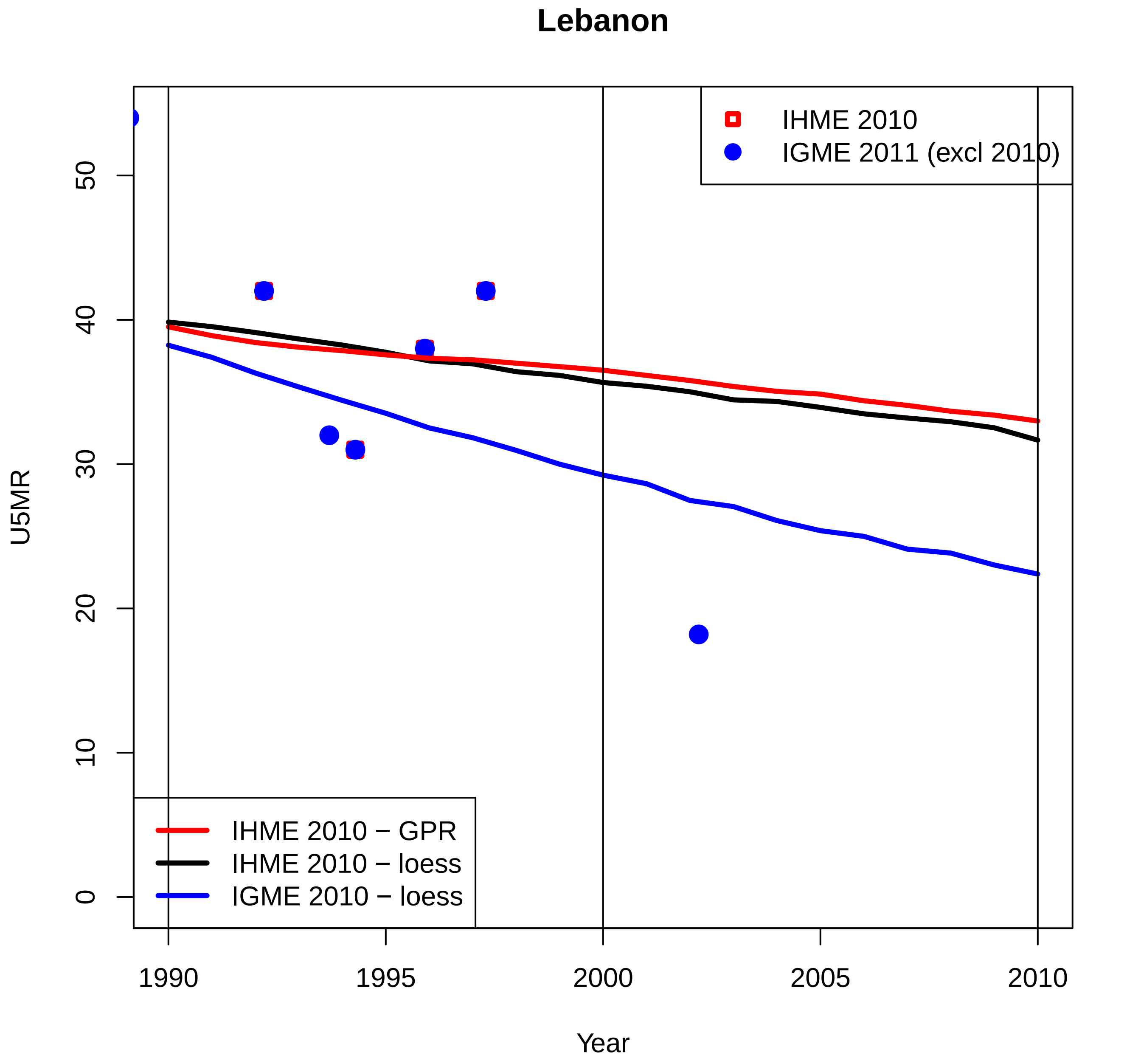
Lebanon
IHME 2010
IGME 2011 (excl 2010)
IHME 2010 − GPR
IHME 2010 − loess
IGME 2010 − loess
U5MR
Year
0
10
20
30
40
50
1990
1995
2000
2005
2010

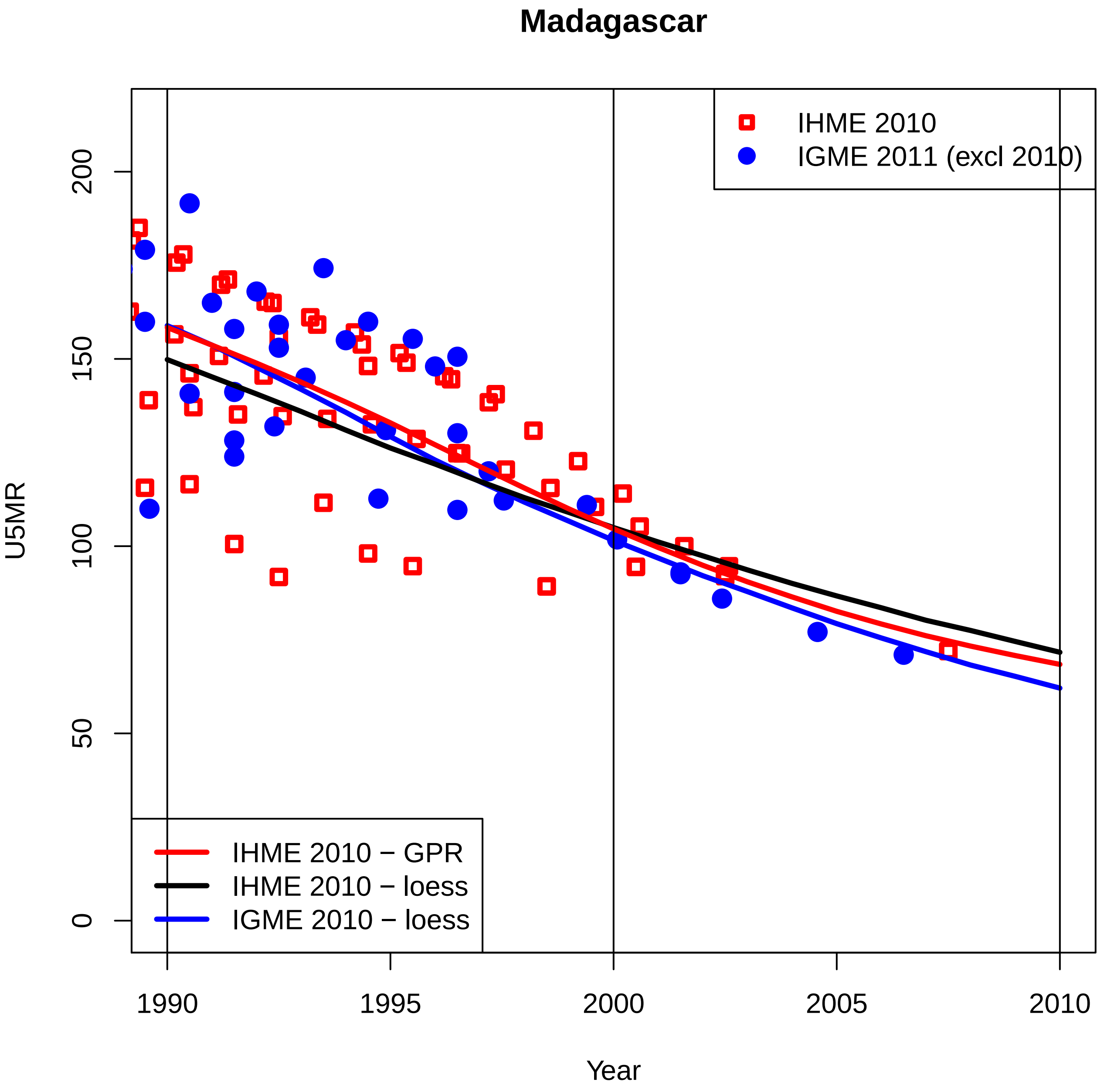
Madagascar
IHME 2010
IGME 2011 (excl 2010)
IHME 2010 − GPR
IHME 2010 − loess
IGME 2010 − loess
U5MR
0
50
100
150
200
1990
1995
2000
2005
2010
Year

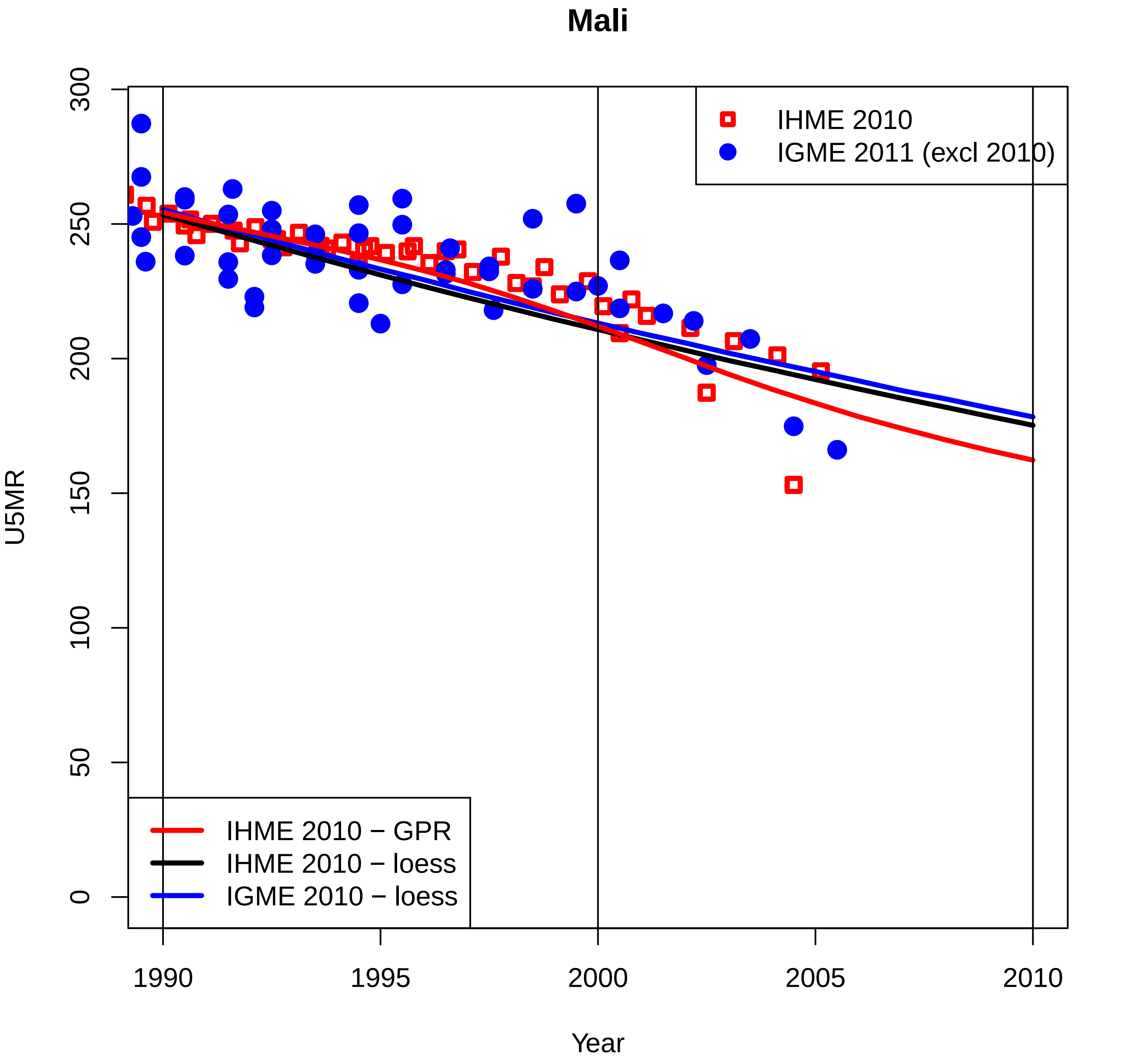

Mali
U5MR
Year
300
250
200
150
100
50
0
1990
1995
2000
2005
2010
IHME 2010
IGME 2011 (excl 2010)
IHME 2010 − GPR
IHME 2010 − loess
IGME 2010 − loess

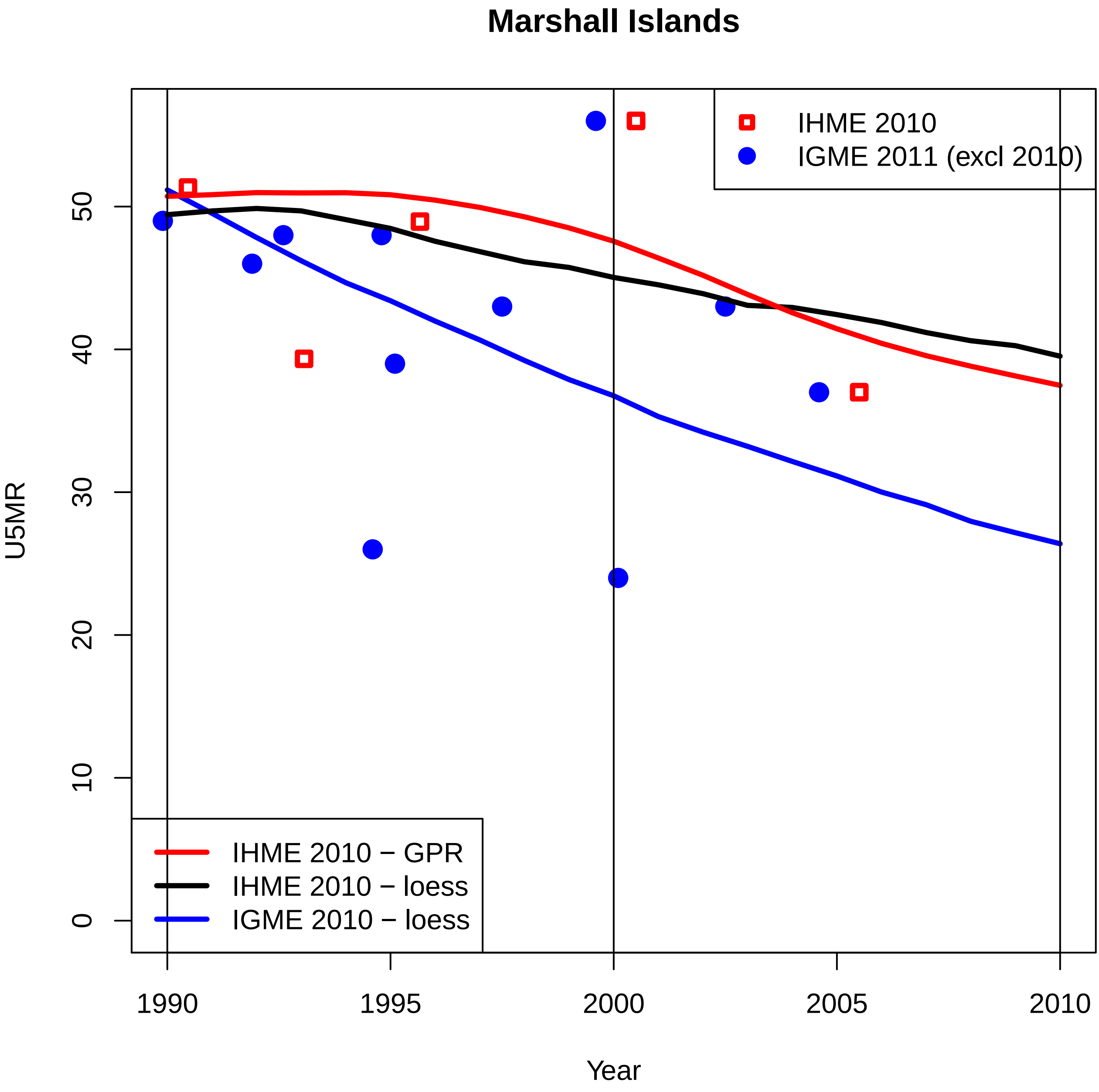
Marshall Islands
U5MR
Year
0
10
20
30
40
50
1990
1995
2000
2005
2010
IHME 2010
IGME 2011 (excl 2010)
IHME 2010 − GPR
IHME 2010 − loess
IGME 2010 − loess

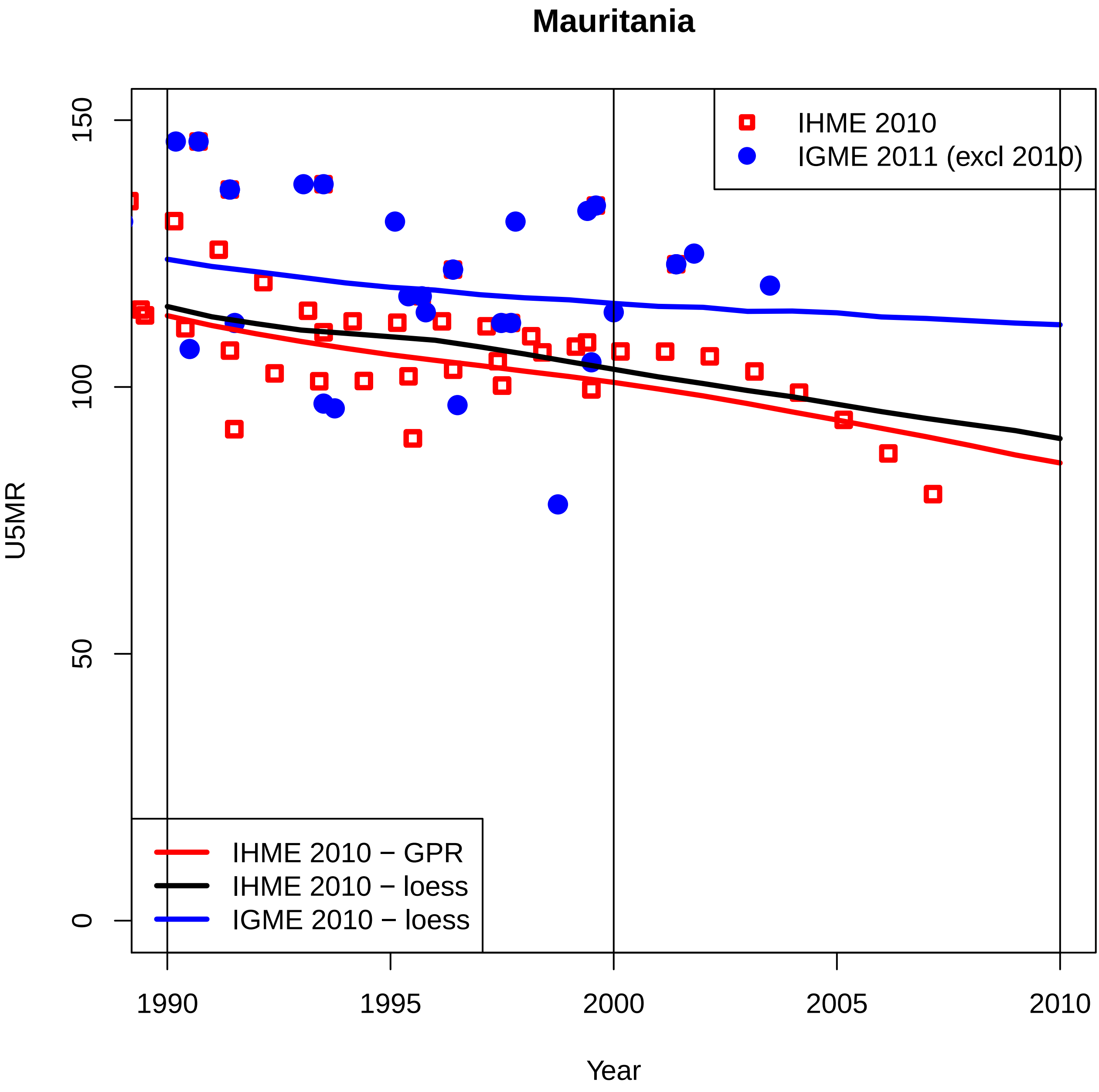
Mauritania
IHME 2010
IGME 2011 (excl 2010)
IHME 2010 − GPR
IHME 2010 − loess
IGME 2010 − loess
U5MR
0
50
100
150
1990
1995
2000
2005
2010
Year

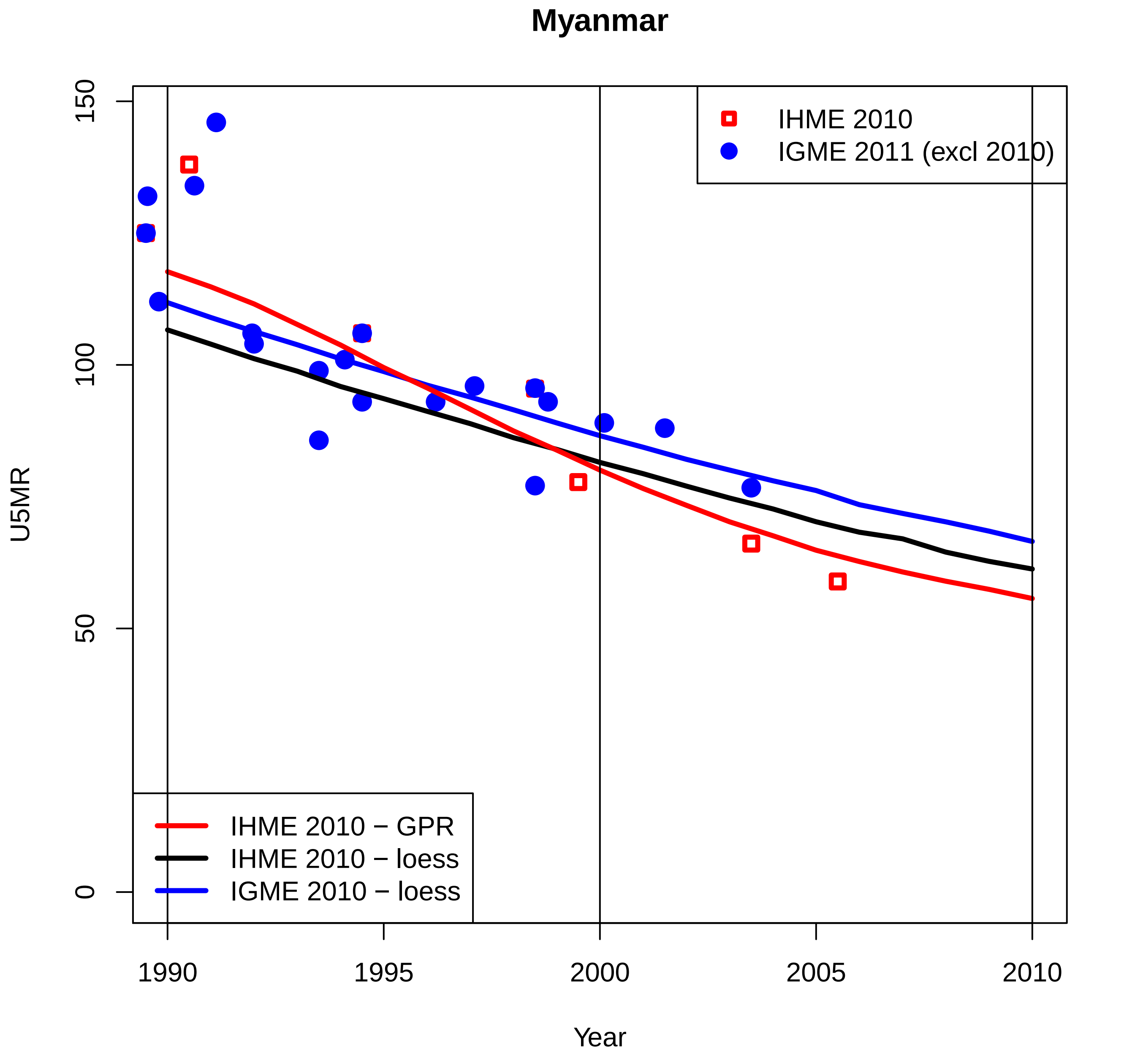

Myanmar
IHME 2010
IGME 2011 (excl 2010)
IHME 2010 − GPR
IHME 2010 − loess
IGME 2010 − loess
U5MR
0
50
100
150
1990
1995
2000
2005
2010
Year

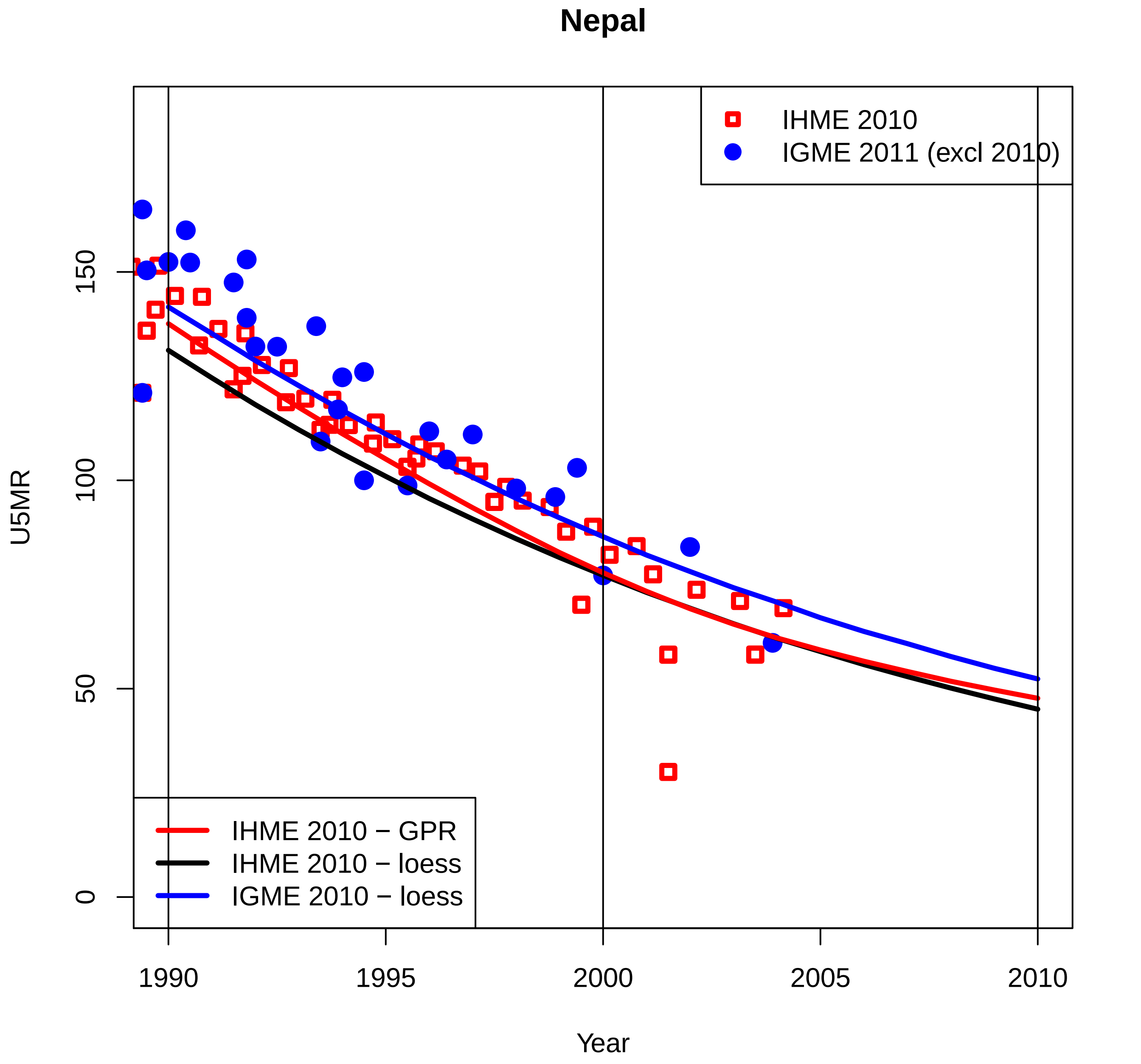

Nepal
U5MR
0
50
100
150
1990
1995
2000
2005
2010
Year
IHME 2010
IGME 2011 (excl 2010)
IHME 2010 − GPR
IHME 2010 − loess
IGME 2010 − loess

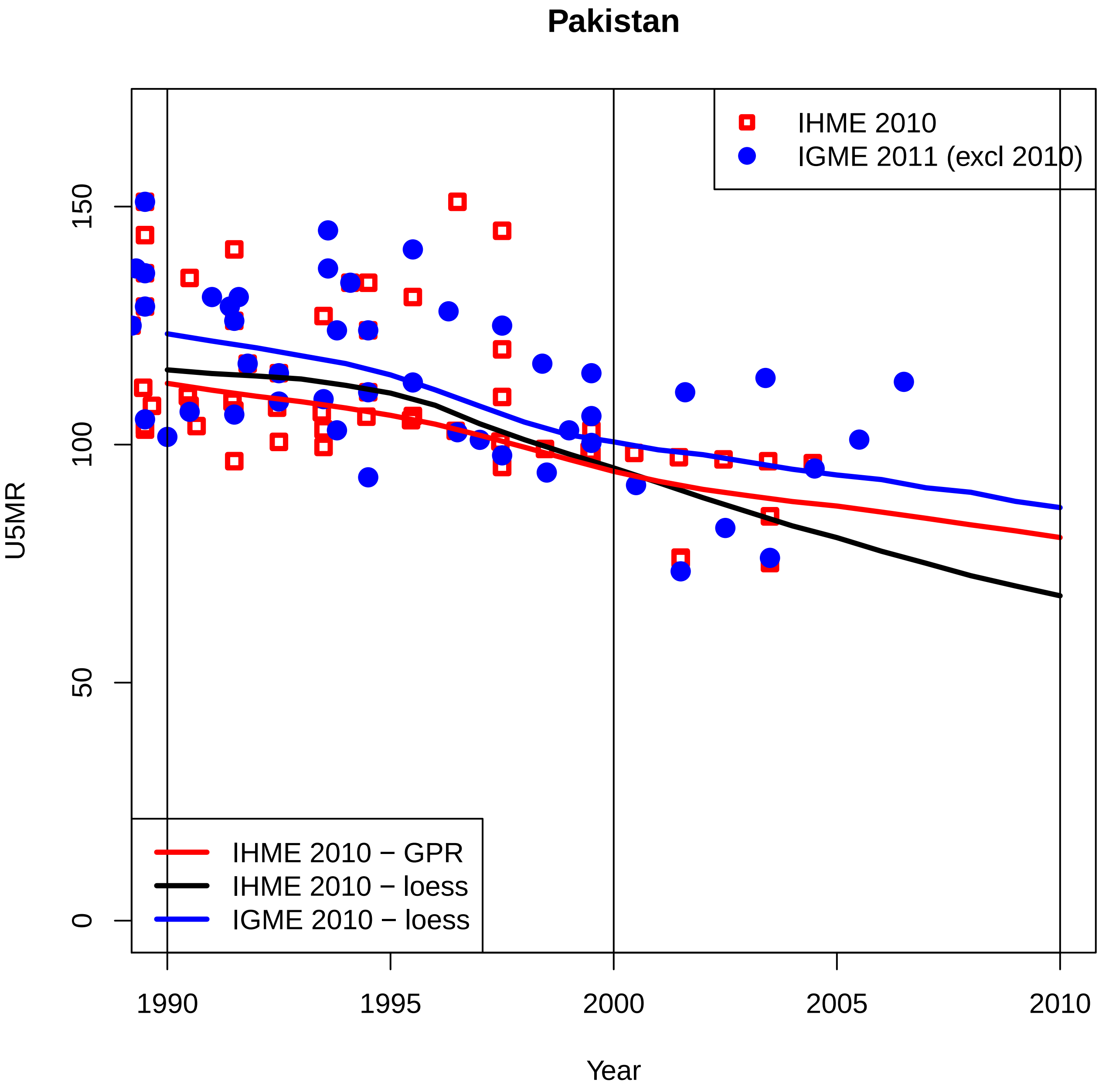
Pakistan
IHME 2010
IGME 2011 (excl 2010)
IHME 2010 − GPR
IHME 2010 − loess
IGME 2010 − loess
U5MR
0
50
100
150
1990
1995
2000
2005
2010
Year

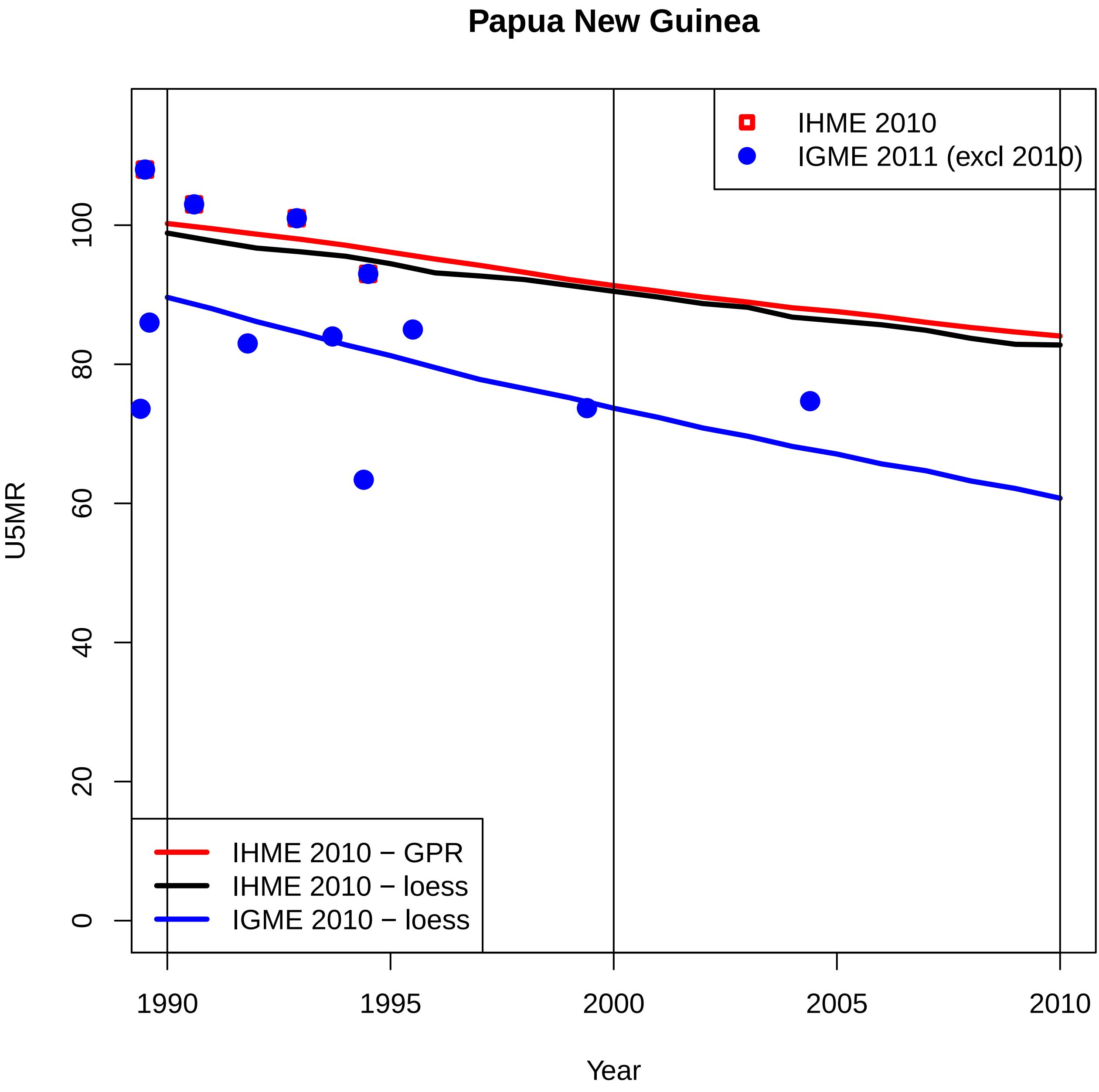

Papua New Guinea
IHME 2010
IGME 2011 (excl 2010)
IHME 2010 − GPR
IHME 2010 − loess
IGME 2010 − loess
U5MR
0
20
40
60
80
100
1990
1995
2000
2005
2010
Year

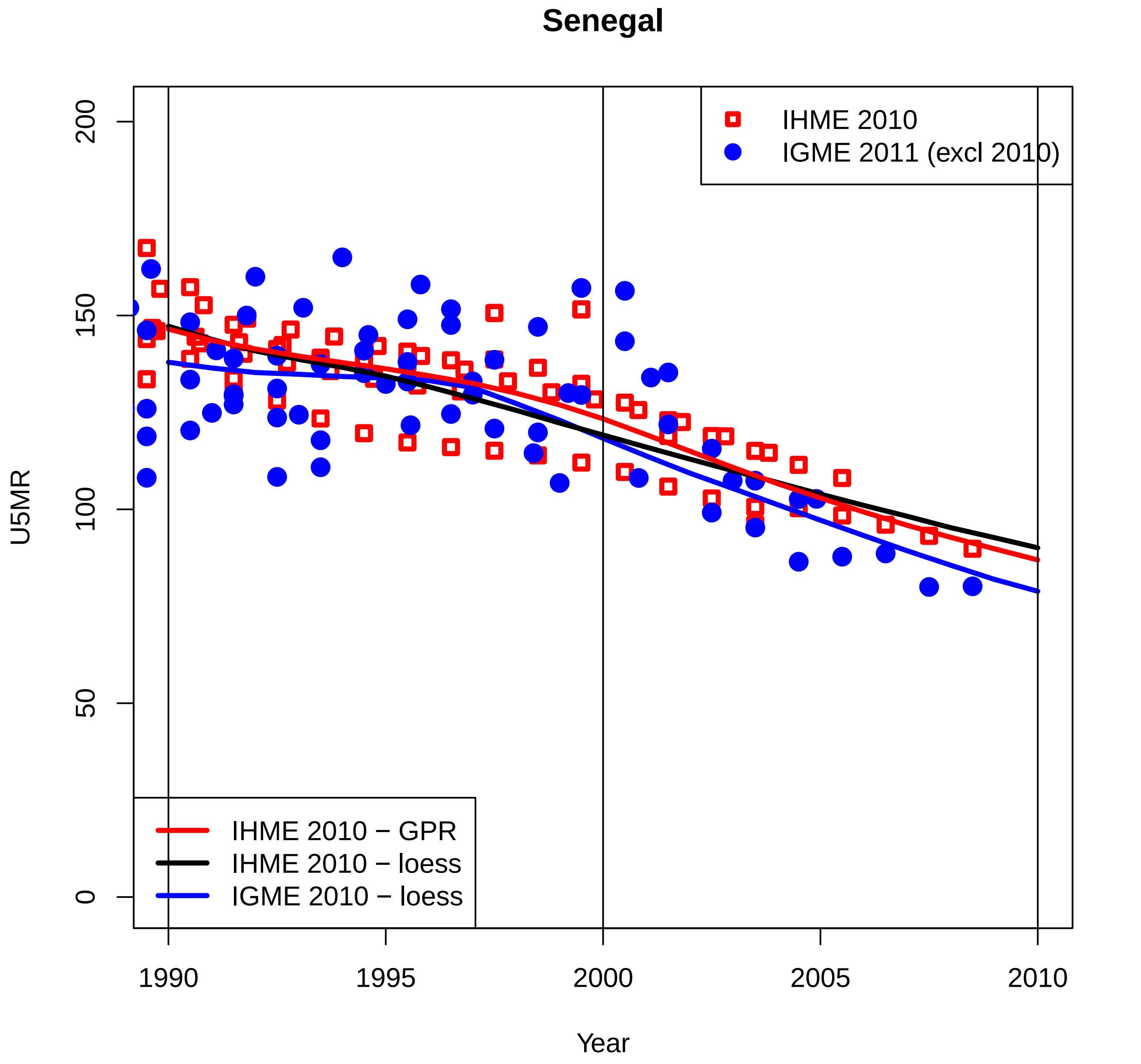

Senegal
IHME 2010
IGME 2011 (excl 2010)
IHME 2010 − GPR
IHME 2010 − loess
IGME 2010 − loess
U5MR
0
50
100
150
200
1990
1995
2000
2005
2010
Year

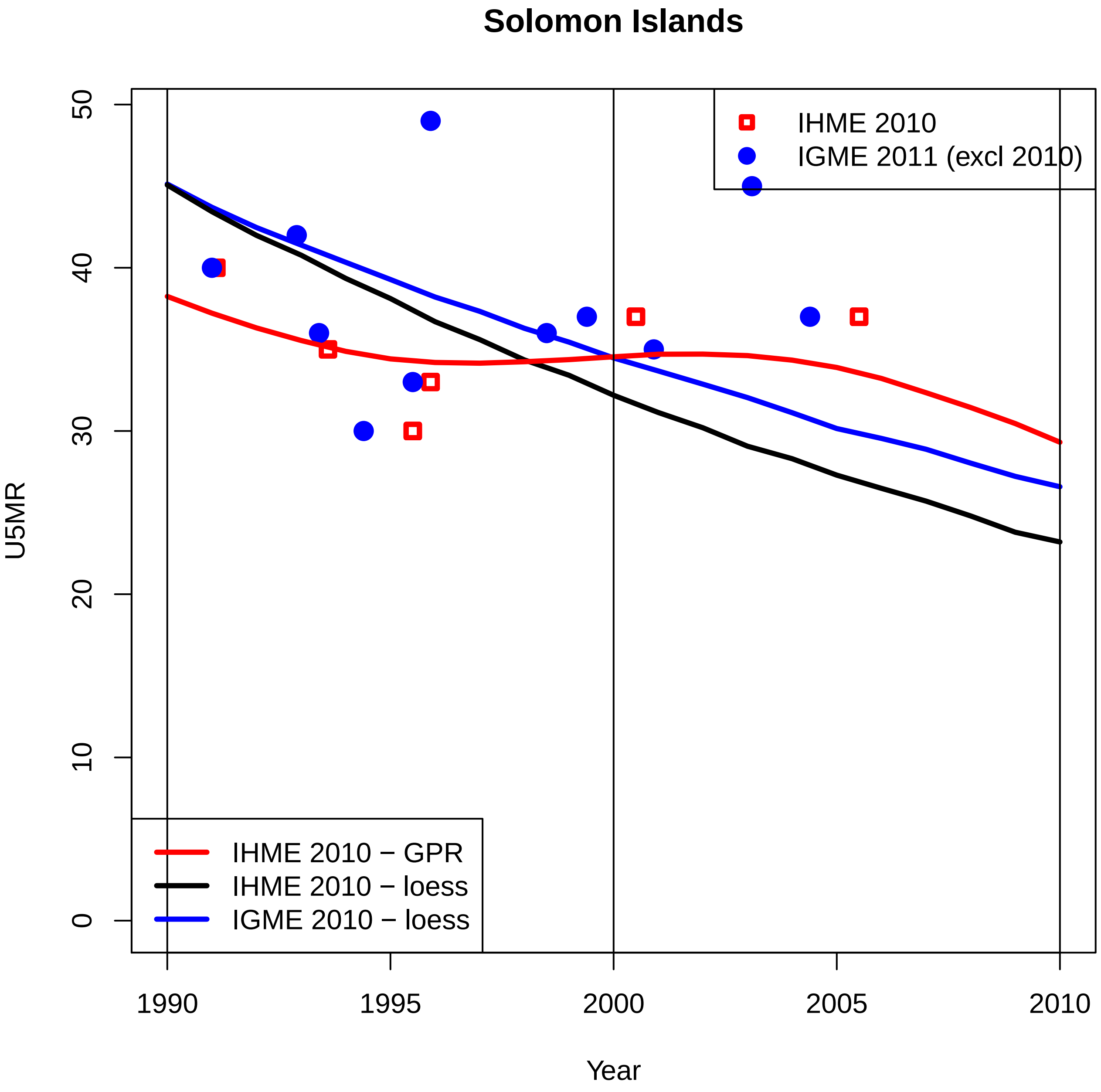
Solomon Islands
IHME 2010
IGME 2011 (excl 2010)
IHME 2010 − GPR
IHME 2010 − loess
IGME 2010 − loess
U5MR
0
10
20
30
40
50
1990
1995
2000
2005
2010
Year

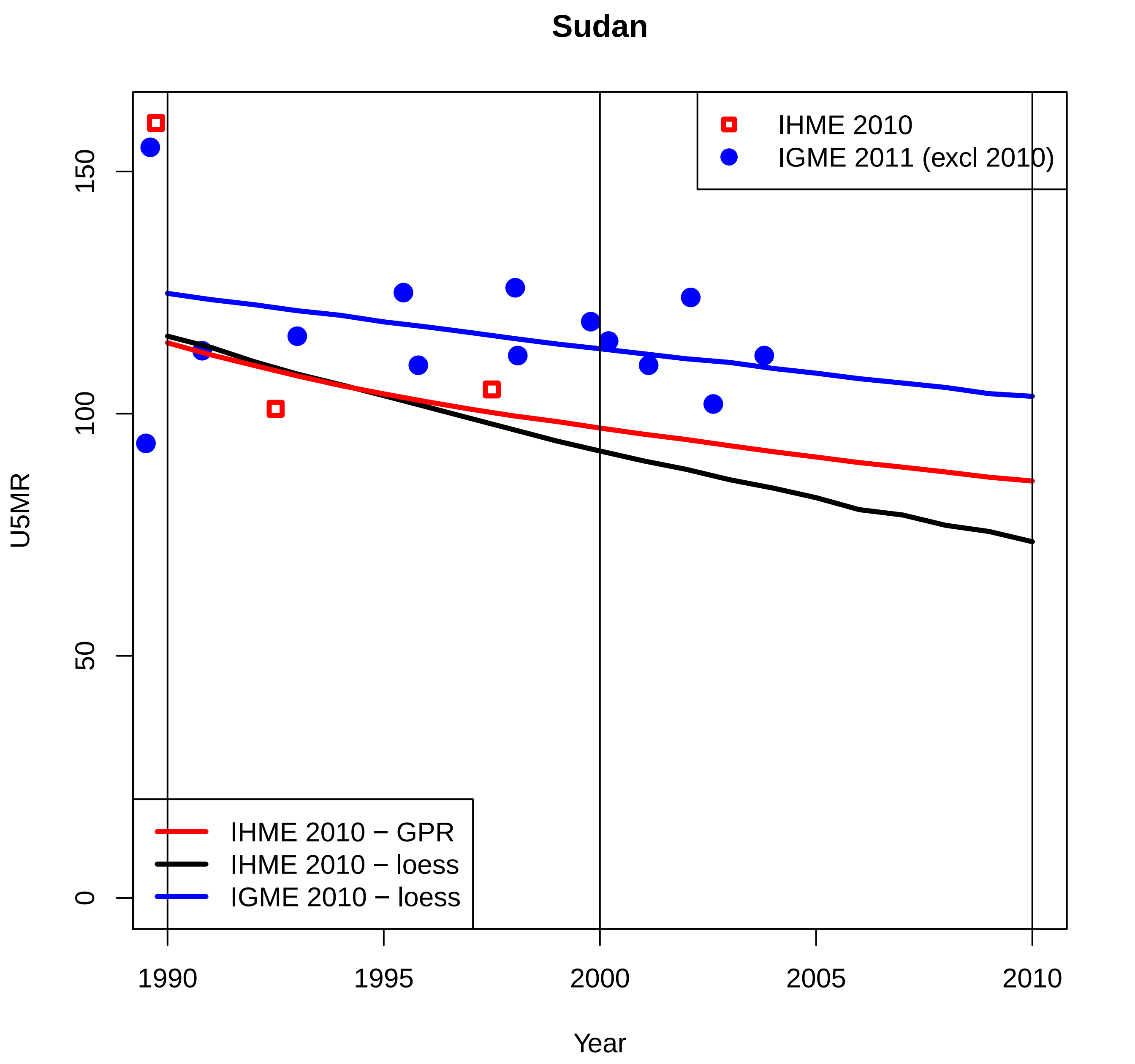

Sudan
U5MR
Year
0
50
100
150
1990
1995
2000
2005
2010
IHME 2010
IGME 2011 (excl 2010)
IHME 2010 − GPR
IHME 2010 − loess
IGME 2010 − loess

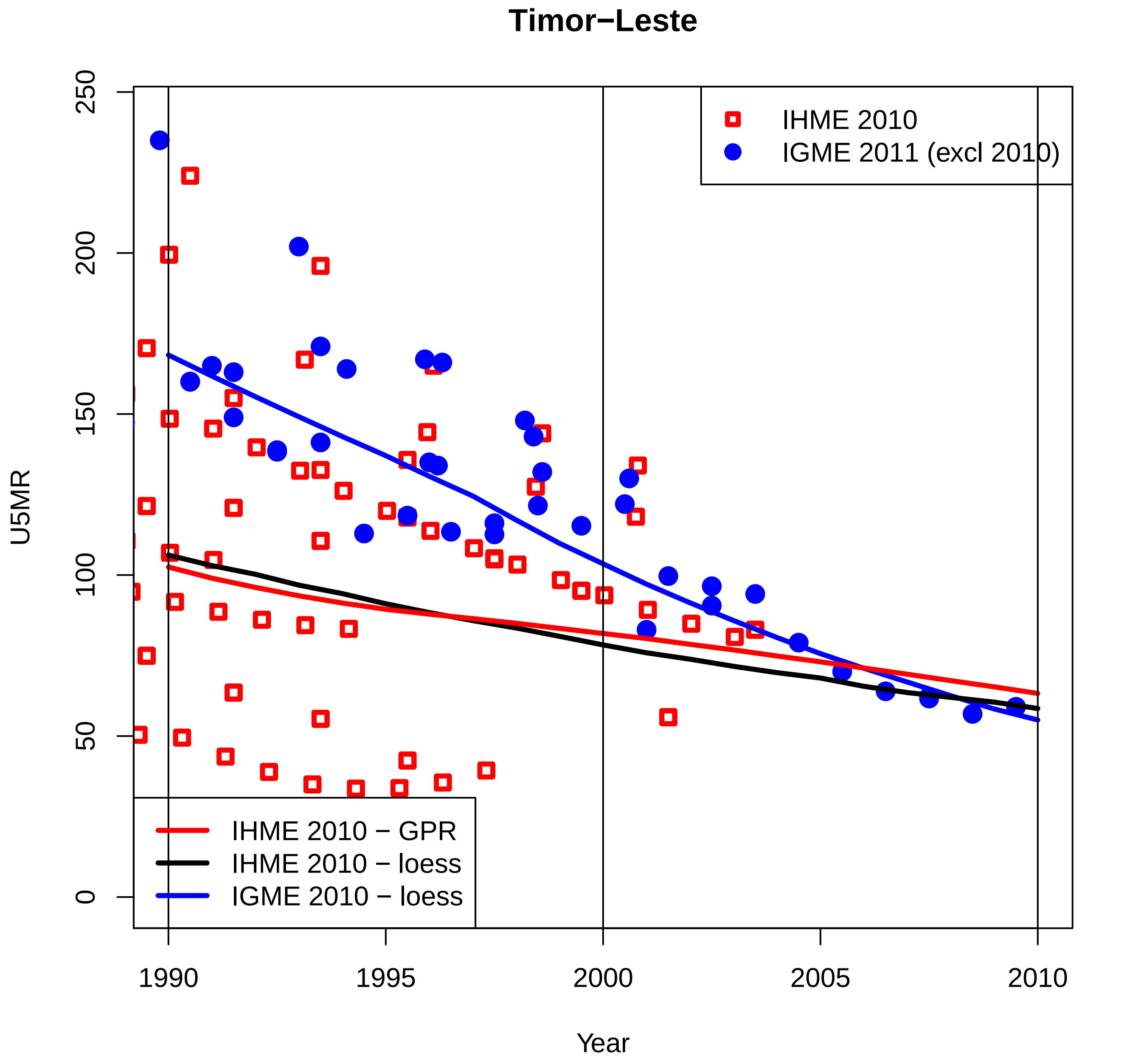

Timor−Leste
U5MR
Year
0
50
100
150
200
250
1990
1995
2000
2005
2010
IHME 2010
IGME 2011 (excl 2010)
IHME 2010 − GPR
IHME 2010 − loess
IGME 2010 − loess

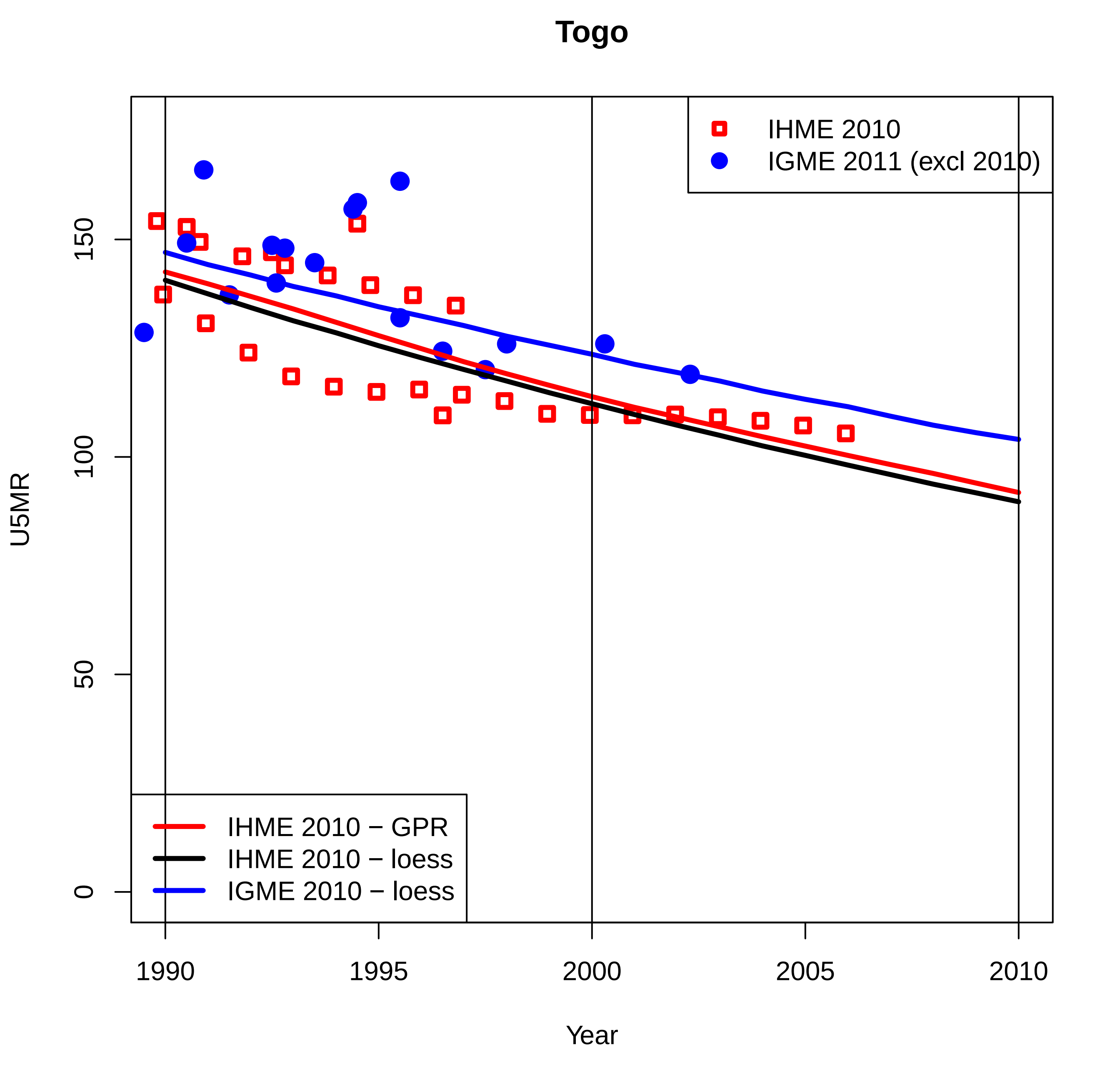

Togo
IHME 2010
IGME 2011 (excl 2010)
IHME 2010 − GPR
IHME 2010 − loess
IGME 2010 − loess
U5MR
0
50
100
150
1990
1995
2000
2005
2010
Year

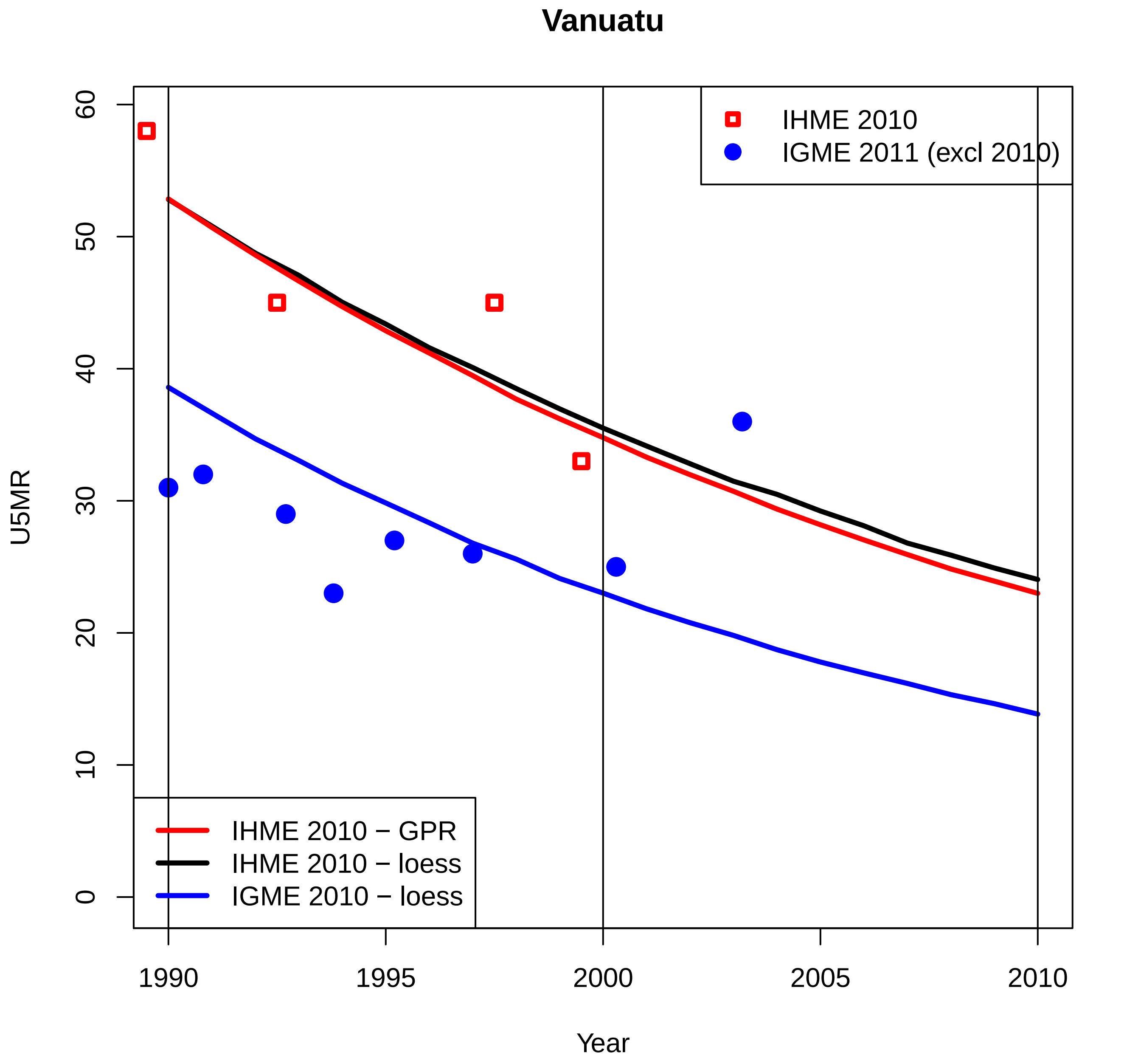

Vanuatu
U5MR
Year
0
10
20
30
40
50
60
1990
1995
2000
2005
2010
IHME 2010
IGME 2011 (excl 2010)
IHME 2010 − GPR
IHME 2010 − loess
IGME 2010 − loess

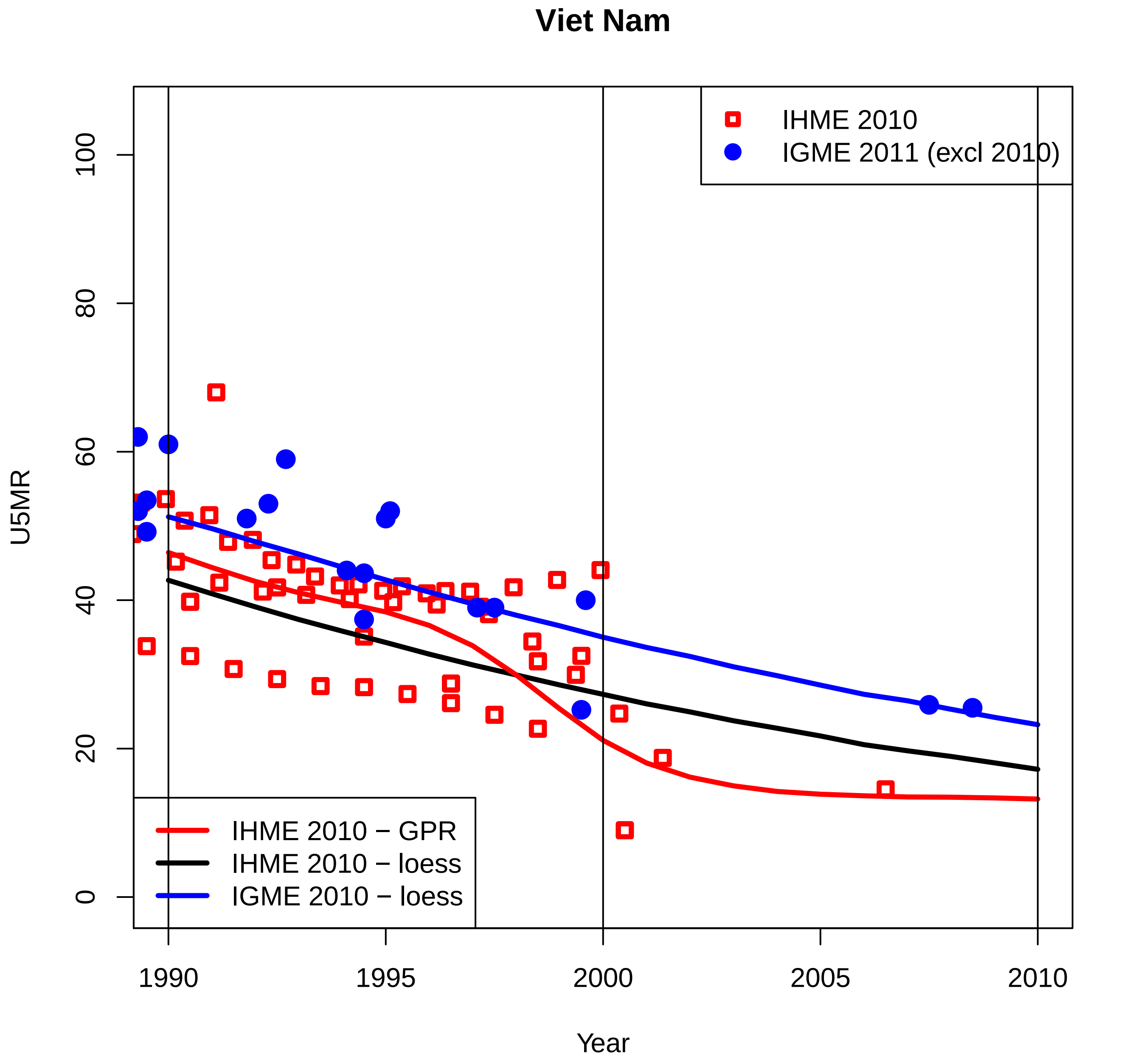

Viet Nam
IHME 2010
IGME 2011 (excl 2010)
IHME 2010 − GPR
IHME 2010 − loess
IGME 2010 − loess
U5MR
0
20
40
60
80
100
1990
1995
2000
2005
2010
Year

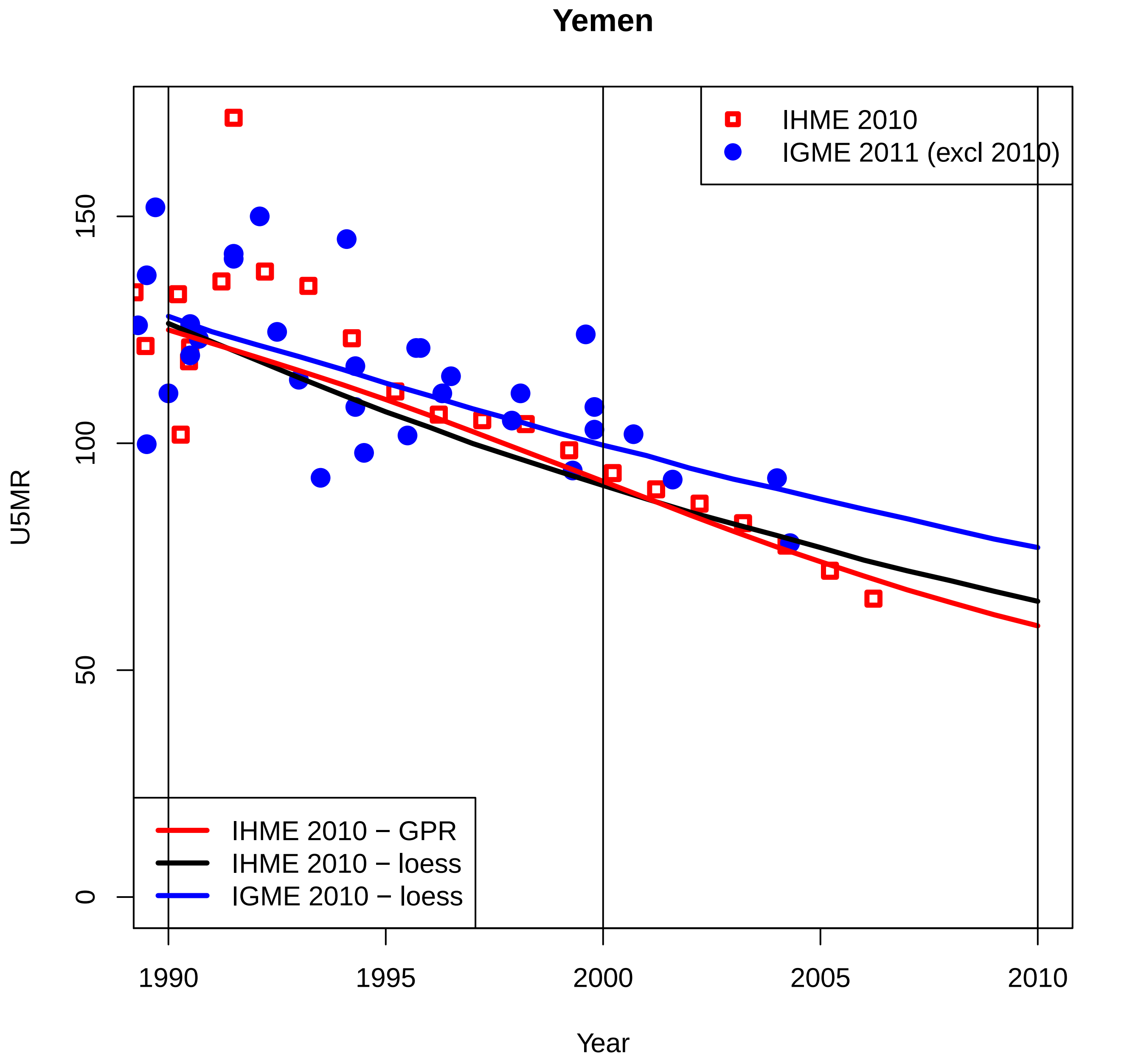

Yemen
IHME 2010
IGME 2011 (excl 2010)
IHME 2010 − GPR
IHME 2010 − loess
IGME 2010 − loess
U5MR
0
50
100
150
1990
1995
2000
2005
2010
Year
