# Supplementary material for: Child Mortality Estimation: A Comparison of UN IGME and IHME Estimates of Levels and Trends in Under-Five Mortality Rates and Deaths
Source: PLoS Med. 2012 Aug 28;9(8):e1001288. doi: 10.1371/journal.pmed.1001288 (PMC3429386; doi:10.1371/journal.pmed.1001288)
Supplement: Table S1 — Overview of UN IGME and IHME estimates and their (relative) differences for the U5MR and the number of under-five deaths for 1990, 2000, and 2010. Differences in U5MR estimates of more than 10% or more than ten deaths per 1,000 births, as well as relative differences in the estimates of under-five deaths of more than 10% or more than 10,000 deaths, are highlighted. (PDF) [file pmed.1001288.s003.pdf]

	1990 Under-five mortality rate (deaths per 1,000 live births)				2000 Under-five mortality rate (deaths per 1,000 live births)				2010 Under-five mortality rate (deaths per 1,000 live births)			
**Country**	**IGME**	**IHME**	**Diff**	**Diff (%)**	**IGME**	**IHME**	**Diff**	**Diff (%)**	**IGME**	**IHME**	**Diff**	**Diff (%)**
Afghanistan	208.7	156.8	-51.9	-24.9	151.1	154.3	3.2	2.1	149.2	113.4	-35.8	-24.0
Albania	41.1	40.5	-0.6	-1.5	28.6	24.5	-4.1	-14.3	18.4	15	-3.4	-18.5
Algeria	67.6	54.4	-13.2	-19.5	48.9	34.1	-14.8	-30.3	36	20.4	-15.6	-43.3
Andorra	8.8	7.8	-1	-11.4	4.9	5.5	0.6	12.2	3.8	3.8	0	0.0
Angola	243	239.4	-3.6	-1.5	199.6	200	0.4	0.2	160.5	137.2	-23.3	-14.5
Antigua and Barbuda	26.2	19.1	-7.1	-27.1	14.9	21.3	6.4	43.0	8.1	13.3	5.2	64.2
Argentina	26.9	28.5	1.6	5.9	19.6	19.4	-0.2	-1.0	13.8	14.7	0.9	6.5
Armenia	54.5	49.4	-5.1	-9.4	33.3	31.1	-2.2	-6.6	19.6	16.8	-2.8	-14.3
Australia	9.1	9.6	0.5	5.5	6.2	6.3	0.1	1.6	4.9	4.7	-0.2	-4.1
Austria	9.4	9.8	0.4	4.3	5.6	5.5	-0.1	-1.8	4.2	4.4	0.2	4.8
Azerbaijan	92.9	77.8	-15.1	-16.3	67.4	56.8	-10.6	-15.7	45.9	40.6	-5.3	-11.5
Bahamas	22	32	10	45.5	16.8	17.8	1	6.0	16.1	17.8	1.7	10.6
Bahrain	17	19.9	2.9	17.1	12.2	11.4	-0.8	-6.6	10.2	7.2	-3	-29.4
Bangladesh	143.4	137.2	-6.2	-4.3	85.7	85.9	0.2	0.2	47.8	56.4	8.6	18.0
Barbados	17.9	24.2	6.3	35.2	17.1	21.2	4.1	24.0	19.6	16.1	-3.5	-17.9
Belarus	17.2	21.7	4.5	26.2	13.7	17.5	3.8	27.7	6	9.6	3.6	60.0
Belgium	10	10.3	0.3	3.0	5.8	6.1	0.3	5.2	4.4	4.4	0	0.0
Belize	44	39.5	-4.5	-10.2	27	28.3	1.3	4.8	16.5	20.6	4.1	24.8
Benin	177.7	180.9	3.2	1.8	143.4	140.6	-2.8	-2.0	115.4	101.7	-13.7	-11.9
Bhutan	138.5	147.5	9	6.5	89	90.9	1.9	2.1	56.1	60.8	4.7	8.4
Bolivia	120.8	111	-9.8	-8.1	82.2	75.6	-6.6	-8.0	54.2	55.6	1.4	2.6
Bosnia and Herzegovina	18.8	18.8	0	0.0	9.6	10.7	1.1	11.5	8.4	6.6	-1.8	-21.4
Botswana	58.7	55.2	-3.5	-6.0	95.9	65.1	-30.8	-32.1	47.7	48.6	0.9	1.9
Brazil	59.3	53	-6.3	-10.6	35.7	31.5	-4.2	-11.8	19.4	21.8	2.4	12.4
Brunei Darussalam	12.2	11.3	-0.9	-7.4	9.3	9.4	0.1	1.1	7.3	7.2	-0.1	-1.4
Bulgaria	22.2	18.6	-3.6	-16.2	20.7	17.3	-3.4	-16.4	12.7	10.2	-2.5	-19.7
Burkina Faso	204.5	212	7.5	3.7	190.8	182.9	-7.9	-4.1	176.2	160.1	-16.1	-9.1
Burundi	183.2	189	5.8	3.2	163.8	163.3	-0.5	-0.3	141.9	120.6	-21.3	-15.0
Cambodia	121	114.1	-6.9	-5.7	103.1	102.3	-0.8	-0.8	51	53.9	2.9	5.7
Cameroon	136.6	147.2	10.6	7.8	147.5	144	-3.5	-2.4	136.2	113.9	-22.3	-16.4
Canada	8.3	8.4	0.1	1.2	6.2	6.1	-0.1	-1.6	5.9	4.9	-1	-16.9
Cape Verde	59.2	60.5	1.3	2.2	46	47.9	1.9	4.1	35.6	33.3	-2.3	-6.5
Central African Republic	165.2	171.2	6	3.6	175.9	176.5	0.6	0.3	158.8	158.3	-0.5	-0.3
Chad	207.3	213.9	6.6	3.2	189.5	192.1	2.6	1.4	173.4	170.4	-3	-1.7
Chile	18.7	18.1	-0.6	-3.2	10.8	10.9	0.1	0.9	8.8	6.8	-2	-22.7
China	48.3	40.8	-7.5	-15.5	33	31.1	-1.9	-5.8	18.4	14.6	-3.8	-20.7
Colombia	36.6	33.7	-2.9	-7.9	26.8	26.8	0	0.0	19.1	22.6	3.5	18.3

Comoros	125.2	117.8	-7.4	-5.9	104.3	85.4	-18.9	-18.1	85.6	64.6	-21	-24.5
Congo	116.3	105.7	-10.6	-9.1	104.2	116.4	12.2	11.7	93.4	102.7	9.3	10.0
Congo, Republic of the	181.4	182.6	1.2	0.7	181.4	167.6	-13.8	-7.6	169.9	132	-37.9	-22.3
Costa Rica	17.2	20.9	3.7	21.5	12.9	14.1	1.2	9.3	10.1	8.4	-1.7	-16.8
Côte d'Ivoire	151.4	152.9	1.5	1.0	147.7	141.3	-6.4	-4.3	123	108.9	-14.1	-11.5
Croatia	12.9	12.8	-0.1	-0.8	8.4	8.6	0.2	2.4	5.5	5.8	0.3	5.5
Cuba	13.4	13.6	0.2	1.5	8.5	8.5	0	0.0	5.9	5.3	-0.6	-10.2
Cyprus	11	12.1	1.1	10.0	6.5	6.3	-0.2	-3.1	3.8	3.6	-0.2	-5.3
Czech Republic	14.3	12.3	-2	-14.0	6.7	5.4	-1.3	-19.4	4	3.6	-0.4	-10.0
Denmark	8.7	9.3	0.6	6.9	5.6	5.6	0	0.0	3.9	4.4	0.5	12.8
Djibouti	122.7	113.7	-9	-7.3	105.6	91.7	-13.9	-13.2	91.1	69.3	-21.8	-23.9
Dominica	17.4	21.9	4.5	25.9	14.9	22.7	7.8	52.3	12.4	19.3	6.9	55.6
Dominican Republic	62.2	57.8	-4.4	-7.1	40.6	38.2	-2.4	-5.9	26.5	28.9	2.4	9.1
Ecuador	52.3	50.1	-2.2	-4.2	32.7	36.1	3.4	10.4	20.1	20.5	0.4	2.0
Egypt	93.5	83.5	-10	-10.7	46.5	45.3	-1.2	-2.6	21.8	24.1	2.3	10.6
El Salvador	61.9	58	-3.9	-6.3	34	34.3	0.3	0.9	16.2	17.2	1	6.2
Equatorial Guinea	189.5	183.8	-5.7	-3.0	152.3	189.7	37.4	24.6	120.8	191.5	70.7	58.5
Eritrea	140.6	146.6	6	4.3	92.7	94.7	2	2.2	60.8	72.2	11.4	18.8
Estonia	21.1	21	-0.1	-0.5	13	12.5	-0.5	-3.8	5.4	6.8	1.4	25.9
Ethiopia	184.1	203.8	19.7	10.7	140.7	135	-5.7	-4.1	105.9	99.2	-6.7	-6.3
Fiji	29.5	33.3	3.8	12.9	22.6	32.3	9.7	42.9	17.4	28	10.6	60.9
Finland	6.7	6.9	0.2	3.0	4.3	4.2	-0.1	-2.3	3	3	0	0.0
France	8.8	9	0.2	2.3	5.4	5.5	0.1	1.9	4.1	4	-0.1	-2.4
Gabon	92.6	93.3	0.7	0.8	87.5	84.6	-2.9	-3.3	73.5	68.5	-5	-6.8
Gambia	165.2	158.9	-6.3	-3.8	127.5	134.2	6.7	5.3	98.1	97.7	-0.4	-0.4
Georgia	46.8	43.6	-3.2	-6.8	33.4	38.5	5.1	15.3	22.4	27.7	5.3	23.7
Germany	8.5	9.3	0.8	9.4	5.4	5.4	0	0.0	4.1	4	-0.1	-2.4
Ghana	121.7	121.8	0.1	0.1	99.1	102.2	3.1	3.1	74.4	63.8	-10.6	-14.2
Greece	12.7	10.5	-2.2	-17.3	7.7	6.2	-1.5	-19.5	4.1	3.6	-0.5	-12.2
Grenada	21.2	26.6	5.4	25.5	15.1	21.2	6.1	40.4	11.1	14.3	3.2	28.8
Guatemala	77.9	77	-0.9	-1.2	48.5	51.4	2.9	6.0	31.8	34.3	2.5	7.9
Guinea	229.2	228.5	-0.7	-0.3	175	177.2	2.2	1.3	129.9	134.5	4.6	3.5
Guinea-Bissau	209.9	235.1	25.2	12.0	176.8	203	26.2	14.8	149.5	172.4	22.9	15.3
Guyana	65.8	59.5	-6.3	-9.6	46.8	46.3	-0.5	-1.1	30.4	39.8	9.4	30.9
Haiti	150.5	148.3	-2.2	-1.5	109	99.5	-9.5	-8.7	164.8	109.2	-55.6	-33.7
Honduras	58.1	56.4	-1.7	-2.9	37.4	37.6	0.2	0.5	24	22.9	-1.1	-4.6
Hungary	18.7	16.2	-2.5	-13.4	11	9.8	-1.2	-10.9	6.4	5.8	-0.6	-9.4
Iceland	6.3	6.8	0.5	7.9	3.9	3.9	0	0.0	2.4	2.7	0.3	12.5
India	114.8	114.2	-0.6	-0.5	85.5	84.9	-0.6	-0.7	62.7	62.6	-0.1	-0.2
Indonesia	85	75.4	-9.6	-11.3	54	51.1	-2.9	-5.4	35.3	38.8	3.5	9.9
Iran (Islamic Republic of)	64.8	70.6	5.8	9.0	43.9	45.4	1.5	3.4	25.8	25.5	-0.3	-1.2
Iraq	46.1	57.1	11	23.9	42.8	42	-0.8	-1.9	38.6	35.8	-2.8	-7.3
Ireland	9	9.6	0.6	6.7	7	7	0	0.0	3.9	4.2	0.3	7.7

Israel	11.5	12.1	0.6	5.2	6.9	7.3	0.4	5.8	4.5	4.7	0.2	4.4
Italy	9.6	9.5	-0.1	-1.0	5.6	5.5	-0.1	-1.8	3.7	3.6	-0.1	-2.7
Jamaica	38	34.7	-3.3	-8.7	30.3	26.1	-4.2	-13.9	23.8	18.2	-5.6	-23.5
Japan	6.4	6	-0.4	-6.3	4.5	4.5	0	0.0	3.2	3	-0.2	-6.3
Jordan	38.3	35.1	-3.2	-8.4	29.4	27	-2.4	-8.2	21.7	23.8	2.1	9.7
Kazakhstan	57.4	50.6	-6.8	-11.8	43.9	45.6	1.7	3.9	33.2	30.4	-2.8	-8.4
Kenya	99.4	100.7	1.3	1.3	111.1	95	-16.1	-14.5	84.7	70.7	-14	-16.5
Kiribati	87.4	80.8	-6.6	-7.6	65.2	59.2	-6	-9.2	48.6	46.5	-2.1	-4.3
Korea, North	45	38.2	-6.8	-15.1	57.7	31.9	-25.8	-44.7	33.2	23.7	-9.5	-28.6
Korea, South	7.5	10.8	3.3	44.0	5.8	7.1	1.3	22.4	4.9	3.6	-1.3	-26.5
Kuwait	15.4	21.3	5.9	38.3	12.6	17	4.4	34.9	11.1	14.2	3.1	27.9
Kyrgyzstan	71.8	69.1	-2.7	-3.8	51.9	51.4	-0.5	-1.0	37.7	45.7	8	21.2
Lao People's Democratic Republic	144.8	141.8	-3	-2.1	88.1	99.3	11.2	12.7	53.8	71.4	17.6	32.7
Latvia	20.6	18	-2.6	-12.6	17.3	14.1	-3.2	-18.5	9.6	8.9	-0.7	-7.3
Lebanon	38.3	30.4	-7.9	-20.6	29.3	17.9	-11.4	-38.9	22.1	11.2	-10.9	-49.3
Lesotho	89.3	90.1	0.8	0.9	126.8	98.7	-28.1	-22.2	85	104.4	19.4	22.8
Liberia	226.6	252.6	26	11.5	168.9	166.7	-2.2	-1.3	102.6	104.8	2.2	2.1
Libyan Arab Jamahiriya	44.5	39.9	-4.6	-10.3	27.2	23.8	-3.4	-12.5	16.9	14.2	-2.7	-16.0
Lithuania	17.4	15.4	-2	-11.5	11.8	10.7	-1.1	-9.3	6.5	6.3	-0.2	-3.1
Luxembourg	8.4	9.3	0.9	10.7	5	5.2	0.2	4.0	3.1	2.8	-0.3	-9.7
Macedonia	39.1	26.6	-12.5	-32.0	16	18.5	2.5	15.6	11.7	11.2	-0.5	-4.3
Madagascar	158.8	153.6	-5.2	-3.3	101.5	99.6	-1.9	-1.9	62.1	61	-1.1	-1.8
Malawi	222.1	209.8	-12.3	-5.5	166.5	149.5	-17	-10.2	92.1	101.8	9.7	10.5
Malaysia	17.9	17.2	-0.7	-3.9	10.6	9.1	-1.5	-14.2	6.3	5	-1.3	-20.6
Maldives	101.6	74.4	-27.2	-26.8	46.6	39.1	-7.5	-16.1	15	15.4	0.4	2.7
Mali	255.4	250.6	-4.8	-1.9	213.2	216.9	3.7	1.7	178.1	159.9	-18.2	-10.2
Malta	11.3	10.3	-1	-8.8	7.7	7.3	-0.4	-5.2	6	6.8	0.8	13.3
Marshall Islands	51.1	43.2	-7.9	-15.5	36.8	40.8	4	10.9	26.3	41.8	15.5	58.9
Mauritania	124.1	106.4	-17.7	-14.3	115.6	102.3	-13.3	-11.5	111.2	80.6	-30.6	-27.5
Mauritius	23.9	21.3	-2.6	-10.9	18.6	17.2	-1.4	-7.5	15.1	13.6	-1.5	-9.9
Mexico	48.6	39.8	-8.8	-18.1	29.1	25.2	-3.9	-13.4	16.7	18.7	2	12.0
Micronesia (Fed. States of)	56.4	51.8	-4.6	-8.2	48.7	23.6	-25.1	-51.5	42.1	11.9	-30.2	-71.7
Moldova	37.1	30.8	-6.3	-17.0	26.4	23.2	-3.2	-12.1	19	12.8	-6.2	-32.6
Mongolia	106.9	94.4	-12.5	-11.7	60.9	56.4	-4.5	-7.4	31.6	33.5	1.9	6.0
Montenegro	17.6	26	8.4	47.7	12.6	13.9	1.3	10.3	8	7.6	-0.4	-5.0
Morocco	85.9	77	-8.9	-10.4	55.3	50.4	-4.9	-8.9	35.5	34.6	-0.9	-2.5
Mozambique	218.7	226.2	7.5	3.4	176.7	172.2	-4.5	-2.5	135	138.7	3.7	2.7
Myanmar	111.7	107.2	-4.5	-4.0	86.7	75.6	-11.1	-12.8	66.2	57	-9.2	-13.9
Namibia	73.1	72.5	-0.6	-0.8	73.9	63	-10.9	-14.7	40.1	59.2	19.1	47.6
Nepal	140.9	138.6	-2.3	-1.6	84.4	79.5	-4.9	-5.8	49.5	52.1	2.6	5.3
Netherlands	8.3	8.8	0.5	6.0	6.2	6.5	0.3	4.8	4.3	4.4	0.1	2.3
New Zealand	11.1	11.3	0.2	1.8	7.4	7.6	0.2	2.7	6	6.1	0.1	1.7
Nicaragua	68	66.6	-1.4	-2.1	42.7	40.4	-2.3	-5.4	26.9	30.3	3.4	12.6

Niger	311	300.2	-10.8	-3.5	217.8	227.5	9.7	4.5	143.3	164	20.7	14.4
Nigeria	212.6	216.4	3.8	1.8	186	186.8	0.8	0.4	142.9	143.3	0.4	0.3
Norway	8.5	8.9	0.4	4.7	4.9	4.9	0	0.0	3.4	3.5	0.1	2.9
Occupied Palestinian Territory	44.7	42.8	-1.9	-4.3	30.6	34.5	3.9	12.7	22.3	23.1	0.8	3.6
Oman	47.2	38.7	-8.5	-18.0	21.5	10.6	-10.9	-50.7	9.3	10.9	1.6	17.2
Pakistan	123.6	113	-10.6	-8.6	100.5	94.6	-5.9	-5.9	86.5	84.7	-1.8	-2.1
Panama	33.2	27.4	-5.8	-17.5	25.7	23.2	-2.5	-9.7	20.2	16.7	-3.5	-17.3
Papua New Guinea	89.5	88.7	-0.8	-0.9	73.9	74.9	1	1.4	60.8	66.8	6	9.9
Paraguay	50.1	37.7	-12.4	-24.8	35.3	28	-7.3	-20.7	24.6	22.5	-2.1	-8.5
Peru	77.6	71.8	-5.8	-7.5	40.6	40	-0.6	-1.5	19.2	25.3	6.1	31.8
Philippines	59.4	54.1	-5.3	-8.9	40.4	38.8	-1.6	-4.0	29.4	27.6	-1.8	-6.1
Poland	17.3	17.7	0.4	2.3	9.6	9.4	-0.2	-2.1	6	6.6	0.6	10.0
Portugal	14.6	14.1	-0.5	-3.4	7.2	7.3	0.1	1.4	3.7	4	0.3	8.1
Qatar	20.8	19.4	-1.4	-6.7	12.7	16.6	3.9	30.7	8.2	11.9	3.7	45.1
Romania	37.4	31	-6.4	-17.1	26.8	22.4	-4.4	-16.4	13.6	13.2	-0.4	-2.9
Russian Federation	26.5	27	0.5	1.9	22.7	22	-0.7	-3.1	11.6	14	2.4	20.7
Rwanda	162.8	159.3	-3.5	-2.1	176.7	163.2	-13.5	-7.6	91.1	77.4	-13.7	-15.0
Saint Lucia	22.5	20.4	-2.1	-9.3	18.1	16	-2.1	-11.6	15.9	13.9	-2	-12.6
Saint Vincent and the Grenadines	26.5	24.5	-2	-7.5	21.9	23.4	1.5	6.8	21.2	22.1	0.9	4.2
Samoa	27.1	27.8	0.7	2.6	23.1	24.8	1.7	7.4	20.2	17.4	-2.8	-13.9
Sao Tome and Principe	93.8	106.4	12.6	13.4	87	80.5	-6.5	-7.5	79.9	54.3	-25.6	-32.0
Saudi Arabia	44.6	22.3	-22.3	-50.0	25.8	18.7	-7.1	-27.5	17.5	16.8	-0.7	-4.0
Senegal	138.6	141.4	2.8	2.0	118.8	123.4	4.6	3.9	75.2	79.8	4.6	6.1
Serbia	28.6	12.7	-15.9	-55.6	12.7	7.9	-4.8	-37.8	7.1	4.3	-2.8	-39.4
Seychelles	16.6	17.1	0.5	3.0	13.8	14.5	0.7	5.1	13.5	13.7	0.2	1.5
Sierra Leone	276	252.5	-23.5	-8.5	232.8	216.1	-16.7	-7.2	174	128.9	-45.1	-25.9
Singapore	7.5	7.8	0.3	4.0	3.9	3.6	-0.3	-7.7	2.6	3	0.4	15.4
Slovakia	17.6	14.1	-3.5	-19.9	11.6	9.5	-2.1	-18.1	8.2	7.4	-0.8	-9.8
Slovenia	10.4	10.3	-0.1	-1.0	5.4	5.5	0.1	1.9	2.9	3.1	0.2	6.9
Solomon Islands	44.9	38.1	-6.8	-15.1	34.5	35.2	0.7	2.0	26.7	28	1.3	4.9
Somalia	180	186.2	6.2	3.4	180	144.8	-35.2	-19.6	180	101.9	-78.1	-43.4
South Africa	59.8	60.3	0.5	0.8	77.9	36.1	-41.8	-53.7	56.6	52.3	-4.3	-7.6
Spain	10.9	9.4	-1.5	-13.8	6.6	5.4	-1.2	-18.2	4.8	3.9	-0.9	-18.8
Sri Lanka	31.5	29.4	-2.1	-6.7	22.7	17	-5.7	-25.1	16.5	10.9	-5.6	-33.9
Sudan	124.9	113.4	-11.5	-9.2	113.5	100.3	-13.2	-11.6	103.3	83.1	-20.2	-19.6
Suriname	51.8	51.4	-0.4	-0.8	39.8	42.1	2.3	5.8	30.5	38.8	8.3	27.2
Swaziland	96.1	72.4	-23.7	-24.7	114	98.5	-15.5	-13.6	77.7	101.3	23.6	30.4
Sweden	6.7	7.3	0.6	9.0	4.1	4	-0.1	-2.4	3	2.9	-0.1	-3.3
Switzerland	8.1	8.6	0.5	6.2	5.7	5.8	0.1	1.8	4.6	4.3	-0.3	-6.5
Syrian Arab Republic	38.2	34.7	-3.5	-9.2	23	19.4	-3.6	-15.7	16	11.1	-4.9	-30.6
Tajikistan	116.4	104.8	-11.6	-10.0	93.4	76.2	-17.2	-18.4	62.6	45.6	-17	-27.2
Tanzania	154.8	153.3	-1.5	-1.0	130.2	125.3	-4.9	-3.8	75.8	83.5	7.7	10.2
Thailand	31.8	22.6	-9.2	-28.9	17.7	14.2	-3.5	-19.8	13	10.4	-2.6	-20.0

Timor-Leste	168.7	115.7	-53	-31.4	103.5	99.5	-4	-3.9	54.8	54.6	-0.2	-0.4
Togo	147.1	148.4	1.3	0.9	123.7	123.3	-0.4	-0.3	103.4	110.2	6.8	6.6
Tonga	24.5	27.2	2.7	11.0	19.7	26.4	6.7	34.0	15.6	23.3	7.7	49.4
Trinidad and Tobago	36.6	30.2	-6.4	-17.5	31.6	32.2	0.6	1.9	27.1	25.2	-1.9	-7.0
Tunisia	49.3	48.6	-0.7	-1.4	28.4	27.2	-1.2	-4.2	16.1	14.4	-1.7	-10.6
Turkey	79.8	71.1	-8.7	-10.9	42.7	41	-1.7	-4.0	17.6	31.3	13.7	77.8
Turkmenistan	98.3	106.3	8	8.1	74	70.4	-3.6	-4.9	55.5	30.9	-24.6	-44.3
Uganda	174.9	165.3	-9.6	-5.5	144.3	136.1	-8.2	-5.7	98.9	93.3	-5.6	-5.7
UK	9.3	9.6	0.3	3.2	6.6	6.3	-0.3	-4.5	5.4	5.5	0.1	1.9
Ukraine	20.7	19.9	-0.8	-3.9	17.6	19.3	1.7	9.7	13.2	12.7	-0.5	-3.8
United Arab Emirates	21.5	15.1	-6.4	-29.8	12.4	8.2	-4.2	-33.9	7.1	3.6	-3.5	-49.3
Uruguay	23.1	23.1	0	0.0	17.1	16	-1.1	-6.4	10.8	11.7	0.9	8.3
USA	11.3	11.5	0.2	1.8	8.5	8.4	-0.1	-1.2	7.5	7.3	-0.2	-2.7
Uzbekistan	77.3	59.4	-17.9	-23.2	62.9	54.8	-8.1	-12.9	51.5	41.7	-9.8	-19.0
Vanuatu	38.6	32.2	-6.4	-16.6	23.2	34.1	10.9	47.0	13.9	34.4	20.5	147.5
Venezuela (Bolivarian Republic of)	33.3	29.2	-4.1	-12.3	24.8	23.1	-1.7	-6.9	18.3	15.4	-2.9	-15.8
Viet Nam	51.2	42.4	-8.8	-17.2	35	24.1	-10.9	-31.1	23.3	11	-12.3	-52.8
Yemen	128	122.9	-5.1	-4.0	99.5	95	-4.5	-4.5	77	58.4	-18.6	-24.2
Zambia	182.8	171.5	-11.3	-6.2	157.3	144.7	-12.6	-8.0	111	113	2	1.8
Zimbabwe	77.6	73.1	-4.5	-5.8	115	74.9	-40.1	-34.9	79.8	71.8	-8	-10

	**1990 Number of under-five deaths (thousands)**				**2000 Number of under-five deaths (thousands)**				**2010 Number of under-five deaths (thousands)**			
**Country**	**IGME**	**IHME**	**Diff**	**Diff (%)**	**IGME**	**IHME**	**Diff**	**Diff (%)**	**IGME**	**IHME**	**Diff**	**Diff (%)**
Afghanistan	128.5	112.9	-15.6	-12.1	161.0	178.0	17.0	10.6	191.0	154.7	-36.3	-19.0
Albania	3.5	3.2	-0.3	-8.6	1.6	1.4	-0.2	-12.5	0.7	0.6	-0.1	-14.3
Algeria	53.2	44.2	-9.0	-16.9	27.8	21.7	-6.1	-21.9	25.9	14.5	-11.4	-44.0
Andorra	0.0	0.0	0.0	-	0.0	0.0	0.0	-	0.0	0.0	0.0	-
Angola	123.3	127.3	4.0	3.2	130.9	137.5	6.6	5.0	121.1	107.8	-13.3	-11.0
Antigua and Barbuda	0.0	0.0	0.0	-	0.0	0.0	0.0	-	0.0	0.0	0.0	-
Argentina	19.7	20.1	0.4	2.0	13.5	13.5	0.0	0.0	9.8	10.2	0.4	4.1
Armenia	4.4	3.7	-0.7	-15.9	1.2	1.3	0.1	8.3	0.9	0.8	-0.1	-11.1
Australia	2.3	2.4	0.1	4.3	1.5	1.6	0.1	6.7	1.5	1.4	-0.1	-6.7
Austria	0.8	0.9	0.1	12.5	0.4	0.4	0.0	0.0	0.3	0.3	0.0	0.0
Azerbaijan	19.1	15.0	-4.1	-21.5	8.7	8.2	-0.5	-5.7	8.6	7.3	-1.3	-15.1
Bahamas	0.1	0.2	0.1	100.0	0.1	0.1	0.0	0.0	0.1	0.1	0.0	0.0
Bahrain	0.2	0.3	0.1	50.0	0.1	0.2	0.1	100.0	0.2	0.2	0.0	0.0
Bangladesh	527.5	514.1	-13.4	-2.5	299.9	304.3	4.4	1.5	139.7	172.6	32.9	23.6
Barbados	0.1	0.1	0.0	0.0	0.1	0.1	0.0	0.0	0.1	0.0	-0.1	-100.0
Belarus	2.7	3.2	0.5	18.5	1.3	1.6	0.3	23.1	0.6	1.0	0.4	66.7
Belgium	1.1	1.2	0.1	9.1	0.6	0.7	0.1	16.7	0.5	0.5	0.0	0.0

Belize	0.3	0.3	0.0	0.0	0.2	0.2	0.0	0.0	0.1	0.2	0.1	100.0
Benin	37.4	39.3	1.9	5.1	38.4	38.6	0.2	0.5	39.0	34.9	-4.1	-10.5
Bhutan	2.9	3.0	0.1	3.4	1.3	1.4	0.1	7.7	0.8	0.9	0.1	12.5
Bolivia	28.0	26.5	-1.5	-5.4	20.5	19.7	-0.8	-3.9	13.8	14.6	0.8	5.8
Bosnia and Herzegovina	1.3	1.2	-0.1	-7.7	0.5	0.4	-0.1	-20.0	0.3	0.2	-0.1	-33.3
Botswana	2.7	2.6	-0.1	-3.7	4.5	3.1	-1.4	-31.1	2.3	2.3	0.0	0.0
Brazil	209.6	192.8	-16.8	-8.0	134.5	114.3	-20.2	-15.0	54.8	66.3	11.5	21.0
Brunei Darussalam	0.1	0.1	0.0	0.0	0.1	0.1	0.0	0.0	0.1	0.1	0.0	0.0
Bulgaria	2.6	2.0	-0.6	-23.1	1.4	1.2	-0.2	-14.3	1.0	0.8	-0.2	-20.0
Burkina Faso	84.8	90.8	6.0	7.1	100.8	99.0	-1.8	-1.8	120.4	110.9	-9.5	-7.9
Burundi	44.7	47.0	2.3	5.1	38.6	40.3	1.7	4.4	38.4	33.2	-5.2	-13.5
Cambodia	38.2	47.6	9.4	24.6	32.7	35.2	2.5	7.6	16.3	17.2	0.9	5.5
Cameroon	66.4	73.9	7.5	11.3	81.8	83.7	1.9	2.3	93.2	79.8	-13.4	-14.4
Canada	3.2	3.3	0.1	3.1	2.0	2.1	0.1	5.0	2.4	1.9	-0.5	-20.8
Cape Verde	0.8	0.8	0.0	0.0	0.6	0.6	0.0	0.0	0.4	0.3	-0.1	-25.0
Central African Republic	19.2	20.4	1.2	6.3	24.8	25.2	0.4	1.6	23.4	24.1	0.7	3.0
Chad	54.6	58.9	4.3	7.9	67.6	73.1	5.5	8.1	80.1	84.0	3.9	4.9
Chile	5.6	5.5	-0.1	-1.8	2.7	2.8	0.1	3.7	2.2	1.7	-0.5	-22.7
China	1279.5	998.2	-281.3	-22.0	673.8	584.4	-89.4	-13.3	314.6	241.4	-73.2	-23.3
Colombia	32.7	30.3	-2.4	-7.3	24.1	24.4	0.3	1.2	17.6	20.7	3.1	17.6
Comoros	1.8	1.9	0.1	5.6	2.2	1.9	-0.3	-13.6	2.3	1.8	-0.5	-21.7
Congo	10.2	9.5	-0.7	-6.9	11.7	13.3	1.6	13.7	12.7	14.4	1.7	13.4
Congo, Republic of the	311.7	325.4	13.7	4.4	415.1	402.8	-12.3	-3.0	464.6	372.9	-91.7	-19.7
Costa Rica	1.5	1.7	0.2	13.3	1.0	1.1	0.1	10.0	0.7	0.6	-0.1	-14.3
Côte d'Ivoire	75.9	77.8	1.9	2.5	87.5	85.3	-2.2	-2.5	80.5	72.7	-7.8	-9.7
Croatia	0.7	0.7	0.0	0.0	0.4	0.4	0.0	0.0	0.2	0.2	0.0	0.0
Cuba	2.5	2.4	-0.1	-4.0	1.3	1.2	-0.1	-7.7	0.6	0.6	0.0	0.0
Cyprus	0.2	0.1	-0.1	-50.0	0.1	0.1	0.0	0.0	0.1	0.0	-0.1	-100.0
Czech Republic	2.1	1.6	-0.5	-23.8	0.5	0.5	0.0	0.0	0.5	0.4	-0.1	-20.0
Denmark	0.6	0.6	0.0	0.0	0.4	0.4	0.0	0.0	0.3	0.3	0.0	0.0
Djibouti	2.8	2.5	-0.3	-10.7	2.4	2.2	-0.2	-8.3	2.3	1.8	-0.5	-21.7
Dominica	0.0	0.0	0.0	-	0.0	0.0	0.0	-	0.0	0.0	0.0	-
Dominican Republic	13.0	12.4	-0.6	-4.6	8.4	8.2	-0.2	-2.4	5.7	6.3	0.6	10.5
Ecuador	15.4	14.9	-0.5	-3.2	10.1	11.0	0.9	8.9	6.0	6.2	0.2	3.3
Egypt	173.8	152.5	-21.3	-12.3	82.7	78.5	-4.2	-5.1	40.7	45.1	4.4	10.8
El Salvador	10.4	9.8	-0.6	-5.8	5.2	5.1	-0.1	-1.9	2.2	2.2	0.0	0.0
Equatorial Guinea	3.2	3.1	-0.1	-3.1	3.0	3.9	0.9	30.0	2.9	4.8	1.9	65.5
Eritrea	17.8	18.1	0.3	1.7	12.1	13.0	0.9	7.4	11.2	13.5	2.3	20.5
Estonia	0.5	0.5	0.0	0.0	0.2	0.2	0.0	0.0	0.1	0.1	0.0	0.0
Ethiopia	398.4	457.9	59.5	14.9	370.9	364.7	-6.2	-1.7	270.9	259.6	-11.3	-4.2
Fiji	0.6	0.7	0.1	16.7	0.4	0.7	0.3	75.0	0.3	0.5	0.2	66.7
Finland	0.4	0.4	0.0	0.0	0.2	0.2	0.0	0.0	0.2	0.2	0.0	0.0
France	6.4	6.7	0.3	4.7	4.1	4.1	0.0	0.0	3.3	3.2	-0.1	-3.0

Gabon	3.1	3.2	0.1	3.2	3.2	3.3	0.1	3.1	2.9	2.8	-0.1	-3.4
Gambia	6.9	6.9	0.0	0.0	6.8	7.5	0.7	10.3	6.1	6.3	0.2	3.3
Georgia	4.4	4.0	-0.4	-9.1	1.7	2.3	0.6	35.3	1.2	1.4	0.2	16.7
Germany	7.3	7.8	0.5	6.8	4.7	4.1	-0.6	-12.8	2.9	2.8	-0.1	-3.4
Ghana	67.6	69.1	1.5	2.2	62.0	66.2	4.2	6.8	56.6	48.5	-8.1	-14.3
Greece	1.3	1.1	-0.2	-15.4	0.8	0.6	-0.2	-25.0	0.5	0.4	-0.1	-20.0
Grenada	0.1	0.1	0.0	0.0	0.0	0.0	0.0	-	0.0	0.0	0.0	-
Guatemala	25.7	26.4	0.7	2.7	19.2	20.9	1.7	8.9	14.3	15.8	1.5	10.5
Guinea	58.4	60.5	2.1	3.6	58.8	61.8	3.0	5.1	48.5	51.7	3.2	6.6
Guinea-Bissau	8.8	10.7	1.9	21.6	8.5	10.4	1.9	22.4	8.2	9.9	1.7	20.7
Guyana	1.3	1.1	-0.2	-15.4	0.7	0.7	0.0	0.0	0.3	0.5	0.2	66.7
Haiti	38.2	39.4	1.2	3.1	29.1	26.5	-2.6	-8.9	45.4	29.4	-16.0	-35.2
Honduras	10.6	10.5	-0.1	-0.9	7.2	7.4	0.2	2.8	4.8	4.6	-0.2	-4.2
Hungary	2.6	2.0	-0.6	-23.1	1.0	1.0	0.0	0.0	0.6	0.6	0.0	0.0
Iceland	0.0	0.0	0.0	-	0.0	0.0	0.0	-	0.0	0.0	0.0	-
India	3078.3	3106.2	27.9	0.9	2294.0	2317.2	23.2	1.0	1696.3	1699.5	3.2	0.2
Indonesia	403.2	362.0	-41.2	-10.2	240.2	233.3	-6.9	-2.9	151.4	170.8	19.4	12.8
Iran (Islamic Republic of)	121.9	131.0	9.1	7.5	56.3	57.0	0.7	1.2	34.2	32.3	-1.9	-5.6
Iraq	30.2	38.0	7.8	25.8	37.4	37.1	-0.3	-0.8	42.6	39.8	-2.8	-6.6
Ireland	0.5	0.5	0.0	0.0	0.4	0.4	0.0	0.0	0.3	0.3	0.0	0.0
Israel	1.2	1.2	0.0	0.0	0.9	0.9	0.0	0.0	0.7	0.7	0.0	0.0
Italy	5.4	5.3	-0.1	-1.9	2.9	2.9	0.0	0.0	2.2	2.0	-0.2	-9.1
Jamaica	2.2	2.1	-0.1	-4.5	1.7	1.5	-0.2	-11.8	1.3	0.9	-0.4	-30.8
Japan	7.9	7.9	0.0	0.0	5.3	5.3	0.0	0.0	3.5	3.3	-0.2	-5.7
Jordan	4.8	4.3	-0.5	-10.4	4.5	3.9	-0.6	-13.3	3.7	3.6	-0.1	-2.7
Kazakhstan	23.0	19.2	-3.8	-16.5	9.4	11.0	1.6	17.0	12.7	10.3	-2.4	-18.9
Kenya	97.0	98.5	1.5	1.5	127.3	110.8	-16.5	-13.0	122.3	106.0	-16.3	-13.3
Kiribati	0.2	0.2	0.0	0.0	0.1	0.1	0.0	0.0	0.1	0.1	0.0	0.0
Korea, North	16.5	15.8	-0.7	-4.2	23.6	13.4	-10.2	-43.2	11.7	8.3	-3.4	-29.1
Korea, South	4.3	7.3	3.0	69.8	3.1	4.0	0.9	29.0	2.7	1.7	-1.0	-37.0
Kuwait	0.7	0.9	0.2	28.6	0.5	0.7	0.2	40.0	0.6	0.7	0.1	16.7
Kyrgyzstan	9.9	9.5	-0.4	-4.0	5.7	5.6	-0.1	-1.8	5.3	5.8	0.5	9.4
Lao People's Democratic Republic	24.3	24.2	-0.1	-0.4	14.0	16.3	2.3	16.4	7.7	10.1	2.4	31.2
Latvia	0.9	0.7	-0.2	-22.2	0.3	0.3	0.0	0.0	0.2	0.2	0.0	0.0
Lebanon	2.8	2.4	-0.4	-14.3	2.4	1.4	-1.0	-41.7	1.5	0.7	-0.8	-53.3
Lesotho	5.1	5.3	0.2	3.9	8.0	6.2	-1.8	-22.5	5.0	6.3	1.3	26.0
Liberia	20.8	25.3	4.5	21.6	19.4	18.9	-0.5	-2.6	15.5	15.8	0.3	1.9
Libyan Arab Jamahiriya	4.6	4.7	0.1	2.2	3.2	2.9	-0.3	-9.4	2.4	2.1	-0.3	-12.5
Lithuania	1.0	0.9	-0.1	-10.0	0.4	0.4	0.0	0.0	0.2	0.2	0.0	0.0
Luxembourg	0.0	0.0	0.0	-	0.0	0.0	0.0	-	0.0	0.0	0.0	-
Macedonia	1.3	0.9	-0.4	-30.8	0.4	0.5	0.1	25.0	0.3	0.2	-0.1	-33.3
Madagascar	78.1	75.4	-2.7	-3.5	62.1	61.6	-0.5	-0.8	44.1	43.8	-0.3	-0.7
Malawi	92.1	88.3	-3.8	-4.1	77.0	72.6	-4.4	-5.7	55.9	65.0	9.1	16.3

Malaysia	8.9	8.7	-0.2	-2.2	5.9	5.2	-0.7	-11.9	3.5	2.9	-0.6	-17.1
Maldives	0.9	0.7	-0.2	-22.2	0.3	0.2	-0.1	-33.3	0.1	0.1	0.0	0.0
Mali	102.2	104.2	2.0	2.0	110.4	116.5	6.1	5.5	120.5	111.3	-9.2	-7.6
Malta	0.1	0.1	0.0	0.0	0.0	0.0	0.0	-	0.0	0.0	0.0	-
Marshall Islands	0.1	0.1	0.0	0.0	0.1	0.1	0.0	0.0	0.0	0.1	0.1	-
Mauritania	9.7	8.5	-1.2	-12.4	11.0	9.9	-1.1	-10.0	12.6	9.3	-3.3	-26.2
Mauritius	0.6	0.5	-0.1	-16.7	0.4	0.4	0.0	0.0	0.2	0.2	0.0	0.0
Mexico	115.3	94.6	-20.7	-18.0	62.6	57.6	-5.0	-8.0	36.6	41.7	5.1	13.9
Micronesia (Fed. States of)	0.2	0.2	0.0	0.0	0.2	0.1	-0.1	-50.0	0.1	0.0	-0.1	-100.0
Moldova	3.2	2.5	-0.7	-21.9	1.4	1.1	-0.3	-21.4	0.9	0.6	-0.3	-33.3
Mongolia	8.0	6.7	-1.3	-16.3	3.2	2.8	-0.4	-12.5	2.2	2.1	-0.1	-4.5
Montenegro	0.2	0.2	0.0	0.0	0.1	0.1	0.0	0.0	0.1	0.1	0.0	0.0
Morocco	60.5	56.7	-3.8	-6.3	34.7	32.0	-2.7	-7.8	22.7	21.6	-1.1	-4.8
Mozambique	123.0	135.2	12.2	9.9	132.6	133.1	0.5	0.4	114.3	121.7	7.4	6.5
Myanmar	120.0	115.0	-5.0	-4.2	83.5	70.7	-12.8	-15.3	56.0	66.6	10.6	18.9
Namibia	3.7	3.8	0.1	2.7	4.2	3.7	-0.5	-11.9	2.4	3.5	1.1	45.8
Nepal	99.2	100.5	1.3	1.3	67.2	64.1	-3.1	-4.6	34.5	37.9	3.4	9.9
Netherlands	1.6	1.7	0.1	6.2	1.2	1.3	0.1	8.3	0.8	0.8	0.0	0.0
New Zealand	0.6	0.6	0.0	0.0	0.4	0.4	0.0	0.0	0.4	0.4	0.0	0.0
Nicaragua	9.8	10.1	0.3	3.1	5.9	5.8	-0.1	-1.7	3.9	4.2	0.3	7.7
Niger	123.8	125.7	1.9	1.5	113.8	125.3	11.5	10.1	100.0	119.1	19.1	19.1
Nigeria	871.6	906.4	34.8	4.0	916.4	942.4	26.0	2.8	860.6	885.3	24.7	2.9
Norway	0.5	0.5	0.0	0.0	0.3	0.3	0.0	0.0	0.2	0.2	0.0	0.0
Occupied Palestinian Territory	3.8	3.9	0.1	2.6	3.5	4.1	0.6	17.1	3.0	3.1	0.1	3.3
Oman	3.4	2.8	-0.6	-17.6	1.2	0.6	-0.6	-50.0	0.5	0.5	0.0	0.0
Pakistan	551.1	503.7	-47.4	-8.6	466.4	429.7	-36.7	-7.9	423.1	398.9	-24.2	-5.7
Panama	2.1	1.7	-0.4	-19.0	1.8	1.6	-0.2	-11.1	1.4	1.2	-0.2	-14.3
Papua New Guinea	12.3	12.7	0.4	3.3	13.7	13.9	0.2	1.5	12.5	13.8	1.3	10.4
Paraguay	6.9	5.3	-1.6	-23.2	5.2	4.2	-1.0	-19.2	3.7	3.5	-0.2	-5.4
Peru	50.2	46.2	-4.0	-8.0	24.9	25.0	0.1	0.4	11.4	15.1	3.7	32.5
Philippines	119.9	108.1	-11.8	-9.8	91.4	88.1	-3.3	-3.6	66.1	64.5	-1.6	-2.4
Poland	9.0	10.0	1.0	11.1	3.6	3.6	0.0	0.0	2.5	2.6	0.1	4.0
Portugal	1.6	1.6	0.0	0.0	0.8	0.8	0.0	0.0	0.4	0.4	0.0	0.0
Qatar	0.2	0.2	0.0	0.0	0.1	0.2	0.1	100.0	0.2	0.2	0.0	0.0
Romania	15.3	10.2	-5.1	-33.3	7.4	5.1	-2.3	-31.1	3.1	2.9	-0.2	-6.5
Russian Federation	60.0	56.1	-3.9	-6.5	32.8	29.3	-3.5	-10.7	19.6	23.3	3.7	18.9
Rwanda	52.5	48.5	-4.0	-7.6	59.0	47.7	-11.3	-19.2	37.6	32.8	-4.8	-12.8
Saint Lucia	0.1	0.1	0.0	0.0	0.1	0.0	-0.1	-100.0	0.1	0.0	-0.1	-100.0
Saint Vincent and the Grenadines	0.1	0.1	0.0	0.0	0.0	0.1	0.1	-	0.0	0.0	0.0	-
Samoa	0.1	0.1	0.0	0.0	0.1	0.1	0.0	0.0	0.1	0.1	0.0	0.0
Sao Tome and Principe	0.4	0.5	0.1	25.0	0.4	0.4	0.0	0.0	0.4	0.3	-0.1	-25.0
Saudi Arabia	24.5	12.8	-11.7	-47.8	15.1	10.5	-4.6	-30.5	11.5	9.9	-1.6	-13.9
Senegal	41.7	43.9	2.2	5.3	43.1	46.2	3.1	7.2	34.0	36.5	2.5	7.4

Serbia	4.1	1.8	-2.3	-56.1	1.5	1.0	-0.5	-33.3	0.8	0.5	-0.3	-37.5
Seychelles	0.0	0.0	0.0	-	0.0	0.0	0.0	-	0.0	0.0	0.0	-
Sierra Leone	44.7	41.8	-2.9	-6.5	39.8	39.7	-0.1	-0.3	39.1	28.9	-10.2	-26.1
Singapore	0.3	0.4	0.1	33.3	0.2	0.2	0.0	0.0	0.1	0.1	0.0	0.0
Slovakia	1.5	1.2	-0.3	-20.0	0.6	0.5	-0.1	-16.7	0.5	0.4	-0.1	-20.0
Slovenia	0.2	0.2	0.0	0.0	0.1	0.1	0.0	0.0	0.1	0.1	0.0	0.0
Solomon Islands	0.5	0.5	0.0	0.0	0.5	0.5	0.0	0.0	0.4	0.5	0.1	25.0
Somalia	52.1	54.7	2.6	5.0	57.7	47.7	-10.0	-17.3	69.8	40.7	-29.1	-41.7
South Africa	63.9	65.2	1.3	2.0	83.9	39.5	-44.4	-52.9	58.1	55.7	-2.4	-4.1
Spain	4.5	3.8	-0.7	-15.6	2.5	2.1	-0.4	-16.0	2.4	1.9	-0.5	-20.8
Sri Lanka	10.8	10.5	-0.3	-2.8	7.2	5.7	-1.5	-20.8	6.3	4.1	-2.2	-34.9
Sudan	126.5	121.1	-5.4	-4.3	140.0	127.0	-13.0	-9.3	143.1	117.3	-25.8	-18.0
Suriname	0.5	0.5	0.0	0.0	0.4	0.4	0.0	0.0	0.3	0.4	0.1	33.3
Swaziland	3.6	2.6	-1.0	-27.8	3.8	3.4	-0.4	-10.5	2.6	3.5	0.9	34.6
Sweden	0.8	0.8	0.0	0.0	0.3	0.4	0.1	33.3	0.3	0.3	0.0	0.0
Switzerland	0.6	0.7	0.1	16.7	0.4	0.4	0.0	0.0	0.4	0.3	-0.1	-25.0
Syrian Arab Republic	16.8	15.3	-1.5	-8.9	10.4	8.7	-1.7	-16.3	7.8	5.1	-2.7	-34.6
Tajikistan	25.0	21.2	-3.8	-15.2	17.9	14.6	-3.3	-18.4	11.8	8.7	-3.1	-26.3
Tanzania	165.6	167.9	2.3	1.4	173.9	172.8	-1.1	-0.6	133.0	151.3	18.3	13.8
Thailand	34.7	24.7	-10.0	-28.8	16.1	13.4	-2.7	-16.8	10.9	8.8	-2.1	-19.3
Timor-Leste	4.5	3.6	-0.9	-20.0	3.9	3.8	-0.1	-2.6	2.4	2.4	0.0	0.0
Togo	21.9	22.5	0.6	2.7	21.4	21.5	0.1	0.5	19.3	21.1	1.8	9.3
Tonga	0.1	0.1	0.0	0.0	0.1	0.1	0.0	0.0	0.0	0.1	0.1	-
Trinidad and Tobago	0.9	0.8	-0.1	-11.1	0.6	0.6	0.0	0.0	0.5	0.5	0.0	0.0
Tunisia	11.4	11.0	-0.4	-3.5	4.5	4.5	0.0	0.0	3.0	2.6	-0.4	-13.3
Turkey	106.8	101.2	-5.6	-5.2	57.3	55.0	-2.3	-4.0	23.7	40.7	17.0	71.7
Turkmenistan	13.1	13.4	0.3	2.3	6.9	7.5	0.6	8.7	5.7	3.4	-2.3	-40.4
Uganda	143.5	139.2	-4.3	-3.0	157.4	155.0	-2.4	-1.5	141.5	137.8	-3.7	-2.6
UK	7.1	7.4	0.3	4.2	4.6	4.4	-0.2	-4.3	4.3	4.1	-0.2	-4.7
Ukraine	14.9	13.8	-1.1	-7.4	7.1	7.9	0.8	11.3	6.9	6.2	-0.7	-10.1
United Arab Emirates	1.0	0.7	-0.3	-30.0	0.6	0.4	-0.2	-33.3	0.6	0.3	-0.3	-50.0
Uruguay	1.4	1.3	-0.1	-7.1	0.9	0.9	0.0	0.0	0.5	0.6	0.1	20.0
USA	43.9	45.3	1.4	3.2	31.8	33.0	1.2	3.8	32.3	31.5	-0.8	-2.5
Uzbekistan	56.1	42.2	-13.9	-24.8	34.9	32.0	-2.9	-8.3	31.5	24.3	-7.2	-22.9
Vanuatu	0.2	0.2	0.0	0.0	0.1	0.2	0.1	100.0	0.1	0.2	0.1	100.0
Venezuela (Bolivarian Republic of)	19.2	16.4	-2.8	-14.6	14.3	13.2	-1.1	-7.7	10.9	9.2	-1.7	-15.6
Viet Nam	98.7	85.0	-13.7	-13.9	42.1	34.0	-8.1	-19.2	34.5	16.0	-18.5	-53.6
Yemen	74.6	75.4	0.8	1.1	68.4	68.3	-0.1	-0.1	68.7	52.8	-15.9	-23.1
Zambia	60.2	56.9	-3.3	-5.5	67.9	63.5	-4.4	-6.5	60.3	65.0	4.7	7.8
Zimbabwe	29.2	28.2	-1.0	-3.4	42.8	28.3	-14.5	-33.9	29.1	26.7	-2.4	-8.2
